# Charge-switching ionizable lipids lower the toxicity of lipid nanoparticles

**DOI:** 10.1038/s41565-026-02262-6

**Published:** 2026-09-08

**Authors:** Dengpan Liang, Yalin Qi, Hesong Han, Negar Ahmadian, Kewa Gao, Kaycee Sapasap, Yuxi Zhang, Silin Guo, Atip Lawanprasert, Sopida Pimcharoen, Sheng Zhao, Milan T. Del Buono, He Xia, Zoe O. Enders, Benjamin W. Burgstone, Antonino Calio, Neel Dankar, Bingwei Lu, Lei S. Qi, Aijun Wang, Niren Murthy

**Affiliations:** 1 Innovative Genomics Institute, University of California, Berkeley, Berkeley, CA USA; 2 Department of Bioengineering, University of California, Berkeley, Berkeley, CA USA; 3 Department of Surgery, School of Medicine, University of California, Davis, Sacramento, CA USA; 4 Shriners Children’s Northern California, Sacramento, CA USA; 5 Center for Bioengineering in Medicine, School of Medicine, University of California, Davis, Sacramento, CA USA; 6 Department of Bioengineering, Stanford University, Stanford, CA USA; 7 College of Bioengineering, Chongqing University, Chongqing, China; 8 Molecular Biophysics and Integrated Bioimaging Division, Lawrence Berkeley National Laboratory, CA USA; 9 Department of Pathology, School of Medicine, Stanford University, Stanford, CA USA; 10 Sarafan ChEM-H, Stanford University, Stanford, CA USA; 11 Chan Zuckerberg Biohub, San Francisco, CA USA; 12 Department of Biomedical Data Science, School of Medicine, Stanford University, Stanford, CA USA; 13 Department of Biomedical Engineering, University of California, Davis, Davis, CA USA

**Keywords:** Drug delivery, RNA nanotechnology

## Abstract

Lipid nanoparticles (LNPs) have great potential as nucleic acid delivery vehicles; however, they trigger the production of inflammatory cytokines, which limits their medical applications. Developing non-inflammatory LNPs is challenging because the LNP’s ionizable lipid and the process of endosomal disruption are the major sources of LNP toxicity but are also essential for delivering nucleic acids. Here we demonstrate that ionizable lipids containing a carboxylic acid and an amine (termed S-lipid) switch their charged state between the pHs of 7.4 and 4.0, allowing them to generate LNPs (termed switchable nanoparticles) that efficiently encapsulate nucleic acid and trigger endosomal release without activation of the TLR4, complement, galectin-8 and platelet activating factor signalling pathways. Finally, we demonstrate that switchable nanoparticles are better at treating lipopolysaccharide-induced acute lung injury than traditional LNPs because they do not exacerbate pre-existing inflammation. Collectively, these results demonstrate that negatively charged ionizable lipids can mitigate the toxicity of LNPs.

## Abstract (web summary)

Charge-switching S-lipids generate S-lipid nanoparticles that are neutral or negative at physiological pH but become cationic in acidic endosomes, enabling nucleic acid delivery with reduced inflammation compared with conventional LNPs.

## Main

Lipid nanoparticle (LNP)–mRNA complexes have the transfection efficiency and tissue tropism needed to treat numerous diseases; however, their clinical translation beyond vaccines has been slow owing to toxicity^1–4^. LNP–mRNA complexes induce toxicity by activating multiple inflammatory pathways, such as the complement cascade, the TLR4 signalling pathway and the galectin signalling pathway^1,5,6^. In addition, LNPs cause inflammation by triggering the release of platelet activating factor (PAF). Finally, LNP-mediated toxicity is exacerbated in patients with pre-existing inflammation^7–9^, which is concerning because a large fraction of the patient population that need an LNP-based therapy have pre-existing inflammation^10–13^. The toxicity and inflammation caused by LNP–nucleic acid complexes prevents them from being administered chronically^14,15^ or at high doses^16^ and limits their medical applications^17–19^. LNPs that do not trigger inflammation have the potential to accelerate the clinical development of nucleic acid-based therapeutics and expand their therapeutic applications^17,20^.

However, developing LNPs that do not cause inflammation has been challenging because the positive charge of the LNP’s ionizable lipid and its ability to disrupt endosomes are the major sources of LNP toxicity^21–24^ but are also essential for delivering mRNA. In particular, the cationic charge of the ionizable lipid is needed for LNPs to bind mRNA^25^, generate the proper protein corona^26^ and trigger endosomal release^27^; however, it also causes the activation of complement, TLR4 and various other inflammatory pathways^1,5,28^. In addition, LNPs trigger endosomal release of mRNA by disrupting endosomes, which also causes inflammation by activating the galectin signalling pathway and by stimulating the release of PAF^6,21^. Current LNPs consequently have a trade-off between toxicity and mRNA delivery efficiency, and resolving this dilemma is critical for expanding the therapeutic applications of nucleic acids.

**Fig. 1:**
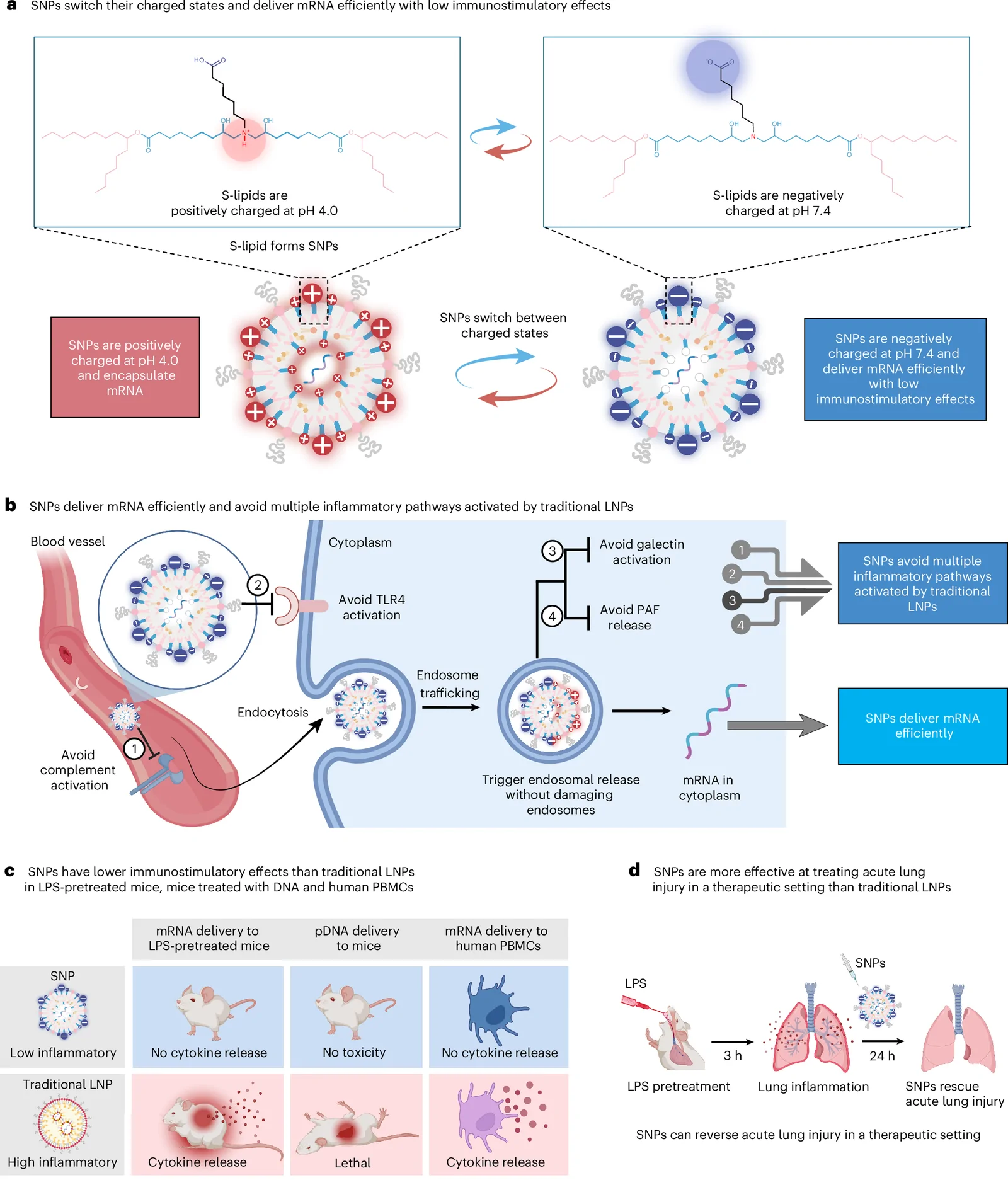
SNPs deliver mRNA and DNA efficiently without causing inflammation. **a**, SNPs contain an ionizable lipid that has a single amine and carboxylic acid in its head group, termed S-lipid, and switch their charged state between the pHs of 4.0 and 7.4. The charge-switching ability of the SNPs allows them to deliver nucleic acids efficiently without triggering inflammation. **b**, SNPs deliver mRNA and pDNA efficiently and do not activate the inflammatory pathways commonly activated by traditional LNPs. SNPs are negatively charged at pH 7.4 and consequently do not activate complement or TLR4. SNPs can trigger endosomal release without activating galectin-8 or triggering the release of PAF. **c**, SNPs have lower immunostimulatory effects than traditional LNPs in LPS-pretreated mice, in mice treated with LNPs containing pDNA and in human PBMCs. **d**, SNPs containing anti-inflammatory mRNA are more effective at treating mice with LPS-induced acute lung injury than traditional LNPs. Illustrations created in BioRender; **c**,**d**, Sharma, R. https://biorender.com/l8enkxr (2026); **b**, Sharma, R. https://biorender.com/6toqakn (2026); **c**, Sharma, R. https://biorender.com/3irfxvl (2026); **c**, Sharma, R. https://biorender.com/z9j4osz (2026).

In this Article, we demonstrate that ionizable lipids that are negatively charged at pH 7.4 (termed switchable lipids (S-lipids)) generate LNPs (termed switchable nanoparticles (SNPs)) that deliver mRNA and plasmid DNA (pDNA) efficiently with low toxicity and can resolve the trade-off between nucleic acid delivery efficiency and LNP toxicity. The chemical structure of a representative S-lipid is shown in Fig. 1, it contains a head group that has a carboxylic acid and tertiary amine and switches its charged state between the pHs of 4.0 and 7.4. In addition, the S-lipids have hydrophobic tails that generate LNPs that have a viscous core and trigger endosomal release efficiently without damaging the endosomal membrane. The low toxicity of the SNPs arises from its head group, which is negatively charged at pH 7.4, and its ability to trigger endosomal release efficiently without damaging the endosomal membrane. In addition, the S-lipid is positively charged at pH 4.0 and has branched hydrophobic tails, and these features allow it to encapsulate nucleic acids efficiently and trigger endosomal release efficiently. Collectively, these molecular features allow the S-lipids to generate SNPs that deliver mRNA and pDNA efficiently without triggering the TLR4, complement, galectin-8 and PAF inflammatory signalling pathways, which are ubiquitously activated by traditional LNPs.

The ability of the SNPs to deliver mRNA and pDNA efficiently with low toxicity was demonstrated in a variety of cell and animal models. For example, SNPs delivered mRNA and pDNA to mice and human peripheral blood mononuclear cells (PBMCs) as efficiently as traditional LNPs but did not trigger cytokine production in either lipopolysaccharide (LPS)-pretreated mice or human PBMCs. By contrast, all of the traditional LNPs investigated in this report caused large increases in cytokine production in both of these models. SNPs were also able to deliver nanoplasmid DNA to mice efficiently without causing acute toxicity, whereas traditional LNPs that delivered nanoplasmids caused acute toxicity and death within 24 h. Finally, SNPs containing IL-22 mRNA were more effective at treating LPS-induced acute lung injury than traditional LNPs in a therapeutic setting, because they do not exacerbate pre-existing inflammation. Collectively, these results demonstrate that it is possible to generate LNPs that can deliver nucleic acids with low toxicity by engineering the ionizable lipid head group to be negatively charged at pH 7.4.

## High-throughput synthesis identifies S-lipids that efficiently deliver mRNA in vitro

**Fig. 2:**
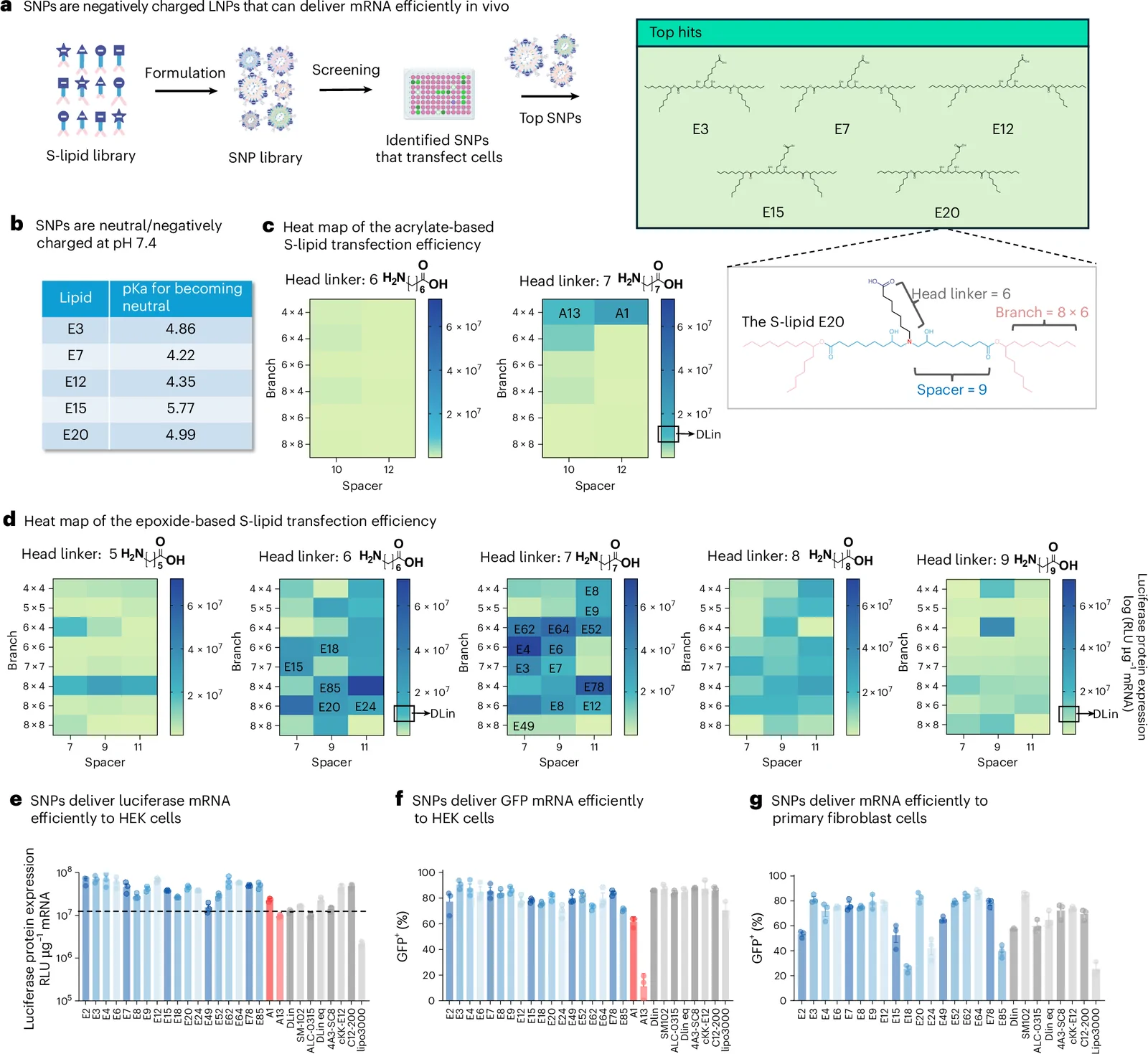
SNPs are negatively charged at pH 7.4 and can deliver mRNA efficiently in vitro. **a**, A library of 144 S-lipids was synthesized, formulated into SNPs and screened in vitro for their ability to deliver mRNA. Chemical structures of five of the top-performing S-lipids are shown. **b**, The p*K*_a_ values of the SNPs for becoming neutral were measured with the R18 dye and are between 4.2 and 5.8. The SNPs are negatively charged at pH 7.4. **c**,**d**, A heat map of the luciferase mRNA transfection efficiency of acrylate-based (**c**) and epoxide-based (**d**) SNPs is shown. The S-lipids in these SNPs had different head-linker lengths (5–9 carbons), branch lengths (for example, 4 × 4 to 8 × 6) and spacer lengths (for example, 7, 9 or 11 carbons). HEK cells were treated with luciferase mRNA-loaded SNPs or MC3-DLin LNPs (500 ng ml^−1^ mRNA) for 24 h (*n* = 3 biologically independent replicates). Heat maps are colour-coded by performance; a darker blue indicates greater transfection. The black box represents the transfection efficiency of MC3-DLin LNPs. **e**, Top-performing S-lipids identified from the screen in **d** were purified and formulated into SNPs to test luciferase mRNA transfection on HEK cells (500 ng ml^−1^ mRNA). Several SNPs delivered luciferase mRNA to HEK293 cells better than MC3-DLin LNPs (*n* = 3 biological independent replicates). **f**,**g**, Several of the SNPs also delivered GFP mRNA (500 ng ml^−1^ mRNA) to HEK293 cells (**f**) and mouse fibroblasts (**g**) more efficiently than traditional LNPs (*n* = 3 biological independent replicates). Schematic in **a** created with BioRender; Sharma, R. https://biorender.com/3irfxvl (2026). Source data

We synthesized a library of 144 S-lipids and measured their ability to deliver luciferase mRNA into HEK cells. Details about the design and synthesis of our S-lipid library are described in the Supplementary Information on page S8. Heat maps of the cell screening results are shown in Fig. 2c,d and demonstrate that the head group of the S-lipid needs to have a 6–8 carbon linker between its amine and carboxyl group to deliver mRNA efficiently (Supplementary Fig. 66). In addition, the S-lipids also need to have branched hydrophobic tails and an internal spacer to function properly, and lipids with these modifications performed up to 9,500 times better than those with linear tails (Supplementary Figs. 69 and 70). Finally, epoxide-based S-lipids were more efficient at transfecting cells than their acrylate analogues (Fig. 2c,d).

We identified the top 18 S-lipids from the S-lipid screen described above and purified them and further verified their transfection activity on HEK cells and primary fibroblasts. Figure 2e demonstrates that SNPs can deliver luciferase mRNA to HEK cells efficiently, and Fig. 2f,g demonstrate that SNPs can deliver GFP mRNA to HEK cells and primary mouse fibroblasts with high efficiency. Notably, 11 out of the 18 S-lipids (61.1%) generated SNPs that transfected fibroblast cells as efficiently as LNPs made from the most efficient ionizable lipids, such as cKK-E12 and SM-102. We also characterized the biophysical properties of the top SNP hits and observed that they had p*K*_a_ values between 4.2 and 5.8 for becoming zwitterionic (Fig. 2b) and had a zeta potential that was more negative than LNPs made from MC3-DLin (Supplementary Figs. 79 and 80). Collectively, these results demonstrate it is possible to develop ionizable lipids that are negatively charged at pH 7.4 that deliver mRNA efficiently.

## SNPs can deliver nucleic acids efficiently in vivo with low toxicity

**Fig. 3:**
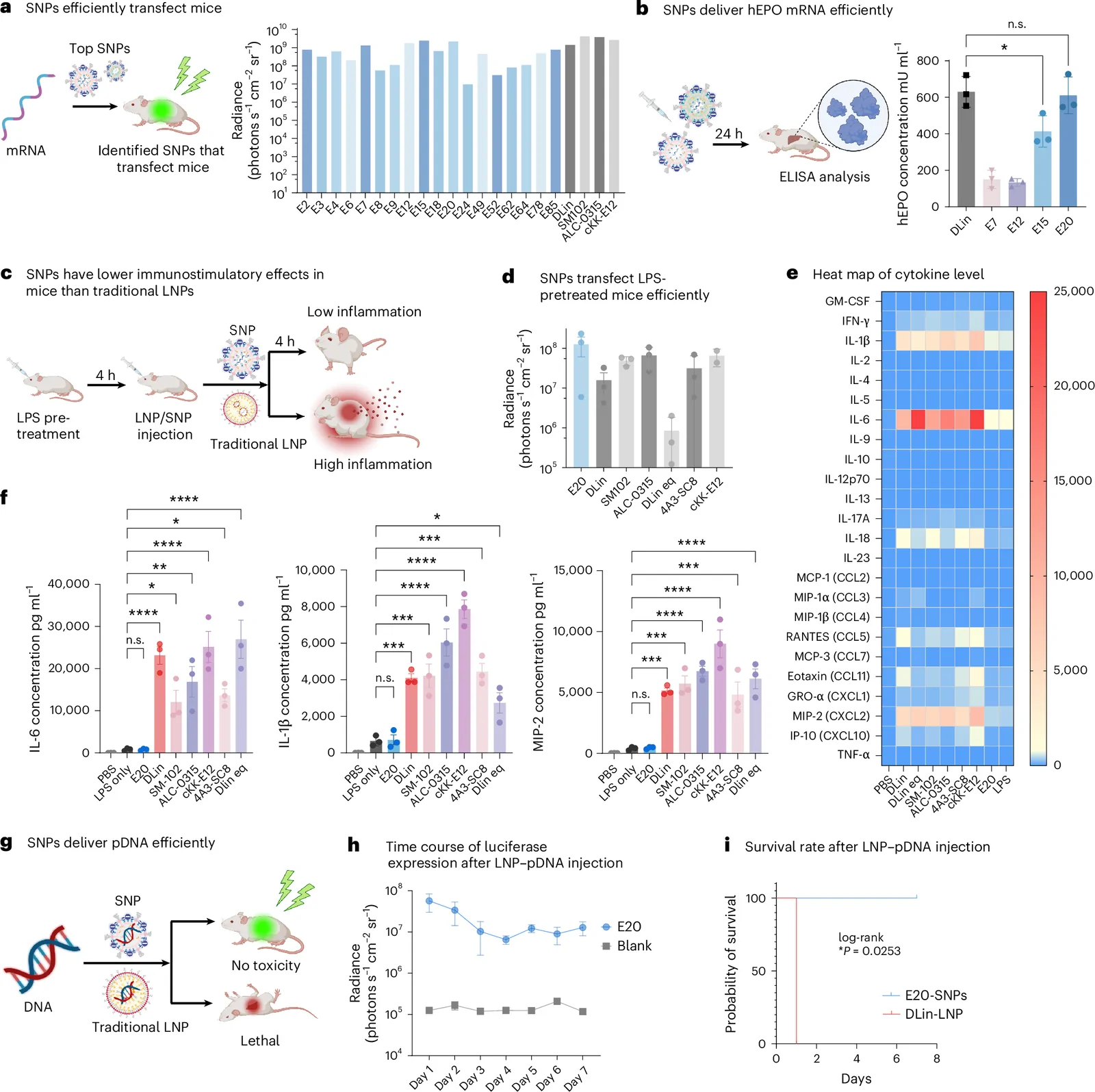
SNPs efficiently deliver mRNA and pDNA in vivo without exacerbating inflammation. **a**, Top SNPs were evaluated in vivo for luciferase mRNA delivery and compared against MC3-DLin LNPs (0.25 mg kg^−1^ mRNA dose, *n* = 2 biological independent replicates). Several SNPs delivered luciferase mRNA to mice as efficiently as MC3-DLin LNPs, based on whole-body IVIS imaging. (**b**) SNPs efficiently deliver hEPO mRNA in vivo. Mice were intravenously injected with SNPs or MC3-DLin LNPs containing hEPO mRNA (0.1 mg kg^−1^ mRNA dose, *n* = 3 biological independent replicates). Blood samples were collected 24 h post injection and analysed by ELISA. **c**, E20-SNPs are substantially less immunostimulatory than traditional LNPs in LPS-pretreated mice. Mice were predosed with 1.0 mg kg^−1^ LPS and, 4 h later, were treated with SNPs or traditional LNPs containing luciferase mRNA (1 mg kg^−1^). The LNP DLin eq was formulated using the same formulation parameters as the SNPs, except that the S-lipid was replaced with MC3-DLin. **d**, The E20-SNPs delivered luciferase mRNA to LPS-pretreated mice more efficiently than LNPs made with cationic ionizable lipids (*n* = 3 biological independent replicates). **e**, Blood samples in the LPS-treated mice were collected 4 h post injection of LNPs or SNPs and were analysed for cytokine levels by Luminex (*n* = 3 biologically independent replicates). **f**, Traditional LNPs induced the production of IL-6, IL-1β and MIP-2, whereas E20-SNPs did not (*n* = 3 biological independent replicates). **g**, Mice injected with E20-SNP–pDNA had reduced toxicity in comparison with mice injected with MC3-DLin LNP–pDNA (1 mg kg^−1^ nanoplasmid DNA dose, *n* = 3 biological independent replicates). **h**, Mice treated with E20-SNP–pDNA (1 mg kg^−1^ pDNA) expressed luciferase throughout the 7-day observation period (*n* = 3 biologically independent replicates). **i**, All mice treated with MC3-DLin LNP–pDNA died within 24 h, whereas mice treated with E20-SNP–pDNA survived and were healthy. Statistical differences were determined using a one-way ANOVA followed by Dunnett’s test in **b** and **f**. Survival curves in (**i**) were analysed using the Log-rank (Mantel–Cox) test. *P < 0.05, **P < 0.01, ***P < 0.001, ****P < 0.0001 for **b** and **f**. Data are presented as mean ± s.e.m. Schematics in **a**–**c**, **g** created in BioRender; Sharma, R. https://biorender.com/3irfxvl (2026); Sharma, R. https://biorender.com/z9j4osz (2026); Sharma, R. https://biorender.com/l8enkxr (2026). Source data

We investigated whether SNPs could deliver mRNA in vivo in mice. The top 18 SNPs from the cell experiments were investigated for their ability to deliver luciferase mRNA in vivo and were compared against a panel of benchmark LNPs. Figure 3a and Supplementary Fig. 86 demonstrate that SNPs can deliver mRNA in vivo efficiently. For example, five SNPs delivered luciferase mRNA in vivo with an efficiency equalling or surpassing MC3-DLin LNPs, and several of them generated signals comparable to LNPs made from second- and third-generation ionizable lipids, such as SM-102. In addition, SNPs had a strong tropism for the liver, similar to traditional LNPs, and ten SNPs generated more than 80% of their luciferase signal from the liver (Supplementary Fig. 88). The remaining eight SNPs showed a tendency towards spleen tropism.

On the basis of these results, we further investigated if SNPs could be used as a platform for developing protein replacement therapies that targeted liver, using human erythropoietin (hEPO) as a representative therapeutic mRNA. The top four SNPs from the luciferase study, E7, E12, E15 and E20 were formulated with hEPO mRNA and administered to mice at a 0.1 mg kg^−1^ mRNA dose, and their ability to generate therapeutic levels of hEPO protein in the blood was evaluated against MC3-DLin LNPs. Figure 3b demonstrates that SNPs can efficiently deliver hEPO mRNA in vivo. In particular, SNPs made from E20 generated blood hEPO concentrations of 600 mU ml^−1^, which is 100 times greater than the therapeutic threshold for hEPO and was also equivalent to the hEPO serum concentrations generated from MC3-DLin LNPs^29,30^.

Next, we investigated the immunostimulatory effects of E20-SNPs in LPS-pretreated mice, which is an inflammation exacerbation model that mimics administering LNPs to patients with pre-existing inflammation^10–13^. The immunostimulatory effects of the E20-SNP were compared against benchmark LNPs, in particular MC3-DLin, SM-102, ALC-0315, 4A3-SC8 and cKK-E12 LNPs. Mice were treated with LPS (1 mg kg^−1^) and, 4 h later, were injected with either SNPs or LNPs (1 mg kg^−1^ mRNA) and analysed 4 and 24 h later for cytokines in their blood. Figure 3e,f and Supplementary Figs. 95–97 demonstrate that E20-SNPs do not exacerbate inflammation in LPS-pretreated mice, whereas traditional LNPs caused high levels of inflammation exacerbation. For example, LNPs made from MC3-DLin, cKK-E12 and SM-102 caused large increases in the serum concentrations of IL-6, IL-1β and MIP-2. By contrast, E20-SNPs did not increase the concentration of these cytokines, and it was the only LNP investigated that did not exacerbate pre-existing inflammation. In addition, E20-SNP-treated mice appeared healthy, similar to untreated controls, whereas those treated with LNPs exhibited signs of distress and had a hunched posture, reduced mobility and narrowed eyes.

**Extended Data Fig. 2:**
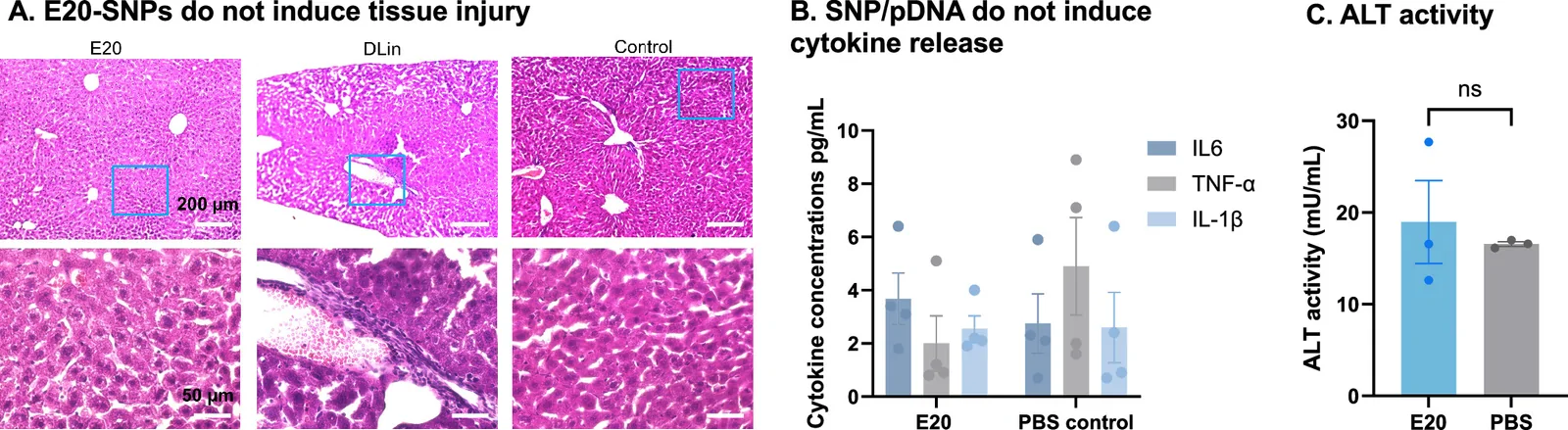
E20-SNP/pDNA complexes are not toxic to mice. (**a**) Histological analysis shows that E20-SNP treated mice do not have liver damage. (**b**) SNPs deliver DNA to mice with minimal cytokine release (n = 3 biologically independent replicates). (**c**) Serum alanine aminotransferase (ALT) analysis showed no evidence of liver damage in E20-SNP–treated mice 7 days after injection (n = 3 biologically independent replicates). ALT activity (**c**) was analyzed using a one-tailed unpaired t-test. *P < 0.05, **P < 0.01, ***P < 0.001, ****P < 0.0001 for **b** and **c**. Data are presented as mean ± SEM. Source data

Finally, we investigated if SNPs could lower the toxicity associated with pDNA delivery. Mice were injected with 1 mg kg^−1^ pDNA encapsulated in either E20-SNPs or MC3-DLin LNPs and monitored for survival and luciferase expression. All mice treated with MC3-DLin LNPs died within 24 h, consistent with previously reported DNA–LNP toxicity^31,32^. By contrast, mice treated with E20-SNP–pDNA showed no signs of distress and survived the entire 7-day observation period (Fig. 3i and Extended Data Fig. 2a–c). In addition, mice treated with E20-SNPs also generated high levels of luciferase luminescence, demonstrating that the low toxicity of the SNPs was not due to low transfection activity (Fig. 3h).

## SNPs have a unique combination of high transfection efficiency and low immunostimulatory effects in human PBMCs

The mRNA delivery efficiency and immunostimulatory effects of the SNPs were also evaluated in human PBMCs. Human PBMCs were selected as an additional test bed for the SNPs because they are the primary source of cytokines produced in response to systemic LNP administration^9^. In addition, PBMCs have a well-established in vitro-in vivo correlation and are a widely used model for assessing the immunostimulatory effects of nanoparticles^9,33,34^. PBMCs from healthy donors were isolated and treated with either E20-SNPs or a panel of traditional LNPs containing luciferase mRNA, at three different doses, and their immunostimulatory effects and transfection efficiency were determined and compared.

**Fig. 4:**
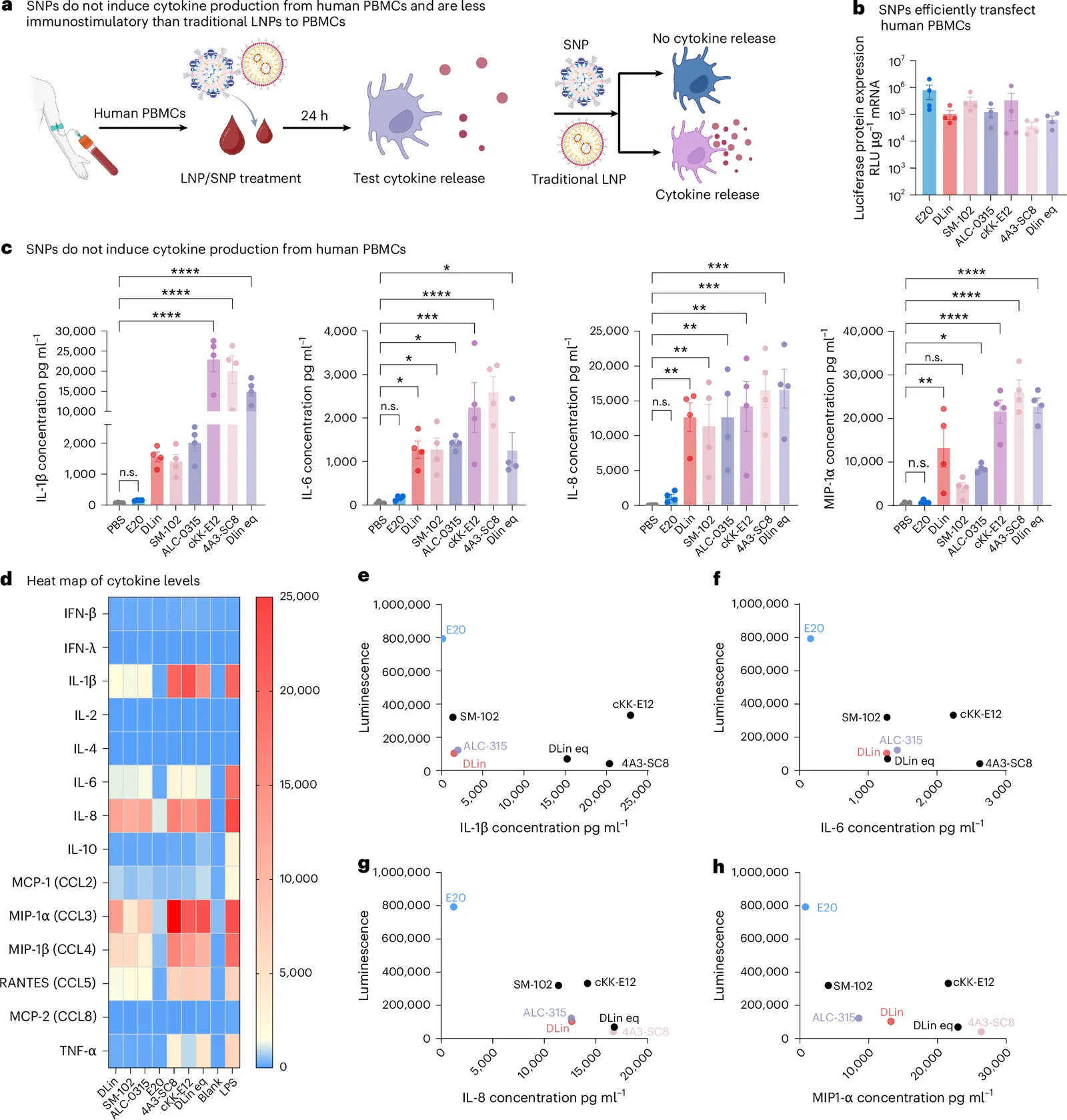
SNPs have lower immunostimulatory effects than traditional LNPs in human PBMCs. **a**, SNPs or LNPs containing luciferase mRNA were incubated with human PBMCs at 2,000 ng ml^−1^ for 24 h. **b**, SNPs efficiently transfect human PBMCs (*n* = 4 healthy donors). The LNP DLin eq was formulated using the same formulation parameters as the SNPs, except that the S-lipid was replaced with MC3-DLin. LPS was used as a positive control. **c**, SNPs do not induce cytokine production from human PBMCs (*n* = 4 healthy donors), whereas traditional LNPs induce large amounts of cytokine release (*n* = 4 healthy donors). Human PBMCs (*n* = 4 healthy donors) were treated with E20-SNPs or traditional LNPs (2,000 ng ml^−1 ^mRNA) and analysed for the release of IL-1β, IL-6, IL-8 and MIP-1α (*n* = 4 healthy donors). **d**, A heat map of cytokine levels in PBMCs (*n* = 4 healthy donors) treated with E20-SNPs or traditional LNPs (2,000 ng ml^−1 ^mRNA) for 24 h. E20-SNPs did not produce inflammatory cytokines. By contrast, traditional LNPs produced large amounts of inflammatory cytokines. **e**–**h**, SNPs have a unique combination of high mRNA delivery efficiency and low immunostimulatory effects (*n* = 4 healthy donors); luciferase/IL-1β (**e**), luciferase/IL-6 (**f**), luciferase/IL-8 (**g**) and luciferase/ MIP-1α (**h**). Statistical differences were determined using a one-way ANOVA followed by Holm-Šídák test in **c**. *P < 0.05, **P < 0.01, ***P < 0.001, ****P < 0.0001. Data are presented as mean ± s.e.m. Schematic in **a** created in BioRender; Sharma, R. https://biorender.com/z9j4osz (2026). Source data

Figure 4e–h demonstrate that E20-SNPs have a unique combination of high transfection efficiency and low immunostimulatory effects in PBMCs and were the only LNP in the panel that did not stimulate human PBMCs to secrete cytokines. For example, LNPs based on MC3-DLin, 4A3-SC8, ALC-0315, SM-102 and cKK-E12 caused human PBMCs to make a variety of inflammatory cytokines. By contrast, the E20-SNP did not cause the production of any of the 14 cytokines investigated (Fig. 4d and Supplementary Figs. 90 and 91). In addition, the E20-SNP also transfected human PBMCs with luciferase mRNA efficiently and generated luciferase signals that were much higher than several of the LNPs investigated.

## SNPs do not activate the galectin-8, TLR4 and complement signalling pathways and also do not trigger the release of PAF

**Fig. 5:**
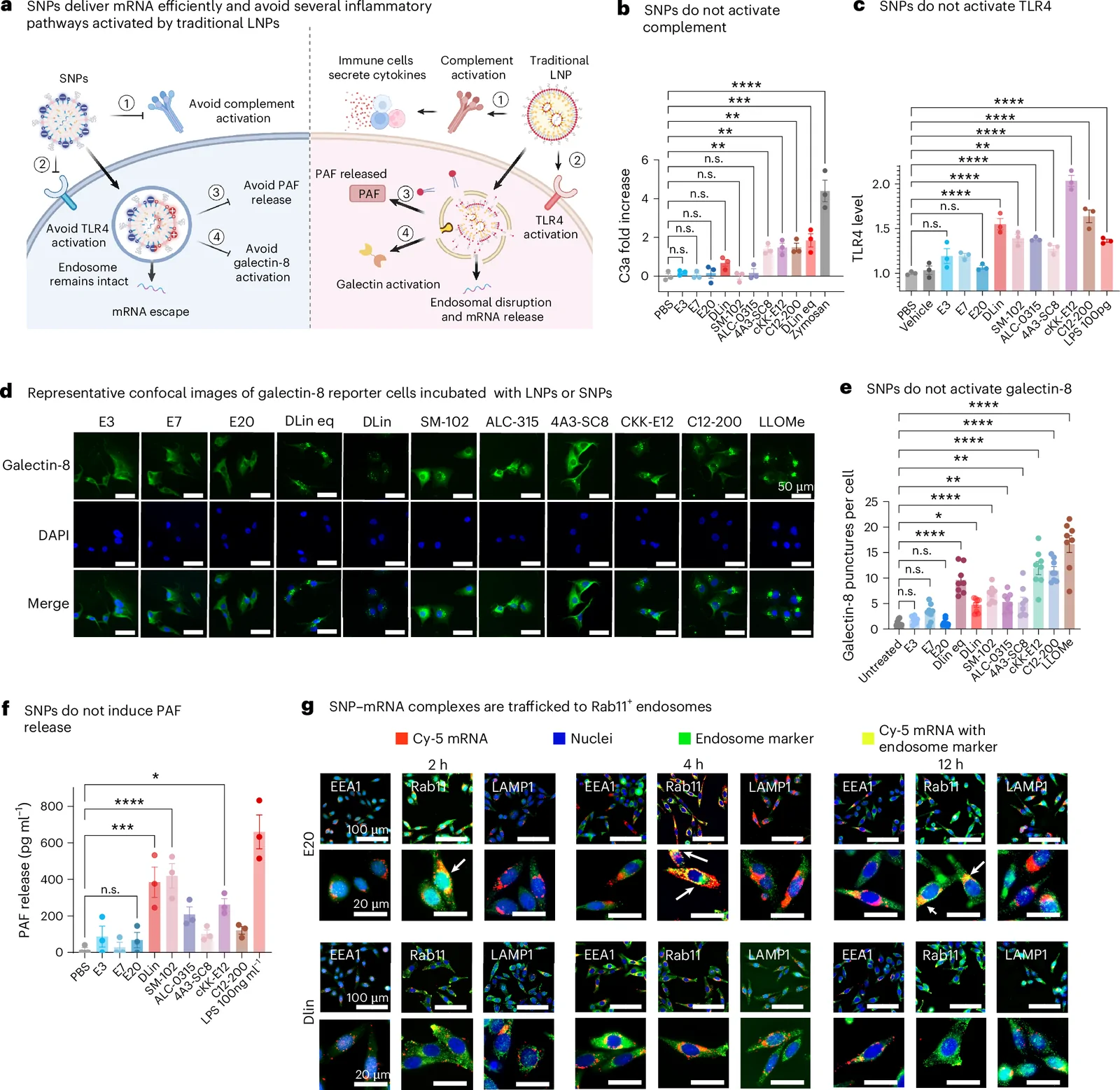
SNPs do not activate the common inflammatory pathways activated by traditional LNPs. **a**, SNPs avoid activating TLR4 or complement while circulating in the blood owing to their negative charge density. After endocytosis, SNPs avoid activating galectin-8 or stimulating PAF release because they trigger endosomal release without damaging cell membranes. By contrast, traditional LNPs activate multiple inflammatory signalling pathways. **b**, Human serum mixed with SNPs at a concentration of 2,000 ng ml^−1^ mRNA for 1 h at 37 °C showed no increase in complement C3a: negative control, untreated human serum; positive control, Zymosan (*n* = 3 biologically independent replicates). **c**, HEK293 cells containing a human TLR4 reporter gene were incubated with SNPs or traditional LNPs (2,000 ng ml^−1^ mRNA). SNPs do not activate TLR4, but traditional LNPs do; positive control, LPS; negative controls, PBS and vehicle (no lipids) (*n* = 3 biologically independent replicates). **d**, Representative fluorescent microscopy images of galectin-8-YFP reporter cells treated with LNPs or SNPs. **e**, E20-SNPs caused minimal galectin-8 recruitment to endosomes at an mRNA concentration of 1,000 ng ml^−1^, whereas traditional LNPs caused substantial levels of galectin-8 recruitment to endosomes (*n* = 8 views from three biologically independent replicates). **f**, SNPs do not trigger PAF release from human PBMCs, but traditional LNPs do (2,000 ng ml^−1^ mRNA, *n* = 3 biologically independent replicates). **g**, HeLa cells incubated with SNPs or LNPs carrying Cy5-labelled mRNA were stained for the endosomal markers EEA1 and Rab11 and the lysosomal marker LAMP1. SNPs preferentially accumulated in Rab11^+^ endosomes. Statistical differences were determined using one-way ANOVA followed by Dunnett’s test in **b**, **c**, **e** and **f**. *P < 0.05, **P < 0.01, ***P < 0.001, ****P < 0.0001 for **b**, **c**, **e** and **f**. Data are presented as mean ± s.e.m. Schematic in **a** created in BioRender; Sharma, R. https://biorender.com/q07dtqa (2026). Source data

LNPs cause inflammation by at least four different mechanisms. In particular, they activate the complement pathway, the TLR4 signalling pathway, the galectin-8 signalling pathway and trigger PAF release. LNP-mediated activation of the complement and TLR4 signalling pathways are caused by the positively charged head group of the ionizable lipid, but E20-SNPs are either negatively charged or neutral at pH 7.4. We therefore investigated whether E20-SNPs caused less activation of the TLR4 and complement signalling pathways than traditional LNPs. Figure 5b,c demonstrate that E20-SNPs do not activate either TLR4 or complement, whereas several traditional LNPs made with a variety of different ionizable lipids activated both signalling pathways.

**Extended Data Fig. 3:**
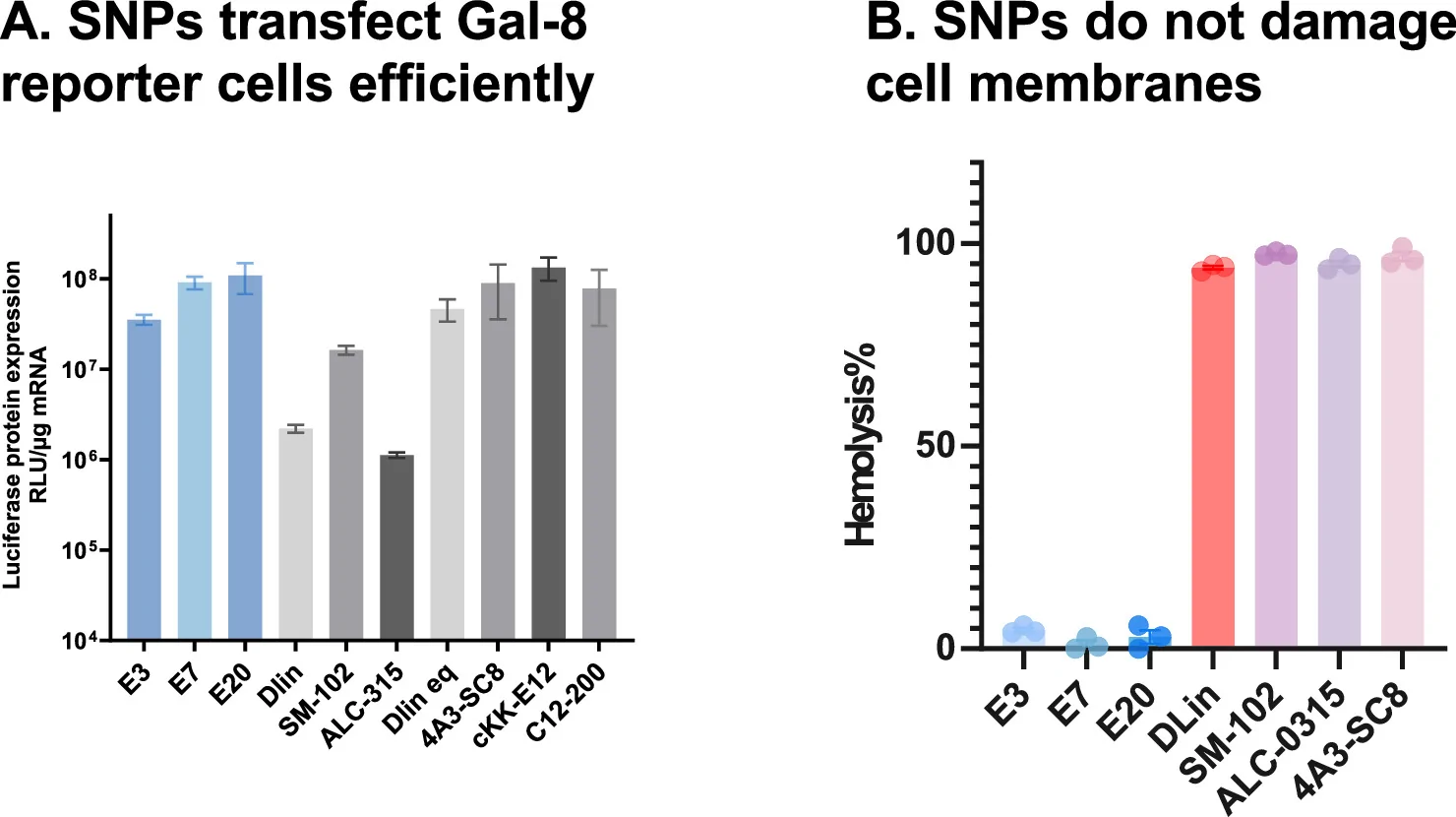
SNPs efficiently transfect Gal-8 reporter cells and do not disrupt red blood cell membranes. (**a**) SNPs efficiently transfect Gal-8 reporter cells (1000 ng/mL mRNA), indicating that the low galectin-8 recruitment of SNPs is not due to low transfection efficiency. (**b**) SNPs are not membrane disruptive, but traditional LNPs containing a range of ionizable lipids are membrane disruptive (n = 3 biologically independent replicates). PBS was used as the negative control (0% hemolysis) and lysis buffer was used as the positive control (100% hemolysis). Source data

We further investigated if SNPs caused less activation of the galectin-8 and PAF signalling pathways than traditional LNPs using a galectin-8 reporter cell line and human PBMCs as test beds. Figure 5d–f demonstrate that E20-SNPs do not activate either the galectin-8 or PAF signalling pathways, whereas all the traditional LNPs investigated activated both signalling pathways. In addition, E20-SNPs were also able to deliver mRNA efficiently to the galectin-8 reporter cell line and generated luciferase expression levels similar to traditional LNPs (Extended Data Fig. 3a). These experiments demonstrate that the E20-SNP can deliver mRNA into the cytoplasm without permeabilizing the endosome to galectin-8. We investigated the membrane-disruptive properties of E20-SNPs at pH 5.5 using a haemolysis assay to gain insight into E20-SNPs ability to avoid galectin-8 activation. Extended Data Fig. 3b demonstrates that E20-SNPs are not membrane disruptive at pH 5.5, whereas all the traditional LNPs investigated are membrane disruptive at pH 5.5.

We performed fluorescent intracellular trafficking experiments to identify the endosomal compartment from which the E20-SNP was being released from to gain further insight into their ability to trigger endosomal release without disrupting membranes. E20-SNPs were loaded with Cy5-labelled mRNA, incubated with cells for various timepoints and stained for the endosomal markers EEA1 and Rab11 and the lysosomal marker LAMP1. Figure 5g demonstrates that E20-SNPs accumulate in Rab11+ endosomes, which is a release competent endosomal compartment^35^. These experiments suggest that being trafficked to Rab11+ endosomes can allow LNPs to enter the cytoplasm, without activating the galectin-8 or PAF signalling pathways.

## SNPs containing IL-22 mRNA are better at treating mice suffering from acute lung injury than traditional LNPs

Finally, we used LPS-induced lung injury as a test bed to investigate if E20-SNPs could treat inflammatory diseases better than traditional LNPs, owing to their ability to avoid inflammation exacerbation. We selected IL-22 mRNA as the therapeutic cargo because the IL-22 protein is a well-established therapeutic for treating LPS-induced lung injury^36,37^. Mice were predosed with LPS intratracheally, and after 3 h, E20-SNPs, MC3-DLin, SM-102, ALC-0315 or 4A3-SC8 LNPs were administered via intratracheal instillation (IL-22 mRNA dose 2.5 mg kg^−1^). At 24 h after the LNP treatment, mice were killed and analysed for inflammation and for IL-22 protein in the serum.

**Fig. 6:**
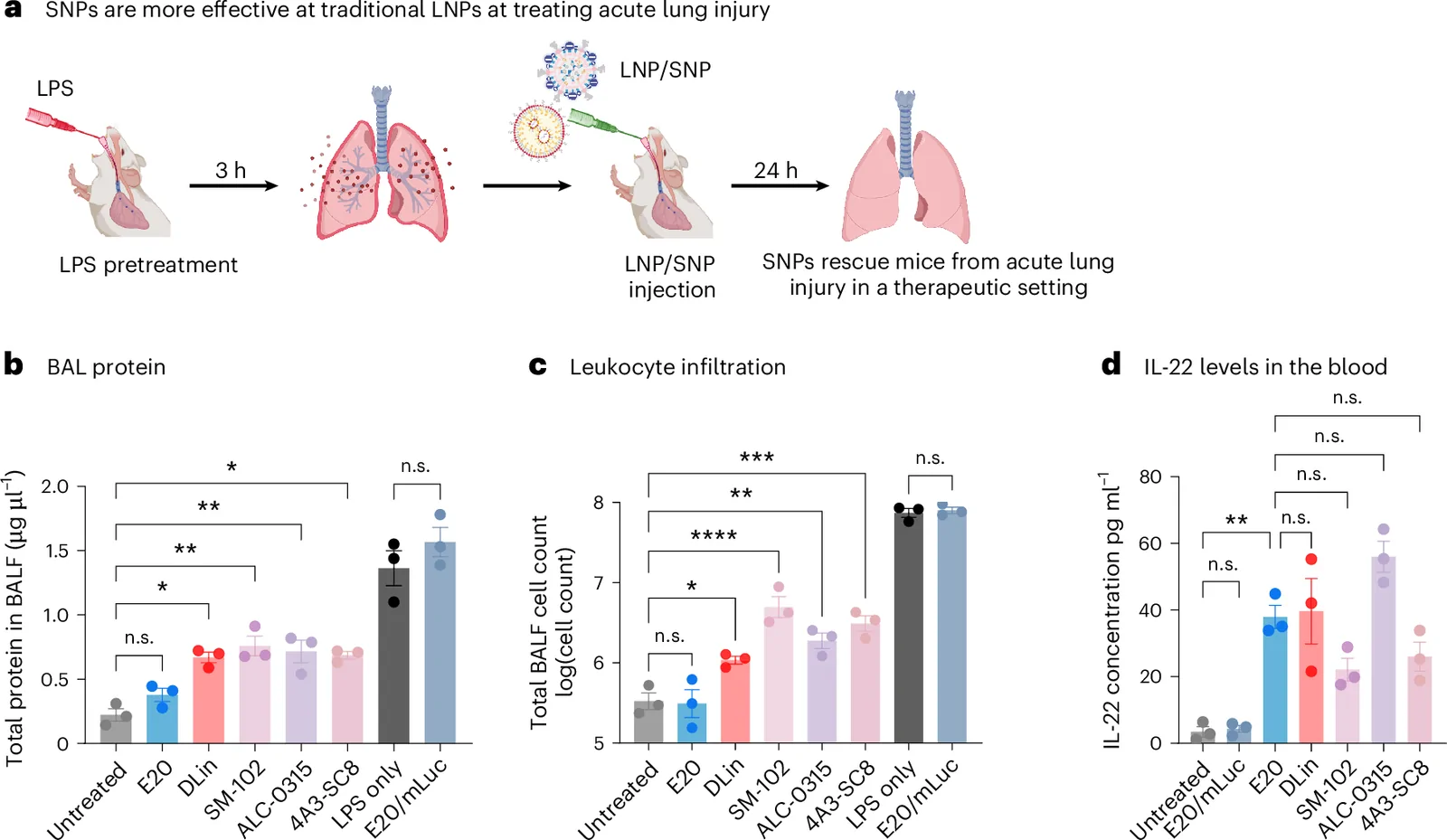
SNPs can rescue mice from acute lung injury and are more effective than traditional LNPs at treating acute lung injury. **a**, Mice were treated with LPS (3 mg kg^−1^, intratracheally) and, 3 h later, were treated with IL-22 mRNA formulated in E20-SNPs or LNPs (2.5 mg kg^−1 ^mRNA dose, intratracheal administration). A scrambled mRNA control group (E20-SNPs/mLuc, 2.5 mg kg^−1^, intratracheally) was included. Inflammation was assessed 24 h after the mRNA treatment by measuring the total number of cells infiltrating into the lungs and the amount of protein leaking into the lungs. **b**, The E20-SNP-treated mice had their lung permeability phenotype completely rescued and had pulmonary protein levels similar to healthy mice. By contrast, mice treated with traditional LNPs had increased levels of protein in their lungs (*n* = 3 biologically independent replicates). **c**, The E20-SNP-treated mice had cell infiltration levels similar to the untreated controls, whereas mice treated with traditional LNPs had increased infiltration (*n* = 3 biologically independent replicates). **d**, E20-SNPs deliver IL-22 mRNA with an efficiency comparable to traditional LNPs (*n* = 3 biologically independent replicates). Statistical differences were determined using one-way ANOVA followed by Tukey’s test (**b**–**d**). *P < 0.05, **P < 0.01, ***P < 0.001, ****P < 0.0001 for **b**–**d**. Data are presented as mean ± s.e.m. Schematic in **a** created in BioRender; Sharma, R. https://biorender.com/l8enkxr (2026). Source data

Figure 6b,c demonstrates that E20-SNPs are more effective at treating acute lung injury in mice with pre-existing inflammation than traditional LNPs. For example, mice treated with E20-SNPs had their lung permeabilization phenotype completely rescued. By contrast, mice treated with LNPs made from all of the traditional LNPs still showed substantial levels of lung permeabilization (Fig. 6b). In addition, mice treated with E20-SNPs had markedly lower numbers of cells infiltrating into their lungs in comparison with mice treated with traditional LNPs, and E20-SNPs also rescued this inflammatory phenotype (Fig. 6c). The IL-22 mRNA delivery efficiency of E20-SNPs was similar to traditional LNPs (Fig. 6d), and E20-SNP’s superior ability to treat acute lung injury probably results from its low immunostimulatory properties in comparison with traditional LNPs.

## Conclusion

In this Article, we demonstrate that ionizable lipids that contain a single tertiary amine and carboxylic acid can generate LNPs (termed SNPs) that deliver mRNA and pDNA efficiently with low toxicity. The low toxicity of the SNPs arises from their ability to avoid activating the complement pathway, the TLR4 signalling pathway, the galectin-8 signalling pathway and the release of PAF. However, the intracellular metabolism and accumulation of the S-lipids was not investigated, and the SNPs could cause toxicity after chronic administration by accumulating in cells. Future generations of S-lipids may therefore need to incorporate additional esters in their hydrophobic tails to accelerate their intracellular clearance^38,39^. The S-lipids were able to lower the toxicity of both mRNA and pDNA in a variety of cell and animal models, and collectively, the experiments in this report demonstrate that ionizable lipids with a single amine and carboxylic acid have numerous potential applications in drug delivery.

## Method

### Ethics statement

The research complied with all relevant ethical regulations. Animal housing, maintenance and experimental procedures were approved by the Institutional Animal Care and Use Committees of the University of California, Davis (protocol number 23430) and the University of California, Berkeley (AUP-2015-02-7140-3). All animal facilities used during the study period were accredited by the Association for Assessment and Accreditation of Laboratory Animal Care International.

### LNP preparation

LNPs were prepared by mixing lipids in ethanol with mRNA dissolved in an acidic aqueous buffer at a volume ratio of 1 to 3. LNPs were dialysed against PBS, unless otherwise indicated. Detailed protocols for the formation of LNPs are described in the Supplementary Information in section 4.1.

### R18 assay for p*K*_a_ measurements

The cationic fluorescent probe octadecyl rhodamine B chloride (R18) was used to evaluate the p*K*_a_ at which SNPs become neutral. SNP–mRNA complexes were prepared without dialysis and diluted in PBS at pH 7.0 to achieve a final lipid concentration of 6 mM. They were then incubated for 15 min at room temperature and vortexed for 2 min. The solution was diluted to 600 μM lipid in PBS pH 7.0, and the R18 dye was added to generate a final concentration of 30 μM. This mixture was further diluted to 12.0 μM lipids in 1.5 ml of buffered solutions with pH values ranging from 2.0 to 12.0. After 30 min of incubation at room temperature, rhodamine fluorescence was measured at 20 °C with excitation and emission wavelengths of 568 and 594 nm, respectively. A sigmoidal best-fit analysis was used to determine the lipid p*K*_a_, defined as the pH at which 50% of the maximum fluorescence intensity was observed.

### Delivery of firefly luciferase mRNA to HEK 293T cells with LNPs

HEK 293T cells were seeded in 96-well plates at a density of 10,000 cells per well in 100 µl of Dulbecco’s modified Eagle medium (DMEM) with 10% foetal bovine serum (FBS) and incubated at 37 °C overnight. LNPs were formulated with firefly luciferase mRNA (GenScript) and were diluted with PBS to a final mRNA concentration of 5 µg ml^−1^. Next, 10 μl of the diluted LNP mixture and 90 μl of fresh Opti-MEM were added to each well, replacing the old medium. The final dose of mRNA was 50 ng per well (*n* = 3 biologically independent replicates). After 24 h, the cells were washed with PBS (pH 7.4) and analysed using a luciferase assay kit (Goldbio) according to the manufacturer’s instructions. Cells were washed with 200 μl PBS (pH 7.4) and lysed with 40 μl lysis buffer for 15 min. A 10-μl aliquot of the lysate was transferred to a black flat-bottom plate and mixed with 100 μl luciferase working solution (D-luciferin, 10 mg ml^−1^, diluted 1:50 in luciferase assay buffer). Luminescence was measured immediately using a Tecan Infinite M200 plate reader (Tecan). Luciferase expression levels were normalized relative to total cell number, which was quantified by analysing 10 µl of cell lysate using a bicinchoninic acid (BCA) assay kit (Thermo Fisher). The relative cell number was calculated by comparing the BCA values in the treatment group with those in the control group.

### Delivery of EGFP mRNA to HEK 293 T cells with LNPs

HEK 293T cells were seeded in 96-well plates at a density of 10,000 cells in a 100 μl volume per well and incubated at 37 °C overnight in DMEM with 10% FBS. LNP–mRNA complexes containing 10 μg of GFP mRNA (GenScript) were collected from the dialysis kit and diluted with PBS to a final volume of 200 μl. Aliquots of 2 μl of this dilution were mixed with Opti-MEM to a final volume of 100 μl. The resulting formulations were then added to each well without removing the existing culture medium (final mRNA concentration of 500 ng ml^−1^). After 17 h, the cells were washed with PBS at pH 7.4 and detached with Accutase solution for 10 min at 37 °C. The transfection efficiency of the LNPs was analysed using an Attune flow cytometer (Thermo Fisher). Data analysis was performed using FlowJo v10.7.1 software (FlowJo).

### Endosomal membrane disruption study by galectin-8 reporter cells

Galectin-8-YFP-MDA-MB231 cells were graciously provided by Dr. Craig L. Duvall. Cells were cultured in a 96-well plate at a density of 20,000 cells ml^−1^ in 100 μl DMEM with 10% FBS per well and incubated at 37 °C overnight. LNP–mRNA complexes containing 10 μg of firefly luciferase mRNA (GenScript) were formulated as described in Supplementary Information section 4.1.4 and diluted with PBS to a total volume of 400 μl. Aliquots of 0.8, 4 or 8 μl of this dilution were mixed with Opti-MEM to a final volume of 100 μl. The resulting formulations were then added to each well without removing the existing culture medium, generating solutions with mRNA concentrations of 0.1 µg ml^−1^, 0.5 µg ml^−1^ and 1 µg ml^−1^. Cells were incubated with LNPs or SNPs for 4 h. After incubation, the supernatants were aspirated and the cells were washed three times with PBS at pH 7.4. Cells were fixed in 10% formalin for 24 h, and the nuclei were stained with DAPI. Fluorescence microscopy was then performed, and the number of puncta per cell was quantified. Details are described in the Supplementary Information in section 7.5.

### Activation of complement with LNPs and SNPs

Human serum was purchased from STEMCELL Technologies (200-0159). Samples (90 μl) were incubated with 10 μl of either control or SNPs/LNPs (final mRNA concentration: 2,000 ng ml^−1^). Zymosan (5 mg ml^−1^) was used as a positive control. PBS was used as a negative control. Samples were incubated for 1 h at 37 °C and subsequently centrifuged at 500*g* for 5 min to pellet the zymosan. Supernatants were transferred to fresh polypropylene tubes, immediately sealed and stored at −80 °C until analysis. On the day of analysis, serum samples were rapidly thawed at 37 °C and assayed for C3a using a Human Complement C3a enzyme-linked immunosorbent assay (ELISA) (BMS2089, Invitrogen), according to the manufacturer’s instructions.

### Cellular trafficking of SNPs and LNPs

HeLa cells were seeded in confocal dishes with complete medium and incubated overnight. Cy5-labelled SNPs or LNPs (2,000 ng ml^−1^ mRNA) were added to the dishes with complete medium and incubated for 2 h, 4 h and 12 h. The cells were then washed with PBS and fixed with 4% paraformaldehyde. The fixed cells were blocked with 5% BSA for 30 min. Primary antibody dilutions of anti-EEA1 antibody (EPR4245, Abcam), anti-LAMP1 antibody (EPR21026, Abcam) and anti-Rab11 antibody (EPR7587(B), Abcam) were added to the dishes and incubated for 2 h. The primary antibodies were removed, and the cells were washed with PBS three times; secondary antibodies were added and incubated for 1 h. Secondary antibodies were removed and washed three times with PBS, after which, the cells were stained with DAPI and analysed by confocal microscopy (ImageXpress).

### Immunostimulation of human PBMCs with LNPs or SNPs

PBMCs were isolated from fresh leukopaks from four healthy donors (STEMCELL Technologies). The leukopak was diluted 1:1 with MACS/EasySep buffer (2% FBS, 2 mM EDTA in PBS) and centrifuged at 100*g* for 15 min. The supernatant was discarded, and the cell pellet was resuspended in 50 ml of MACS/EasySep buffer. The suspension was then centrifuged at 400*g* for 5 min, and the supernatant was removed again. This washing step was repeated twice. PBMCs were counted using a 3% acetic acid solution containing methylene blue. Cells were cultured in 48-well plates at a density of 5–10 × 10^5^ per well in 200 μl RPMI medium supplemented with 10% FBS and incubated overnight at 37 °C. LNP–mRNA complexes containing 10 μg of firefly luciferase mRNA (GenScript) were formulated as described in Supplementary Information section 4.1.4 and diluted with PBS to a total volume of 100 μl. Aliquots of 2, 4 or 8 μl of this dilution were further diluted with Opti-MEM to a final volume of 200 µl. These dilutions were added to each well without removing the existing culture medium, resulting in final mRNA concentrations of 0.5 μg ml^−1^, 1 μg ml^−1^ or 2 μg ml^−1^, respectively. Cells were incubated at 37 °C in a humidified atmosphere with 5% CO_2_ for 24 h or 48 h. After incubation, the cells were pelleted by centrifugation at 1,000*g* for 10 min at 4 °C. The supernatants were collected and stored at −80 °C until cytokine analysis. Cytokine levels were measured using ELISA kits (R&D Systems) according to the manufacturer’s instructions. The PBMC pellets were collected, and their luciferase expression was tested by the method mentioned in the luciferase mRNA transfection study.

### In vivo firefly luciferase mRNA delivery with SNP–mRNA complexes

Wild-type BALB/c mice weighing between 18 and 20 g and aged 10–16 weeks old were used for firefly luciferase mRNA delivery experiments. An aliquot of dialysed SNP–mRNA complexes containing 25 µg mRNA was diluted to 500 µl using PBS (pH 7.4). A 100-µl volume of LNPs (0.25 mg kg^−1^ mRNA) was administered intravenously via retro-orbital injection; 4–6 h later, a D-luciferin solution in PBS pH 7.4 (50 mg kg^−1^) was injected into the intraperitoneal cavity of the mice. After 10 min, the luciferase luminescence intensity of the whole body and organs (lungs, liver, kidneys, heart and spleen) was imaged using an In Vivo Imaging System (IVIS) spectrum imager (PerkinElmer).

### In vivo firefly luciferase pDNA delivery with SNP–pDNA complexes

SNP–pDNA complexes were prepared with luciferase nanoplasmid (pALD-Nanoplasmid-CMV-luc, Aldevron). An aliquot of dialysed DLin LNP–pDNA or E20-SNP–pDNA complexes containing 100 µg of pDNA was diluted with PBS (pH 7.4) to a final volume of 600 µl. A 120-µl dose (20 µg pDNA, 1.0 mg kg^−1^) was administered intravenously via retro-orbital injection to three mice in each group. A group of three mice injected with PBS served as the blank control. At 1, 2, 3, 4, 5, 6 and 7 days post injection, a D-luciferin solution in PBS pH 7.4 (50 mg kg^−1^) was administered to mice by intraperitoneal injection. At 10 min after the D-luciferin injection, the luminescence intensity of the whole body was measured using an IVIS spectrum imager (PerkinElmer). Details are described in the Supplementary Information in section 6.2.

### In vivo human EPO mRNA delivery with SNP–mRNA complexes

Wild-type BALB/c mice weighing between 18 and 20 g and aged 10–16 weeks were used for the hEPO mRNA delivery experiments. DLin LNP–mRNA and SNP–mRNA complexes were made with hEPO mRNA (RP-A00034-1, GenScript). An aliquot of dialysed LNP–mRNA or SNP–mRNA complexes containing 10 µg mRNA was diluted to 500 µl with PBS (pH 7.4). A 100 µl volume of LNPs (0.1 mg kg^−1^ mRNA) was administered intravenously via retro-orbital injection. The blood of the mice was collected 24 h after injection and analysed using an hEPO ELISA kit (DY286-05, R&D Systems), following the manufacturer’s protocol.

### Immunostimulatory effects of SNP–mRNA complexes in LPS-treated mice

Wild-type BALB/c mice and C57BL/6 mice weighing between 18 and 20 g and aged 10–16 weeks were used to investigate LNP-mediated inflammation exacerbation. LPS from *Escherichia* *coli* (Sigma) was injected intravenously (retro-orbital intravenous) into mice at 1 mg kg^−1^. LNP–mRNA complexes were made with luciferase mRNA and dialysed in PBS at 4 °C for 2 h. At 4 h after the LPS challenge, LNP–mRNA complexes were administered via the retro-orbital intravenous injection route at a dose of 1 mg kg^−1^ mRNA. The blood of the mice was collected 4 h or 24 h for BALB/c, and 4 h for C57BL/6, after LNP–mRNA injection and analysed using Luminex (Invitrogen), following the manufacturer’s protocols.

### Immunostimulatory effects of SNP–pDNA complexes in mice

Wild-type BALB/c mice weighing between 18 and 20 g and aged 10–16 weeks were used for firefly luciferase pDNA delivery experiments. LNP and SNP formulations were prepared with nanoplasmid (pALD-Nanoplasmid-CMV-luc, Aldevron). An aliquot of dialysed DLin LNP–pDNA or E20-SNP–pDNA complexes containing 100 µg of pDNA was diluted with PBS (pH 7.4) to a final volume of 600 µl. A 120-µl dose (20 µg pDNA, 1.0 mg kg^−1^) of pDNA–LNP complexes was administered intravenously via retro-orbital injection to three mice for each group. A group of three mice injected with PBS served as the blank control. Mice were monitored and a survival curve was recorded. Mice were killed after 7 days of observation, and their blood and organs were collected. All three mice in the DLin treated group died, and we could not collect their blood; their organs were collected. The cytokine levels in the serum of SNP treated mice were analysed by Luminex (Invitrogen) according to the manufacturer’s protocol. Liver damage was assessed by measuring the alanine aminotransferase activity in the serum, using a fluorometric kit (AB105134-1001). The heart, lung, liver, spleen and kidney histology were analysed to further evaluate organ damage.

### Rescue of mice from acute lung injury with SNPs

Wild-type BALB/c mice (male and female, Jackson Laboratory, 000651), 10–16 weeks old and weighing between 18 and 20 g, were used in this experiment. The efficacy of E20-SNP–IL-22 mRNA was compared against LNPs formulated with the ionizable lipids 4A3-SC8 (benchmark traditional lipid), DLin-MC3-DMA (FDA-approved ionizable lipid), SM-102 (FDA-approved ionizable lipid), ALC-0315 (FDA-approved ionizable lipid) and IL-22 mRNA. Mice were randomly assigned to four groups: LPS + IL-22 mRNA treatment group, untreated control group, LPS-only group and scrambled mRNA control group. In the treatment group, mice were challenged with 3 mg kg^−1^ LPS intratracheally. They were then maintained for 3 h to stimulate acute inflammatory injury. Subsequently, mice were administered 2.5 mg kg^−1^ doses of LNP–IL-22 mRNA complexes intratracheally (E20-SNPs and MC3-DLin, SM-102, ALC-0315, 4A3-SC8 LNPs prepared as described in the Supplementary Information section 4.1.4) and were killed 24 h later. In the untreated control group, mice received intratracheal PBS and were killed 24 h later. In the LPS group, mice were challenged with 3 mg kg^−1^ LPS via intratracheal administration. After 3 h, they were administered PBS intratracheally and were killed 24 h later. In the scrambled mRNA control group, mice were challenged with 3 mg kg^−1^ LPS via intratracheal administration. After 3 h, they were administered 2.5 mg kg^−1^ doses of E20-SNP–luciferase mRNA complexes intratracheally and were killed 24 h later.

Blood samples and bronchoalveolar lavage fluid from the lower right lung were collected, and the total protein content was analysed using the BCA assay. An equal volume (10 μl) of resuspended bronchoalveolar lavage fluid cell suspension was mixed with 0.4% trypan blue solution (10 μl) and incubated at room temperature for 2 min. Viable cells were quantified using an automated cell counter. Total cell numbers were calculated and reported as cells per millilitre. Blood samples were analysed for cytokines by Luminex (Invitrogen) according to the manufacturer’s protocol, and IL-22 protein level was measured by ELISA.

### Reporting summary

Further information on research design is available in the Nature Portfolio Reporting Summary linked to this article.

## Online content

Any methods, additional references, Nature Portfolio reporting summaries, source data, extended data, supplementary information, acknowledgements, peer review information; details of author contributions and competing interests; and statements of data and code availability are available at https://doi.org/10.1038/s41565-026-02262-6.

## Supplementary information

Supplementary Information Supplementary Figs. 1–106, Discussion and Tables 1 and 2.

Reporting summary

## Source data

Source Data Fig. 2 Statistical source data.

Source Data Fig. 3 Statistical source data.

Source Data Fig. 4 Statistical source data.

Source Data Fig. 5 Statistical source data.

Source Data Fig. 6 Statistical source data.

Source Data Extended Data Fig. 2 Statistical source data.

Source Data Extended Data Fig. 3 Statistical source data.

## Figures and Tables

**Extended Data Fig. 1:**
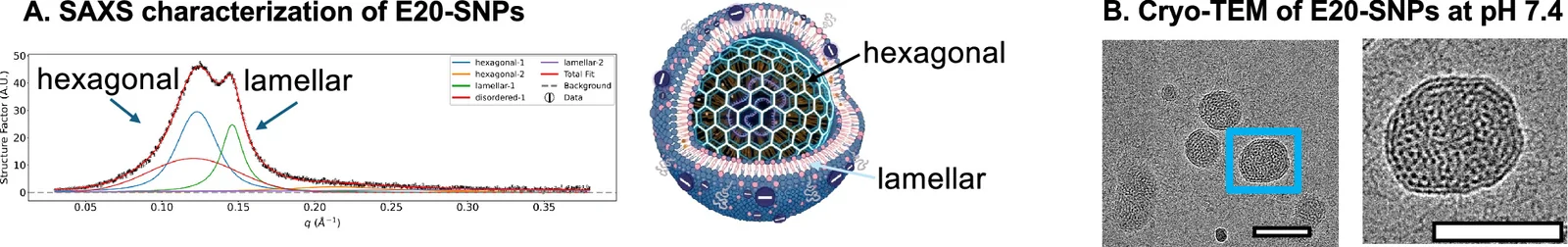
SAXS and cryo-TEM analysis demonstrate that SNPs contain a mixture of inverted hexagonal and bilayer phases in their core. (**a**) SAXS analysis demonstrates that E20-SNPs contain a mixture of inverted hexagonal and bilayer phases. (**b**) Cryo-TEM demonstrates that E20-SNPs contain a mixture of inverted hexagonal and bilayer phases in their core and had a shell that was predominantly composed of bilayer phases. Images are representative of 85 fields of view collected from 3 independently prepared samples. Scale bars: 100 nm (left) and 90 nm (right). Data are presented as mean ± SEM.

## Data Availability

The data supporting the findings of this study are available within the Article and its Supplementary Information. Additional raw data files, including data in alternative formats, are available from the corresponding authors upon request. Source data are provided with this paper.
