# Supplementary material for: Charge-switching ionizable lipids lower the toxicity of lipid nanoparticles
Source: Nat Nanotechnol. 2026 Sep 8;21(10):1479–88. doi: 10.1038/s41565-026-02262-6 (PMC13661284; doi:10.1038/s41565-026-02262-6)
Supplement: Supplementary file 1 [file 41565_2026_2262_MOESM1_ESM.pdf]

nature nanotechnology

Supplementary information

https://doi.org/10.1038/s41565-026-02262-6

Charge-switching ionizable lipids lower the toxicity of lipid nanoparticles

In the format provided by the authors and unedited

Table of Contents

1. General methods and materials 7
   1.1 Reagents and materials 7
   1.2 Instruments 7
   1.3 Statistics and reproducibility 7
2. Synthetic procedures for the S-lipids 8
   2.1 General synthetic procedures for the epoxide-based S-lipids (E1-E120) 9
   2.2 General synthetic procedures for the acrylate-based S-lipids 12
   2.3 General synthetic procedure for branched alcohols 13
   2.4 Synthetic procedure for the linear S-lipids 14
   2.5 Chemical structures of the top-performing S-lipids 15
   2.6 Purity analysis of the top-performing S-lipids 16
3. NMR spectra of compounds 23
4. *In vitro* mRNA delivery experiments with LNPs 125
   4.1 General procedure for preparing LNPs 125
   4.2 Delivery of firefly luciferase mRNA to HEK 293T cells with LNPs 126
   4.3 Delivery of EGFP mRNA to HEK 293T cells with LNPs 130
   4.4 Delivery of EGFP mRNA to primary fibroblast cells with LNPs 131
5. SNP Characterization 132
   5.1 Evaluation of LNP mRNA encapsulation efficiency 132
   5.2 Dynamic light scattering (DLS) analysis of SNPs and LNPs 134
   5.3 Zeta potential of SNPs and LNPs 135
   5.4 R18 assay for pKa measurements 137
   5.5 Cryo-TEM of SNPs 138
   5.6 Small angle X-ray scattering (SAXS) of E20 SNPs 139
   5.7 Viscosity measurement of SNPs 140

5.8 Shelf-life stability of SNPs at 4 °C 142
6. *In vivo* mRNA and pDNA delivery experiments with LNPs 143
6.1 *In vivo* firefly luciferase mRNA delivery with SNP/mRNA complexes 143
6.2 *In vivo* firefly luciferase pDNA delivery with SNP/pDNA complexes 146
6.3 *In vivo* human EPO mRNA delivery with SNP/mRNA complexes 146
6.4 Rescue of mice from acute lung injury with SNPs 147
7. Immunostimulatory effects study of SNPs 148
7.1 Immunostimulation of human PBMCs with LNPs or SNPs 148
7.2 Internalization of LNPs and SNPs by human PBMCs 150
7.3 Immunostimulatory effects of SNP/mRNA complexes in LPS-treated mice 151
7.4 Immunostimulatory effects of SNP/pDNA complexes in mice 154
7.5 Endosomal membrane disruption study by Gal-8 reporter cells 155
7.6 Bafilomycin inhibition of SNP and LNP transfection 159
7.7 Hemolysis of red blood cell membranes with LNPs and SNPs 160
7.8 Cellular trafficking of SNPs and LNPs 161
7.9 Activation of TLR4 in TLR4 Reporter HEK293 cells with LNPs or SNPs 162
7.10 Activation of complement with LNPs or SNPs 163
7.11 SNP/LNP release of platelet activating factor (PAF) 163
7.12 LDH assessment 164
7.13 Endotoxin assessment 165

List of Figures

Figure S1. General synthetic scheme for the synthesis of the S-lipid library. 9
Figure S2. Synthesis of epoxide-based S-lipids (E1-E120). 10
Figure S3. Synthesis of acrylate-based S-lipids (A1-A24). 12
Figure S4. General procedure for the synthesis of branched alcohols 13
Figure S5. Synthesis of linear S-lipids (12a-12f) 14

Figure S6. Chemical structures of the top-performing S-lipids. 15
Figure S7. LCMS of top performing S-lipids part 1. 17
Figure S8. LCMS of top performing S-lipids part 2. 18
Figure S9. LCMS of top performing S-lipids part 3. 19
Figure S10. High-resolution mass spectrometry of S-lipid E3. 20
Figure S11. High-resolution mass spectrometry of S-lipid E7. 20
Figure S12. High-resolution mass spectrometry of S-lipid E12. 21
Figure S13. High-resolution mass spectrometry S-lipid E15. 21
Figure S14. High-resolution mass spectrometry of S-lipid E20. 22
Figure S15. Chemical structure of compound 1a. 23
Figure S16. Chemical Structure of compound 1b. 25
Figure S17. Chemical Structure of compound 1c. 27
Figure S18. Chemical Structure of compound 1d. 29
Figure S19. Chemical Structure of compound 1e. 31
Figure S20. Chemical Structure of compound 1f. 33
Figure S21. Chemical Structure of compound 1g. 35
Figure S22. Chemical Structure of Compound 1h. 37
Figure S23. Chemical structure of compound 4a. 39
Figure S24. Chemical structure of compound 4b. 41
Figure S25. Chemical structure of compound 4c. 43
Figure S26. Chemical structure of compound 4d. 45
Figure S27. Chemical structure of compound 4e. 47
Figure S28. Chemical structure of compound 4f. 49
Figure S29. Chemical structure of compound 4g. 51
Figure S30. Chemical structure of compound 4h. 53
Figure S31. Chemical structure of compound 4i. 55
Figure S32. Chemical structure of compound 4j. 57
Figure S33. Chemical structure of compound 4k. 59
Figure S34. Chemical structure of compound 4l. 61
Figure S35. Chemical structure of compound 4m. 63
Figure S36. Chemical structure of compound 4n. 65
Figure S37. Chemical structure of compound 4o. 67
Figure S38. Chemical structure of compound 4p. 69

Figure S39. Chemical structure of compound 4q. 71
Figure S40. Chemical structure of compound 4r. 73
Figure S41. Chemical structure of compound 4s. 75
Figure S42. Chemical structure of compound 4t. 77
Figure S43. Chemical structure of compound 4u. 79
Figure S44. Chemical structure of compound 4v. 81
Figure S45. Chemical structure of compound 4w. 83
Figure S46. Chemical structure of compound 4x. 85
Figure S47. Chemical structure of compound E3. 87
Figure S48. Chemical structure of compound E7. 89
Figure S49. Chemical structure of compound E12. 91
Figure S50. Chemical structure of compound E15. 93
Figure S51. Chemical structure of compound E20. 95
Figure S52. Chemical structure of compound 8a. 97
Figure S53. Chemical structure of compound 8b. 99
Figure S54. Chemical structure of compound 8c. 101
Figure S55. Chemical structure of compound 8d. 103
Figure S56. Chemical structure of compound 8e. 105
Figure S57. Chemical structure of compound 8f. 107
Figure S58. Chemical structure of compound 8g. 109
Figure S59. Chemical structure of compound 8h. 111
Figure S60. Chemical structure of compound 8i. 113
Figure S61. Chemical structure of compound 8j. 115
Figure S62. Chemical structure of compound 8k. 117
Figure S63. Chemical structure of compound 8l. 119
Figure S64. Chemical structure of compound A1. 121
Figure S65. Chemical structure of compound A13. 123
Figure S66. Effect of head–linker length S-lipid transfection efficiency (Figure 2D heat map). 127
Figure S67. Effect of spacer length on S-lipid transfection efficiency (Figure 2D heat map). 128
Figure S68. Effect of branch length on S-lipid transfection efficiency (Figure 2D heat map). 128

Figure S69. Epoxide based S-lipids with branched hydrophobic tails deliver mRNA more efficiently than S-lipids with linear tails. 129
Figure S70. Epoxide based S-lipids with branched hydrophobic tails deliver mRNA more efficiently than S-lipids with linear tails. 130
Figure S71. Flow cytometry gating strategy for analysis of EGFP mRNA transfection in HEK cells. 131
Figure S72. Flow cytometry gating strategy for analysis of EGFP mRNA transfection in primary fibroblast cells. 132
Figure S73. The GFP mRNA encapsulation efficiency of the SNPs depends on the chemical structure of the S-lipid 133
Figure S74. The luciferase mRNA encapsulation efficiency of the SNPs depends on the chemical structure of the S-lipid. 133
Figure S75. The Cas9 mRNA encapsulation efficiency of the SNPs depends on the chemical structure of the S-lipid. 134
Figure S76. Hydrodynamic size of the SNPs.. 135
Figure S77. Hydrodynamic polydispersity index (PDI) of the SNPs. 135
Figure S78. Zeta potential measurement of the SNPs. 136
Figure S79. pH dependent zeta potential of E20 SNPs. 137
Figure S80. pH dependent zeta potential of DLin LNPs. 137
Figure S81. Representative cryo-TEM images of E20 SNPs. 139
Figure S82. SAXS analysis of E20-SNPs.. 140
Figure S83. The S-lipid spacer length influences the microviscosity of the SNPs. 141
Figure S84. The S-lipid spacer length influences the microviscosity of the SNPs. 142
Figure S85. E20 SNPs and DLin LNPs have similar shelf-lives. 142
Figure S86. In vivo luminescence intensity of mice treated with SNPs. 144
Figure S87. In vivo luciferase luminescence intensity of organs (lungs, liver, kidneys, heart, and spleen) treated with SNPs. 145
Figure S88. SNPs transfect the liver efficiently. 145
Figure S89. SNPs deliver pDNA efficiently. 146
Figure S90. Heat map of cytokine levels in PBMCs (4 healthy donors) treated with E20-SNPs or traditional LNPs (500 ng/mL and 1000 ng/mL mRNA) for 24 hours. 149
Figure S91. Heat map of cytokine levels in PBMCs (4 healthy donors) treated with E20-SNPs or traditional LNPs (500, 1000, and 2000 ng/mL mRNA) for 48 hours. 149

Figure S92. Analysis of neutrophil content in human PBMC preparations 150
Figure S93. Internalization of E20 SNPs and LNPs by human PBMCs. 151
Figure S94. Flow cytometry gating strategy for assessing PBMC uptake of LNPs and SNPs. 151
Figure S95. Heat map of cytokine levels in LPS-treated mice dosed with E20-SNPs or traditional LNPs (1mg/kg mRNA) for 24 hours. 152
Figure S96. In vivo luminescence image in LPS-pre-treated mice dosed with LNPs. . 153
Figure S97. E20 SNPs do not exacerbate inflammation in C57BL/6 mice. 153
Figure S98. Histology of heart, lung, spleen, and kidney in mice dosed with E20 SNPs, DLin LNPs, or PBS. 154
Figure S99. SNPs deliver luciferase mRNA (100 ng/mL) to Gal-8 reporter cells efficiently. 156
Figure S100. SNPs deliver luciferase mRNA (500 ng/mL) to Gal-8 reporter cells efficiently. 157
Figure S101. SNPs deliver luciferase mRNA (1000 ng/mL) to Gal-8 reporter cells efficiently. 157
Figure S102. SNPs do not recruit galectin-8 to endosomes (100 ng/mL mRNA). 158
Figure S103. SNPs do not recruit galectin-8 to endosomes (500 ng/mL mRNA). 159
Figure S104. Bafilomycin inhibition of SNP/LNP transfection. 160
Figure S105. Hemolysis of red blood cell membranes with LNPs and SNPs at pH 7.4 161
Figure S106. LNPs and SNPs do not trigger the release of LDH from cells................ 165

1. General methods and materials

1.1 Reagents and materials

Commercial reagents used in standard organic synthesis were obtained from Sigma-Aldrich (St. Louis, MO, USA), Fisher Scientific (Hampton, NH, USA), and VWR (Radnor, PA, USA). Phosphate-buffered saline (PBS) and Dulbecco's Phosphate Buffered Saline (DPBS) were obtained from Fisher Healthcare. Dulbecco’s Modified Eagle Medium (DMEM) and citrate buffer were purchased from Thermo Scientific (MA, USA). 9-(dicyanovinyl)julolidine (DCVJ) and Octadecyl rhodamine B chloride were purchased from Sigma-Aldrich (St. Louis, MO, USA). R 2/2 Quantifoil Cu grid was purchased from Ted Pella, Inc (Redding, CA, USA). Lipopolysaccharide (LPS) from Escherichia coli O26:B6 (600,000 EU/mg, as provided by the manufacturer) was purchased from Sigma (MO, USA). Luminex plates were purchased from Thermo Fisher Invitrogen (MA, USA) and ELISA kits were purchased from R&D systems (NE, USA). Peripheral blood mononuclear cells (PBMCs) were isolated from fresh leukopaks obtained from healthy donors (Stemcell Technologies, MA, USA). Human serum was purchased from STEMCELL Technologies (Cat. No. 200-0159). Human TLR4 Reporter HEK293 Cells (hkd-htlr4) and QUANTI-Luc™ were purchased from InvivoGen (CA, USA). The platelet Activating Factor (PAF) ELISA Kit was purchased from MyBioSource (CA, USA). Wild-type BALB/c mice and C57BL/6 mice were purchased from Jackson Lab (CA, USA).

1.2 Instruments

^{1}H and ^{13}C NMR spectra were acquired using either a Bruker NEO 500 or JEOL 400 console at the College of Chemistry NMR Facility, UC Berkeley. Chemical shifts are reported in parts per million (ppm) with residual solvent peaks as internal references. Data are presented as follows: chemical shift, multiplicity (s = singlet, d = doublet, t = triplet, q = quartet, m = multiplet), coupling constants (J-values in Hz), and integration. Mass spectra were obtained at the QB3/Chemistry Mass Spectrometry Facility, UC Berkeley. Absorbance measurements were made with a Tecan Infinite M200 plate reader (Männedorf, Switzerland). LNP/mRNA complexes were prepared using either NanoAssemblr™ Ignite (DE, USA) or PreciGenome microfluidic flow devices (San Jose, CA, USA). Flow cytometry experiments were performed with a Thermo Fisher Attune flow cytometer (MA, USA). Cellular trafficking was imaged by confocal microscopy (ImageXpress, San Jose, CA, USA).

1.3 Statistics and reproducibility

All statistical analyses were performed using GraphPad Prism 10. A significance level of α = 0.05 was applied for all significance tests. A one-way ANOVA followed by Dunnett, Holm-Šídák, or Tukey post hoc tests were used to assess differences between treatment groups for experiments involving a single variable with multiple biological replicates. A two-way ANOVA followed by Šidák post hoc test was used to assess differences between treatment groups for experiments involving two variables with multiple biological replicates. An unpaired one-tailed T-test was used to assess differences between a treatment group and a control group. Survival curves were analyzed using the Log-rank (Mantel–Cox) test. P values were described using the * notation, where * = $P < 0.05$, ** = $P < 0.01$, *** = $P < 0.001$ and **** = $P < 0.0001$. Sample sizes were not predetermined using statistical methods, and no data was excluded from the analyses. Except where otherwise indicated, the experiments were not randomized, and investigators were aware of the group allocations during the experiments and outcome assessments.

2. Synthetic procedures for the S-lipids

LNPs that are negatively charged at pH 7.4 have great potential for lowering the toxicity, but they have been challenging to develop because negatively charged materials are inefficient at encapsulating nucleic acids and triggering endosomal release. The S-lipids are designed to overcome these challenges by having a head-group composed of a carboxylate and tertiary amine and lipid tails that are engineered to trigger endosomal release efficiently and form stable LNPs. However, the S-lipids are an unexplored family of ionizable lipids and their structure-activity relationship (SAR) for encapsulating nucleic acids and transfecting cells is unknown, which is essential for developing S-lipids that can deliver nucleic acids efficiently.

To address this problem, we determined the SAR of the S-lipids for delivering nucleic acids by synthesizing a library of 144 S-lipids and screening them for their ability to encapsulate mRNA and transfect cells. The S-lipid library was synthesized by reacting a fragment that contained an amine and carboxyl with epoxides and acrylates that were engineered to form S-lipids that efficiently disrupted endosomes and formed stable LNPs. In particular, the epoxide/acrylates contained branched hydrophobic tails and a hydrocarbon spacer between their acrylate/epoxide warheads and branched hydrophobic tails to enhance their ability to trigger endosomal release and increase LNP stability[1,2]. The epoxide and acrylate intermediates used to make the S-lipids were synthesized in a 3-step synthetic procedure, see pages 10-14 of the SI for details. The conversion rate of the amine alkylation

step in the S-lipid synthesis was > 90% and this allowed crude S-lipids to be formulated into SNPs and screened on cells directly for their transfection activity (see Figure 2C-D). Top S-lipid hits from the HEK cell screening were then purified and further evaluated.

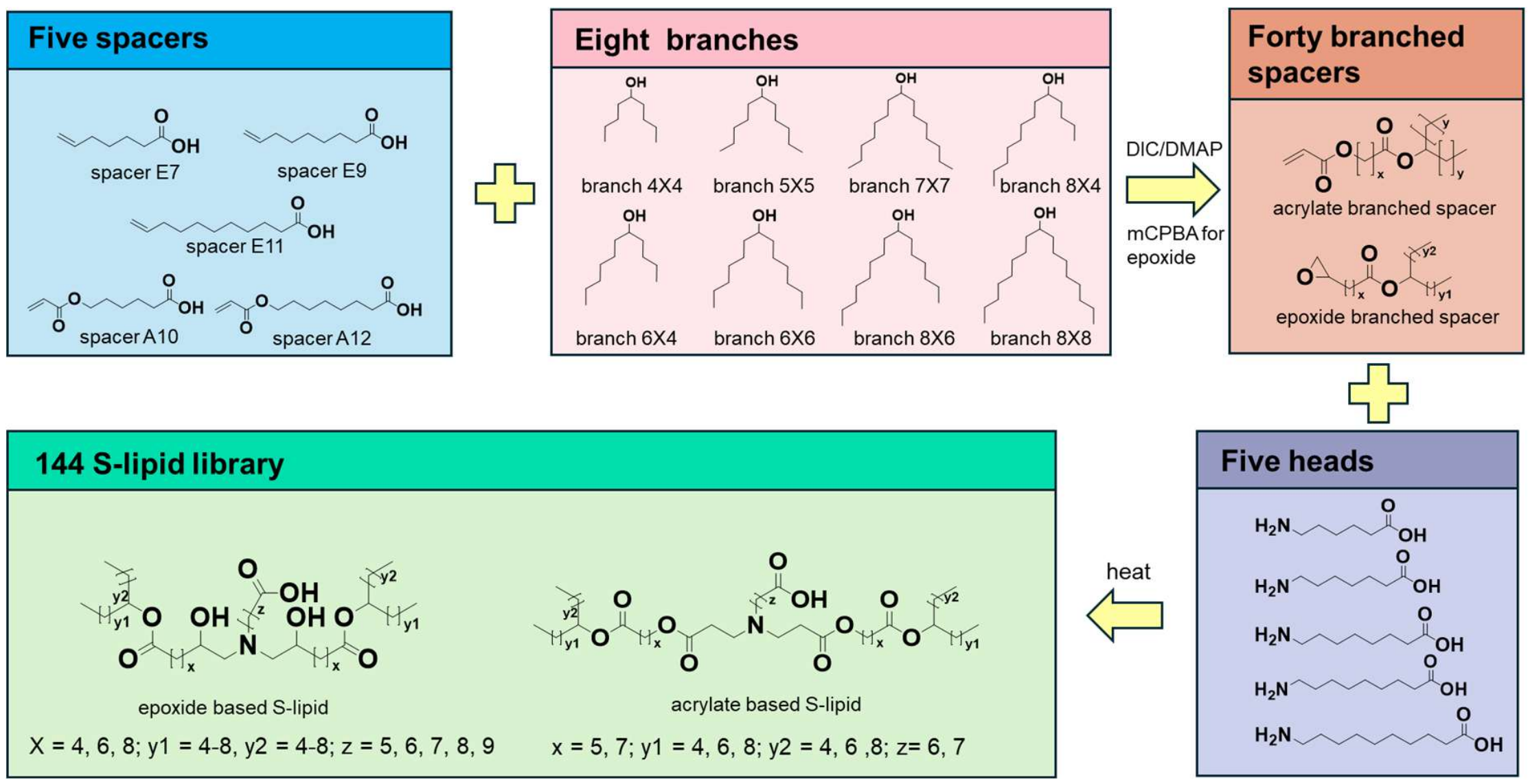

Figure S1. General synthetic scheme for the synthesis of the S-lipid library.

A library of S-lipids was synthesized via the Michael addition reaction and the epoxide ring-opening reaction. A schematic diagram of the synthetic scheme used to generate the S-lipid library is shown. 5 spacers (blue box) were combinatorially reacted with 8 branches (pink box) to generate 36 branched spacers (brown box). The S-lipid library was synthesized by reacting the 36 branched spacers with 5 amine-carboxyl headgroups (purple box). The chemical structures of the S-lipid library, including acrylate-based S-lipids and epoxide-based S-lipids, are shown in the green box.

2.1 General synthetic procedures for the epoxide-based S-lipids (E1-E120)

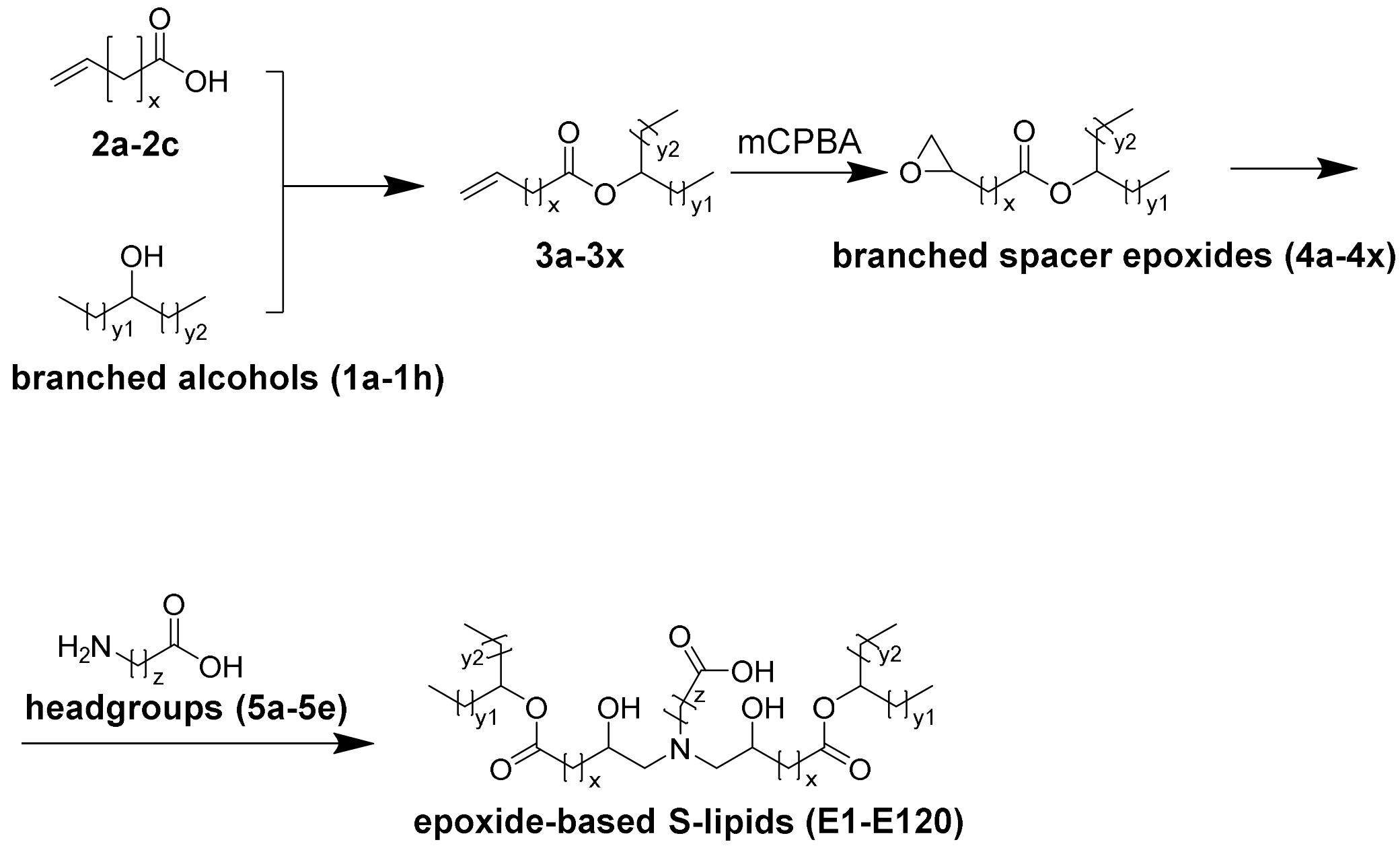

Figure S2. Synthesis of epoxide-based S-lipids (E1-E120).

Epoxide-based S-lipids **E1-E120** were synthesized via the epoxide ring-opening reaction between the branched spacer epoxides **4a-4x** and the headgroups **5a-5e**, as shown in Figure S2. A three-step synthetic procedure was employed to generate a library of 120 epoxide-based S-lipids (**E1-E120**). The amine-carboxylate headgroups **5a-5e** contain carbon spacers ranging from 5 to 9 atoms in length. The branched spacer epoxides **4a-4x** feature a hydrocarbon spacer positioned between the epoxide warhead and the ester group. To synthesize these epoxide-based S-lipids, **2a-2c** were esterified with branched alcohols **1a-1h** to yield branched spacers **3a-3x**. These intermediates **3a-3x** were then oxidized to generate the branched spacer epoxides **4a-4x**, which were subsequently heated with headgroups **5a-5e** to afford the final epoxide-based S-lipids (**E1-E120**). Top performing members of the epoxide-based S-lipid library are shown in Figure S6.

Synthesis of branched spacer epoxides (4a-4x)

To a solution of **2a-2c** (1.5 eq, 1.75 mmol), N,N'-diisopropylcarbodiimide (DIC, 295 mg, 366 µL, 2 eq, 2.34 mmol), and 4-dimethylaminopyridine (DMAP, 28.6 mg, 0.2 eq, 234 µmol) in dichloromethane (40 mL), **1a-1h** (1 eq, 1.17 mmol) were added. The reaction mixtures were stirred at room temperature for 16 hours, then filtered and concentrated under reduced pressure. The residues were redissolved in hexane and ethyl acetate (50 mL) at a 5:1 ratio. The mixtures were subsequently washed with brine (3 × 30 mL), and the organic layer was

dried over magnesium sulfate and concentrated under reduced pressure. The residue was purified by flash column chromatography (25:1 hexanes/EtOAc) to afford **3a-3x** as colorless liquids which were directly used for the next step. To a solution of mCPBA (2.00 g, 55% wt, 2 eq, 6.38 mmol) in dichloromethane (20 mL), **3a-3x** (1 eq, 3.19 mmol) were added. The reactions were stirred for 16 hours at room temperature. The mixtures were diluted with dichloromethane (20 mL) and washed with saturated sodium thiosulfate (50 mL) to remove unreacted mCPBA until the potassium iodide starch test showed no color change. The organic layer was washed with saturated sodium carbonate solution to remove the 3-chlorobenzoic acid side product, dried over magnesium sulfate, and concentrated. The crude samples were purified by flash column chromatography (20:1 hexanes/EtOAc) to afford **4a-4x** as colorless liquids. The overall yields for the two steps ranged from 28% to 77%.

Synthesis of epoxide-based S-lipids (E1-E120)

A mixture of branched spacer epoxides **4a-4x** (1 eq, 17.22 µmol) and headgroups **5a-5e** (2.2 eq, 37.88 µmol) in 100 µL of N-methyl-2-pyrrolidinone in a 2 mL glass vial was heated at 90 C° and stirred for 3 days. The conversions were more than 90% as determined by mass spectrometry. Crude S-lipids **E1-E120** were cooled to room temperature, dissolved in EtOH at 15 mM, and directly used for initial LNP screening in HEK cells. The top hits were purified by flash column chromatography using methanol (0.1% H2O) and DCM at a gradient from 0 to 15%. The resulting products were concentrated and further sequentially extracted by hexane, EtOAc, and MeOH, the solvent was removed under reduced pressure after each extraction step. The yields after purification ranged from 10% to 35%.

2.2 General synthetic procedures for the acrylate-based S-lipids

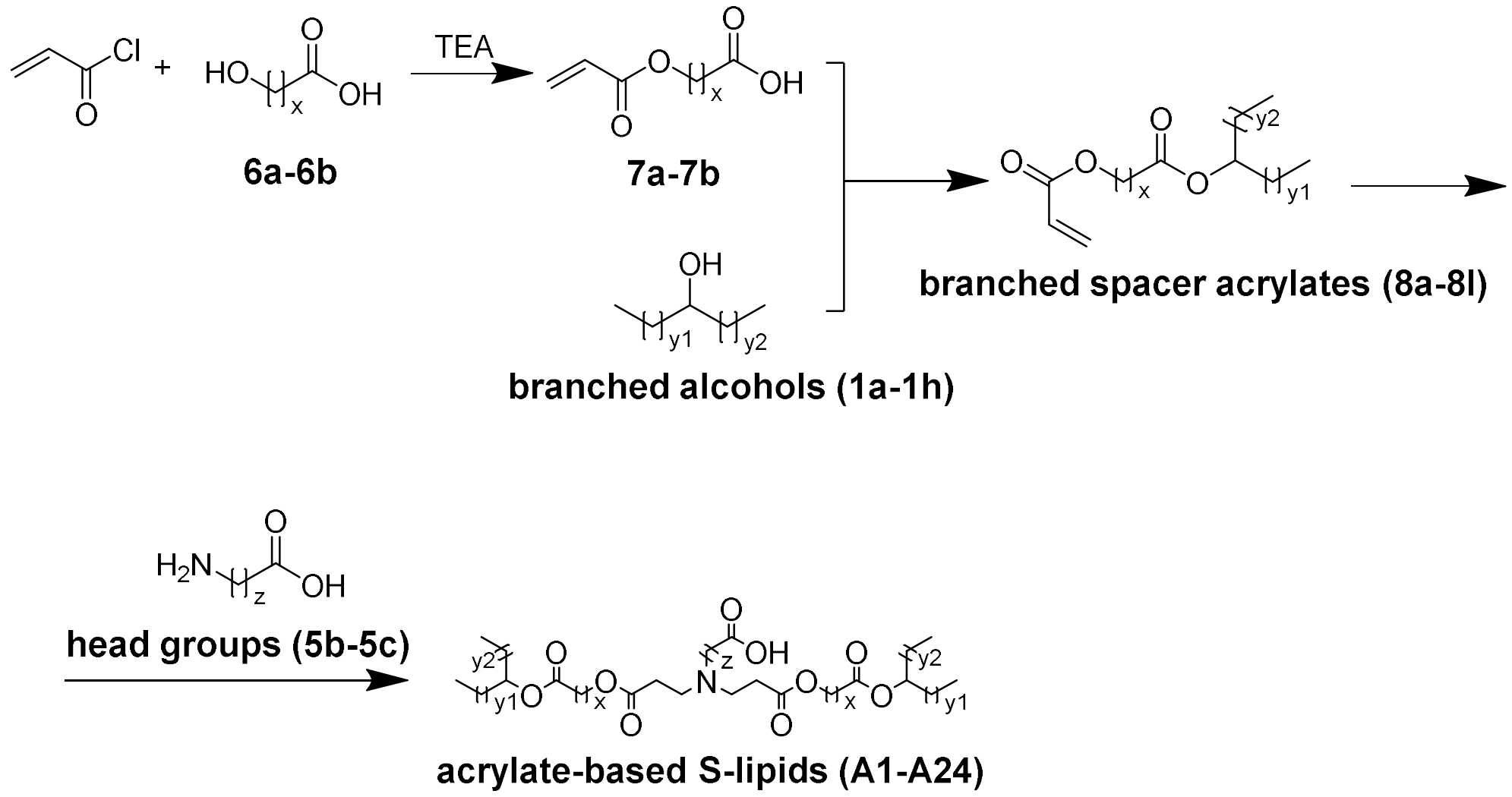

Figure S3. Synthesis of acrylate-based S-lipids (A1-A24).

The synthetic strategy for making **A1-A24** was based on the Michael addition reaction. Headgroups **5b-5c** were reacted with the branched spacer acrylates **8a-8l** as shown in Figure S3. A three-step synthesis was employed to generate a library of 24 acrylate-based S-lipids (**A1-A24**). Some members of the acrylate-based S-lipids **A1-A24** are shown in Figure S6. The amine-carboxylate headgroups **5b-5c** used in the synthesis have carbon spacers ranging from 6 to 7 atoms in length. The branched spacer acrylates **8a-8l** contain a hydrocarbon spacer positioned between the acrylate warhead and the ester group. **8a-8l** were synthesized by esterifying **6a-6b** with acryloyl chloride to yield intermediates **7a-7b**. The products were subsequently esterified with branched alcohols **1a-1h** to obtain the branched spacer acrylates **8a-8l**. The final acrylate-based S-lipids (**A1-A24**) were synthesized by heating the acrylates **8a-8l** with headgroups **5b-5c**.

Synthesis of branched spacer acrylates 8a-8l

To a mixture of **6a-6b** (1 eq, 7.5 mmol) and triethylamine (4 mL, 4 eq, 30 mmol) in dichloromethane (10 mL) in a 50 mL round-bottom flask at 0 °C, acryloyl chloride (1.2 mL, 2 Eq, 15 mmol) was added dropwise. The reactions were stirred at 0 °C for 2 hours, followed by stirring at room temperature for another 2 hours. The mixtures were diluted with dichloromethane (50 mL) and washed with brine (3 x 30 mL). The organic layers were concentrated under reduced pressure, and the residues were purified by flash column chromatography (5:1 hexanes/EtOAc) to afford **7a-7b** as colorless liquids. To a solution of

intermediates **7a-7b** (1.05 eq, 1.07 mmol), N,N'-diisopropylcarbodiimide (DIC, 258 mg, 320 µL, 2.00 eq, 2.05 mmol) and 4-dimethylaminopyridine (DMAP, 25.0 mg, 0.20 eq, 205 µmol) in dichloromethane (4 mL), branched alcohols **1a-1h** (1.00 eq, 1.02 mmol) were added. The reaction mixtures were stirred under a nitrogen atmosphere overnight. The mixtures were then diluted with dichloromethane (30 mL) and washed with brine (3 × 20 mL). The organic layers were concentrated under reduced pressure, and the residues were purified by flash column chromatography (20:1 hexanes/EtOAc) to afford the branched spacer acrylates **8a-8l** as colorless liquids. The overall yields for the two steps ranged from 33% to 52%.

Synthesis of acrylate-based S-lipids (A1-A24)

Mixtures of headgroups **5b-5c** (1 eq, 17.22 µmol) and branched spacer acrylates **8a-8l** (2.2 eq, 37.88 µmol) in a 100 µL of N-methyl-2-pyrrolidinone were heated at 90 C° in 2 mL glass vials and stirred for 3 days. The conversions were more than 90% as determined by mass spectrometry. The crude S-lipids **A1-A24** were cooled to room temperature, dissolved in EtOH at 15 mM, and directly used for initial LNP screening in HEK cells.

The top hits from the cell screening experiments were resynthesized and purified via flash column chromatography. For the top hits, the reaction was repeated as described above, except that 2.4 equiv. of the branched spacer acrylate **8a** and **8g** were used in the Michael addition reaction to ensure complete conversion. The final lipid products were purified by flash column chromatography using methanol (0.1% H2O) and DCM at a gradient from 0 to 15%. The resulting products were concentrated and further sequentially extracted with hexane, EtOAc, and MeOH, the solvent was removed under reduced pressure after each extraction step. The yields after purification ranged from 28% to 36%.

2.3 General synthetic procedure for branched alcohols

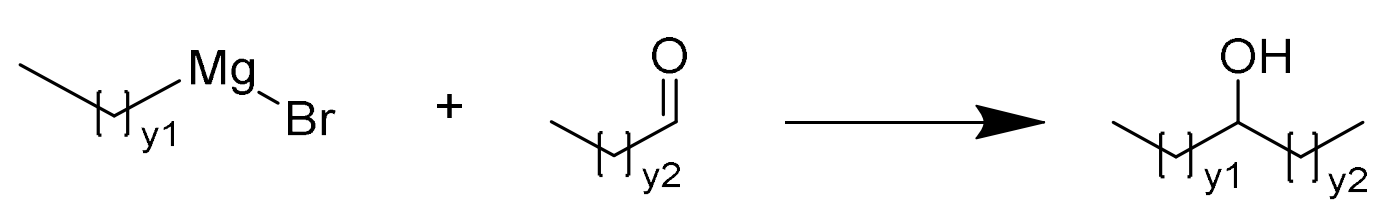

9a-9e **10a-10e** **branched alcohols (1a-1h)**

Figure S4. General procedure for the synthesis of branched alcohols.

9a-9e (1 Eq, 30 mmol) were dissolved in dry THF (20 mL) in 250mL round bottom flasks and cooled to -78 °C under a nitrogen atmosphere. **10a-10e** (1 molar in THF, 1.2 eq, 36 mmol) was added dropwise to the solution at -78 °C. The reaction mixture was then stirred at -78 °C for

15 minutes and then stirred at room temperature for an additional 1 hour. The mixtures were cooled back to –78 °C, and the pH was adjusted to 4 using 1 M HCl. The resulting mixtures were extracted with EtOAc (3 x 25 mL). The organic layers were combined and dried over magnesium sulfate, filtered, and concentrated under reduced pressure. The residues were purified by flash column chromatography (15:1 hexanes/EtOAc) to afford **1a-1h** as white solids or colorless liquids. The yields were all above 95%.

2.4 Synthetic procedure for the linear S-lipids

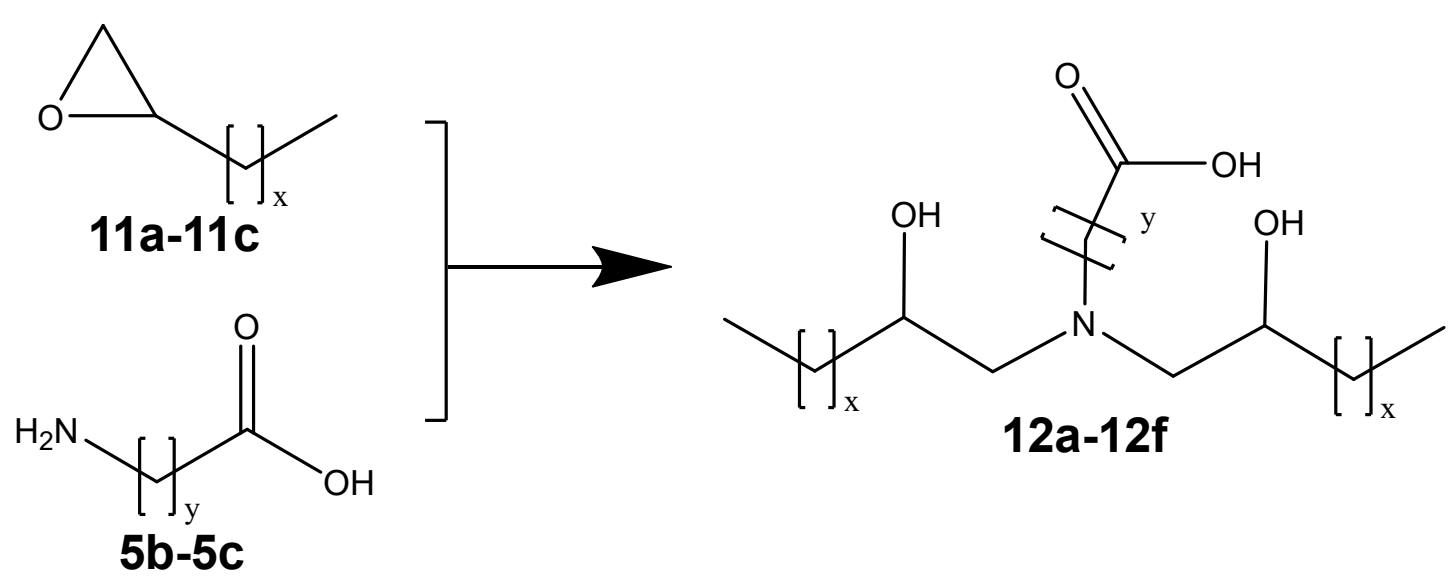

Figure S5. Synthesis of linear S-lipids (12a-12f).

An epoxide ring-opening reaction was performed between the commercially available epoxides 1,2-epoxytetradecane, 1,2-epoxyhexadecane, and 1,2-epoxyoctadecane (**11a-11c**) and headgroups **5b-5c** to generate **12a-12f** as shown in Figure S5. The amine-carboxylate headgroups **5b-5c** contain carbon spacers ranging from 6 to 7 atoms in length. To assemble the linear S-lipids, epoxides and **5b-5c** (2.2 eq, 37.88 μmol) were reacted in 100 μL of N-methyl-2-pyrrolidinone in a 2 mL glass vial, which was heated at 90 C° and stirred for 3 days. The conversions were more than 90% as determined by mass spectrometry. The crude S-lipids **12a-12f** were cooled to room temperature, dissolved in EtOH at 15 mM, and directly used for initial LNP screening in HEK cells.

2.5 Chemical structures of the top-performing S-lipids

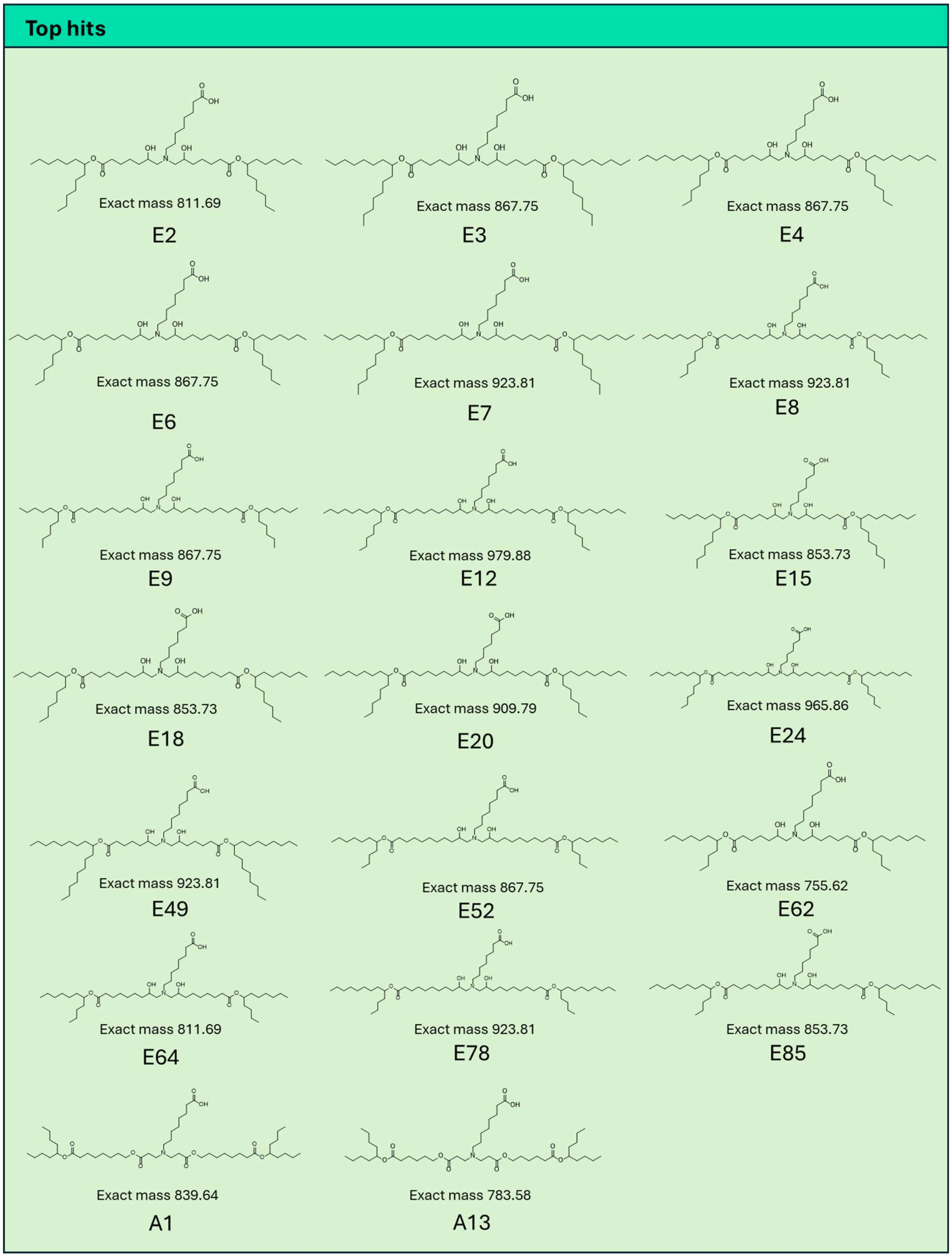

Figure S6. Chemical structures of the top-performing S-lipids.

2.6 Purity analysis of the top-performing S-lipids

LC-MS was employed to test the purities of the top-performing S-lipids. LC–MS analysis was performed on a Shimadzu HPLC system (Shimadzu, Kyoto, Japan) coupled to an Advion CMS mass spectrometer (Advion, Ithaca, NY, USA). HPLC was equipped with a Purospher® STAR RP-18 endcapped (5 µm, 100 mm) column (Merck, Darmstadt, Germany) and operated at a flow rate of 0.30 mL/min. The mobile phase consisted of H2O (0.1% FA) and MeOH (0.1% FA). The following gradient program was used: 85% MeOH (0–5 min), ramp to 100% MeOH (5–20 min), hold at 100% MeOH (20–49 min), return to 85% MeOH (49–53 min), and re-equilibrate at 85% MeOH (53–60 min). Delta Ion Current (ΔIC) was recorded for content determination, and m/z values were used for compound identification. ΔIC based on differential ion current plots provided improved signal-to-noise (S/N) compared to traditional Total Ion Current (TIC) chromatograms by enhancing the detected compound signal while minimizing the background signal.

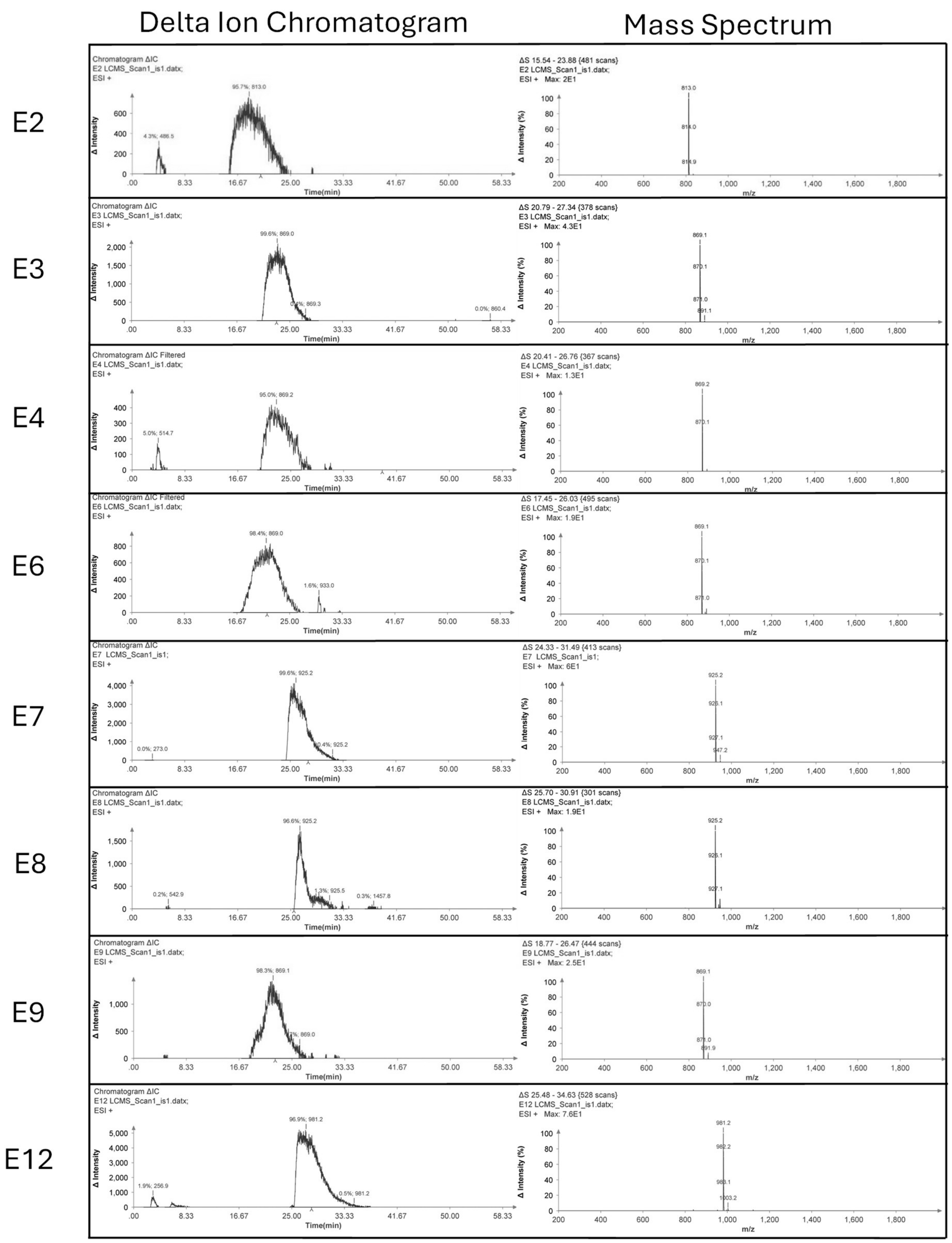

Figure S7. LCMS of top performing S-lipids part 1.

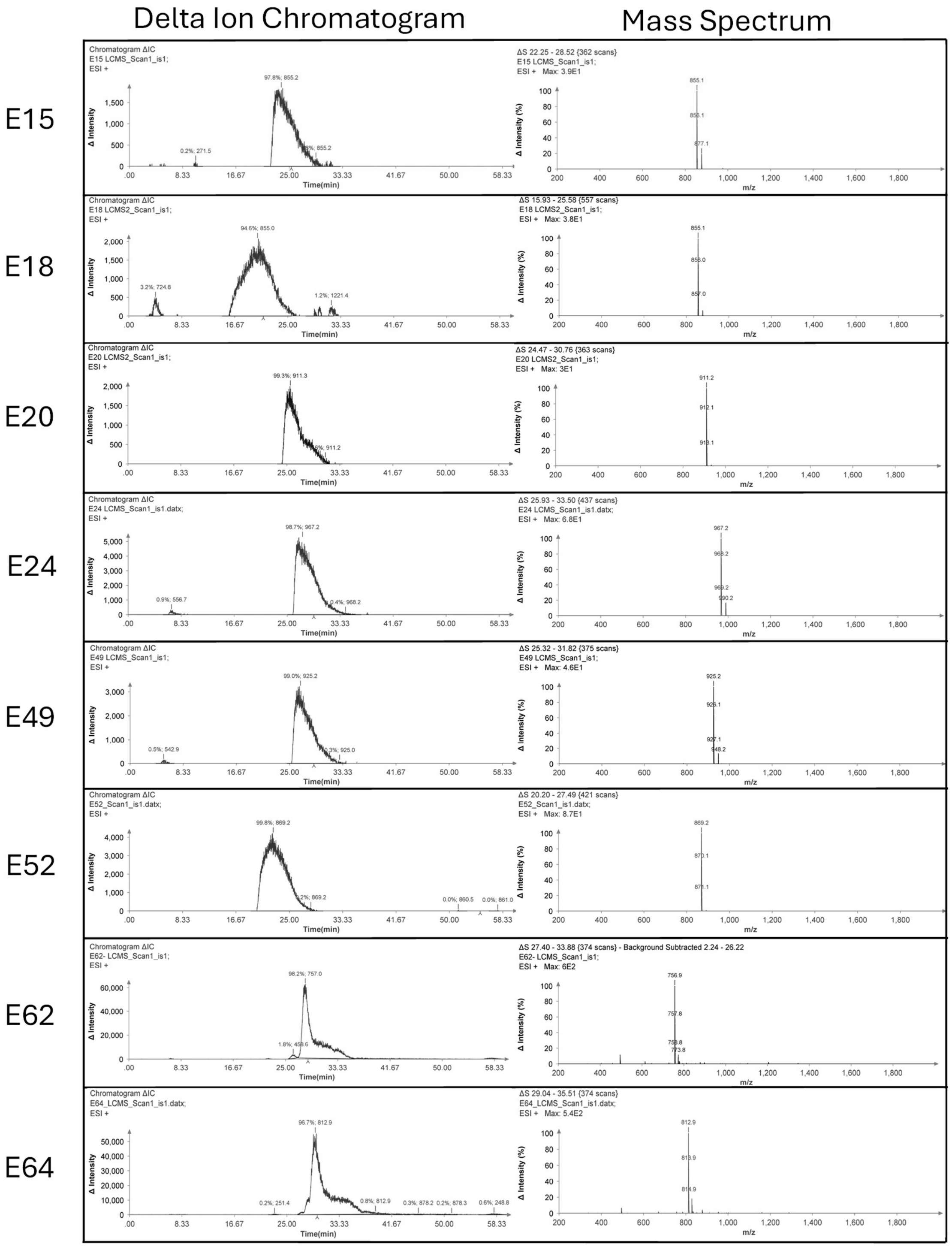

Figure S8. LCMS of top performing S-lipids part 2.

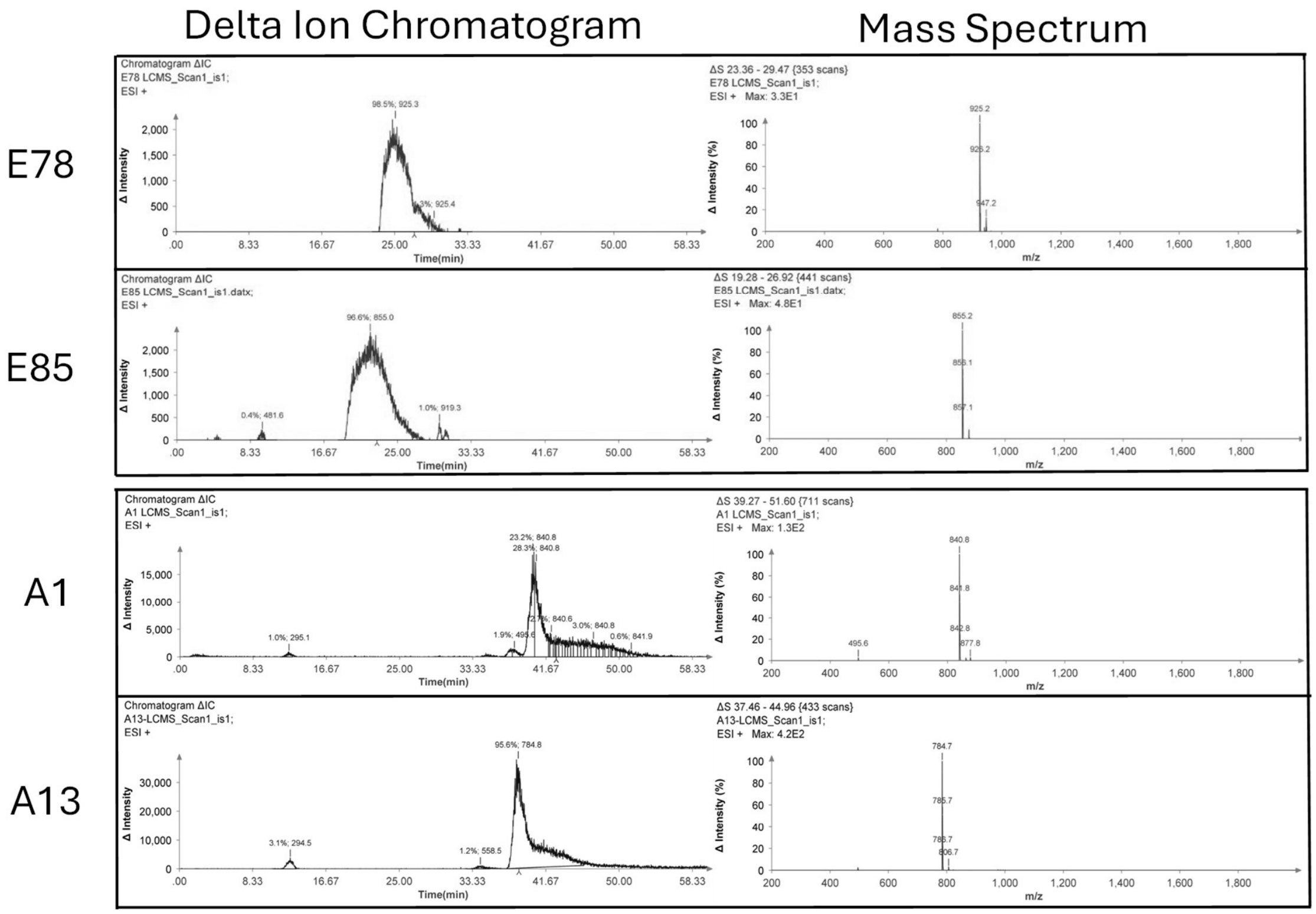

Figure S9. LCMS of top performing S-lipids part 3.

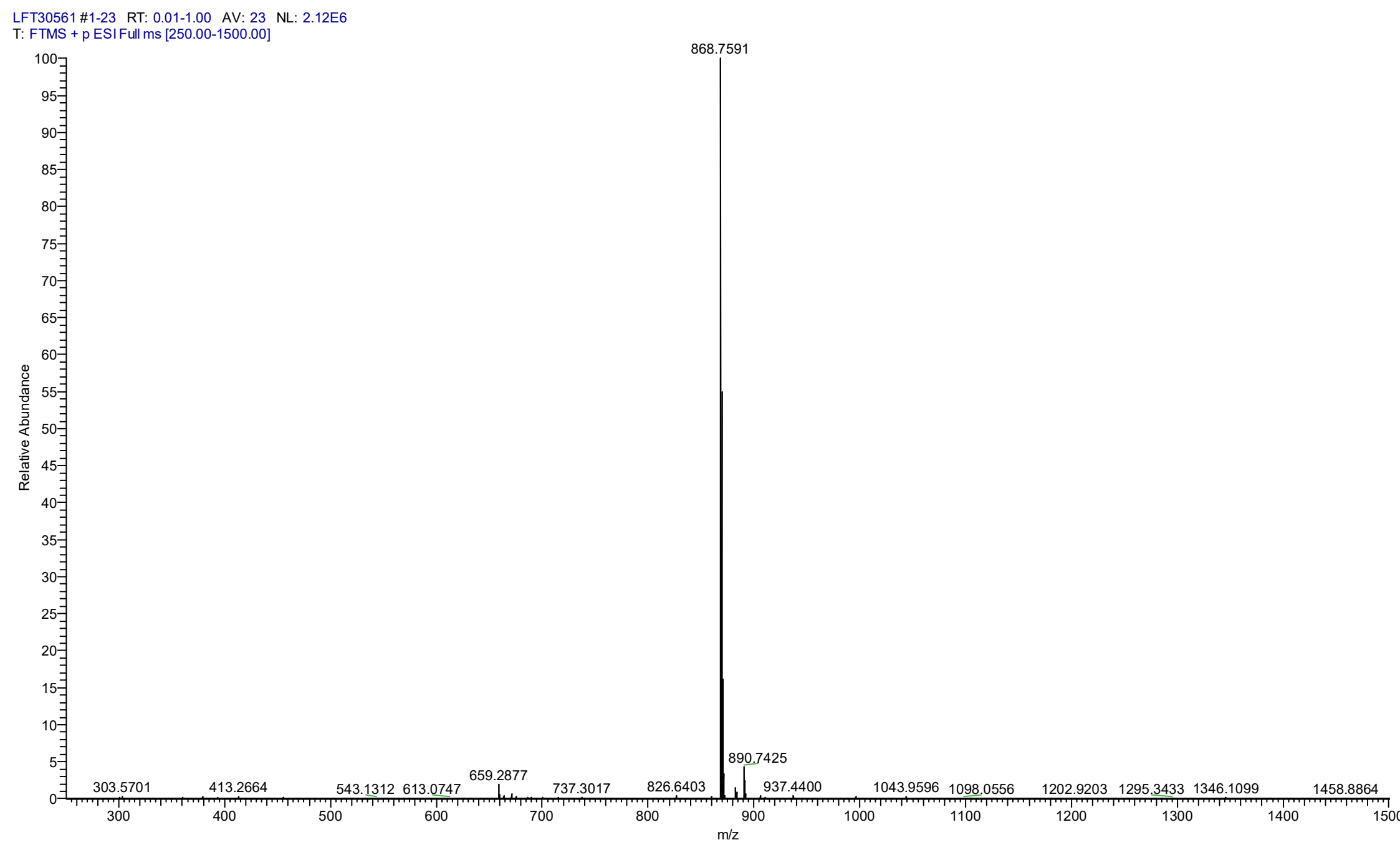

Figure S10. High-resolution mass spectrometry of S-lipid E3.

HRMS *m/z:* calcd for $C_{52}H_{102}NO_8$ $[M+H]^+$ 868.7600; found: 868.7591.

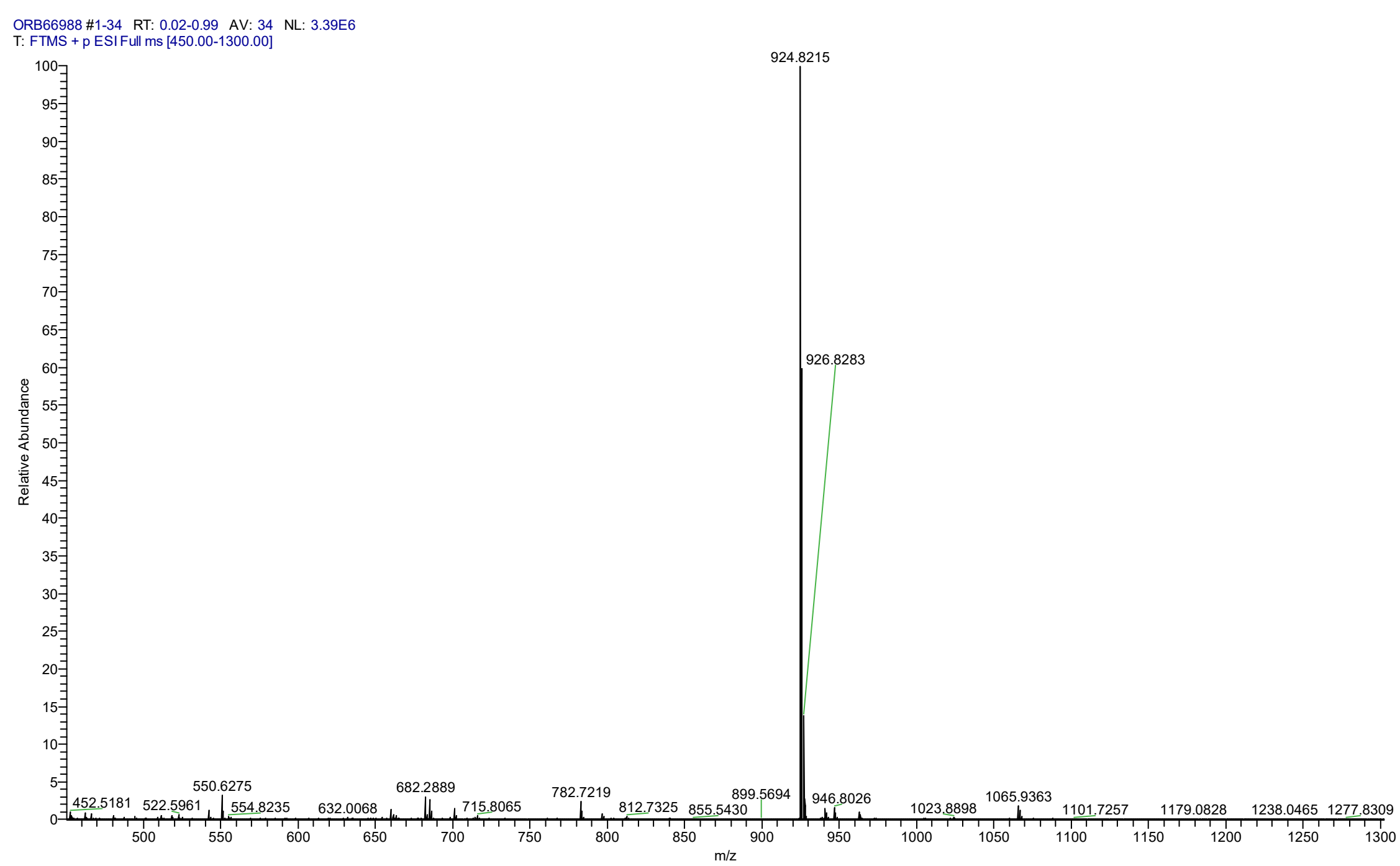

Figure S11. High-resolution mass spectrometry of S-lipid E7.

HRMS *m/z:* calcd for $C_{56}H_{110}NO_8$ $[M+H]^+$ 924.8226; found: 924.8215.

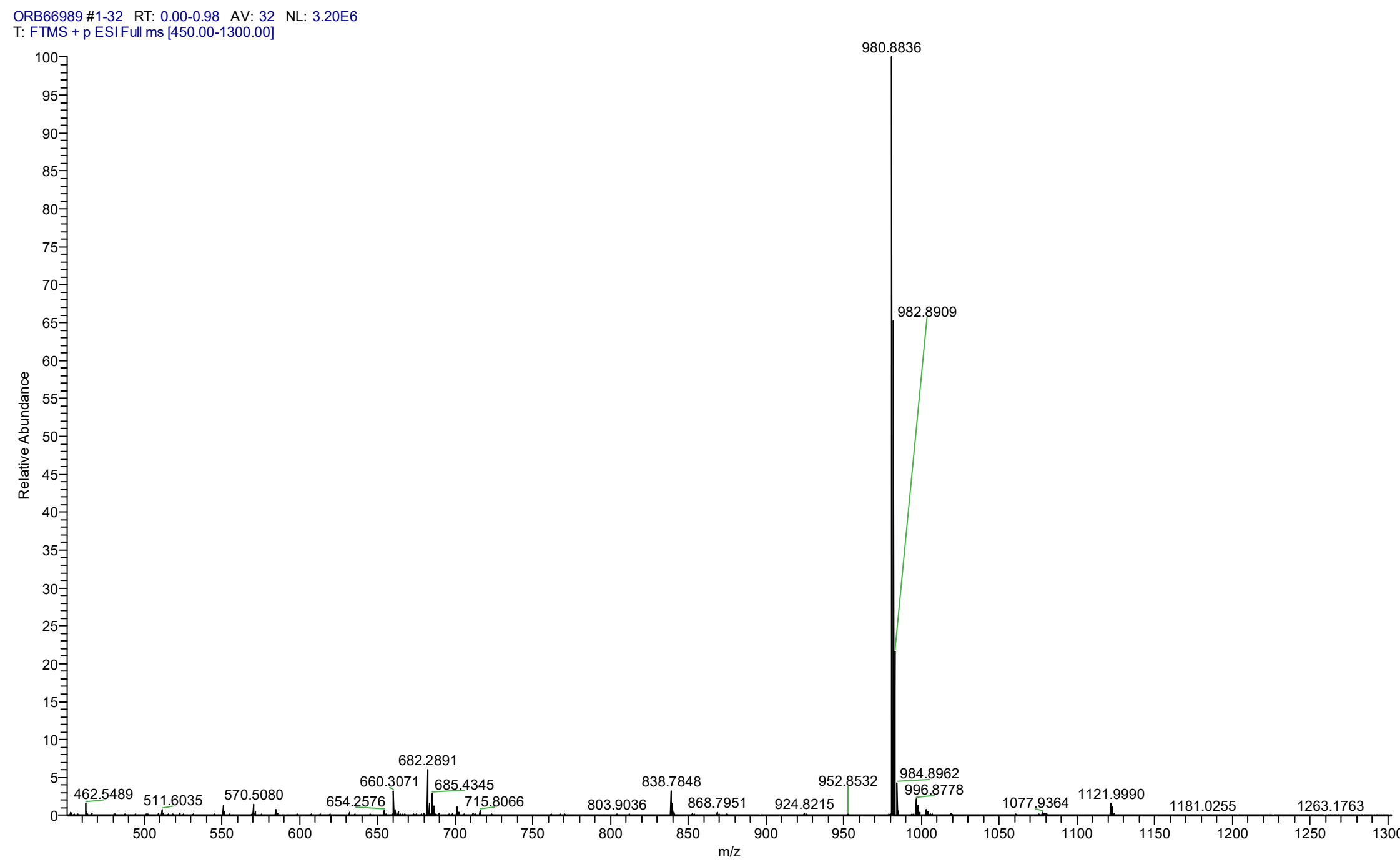

Figure S12. High-resolution mass spectrometry of S-lipid E12.

HRMS *m/z:* calcd for $C_{60}H_{118}NO_8$ $[M+H]^+$ 980.8852; found: 980.8836.

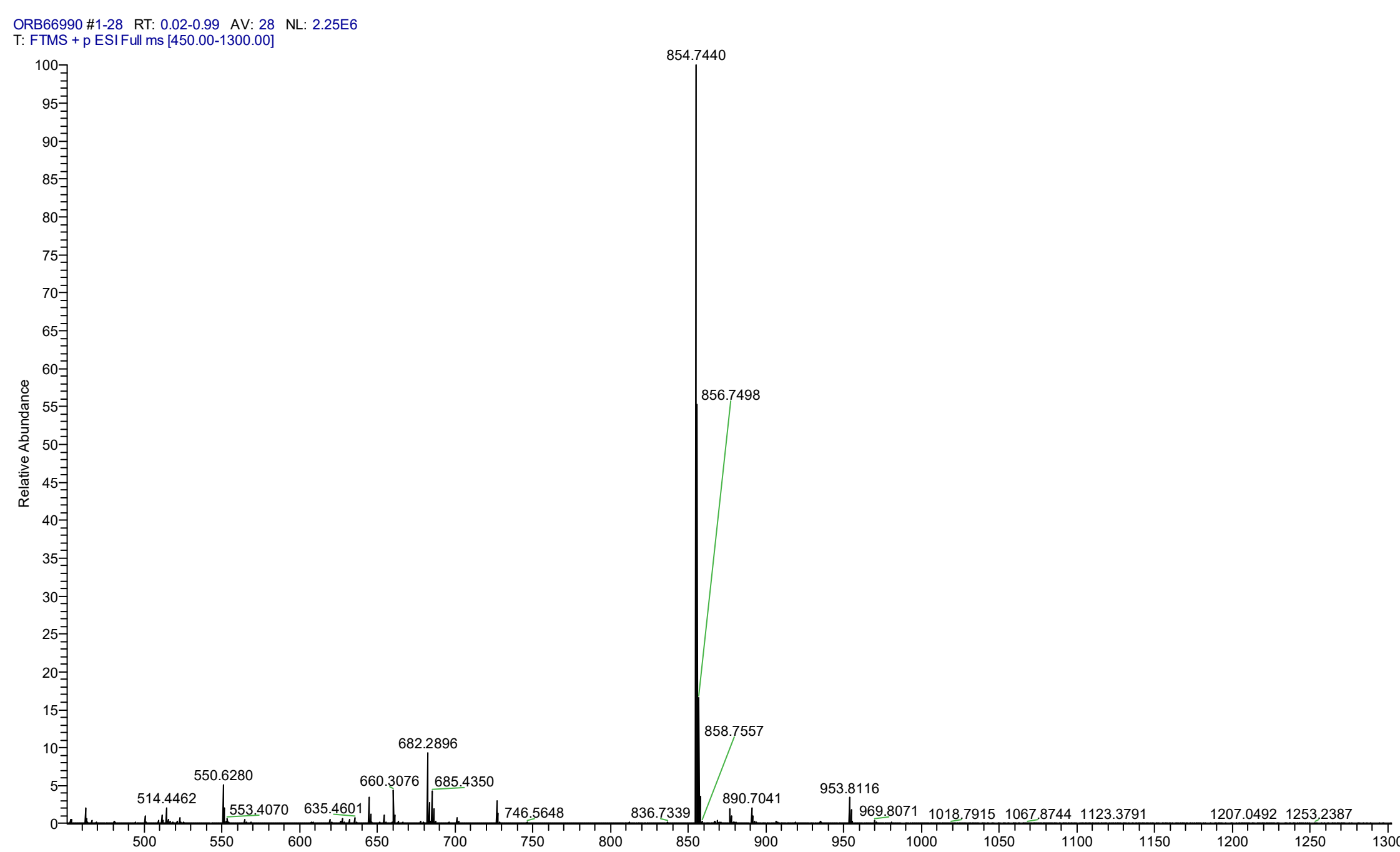

Figure S13. High-resolution mass spectrometry S-lipid E15.

HRMS *m/z:* calcd for $C_{51}H_{100}O_8N$ $[M+H]^+$ 854.7443; found: 854.7440.

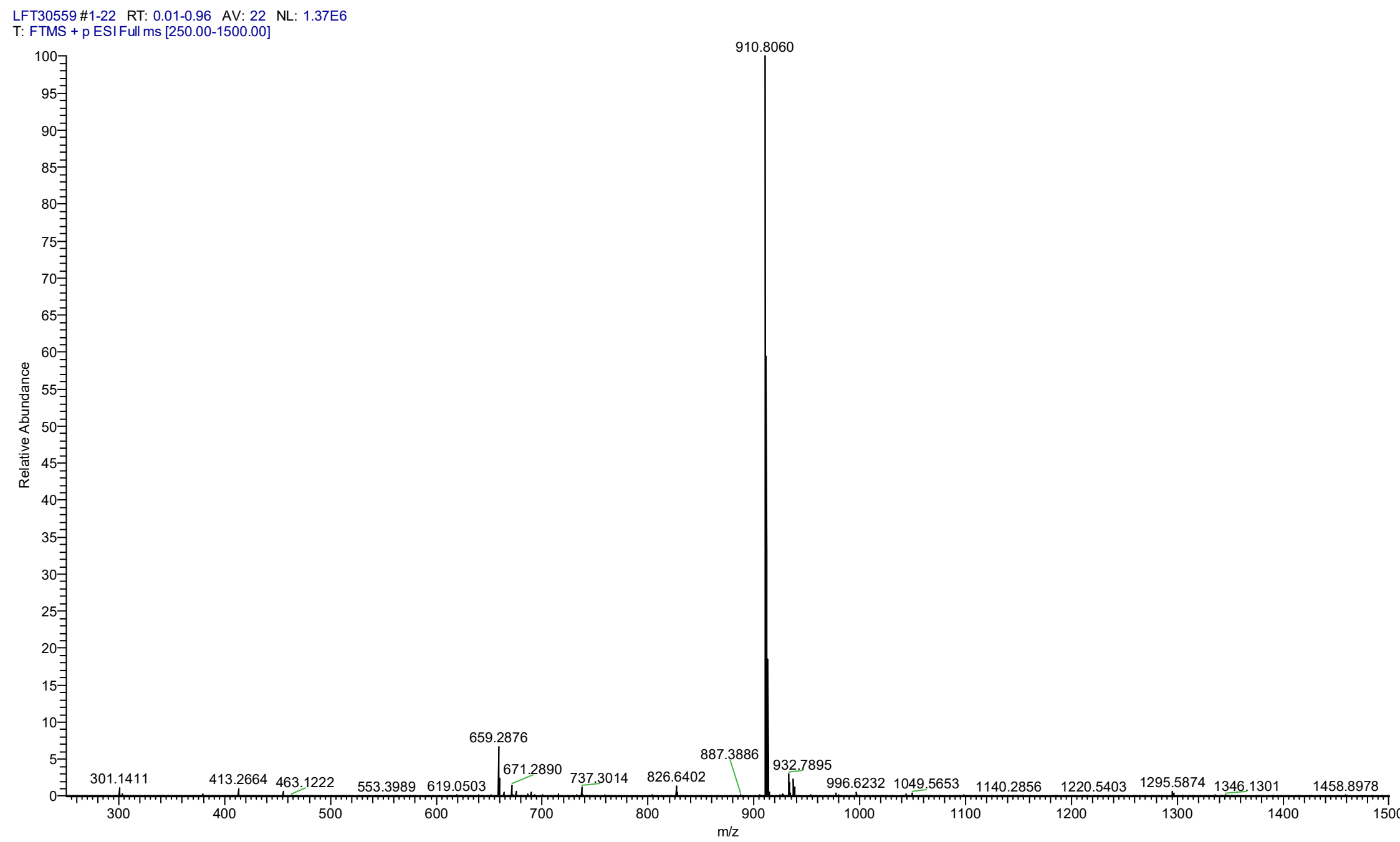

Figure S14. High-resolution mass spectrometry of S-lipid E20.

HRMS *m/z:* calcd for $C_{55}H_{108}NO_8$ $[M+H]^+$ 910.8069; found: 910.8060.

3. NMR spectra of compounds

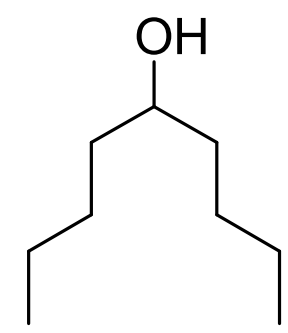

Figure S15. Chemical structure of compound 1a.

^{1}H NMR (500 MHz, $CDCl_3$) δ 3.61 (s, 1H), 1.51 – 1.29 (m, 13H), 0.97 – 0.90 (m, 6H).

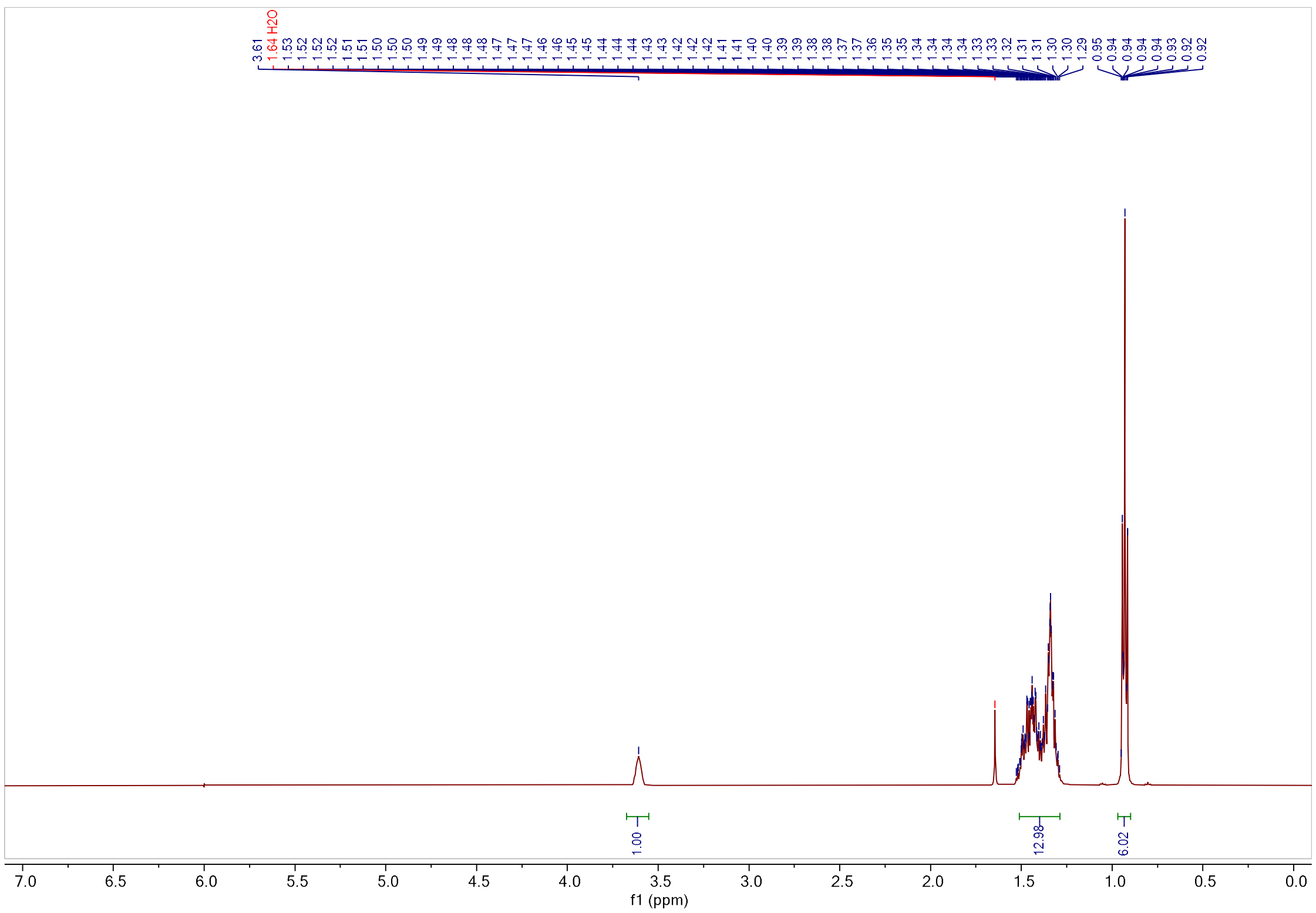

^{1}H NMR spectrum of compound **1a** ($CDCl_3$, 500 MHz)

^{13}C NMR (101 MHz, CHLOROFORM-*D*) δ 72.10, 37.26, 27.93, 22.88, 14.18.

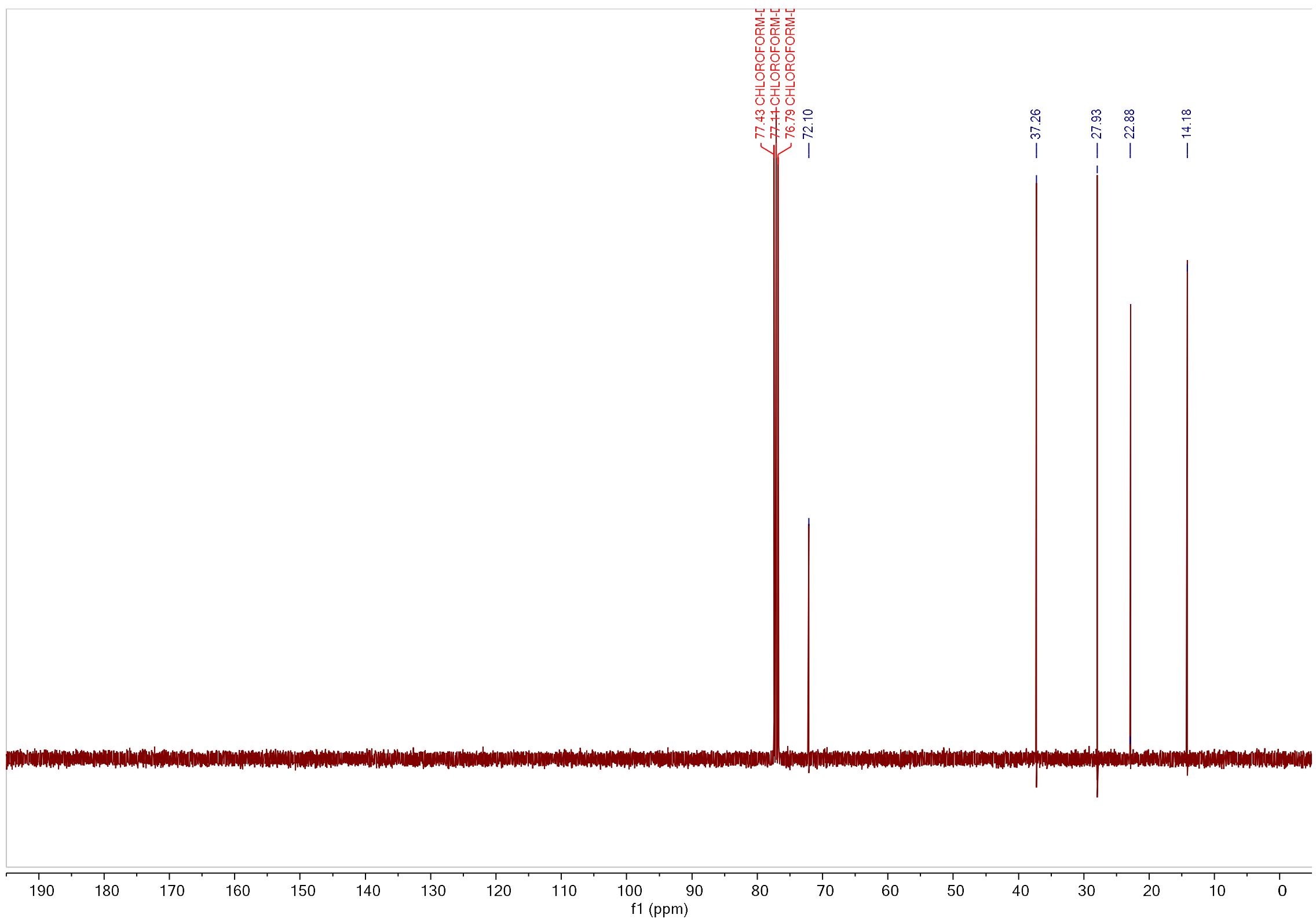

^{13}C-NMR spectrum of compound **1a** ($CDCl_3$, 101 MHz)

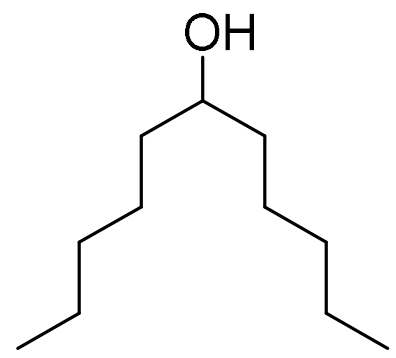

Figure S16. Chemical Structure of compound 1b.

^{1}H NMR (500 MHz, $CDCl_3$) δ 3.57 (s, 1H), 1.46 – 1.23 (m, 17H), 0.91 – 0.84 (m, 6H).

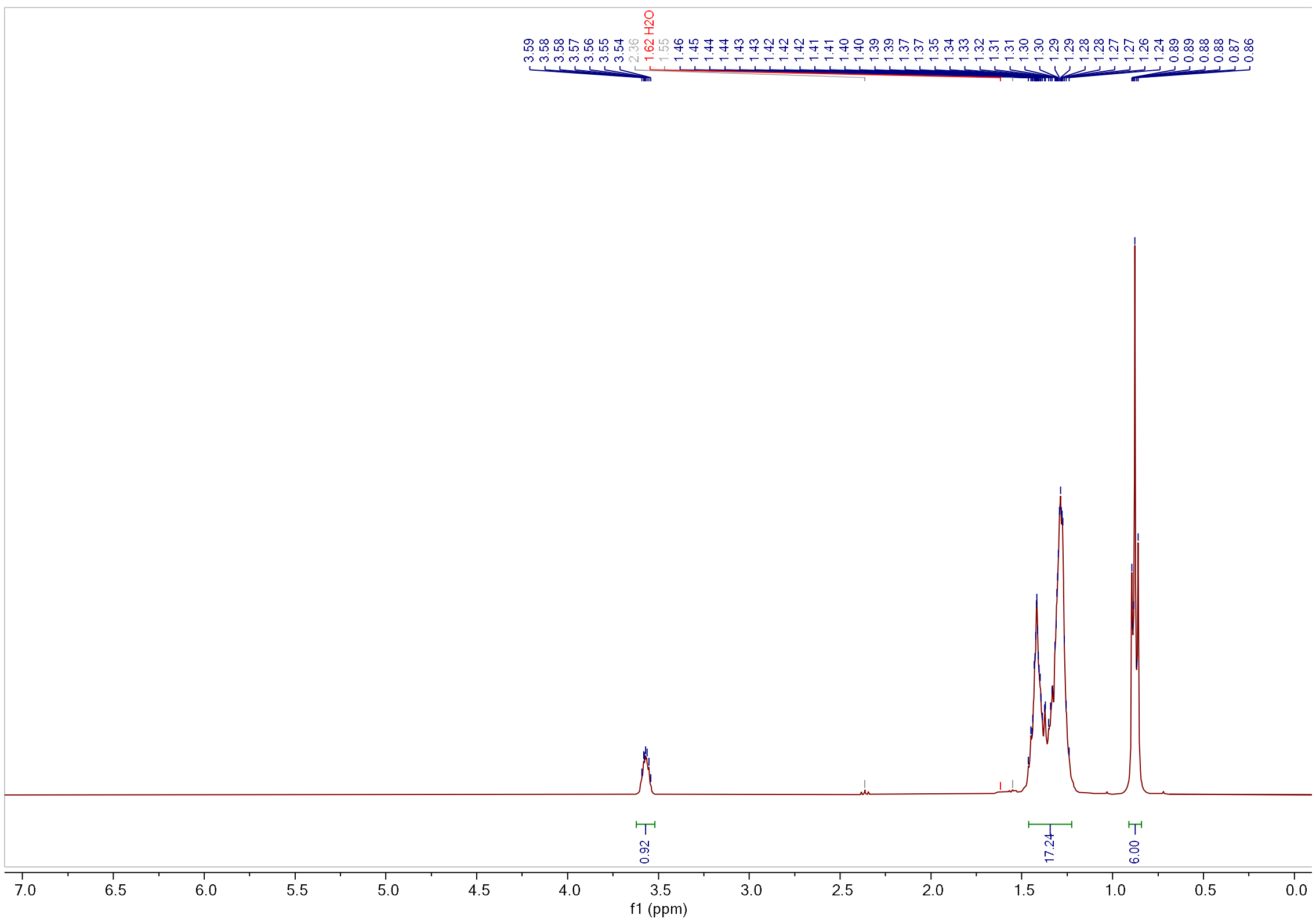

^{1}H NMR spectrum of compound **1b** ($CDCl_3$, 500 MHz)

^{13}C NMR (101 MHz, $CDCl_3$) δ 72.12, 37.54, 32.00, 25.41, 22.73, 14.12.

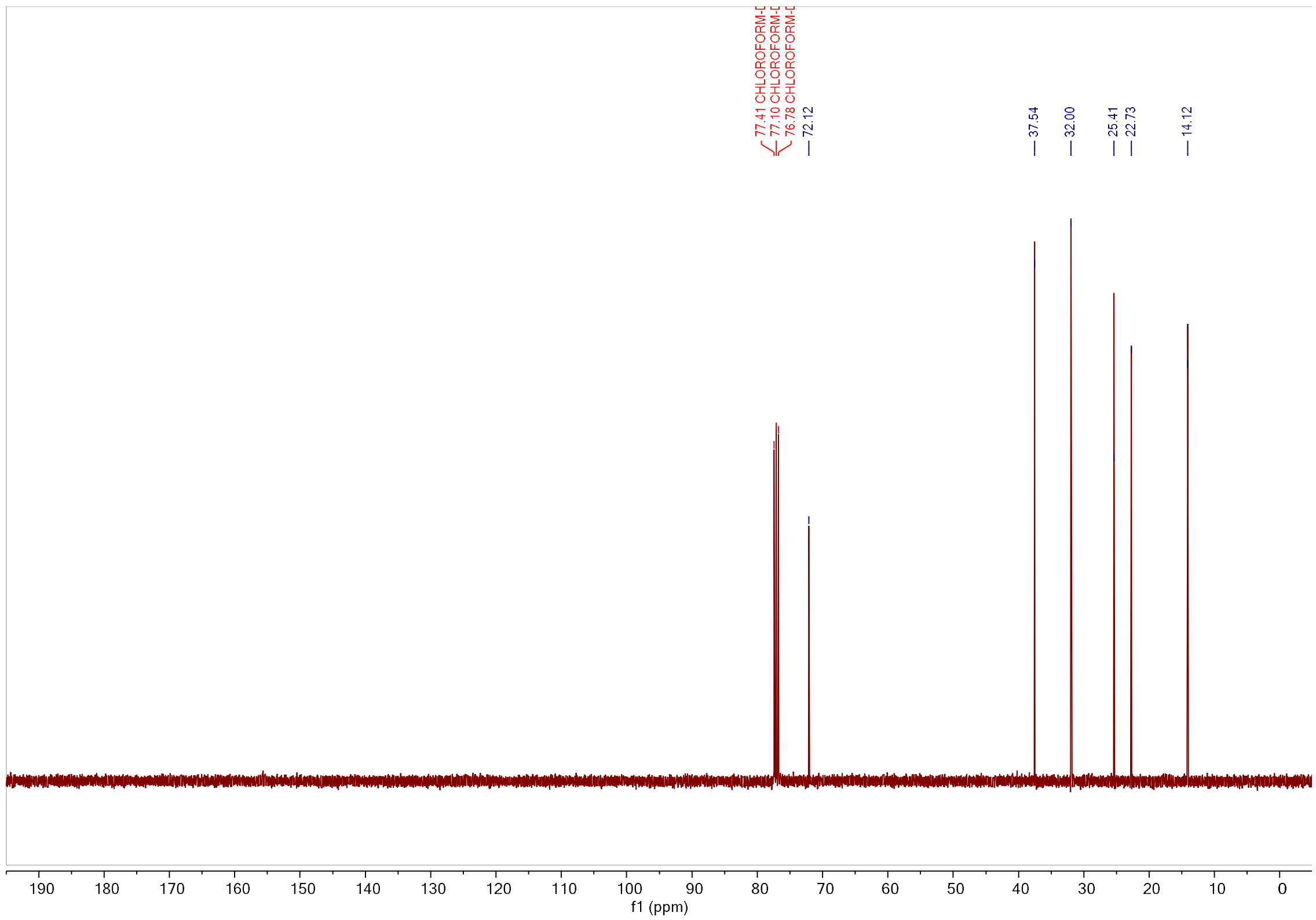

^{13}C-NMR spectrum of compound **1b** ($CDCl_3$, 101 MHz)

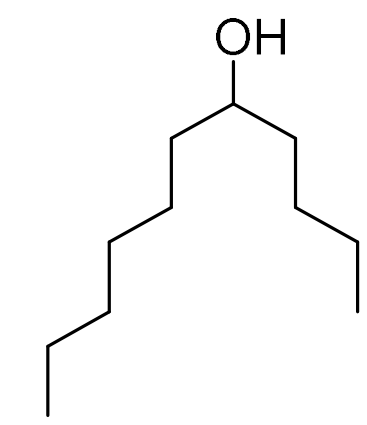

Figure S17. Chemical Structure of compound 1c.

^{1}H NMR (500 MHz, $CDCl_3$) δ 3.62 (s, 1H), 1.51 – 1.26 (m, 17H), 0.97 – 0.87 (m, 6H).

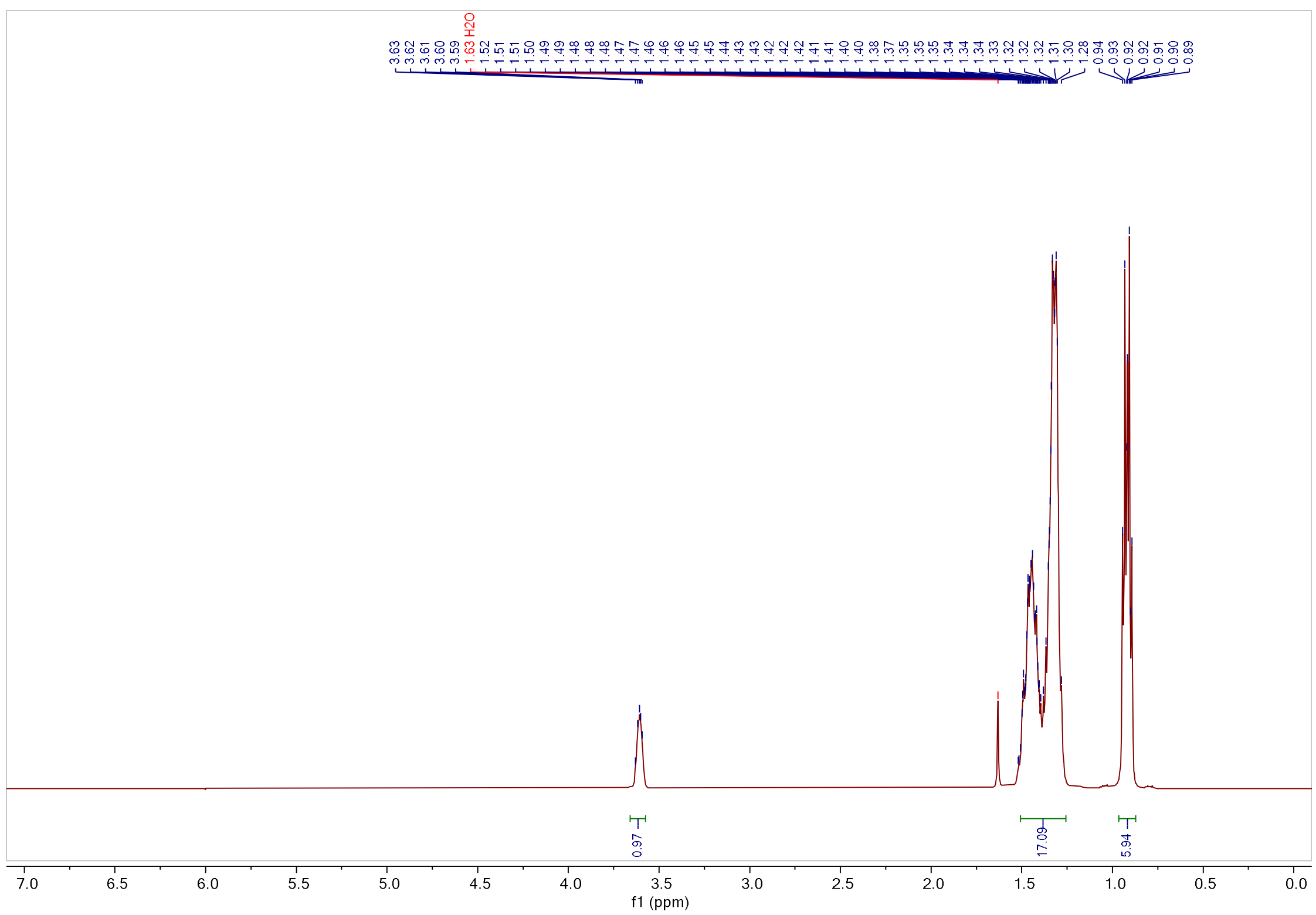

^{1}H NMR spectrum of compound **1c** ($CDCl_3$, 500 MHz)

^{13}C NMR (126 MHz, $CDCl_3$) δ 72.03, 37.51, 37.20, 31.86, 29.39, 27.86, 25.63, 22.78, 22.64, 14.09.

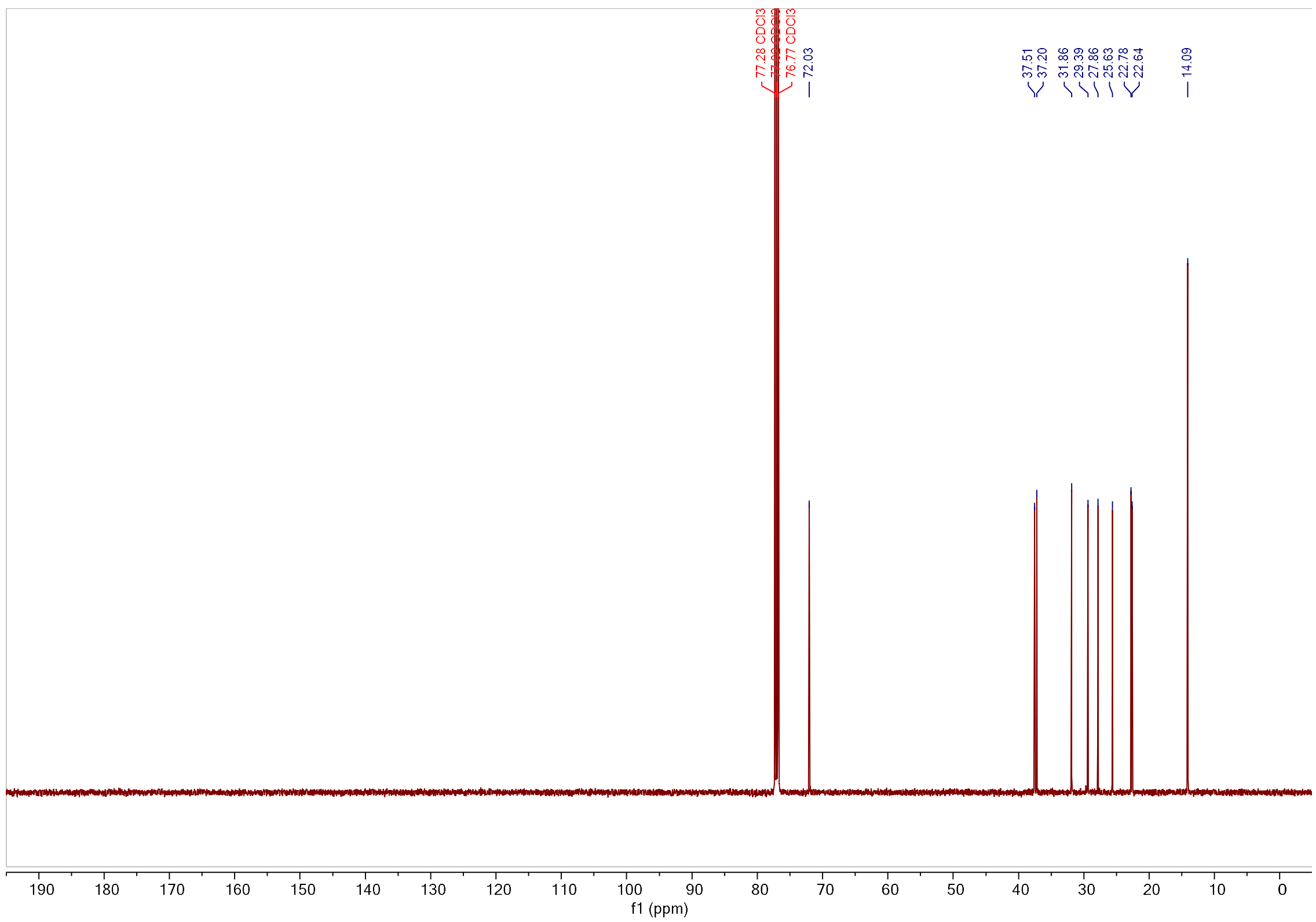

^{13}C-NMR spectrum of compound **1c** ($CDCl_3$, 126 MHz)

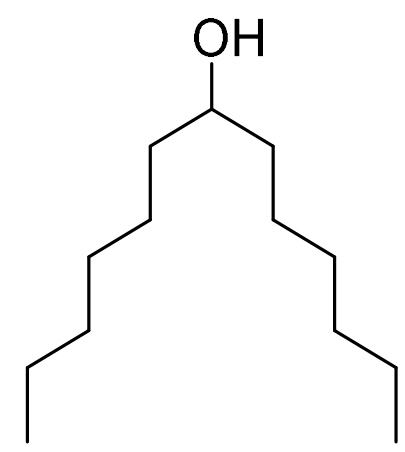

Figure S18. Chemical Structure of compound 1d.

^{1}H NMR (500 MHz, $CDCl_3$) δ 3.60 (s, 1H), 1.51 – 1.26 (m, 17H), 0.95 – 0.87 (m, 6H).

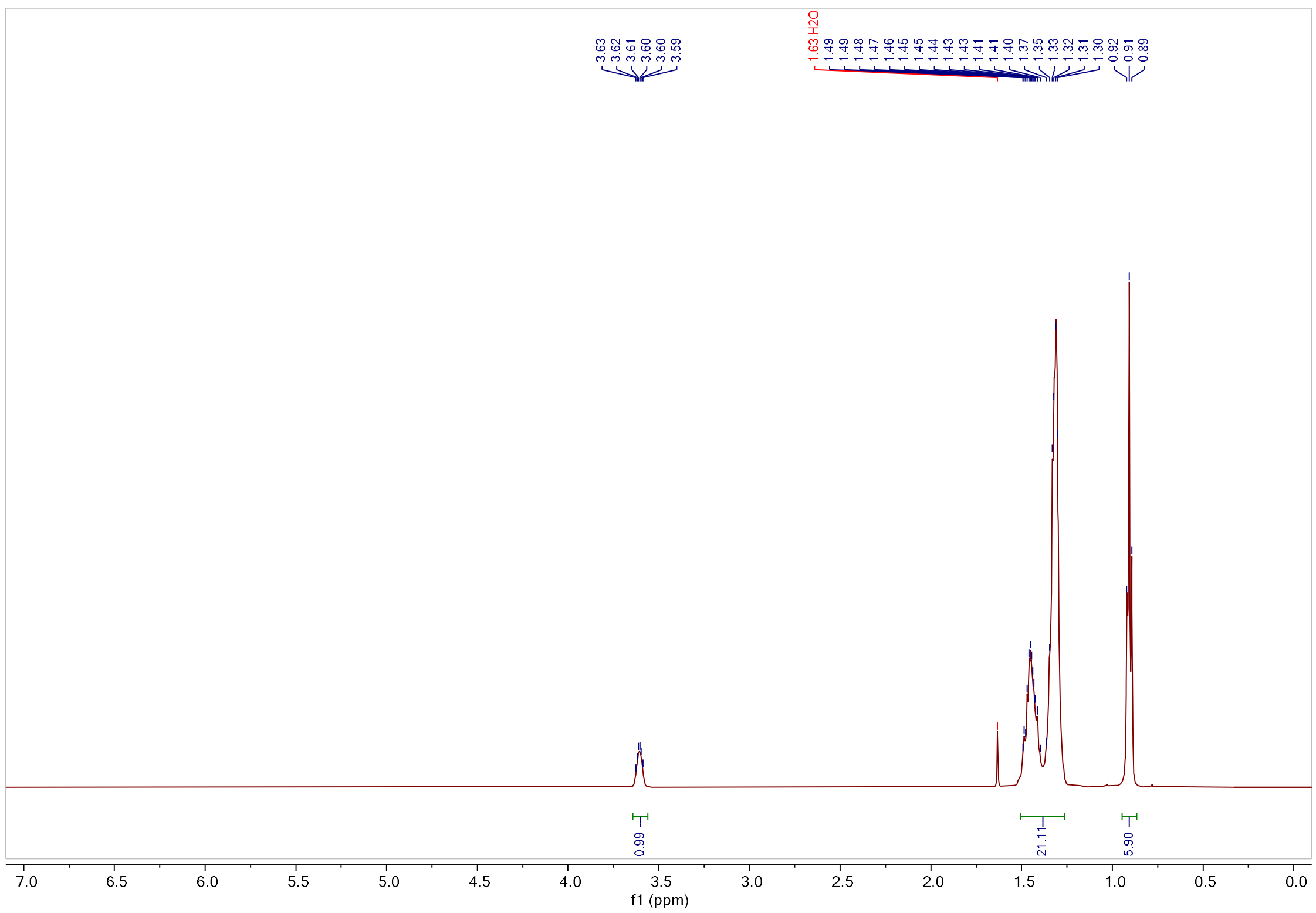

^{1}H NMR spectrum of compound **1d** ($CDCl_3$, 500 MHz)

^{13}C NMR (126 MHz, $CDCl_3$) δ 72.04, 37.51, 31.86, 29.39, 25.63, 22.64, 14.09.

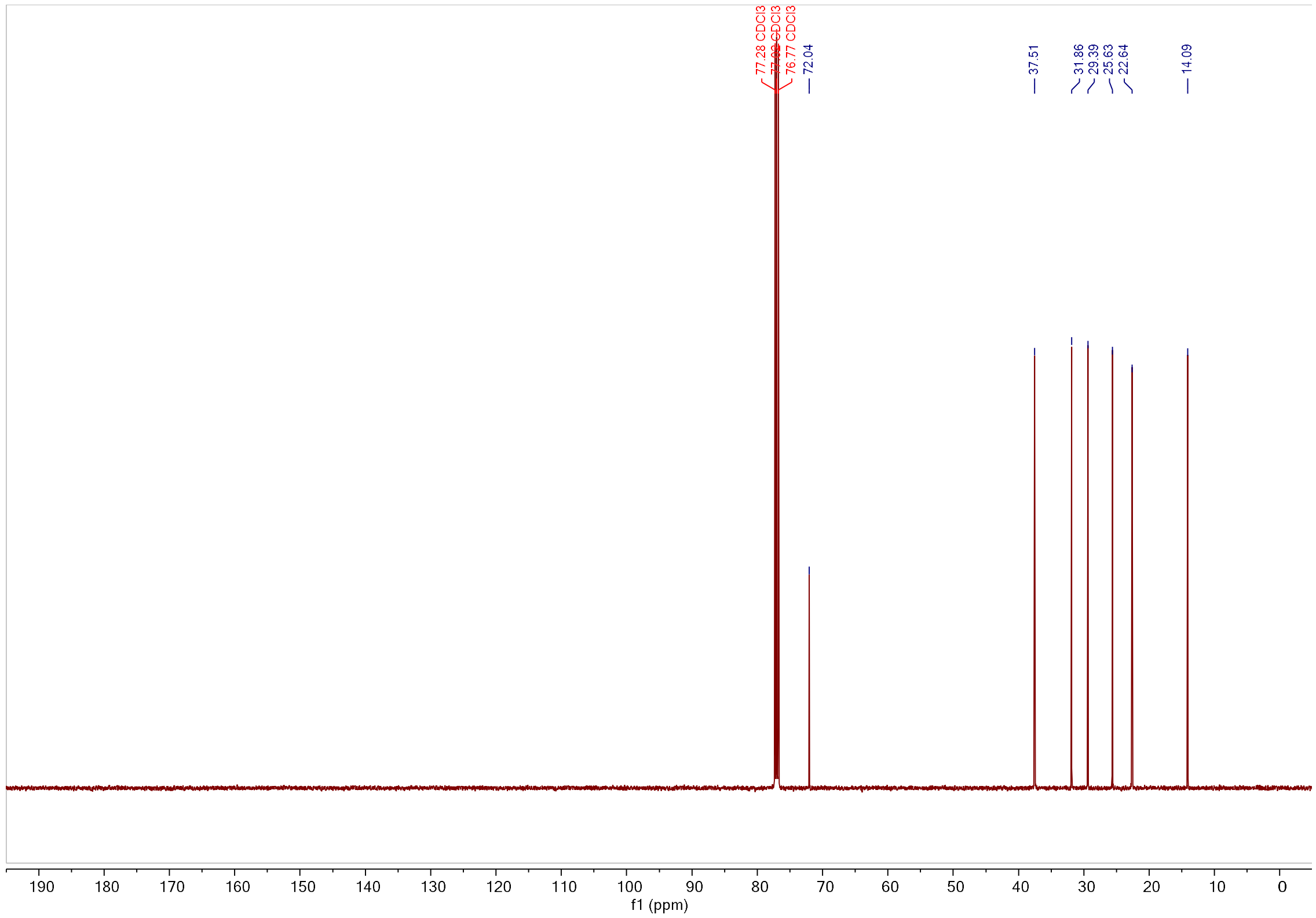

^{13}C-NMR spectrum of compound **1d** ($CDCl_3$, 126 MHz)

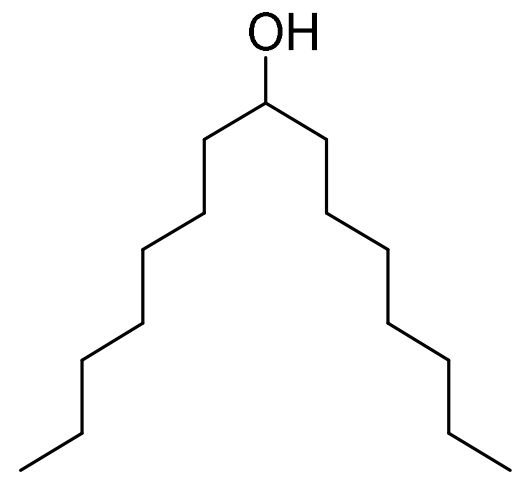

Figure S19. Chemical Structure of compound 1e.

^{1}H NMR (500 MHz, $CDCl_3$) δ 3.51 (s, 1H), 1.41 – 1.16 (m, 17H), 0.86 – 0.77 (m, 6H).

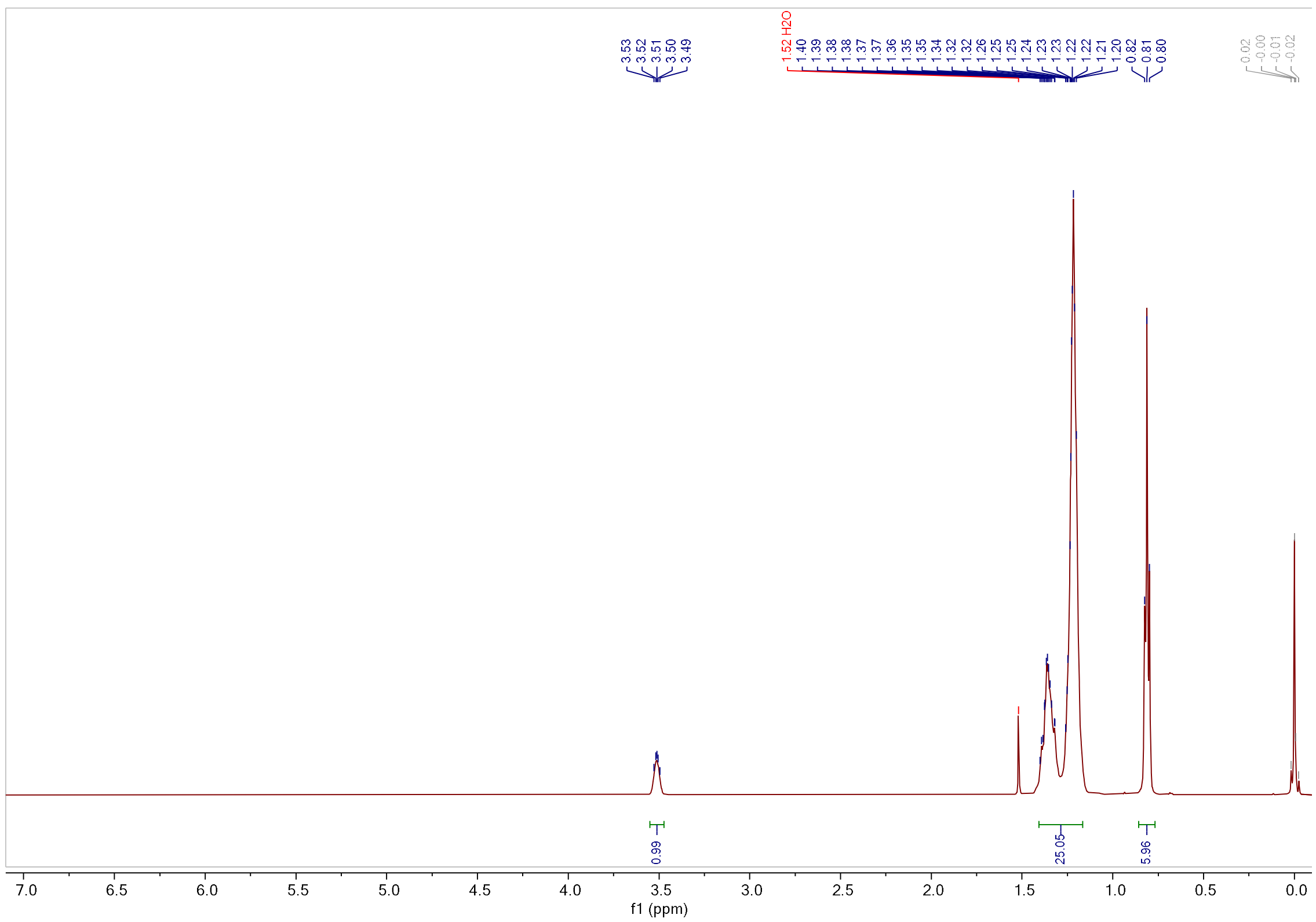

^{1}H NMR spectrum of compound **1e** ($CDCl_3$, 500 MHz)

^{13}C NMR (126 MHz, $CDCl_3$) δ 71.02, 36.48, 30.82, 28.66, 28.28, 24.64, 21.64, 13.08.

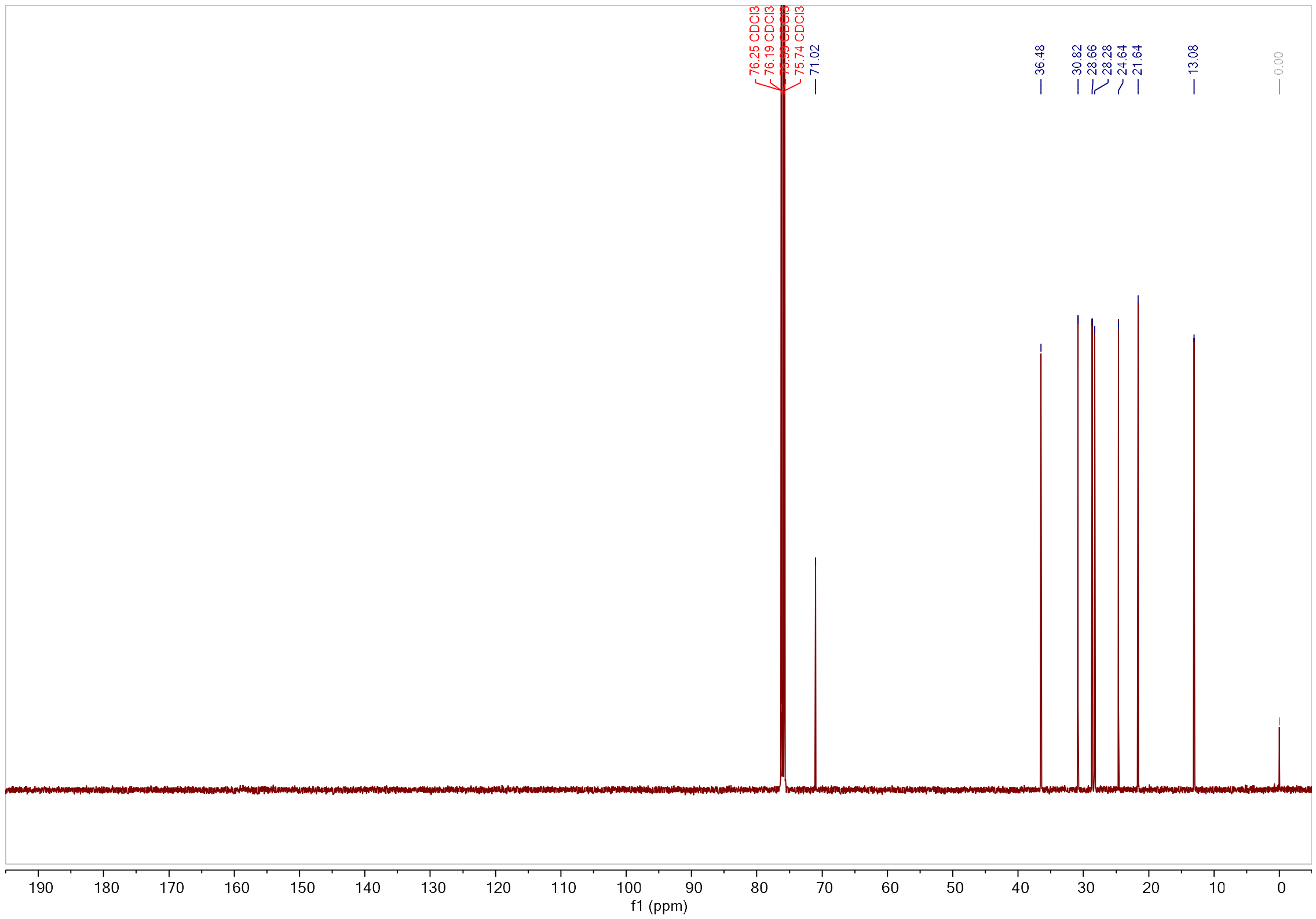

^{13}C-NMR spectrum of compound **1e** ($CDCl_3$, 126 MHz)

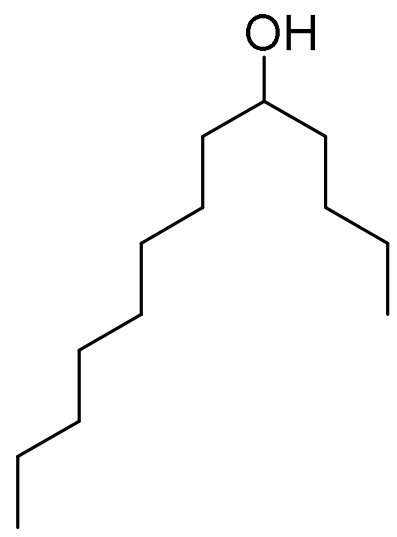

Figure S20. Chemical Structure of compound 1f.

^1H NMR (500 MHz, $CDCl_3$) δ 3.52 (s, 1H), 1.43 – 1.15 (m, 17H), 0.87 – 0.78 (m, 6H).

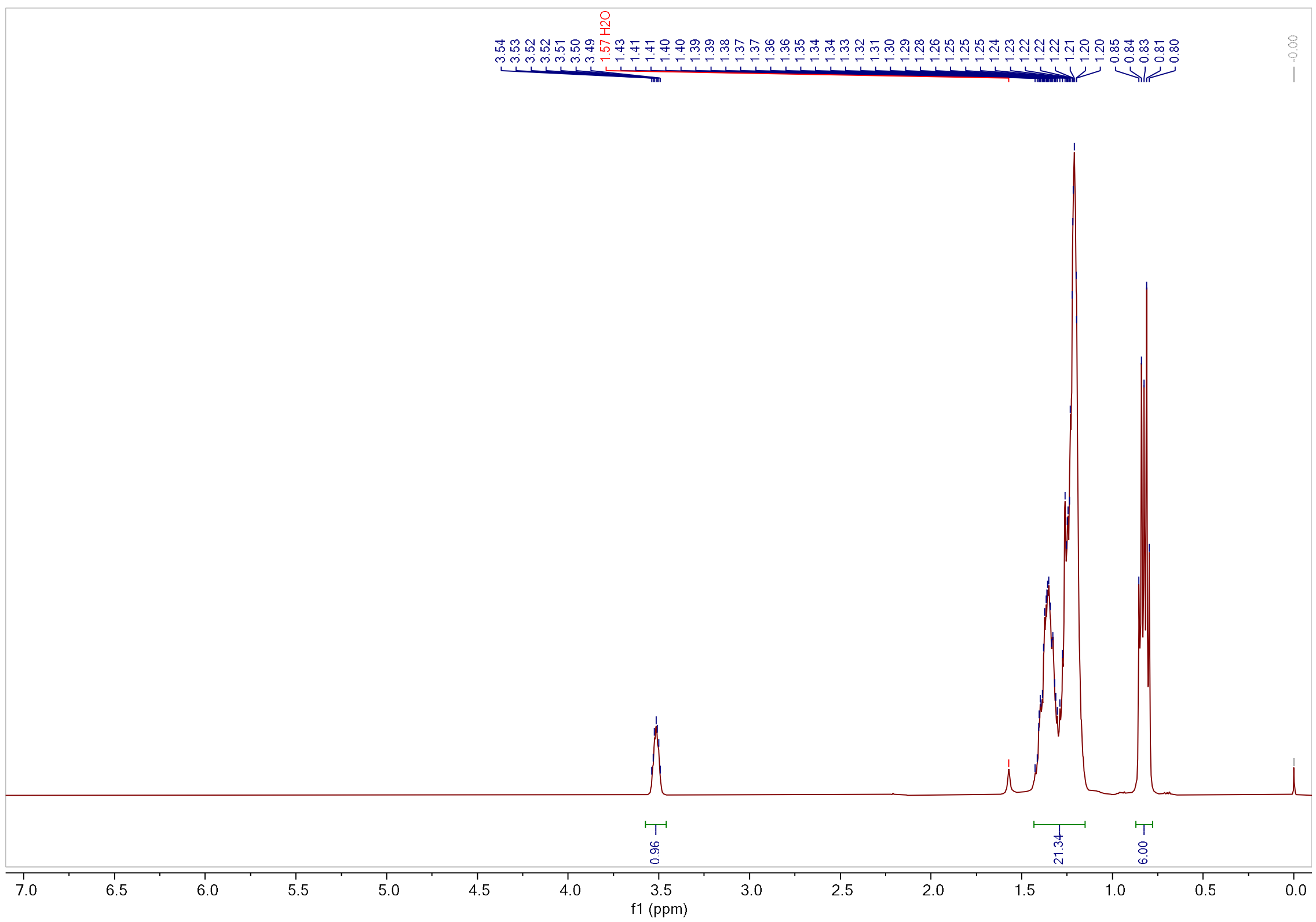

^1H NMR spectrum of compound **1f** ($CDCl_3$, 500 MHz)

^{13}C NMR (126 MHz, $CDCl_3$) δ 72.03, 39.31, 39.01, 37.51, 37.19, 31.90, 29.74, 29.61, 29.30, 27.86, 25.70, 25.67, 23.35, 22.78, 22.68, 14.11, 14.09.

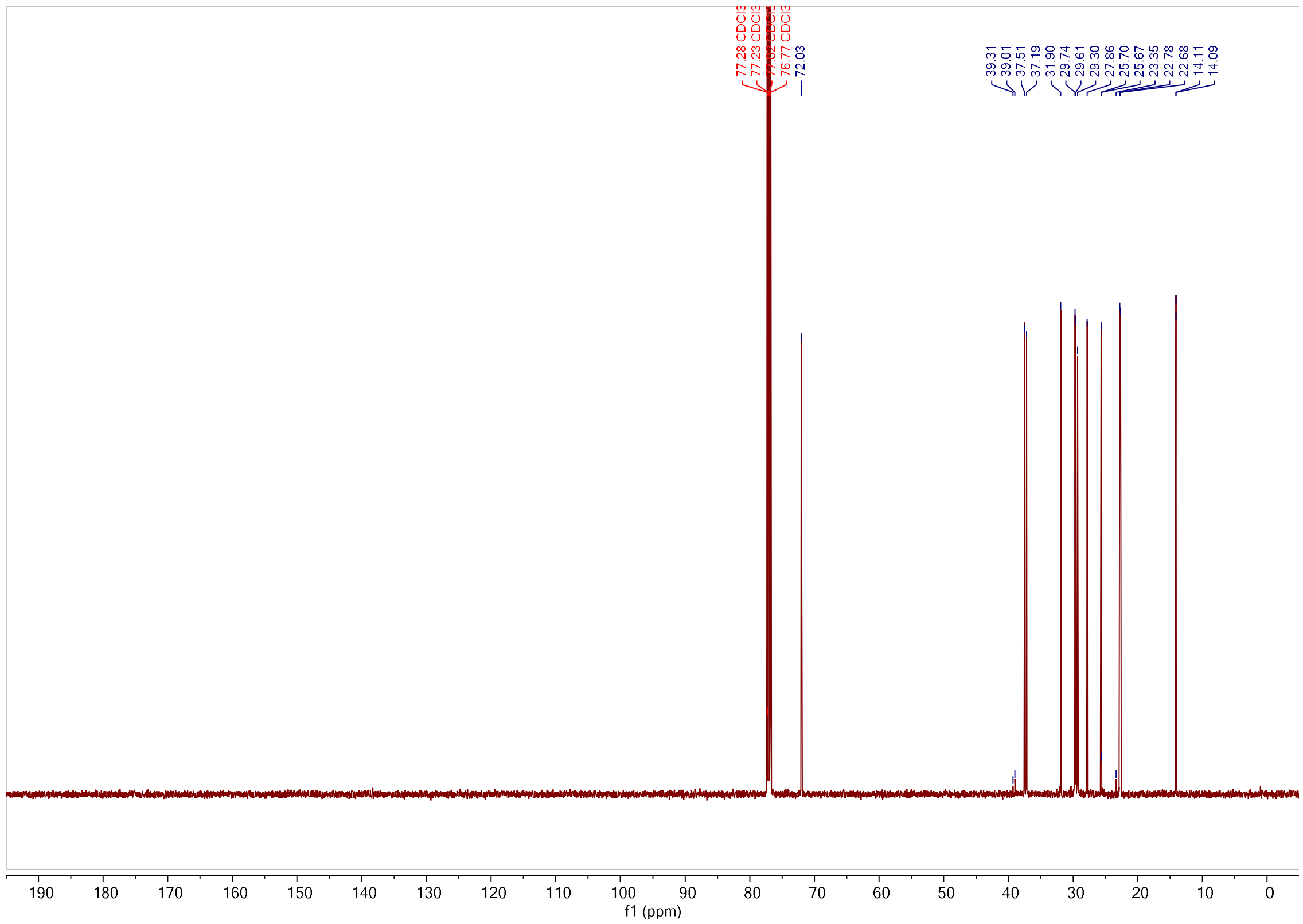

^{13}C-NMR spectrum of compound **1f** ($CDCl_3$, 126 MHz)

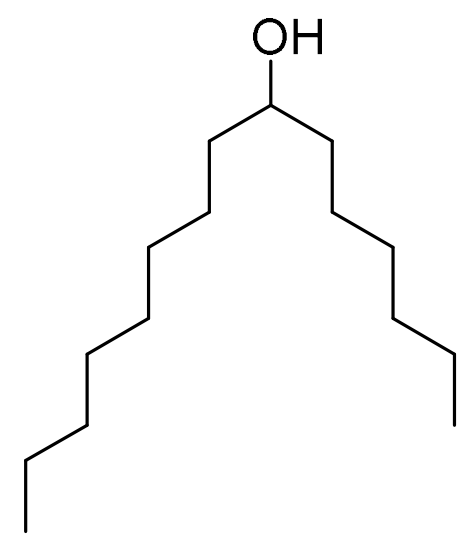

Figure S21. Chemical Structure of compound 1g.

^{1}H NMR (500 MHz, $CDCl_3$) δ 3.52 (s, 1H), 1.41 – 1.17 (m, 17H), 0.87 – 0.77 (m, 6H).

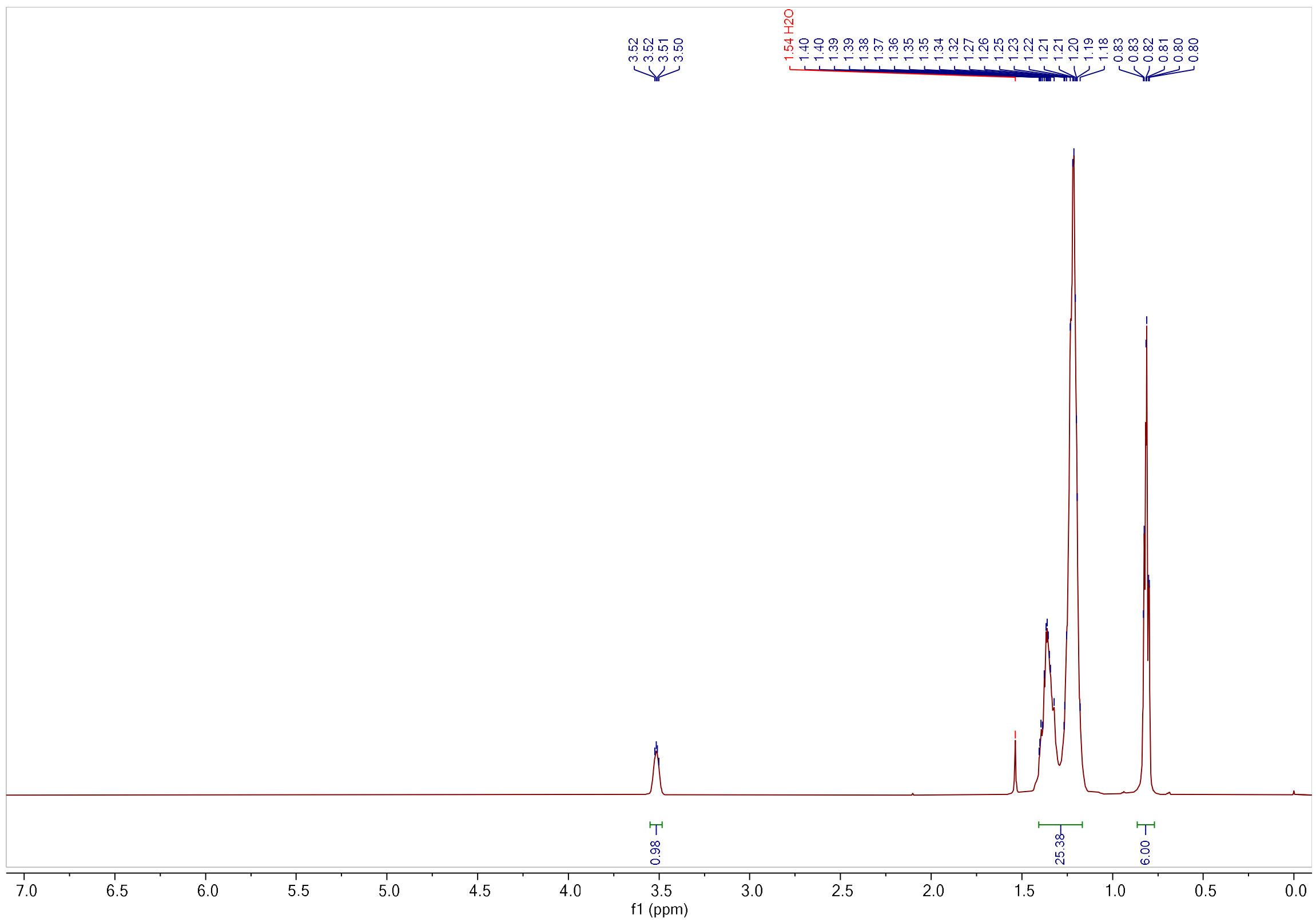

^{1}H NMR spectrum of compound **1g** ($CDCl_3$, 500 MHz)

^{13}C NMR (126 MHz, $CDCl_3$) δ 72.04, 37.51, 31.90, 31.86, 29.73, 29.61, 29.39, 29.29, 25.67, 25.63, 22.68, 22.64, 14.11, 14.09.

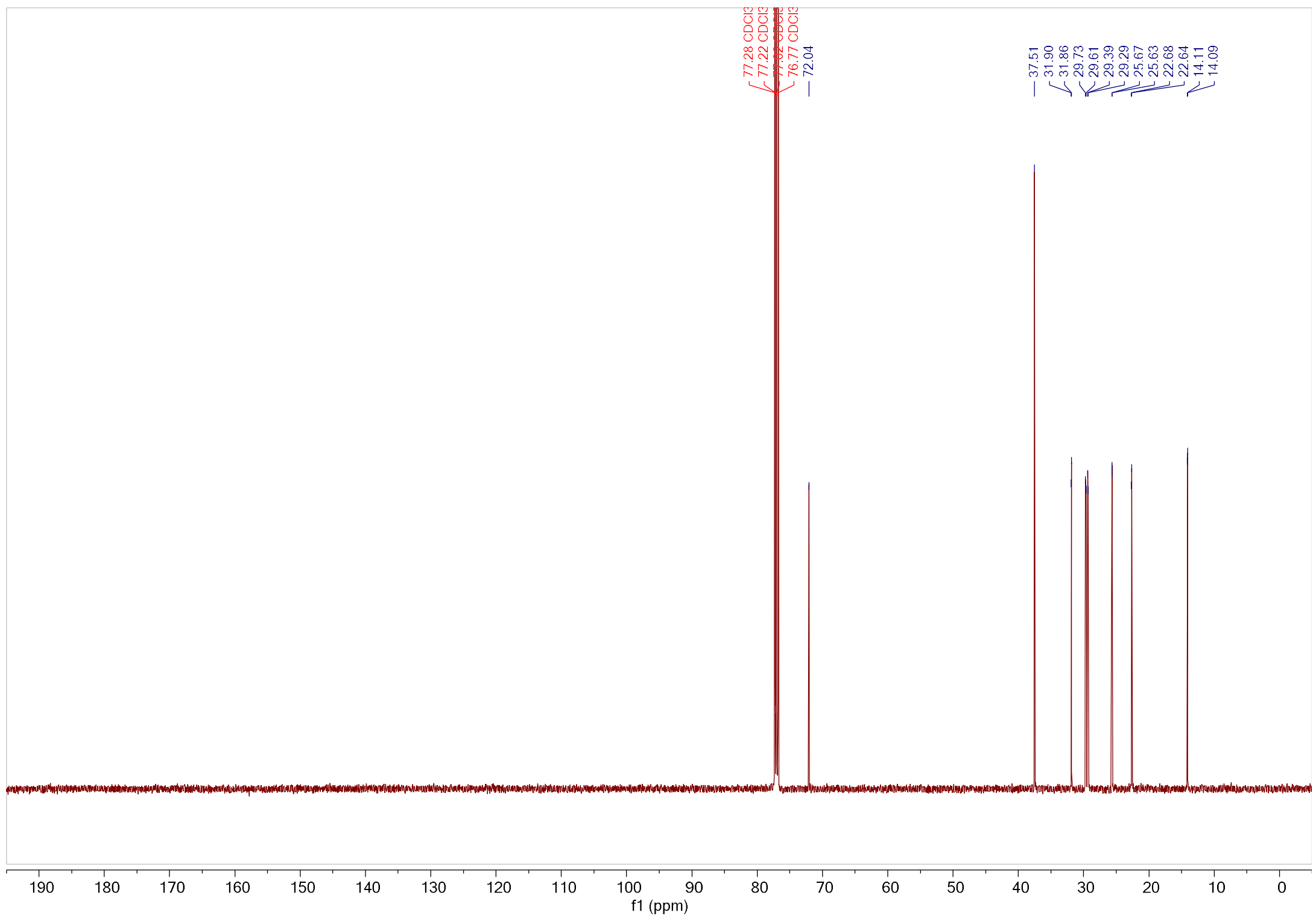

^{13}C-NMR spectrum of compound **1g** ($CDCl_3$, 126 MHz)

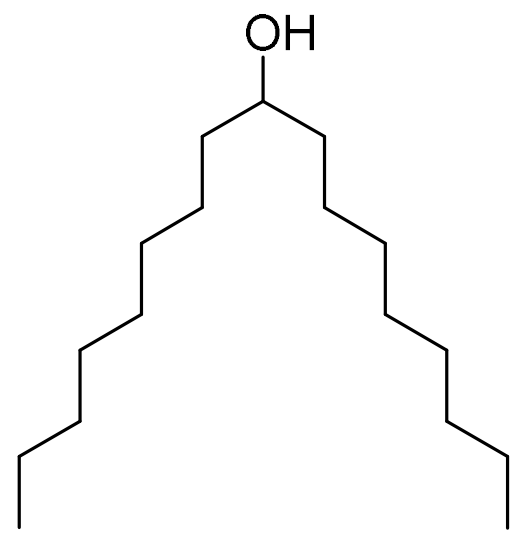

Figure S22. Chemical Structure of Compound 1h.

^{1}H NMR (400 MHz, $CDCl_3$) δ 3.51 (s, 1H), 1.40 – 1.16 (m, 29H), 0.85 – 0.77 (m, 6H).

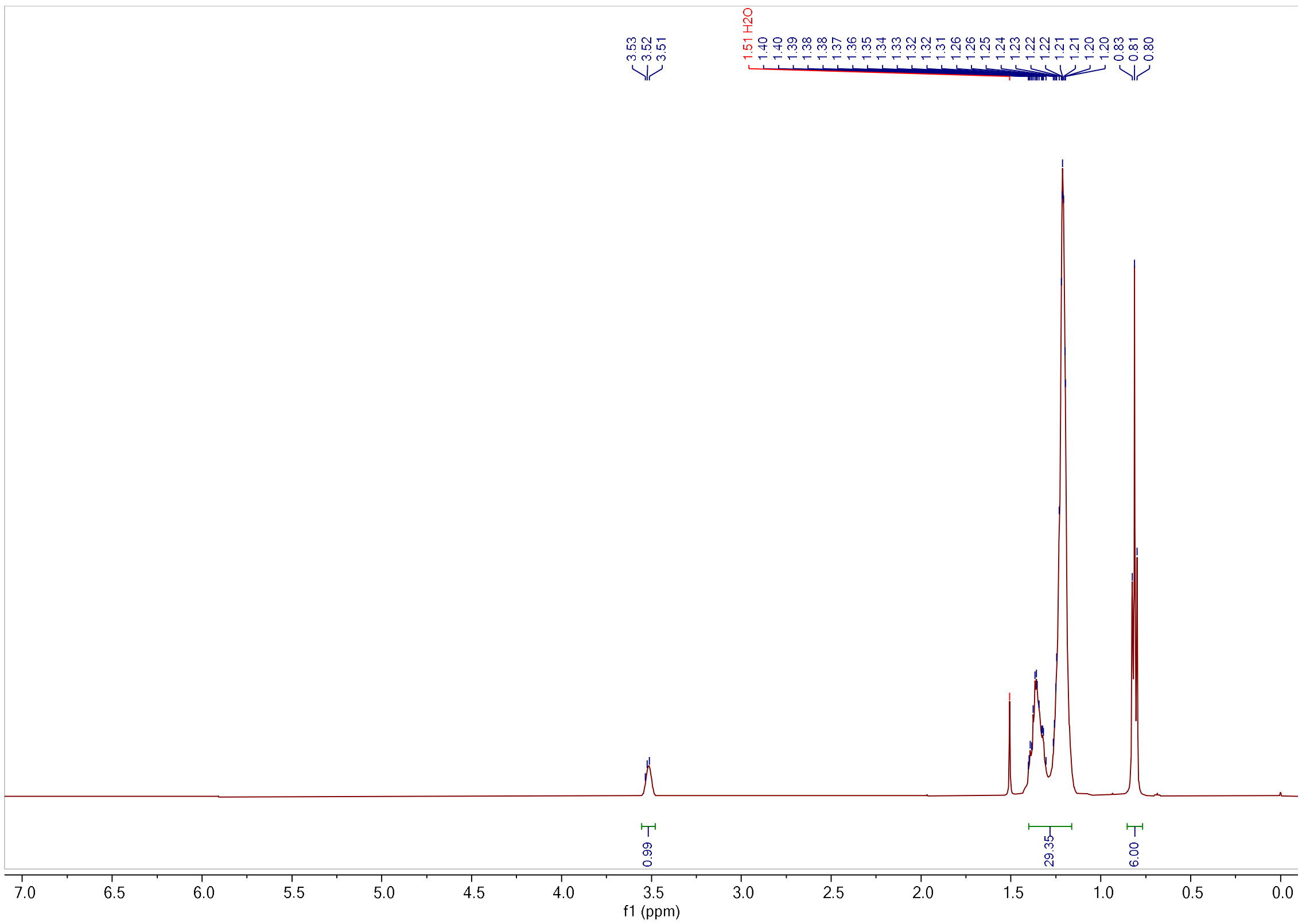

^{1}H NMR spectrum of compound **1h** ($CDCl_3$, 400 MHz)

^{13}C NMR (126 MHz, $CDCl_3$) δ 72.05, 37.51, 31.90, 29.73, 29.61, 29.30, 25.67, 22.68, 14.12.

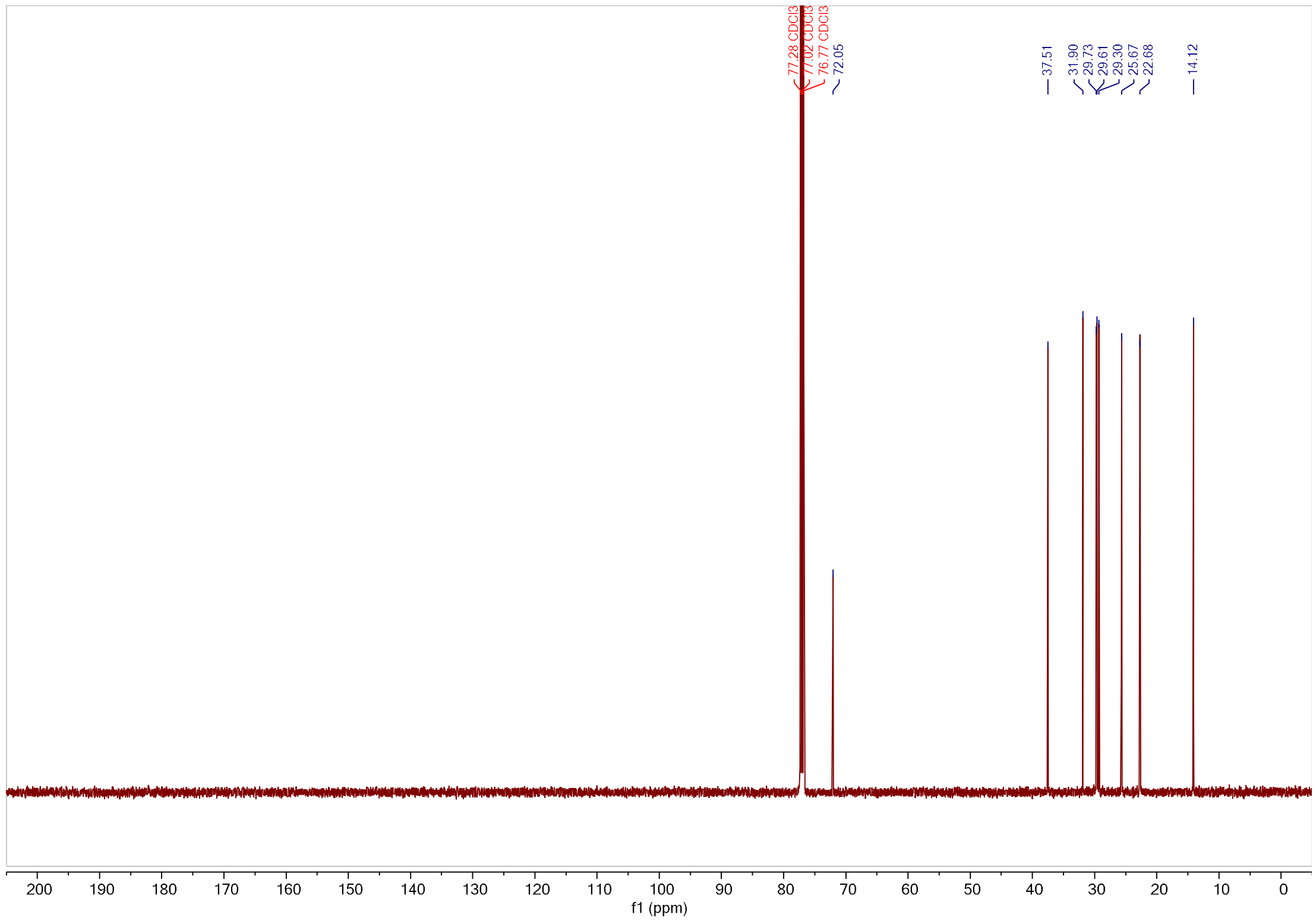

^{13}C-NMR spectrum of compound **1h** ($CDCl_3$, 126 MHz)

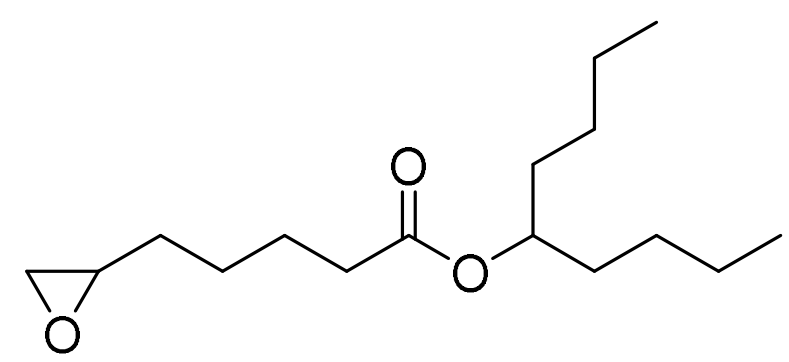

Figure S23. Chemical structure of compound 4a.

1H NMR (500 MHz, $CDCl_3$) δ 4.81 (p, J = 6.3 Hz, 1H), 2.88 – 2.80 (m, 1H), 2.71 – 2.65 (m, 1H), 2.40 (dd, J = 5.0, 2.7 Hz, 1H), 2.24 (t, J = 7.4 Hz, 2H), 1.69 – 1.56 (m, 2H), 1.52 – 1.36 (m, 8H), 1.30 – 1.14 (m, 8H), 0.85 – 0.74 (m, 6H).

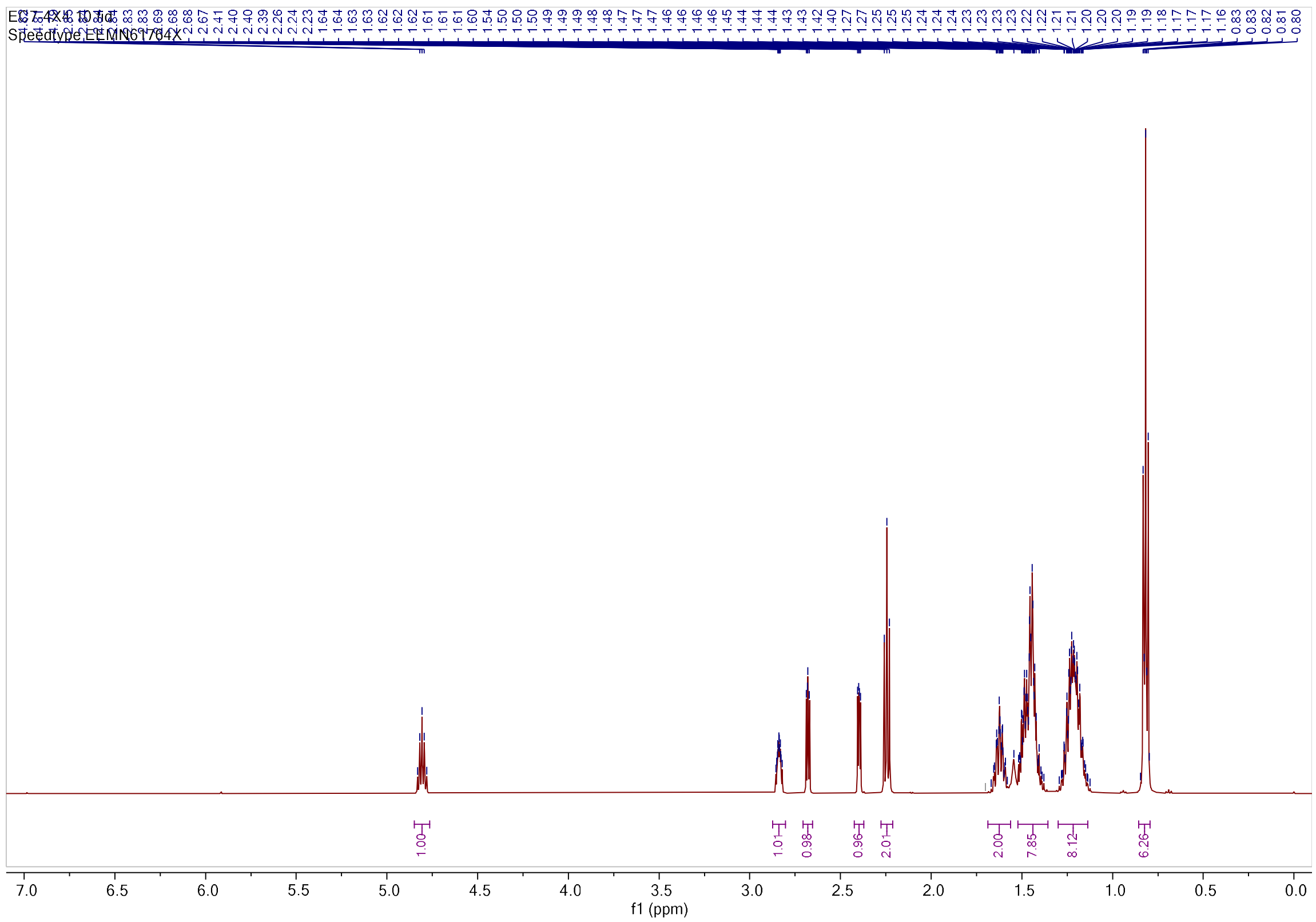

^{1}H NMR spectrum of compound **4a** ($CDCl_3$, 500 MHz)

^{13}C NMR (101 MHz, $CDCl_3$) δ 173.46, 74.32, 52.19, 47.16, 34.59, 33.93, 32.25, 27.59, 25.62, 24.96, 22.68, 14.10.

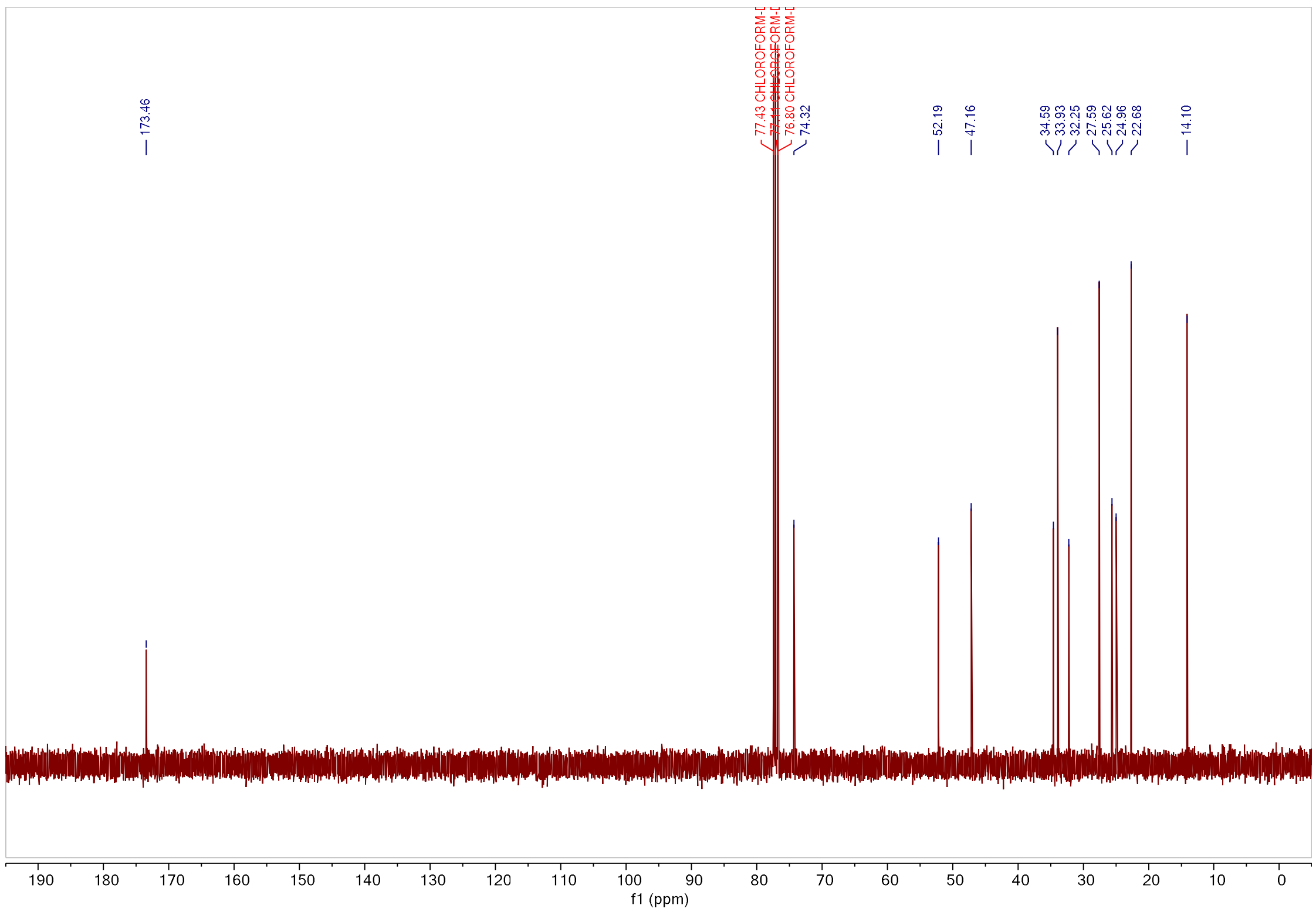

^{13}C-NMR spectrum of compound **4a** ($CDCl_3$, 101 MHz)

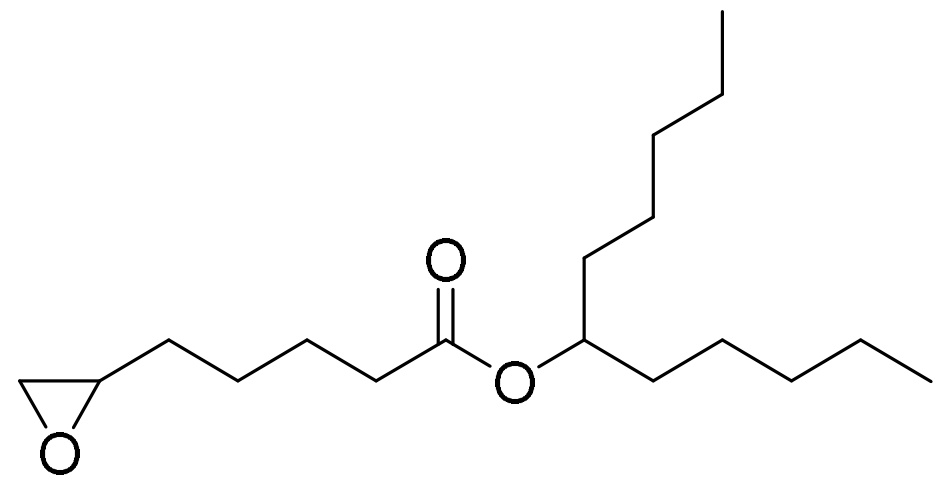

Figure S24. Chemical structure of compound 4b.

1H NMR (500 MHz, $CDCl_3$) δ 4.81 (p, J = 6.2 Hz, 1H), 2.87 – 2.80 (m, 1H), 2.71 – 2.65 (m, 1H), 2.40 (dd, J = 5.0, 2.7 Hz, 1H), 2.24 (t, J = 7.4 Hz, 2H), 1.68 – 1.57 (m, 2H), 1.50 – 1.38 (m, 8H), 1.27 – 1.13 (m, 12H), 0.84 – 0.77 (m, 6H).

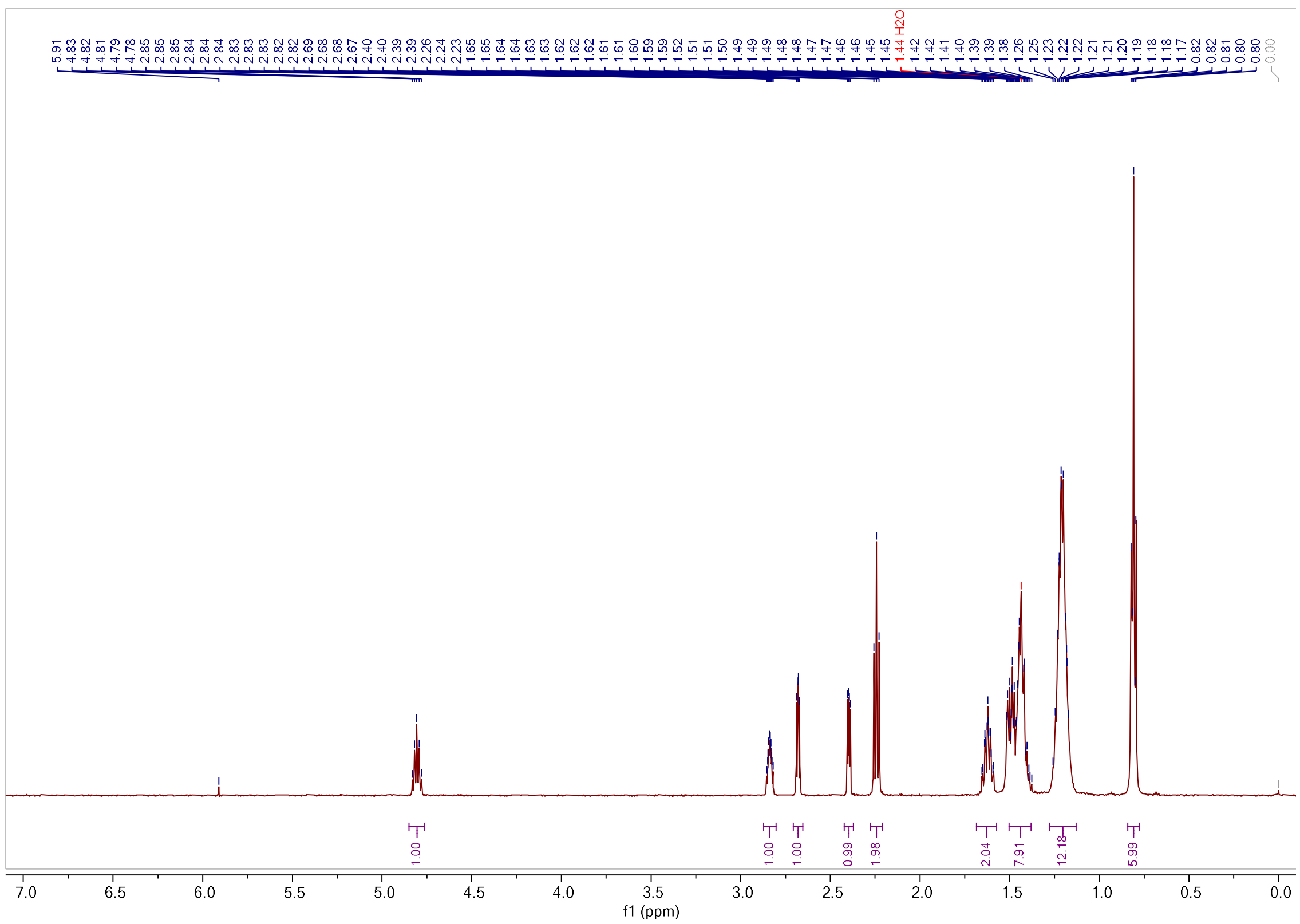

^{1}H NMR spectrum of compound **4b** ($CDCl_3$, 500 MHz)

^{13}C NMR (101 MHz, $CDCl_3$) δ 173.4, 74.34, 52.14, 47.12, 34.60, 34.17, 32.24, 31.78, 25.61, 25.07, 24.97, 22.62, 14.07.

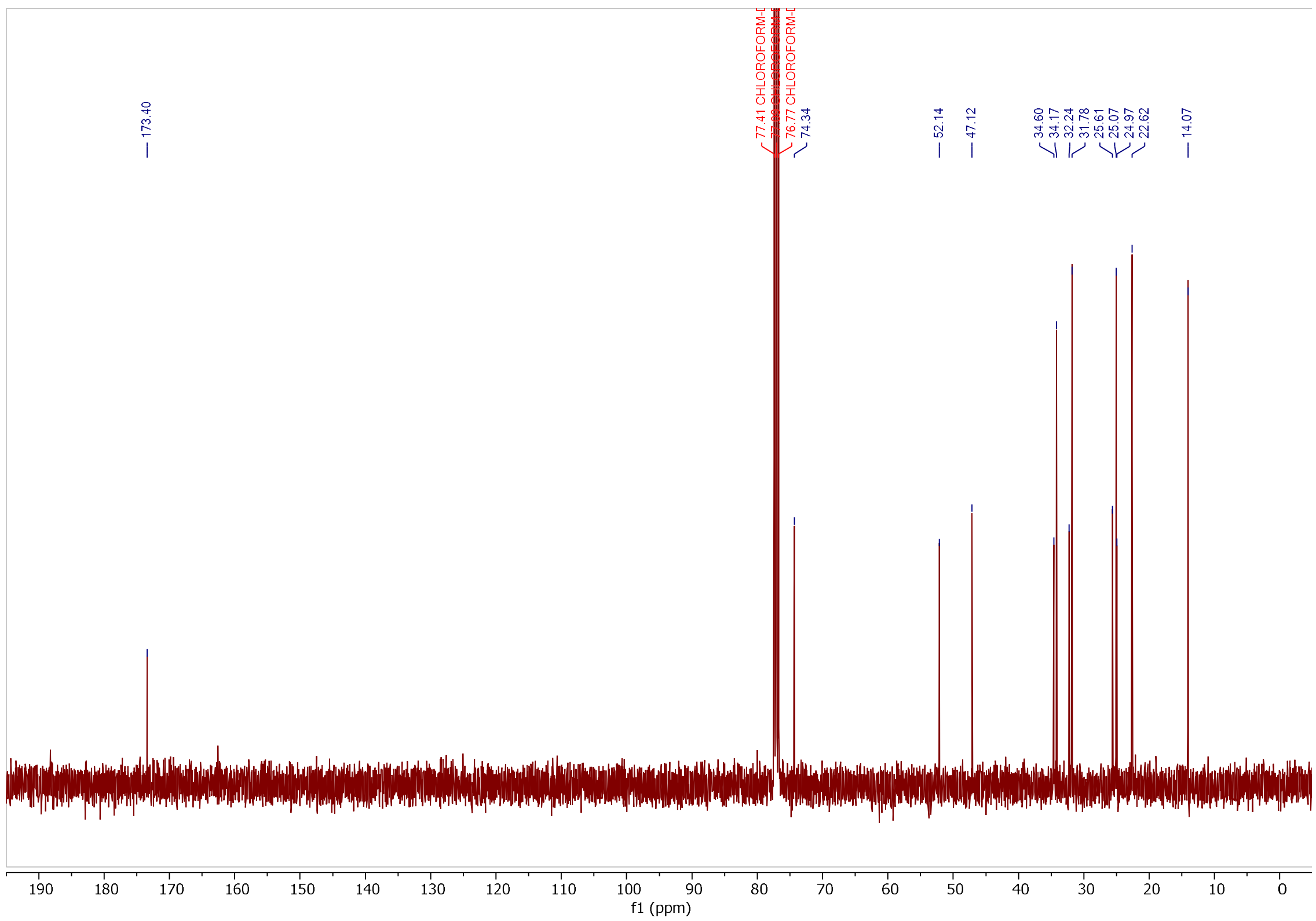

^{13}C-NMR spectrum of compound **4b** ($CDCl_3$, 101 MHz)

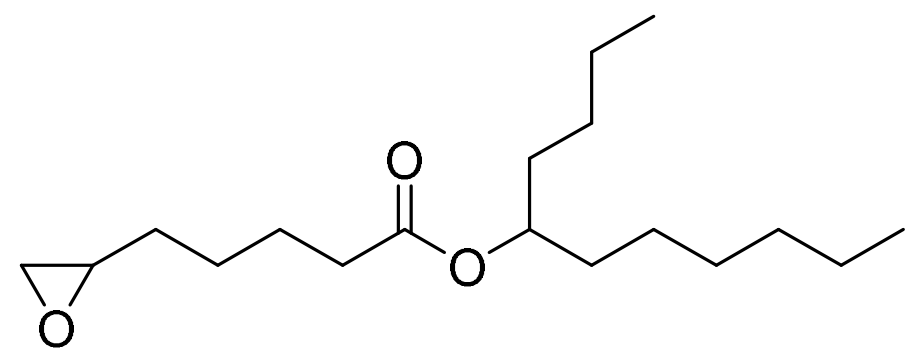

Figure S25. Chemical structure of compound 4c.

1H NMR (500 MHz, $CDCl_3$) δ 4.80 (p, J = 6.3 Hz, 1H), 2.87-2.80 (m, 1H), 2.70 – 2.65 (m, 1H), 2.40 (dd, J = 5.0, 2.7 Hz, 1H), 2.24 (t, J = 7.4 Hz, 2H), 1.69 – 1.58 (m, 2H), 1.52 – 1.39 (m, 8H), 1.28-1.14 (m,12H), 0.86 – 0.78 (m, 6H).

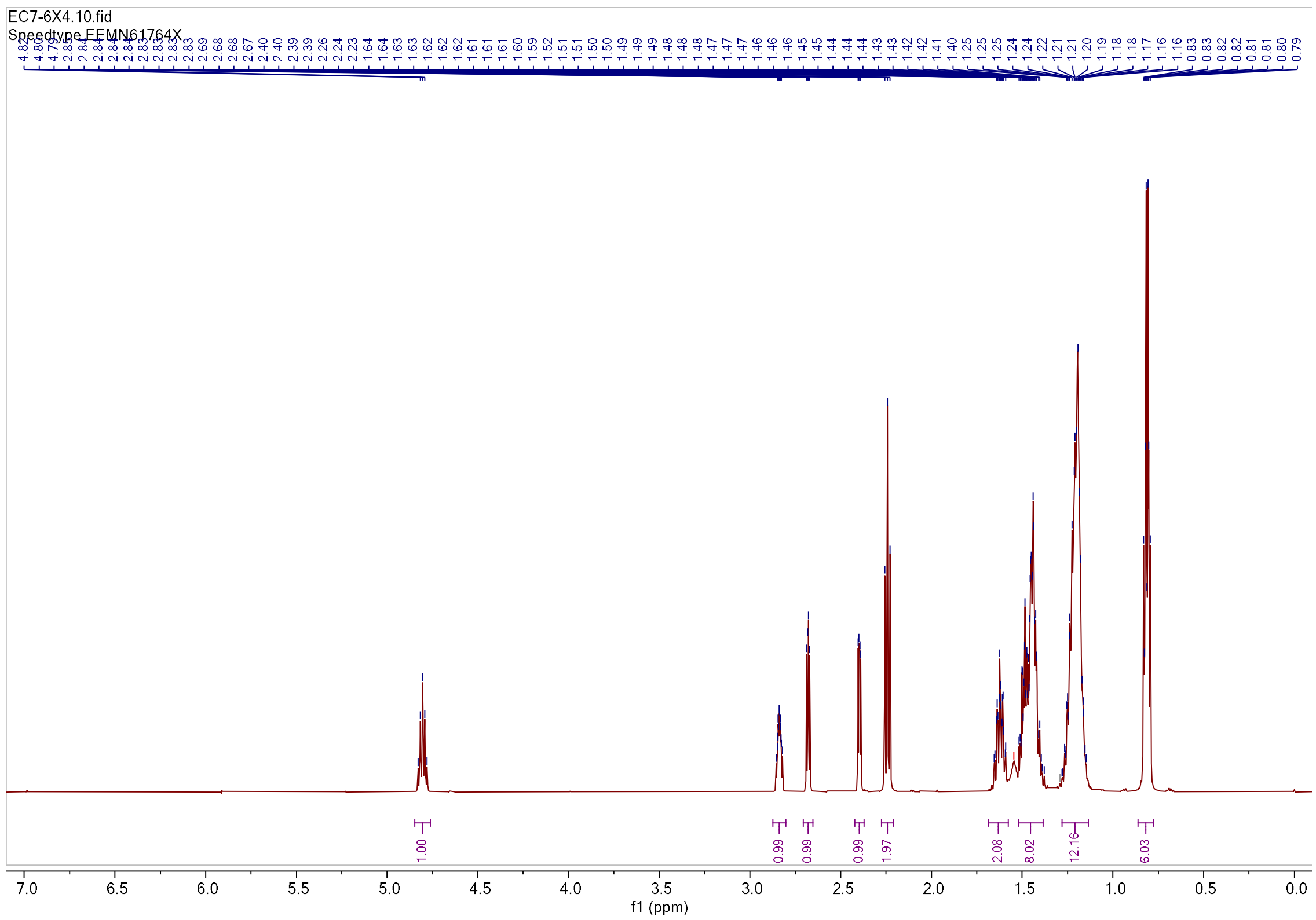

^{1}H NMR spectrum of compound **4c** ($CDCl_3$, 500 MHz)

^{13}C NMR (101 MHz, $CDCl_3$) δ 173.42, 74.34, 52.15, 47.12, 34.59, 34.23, 33.91, 32.24, 31.83, 29.27, 27.58, 25.61, 25.37, 24.96, 22.67, 22.65, 14.13, 14.07.

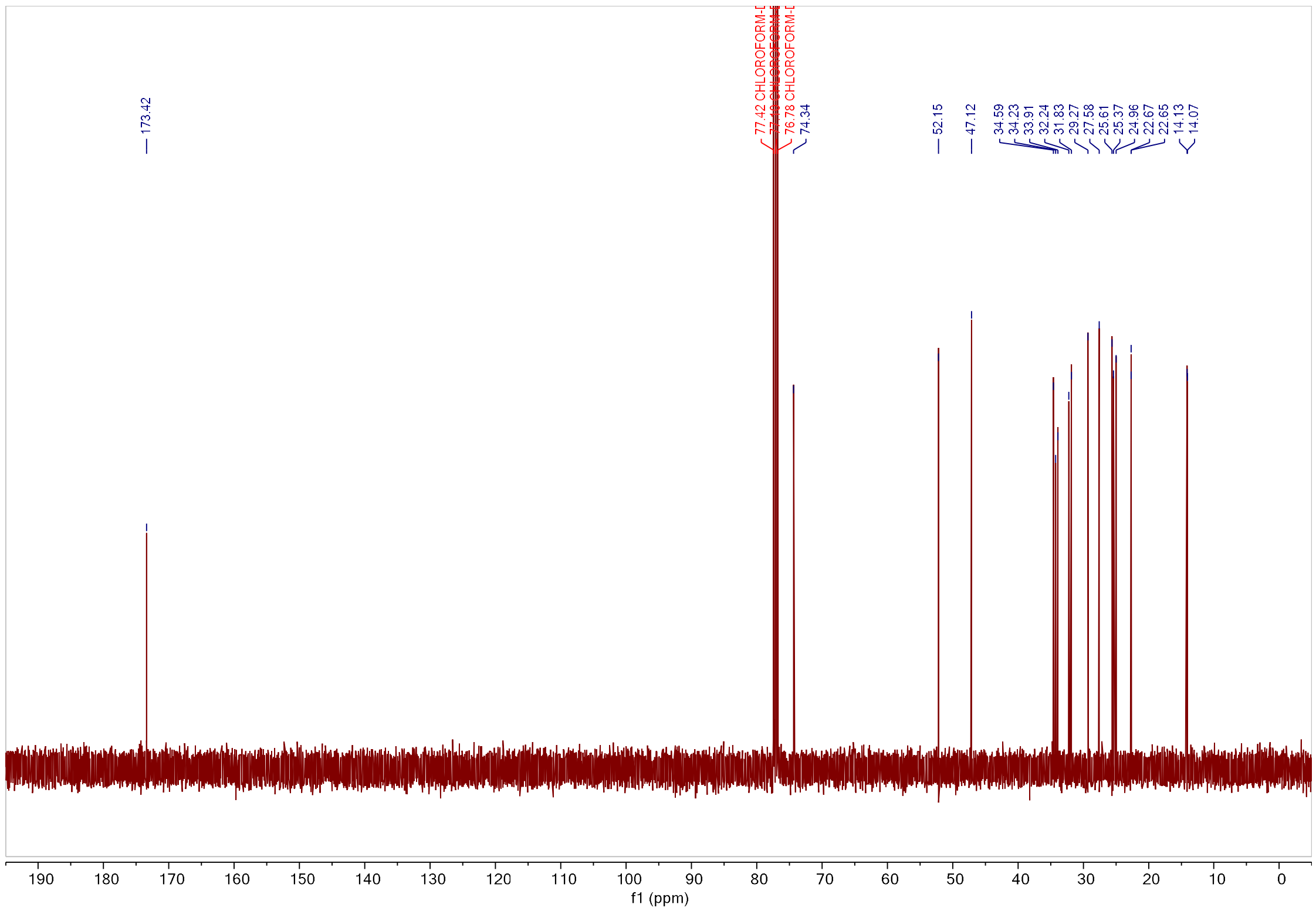

^{13}C-NMR spectrum of compound **4c** ($CDCl_3$, 101 MHz)

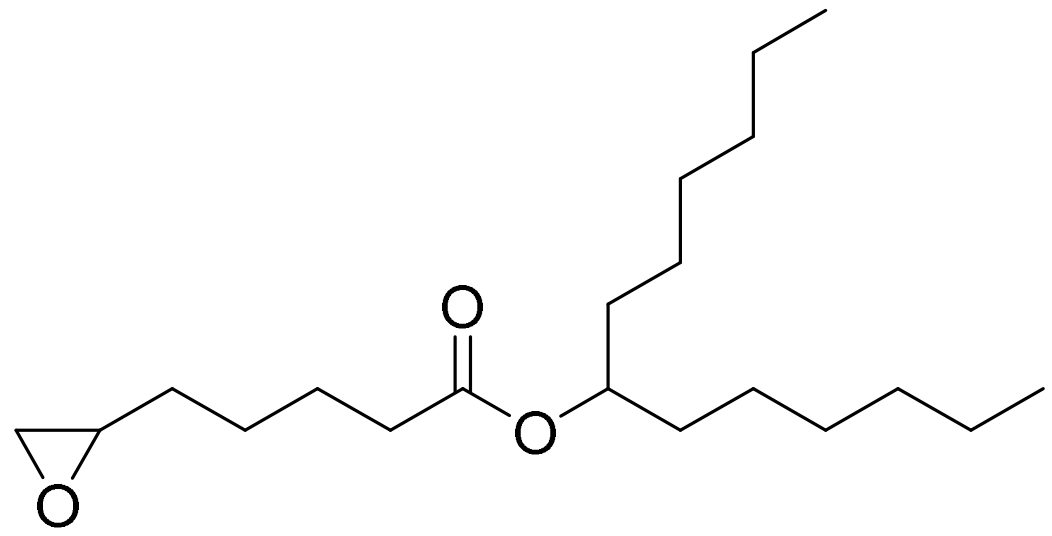

Figure S26. Chemical structure of compound 4d.

1H NMR (500 MHz, $CDCl_3$) δ 4.80 (p, J = 6.2 Hz, 1H), 2.87-2.80 (m, 1H), 2.70 – 2.66 (m, 1H), 2.40 (dd, J = 5.0, 2.7 Hz, 1H), 2.24 (t, J = 7.4 Hz, 2H), 1.66 – 1.58 (m, 2H), 1.52 – 1.37 (m, 8H), 1.26 – 1.14 (m, 16H), 0.84 – 0.77 (m, 6H).

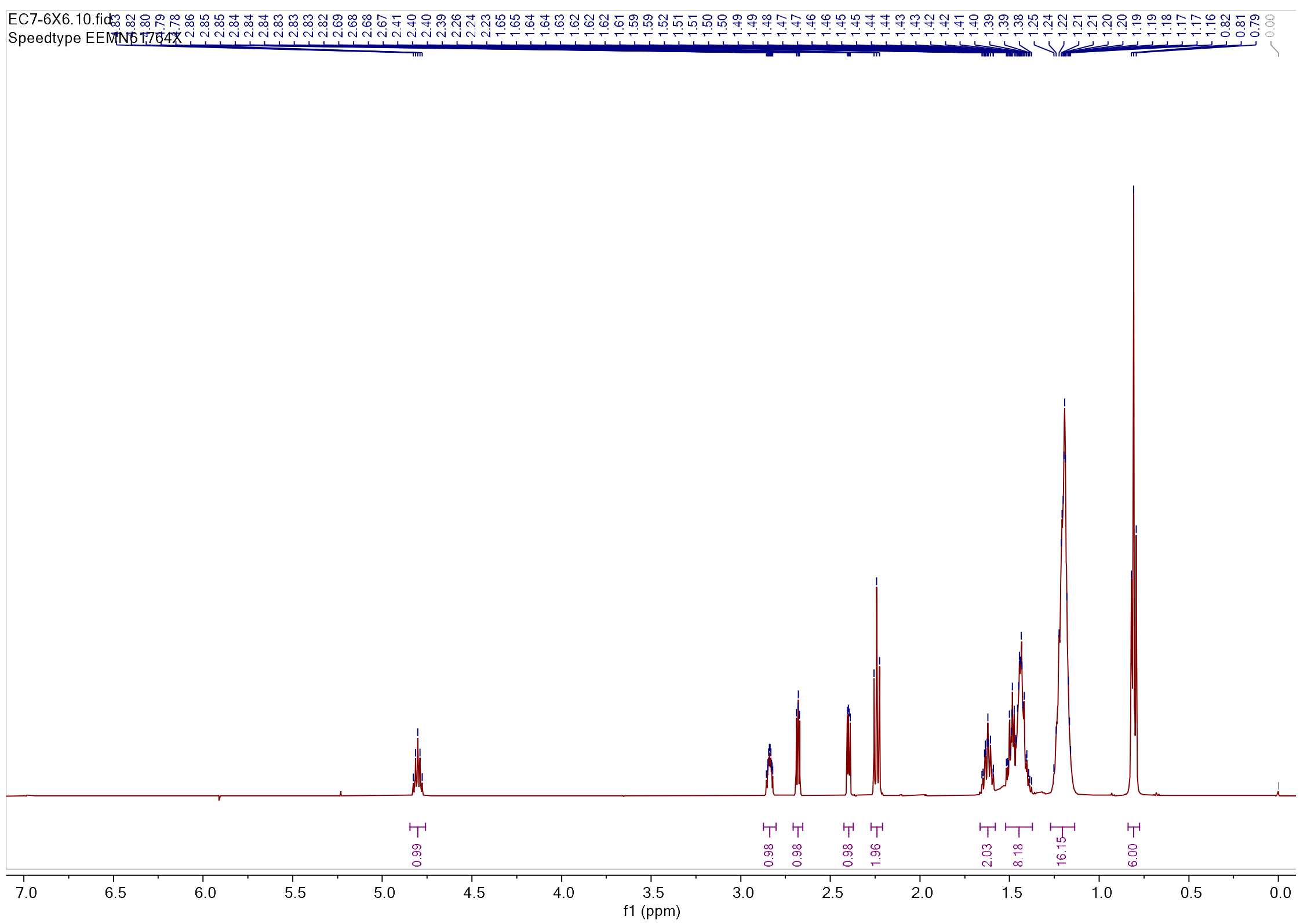

^{1}H NMR spectrum of compound **4d** ($CDCl_3$, 500 MHz)

^{13}C NMR (101 MHz, $CDCl_3$) δ 173.43, 74.36, 52.18, 47.15, 34.60, 34.22, 32.25, 31.84, 29.28, 25.62, 25.37, 24.97, 22.67, 14.16.

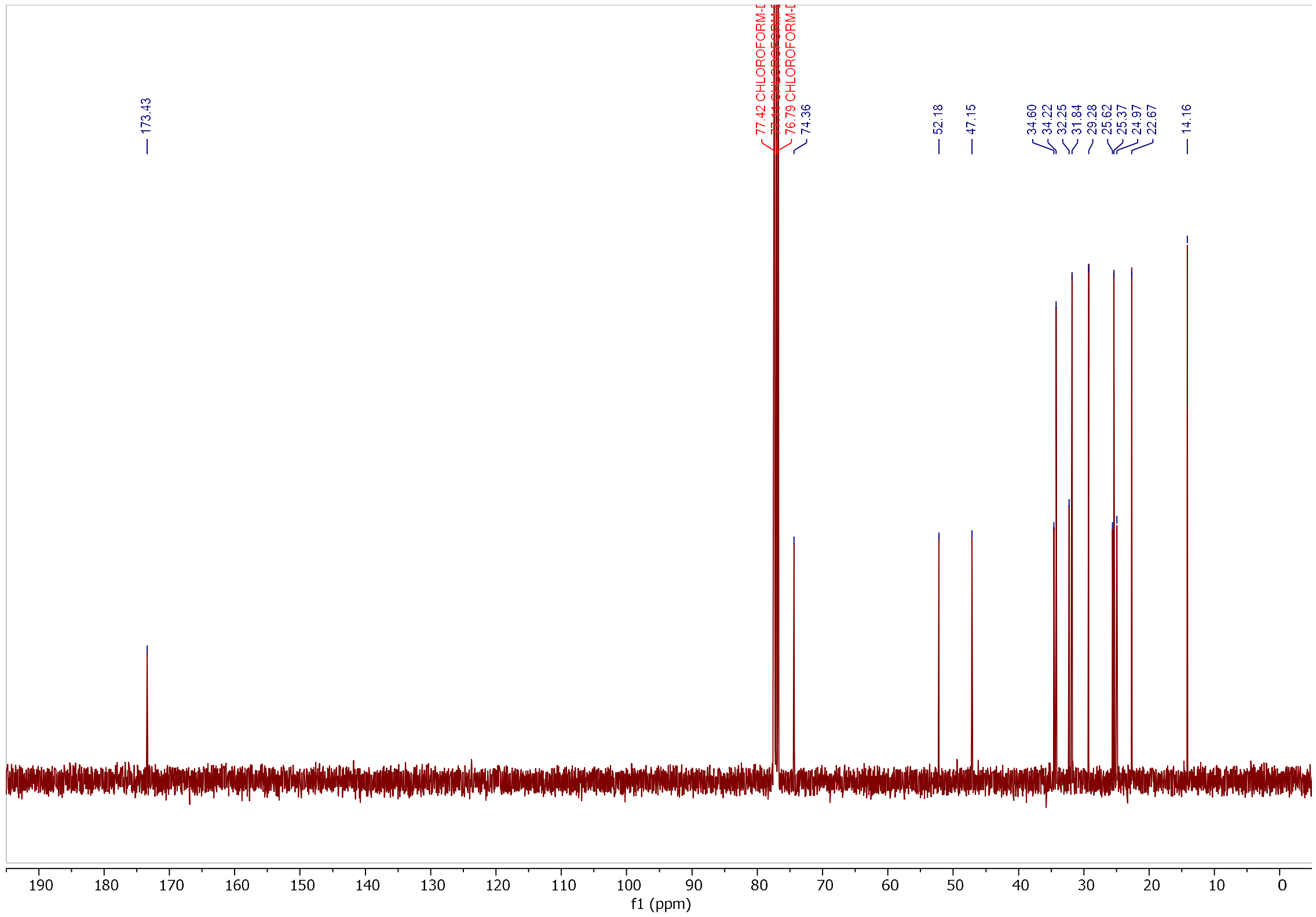

^{13}C-NMR spectrum of compound **4d** ($CDCl_3$, 101 MHz)

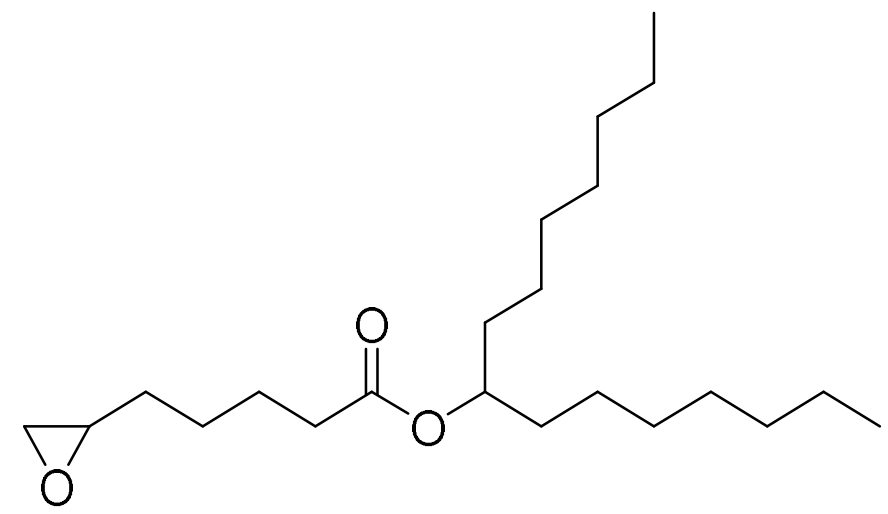

Figure S27. Chemical structure of compound 4e.

1H NMR (500 MHz, $CDCl_3$) δ 4.80 (p, J = 6.3 Hz, 1H), 2.87-2.80 (m, 1H), 2.70 – 2.65 (m, 1H), 2.40 (dd, J = 5.0, 2.7 Hz, 1H), 2.24 (t, J = 7.5 Hz, 2H), 1.66 – 1.58 (m, 2H), 1.53 – 1.38 (m, 8H), 1.27 – 1.15 (m, 20H), 0.81 (t, J = 6.9 Hz, 6H).

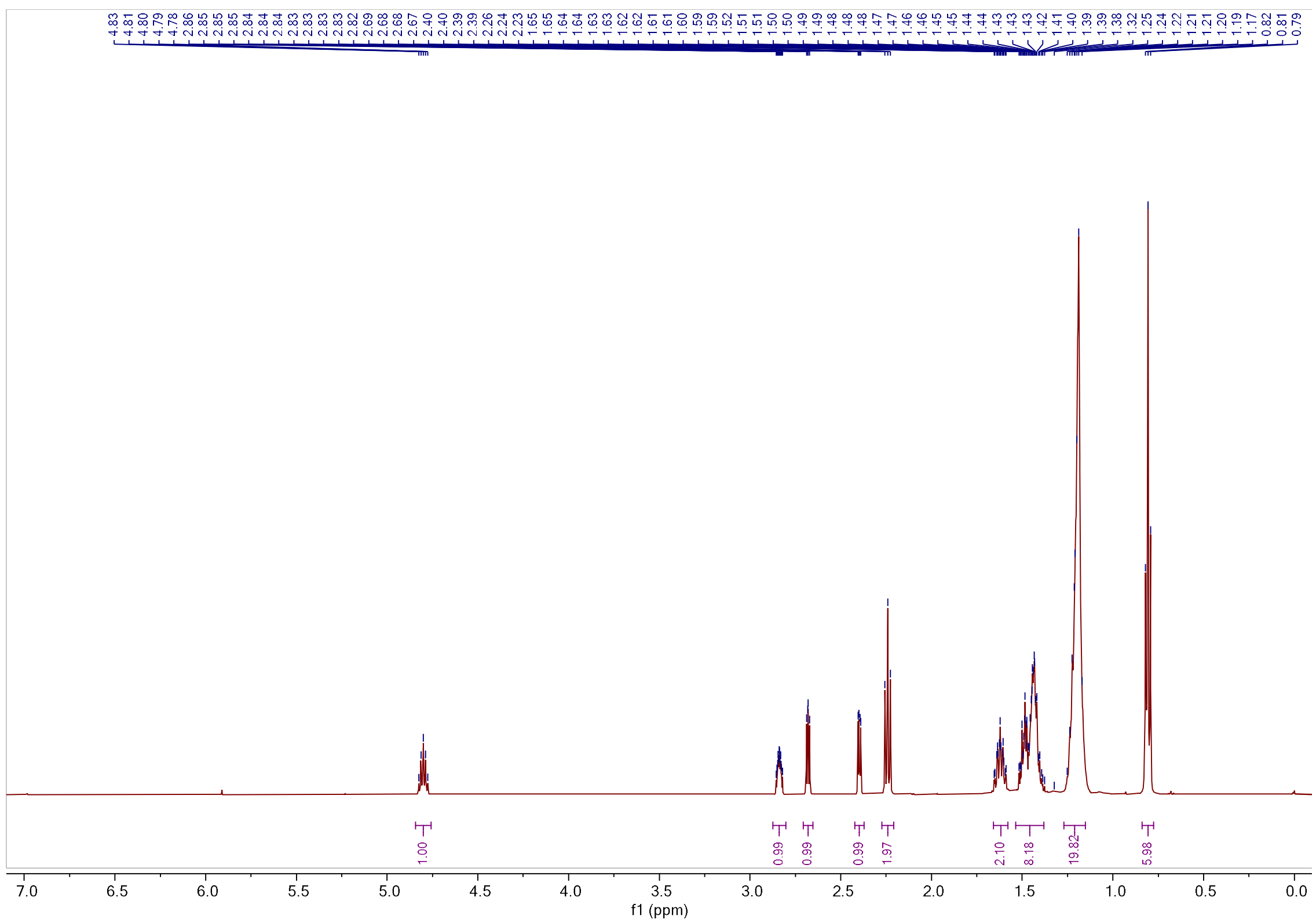

^{1}H NMR spectrum of compound **4e** ($CDCl_3$, 500 MHz)

^{13}C NMR (101 MHz, $CDCl_3$) δ 173.44, 74.37, 47.16, 34.61, 34.22, 32.25, 31.87, 29.58, 29.31, 25.62, 25.42, 24.97, 22.74, 14.19.

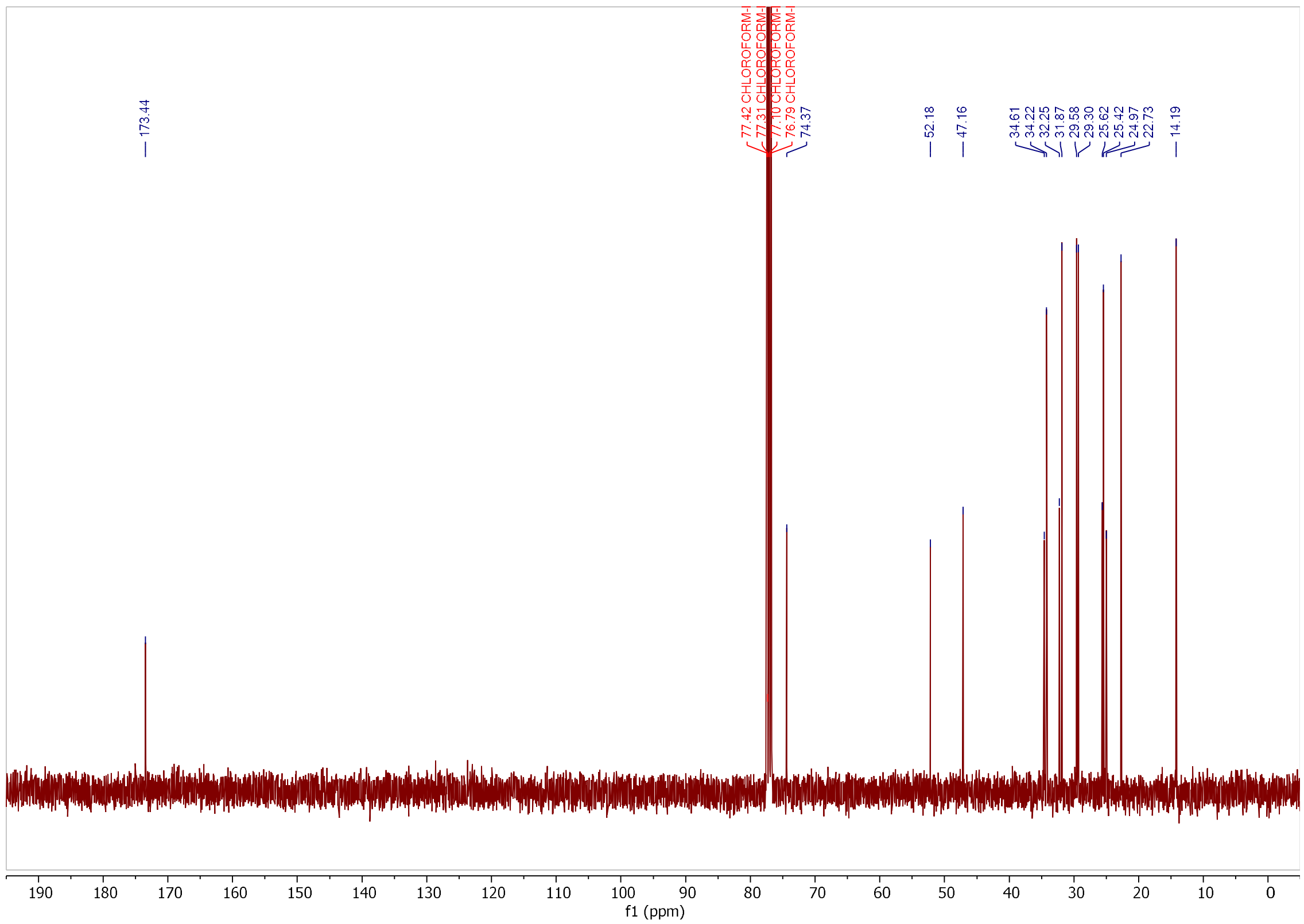

^{13}C-NMR spectrum of compound **4e** ($CDCl_3$, 101 MHz)

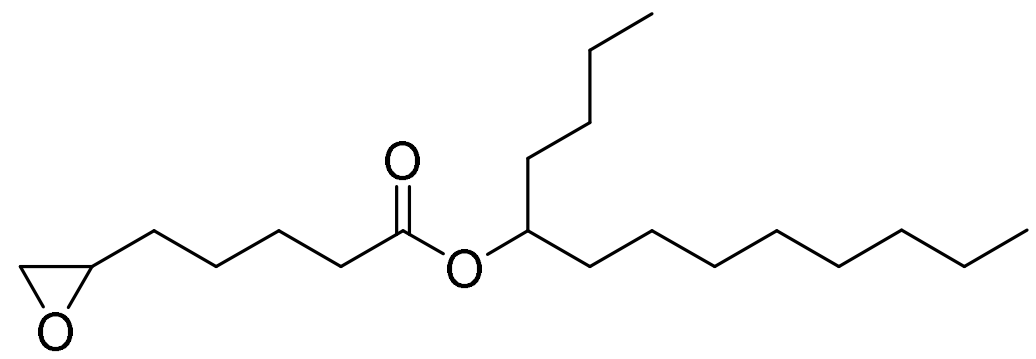

Figure S28. Chemical structure of compound 4f.

^{1}H NMR (500 MHz, $CDCl_3$) δ 4.80 (p, *J* = 6.3 Hz, 1H), 2.87 – 2.80 (m, 1H), 2.70 – 2.65 (m, 1H), 2.40 (dd, *J* = 5.0, 2.7 Hz, 1H), 2.24 (t, *J* = 7.5 Hz, 2H), 1.67-1.57 (m, 2H), 1.51 – 1.39 (m, 8H), 1.28 – 1.13 (m, 16H), 0.85 – 0.78 (m, 6H).

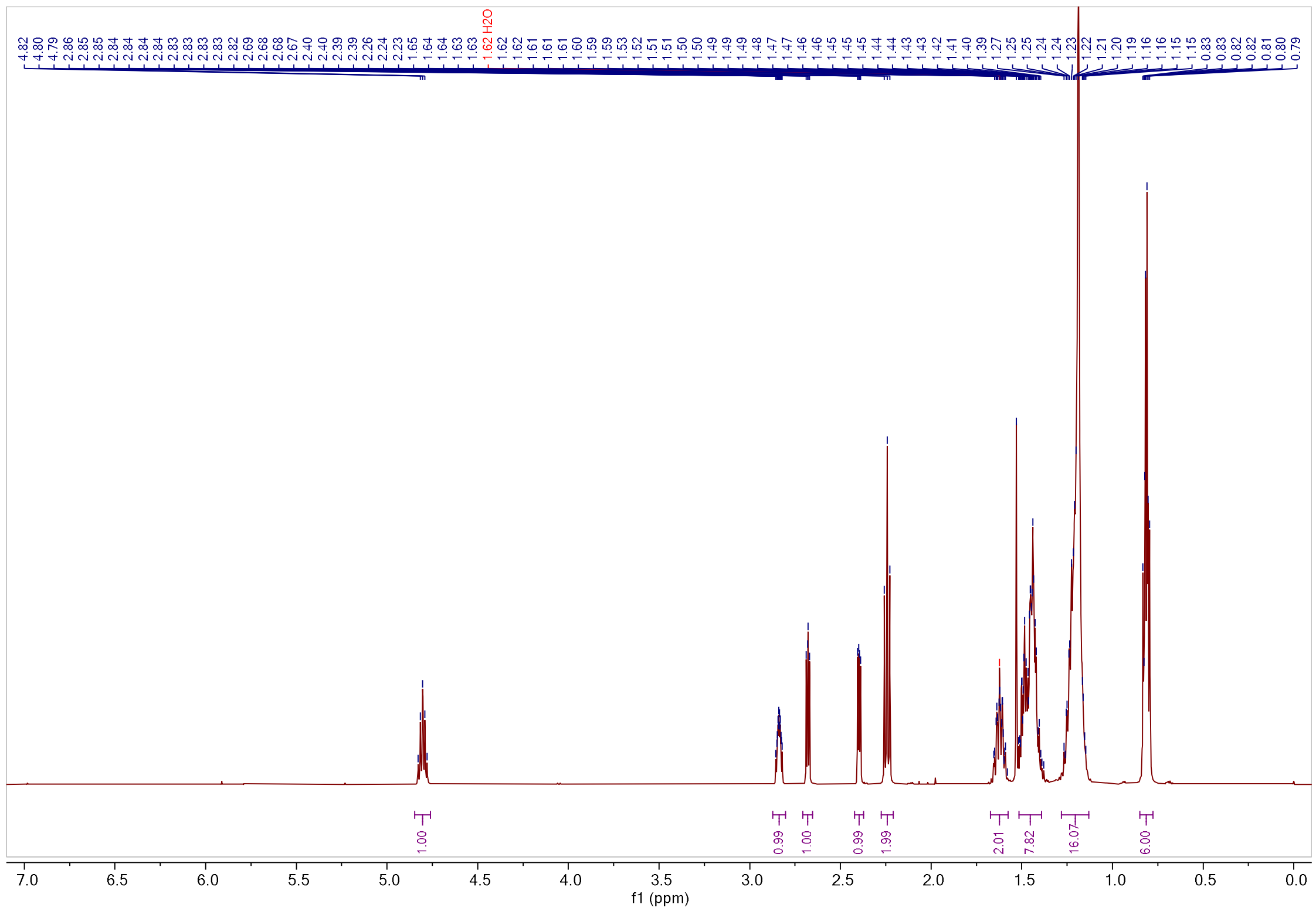

^{1}H NMR spectrum of compound **4f** ($CDCl_3$, 500 MHz)

^{13}C NMR (101 MHz, $CDCl_3$) δ 173.46, 74.35, 52.18, 47.16, 34.60, 34.23, 33.92, 32.25, 31.95, 29.62, 29.33, 27.59, 25.62, 25.42, 22.75, 22.69, 14.20, 14.11.

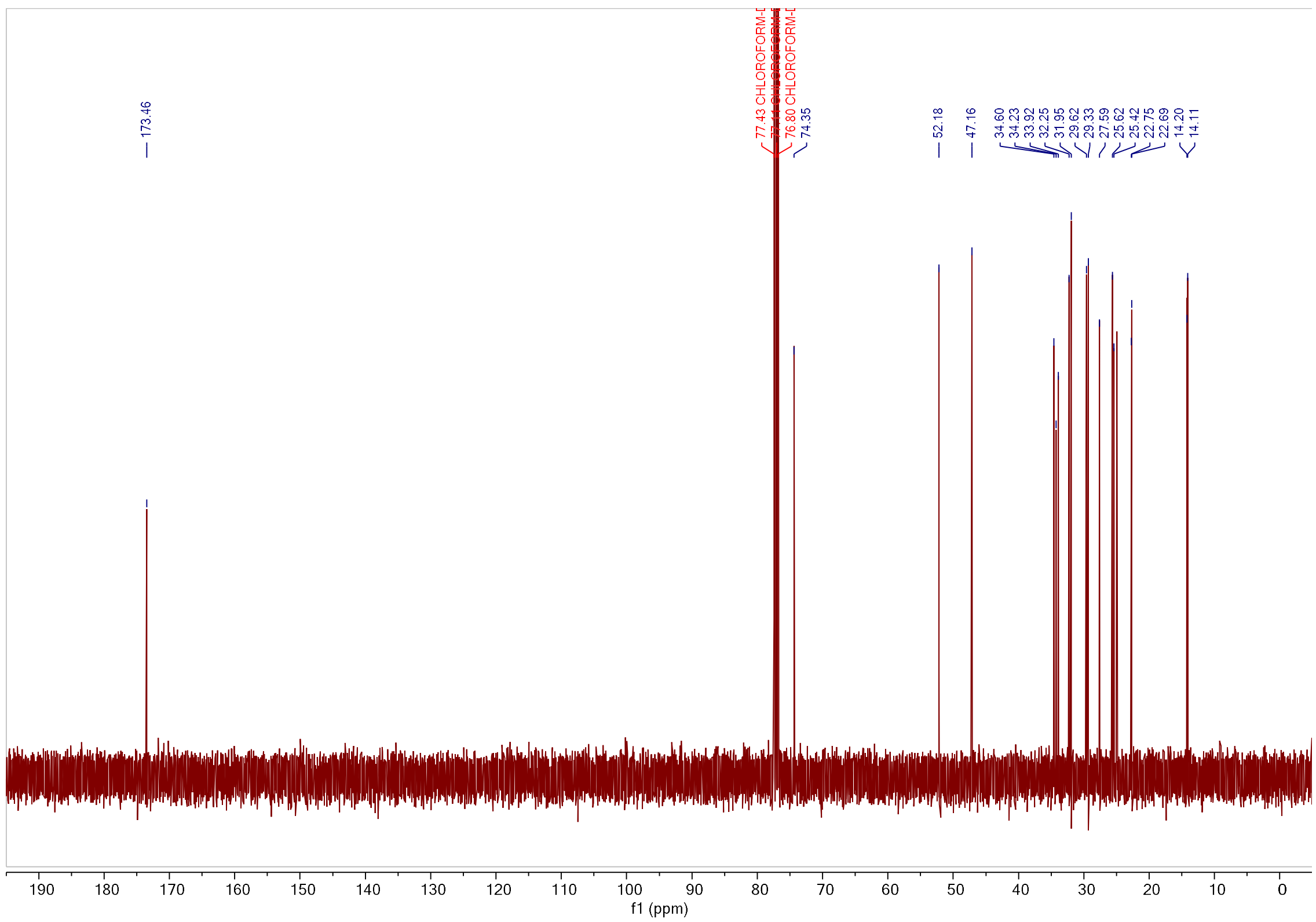

^{13}C-NMR spectrum of compound **4f** ($CDCl_3$, 101 MHz)

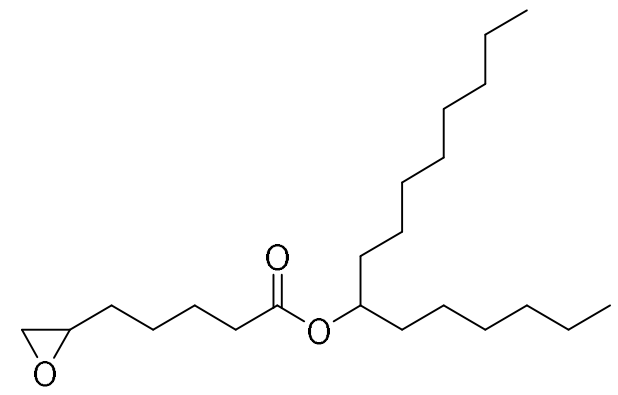

Figure S29. Chemical structure of compound 4g.

^{1}H NMR (500 MHz, $CDCl_3$) δ 4.80 (p, *J* = 7Hz, 1H), 2.87– 2.80 (m, 1H), 2.70 – 2.65 (m, 1H), 2.40 (dd, *J* = 5.0, 2.7 Hz, 2H), 2.24 (t, *J* = 7.4 Hz, 2H), 1.68 – 1.58 (m, 2H), 1.52 – 1.38 (m, 8H), 1.20 (d, *J* = 10.6 Hz, 20H), 0.84 – 0.77 (m, 6H).

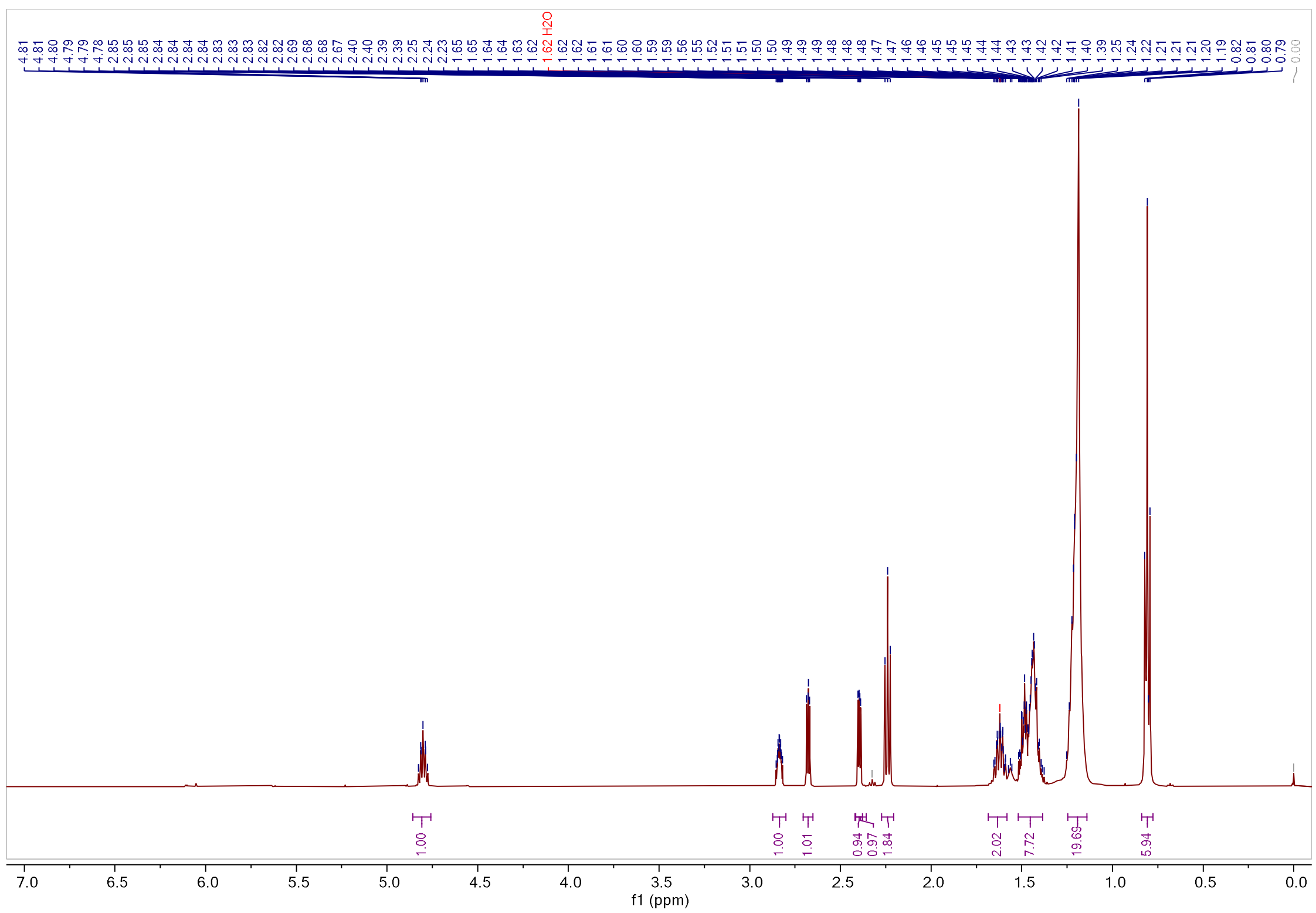

^{1}H NMR spectrum of compound **4g** ($CDCl_3$, 500 MHz)

^{13}C NMR (126 MHz, $CDCl_3$) δ 173.33, 74.28, 52.07, 47.04, 34.52, 34.14, 32.17, 31.86, 31.76, 29.54, 29.51, 29.24, 29.20, 25.54, 25.33, 25.29, 24.89, 22.67, 22.58, 14.10, 14.07.

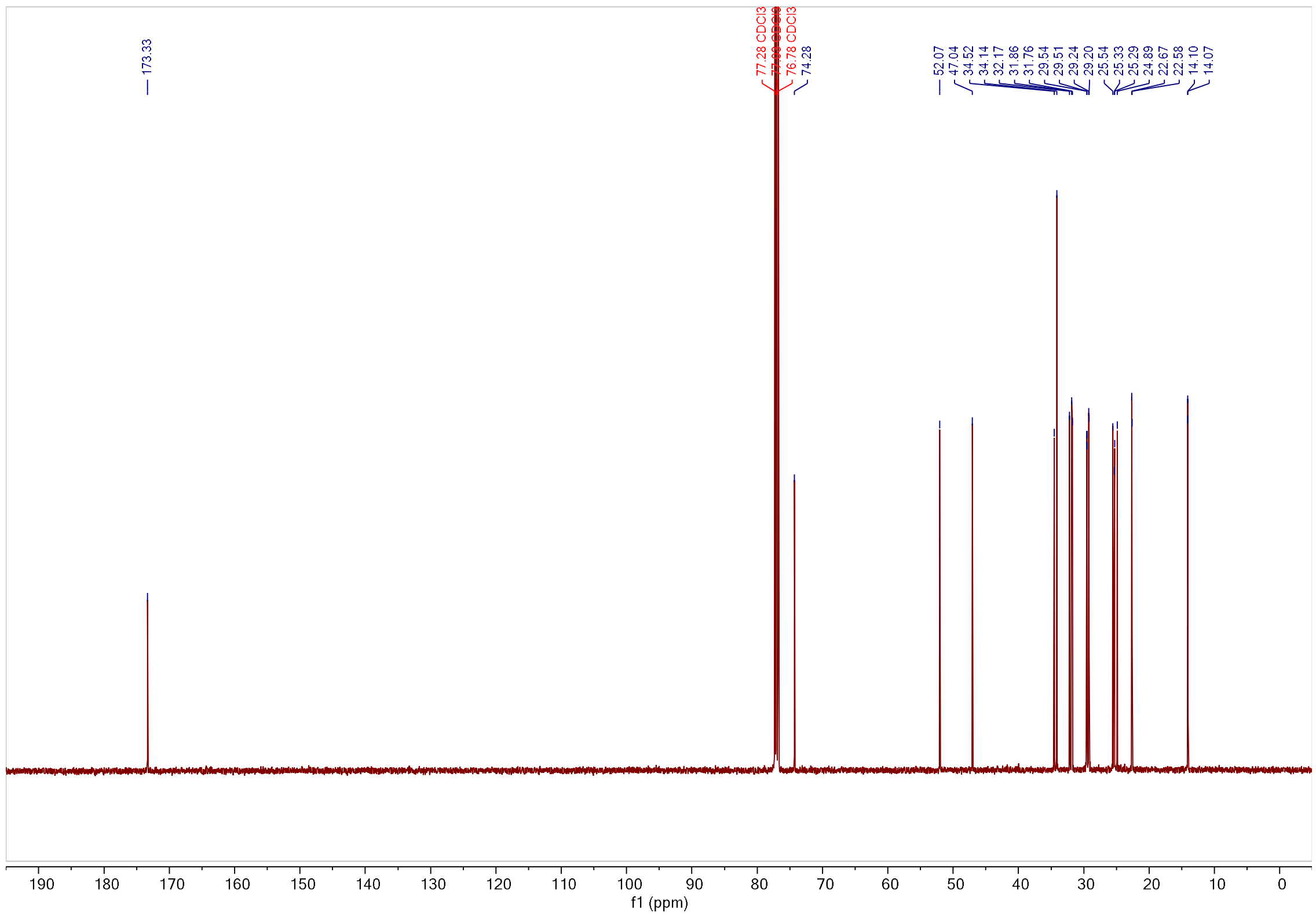

^{13}C-NMR spectrum of compound **4g** ($CDCl_3$, 126 MHz)

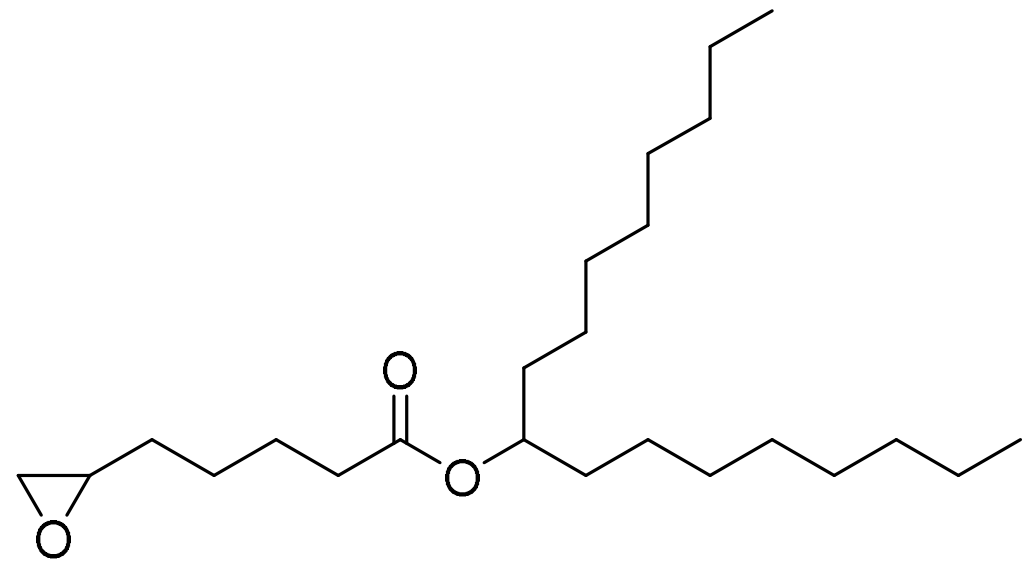

Figure S30. Chemical structure of compound 4h.

^{1}H NMR (500 MHz, $CDCl_3$) δ 4.80 (p, *J* = 6.3 Hz, 1H), 2.87– 2.80 (m, 1H), 2.70 – 2.66 (m, 1H), 2.40 (dd, *J* = 5.0, 2.7 Hz, 1H), 2.24 (t, *J* = 7.4 Hz, 2H), 1.67 – 1.57 (m, 2H), 1.51 – 1.37 (m, 8H), 1.25 – 1.14 (m, 24H), 0.81 (t, *J* = 6.9 Hz, 6H).

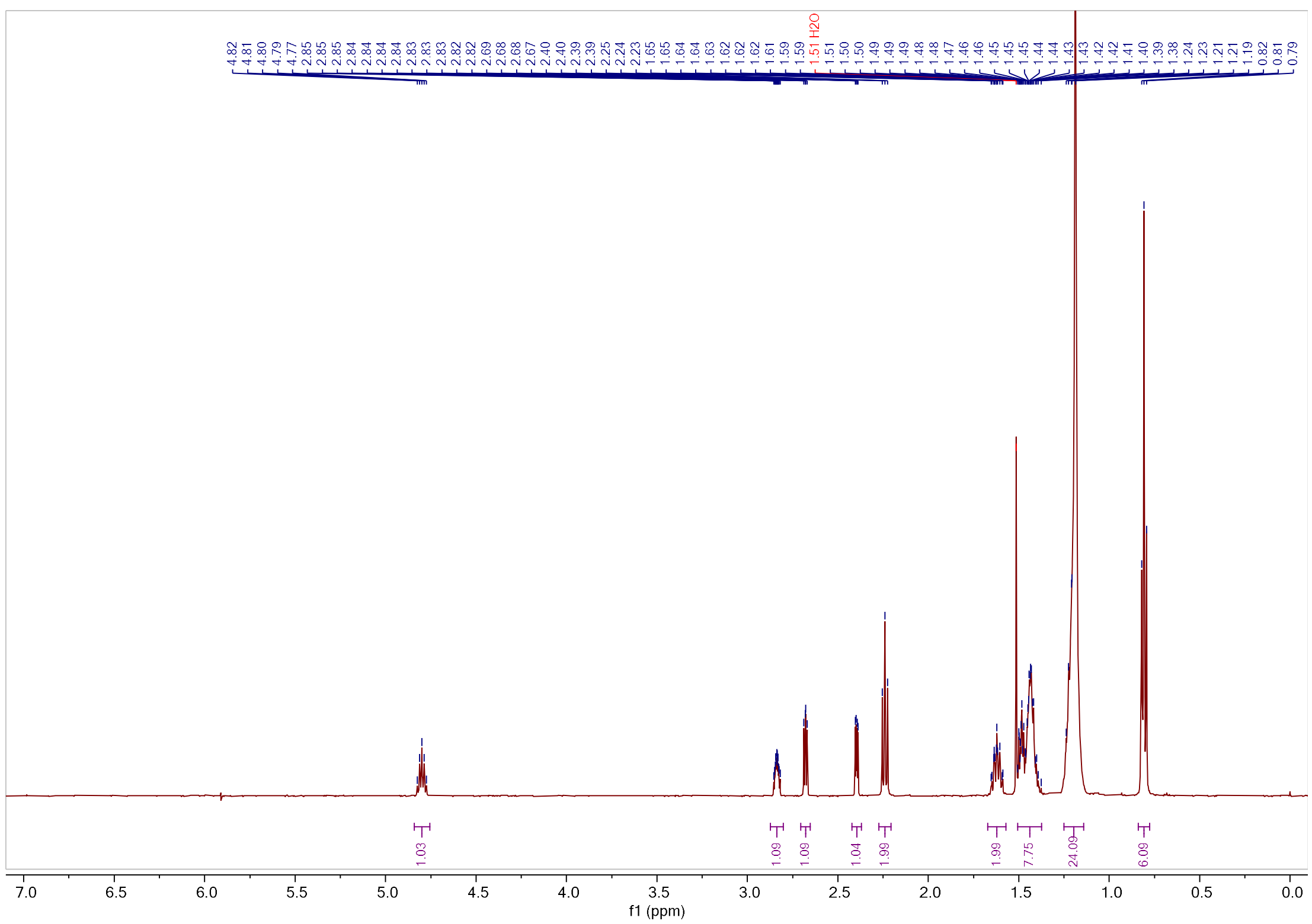

^{1}H NMR spectrum of compound **4h** ($CDCl_3$, 500 MHz)

^{13}C NMR (101 MHz, $CDCl_3$) δ 173.41, 74.36, 52.15, 34.60, 34.22, 32.24, 31.94, 29.61, 29.58, 29.32, 25.62, 25.41, 24.97, 22.74, 14.17.

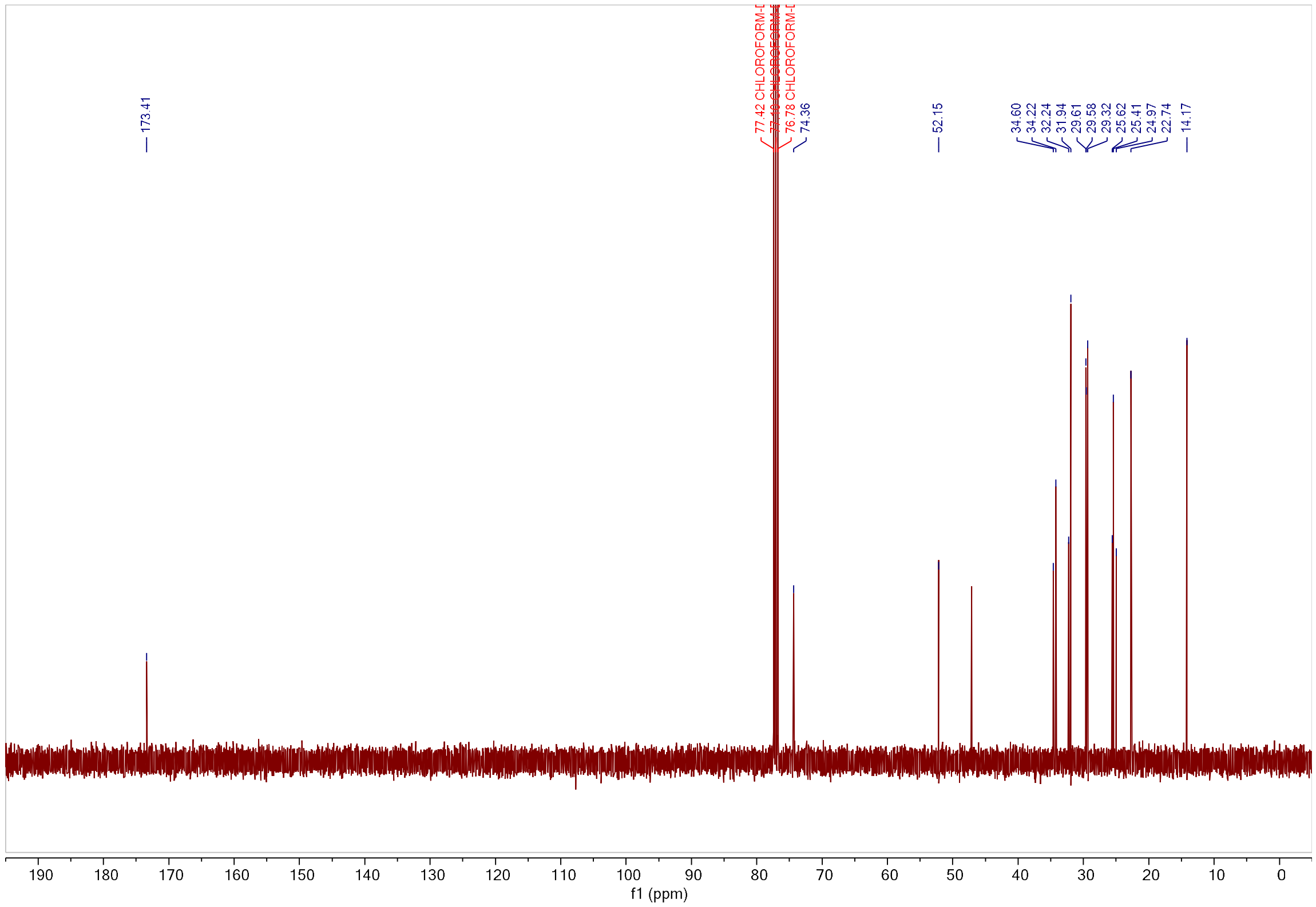

^{13}C-NMR spectrum of compound **4h** ($CDCl_3$, 101 MHz)

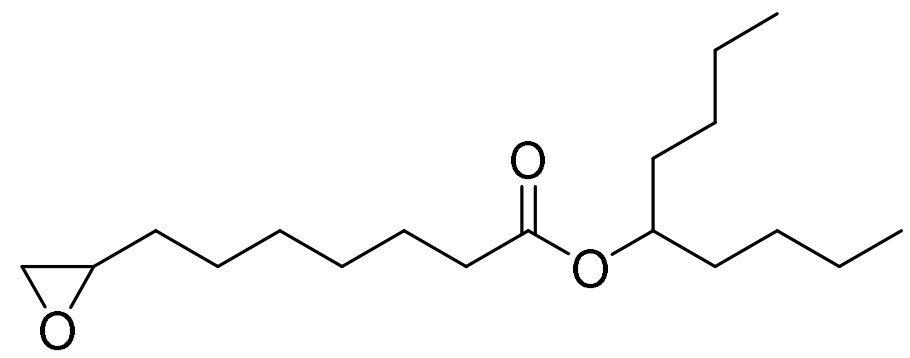

Figure S31. Chemical structure of compound 4i.

^{1}H NMR (500 MHz, $CDCl_3$) δ 4.80 (p, *J* = 6.3 Hz, 1H), 2.87 – 2.79 (m, 1H), 2.70 – 2.65 (m, 1H), 2.39 (dd, *J* = 5.0, 2.7 Hz, 1H), 2.22 (t, *J* = 7.5 Hz, 2H), 1.59-1.52 (m, 2H), 1.49 – 1.34 (m, 8H), 1.33 – 1.12 (m, 12H), 0.85 – 0.78 (m, 6H).

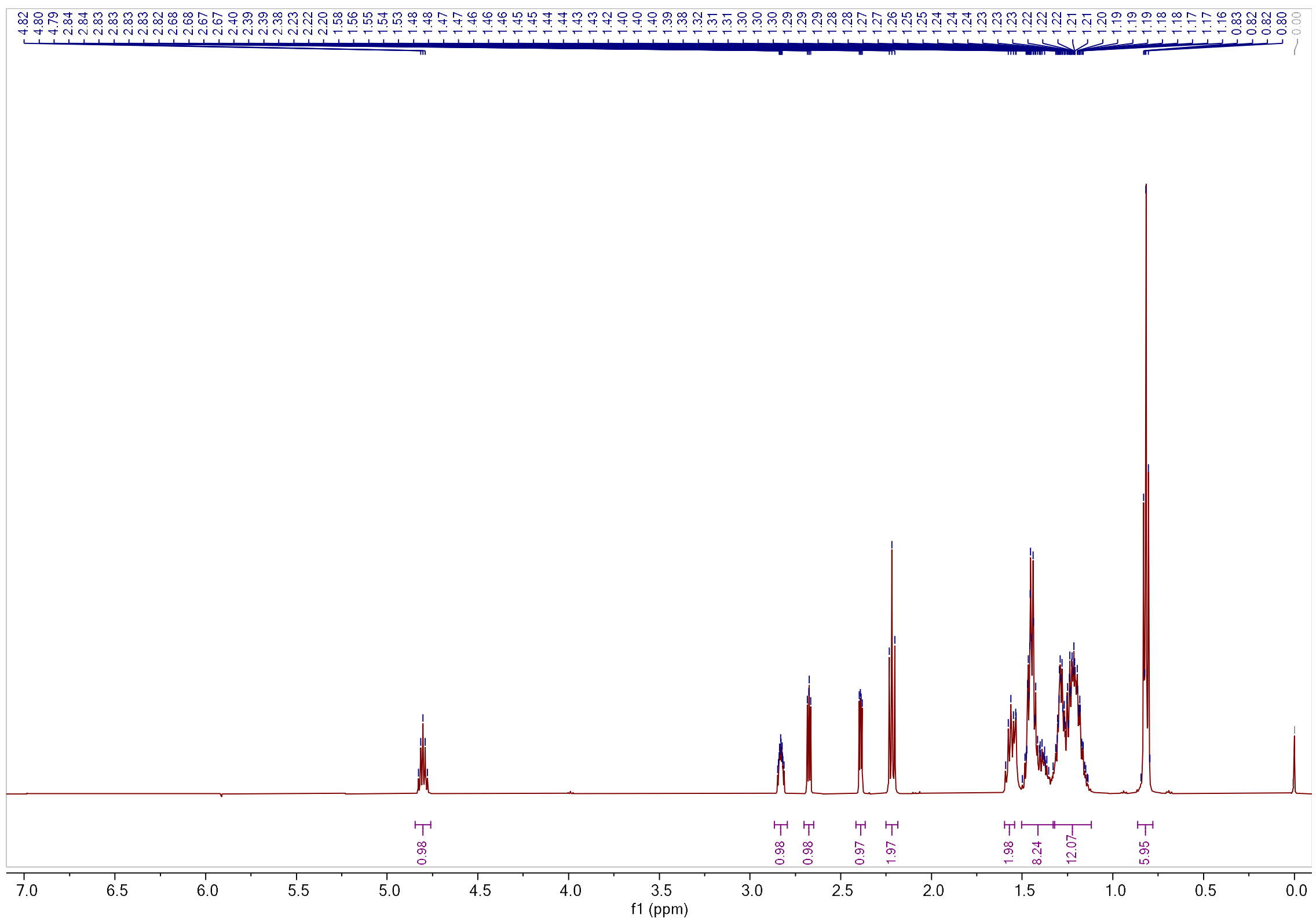

^{1}H NMR spectrum of compound **4i** ($CDCl_3$, 500 MHz)

^{13}C NMR (101 MHz, $CDCl_3$) δ 173.72, 74.22, 52.42, 47.19, 34.74, 34.24, 33.94, 32.51, 31.95, 29.63, 29.60, 29.33, 29.19, 29.16, 27.59, 25.92, 25.41, 25.12, 22.75, 22.69, 14.20, 14.11.

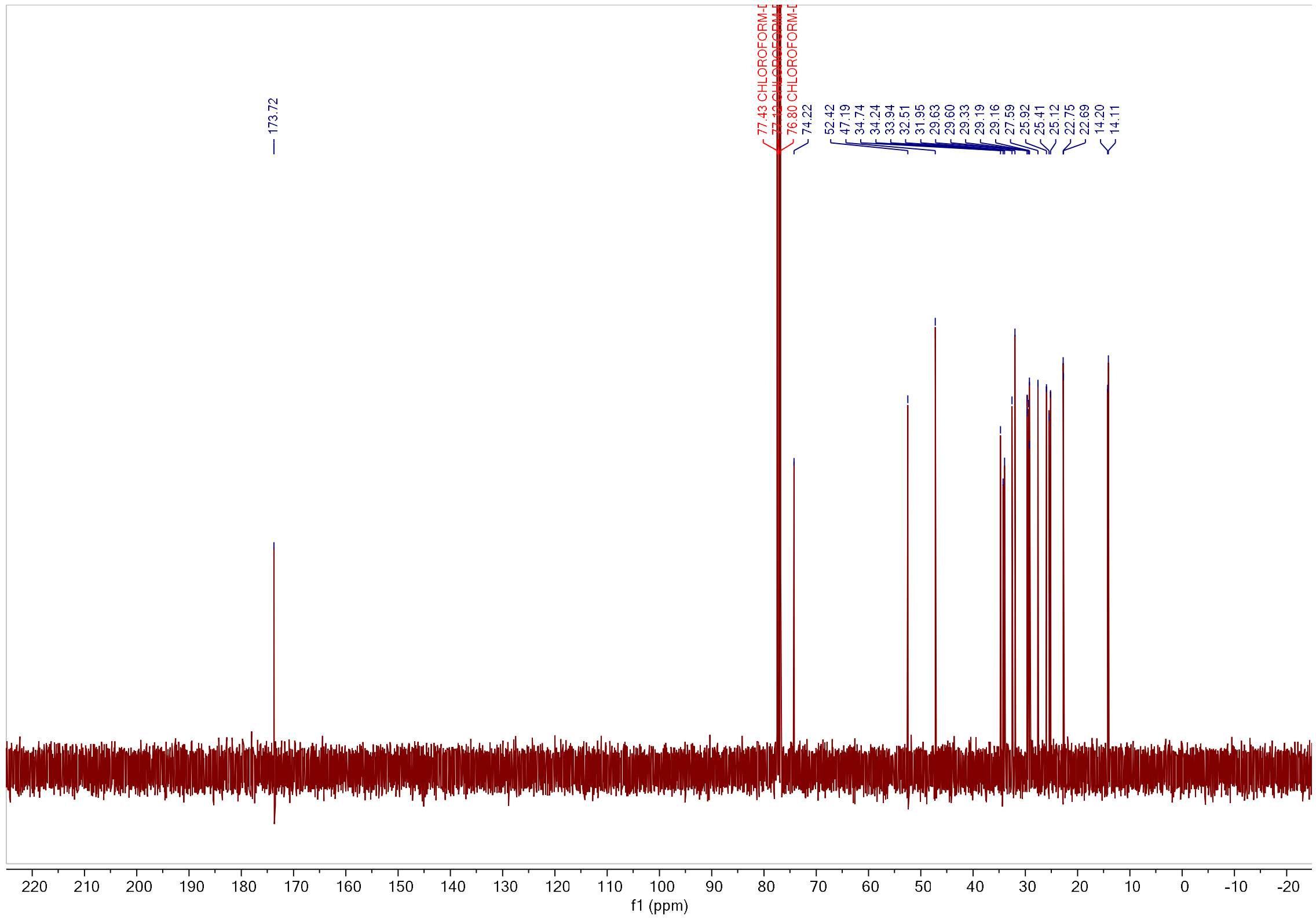

^{13}C-NMR spectrum of compound **4i** ($CDCl_3$, 101 MHz)

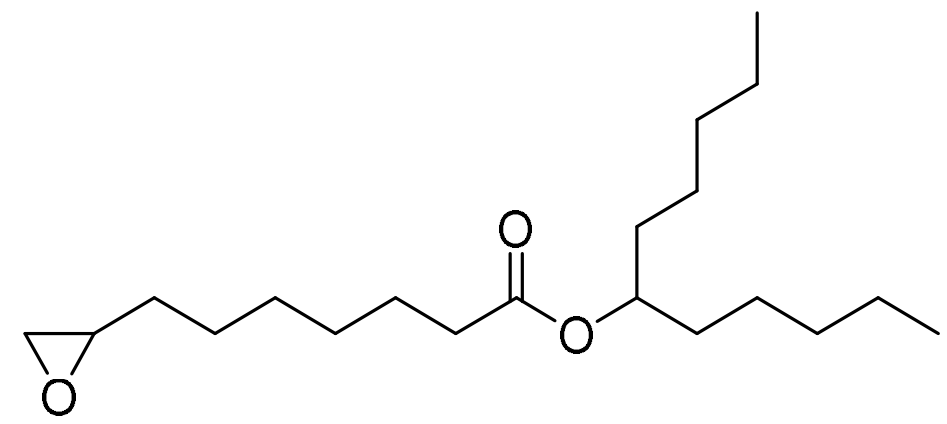

Figure S32. Chemical structure of compound 4j.

^{1}H NMR (500 MHz, $CDCl_3$) δ 4.80 (p, *J* = 6.5 Hz, 1H), 2.86 – 2.79 (m, 1H), 2.69 – 2.66 (m, 1H), 2.39 (dd, *J* = 5.0, 2.7 Hz, 1H), 2.21 (t, *J* = 7.5 Hz, 2H), 1.59-1.54 (m, 2H), 1.48 – 1.34 (m, 8H), 1.31 – 1.13 (m, 16H), 0.84 – 0.78 (m, 6H).

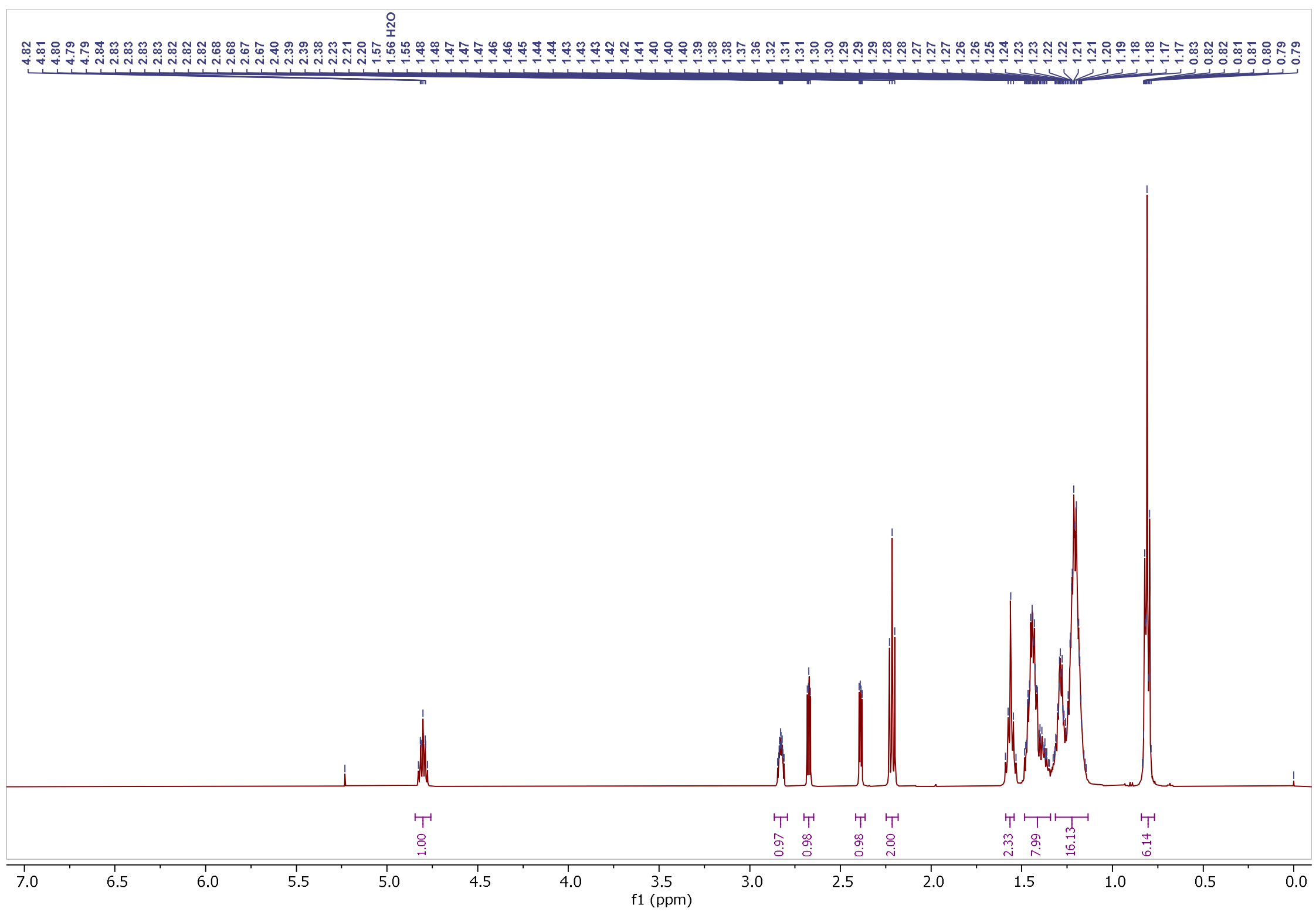

^{1}H NMR spectrum of compound **4j** ($CDCl_3$, 500 MHz)

^{13}C NMR (126 MHz, $CDCl_3$) δ 173.60, 74.14, 52.32, 47.08, 34.66, 34.12, 32.42, 31.72, 29.10, 29.07, 25.83, 25.04, 24.99, 22.56, 14.00.

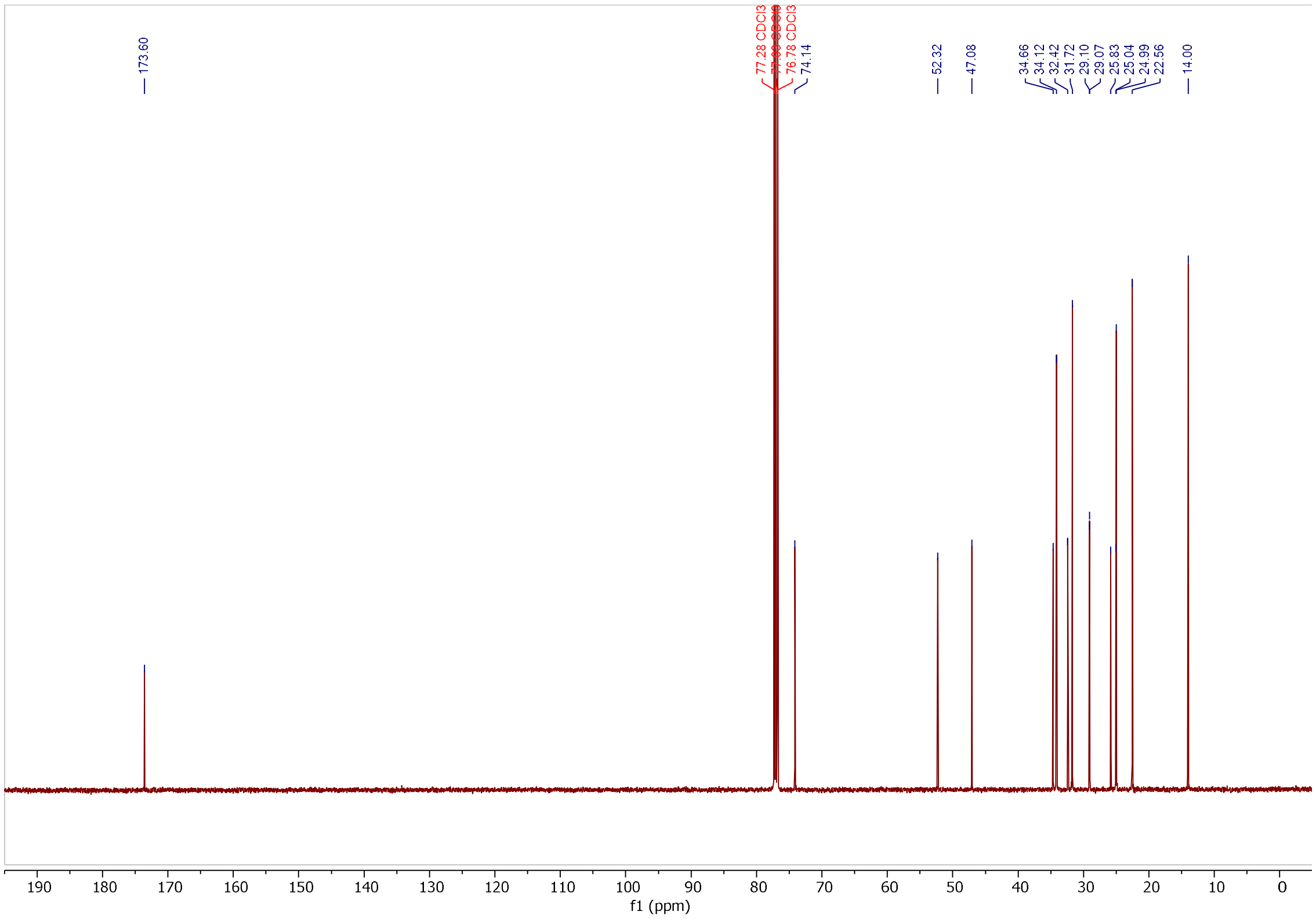

^{13}C-NMR spectrum of compound **4j** ($CDCl_3$, 126 MHz)

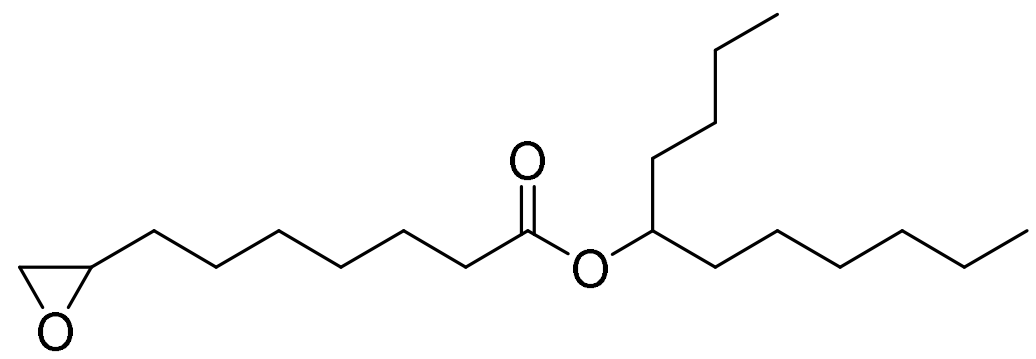

Figure S33. Chemical structure of compound 4k.

^{1}H NMR (500 MHz, $CDCl_3$) δ 4.80 (p, *J* = 6.2 Hz, 1H), 2.86 – 2.79 (m, 1H), 2.69 – 2.66 (m, 1H), 2.39 (dd, *J* = 5.0, 2.7 Hz, 1H), 2.22 (t, *J* = 7.5 Hz, 2H), 1.57 – 1.52 (m, 2H), 1.47 – 1.34 (m, 8H), 1.33 – 1.13 (m, 16H), 0.85 – 0.77 (m, 6H).

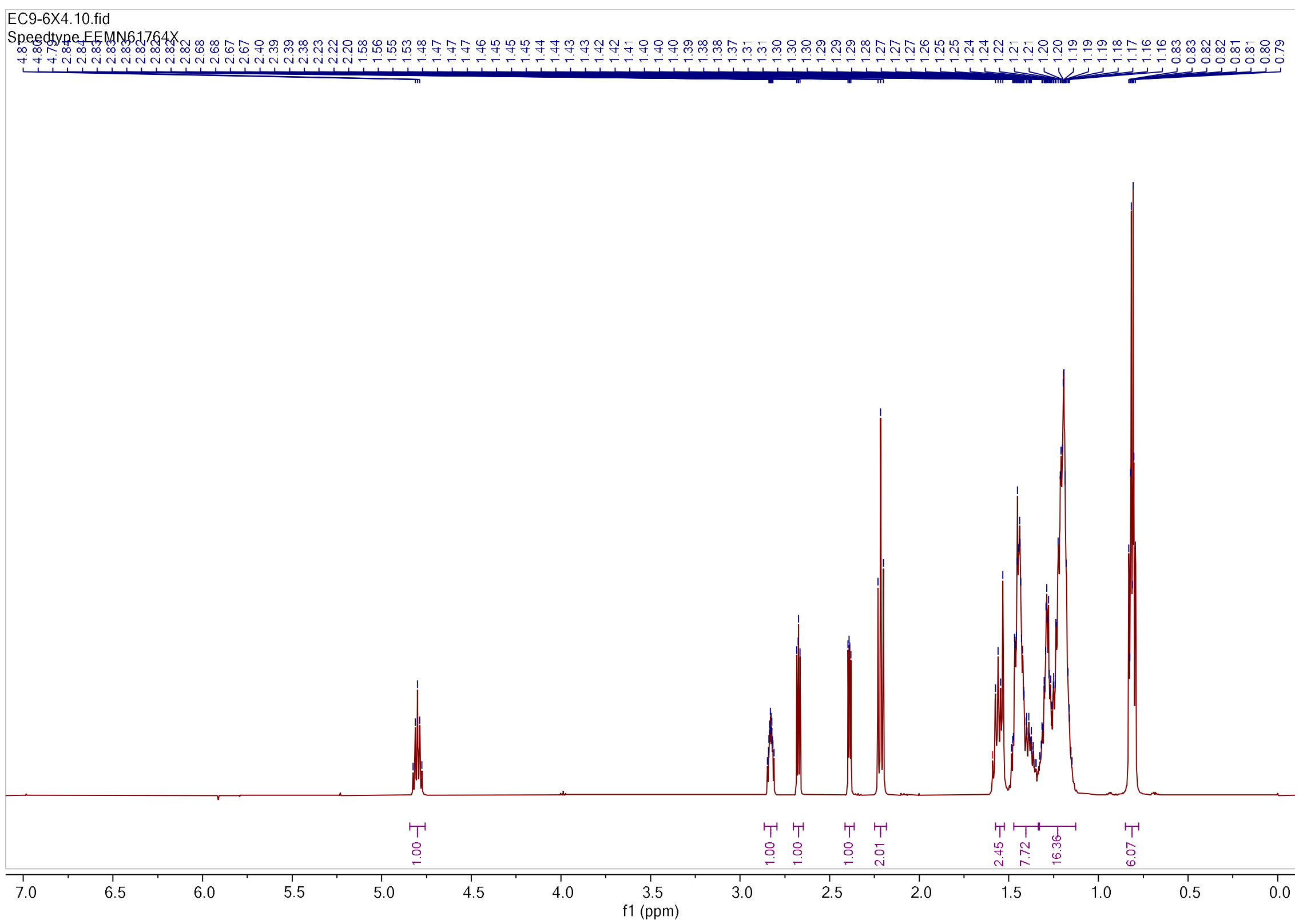

^{1}H NMR spectrum of compound **4k** ($CDCl_3$, 500 MHz)

^{13}C NMR (101 MHz, $CDCl_3$) δ 173.72, 74.22, 52.42, 47.19, 34.74, 34.25, 33.94, 32.50, 31.85, 29.29, 29.19, 29.16, 27.59, 25.92, 25.38, 25.11, 22.69, 22.67, 14.17, 14.11.

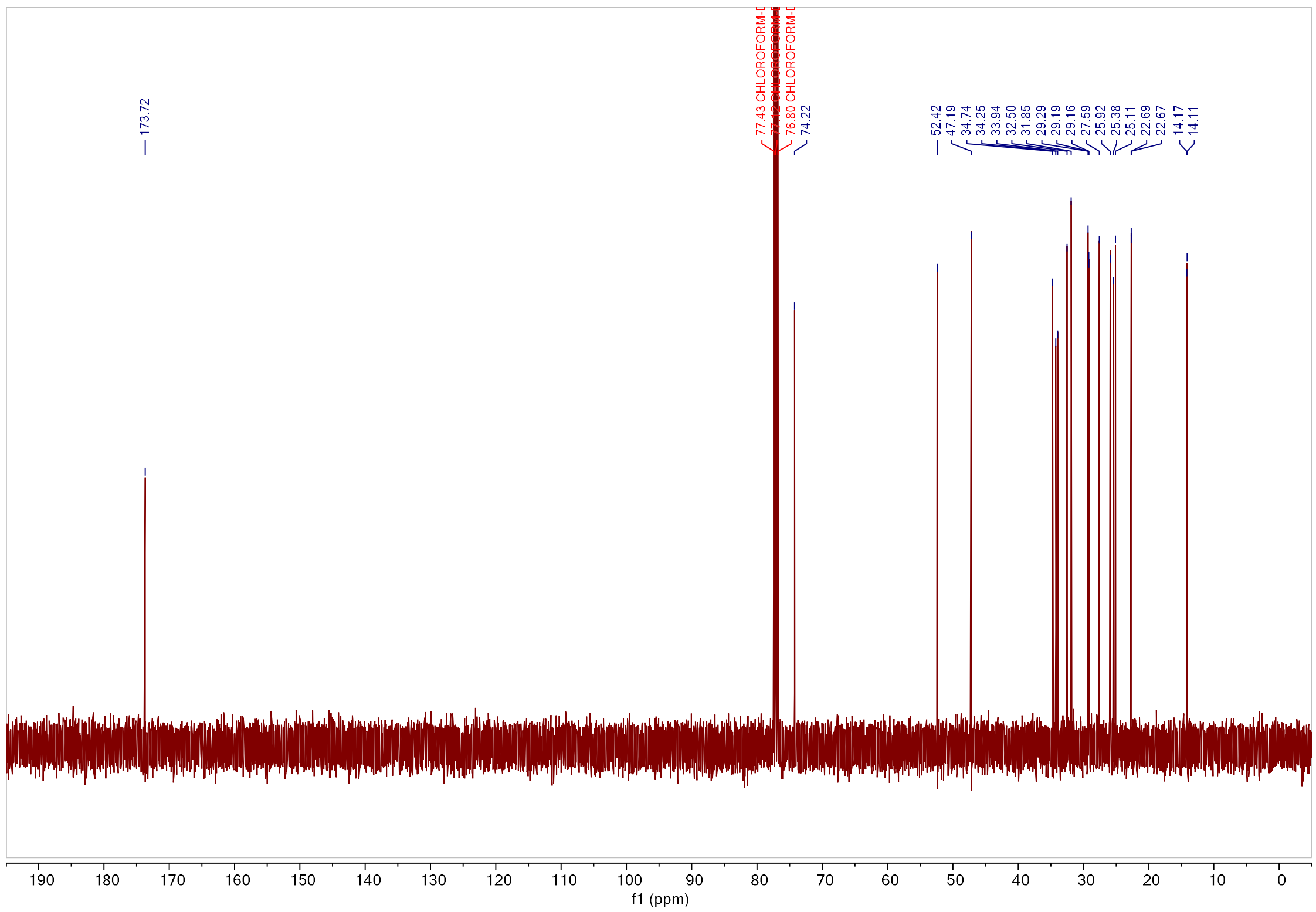

^{13}C-NMR spectrum of compound **4k** ($CDCl_3$, 101 MHz)

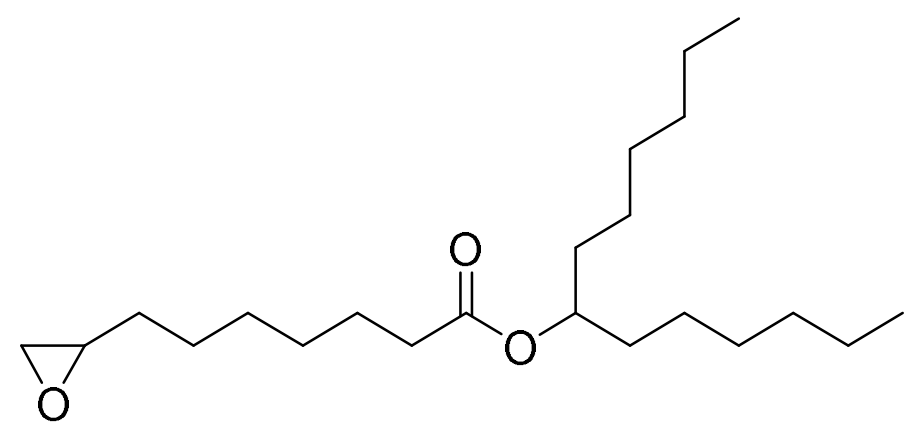

Figure S34. Chemical structure of compound 4l.

^{1}H NMR (500 MHz, $CDCl_3$) δ 4.89 (p, *J* = 6 Hz, 1H), 2.95 – 2.88 (m, 1H), 2.78 – 2.74 (m, 1H), 2.48 (dd, *J* = 5.0, 2.7 Hz, 1H), 2.30 (t, *J* = 7.5 Hz, 2H), 1.68-1.62 (m, 2H), 1.58 – 1.44 (m, 8H), 1.43 – 1.23 (m, 20H), 0.90 (t, *J* = 6.9 Hz, 6H).

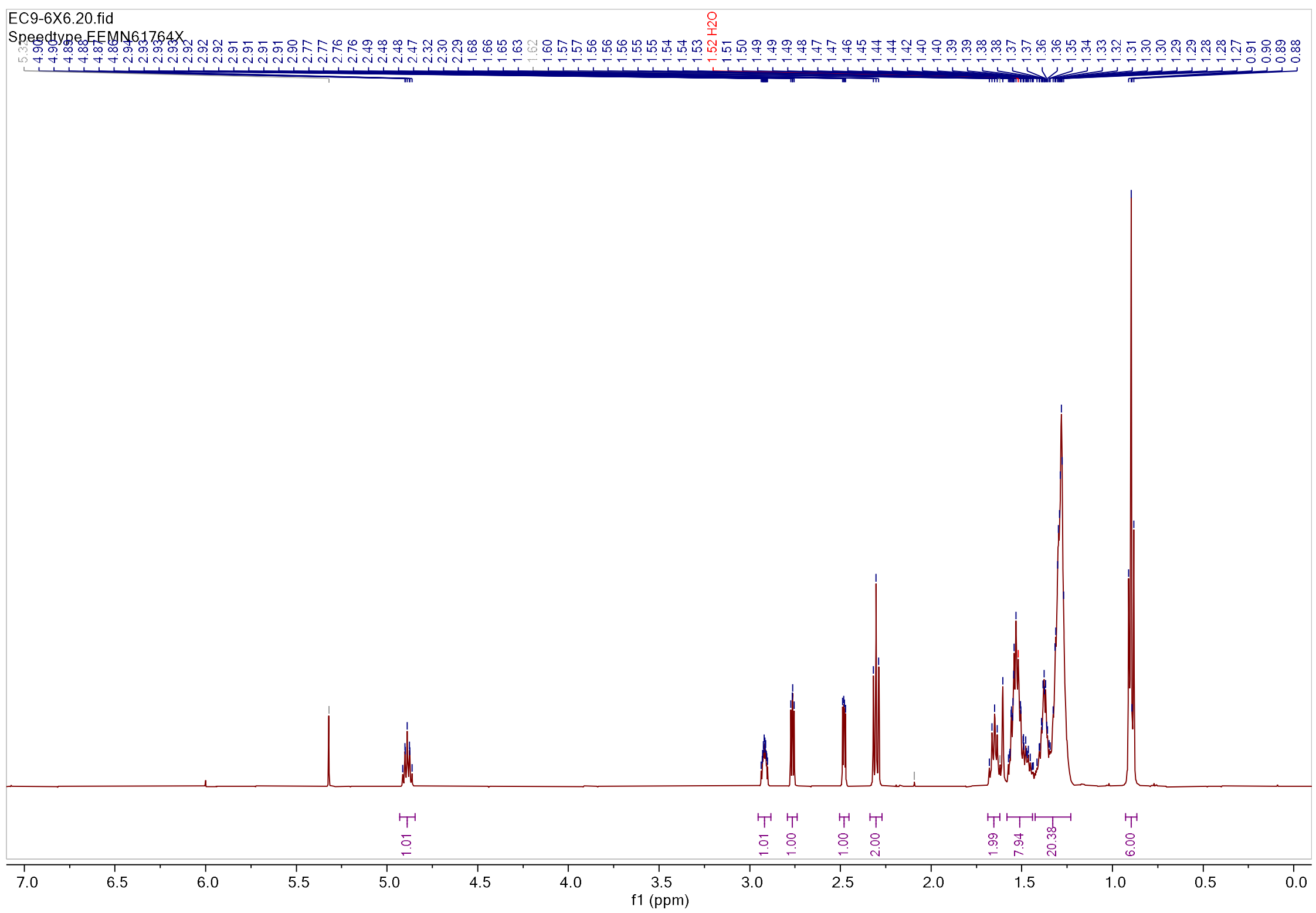

^{1}H NMR spectrum of compound **4l** ($CDCl_3$, 500 MHz)

^{13}C NMR (101 MHz, $CDCl_3$) δ 173.67, 74.23, 52.39, 47.16, 34.74, 34.24, 32.50, 31.84, 29.28, 29.18, 29.16, 25.91, 25.37, 25.12, 22.66, 14.14.

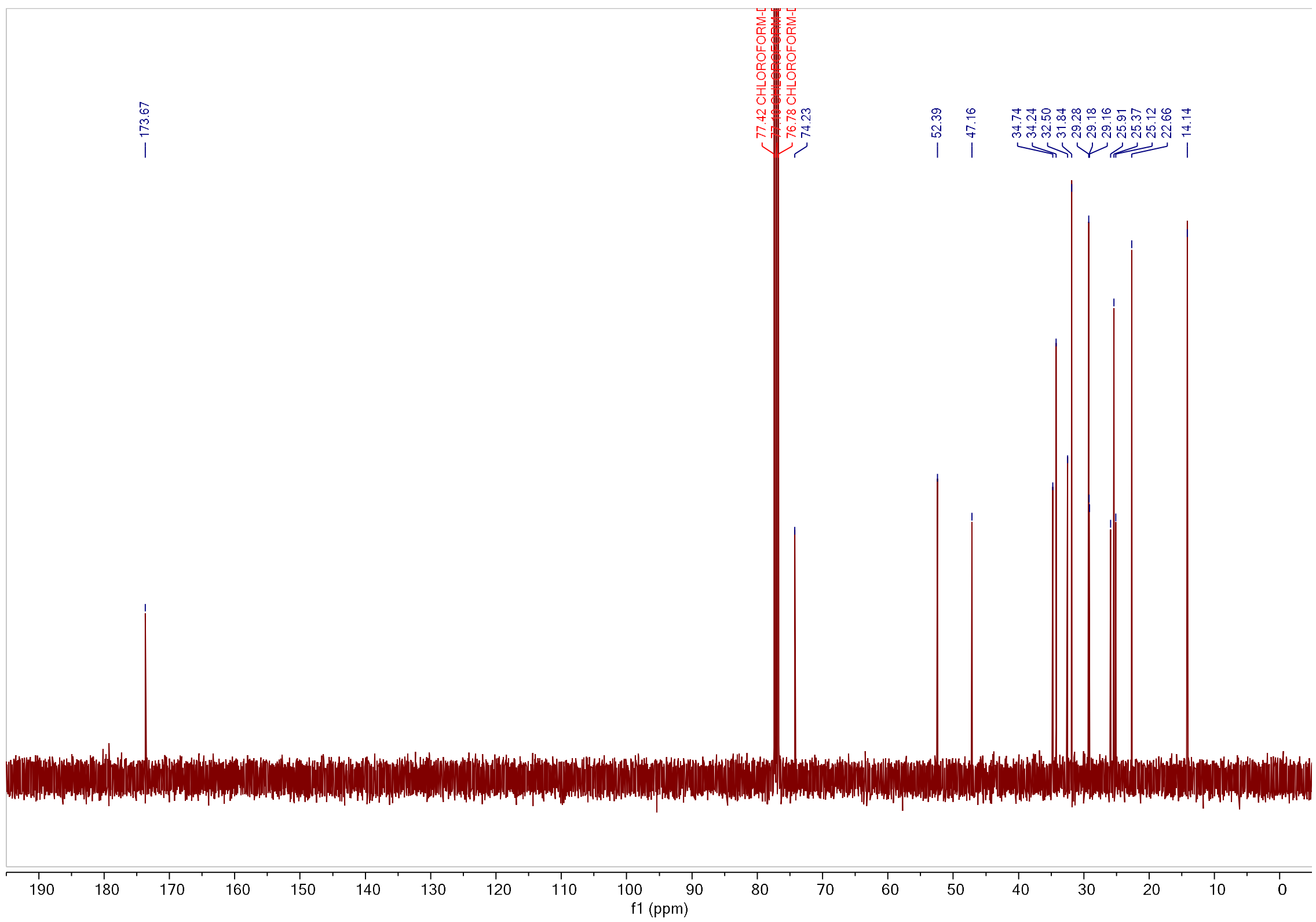

^{13}C-NMR spectrum of compound **4l** ($CDCl_3$, 101 MHz)

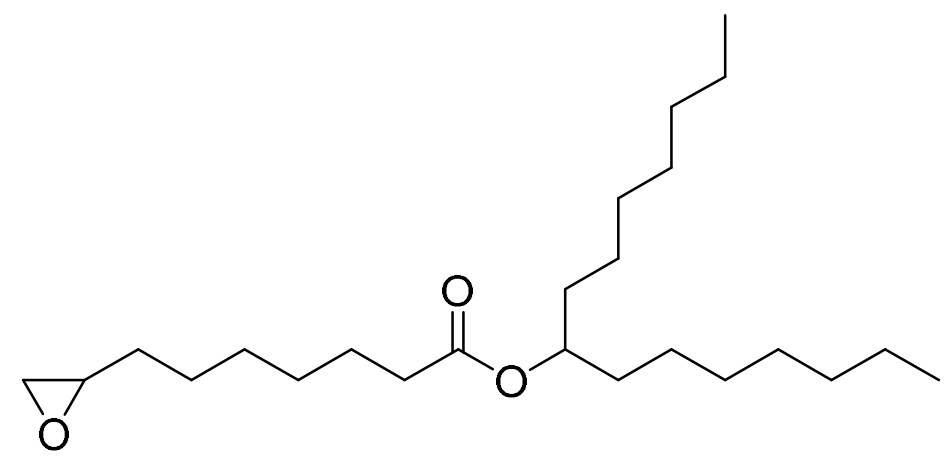

Figure S35. Chemical structure of compound 4m.

^{1}H NMR (500 MHz, $CDCl_3$) δ 4.84 – 4.75 (m, 1H), 2.86 – 2.79 (m, 1H), 2.69 – 2.66 (m, 1H), 2.39 (dd, *J* = 5.0, 2.7 Hz, 1H), 2.21 (t, *J* = 7.5 Hz, 2H), 1.60 – 1.54 (m, 2H), 1.53 – 1.32 (m, 8H), 1.30 – 1.13 (m, 24H), 0.81 (t, *J* = 7.0 Hz, 6H).

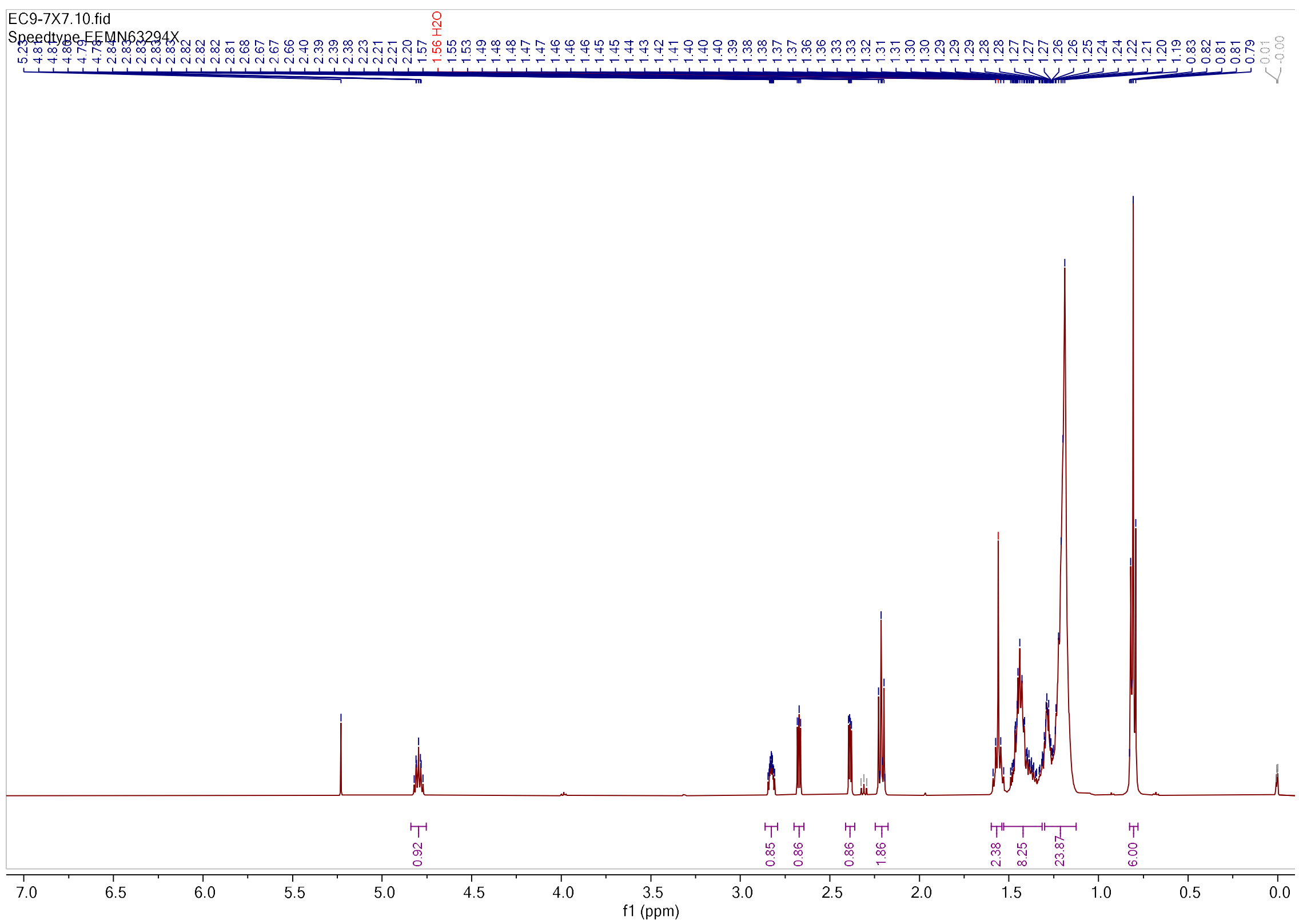

^{1}H NMR spectrum of compound **4m** ($CDCl_3$, 500 MHz)

^{13}C NMR (126 MHz, $CDCl_3$) δ 173.59, 74.15, 52.31, 47.08, 34.66, 34.16, 32.43, 31.79, 29.50, 29.22, 29.11, 29.08, 25.83, 25.34, 25.04, 22.65, 14.09.

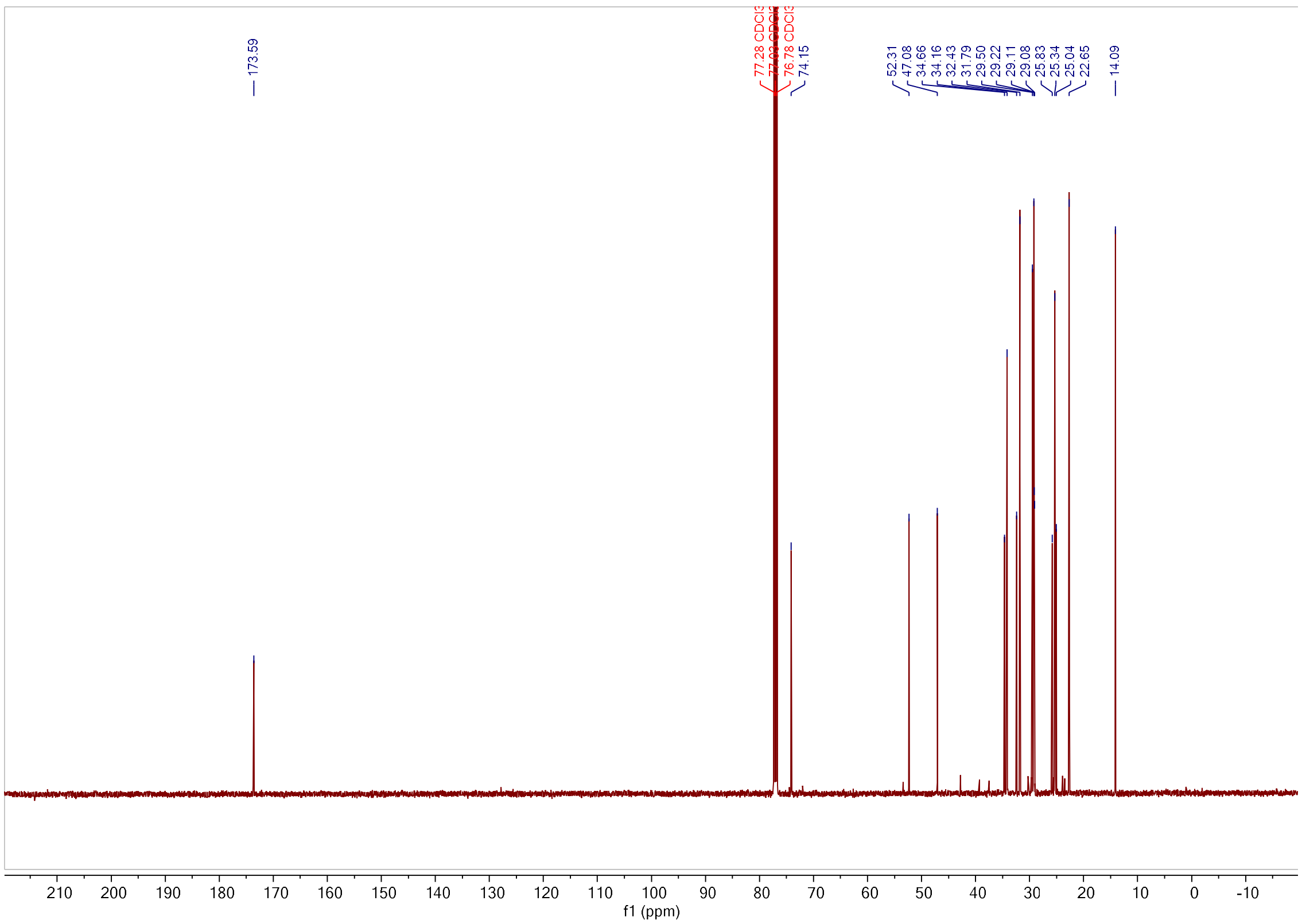

^{13}C-NMR spectrum of compound **4m** ($CDCl_3$, 126 MHz)

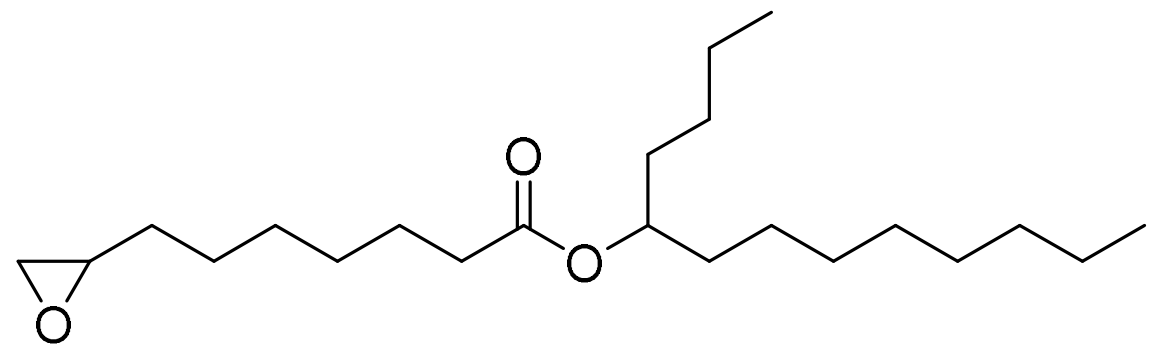

Figure S36. Chemical structure of compound 4n.

^{1}H NMR (500 MHz, $CDCl_3$) δ 4.80 (p, *J* = 6.2 Hz, 1H), 2.83 (m, *J* = 5.3, 3.9, 2.7 Hz, 1H), 2.69 – 2.65 (m, 1H), 2.39 (dd, *J* = 5.0, 2.7 Hz, 1H), 2.22 (t, *J* = 7.5 Hz, 2H), 1.61-1.54 (m, 2H), 1.49 – 1.34 (m, 8H), 1.32 – 1.14 (m, 20H), 0.85 – 0.77 (m, 6H).

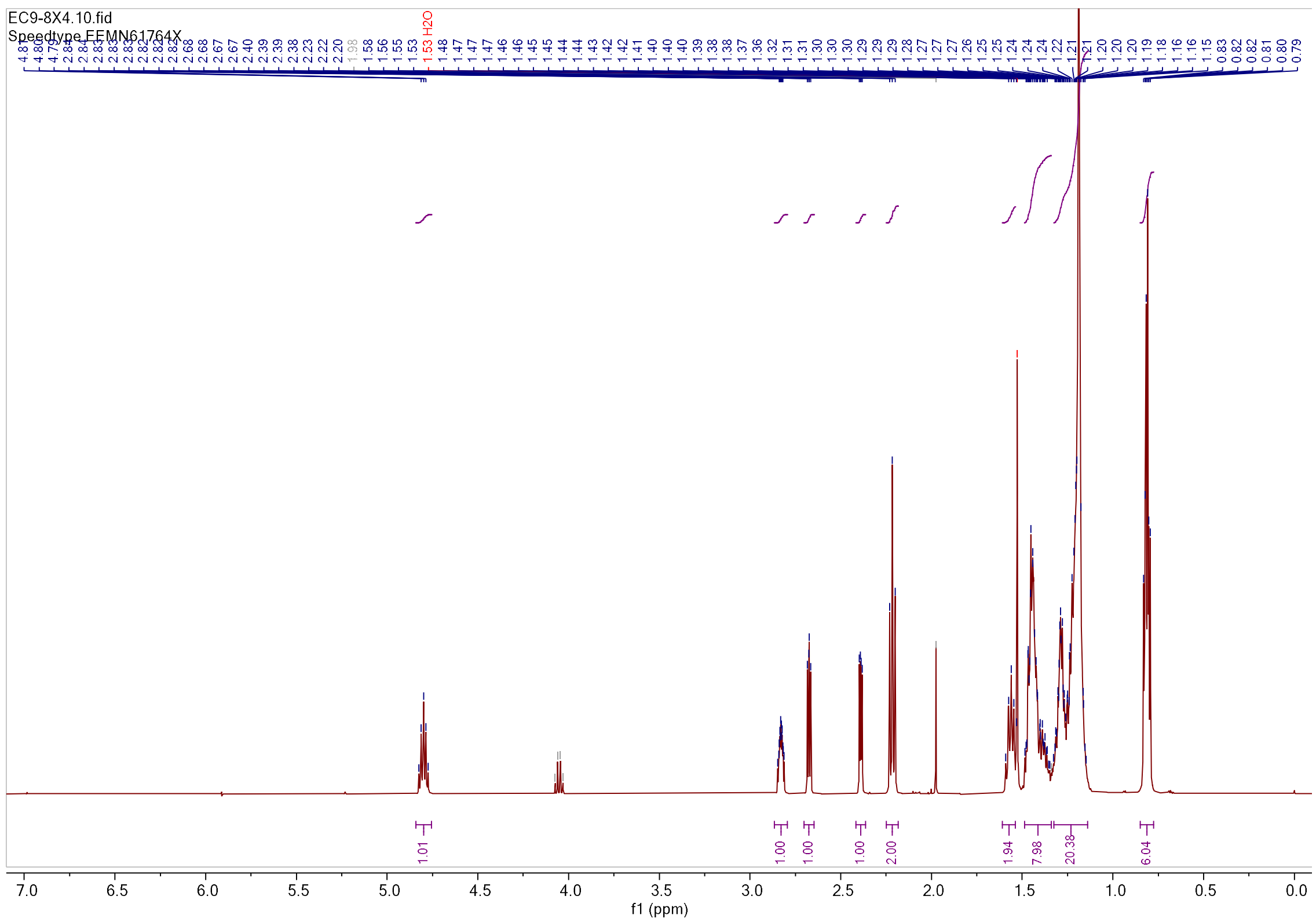

^{1}H NMR spectrum of compound **4n** ($CDCl_3$, 500 MHz)

^{13}C NMR (101 MHz, $CDCl_3$) δ 173.72, 74.19, 52.43, 47.20, 34.73, 33.94, 32.50, 29.18, 29.15, 27.59, 25.92, 25.11, 22.69, 14.11.

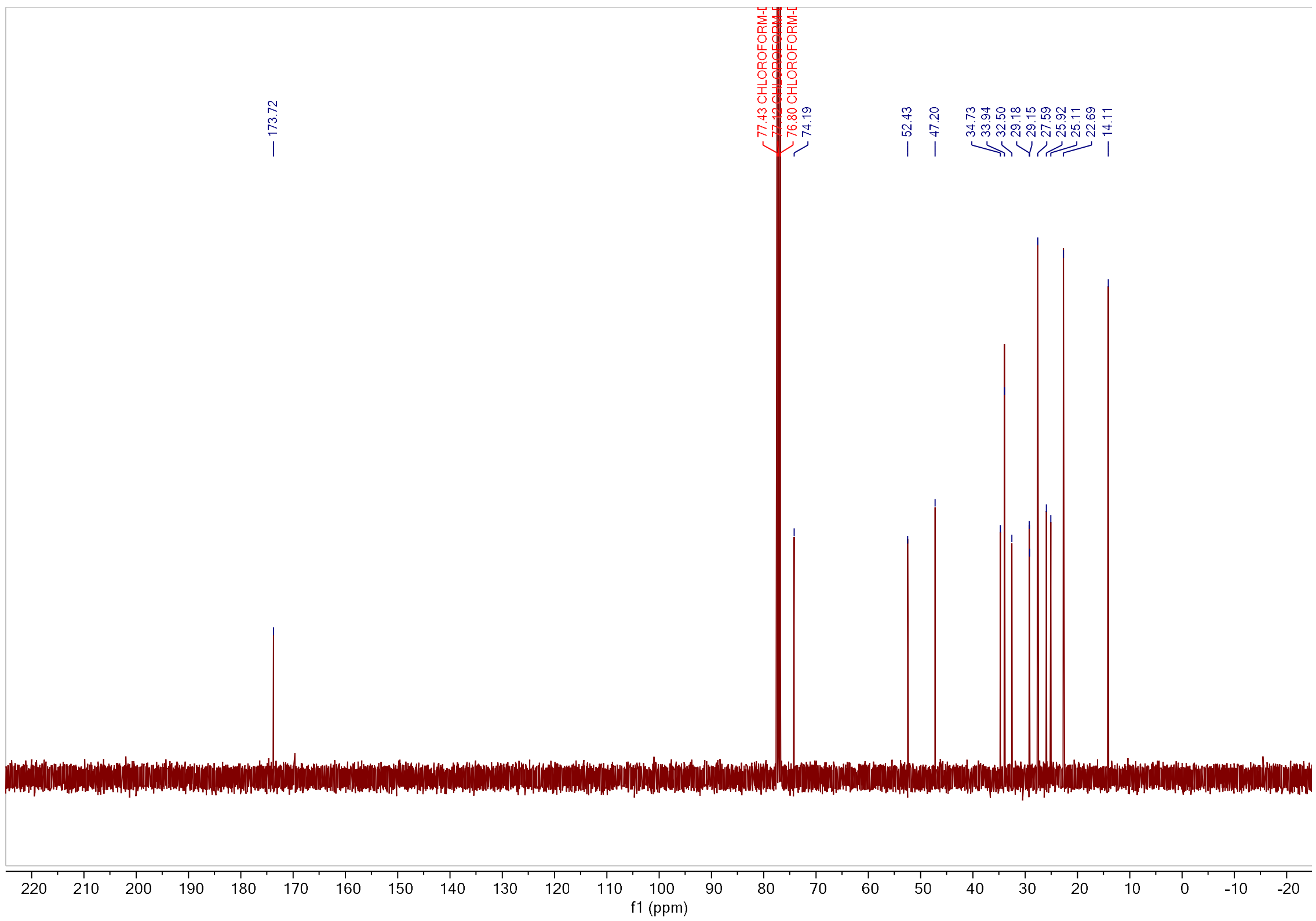

^{13}C-NMR spectrum of compound **4n** ($CDCl_3$, 101 MHz)

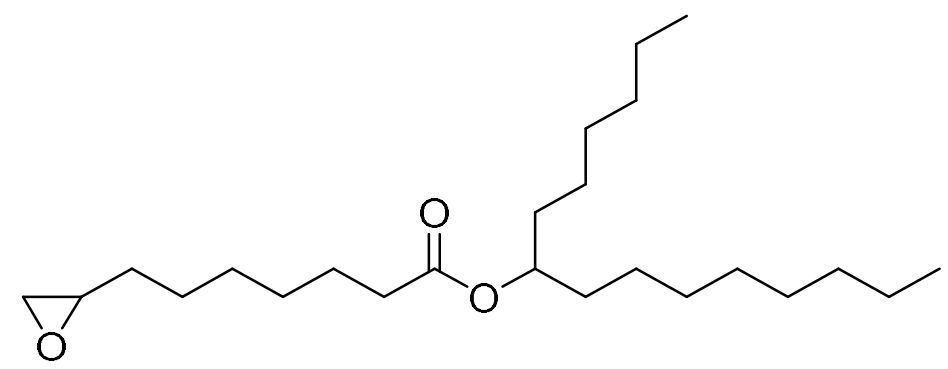

Figure S37. Chemical structure of compound 4o.

^{1}H NMR (500 MHz, $CDCl_3$) δ 4.86 (p, *J* = 7 Hz, 1H), 2.92 – 2.86 (m, 1H), 2.75 – 2.72 (m, 1H), 2.45 (dd, *J* = 5.0, 2.7 Hz, 1H), 2.27 (t, *J* = 7.5 Hz, 2H), 1.65 – 1.44 (m, 10H), 1.40 – 1.21 (m, 24H), 0.87 (t, *J* = 6.9 Hz, 6H).

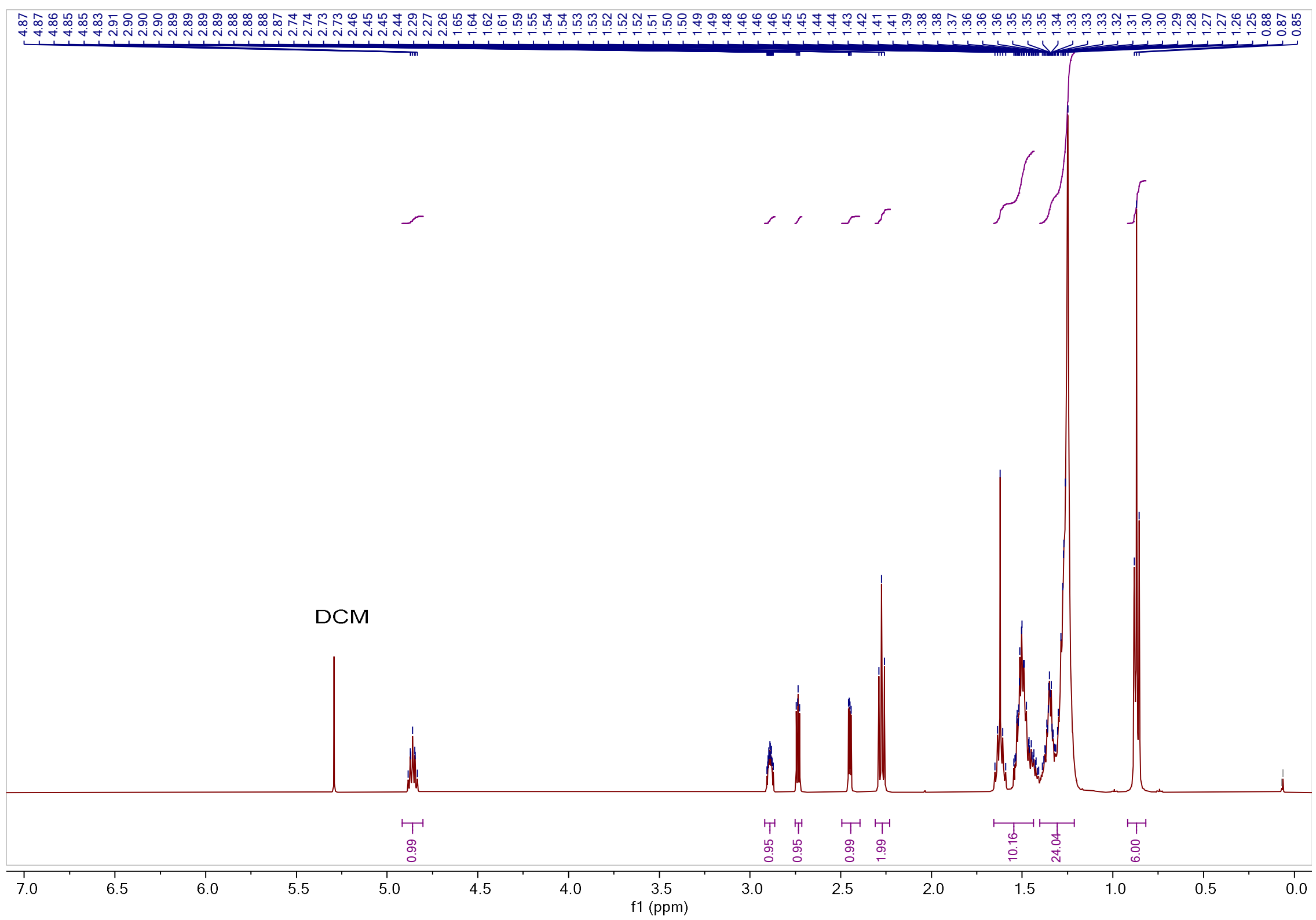

^{1}H NMR spectrum of compound **4o** ($CDCl_3$, 500 MHz)

^{13}C NMR (126 MHz, $CDCl_3$) δ 173.59, 74.15, 52.31, 47.08, 34.66, 34.16, 32.43, 31.87, 31.76, 29.54, 29.51, 29.24, 29.21, 29.11, 29.08, 25.83, 25.33, 25.29, 25.04, 22.67, 22.59, 14.11, 14.07.

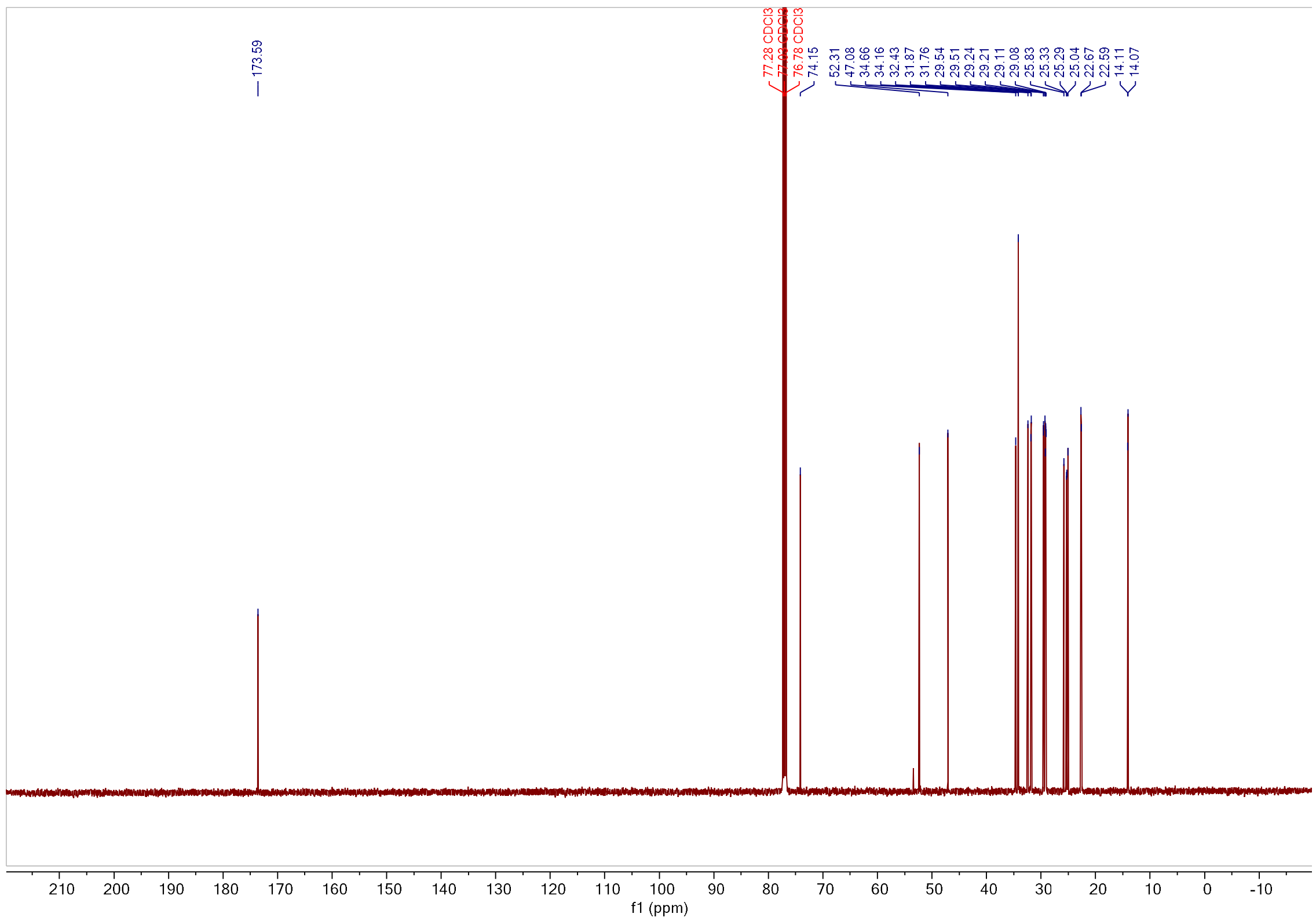

^{13}C-NMR spectrum of compound **4o** ($CDCl_3$, 126 MHz)

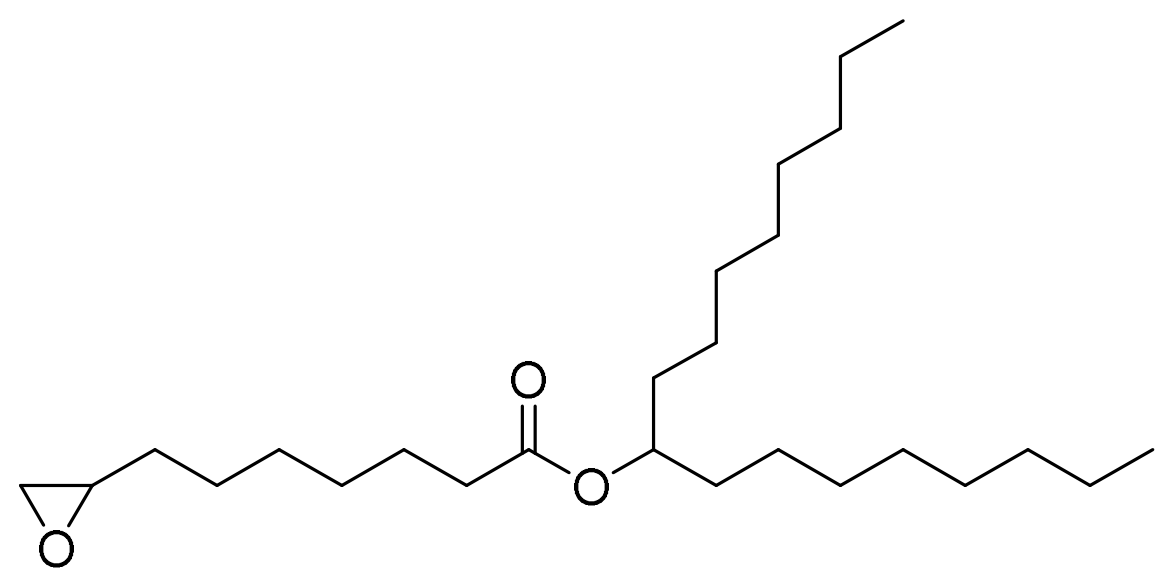

Figure S38. Chemical structure of compound 4p.

^{1}H NMR (500 MHz, $CDCl_3$) δ 4.80 (p, *J* = 6.2 Hz, 1H), 2.86 – 2.79 (m, 1H), 2.69 – 2.66 (m, 1H), 2.39 (dd, *J* = 5.0, 2.7 Hz, 1H), 2.21 (t, *J* = 7.5 Hz, 2H), 1.61-1.52 (m, 2H), 1.50 – 1.35 (m, 8H), 1.32 – 1.14 (m, 28H), 0.81 (t, *J* = 6.9 Hz, 6H).

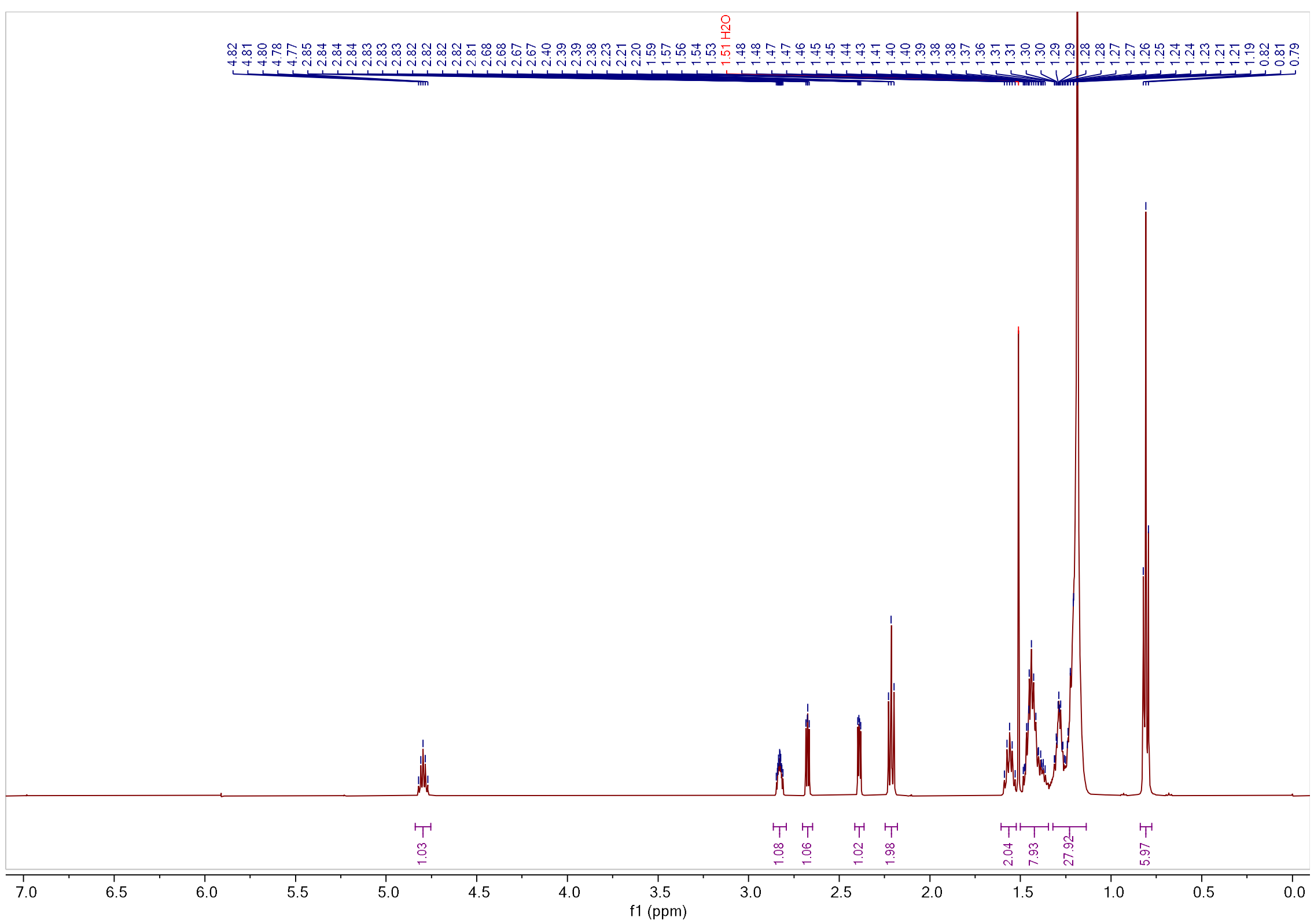

^{1}H NMR spectrum of compound **4p** ($CDCl_3$, 500 MHz)

^{13}C NMR (101 MHz, $CDCl_3$) δ 173.67, 74.24, 52.39, 47.16, 34.74, 34.23, 32.50, 31.94, 29.62, 29.59, 29.32, 29.18, 29.16, 25.91, 25.40, 25.12, 22.74. 14.18.

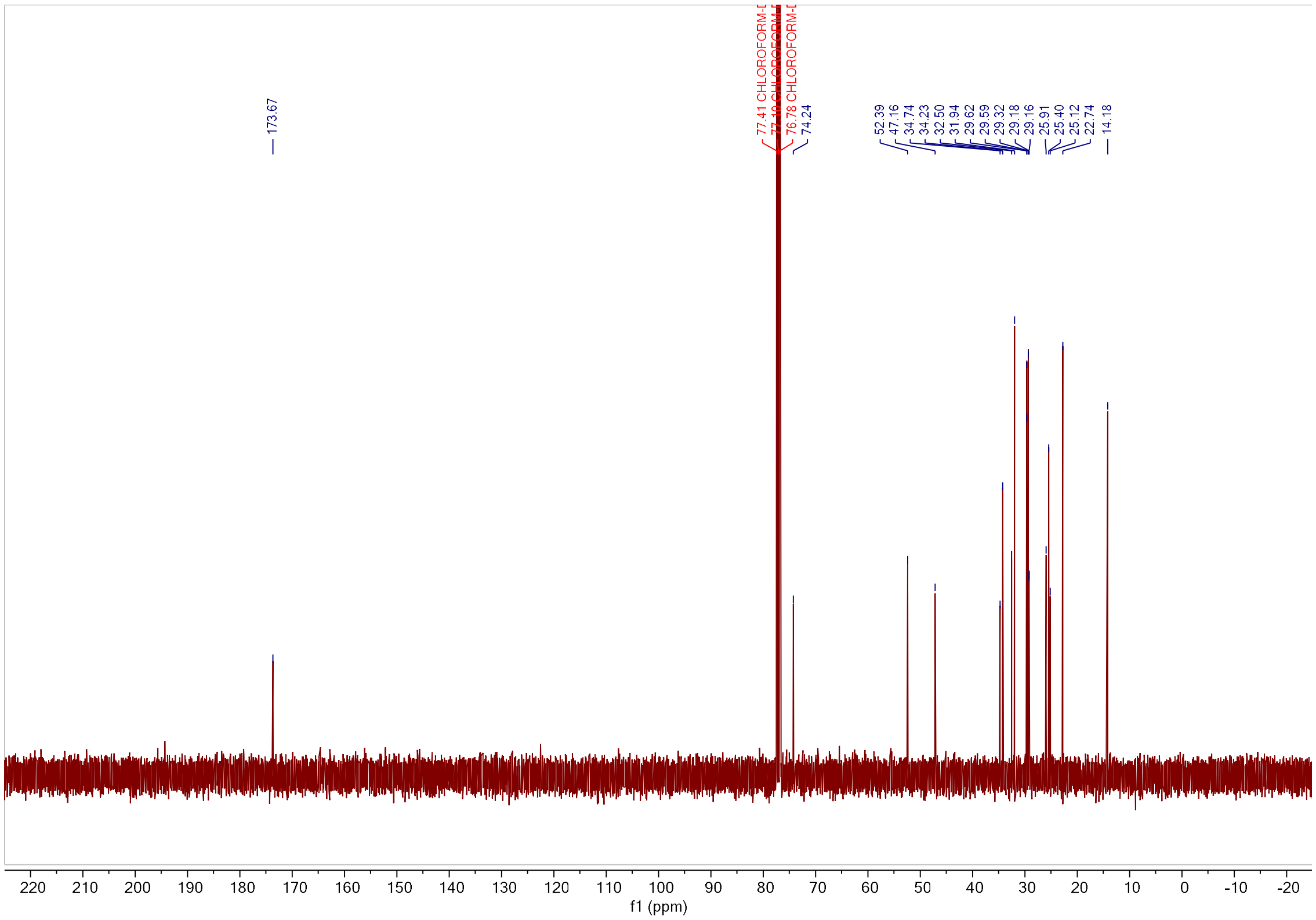

^{13}C-NMR spectrum of compound **4p** ($CDCl_3$, 101 MHz)

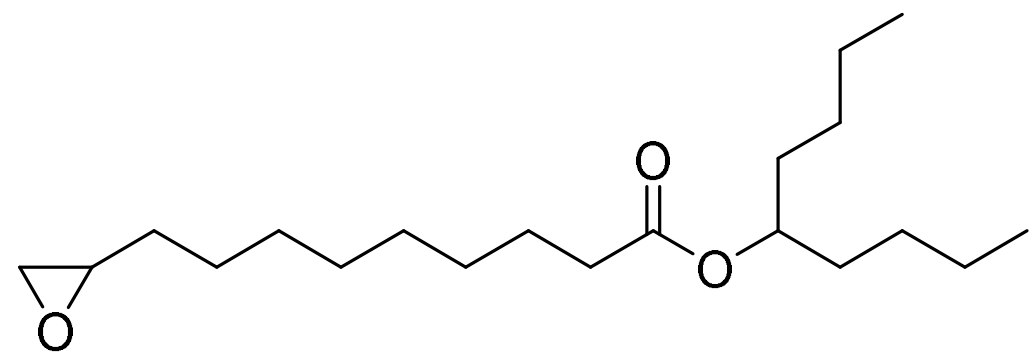

Figure S39. Chemical structure of compound 4q.

^{1}H NMR (500 MHz, $CDCl_3$) δ 4.89 (p, *J* = 6.2 Hz, 1H), 2.92 (m, 1H), 2.78 – 2.74 (m, 1H), 2.48 (dd, *J* = 5.0, 2.7 Hz, 1H), 2.30 (t, *J* = 7.5 Hz, 2H), 1.69-1.62 (m, 2H), 1.57 – 1.41 (m, 8H), 1.38 – 1.24 (m, 16H), 0.91 (t, *J* = 7.0 Hz, 6H).

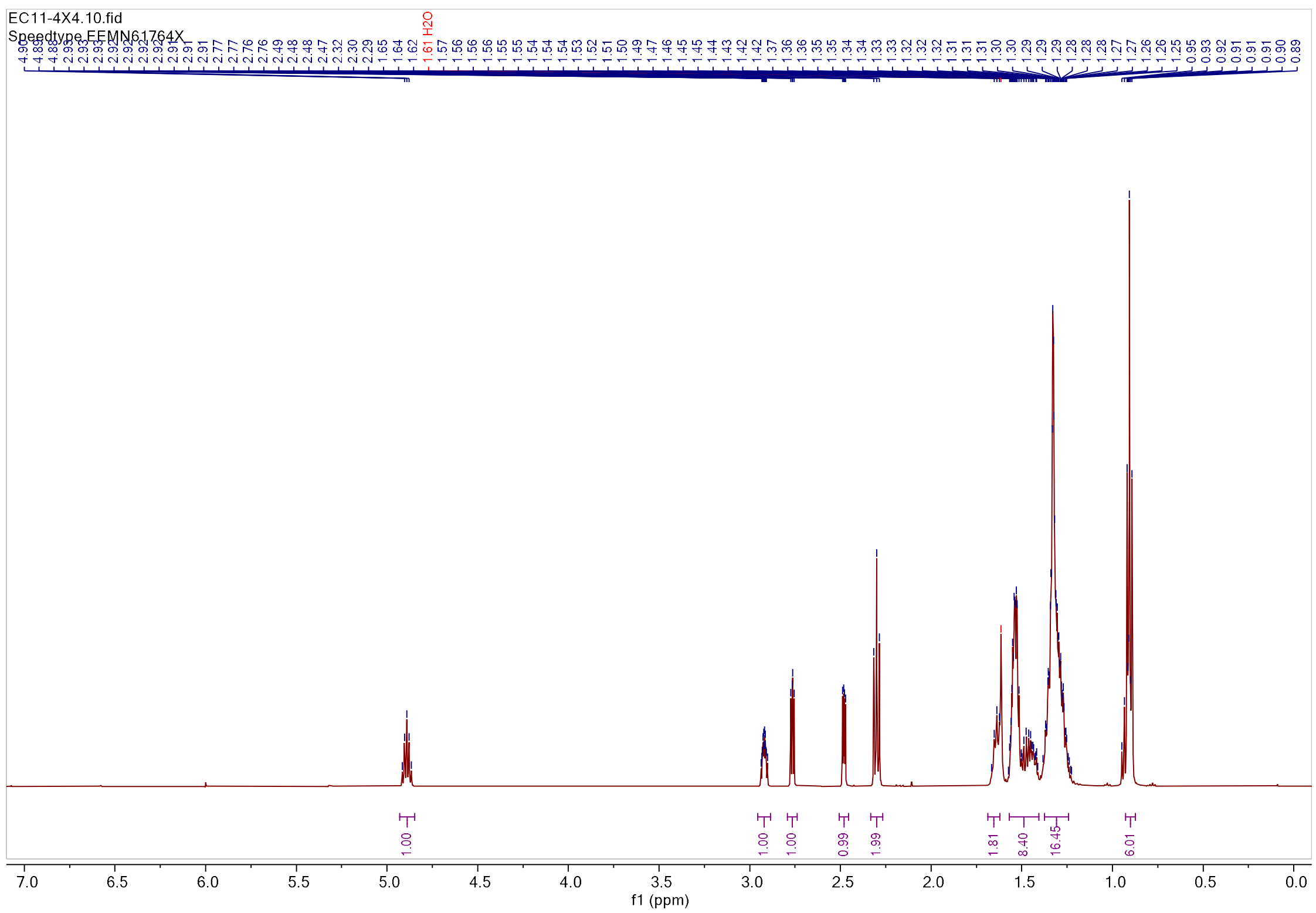

^{1}H NMR spectrum of compound **4q** ($CDCl_3$, 500 MHz)

^{13}C NMR (101 MHz, $CDCl_3$) δ 173.81, 74.15, 52.49, 47.23, 34.79, 33.95, 32.56, 29.47, 29.28, 29.22, 27.59, 26.03, 25.22, 22.69, 14.11.

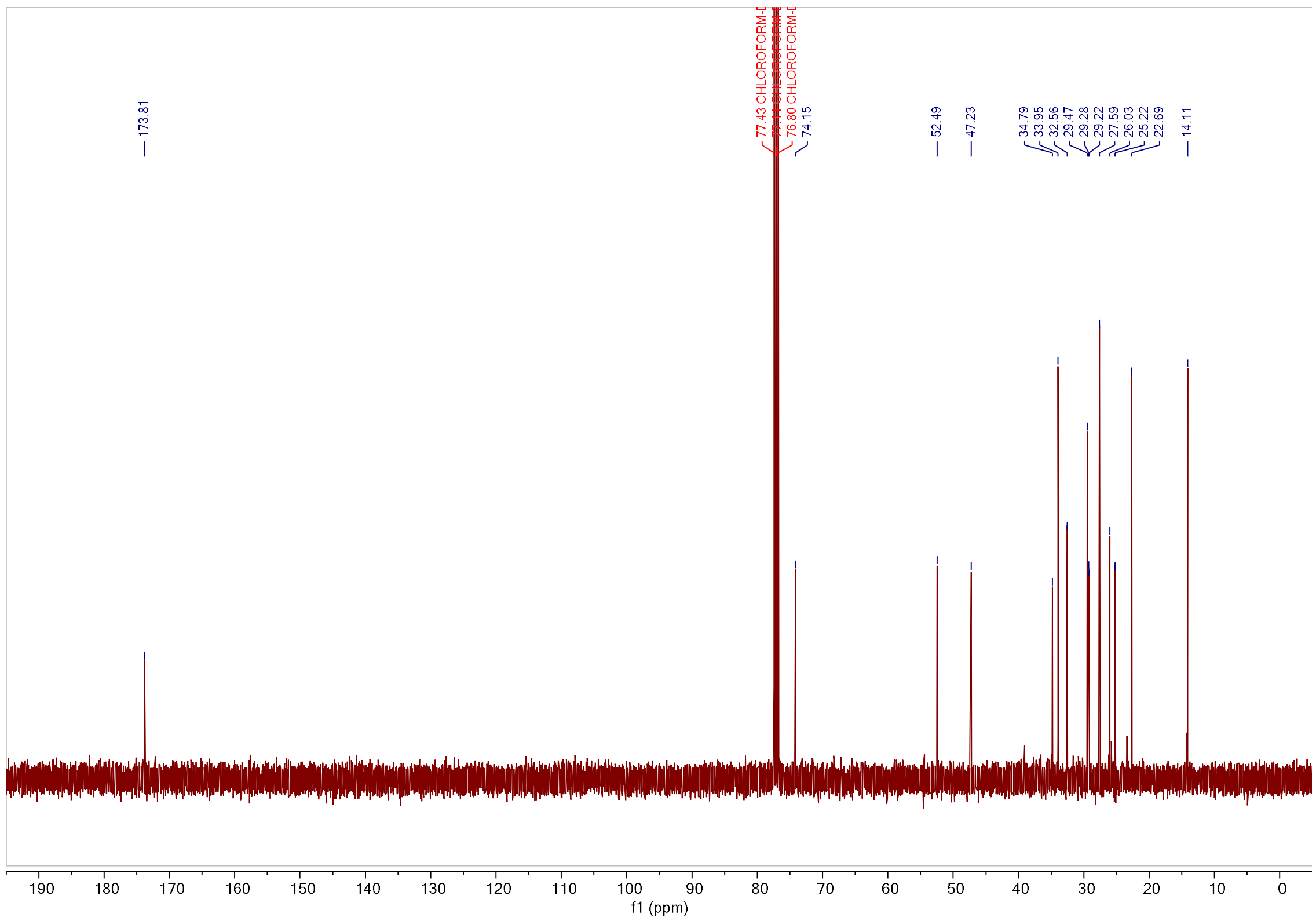

^{13}C-NMR spectrum of compound **4q** ($CDCl_3$, 101 MHz)

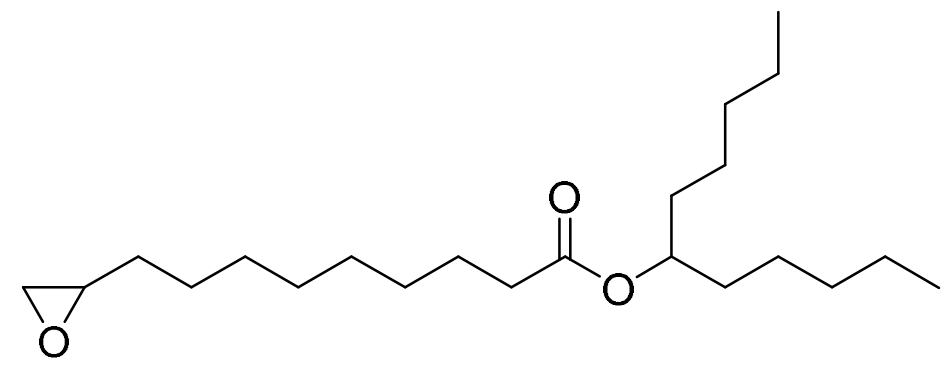

Figure S40. Chemical structure of compound 4r.

^{1}H NMR (500 MHz, $CDCl_3$) δ 4.80 (p, *J* = 7.35, 1H), 2.87 – 2.79 (m, 1H), 2.70 – 2.65 (m, 1H), 2.39 (dd, *J* = 5.0, 2.7 Hz, 1H), 2.21 (t, *J* = 7.5 Hz, 2H), 1.56 – 1.51 (m, 2H), 1.49 – 1.31 (m, 8H), 1.30 – 1.14 (m, 20H), 0.84 – 0.77 (m, 6H).

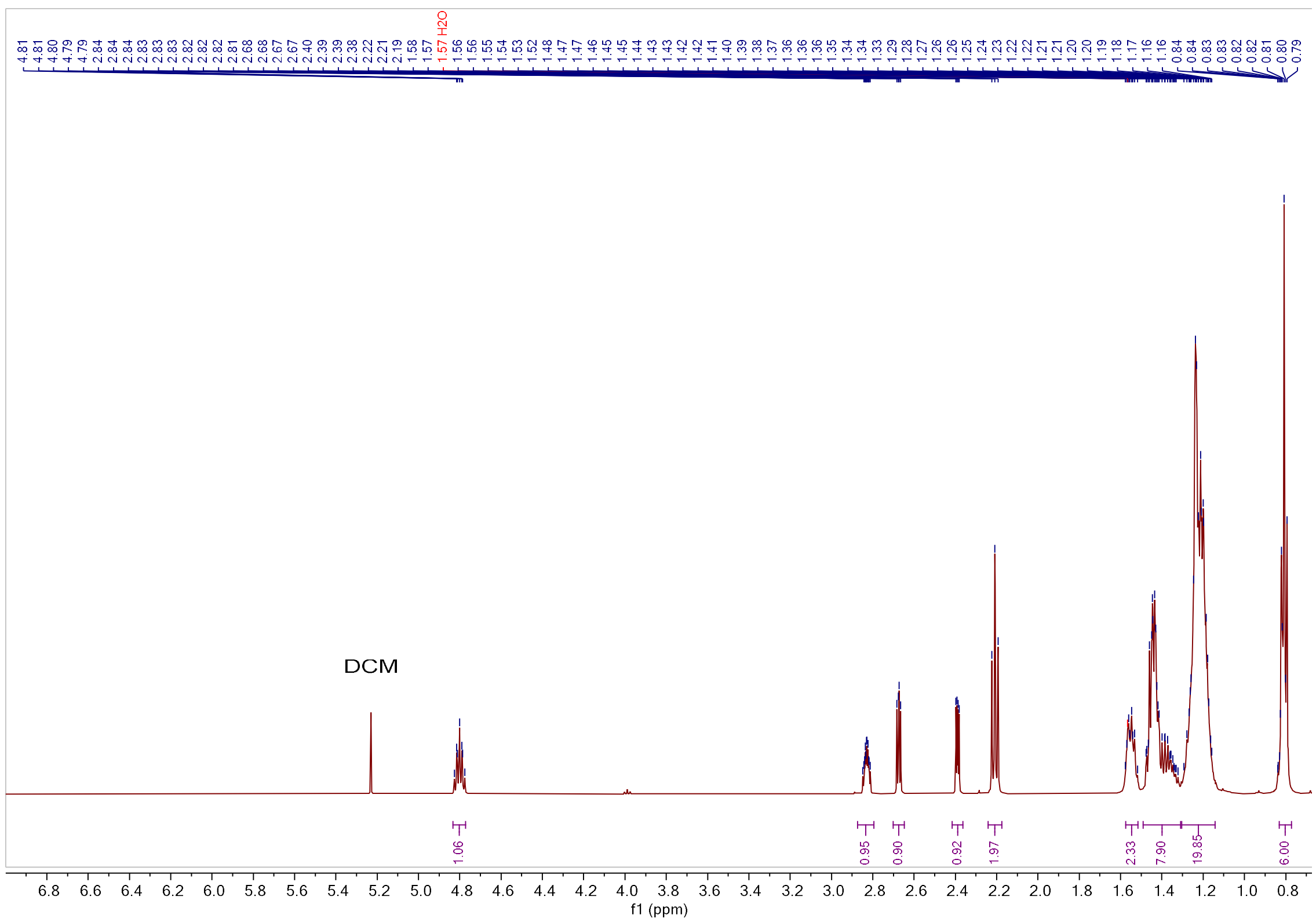

^{1}H NMR spectrum of compound **4r** ($CDCl_3$, 500 MHz)

^{13}C NMR (126 MHz, $CDCl_3$) δ 173.68, 74.09, 52.38, 47.12, 34.72, 34.12, 32.48, 31.72, 29.38, 29.20, 29.14, 25.95, 25.15, 24.99, 22.56, 14.01.

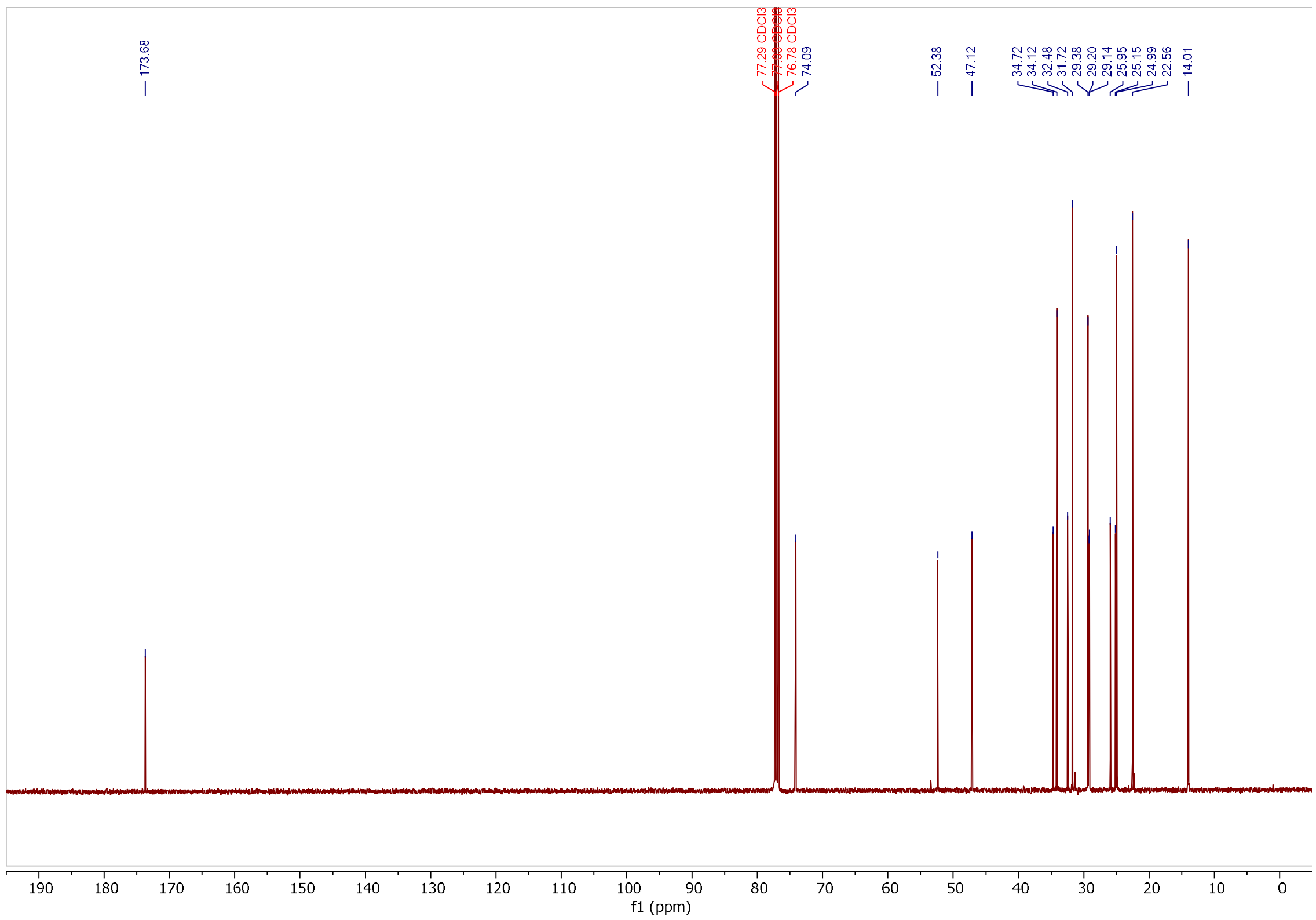

^{13}C-NMR spectrum of compound **4r** ($CDCl_3$, 126 MHz)

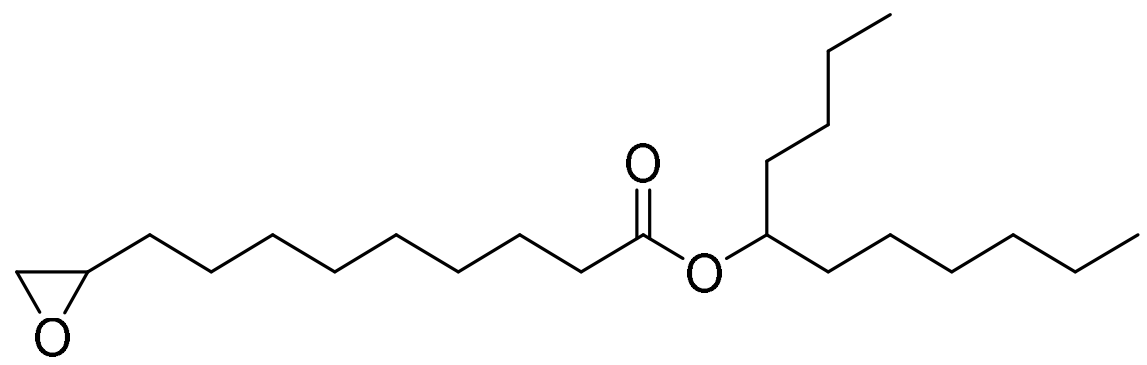

Figure S41. Chemical structure of compound 4s.

^{1}H NMR (500 MHz, CDCl$_3$) δ 4.80 (p, *J* = 6.2 Hz, 1H), 2.87 – 2.80 (m, 1H), 2.69 – 2.66 (m, 1H), 2.39 (dd, *J* = 5.0, 2.8 Hz, 1H), 2.25 – 2.18 (m, 2H), 1.58 – 1.53 (m, 2H), 1.45 – 1.35 (m, 6H), 1.31 – 1.15 (m, 22H), 0.81 (t, *J* = 7.0 Hz, 6H).

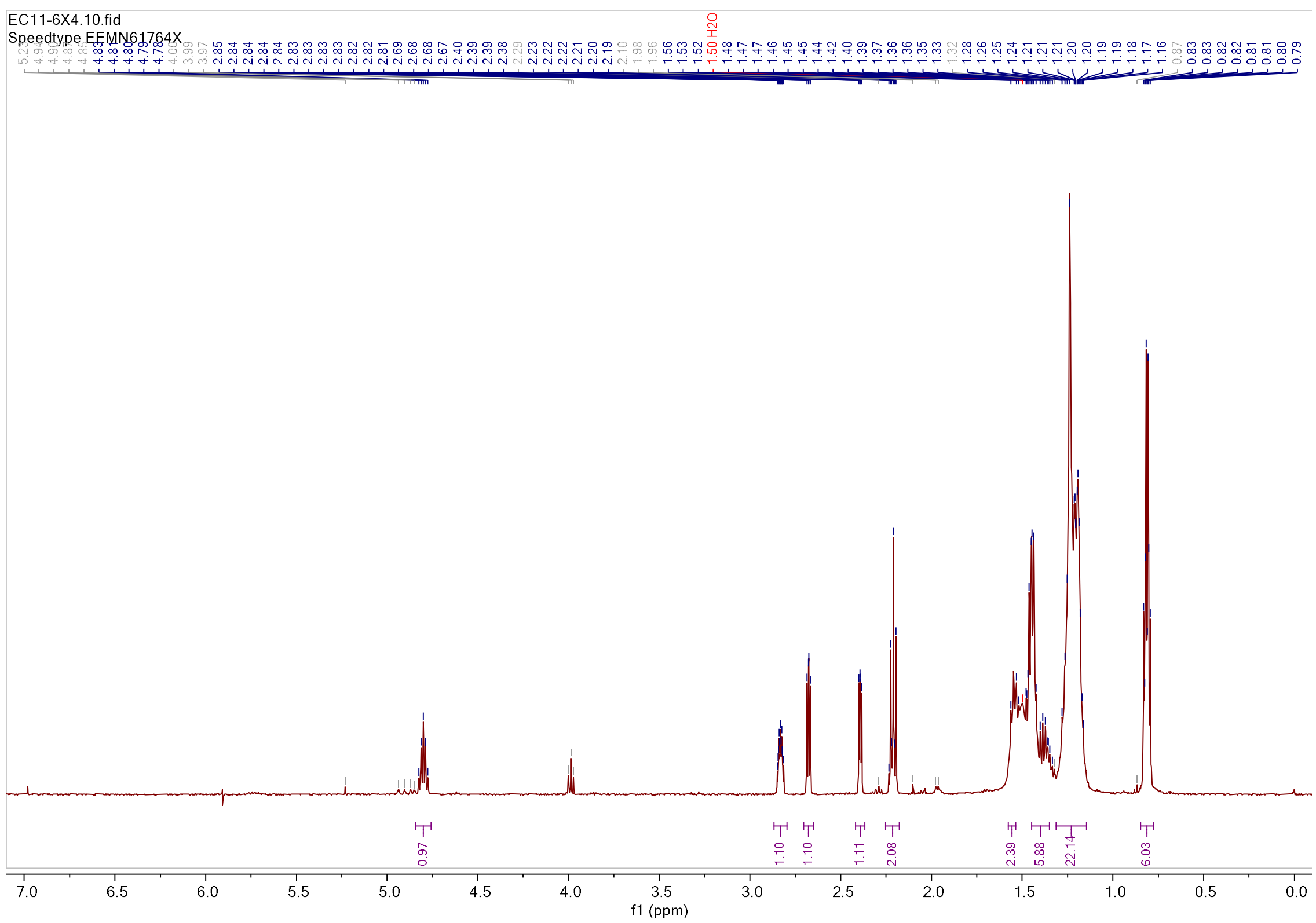

^{1}H NMR spectrum of compound **4s** (CDCl$_3$, 500 MHz)

^{13}C NMR (126 MHz, $CDCl_3$) δ 173.69, 74.09, 52.39, 47.13, 34.72, 34.18, 33.87, 32.49, 31.77, 29.39, 29.22, 29.20, 29.15, 27.51, 25.95, 25.30, 25.15, 22.62, 22.59, 14.21, 14.08, 14.02.

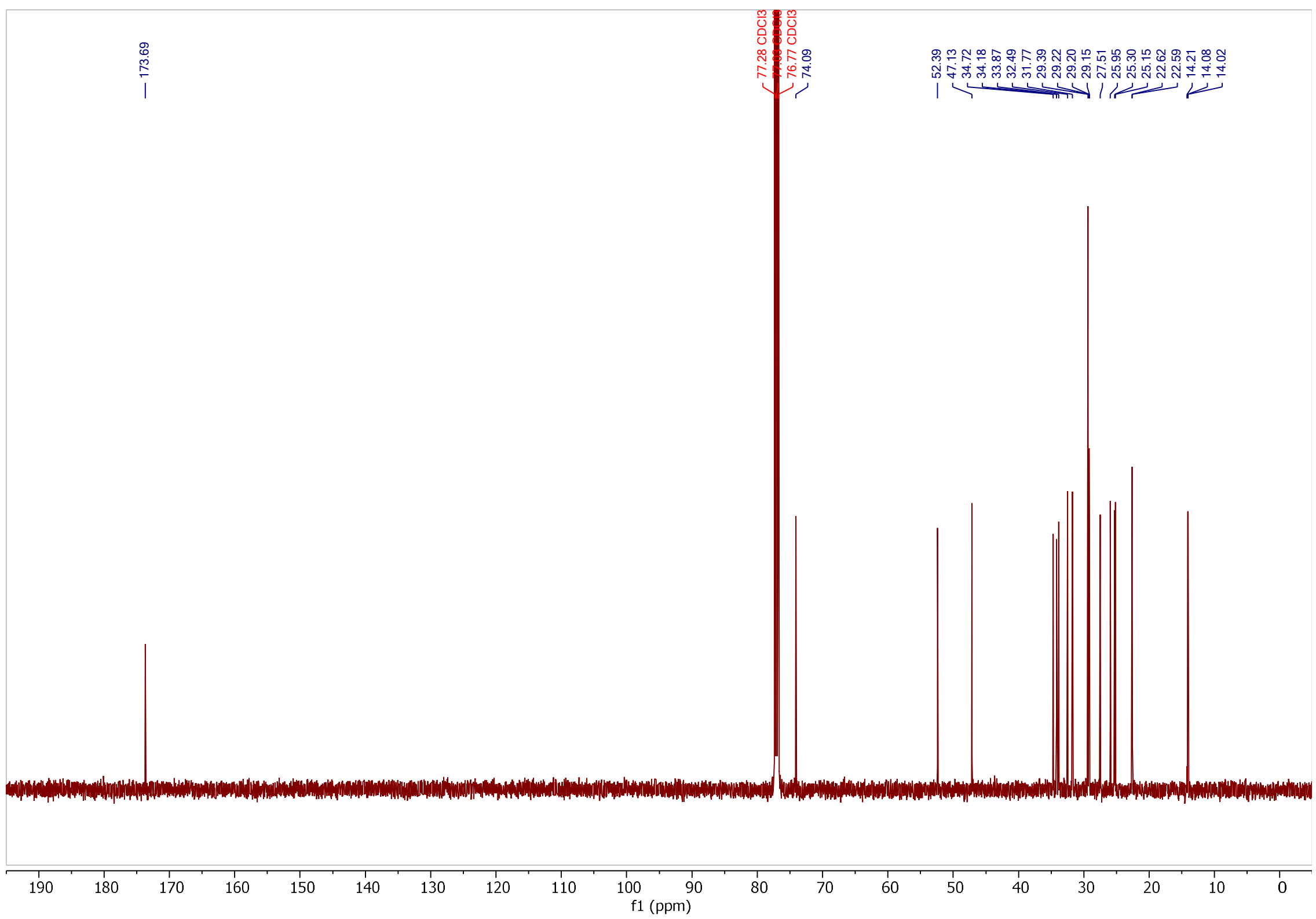

^{13}C-NMR spectrum of compound **4s** ($CDCl_3$, 126 MHz)

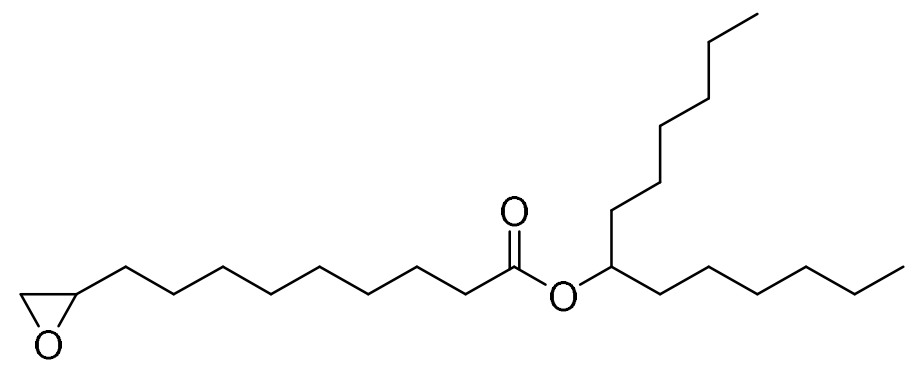

Figure S42. Chemical structure of compound 4t.

^{1}H NMR (500 MHz, $CDCl_3$) δ 4.86 (p, *J* = 6.2 Hz, 1H), 2.92 – 2.86 (m, 1H), 2.75 – 2.72 (m, 1H), 2.45 (dd, *J* = 5.1, 2.7 Hz, 1H), 2.27 (t, *J* = 7.5 Hz, 2H), 1.65 – 1.58 (m, 2H), 1.54 – 1.46 (m, 6H), 1.46 – 1.38 (m, 2H), 1.37 – 1.20 (m, 24H), 0.87 (t, *J* = 6.7Hz, 6H).

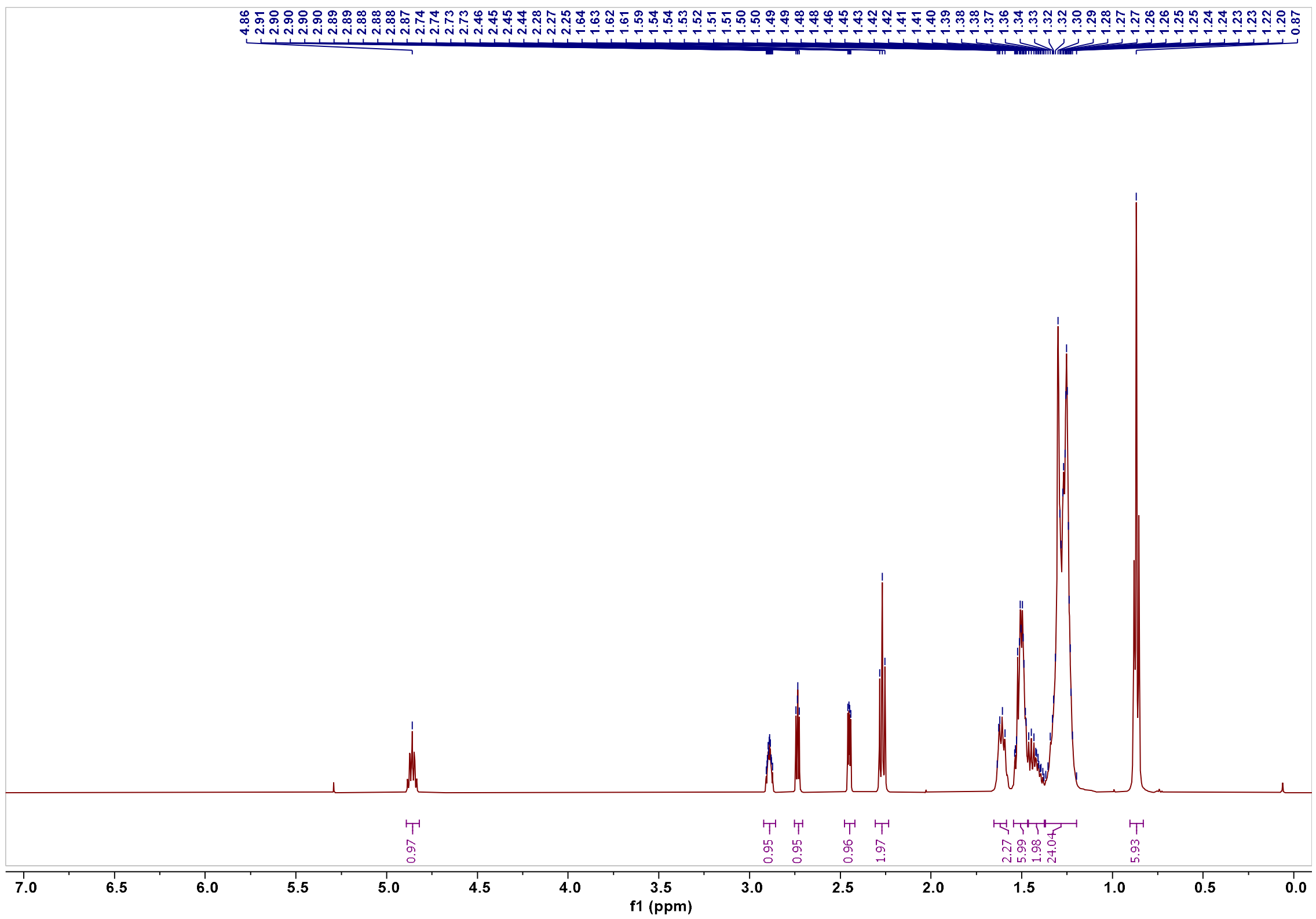

^{1}H NMR spectrum of compound **4t** ($CDCl_3$, 500 MHz)

^{13}C NMR (126 MHz, $CDCl_3$) δ 173.68, 74.10, 52.38, 47.12, 34.72, 34.17, 32.48, 31.77, 31.63, 29.39, 29.21, 29.15, 25.95, 25.29, 25.15, 22.59, 14.07.

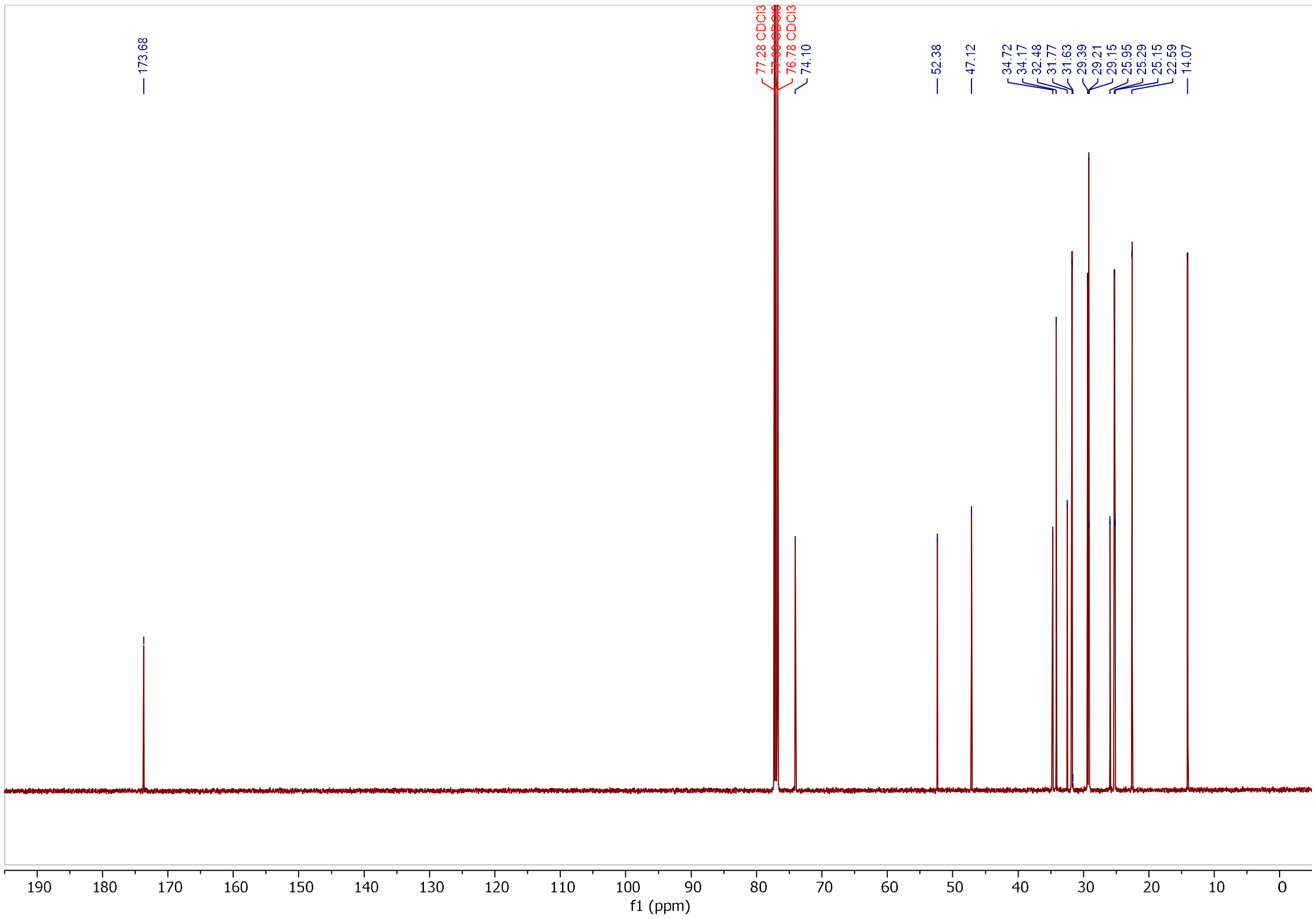

^{13}C-NMR spectrum of compound **4t** ($CDCl_3$, 126 MHz)

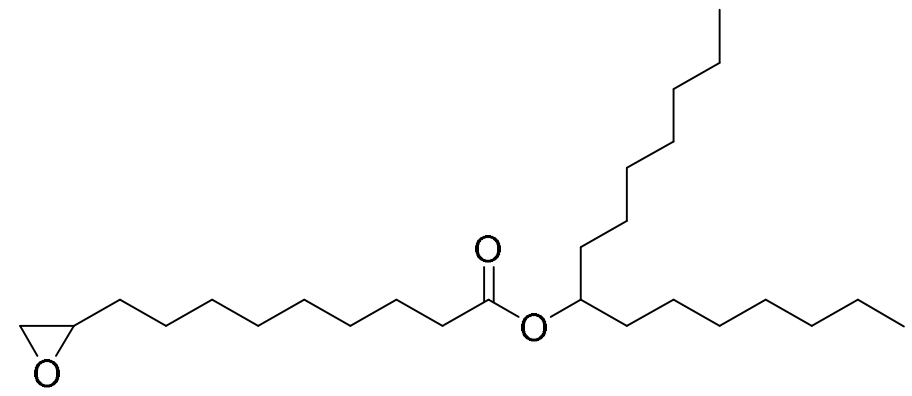

Figure S43. Chemical structure of compound 4u.

^{1}H NMR (500 MHz, $CDCl_3$) δ 4.85 – 4.75 (m, 1H), 2.85-2.81 (m, 1H), 2.69 – 2.66 (m, 1H), 2.39 (dd, *J* = 5.1, 2.7 Hz, 1H), 2.21 (t, *J* = 7.5 Hz, 2H), 1.55 (t, *J* = 7.2 Hz, 2H), 1.48 – 1.31 (m, 8H), 1.30 – 1.14 (m, 28H), 0.81 (t, *J* = 6.9 Hz, 6H).

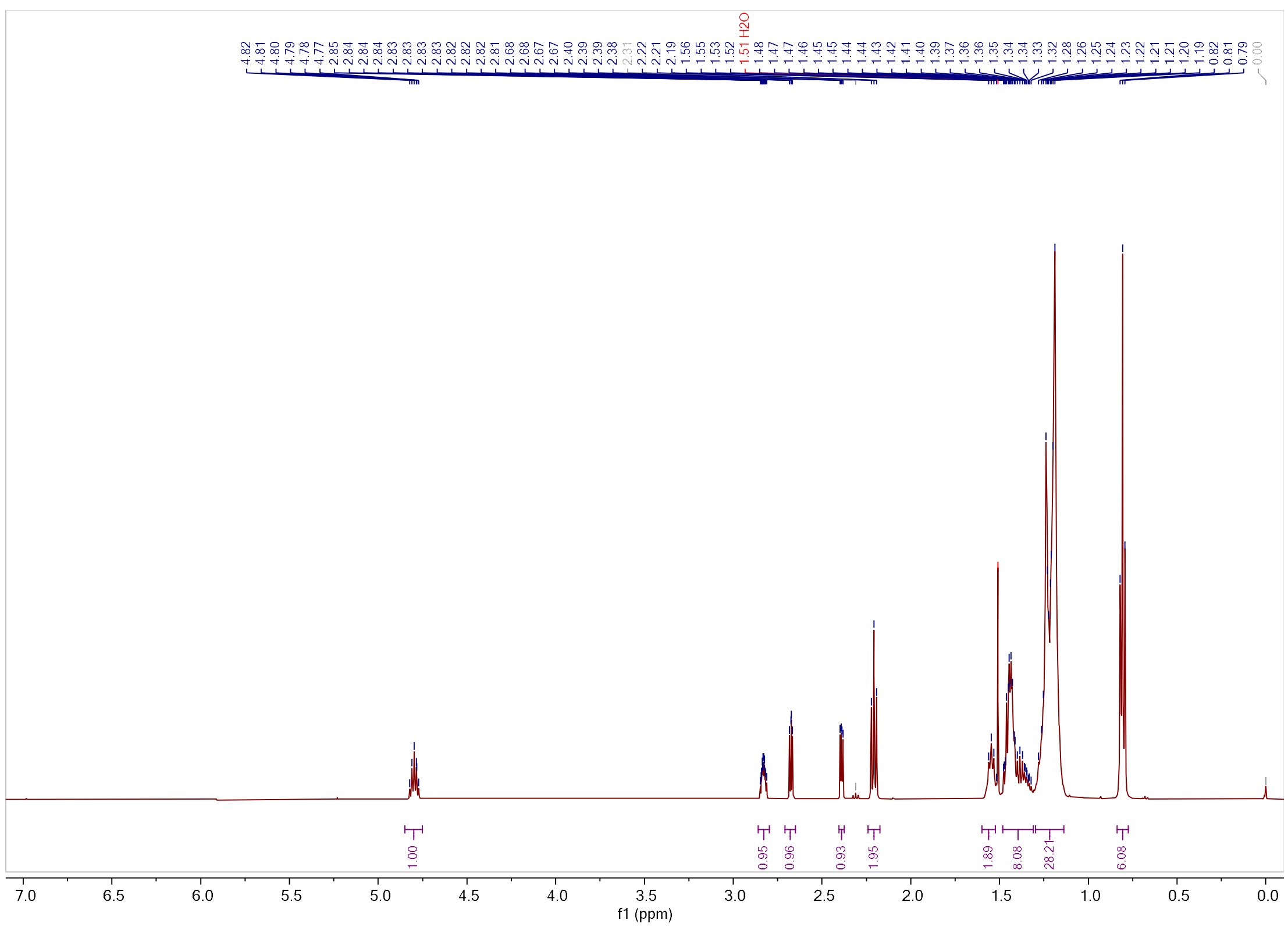

^{1}H NMR spectrum of compound **4u** ($CDCl_3$, 500 MHz)

^{13}C NMR (101 MHz, $CDCl_3$) δ 173.79, 74.19, 52.48, 47.23, 34.81, 34.25, 32.57, 31.88, 29.59, 29.48, 29.31, 29.30, 29.23, 26.04, 25.42, 25.24, 22.74, 14.19.

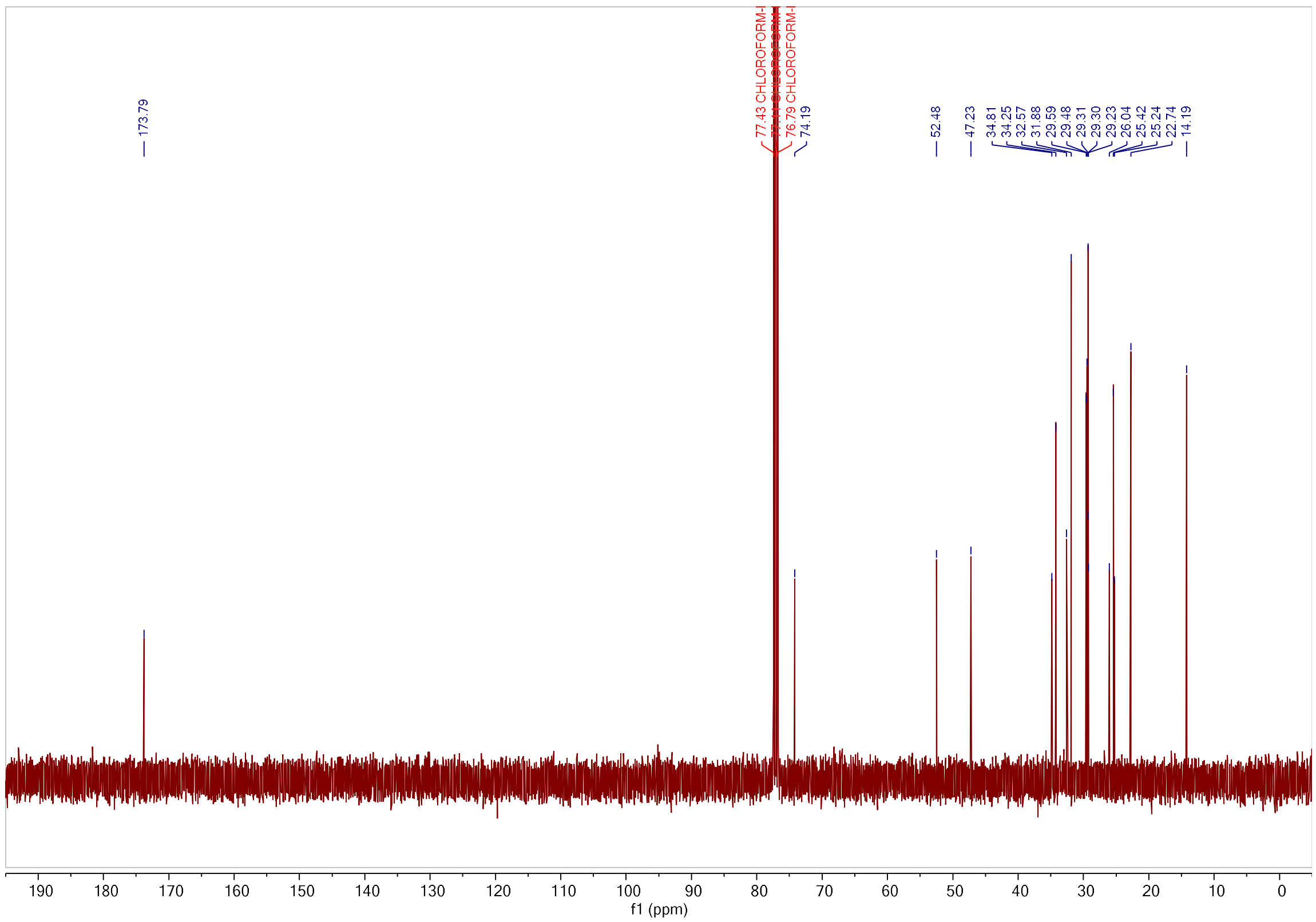

^{13}C-NMR spectrum of compound **4u** ($CDCl_3$, 101 MHz)

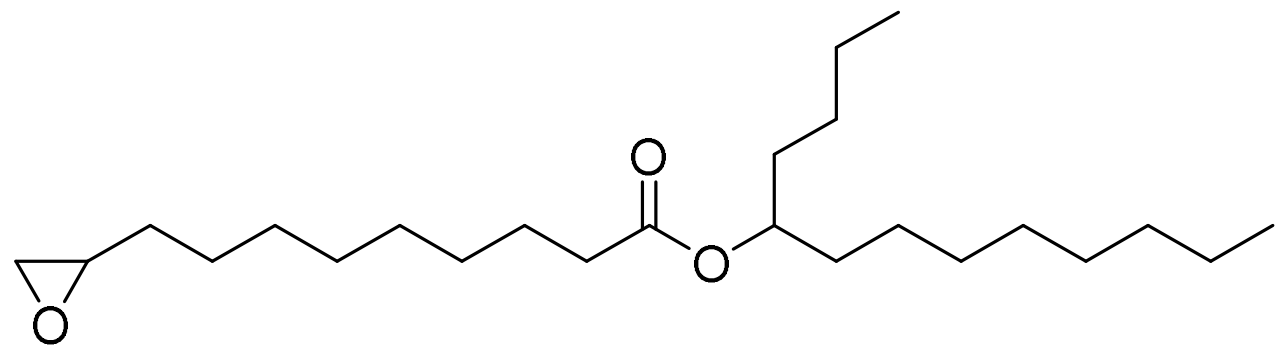

Figure S44. Chemical structure of compound 4v.

^{1}H NMR (500 MHz, $CDCl_3$) δ 4.80 (p, *J* = 6.3 Hz, 1H), 2.83 (m, 1H), 2.69 – 2.66 (m, 1H), 2.39 (dd, *J* = 5.0, 2.7 Hz, 1H), 2.21 (t, *J* = 7.5 Hz, 2H), 1.59 – 1.52 (m, 2H), 1.48 – 1.32 (m, 8H), 1.29 – 1.14 (m, 24H), 0.83 – 0.79 (m, 6H).

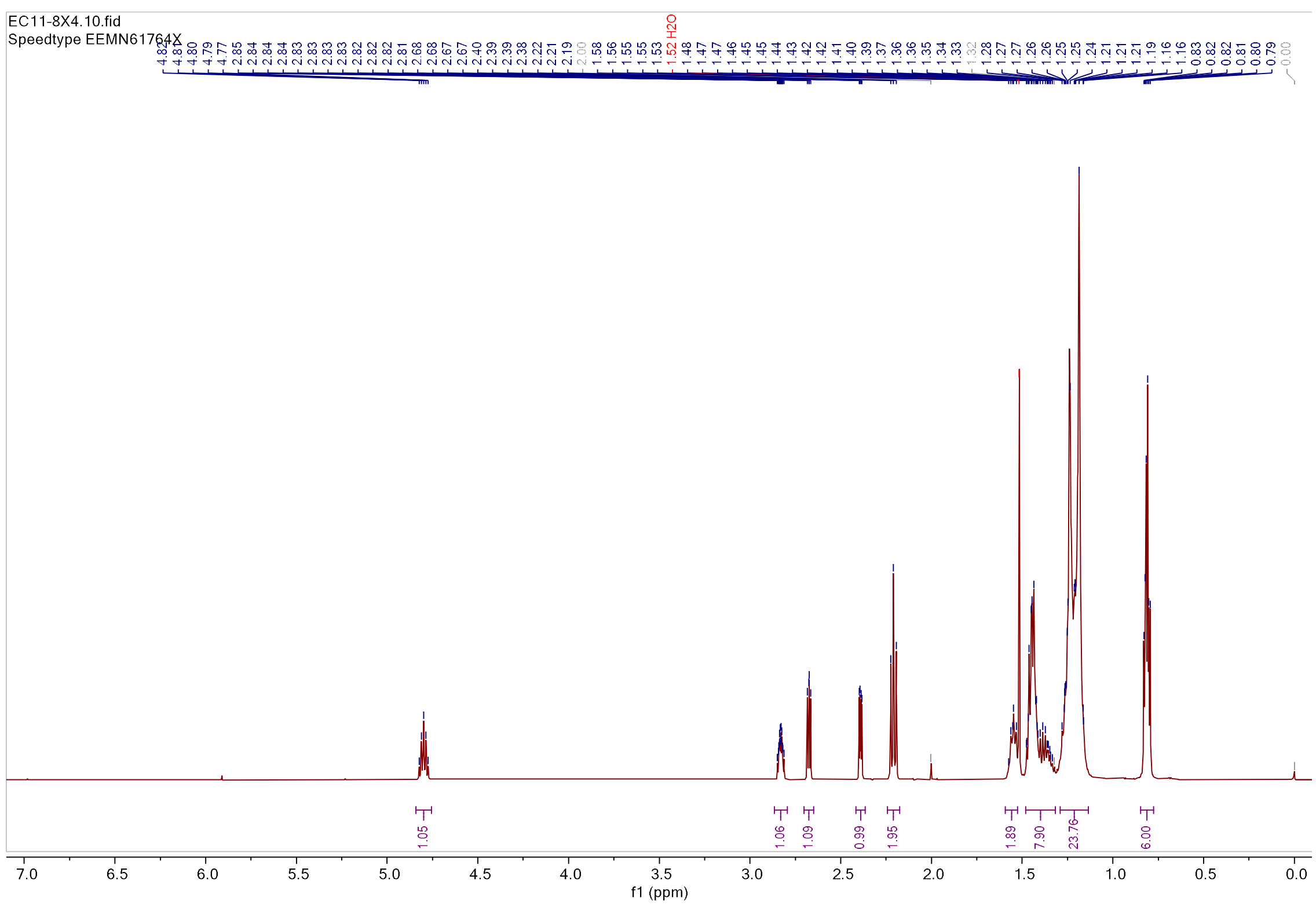

^{1}H NMR spectrum of compound **4v** ($CDCl_3$, 500 MHz)

^{13}C NMR (101 MHz, $CDCl_3$) δ 173.77, 74.16, 52.45, 47.19, 34.80, 34.25, 33.94, 32.56, 31.94, 29.62, 29.59, 29.46, 29.32, 29.27, 29.22, 27.58, 26.02, 25.40, 25.22, 22.74, 22.68, 14.18, 14.08.

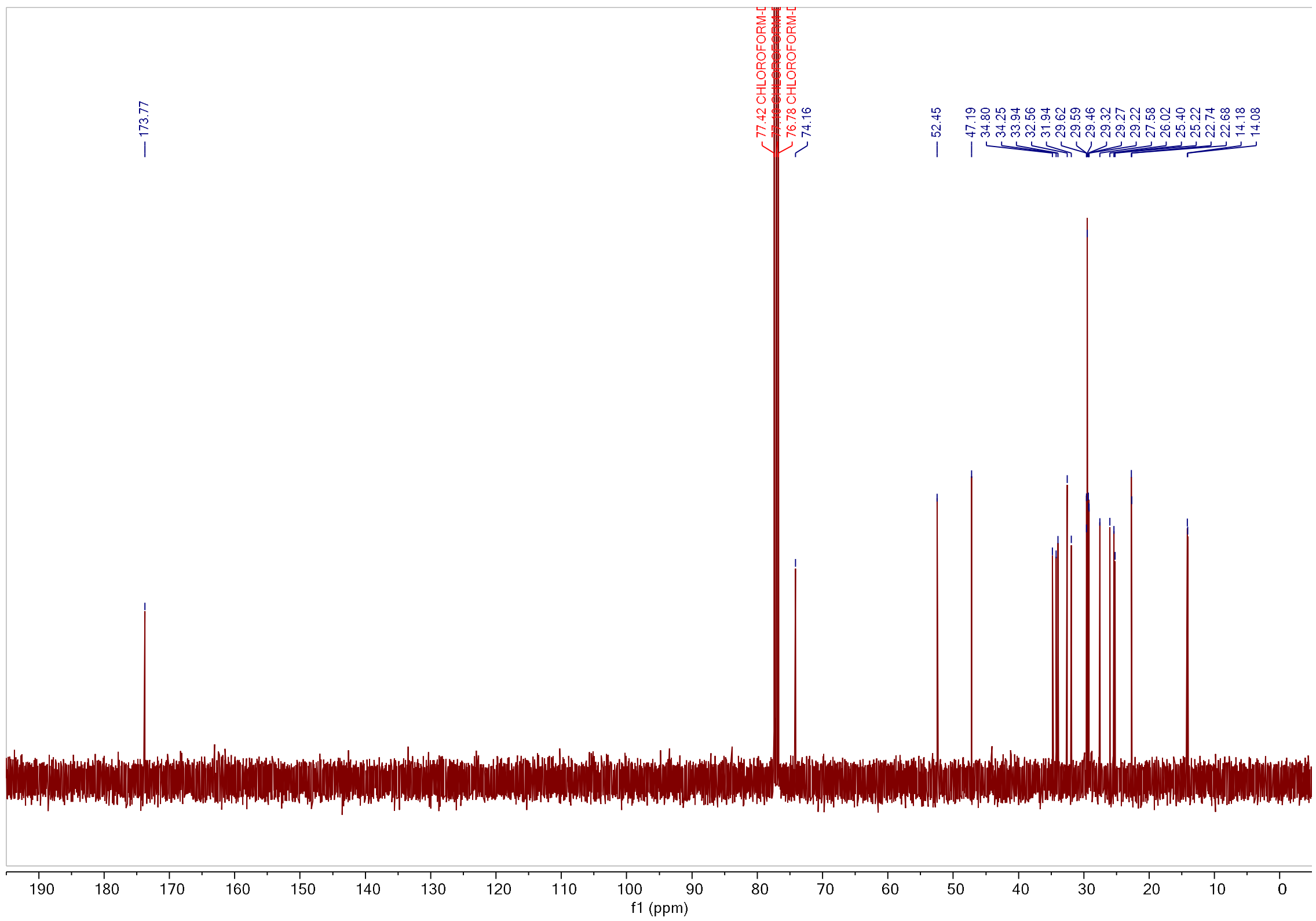

^{13}C-NMR spectrum of compound **4v** ($CDCl_3$, 101 MHz)

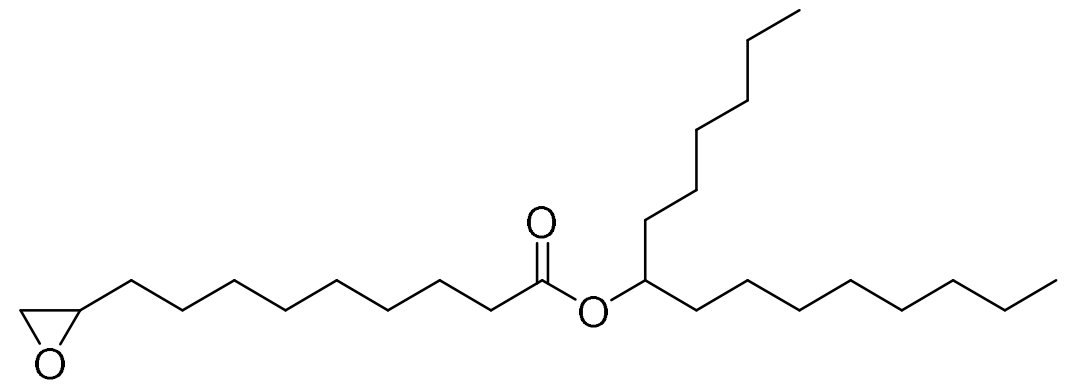

Figure S45. Chemical structure of compound 4w.

^{1}H NMR (500 MHz, $CDCl_3$) δ 4.80 (p, *J* = 6.3 Hz, 1H), 2.85 – 2.81 (m, *J* = 5.6, 2.8 Hz, 1H), 2.69 – 2.66 (m, 1H), 2.39 (dd, *J* = 5.0, 2.7 Hz, 1H), 2.21 (t, *J* = 7.5 Hz, 2H), 1.58 – 1.52 (m, 2H), 1.48 – 1.31 (m, 8H), 1.29 – 1.13 (m, 28H), 0.81 (t, *J* = 6.8 Hz, 6H).

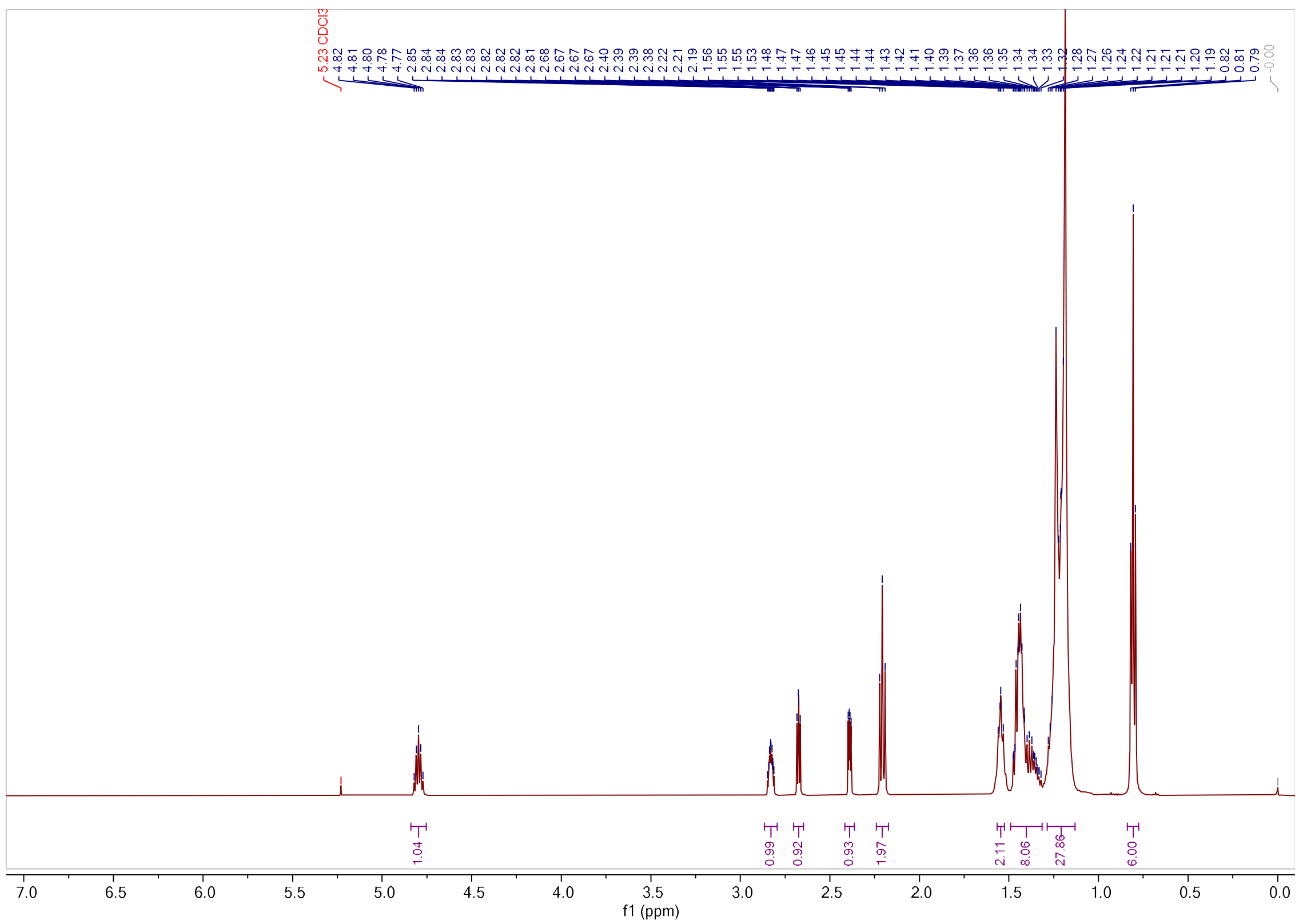

^{1}H NMR spectrum of compound **4w** ($CDCl_3$, 500 MHz)

^{13}C NMR (126 MHz, $CDCl_3$) δ 173.68, 74.11, 52.38, 47.12, 34.73, 34.17, 32.49, 31.87, 31.77, 29.55, 29.52, 29.39, 29.25, 29.21, 29.15, 25.95, 25.33, 25.29, 25.16, 22.67, 22.59, 14.11, 14.07.

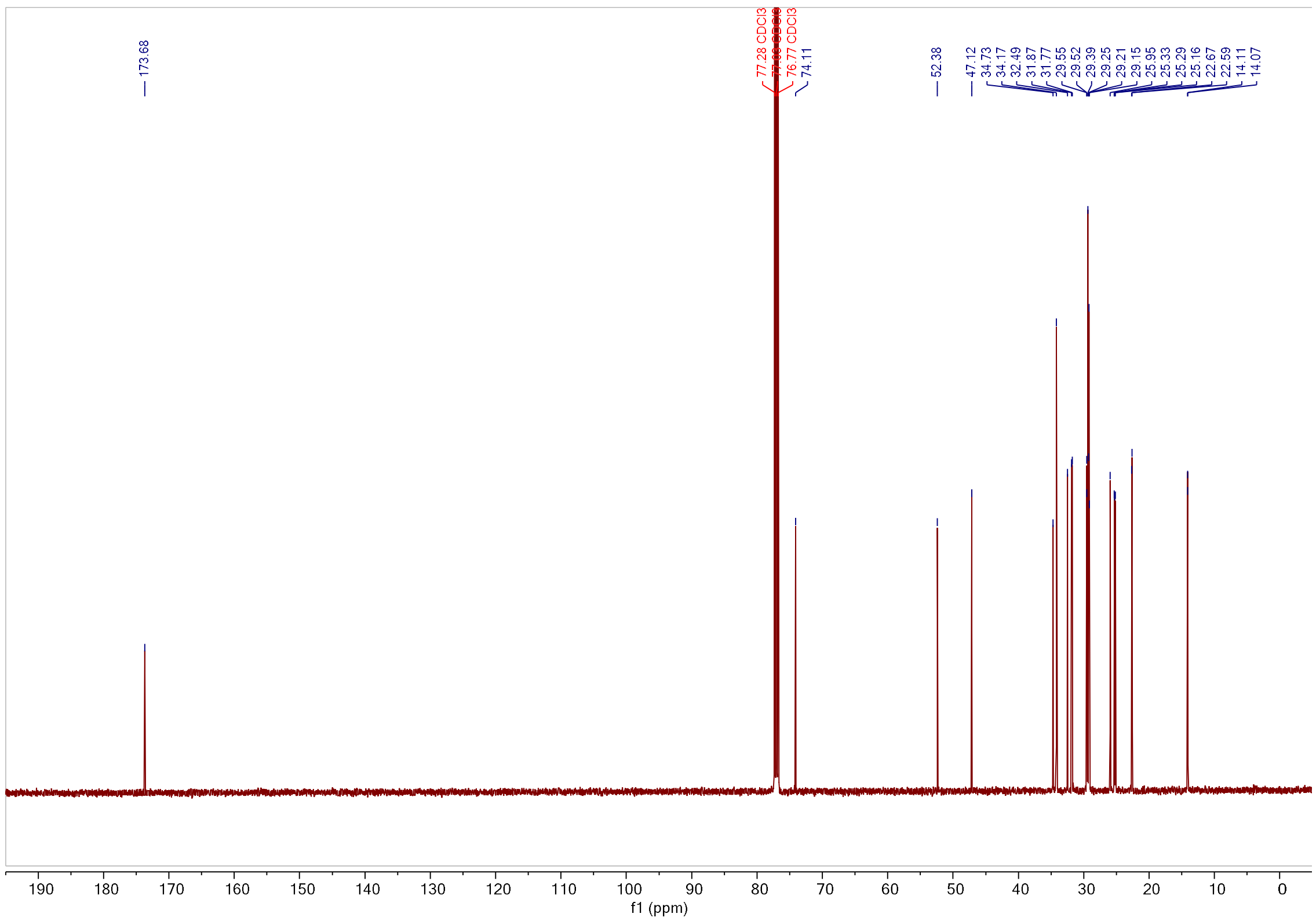

^{13}C-NMR spectrum of compound **4w** ($CDCl_3$, 126 MHz)

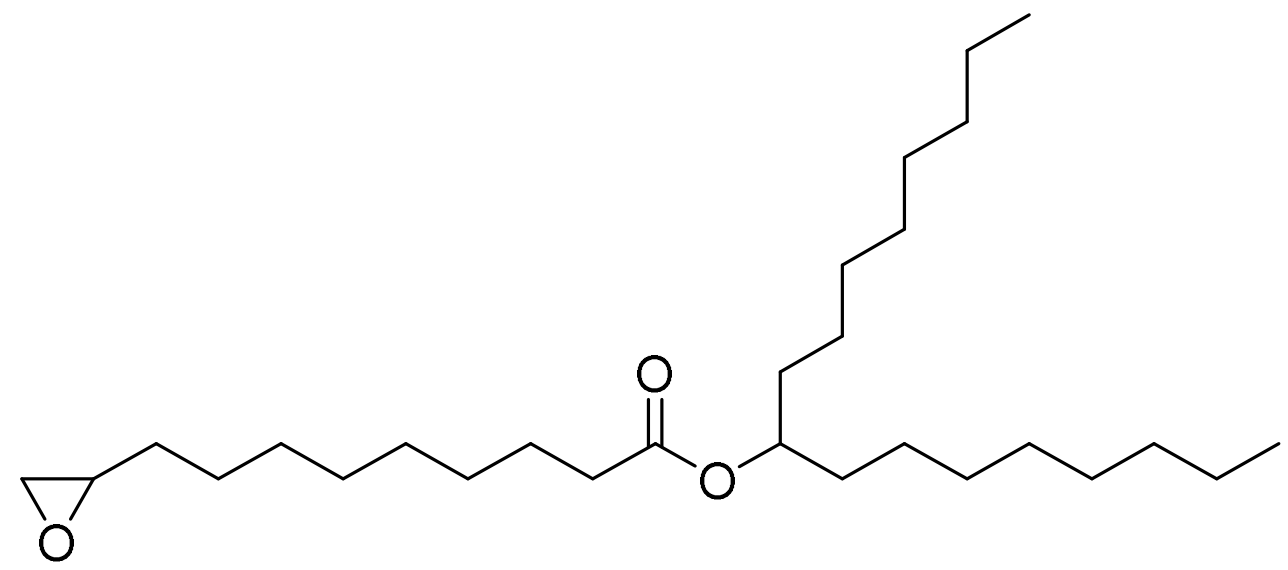

Figure S46. Chemical structure of compound 4x.

^{1}H NMR (500 MHz, $CDCl_3$) δ 4.80 (p, *J* = 6.2 Hz, 1H), 2.87 – 2.79 (m, 1H), 2.69 – 2.66 (m, 1H), 2.39 (dd, *J* = 5.1, 2.7 Hz, 1H), 2.21 (t, *J* = 7.5 Hz, 2H), 1.59 – 1.52 (m, 2H), 1.48 – 1.31 (m, 8H), 1.29 – 1.07 (m, 32H), 0.81 (t, *J* = 6.9 Hz, 6H).

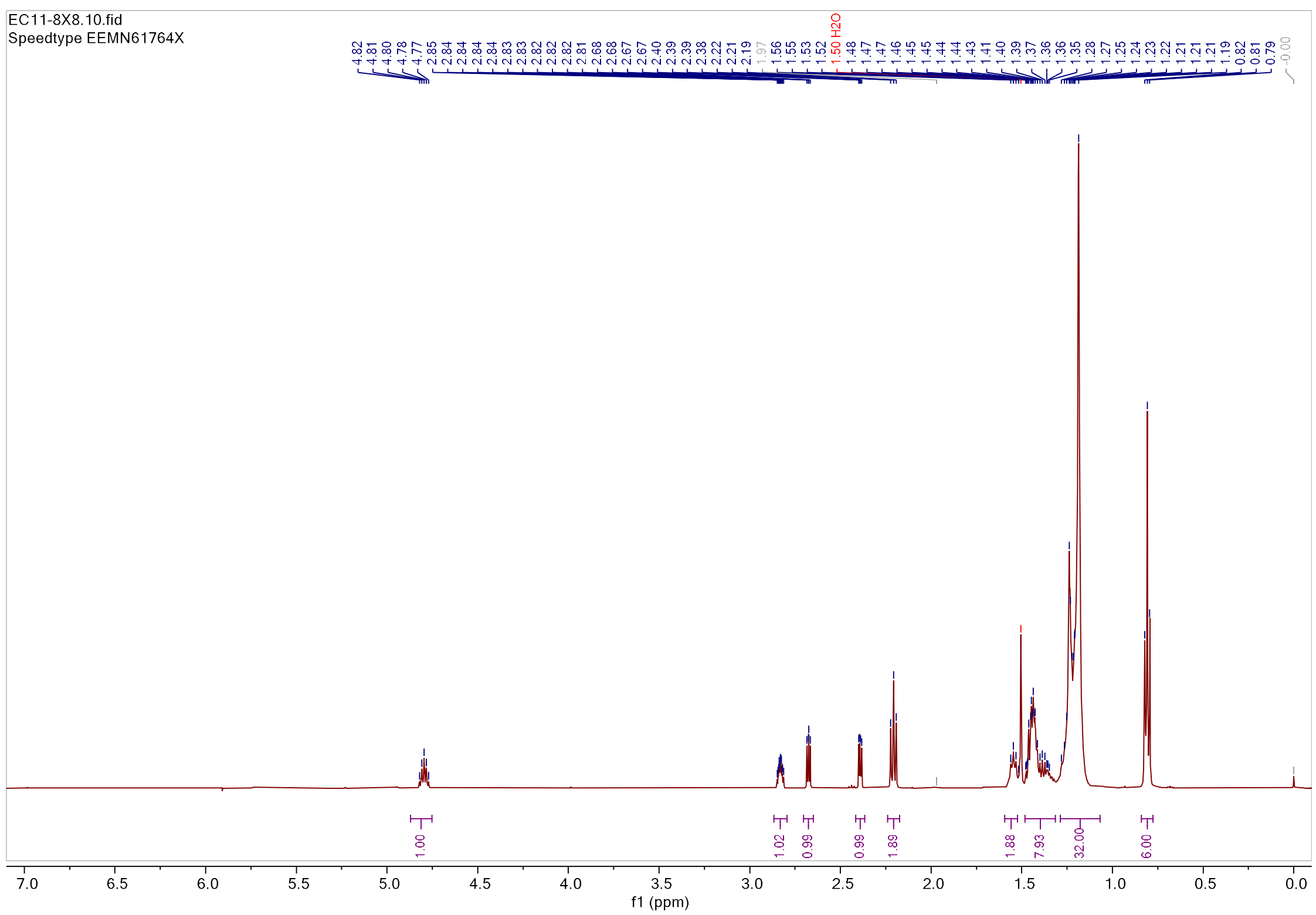

^{1}H NMR spectrum of compound **4x** ($CDCl_3$, 500 MHz)

^{13}C NMR (101 MHz, $CDCl_3$) δ 173.80, 74.20, 52.49, 47.23, 34.81, 34.24, 32.58 31.96, 29.63, 29.60, 29.48, 29.33, 29.30, 29.24, 26.04, 25.41, 25.24, 22.76, 14.20.

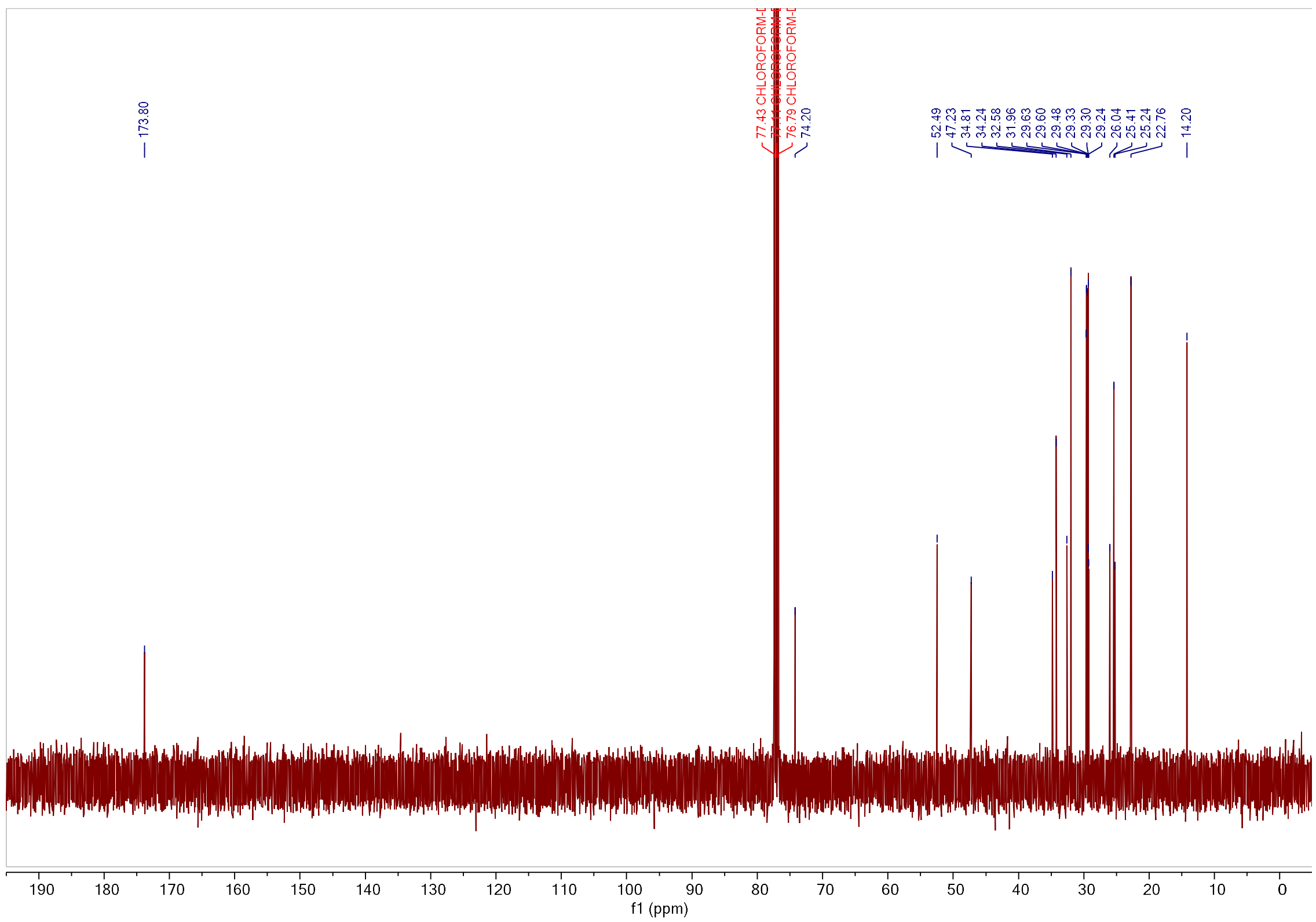

^{13}C-NMR spectrum of compound **4x** ($CDCl_3$, 101 MHz)

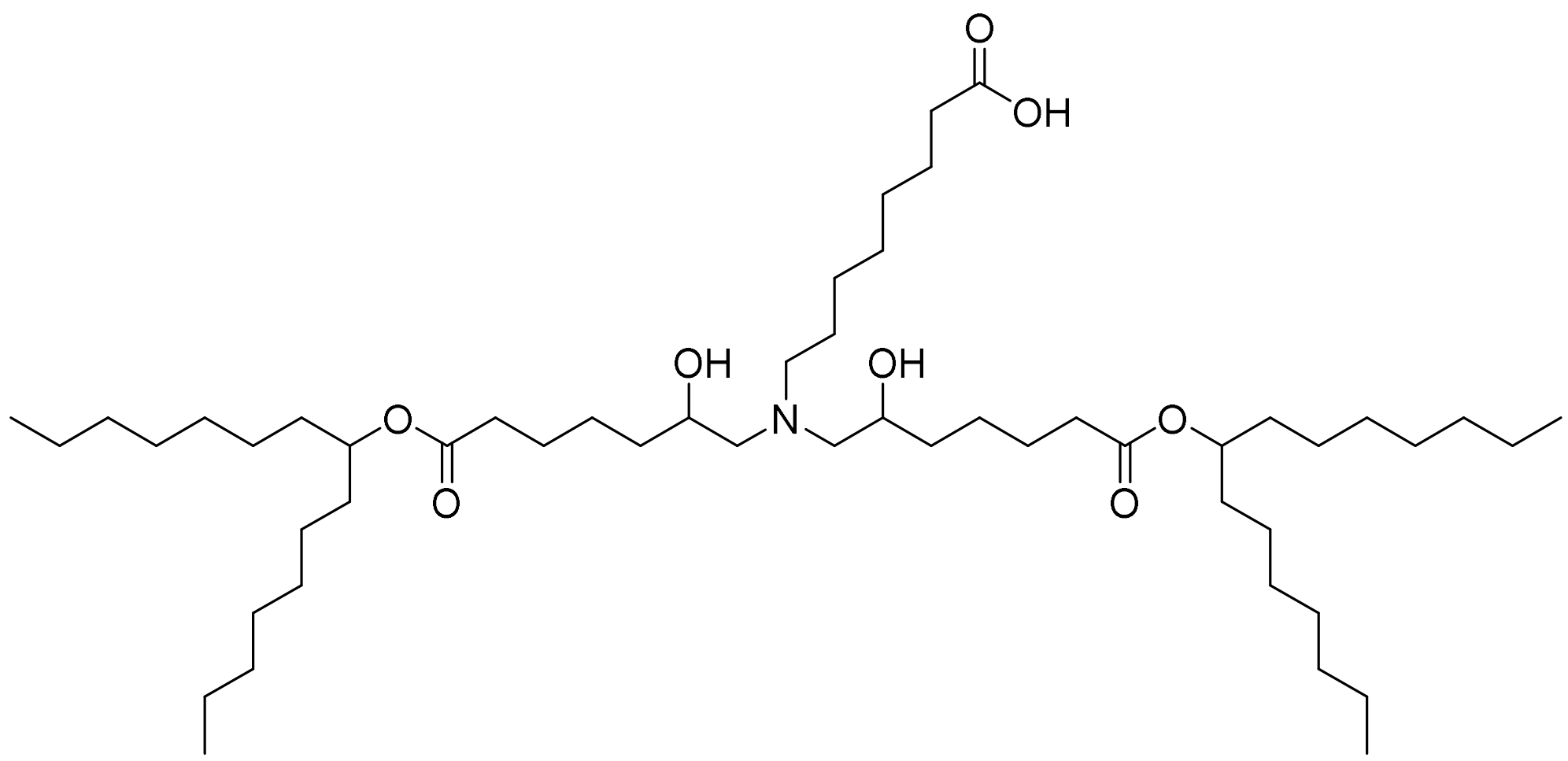

Figure S47. Chemical structure of compound E3.

^{1}H NMR (500 MHz, $CDCl_3$) δ 4.78 (p, *J* = 6.3 Hz, 2H), 4.19 – 4.02 (m, 2H), 3.27 – 2.93 (m, 6H), 2.31 – 2.19 (m, 6H), 1.80 – 1.70 (m, 2H), 1.61 – 1.52 (m, 7H), 1.49 – 1.37 (m, 13H), 1.35 – 1.27 (m, 8H), 1.26 – 1.14 (m, 42H), 0.84 – 0.78 (m, 12H).

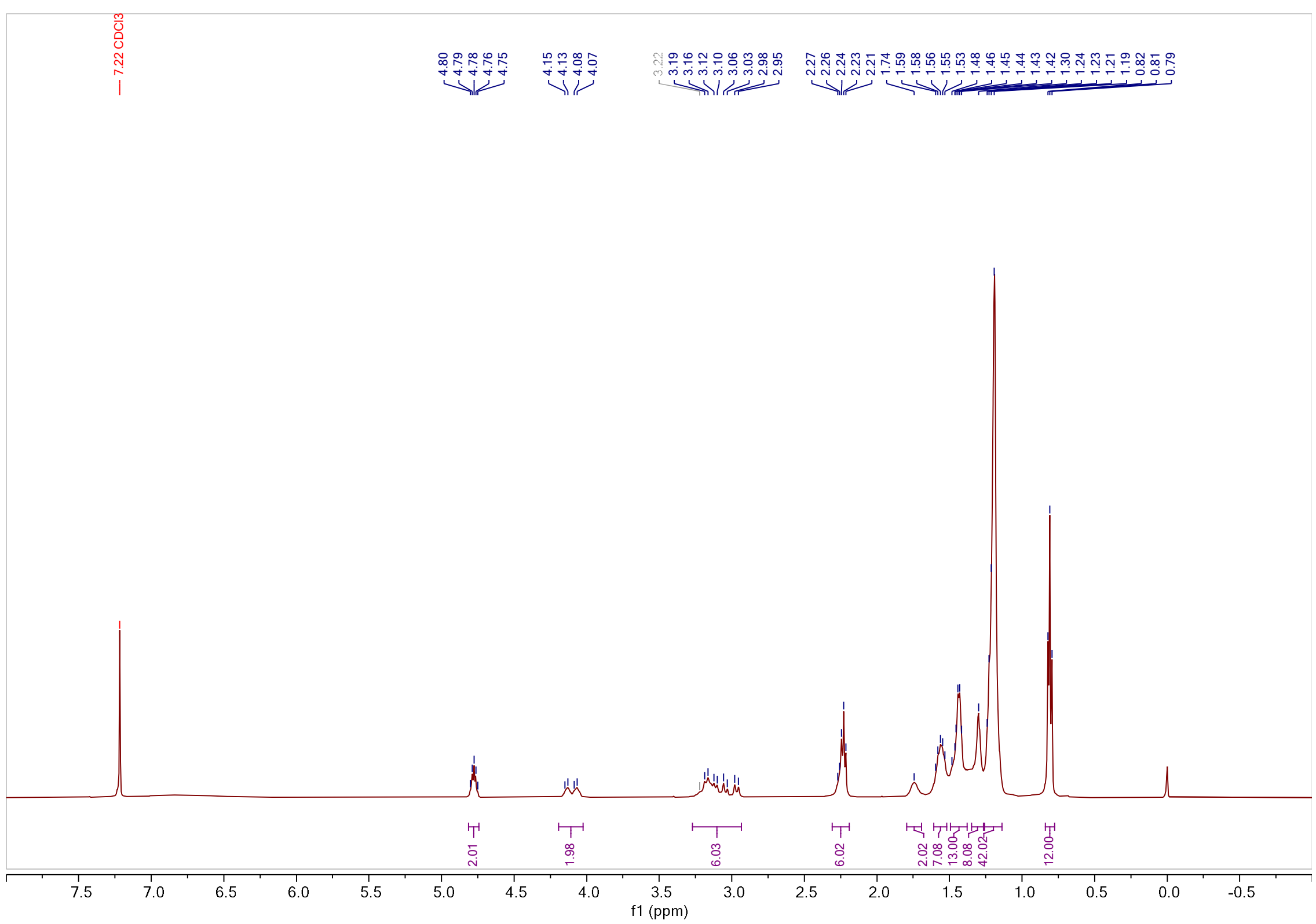

^{1}H NMR spectrum of compound **E3** ($CDCl_3$, 500 MHz)

^{13}C NMR (126 MHz, $CDCl_3$) δ 173.61, 173.59, 74.46, 74.44, 66.05, 65.04, 62.04, 61.46, 55.61, 53.44, 34.55, 34.40, 34.34, 34.07, 31.80, 29.49, 29.20, 28.43, 28.29, 26.10, 26.02, 25.32, 24.86, 24.77, 24.71, 24.51, 23.33, 23.15, 22.64, 14.11.

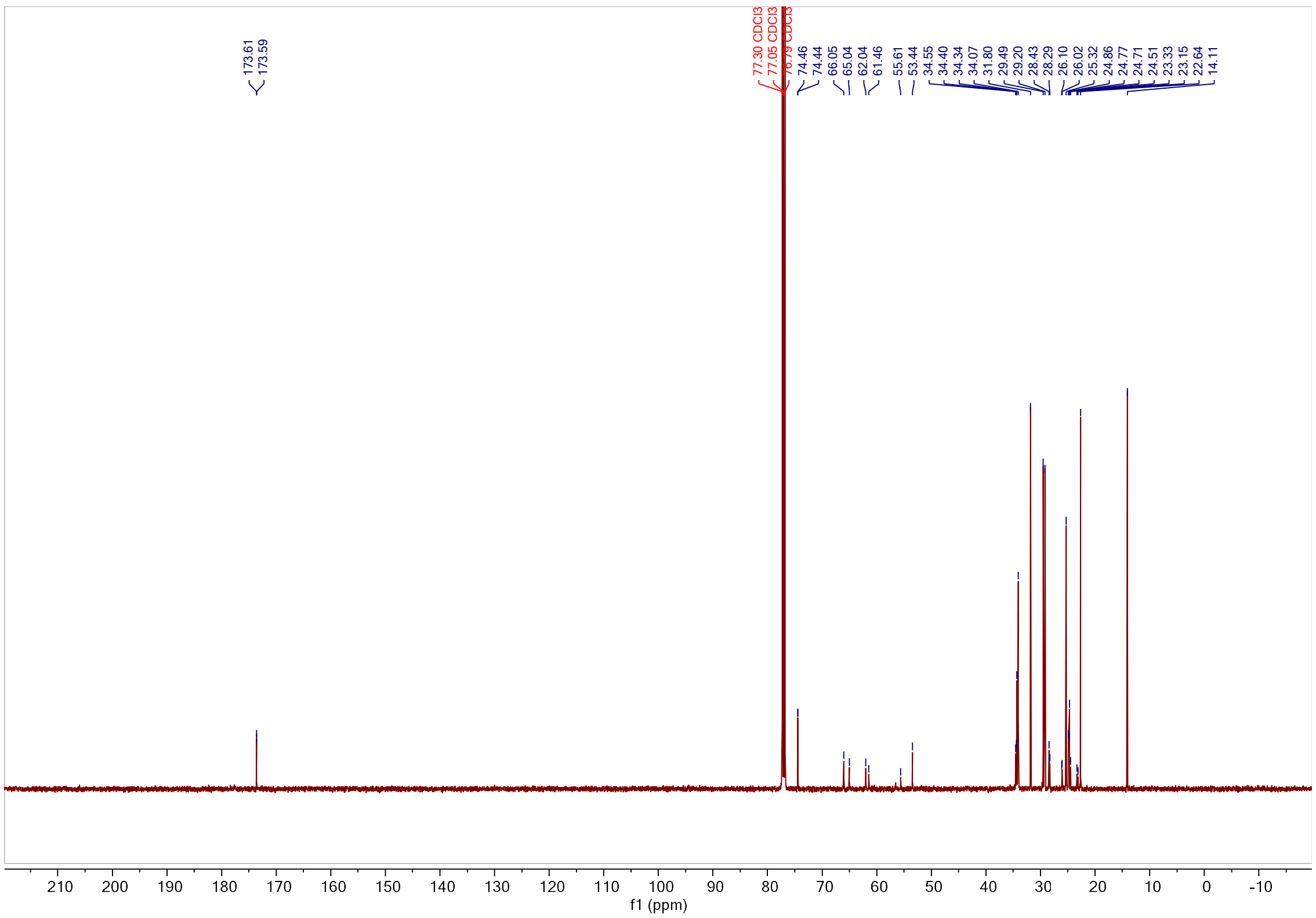

^{13}C-NMR spectrum of compound **E3** ($CDCl_3$, 126 MHz)

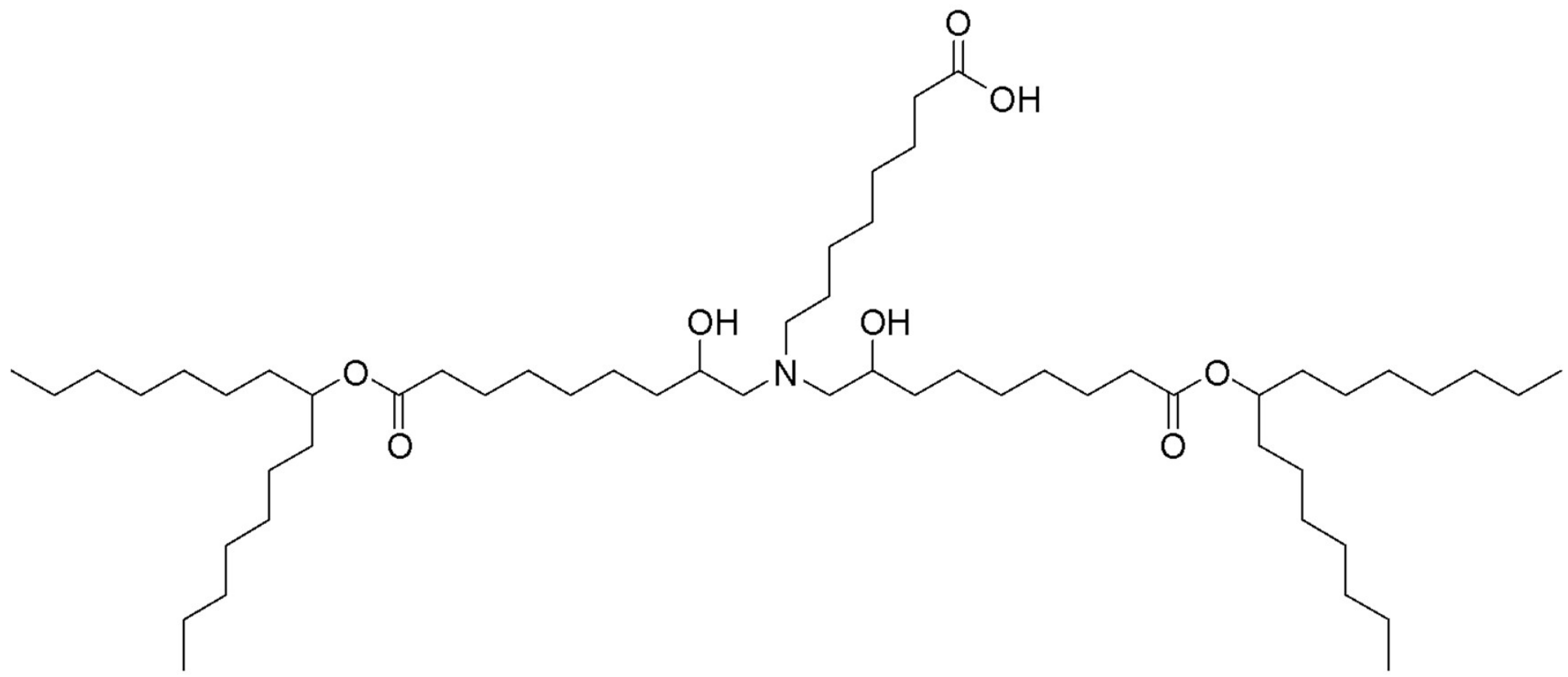

Figure S48. Chemical structure of compound E7.

^{1}H NMR (500 MHz, $CDCl_3$) δ 4.79 (p, *J* = 6.3 Hz, 2H), 4.15 – 4.03 (m, 2H), 3.21 – 3.02 (m, 4H), 3.99 – 2.87 (m, 2H), 2.30 – 2.17 (m, 6H), 1.80 – 1.70 (m, 2H), 1.59 – 1.50 (m, 7H), 1.48 – 1.40 (m, 11H), 1.38 – 1.13 (m, 60H), 0.85 – 0.77 (m, 12H).

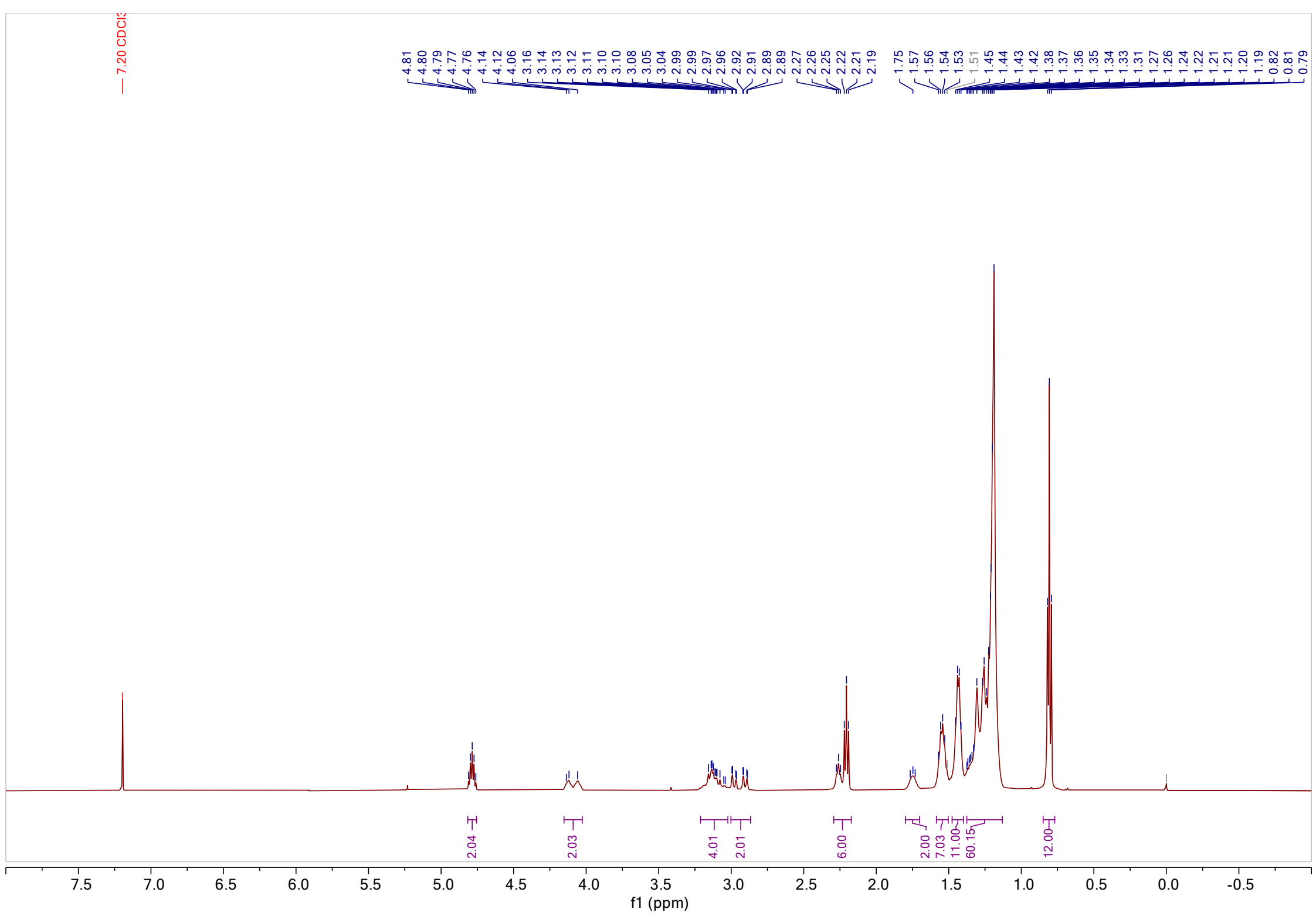

^{1}H NMR spectrum of compound **E7** ($CDCl_3$, 500 MHz)

^{13}C NMR (126 MHz, $CDCl_3$) δ 173.75, 74.27, 65.28, 65.19, 62.30, 55.66, 34.85, 34.63, 34.12,

31.80, 29.50, 29.21, 29.12, 29.10, 28.99, 28.38, 25.33, 25.16, 25.05, 24.99, 22.65, 24.43, 22.65, 14.12.

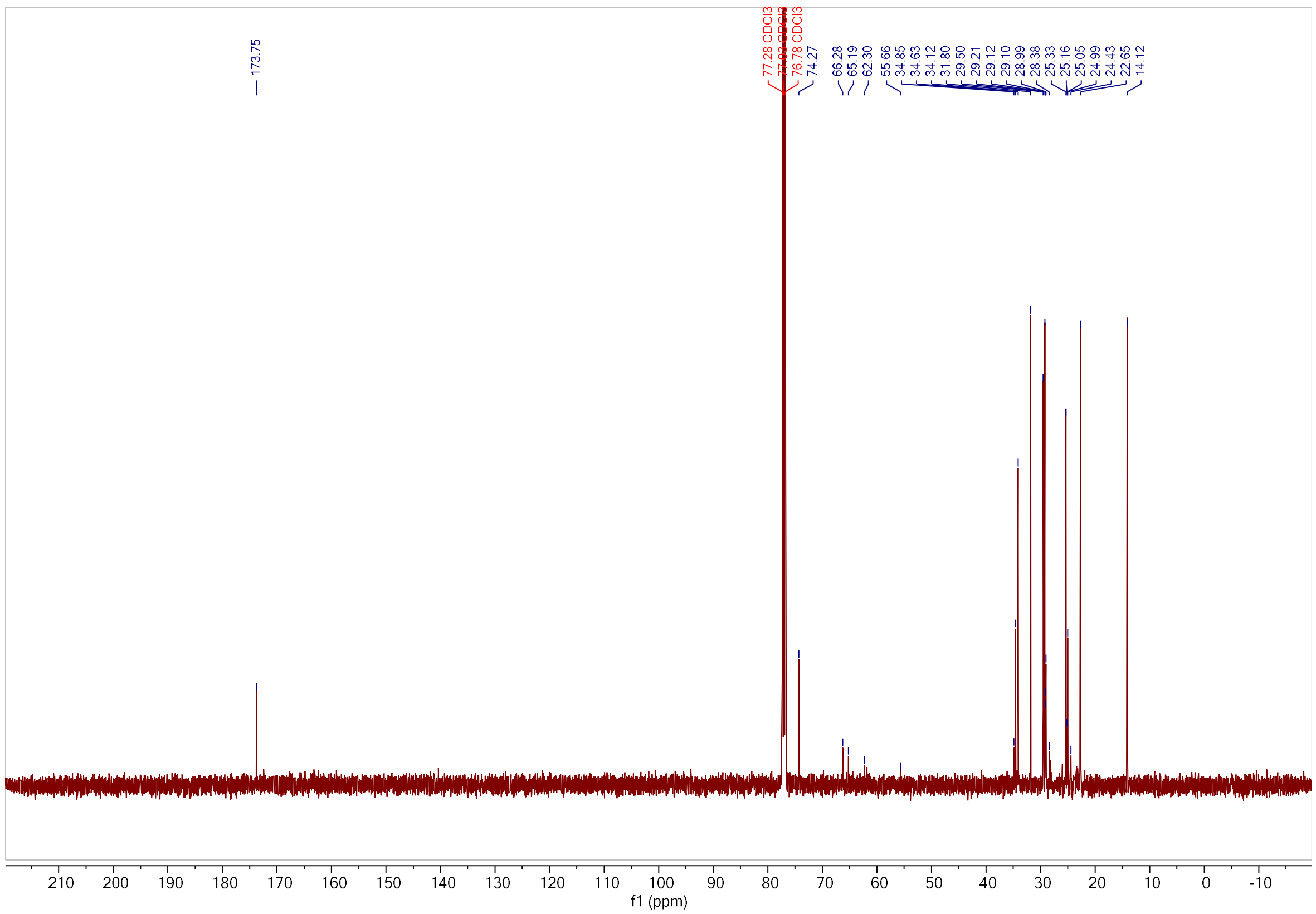

^{13}C-NMR spectrum of compound **E7** ($CDCl_3$, 126 MHz)

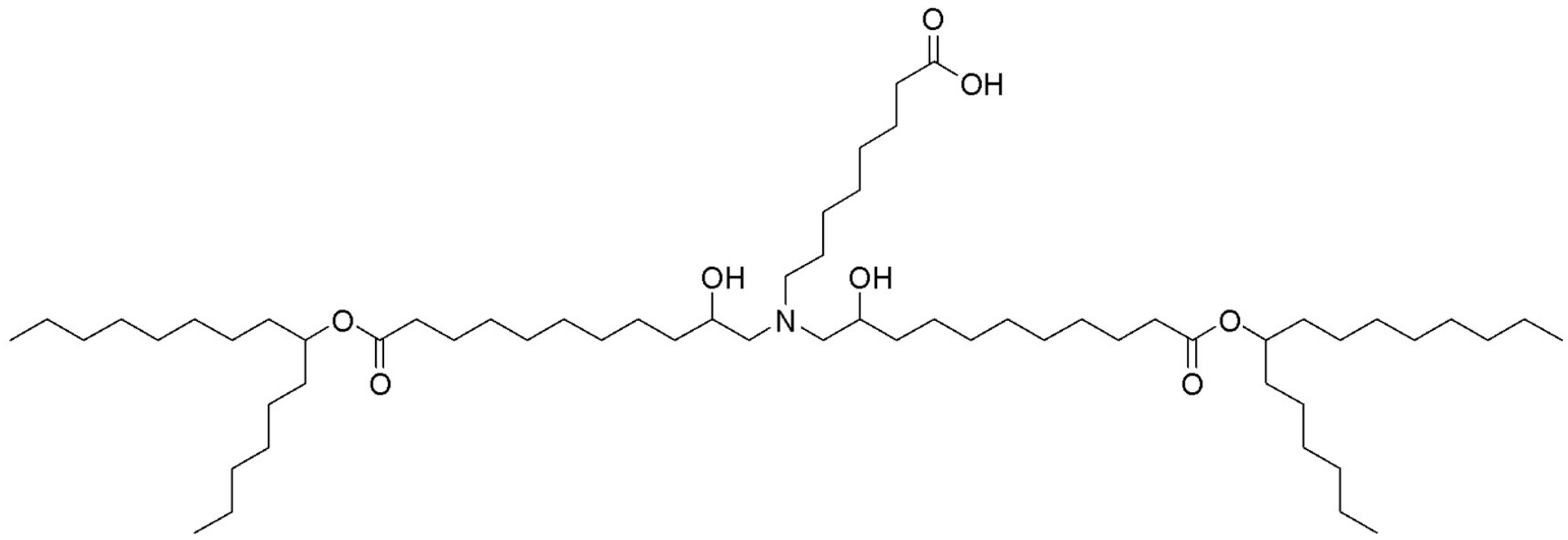

Figure S49. Chemical structure of compound E12.

^{1}H NMR (500 MHz, $CDCl_3$) δ 4.79 (p, *J* = 6.3 Hz, 2H), 4.21 – 3.95 (m, 2H), 3.89 – 2.82 (m, 6H), 2.31 – 2.25 (m, 2H), 2.24 – 2.16 (m, 4H), 1.85 – 1.69 (m, 2H), 1.59 – 1.51 (m, 6H), 1.50 – 1.38 (m, 12H), 1.38 – 1.26 (m, 10H), 1.25 – 1.12 (m, 58H), 0.84 – 0.78(m, 12H).

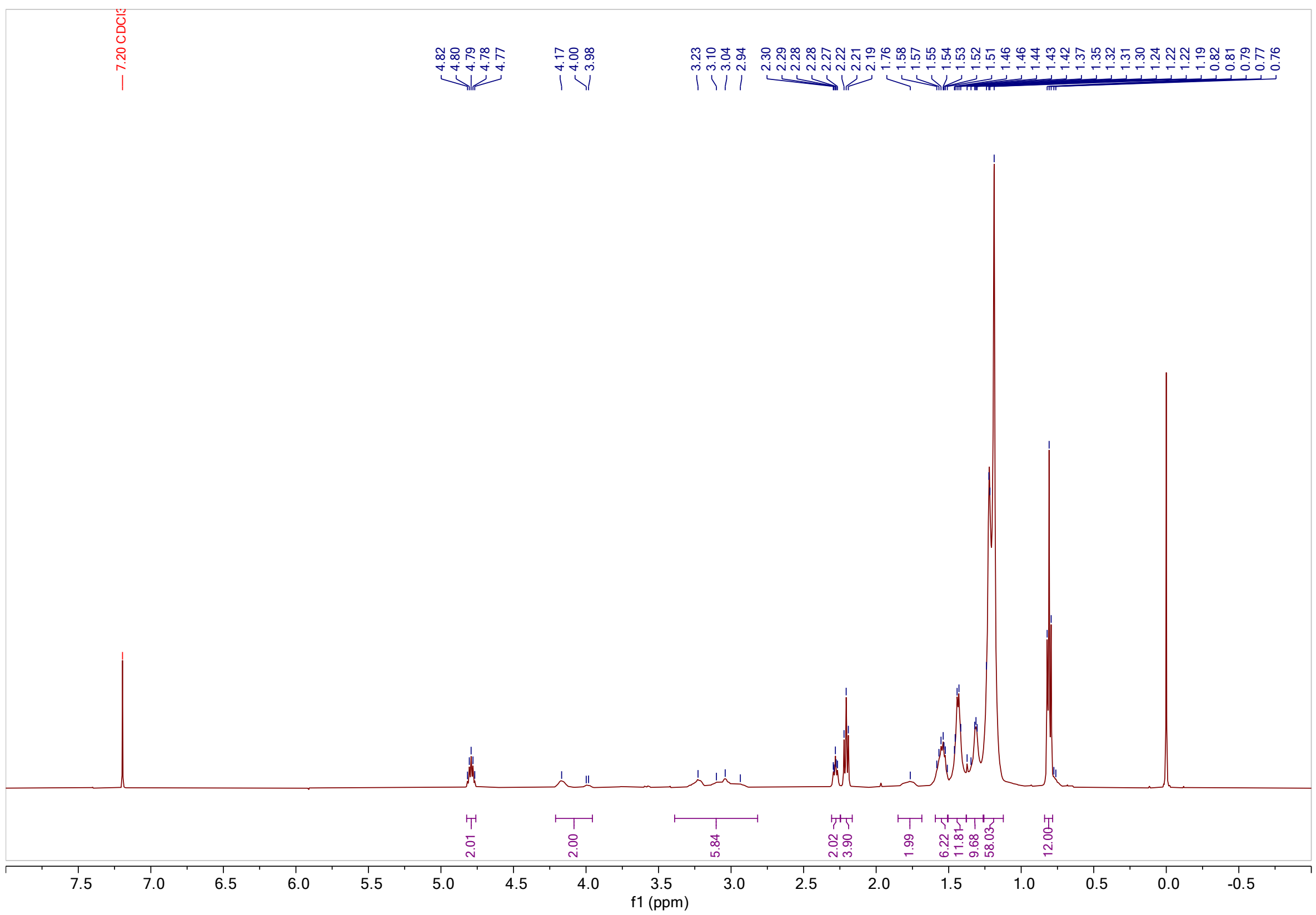

^{1}H NMR spectrum of compound **E12** ($CDCl_3$, 500 MHz)

^{13}C NMR (126 MHz, $CDCl_3$) δ 173.81, 74.19, 66.44, 62.32, 60.42, 35.00, 34.72, 34.15, 31.87, 31.76, 29.54, 29.51, 29.45, 29.43, 29.32, 29.25, 29.21, 29.15, 28.44, 25.32, 25.29, 25.25, 25.14, 22.68, 22.59, 14.13, 14.09.

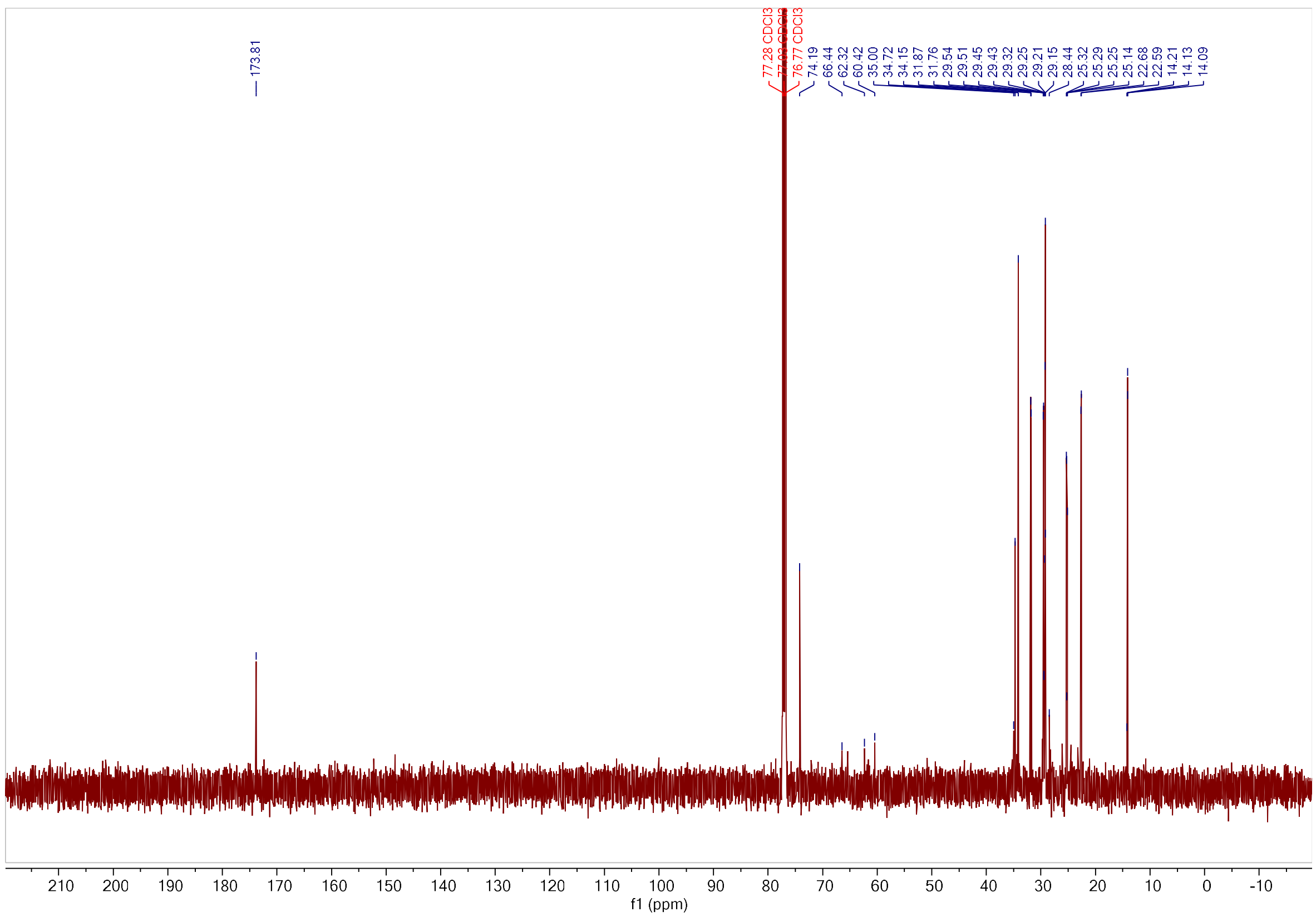

^{13}C-NMR spectrum of compound **E12** ($CDCl_3$, 126 MHz)

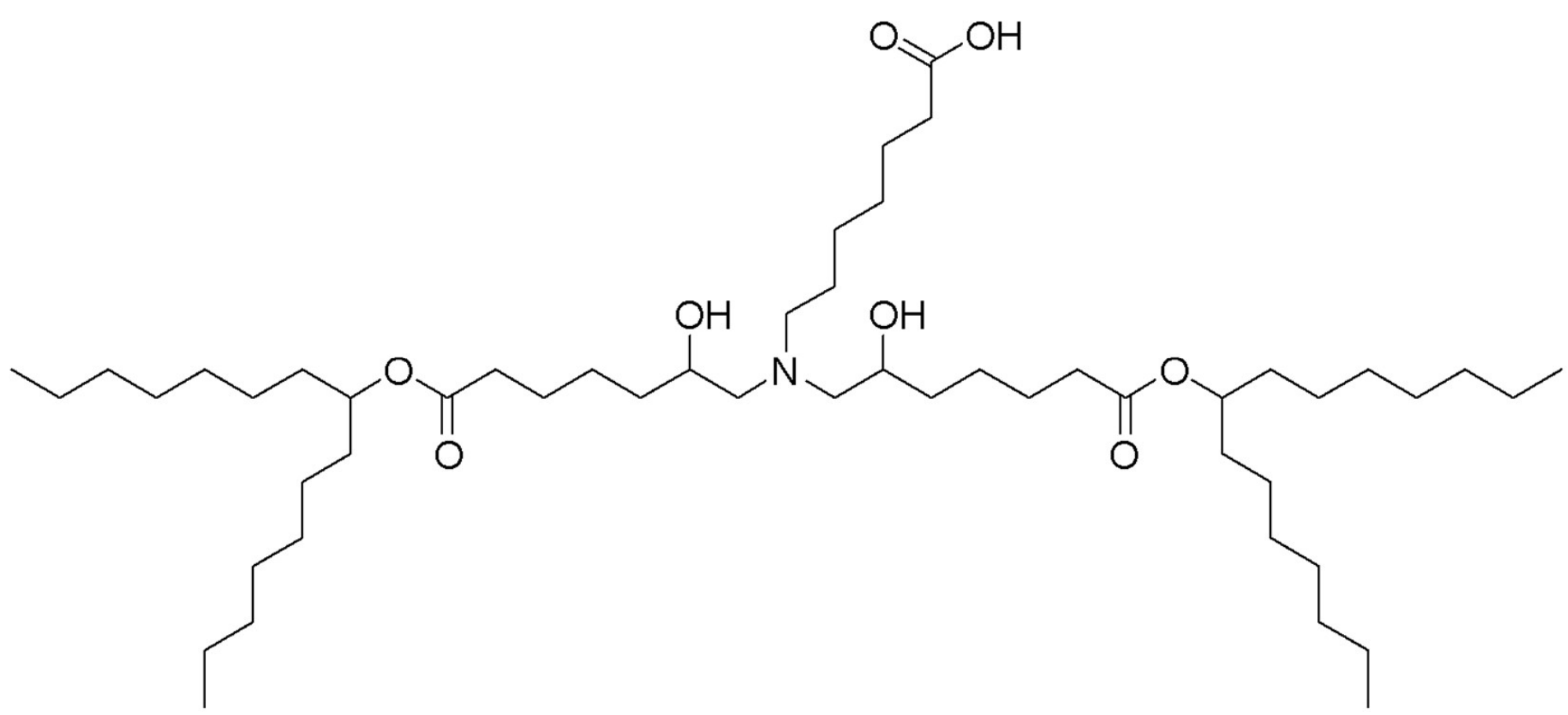

Figure S50. Chemical structure of compound E15.

^{1}H NMR (500 MHz, $CDCl_3$) δ 4.78 (p, *J* = 6.3 Hz, 2H), 4.20 – 4.06 (m, 2H), 3.33 – 3.09 (m, 4H), 3.09 – 2.91 (m, 2H), 2.33 - 2.19 (m, 6H), 1.86 – 1.71 (m, 2H), 1.63 – 1.52 (m, 7H), 1.50 – 1.33 (m, 19H), 1.27 – 1.13 (m, 42H), 0.84 – 0.78 (m, 12H).

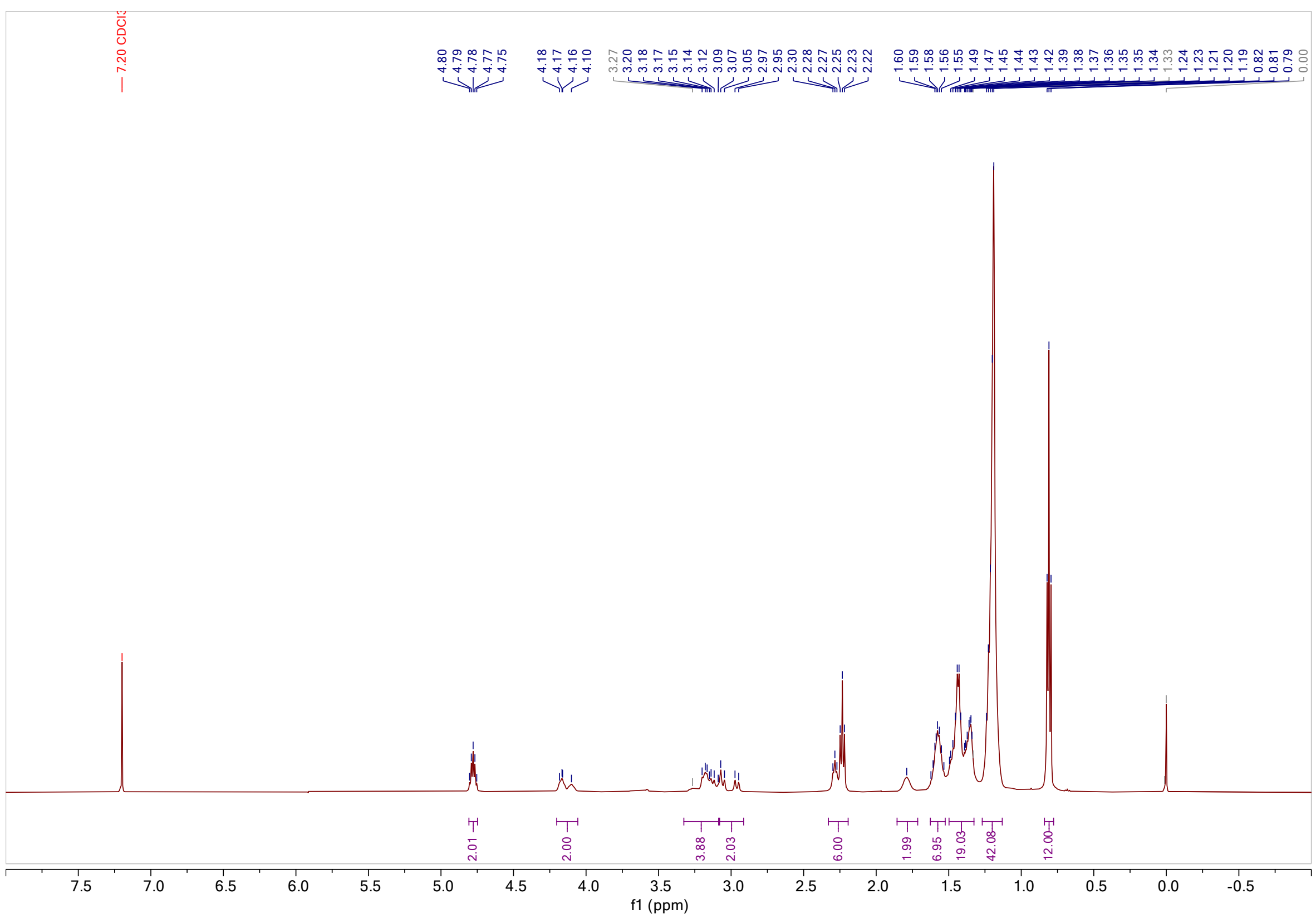

^{1}H NMR spectrum of compound **E15** ($CDCl_3$, 500 MHz)

^{13}C NMR (126 MHz, $CDCl_3$) δ 173.73, 74.55, 65.97, 62.18, 58.49, 50.92, 34.45, 34.33, 34.08, 31.81, 29.50, 29.21, 25.33, 24.80, 24.72, 24.62, 22.65, 14.12.

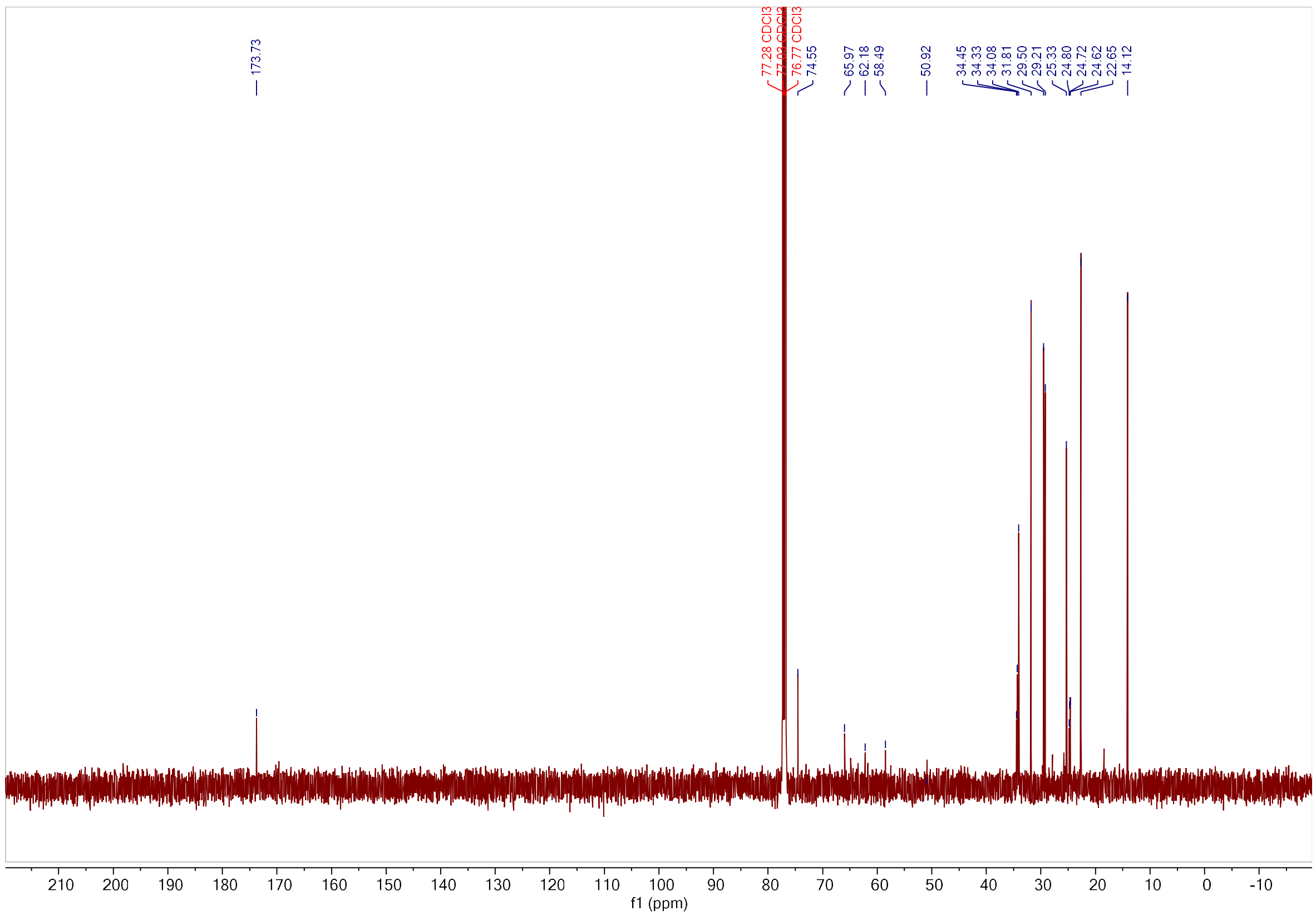

^{13}C-NMR spectrum of compound **E15** ($CDCl_3$, 126 MHz)

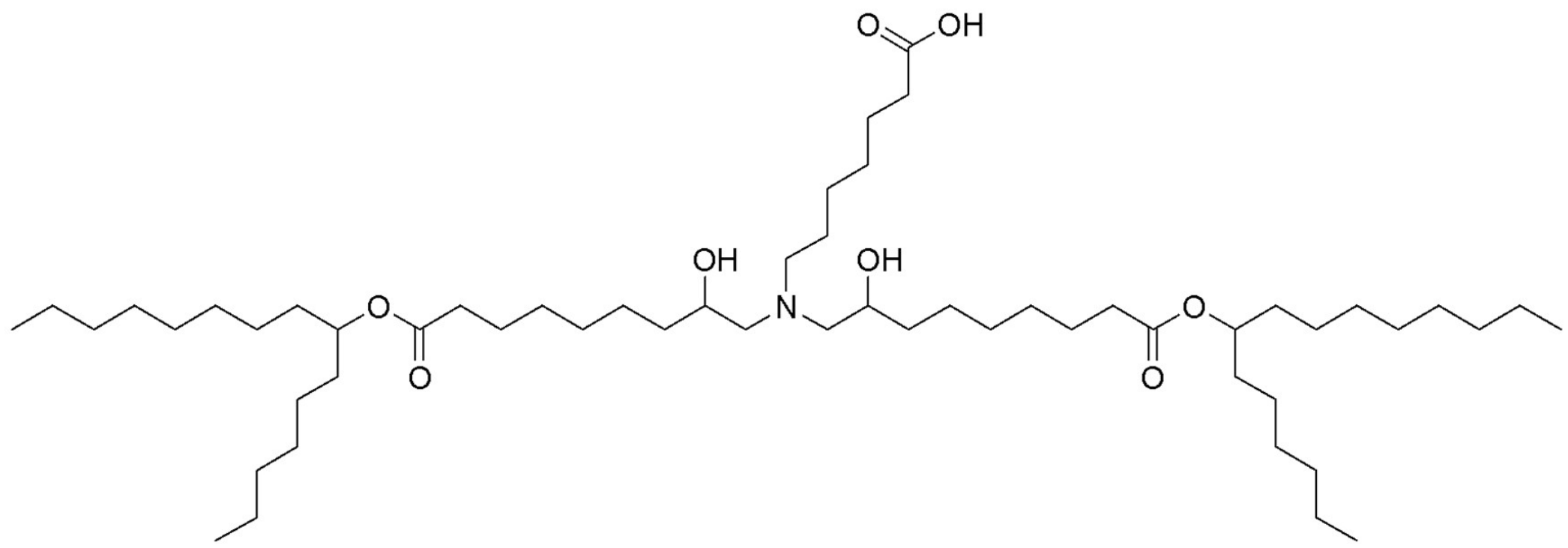

Figure S51. Chemical structure of compound E20.

^{1}H NMR (500 MHz, $CDCl_3$) δ 4.79 (p, *J* = 6.3 Hz, 2H), 4.20 – 4.00 (m, 2H), 3.32 – 3.07 (m, 4H), 3.07 – 2.90 (m, 2H), 2.33 – 2.24 (m, 2H), 2.24 – 2.17 (m, 4H), 1.86 – 1.69 (m, 2H), 1.61 – 1.51 (m, 7H), 1.50 – 1.40 (m, 11H), 1.39 – 1.32 (m, 6H), 1.32 – 1.14 (m, 52H), 0.84 – 0.78 (t, *J* = 6.8 Hz, 12H).

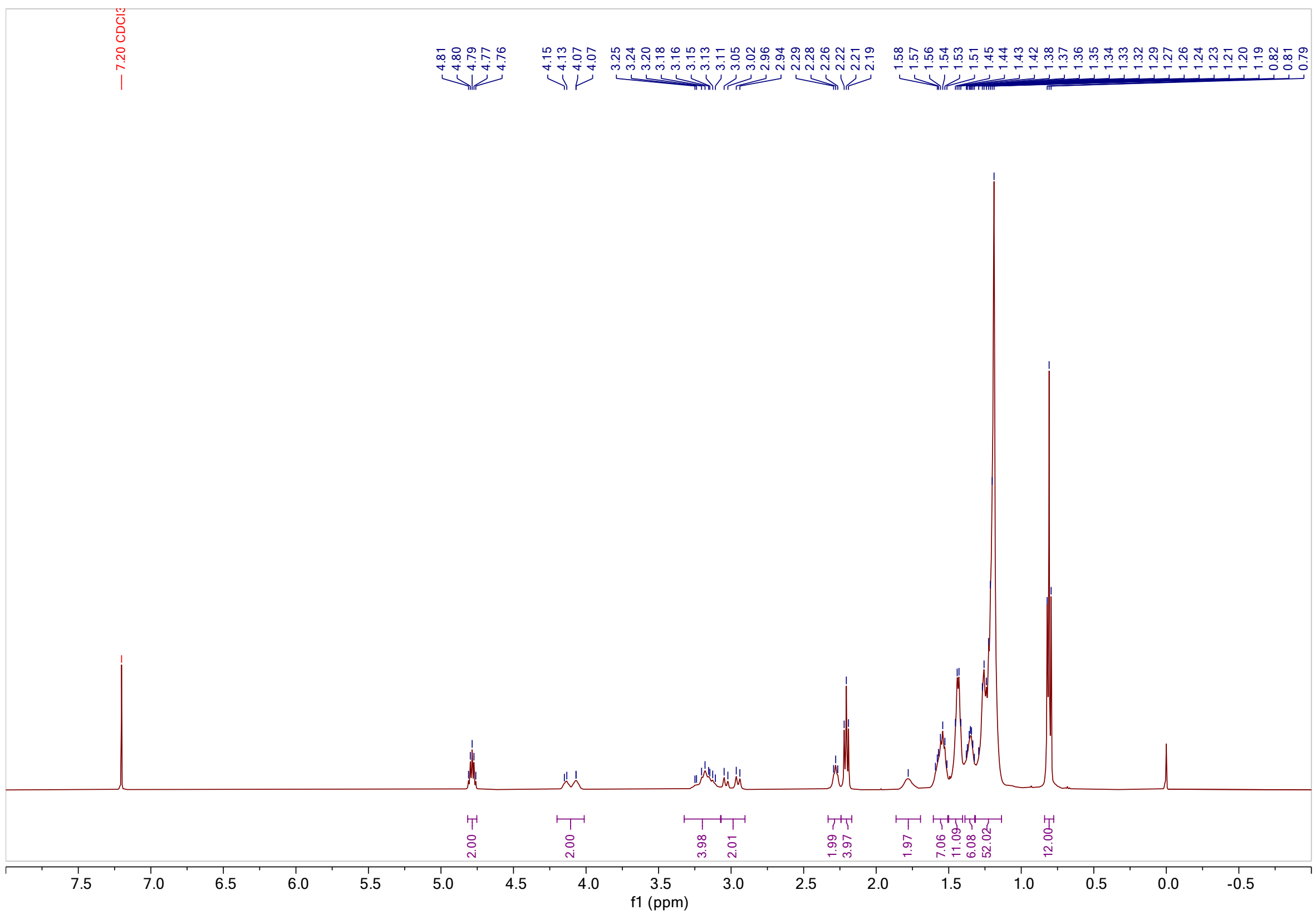

^{1}H NMR spectrum of compound **E20** ($CDCl_3$, 500 MHz)

^{13}C NMR (126 MHz, $CDCl_3$) δ 173.82, 74.13, 66.25, 66.15, 62.22, 61.74, 58.48, 55.54, 53.43, 34.88, 34.68, 34.63, 34.12, 31.87, 31.76, 29.54, 29.50, 29.25, 29.20, 29.08, 29.06, 28.99, 28.04, 25.32, 25.28, 25.13, 24.98, 22.68, 22.59, 18.42, 14.13, 14.10.

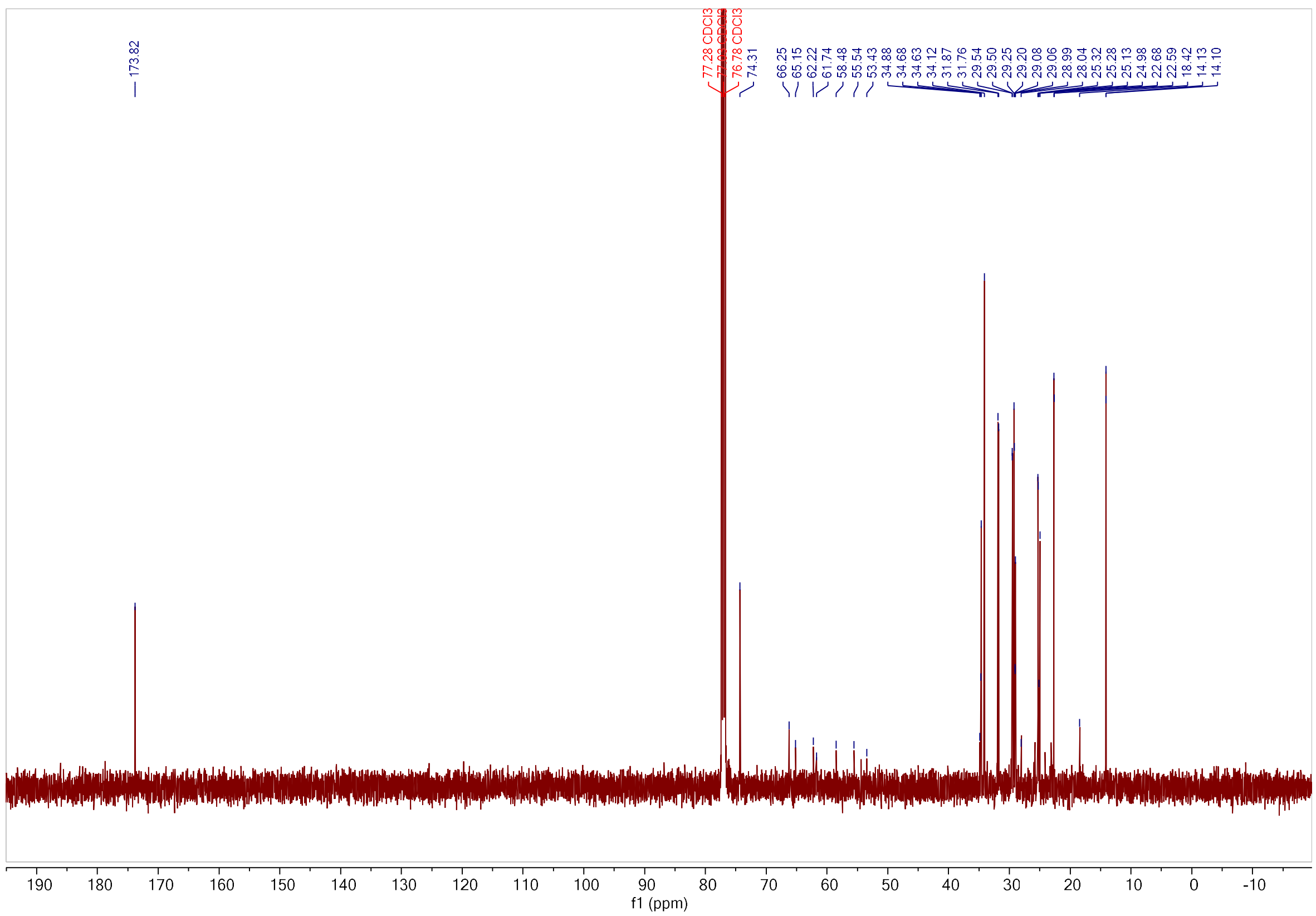

^{13}C-NMR spectrum of compound **E20** ($CDCl_3$, 126 MHz)

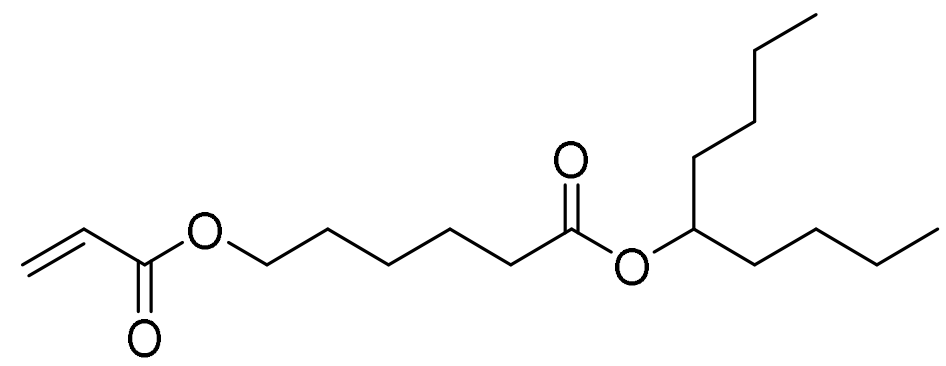

Figure S52. Chemical structure of compound 8a.

^{1}H NMR (500 MHz, $CDCl_3$) δ 6.38 – 6.29 (m, 1H), 6.08 – 6.00 (m, 1H), 5.78 – 5.72 (m, 1H), 4.80 (p, *J* = 6.3 Hz, 1H), 4.09 (t, *J* = 6.6 Hz, 2H), 2.24 (t, *J* = 7.5 Hz, 2H), 1.56-1.68 (m, 4H), 1.47-1.41 (m, 4H), 1.40 – 1.31 (m, 2H), 1.29 – 1.14 (m, 8H), 0.84 – 0.78 (m, 6H).

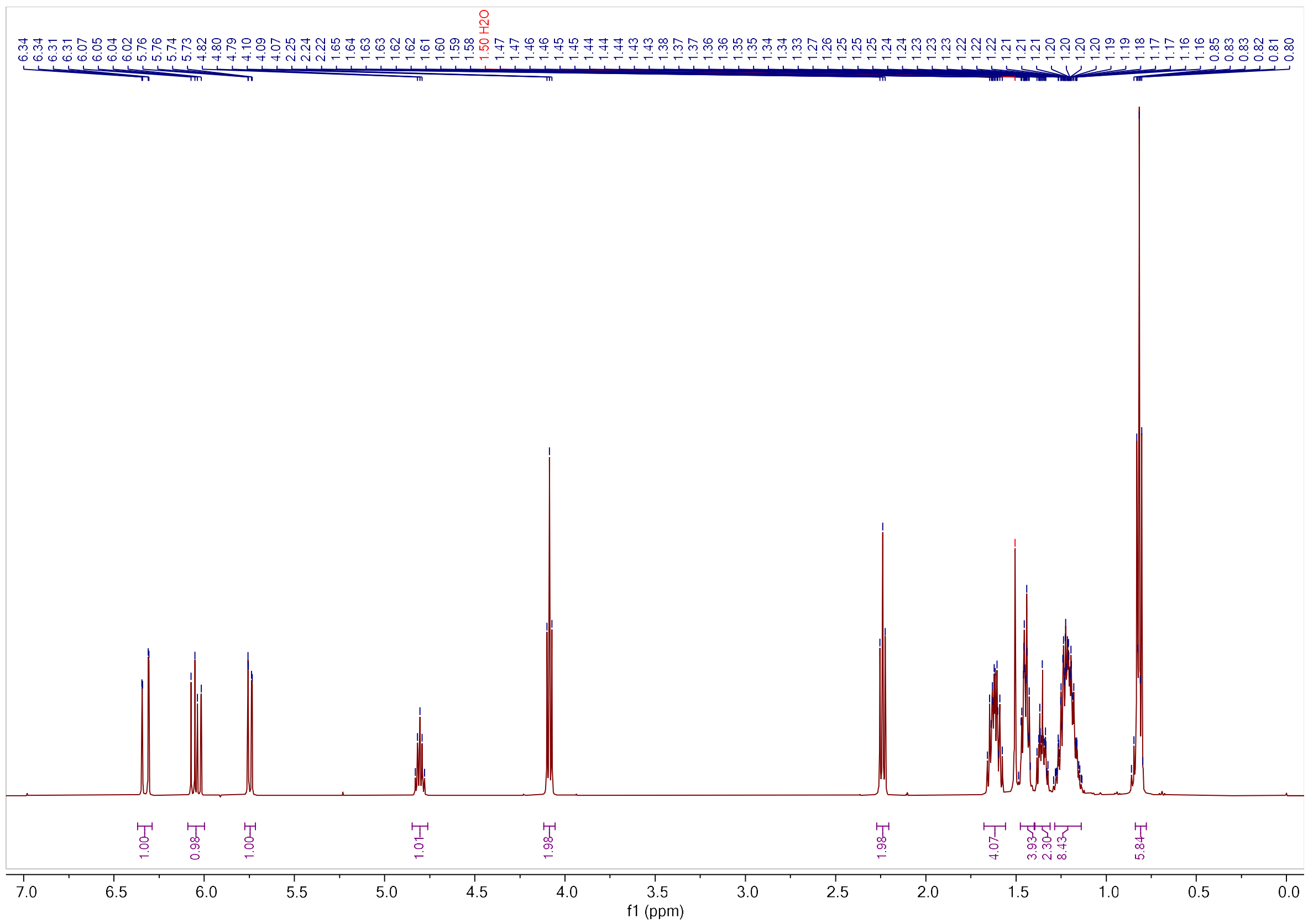

^{1}H NMR spectrum of compound **8a** ($CDCl_3$, 500 MHz)

^{13}C NMR (126 MHz, $CDCl_3$) δ 173.36, 166.29, 130.55, 128.56, 74.23, 64.41, 34.53, 33.84, 28.36, 27.51, 25.60, 24.77, 22.60, 14.02.

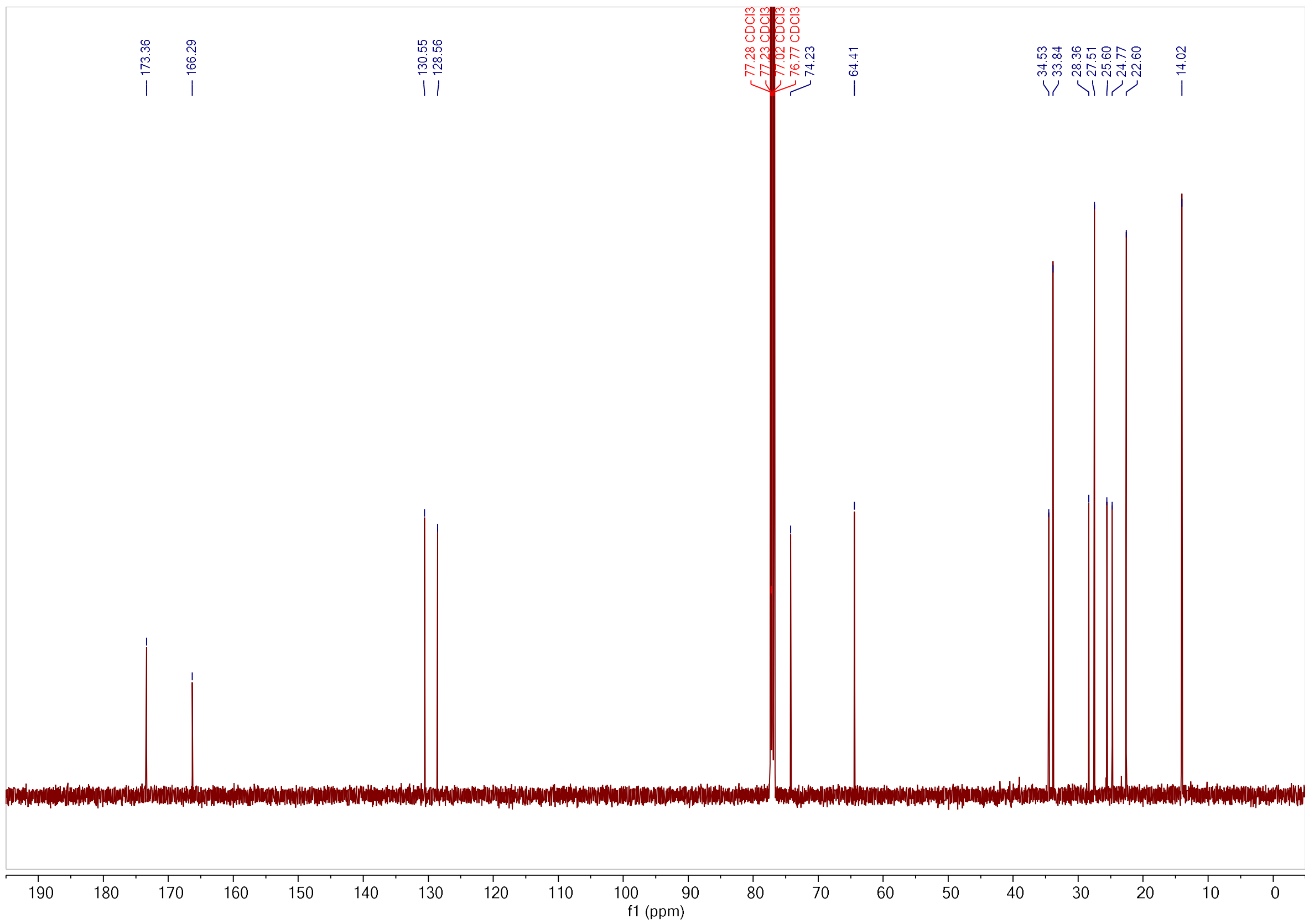

^{13}C-NMR spectrum of compound **8a** ($CDCl_3$, 126 MHz)

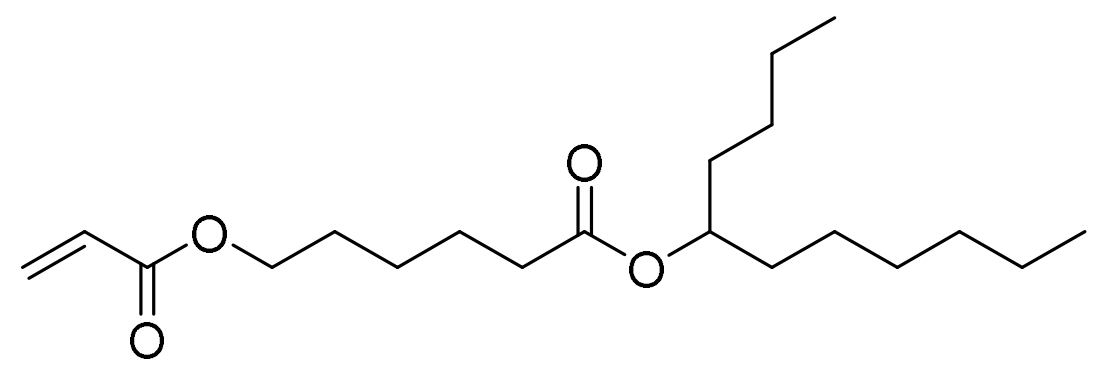

Figure S53. Chemical structure of compound 8b.

^{1}H NMR (500 MHz, $CDCl_3$) δ 6.36 – 6.29 (m, 1H), 6.10 – 6.00 (m, 1H), 5.78 – 5.71 (m, 1H), 4.80 (p, *J* = 6.2 Hz, 1H), 4.09 (t, *J* = 6.6 Hz, 2H), 2.24 (t, *J* = 7.5 Hz, 2H), 1.68 – 1.56 (m, 4H), 1.49-1.39 (m, 4H), 1.40 – 1.30 (m, 2H), 1.30 – 1.13 (m, 13H), 0.88 – 0.77 (m, 6H).

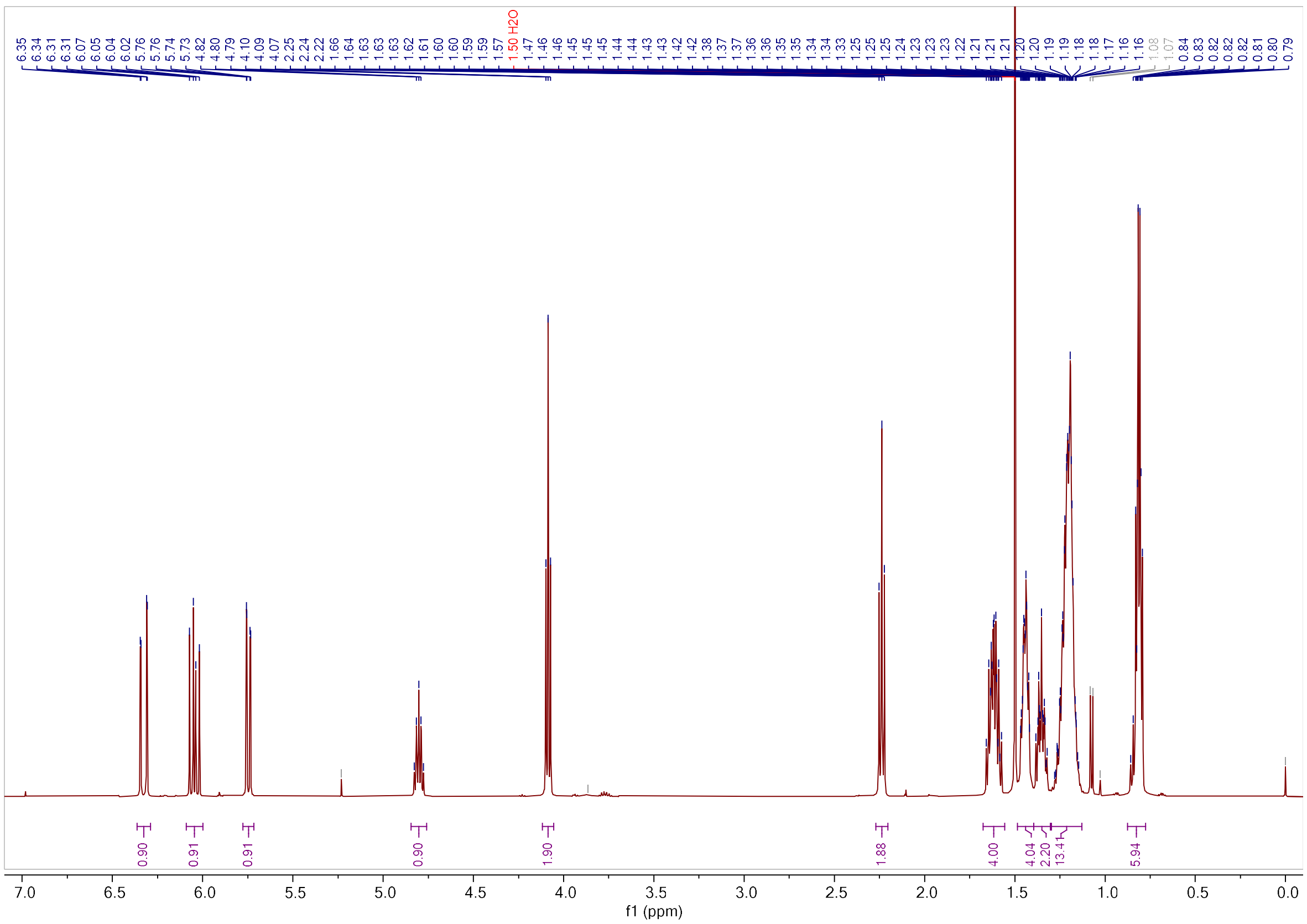

^{1}H NMR spectrum of compound **8b** ($CDCl_3$, 500 MHz)

^{13}C NMR (126 MHz, $CDCl_3$) δ 173.35, 166.29, 130.55, 128.56, 74.25, 64.41, 34.53, 34.15, 33.84, 31.76, 29.20, 28.36, 27.51, 25.60, 25.30, 24.78, 23.51, 22.60, 22.59, 14.07, 14.02.

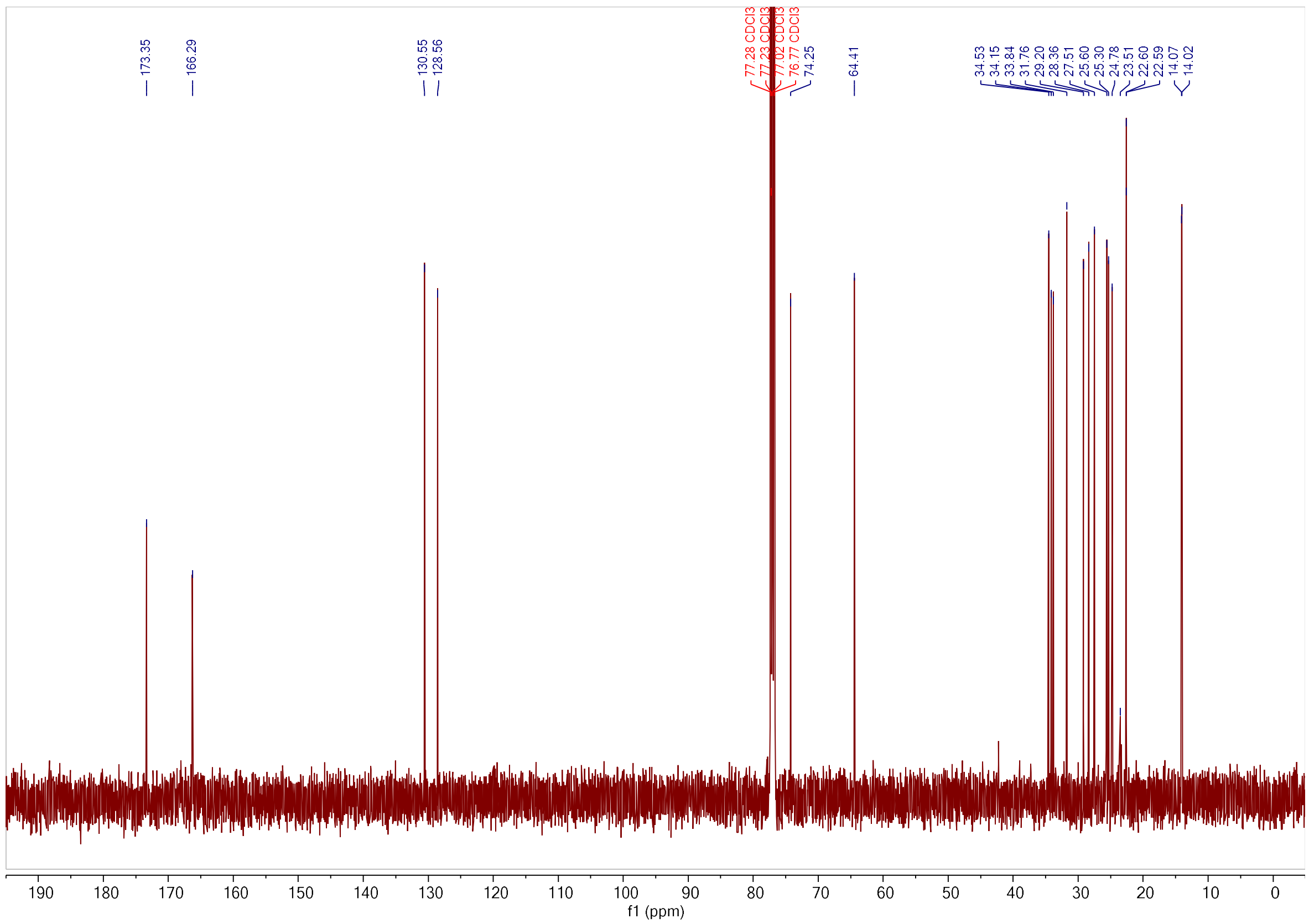

^{13}C-NMR spectrum of compound **8b** ($CDCl_3$, 126 MHz)

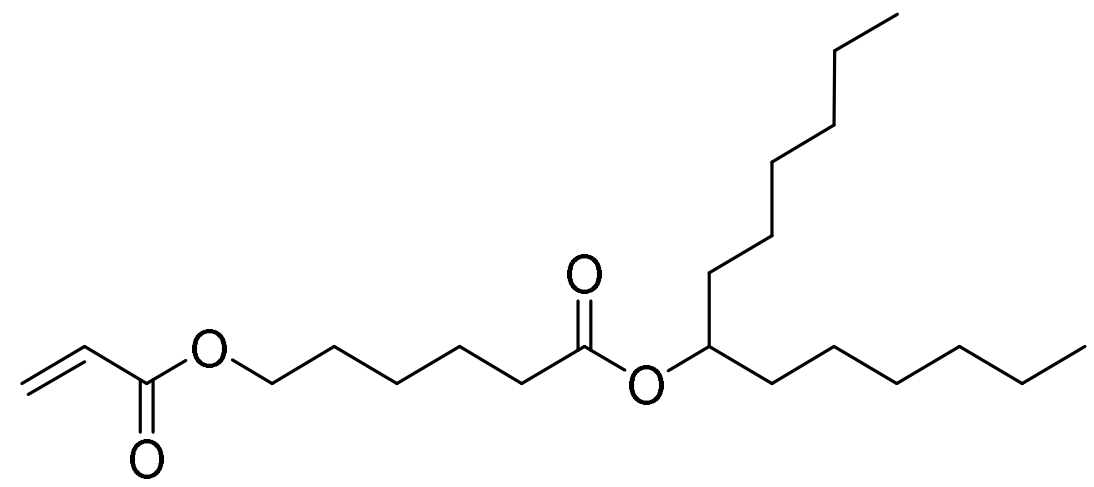

Figure S54. Chemical structure of compound 8c.

^{1}H NMR (500 MHz, $CDCl_3$) δ 6.36 – 6.29 (m, 1H), 6.10 – 6.00 (m, 1H), 5.77 – 5.72 (m, 1H), 4.80 (p, *J=6.5 Hz*, 1H), 4.08 (t, *J* = 6.7 Hz, 2H), 2.23 (t, *J* = 7.5 Hz, 2H), 1.67 – 1.57 (m, 4H), 1.47-1.39 (m, 4H), 1.39 – 1.32 (m, 2H), 1.25 – 1.15 (m, 16H), 0.84 – 0.78 (m, 6H).

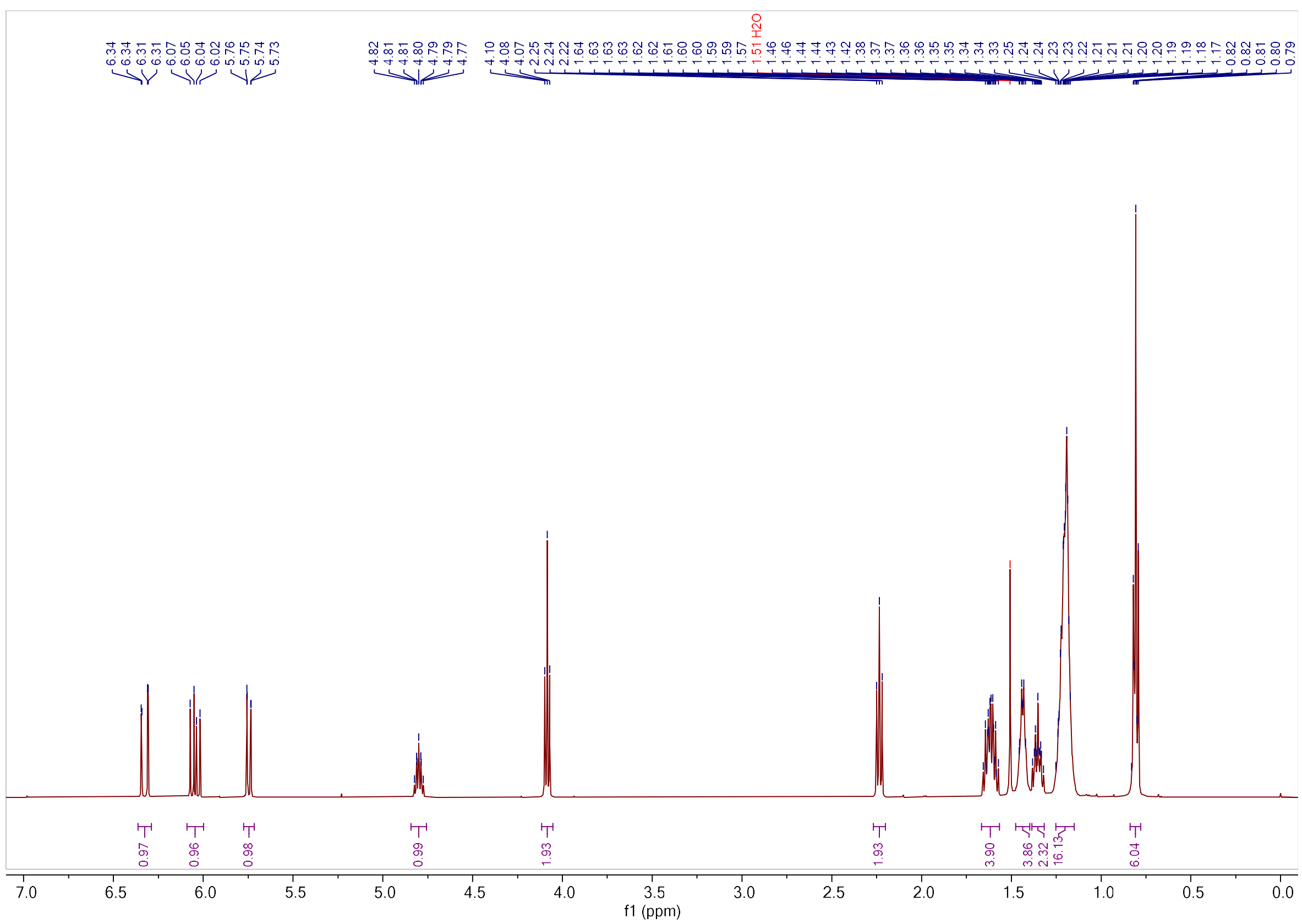

^{1}H NMR spectrum of compound **8c** ($CDCl_3$, 500 MHz)

^{13}C NMR (126 MHz, $CDCl_3$) δ 173.35, 166.28, 130.54, 128.57, 74.28, 64.40, 37.51, 34.54, 34.15, 31.86, 31.76, 29.20, 28.36, 25.60, 25.30, 24.79, 22.59, 14.10, 14.07.

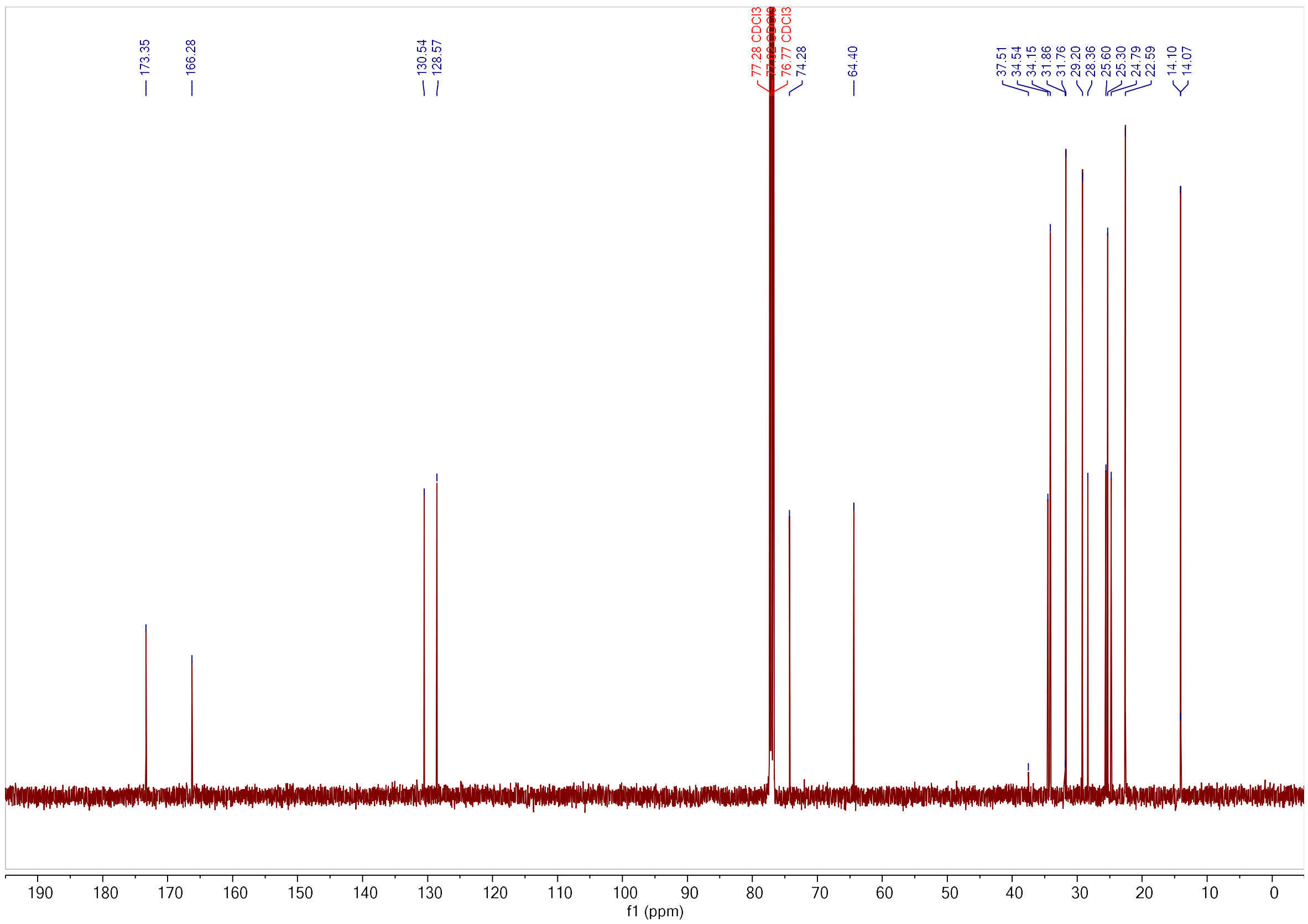

^{13}C-NMR spectrum of compound **8c** ($CDCl_3$, 126 MHz)

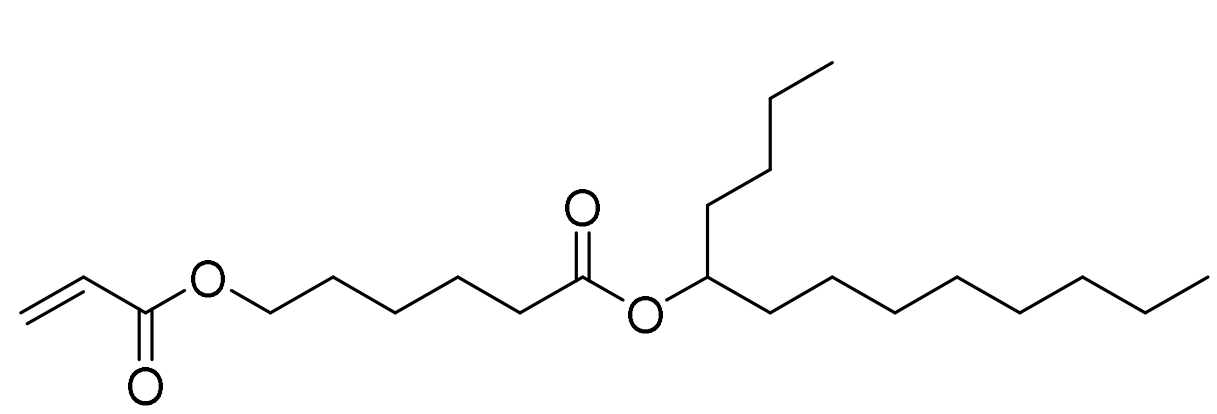

Figure S55. Chemical structure of compound 8d.

1H NMR (400 MHz, $CDCl_3$) δ 6.43 – 6.33 (m, 1H), 6.16 – 6.03 (m, 1H), 5.84 – 5.76 (m, 1H), 4.86 (p, J = 6.3 Hz, 1H), 4.14 (t, J = 6.6 Hz, 2H), 2.29 (t, J = 7.5 Hz, 2H), 1.67 (ddt, J = 15.1, 10.8, 7.1 Hz, 4H), 1.53 – 1.46 (m, 4H), 1.44 – 1.37 (m, 2H), 1.30-1.20 (m, 16H), 0.90 – 0.84 (m, 6H).

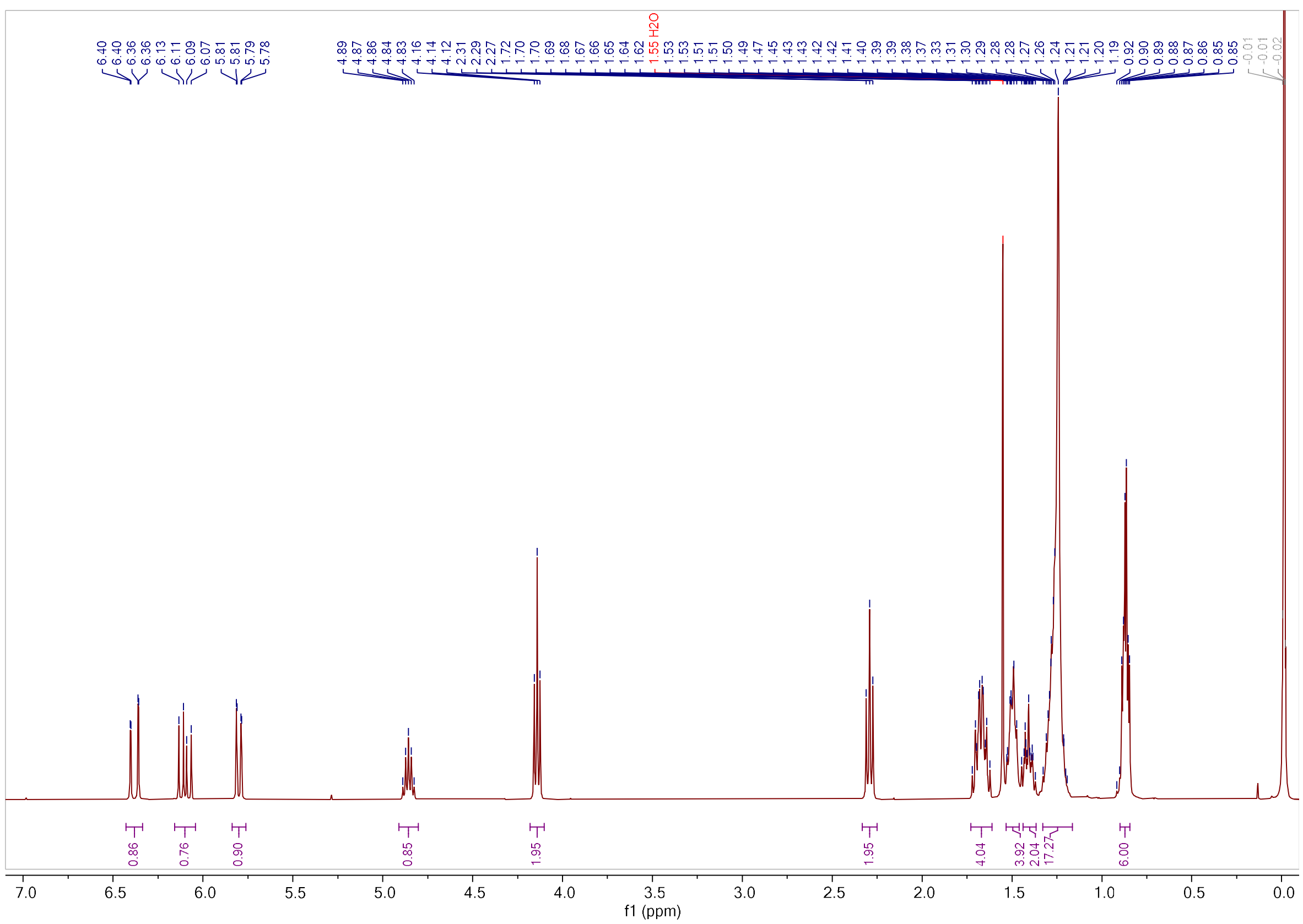

^{1}H NMR spectrum of compound **8d** ($CDCl_3$, 500 MHz)

^{13}C NMR (101 MHz, $CDCl_3$) δ 173.43, 166.36, 130.60, 74.33, 64.48, 34.61, 34.22, 33.90, 31.93, 29.61, 29.58, 29.31, 28.43, 27.58, 25.67, 25.41, 24.85, 22.67, 14.17, 14.08.

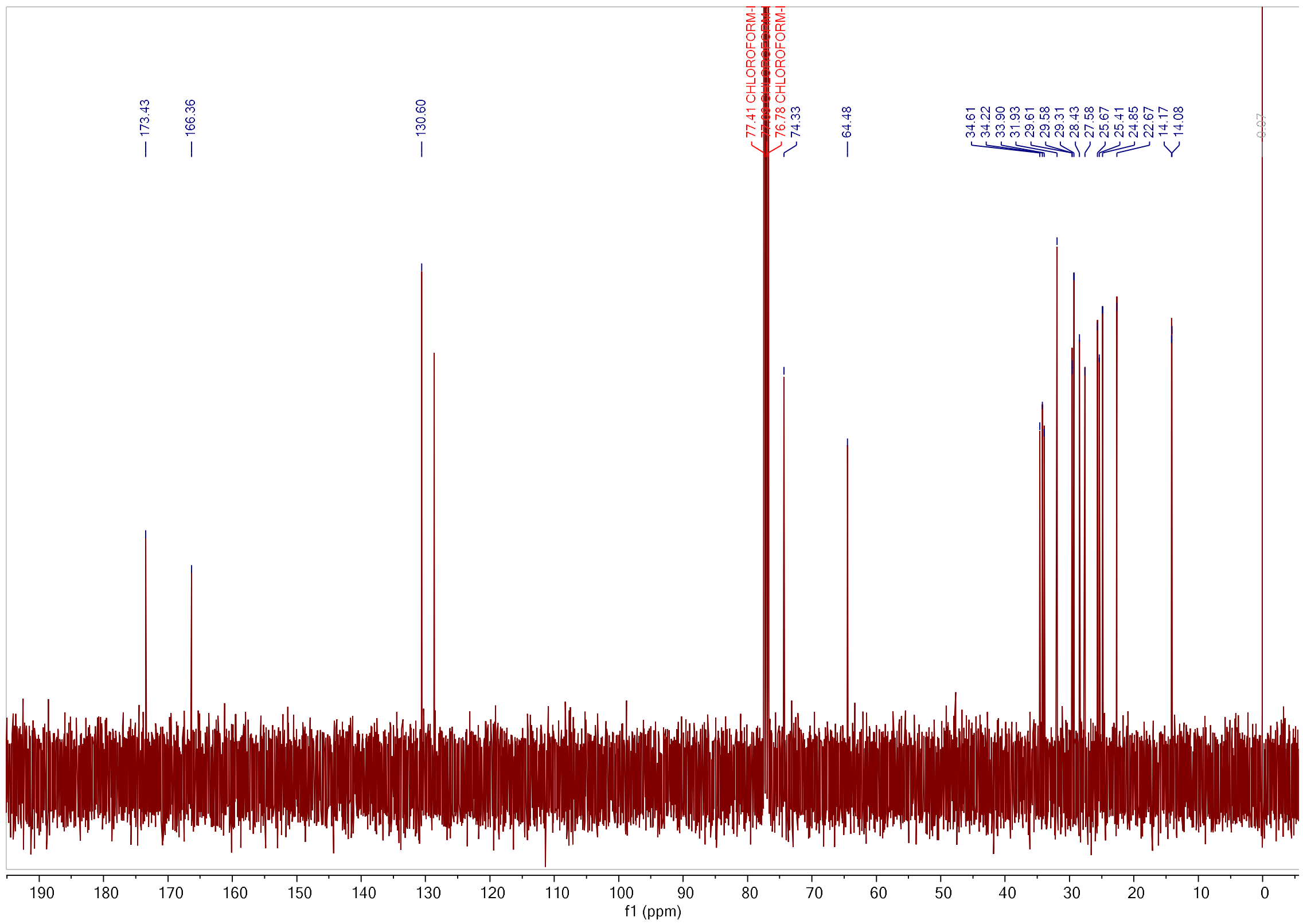

^{13}C-NMR spectrum of compound **8d** ($CDCl_3$, 101 MHz)

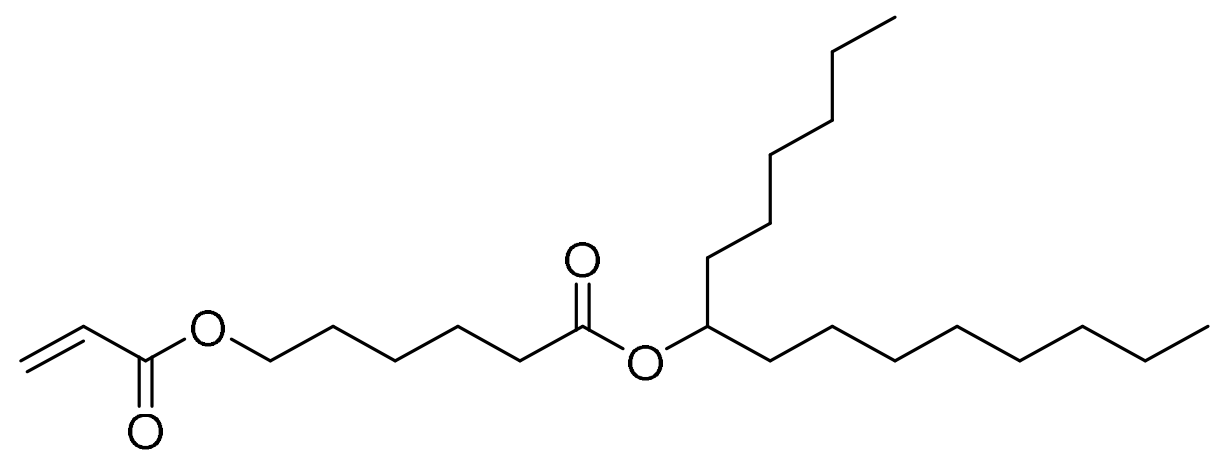

Figure S56. Chemical structure of compound 8e.

^{1}H NMR (400 MHz, $CDCl_3$) δ 6.42 – 6.33 (m, 1H), 6.16 – 6.04 (m, 1H), 5.83 – 5.79 (m, 1H), 4.85 (p, *J* = 6.3 Hz, 1H), 4.14 (t, *J* = 6.6 Hz, 2H), 2.29 (t, *J* = 7.5 Hz, 2H), 1.73-1.59 (m, 4H), 1.52 – 1.35 (m, 6H), 1.31-1.20 (m, 20H), 0.90 – 0.82 (m, 6H).

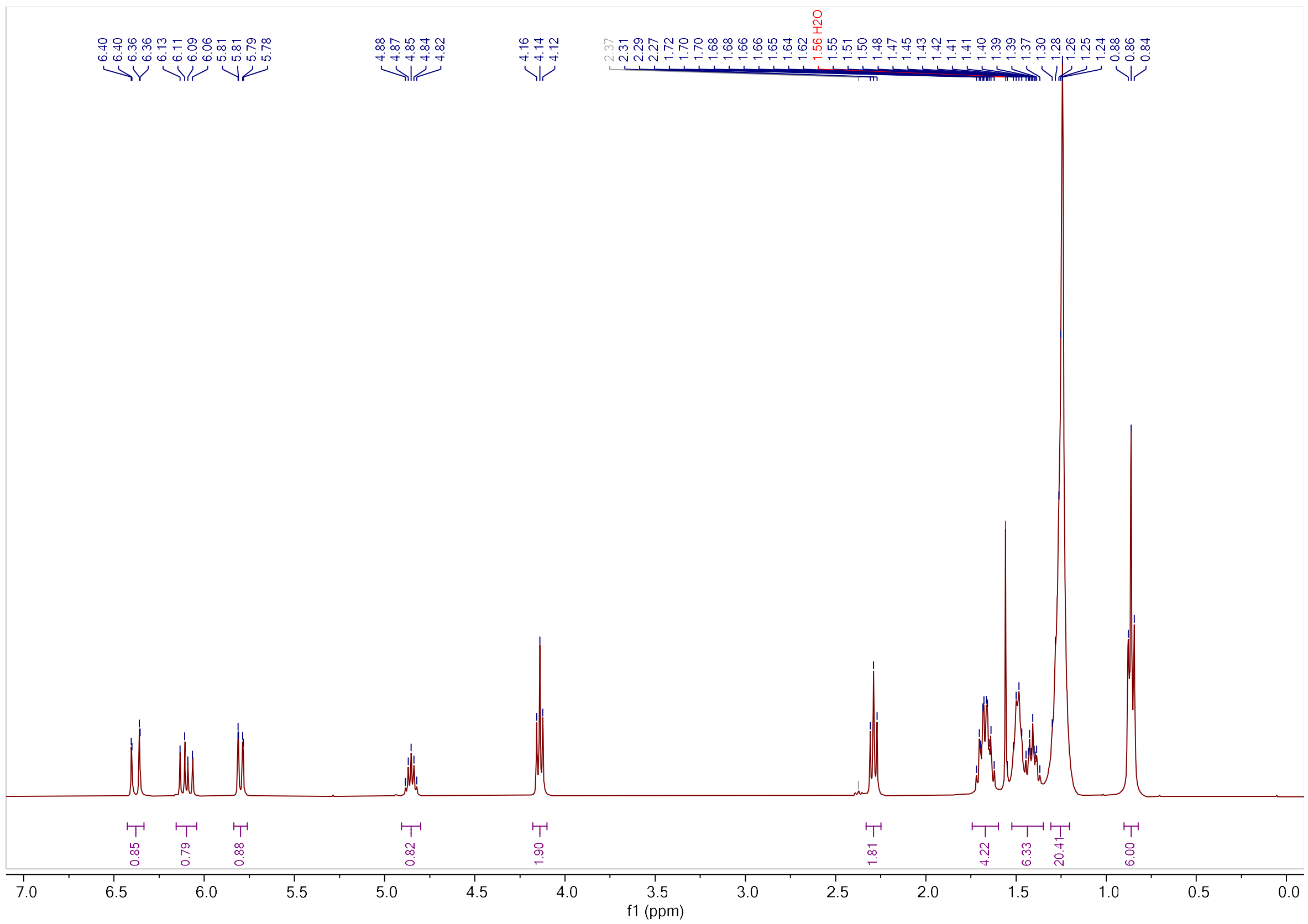

^{1}H NMR spectrum of compound **8e** ($CDCl_3$, 400 MHz)

^{13}C NMR (126 MHz, $CDCl_3$) δ 173.35, 166.28, 130.54, 128.57, 74.28, 64.40, 34.54, 34.14, 31.87, 31.78, 29.54, 29.51, 29.25, 29.21, 28.36, 25.60, 25.33, 25.30, 24.79, 22.67, 22.59, 14.11, 14.07.

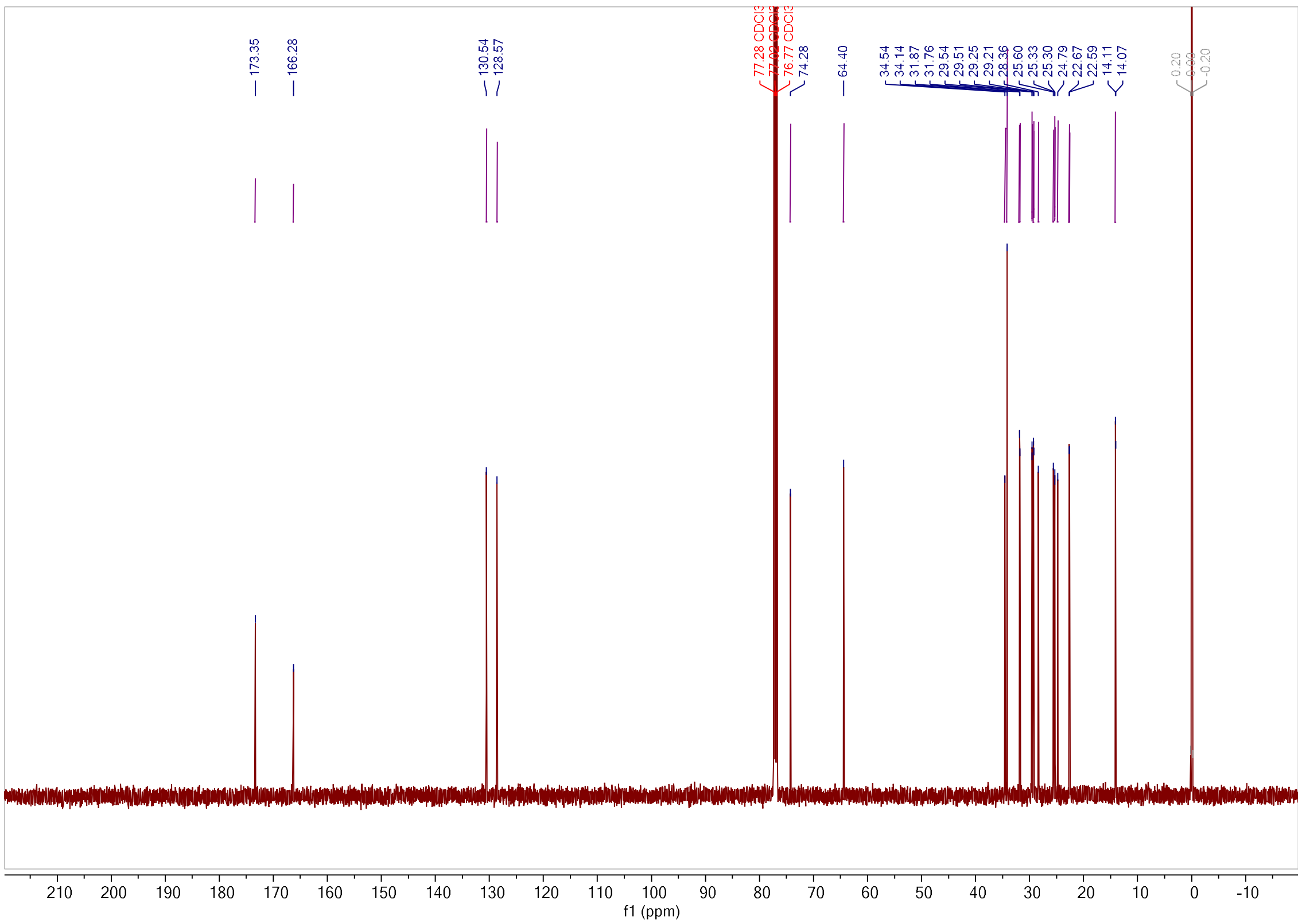

^{13}C-NMR spectrum of compound **8e** ($CDCl_3$, 126 MHz)

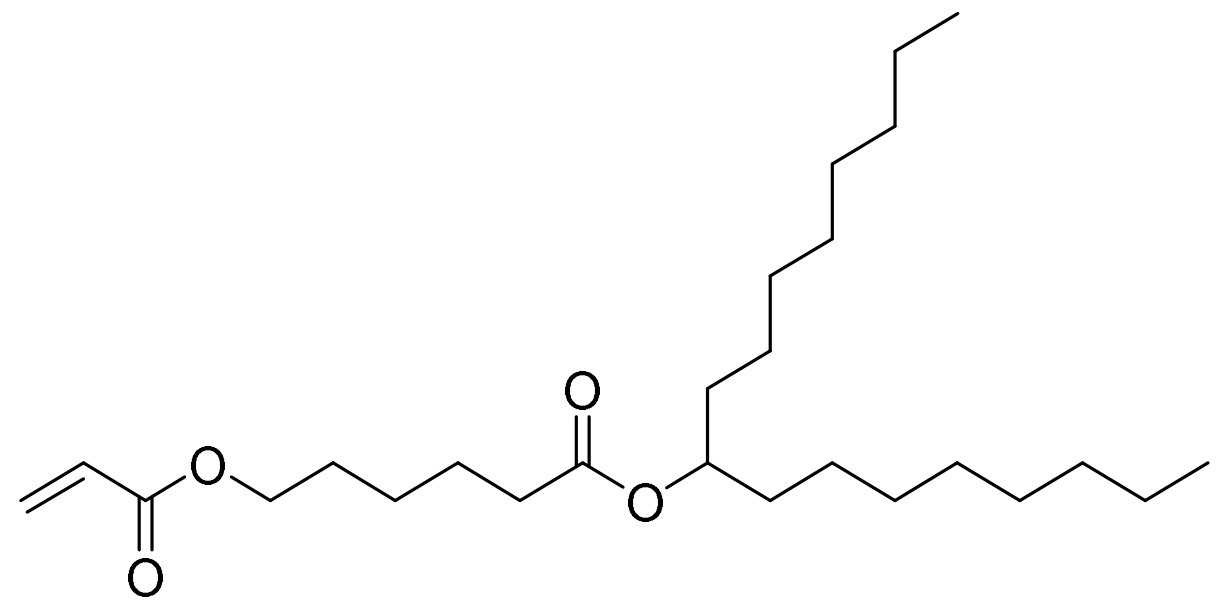

Figure S57. Chemical structure of compound 8f.

^{1}H NMR (400 MHz, $CDCl_3$) δ 6.38 (dd, *J* = 17.3, 1.5 Hz, 1H), 6.10 (dd, *J* = 17.3, 10.4 Hz, 1H), 5.80 (dd, *J* = 10.4, 1.5 Hz, 1H), 4.90 – 4.78 (m, 1H), 4.14 (t, *J* = 6.6 Hz, 2H), 2.29 (t, *J* = 7.5 Hz, 2H), 1.74 – 1.59 (m, 4H), 1.52 – 1.33 (m, 6H), 1.29 – 1.21 (m, 24H), 0.86 (t, *J* = 6.7 Hz, 6H).

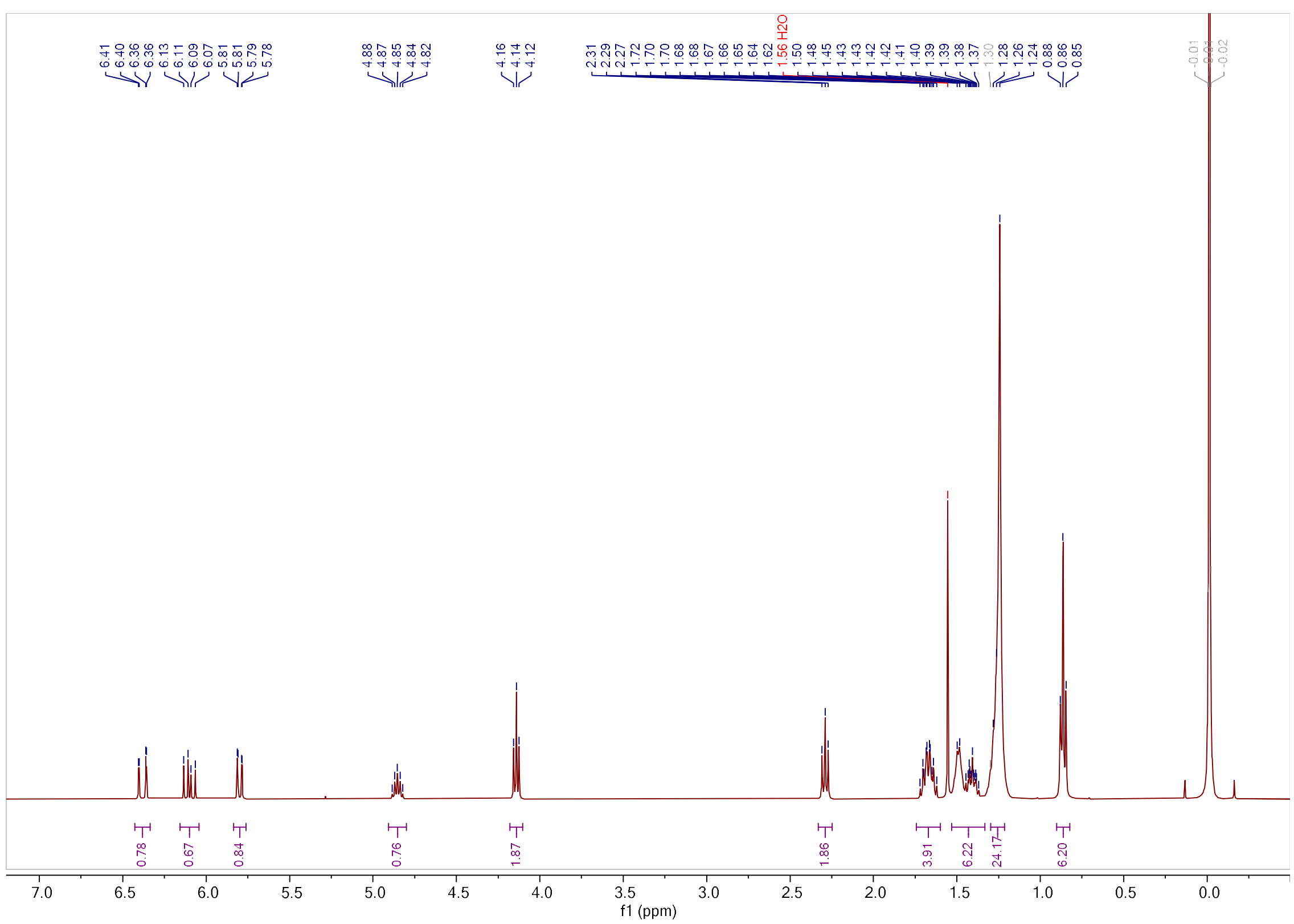

^{1}H NMR spectrum of compound **8f** ($CDCl_3$, 400 MHz)

^{13}C NMR (126 MHz, $CDCl_3$) δ 173.34, 166.27, 130.53, 128.57, 74.28, 64.40, 34.54, 34.14, 31.87, 29.54, 29.51, 29.25, 28.36, 25.60, 25.33, 24.79, 22.67, 14.11.

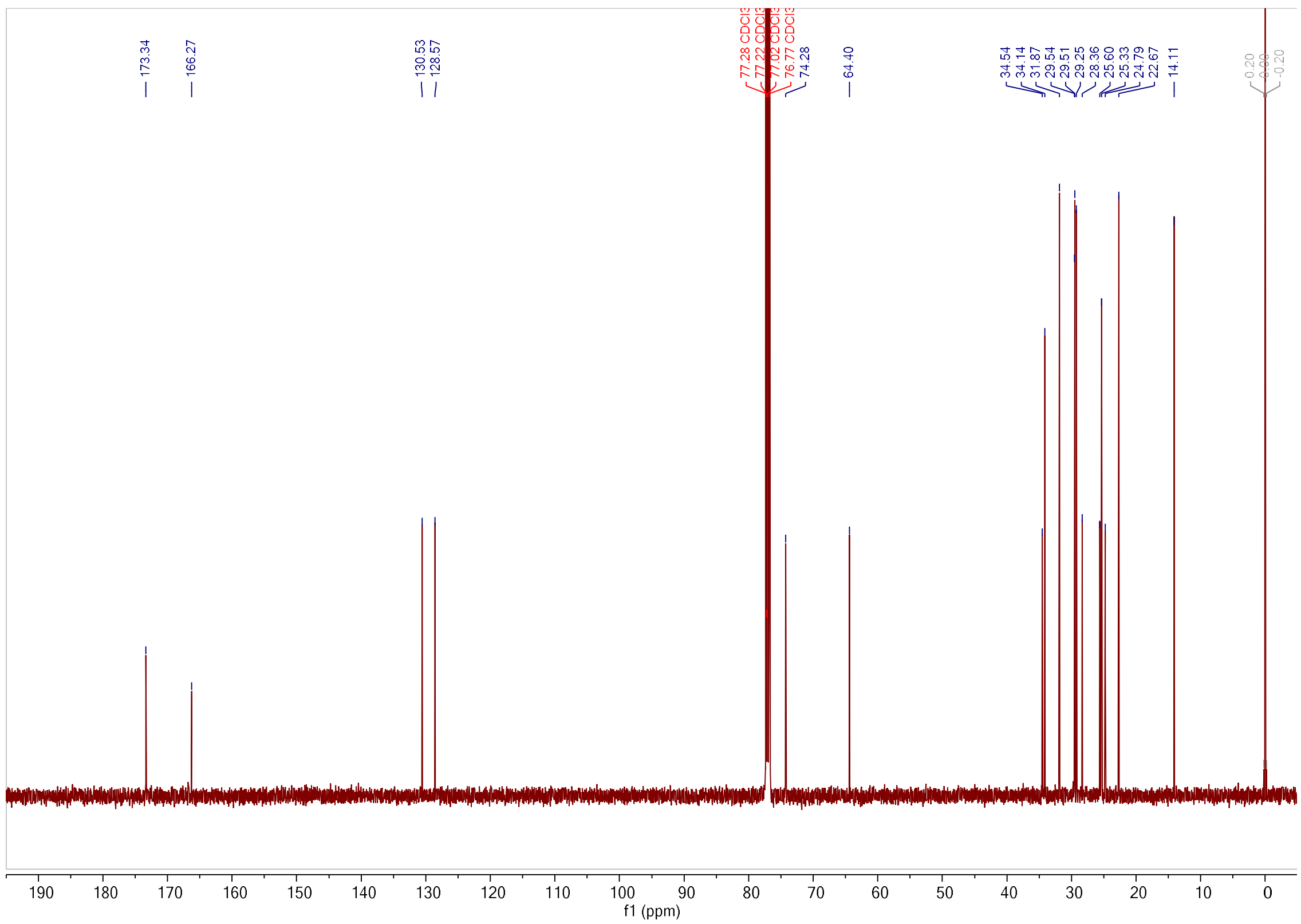

^{13}C-NMR spectrum of compound **8f** ($CDCl_3$, 126 MHz)

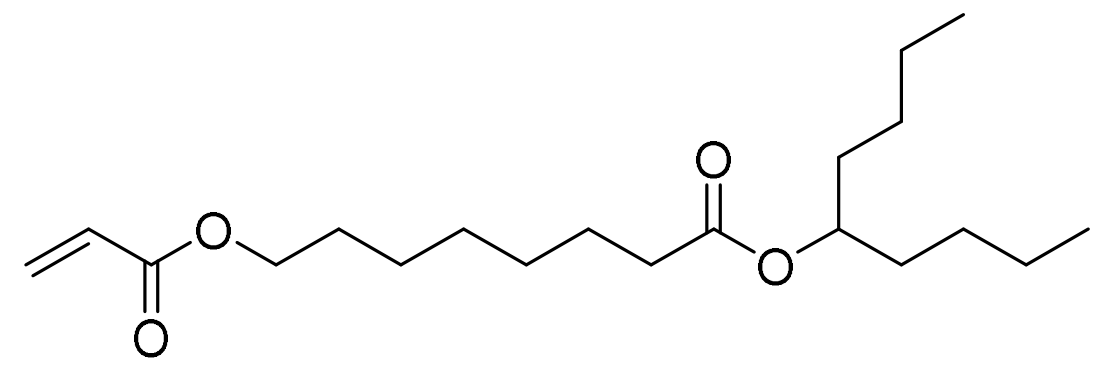

Figure S58. Chemical structure of compound 8g.

^{1}H NMR (500 MHz, $CDCl_3$) δ 6.36 – 6.29 (m, 1H), 6.09 – 6.00 (m, 1H), 5.77 – 5.71 (m, 1H), 4.80 (p, *J* = 6.3 Hz, 1H), 4.08 (t, *J* = 6.7 Hz, 2H), 2.22 (t, *J* = 7.5 Hz, 2H), 1.64 – 1.52 (m, 4H), 1.49 – 1.41 (m, 4H), 1.34 – 1.14 (m, 14H), 0.86 – 0.77 (m, 6H).

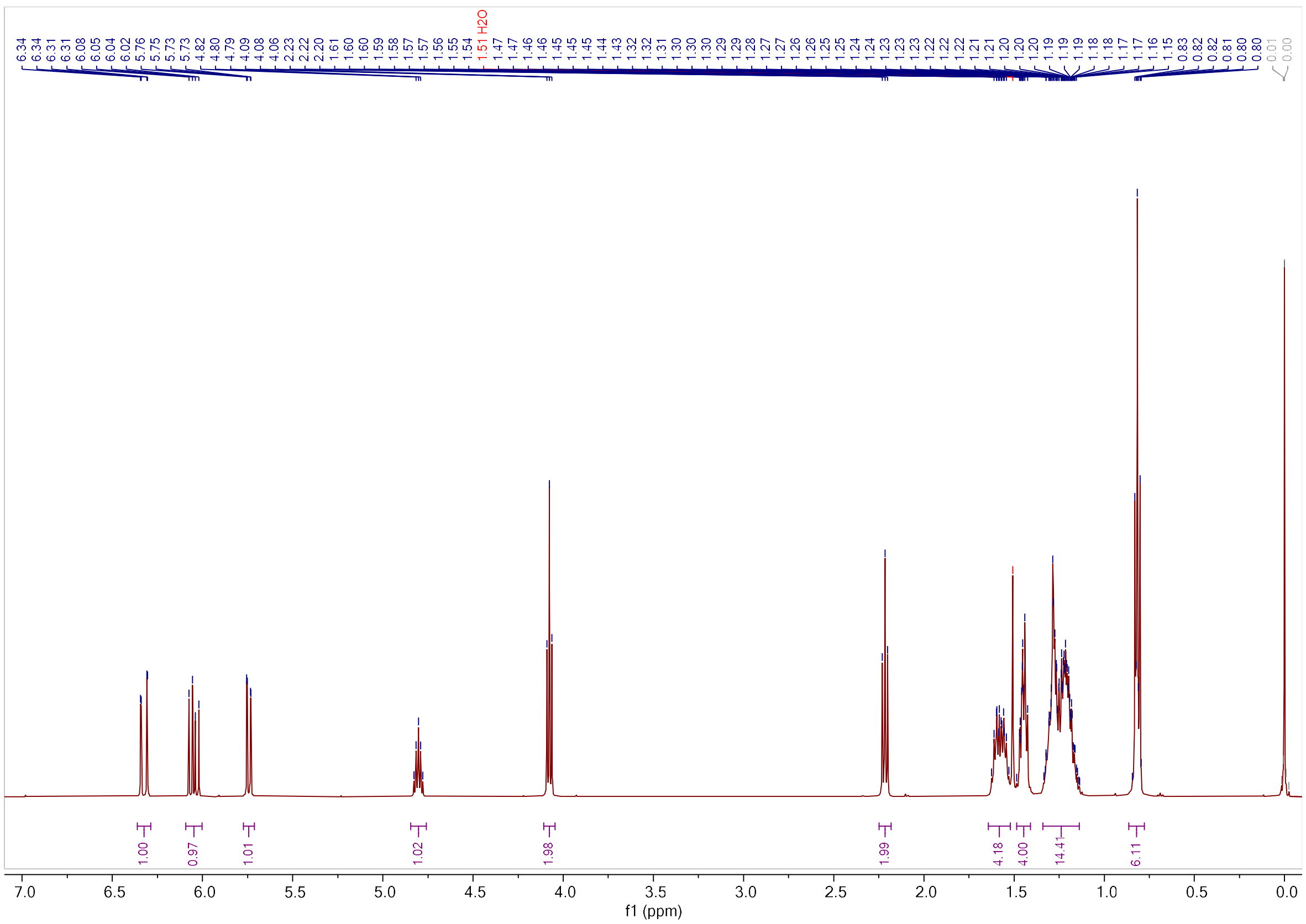

^{1}H NMR spectrum of compound **8g** ($CDCl_3$, 500 MHz)

^{13}C NMR (126 MHz, $CDCl_3$) δ 172.59, 165.31, 129.44, 127.60, 73.08, 63.59, 33.63, 32.84, 28.01, 27.91, 27.54, 26.49, 24.77, 24.02, 21.58, 12.99.

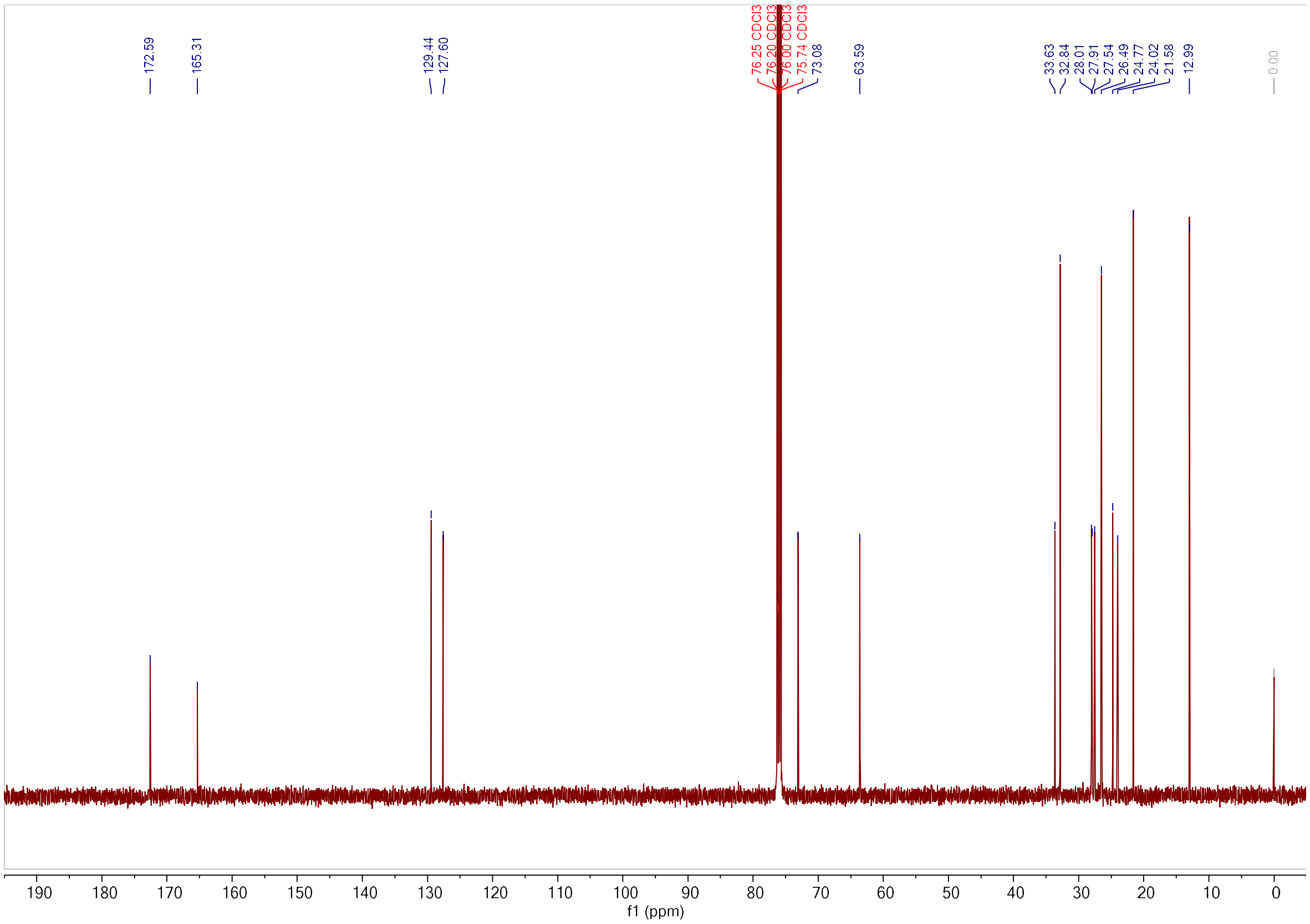

^{13}C-NMR spectrum of compound **8g** ($CDCl_3$, 126 MHz)

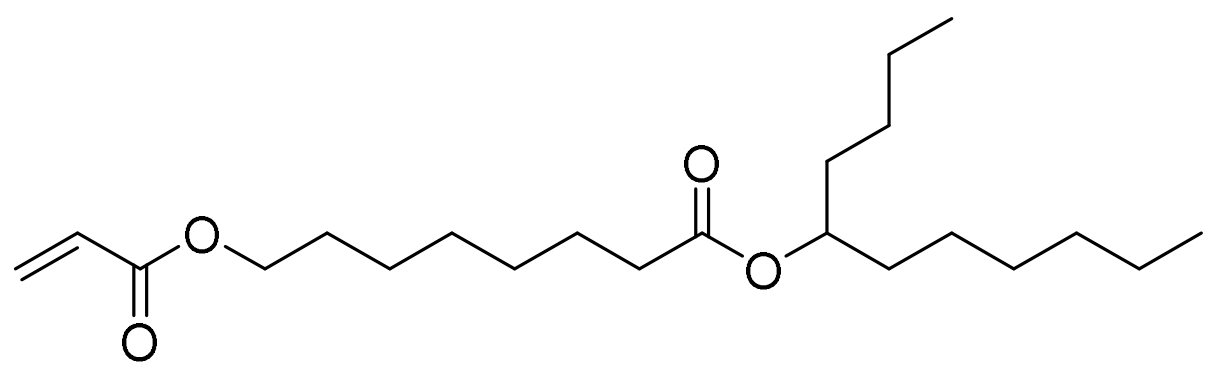

Figure S59. Chemical structure of compound 8h.

1H NMR (500 MHz, $CDCl_3$) δ 6.36 – 6.28 (m, 1H), 6.10 – 6.00 (m, 1H), 5.77 – 5.71 (dd, J = 10.4, 1.5 Hz, 1H), 4.80 (p, J = 6.2 Hz, 1H), 4.08 (t, J = 6.7 Hz, 2H), 2.21 (t, J = 7.5 Hz, 2H), 1.64 – 1.51 (m, 4H), 1.48-1.40 (m, 4H), 1.34 – 1.13 (m, 18H), 0.86 – 0.77 (m, 6H).

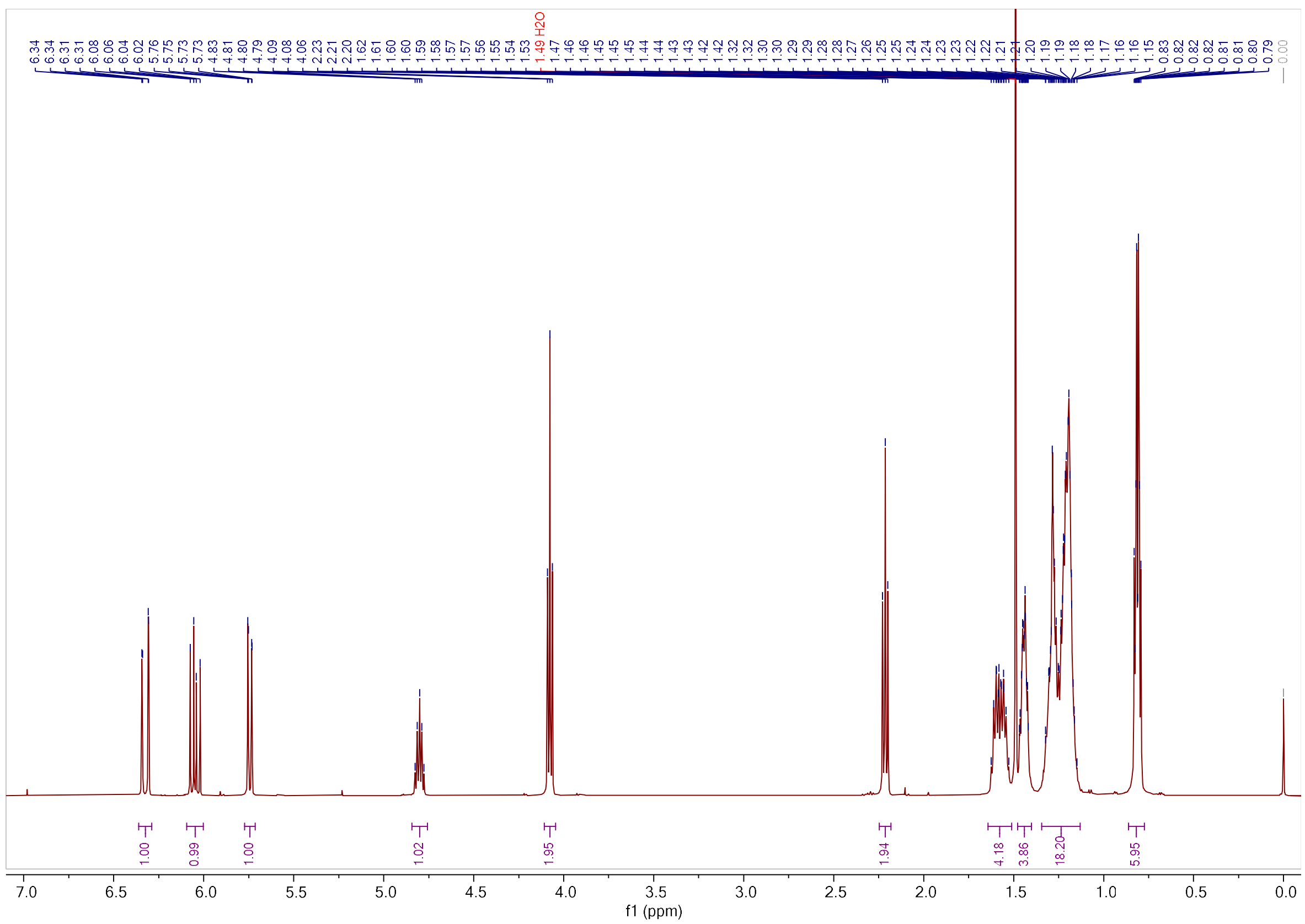

^{1}H NMR spectrum of compound **8h** ($CDCl_3$, 500 MHz)

^{13}C NMR (126 MHz, $CDCl_3$) δ 173.61, 166.35, 130.47, 128.63, 74.14, 64.62, 34.67, 34.18, 33.87, 31.77, 29.21, 29.04, 28.94, 28.57, 27.52, 25.80, 25.30, 25.06, 22.61, 22.59, 14.08, 14.02.

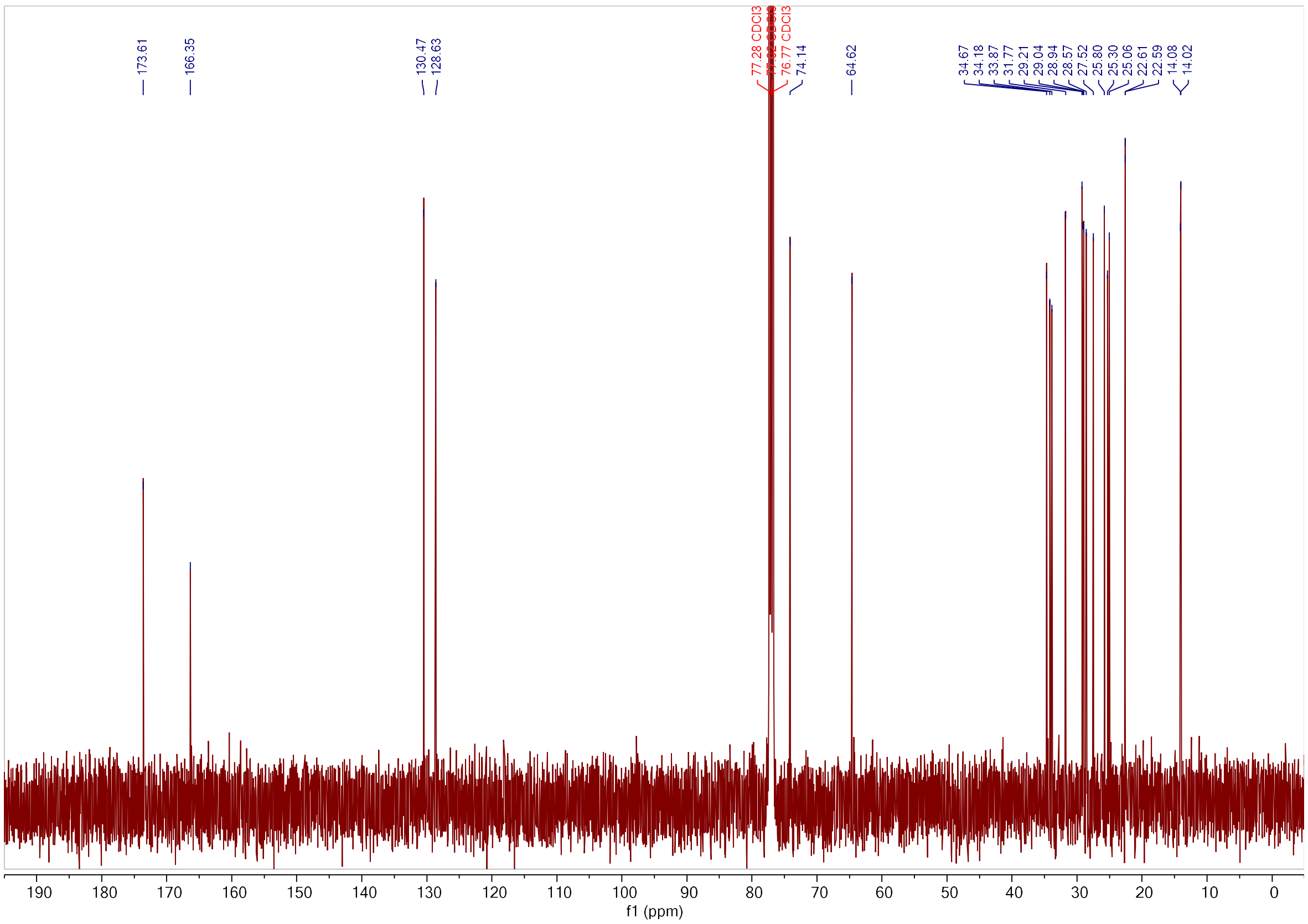

^{13}C-NMR spectrum of compound **8h** ($CDCl_3$, 126 MHz)

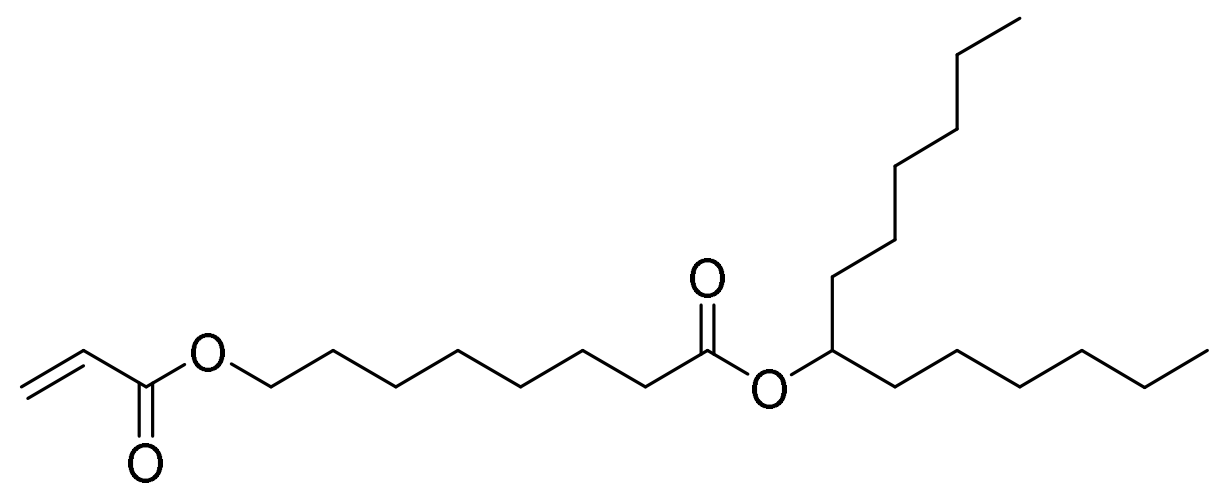

Figure S60. Chemical structure of compound 8i.

^{1}H NMR (500 MHz, $CDCl_3$) δ 6.36 – 6.29 (m, 1H), 6.10 – 6.00 (m, 1H), 5.78 – 5.71 (m, 1H), 4.80 (p, *J* = 6.3 Hz, 1H), 4.08 (t, *J* = 6.7 Hz, 2H), 2.21 (t, *J* = 7.5 Hz, 2H), 1.64 – 1.53 (m, 4H), 1.48 – 1.37 (m, 4H), 1.32 – 1.15 (m, 22H), 0.84 – 0.77 (m, 6H).

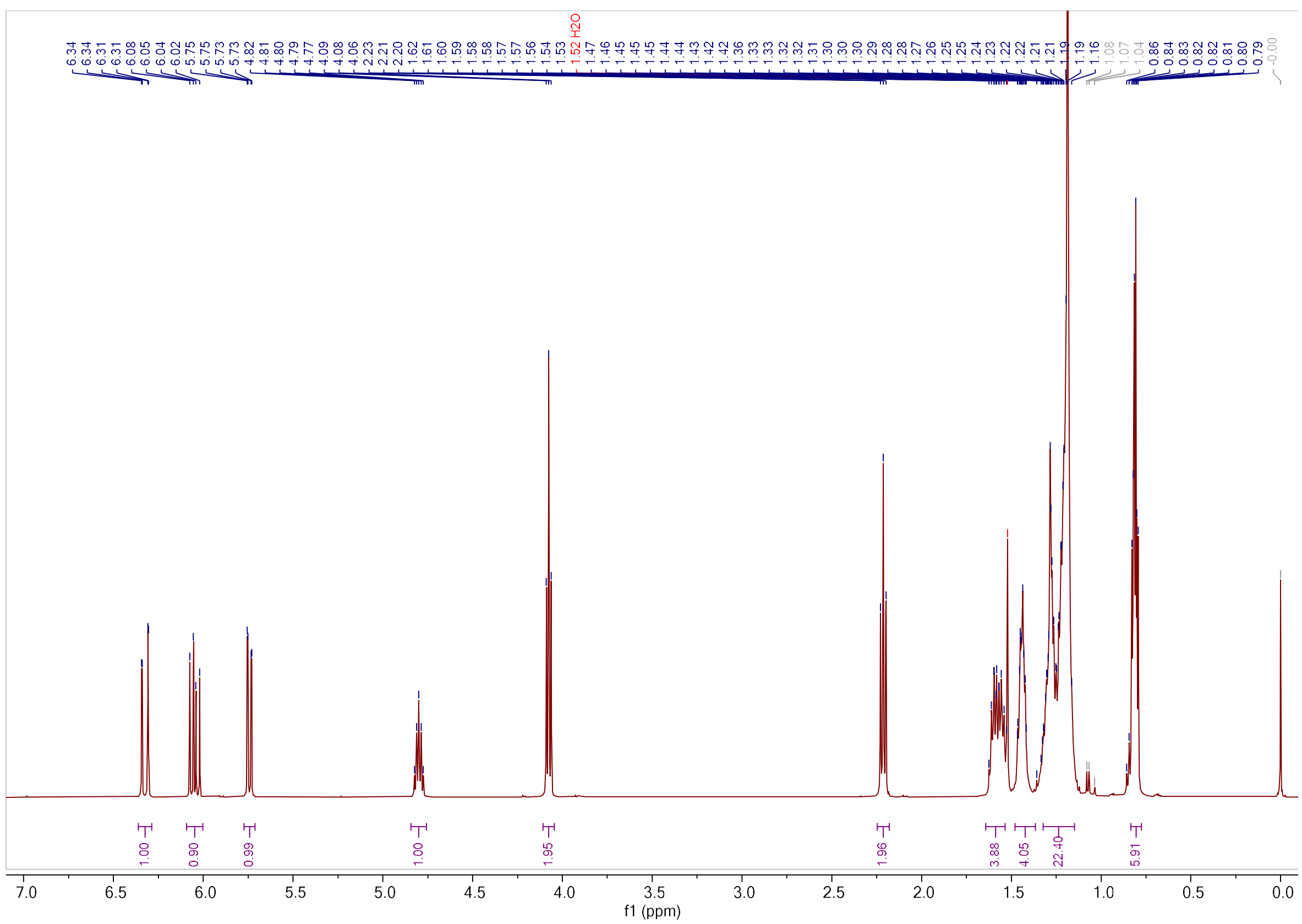

^{1}H NMR spectrum of compound **8i** ($CDCl_3$, 500 MHz)

^{13}C NMR (126 MHz, $CDCl_3$) δ 173.61, 166.33, 130.46, 128.63, 74.13, 64.61, 39.01, 34.66, 34.17, 33.86, 31.87, 29.54, 29.51, 29.24, 29.04, 28.94, 28.57, 27.51, 25.80, 25.71, 25.33, 25.05, 23.35, 22.67, 22.61, 14.11, 14.02.

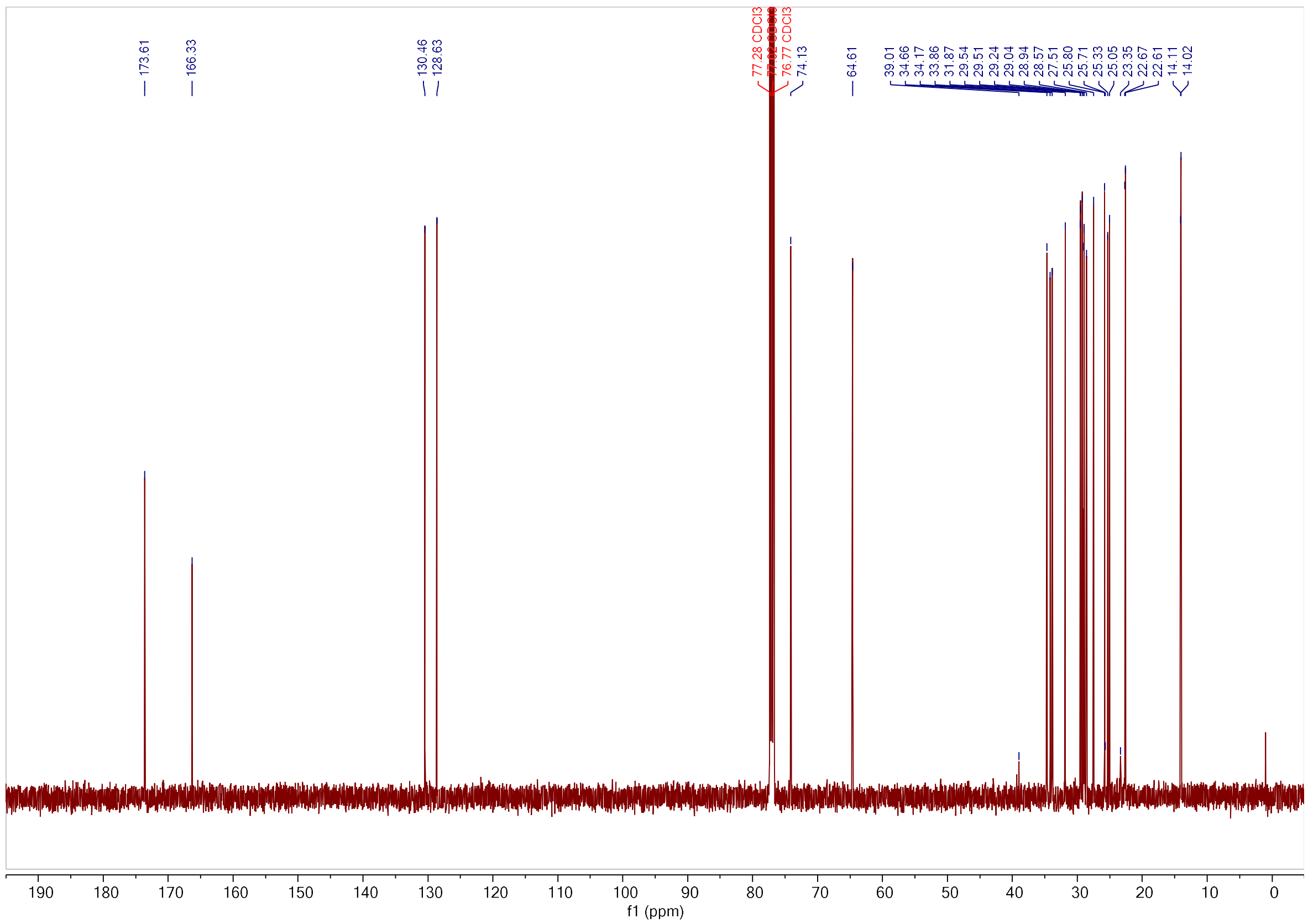

^{13}C-NMR spectrum of compound **8i** ($CDCl_3$, 126 MHz)

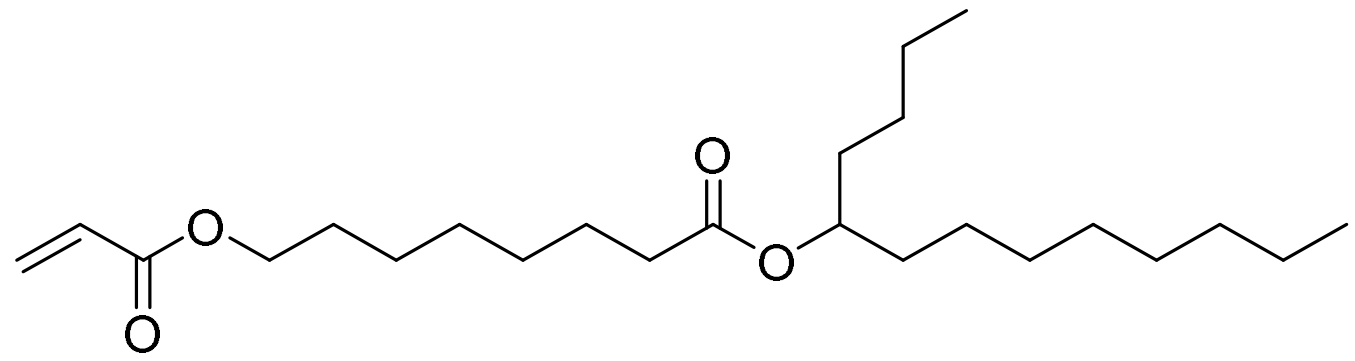

Figure S61. Chemical structure of compound 8j.

^{1}H NMR (500 MHz, $CDCl_3$) δ 6.37 – 6.29 (m, 1H), 6.10 – 6.00 (m, 1H), 5.79 – 5.70 (m, 1H), 4.80 (p, *J* = 6.3 Hz, 1H), 4.08 (t, *J* = 6.7 Hz, 2H), 2.21 (t, *J* = 7.5 Hz, 2H), 1.67 – 1.52 (m, 4H), 1.48 – 1.40 (m, 4H), 1.37 – 1.12 (m, 20H), 0.85 – 0.77 (m, 6H).

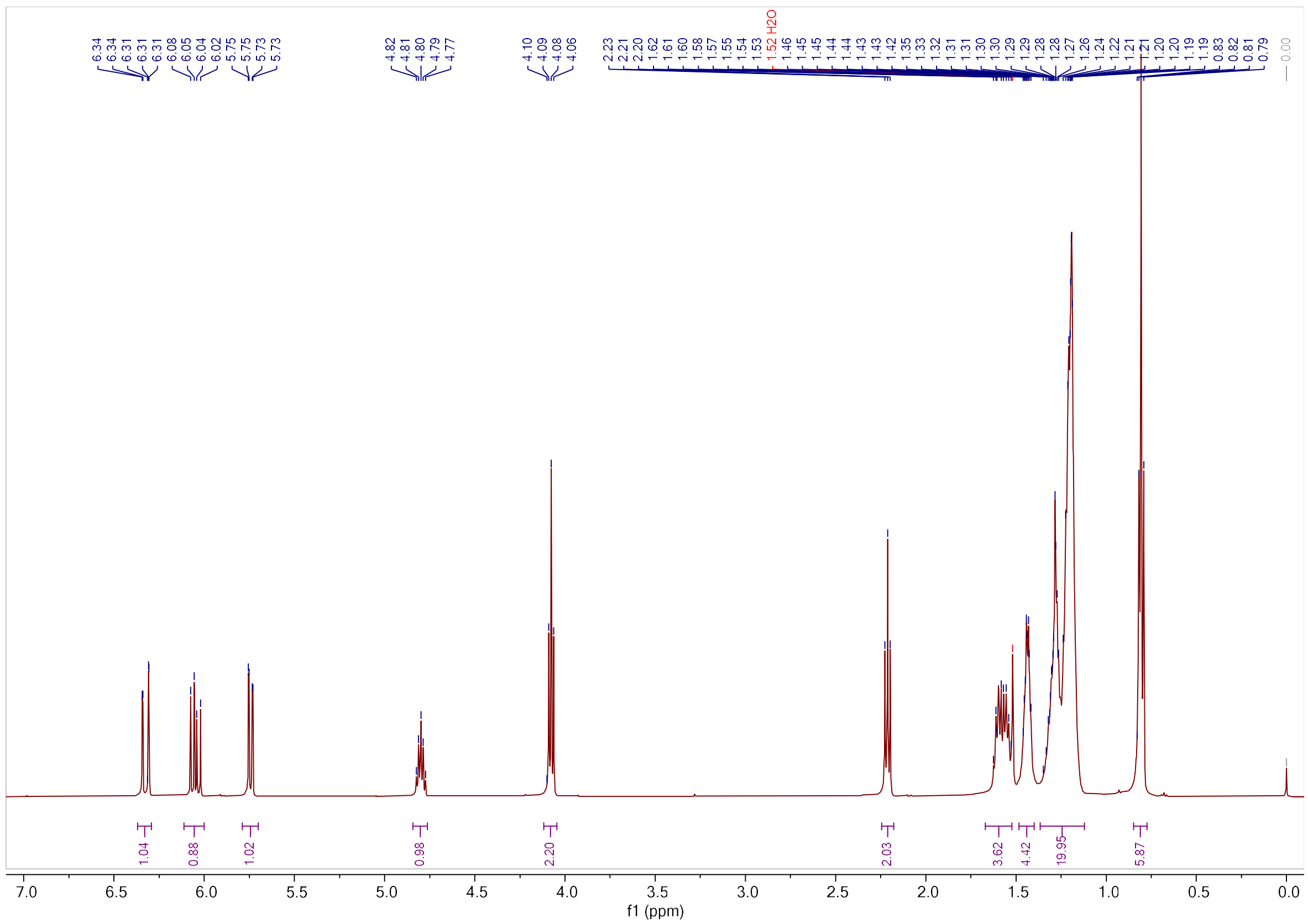

^{1}H NMR spectrum of compound **8j** ($CDCl_3$, 500 MHz)

^{13}C NMR (126 MHz, $CDCl_3$) δ 173.61, 166.33, 130.46, 128.63, 74.13, 64.61, 39.01, 34.66, 34.17, 33.86, 31.87, 29.54, 29.51, 29.25, 29.04, 28.94, 28.57, 27.51, 25.80, 25.71, 25.33, 25.05, 23.35, 22.67, 22.61, 14.11, 14.02.

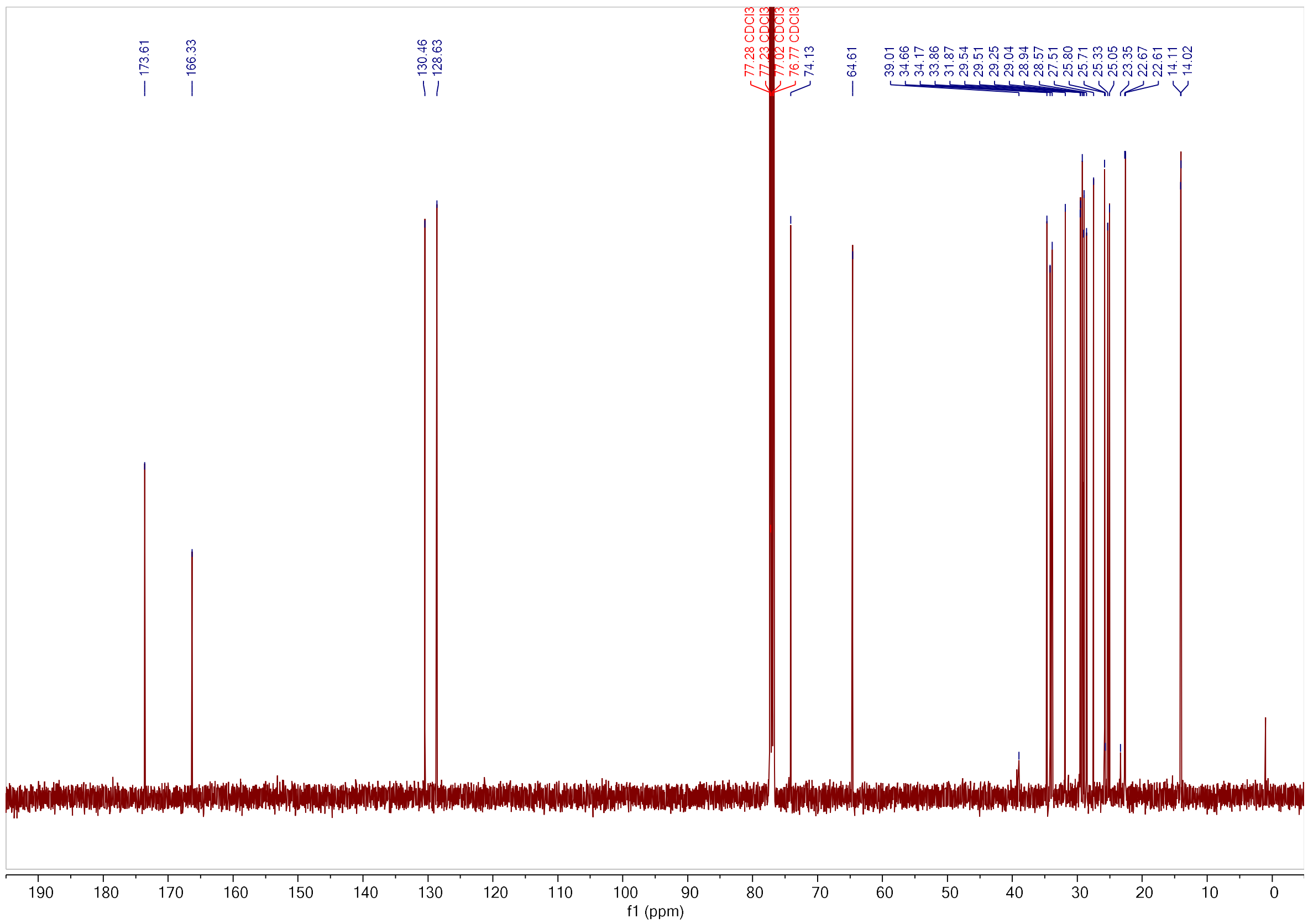

^{13}C-NMR spectrum of compound **8j** ($CDCl_3$, 126 MHz)

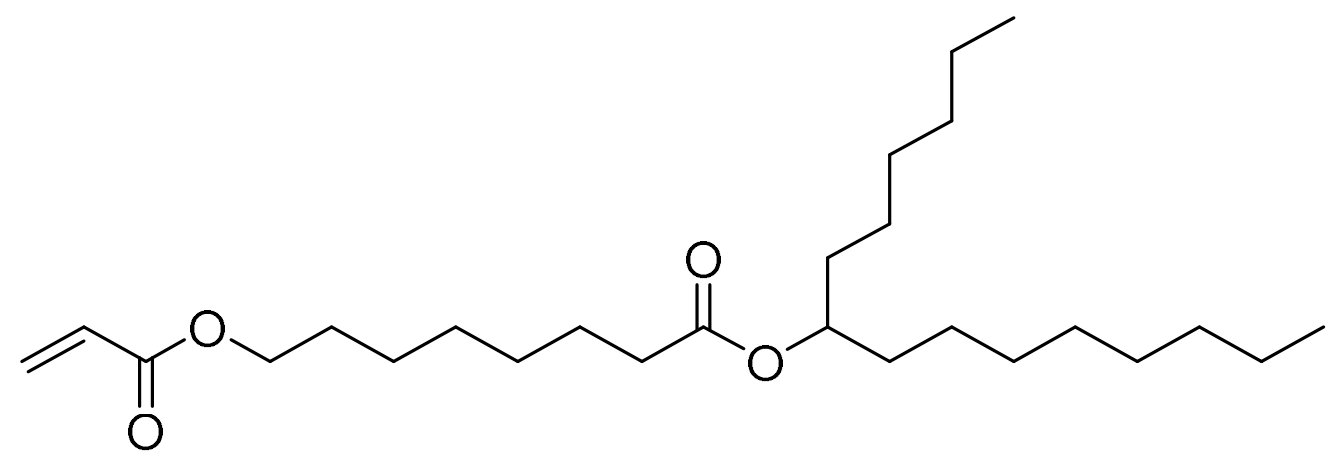

Figure S62. Chemical structure of compound 8k.

^1H NMR (500 MHz, $CDCl_3$) δ 6.36 – 6.29 (m, 1H), 6.09 – 6.00 (m, 1H), 5.76 – 5.72 (m, 1H), 4.80 (p, *J*=6.5 Hz, 1H), 4.08 (t, *J* = 6.7 Hz, 2H), 2.21 (t, *J* = 7.5 Hz, 2H), 1.64 – 1.52 (m, 4H), 1.47 – 1.38 (m, 4H), 1.32 – 1.14 (m, 26H), 0.84 – 0.77 (m, 6H).

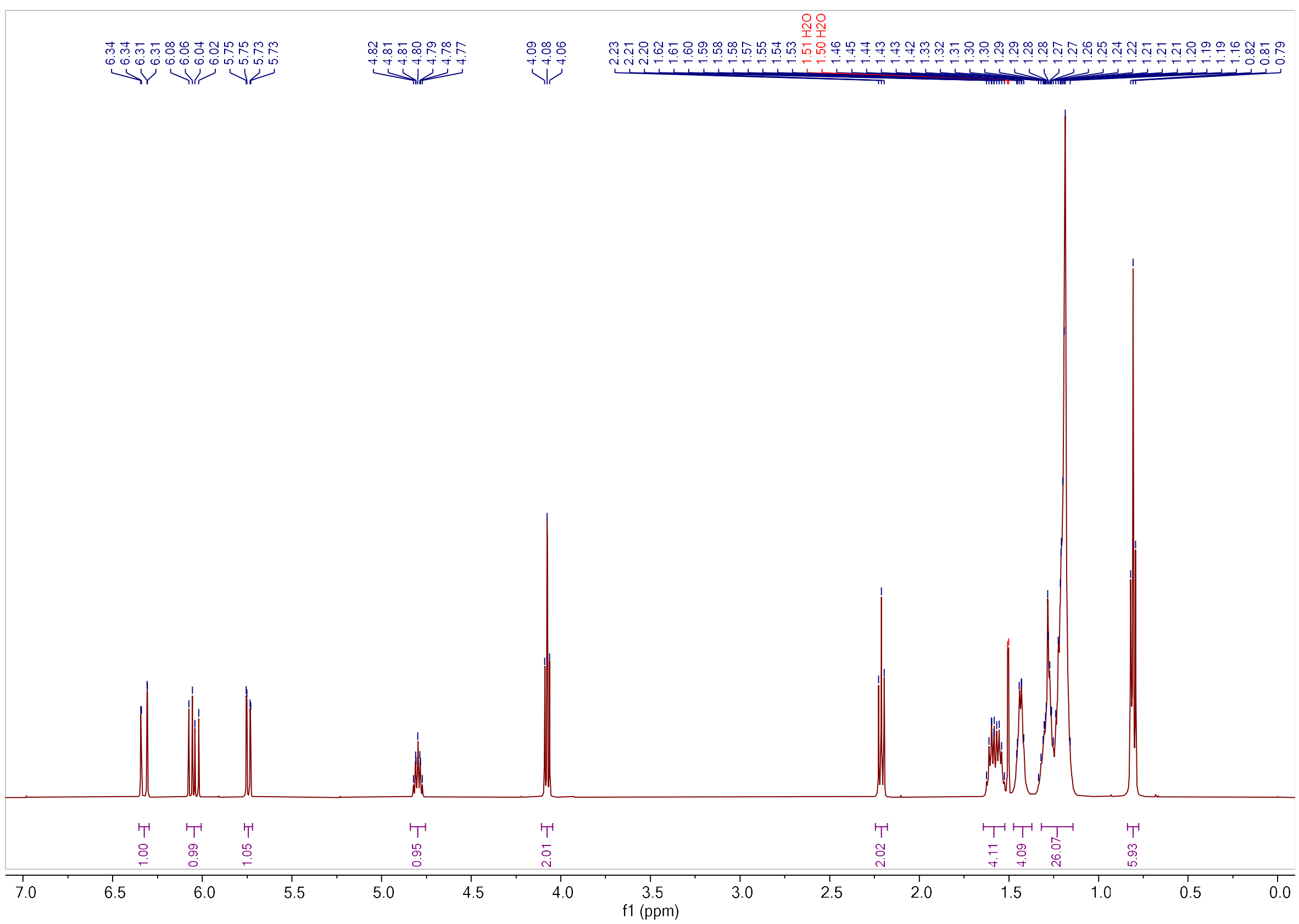

^1H NMR spectrum of compound **8k** ($CDCl_3$, 500 MHz)

^{13}C NMR (126 MHz, $CDCl_3$) δ 173.60, 166.33, 130.46, 128.63, 74.16, 64.62, 34.67, 34.17, 31.87, 31.76, 29.54, 29.51, 29.25, 29.21, 29.05, 28.95, 28.57, 25.80, 25.33, 25.29, 25.06, 22.67, 22.59, 14.11, 14.08.

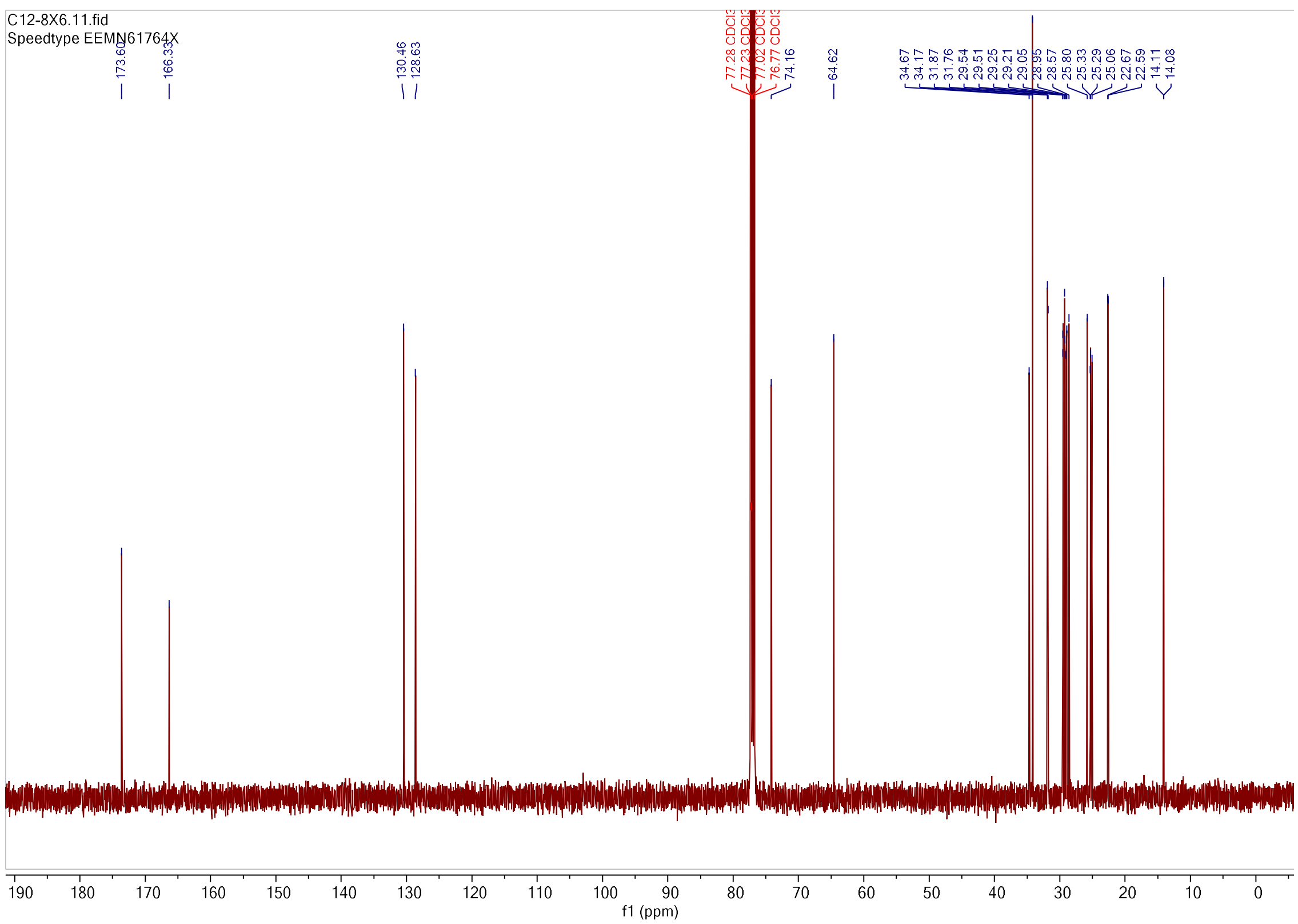

^{13}C-NMR spectrum of compound **8k** ($CDCl_3$, 126 MHz)

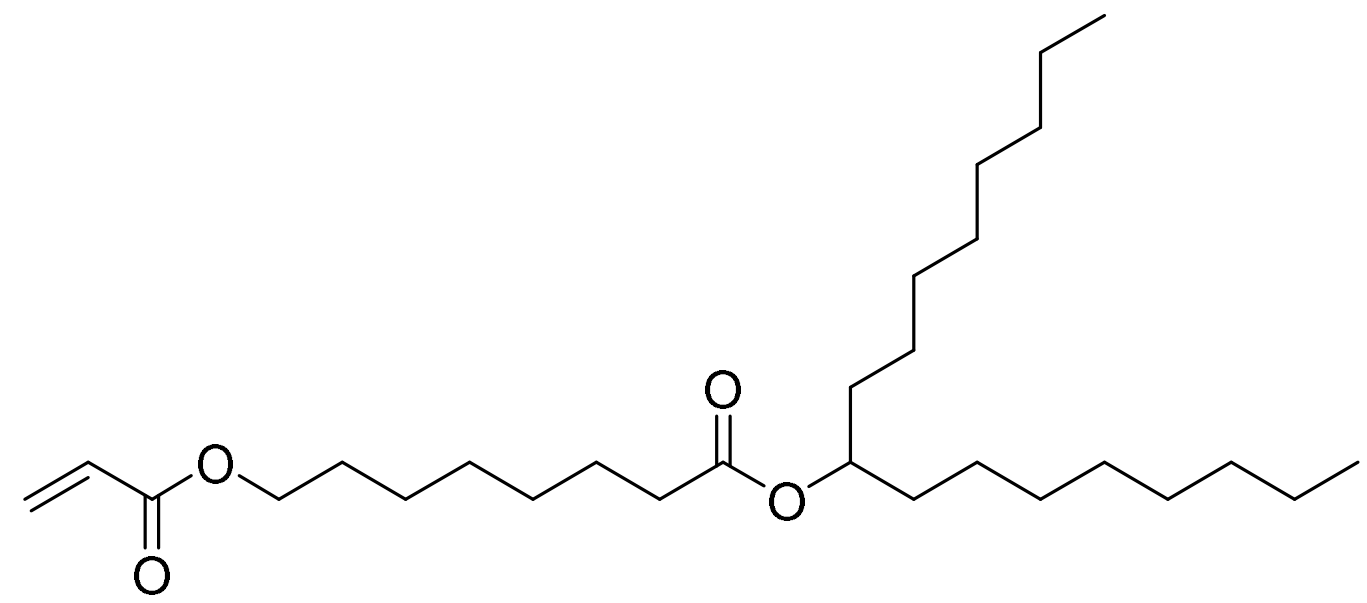

Figure S63. Chemical structure of compound 8l.

^{1}H NMR (500 MHz, $CDCl_3$) δ 6.43 – 6.36 (m, 1H), 6.17 – 6.07 (m, 1H), 5.85 – 5.78 (m, 1H), 4.87 (p, *J* = 6.2 Hz, 1H), 4.15 (t, *J* = 6.7 Hz, 2H), 2.28 (t, *J* = 7.5 Hz, 2H), 1.70 – 1.59 (m, 4H), 1.52-1.41 (m, 4H), 1.36 – 1.23 (m, 30H), 0.90 – 0.85 (m, 6H).

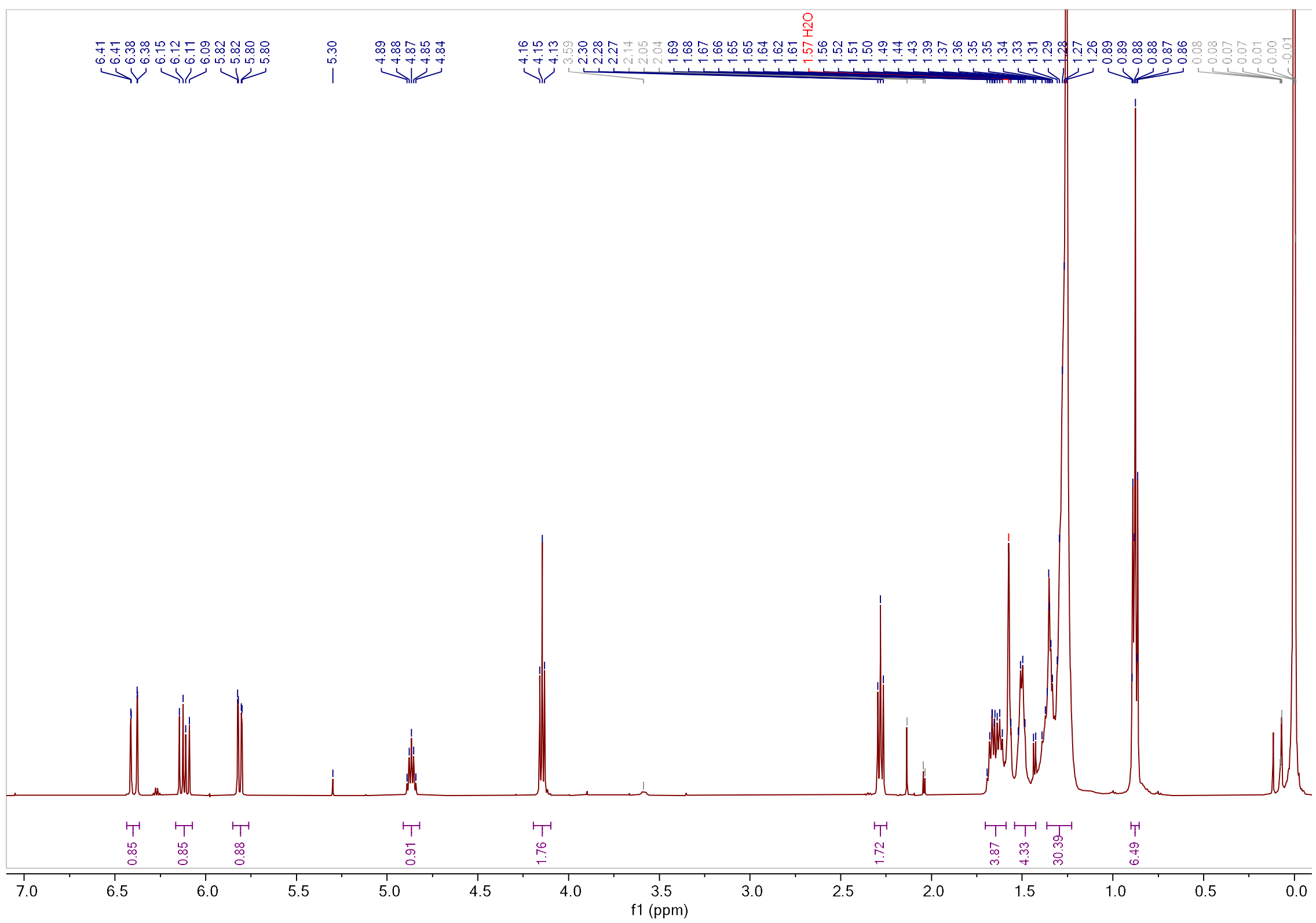

^{1}H NMR spectrum of compound **8l** ($CDCl_3$, 500 MHz)

^{13}C NMR (126 MHz, $CDCl_3$) δ 173.61, 166.34, 130.46, 128.63, 74.17, 64.62, 37.51, 34.67, 34.16, 31.90, 31.87, 29.73, 26.61, 29.55, 29.52, 29.30, 29.25, 29.05, 28.95, 28.57, 25.80, 25.67, 25.33, 25.06, 22.68, 14.11.

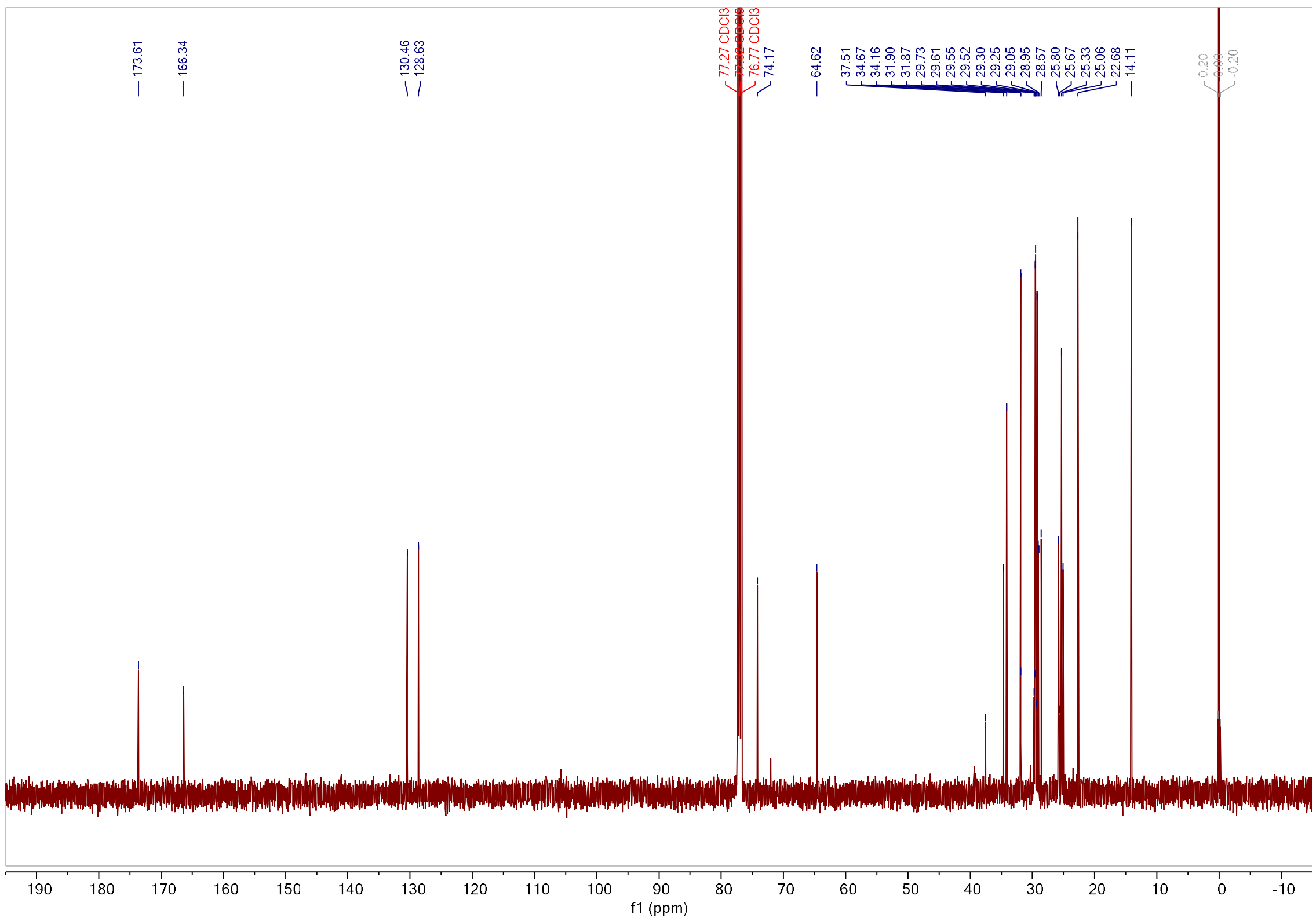

^{13}C-NMR spectrum of compound **8l** ($CDCl_3$, 126 MHz)

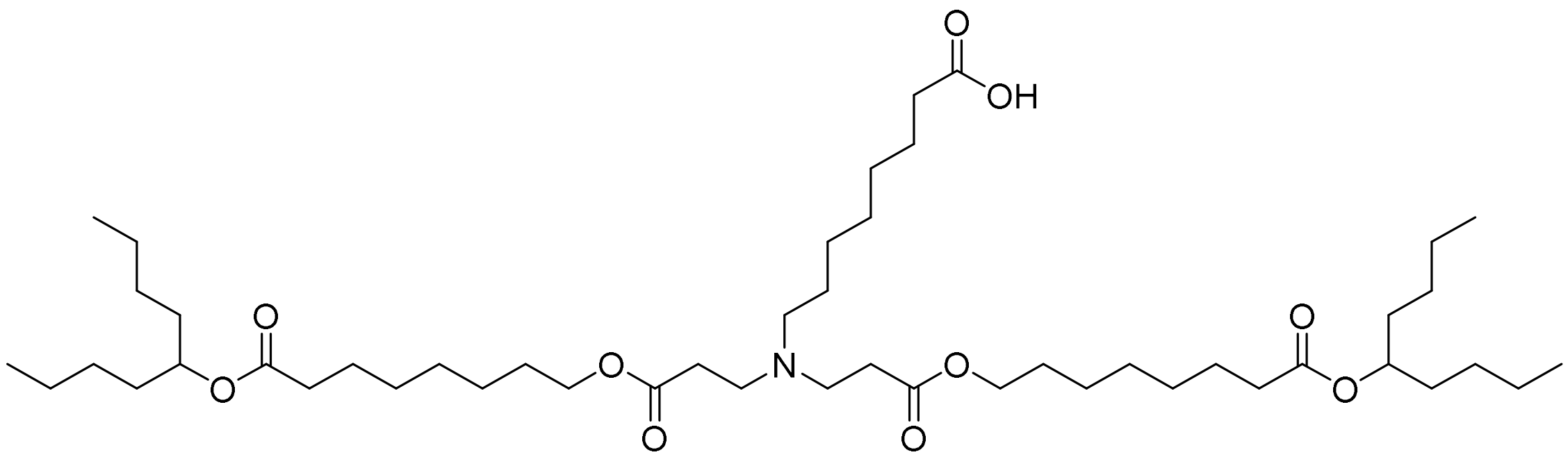

Figure S64. Chemical structure of compound A1.

^{1}H NMR (500 MHz, $CDCl_3$) δ 4.80 (p, *J* = 6.3 Hz, 2H), 4.00 (t, *J* = 6.8 Hz, 4H), 3.09 – 2.79 (m, 4H), 2.75 – 2.43 (m, 6H), 2.27 – 2.20 (m, 6H), 1.59 – 1.52 (m, 11H), 1.49 – 1.42 (m, 9H), 1.29 – 1.17 (m, 34H), 0.84 – 0.80 (m, 12H).

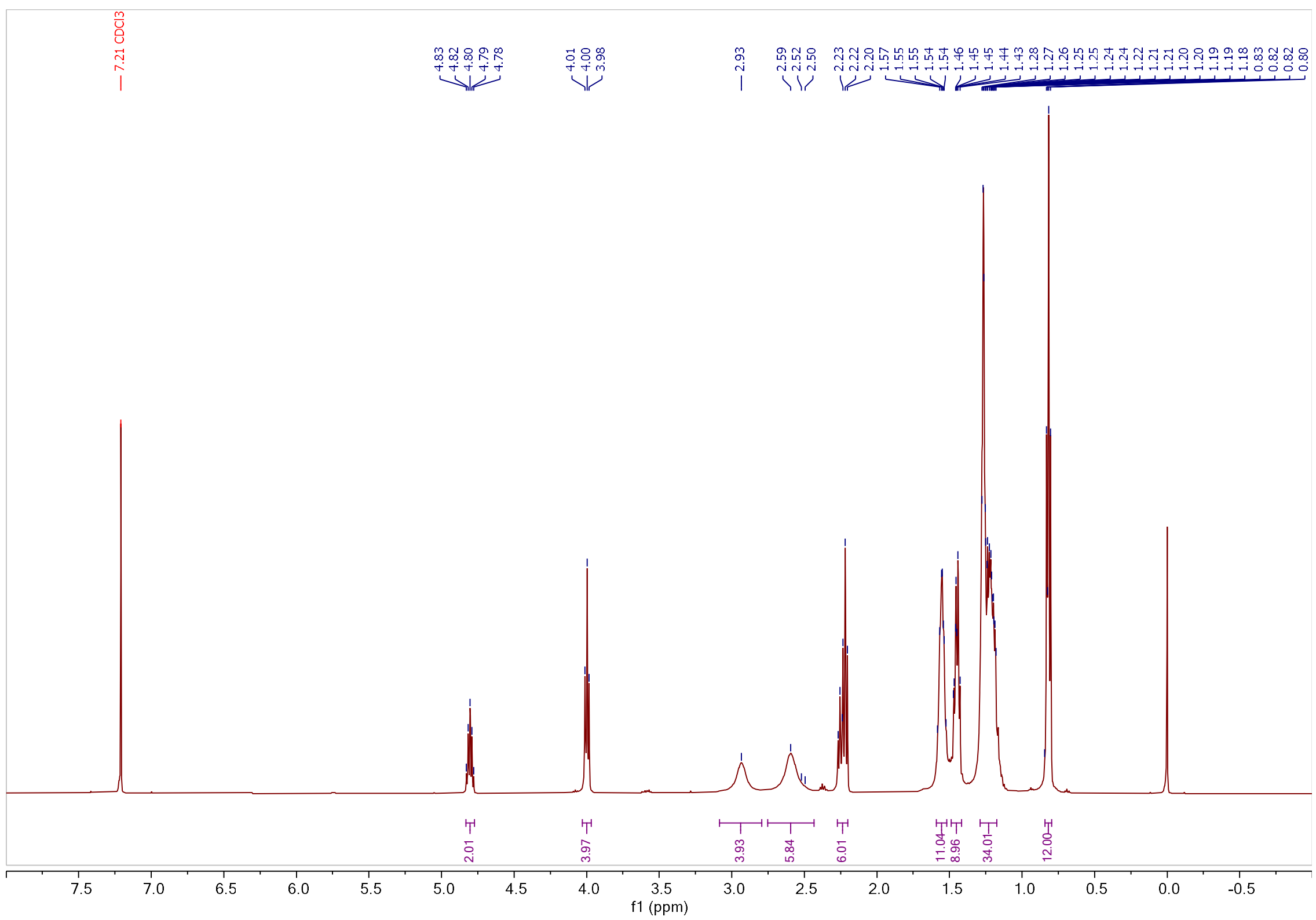

^{1}H NMR spectrum of compound **A1** ($CDCl_3$, 500 MHz)

^{13}C NMR (126 MHz, $CDCl_3$) δ 177.76, 173.71, 172.11, 74.16, 64.83, 53.40, 48.77, 34.64, 34.00, 33.84, 29.03, 28.93, 28.90, 28.52, 27.50, 26.93, 25.75, 25.04, 24.67, 22.60, 14.01.

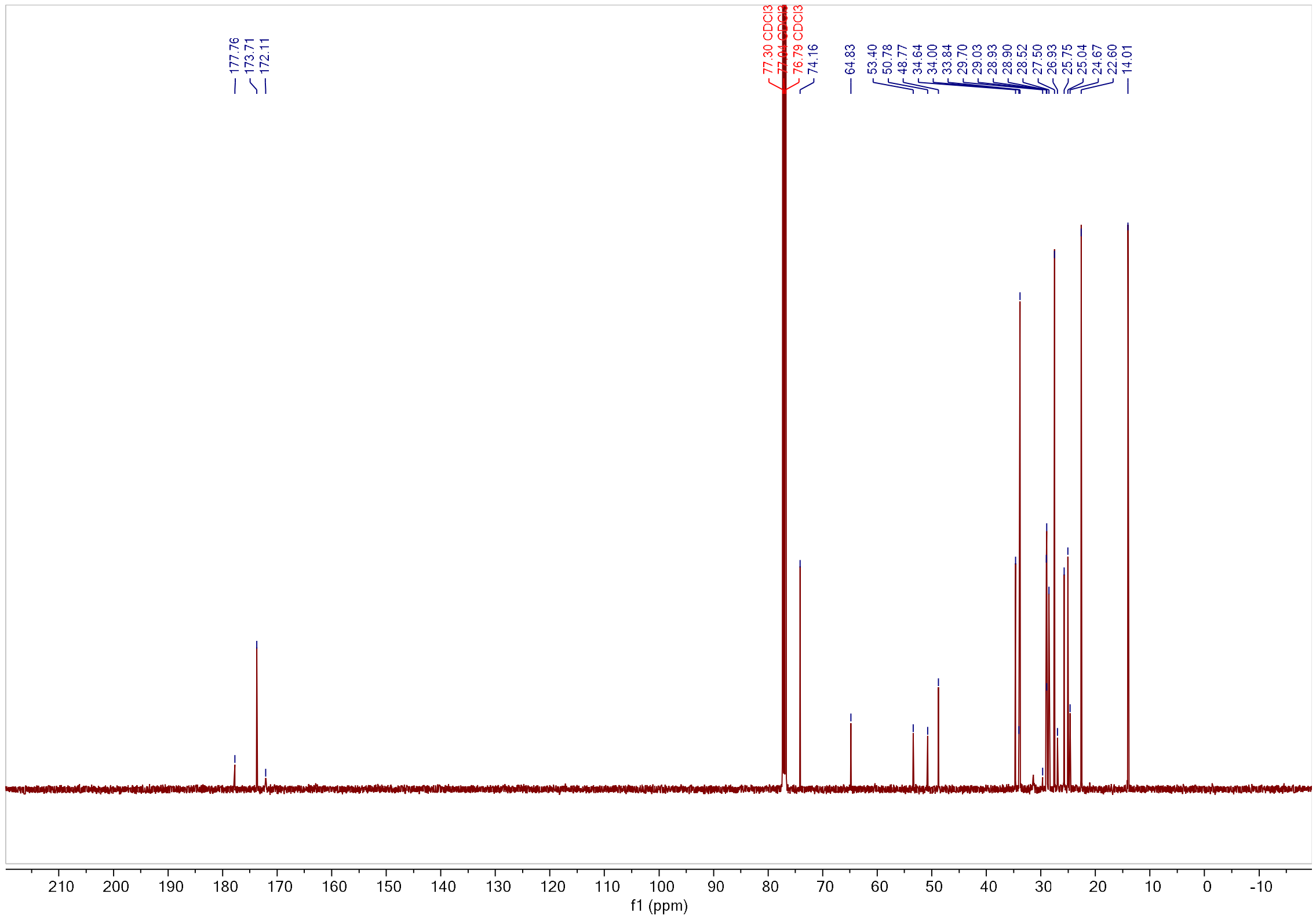

^{13}C-NMR spectrum of compound **A1** ($CDCl_3$, 126 MHz)

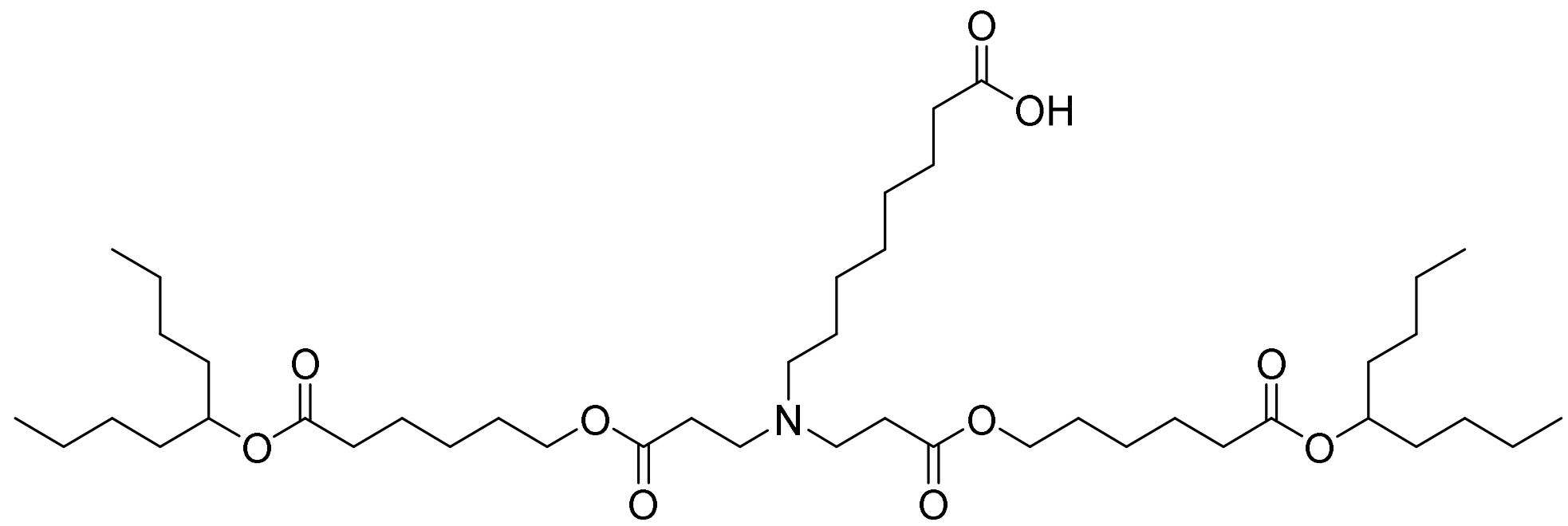

Figure S65. Chemical structure of compound A13.

^1H NMR (500 MHz, $CDCl_3$) δ 4.80 (p, *J* = 6.3 Hz, 2H), 4.01 (t, *J* = 6.7 Hz, 4H), 3.10 – 2.88 (m, 4H), 2.80 – 2.50 (m, 6H), 2.28 – 2.20 (m, 6H), 1.64 – 1.51 (m, 12H), 1.49 – 1.41 (m, 8H), 1.34 – 1.16 (m, 26H), 0.82 (t, *J* = 7.0 Hz, 12H).

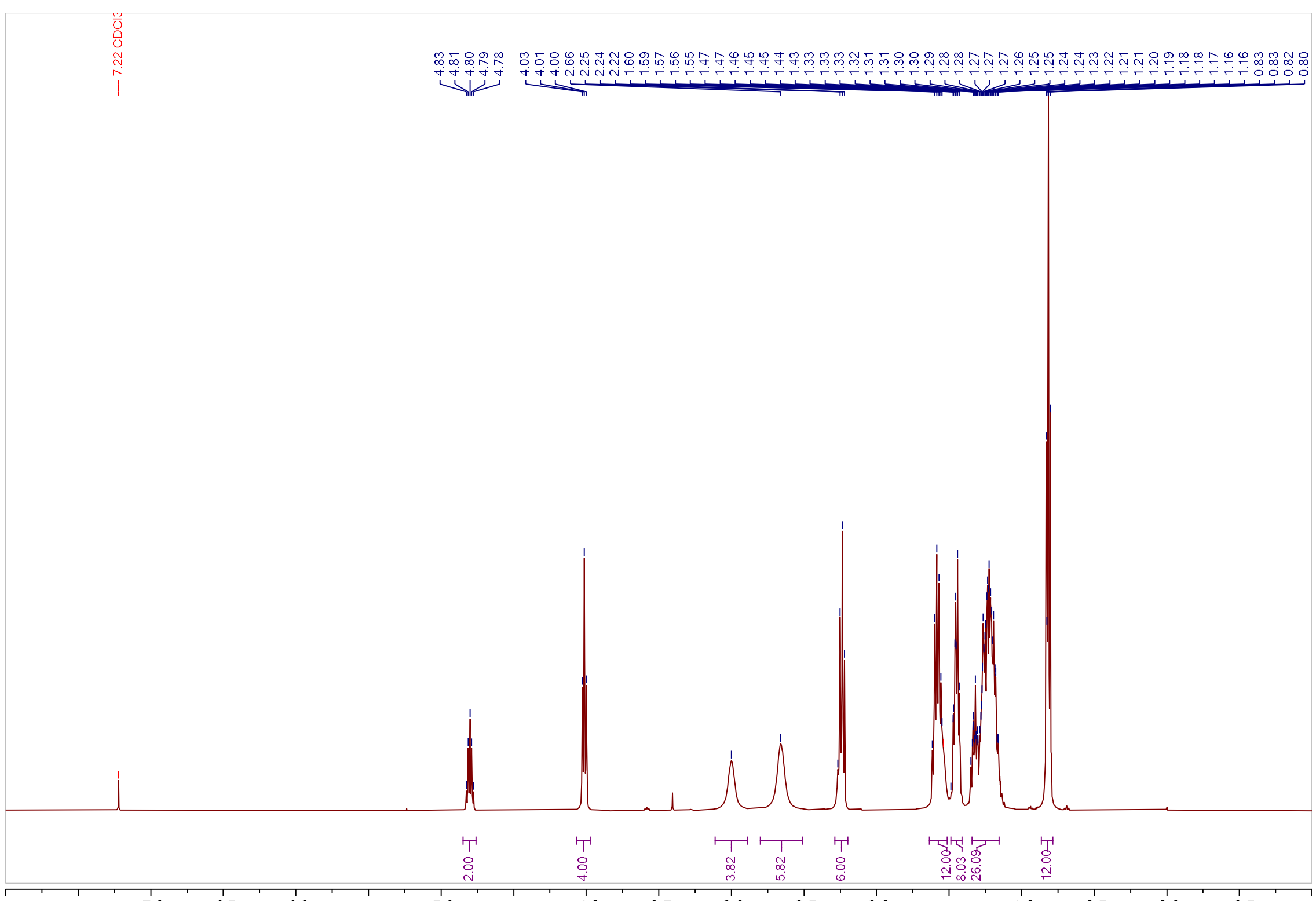

^1H NMR spectrum of compound **A13** ($CDCl_3$, 500 MH

^{13}C NMR (126 MHz, $CDCl_3$) δ 177.97, 173.39, 171.26, 74.26, 64.87, 53.26, 48.53, 34.43, 34.06, 33.80, 30.39, 28.83, 28.80, 28.22, 27.48, 26.77, 25.45, 24.67, 24.63, 22.57, 14.00.

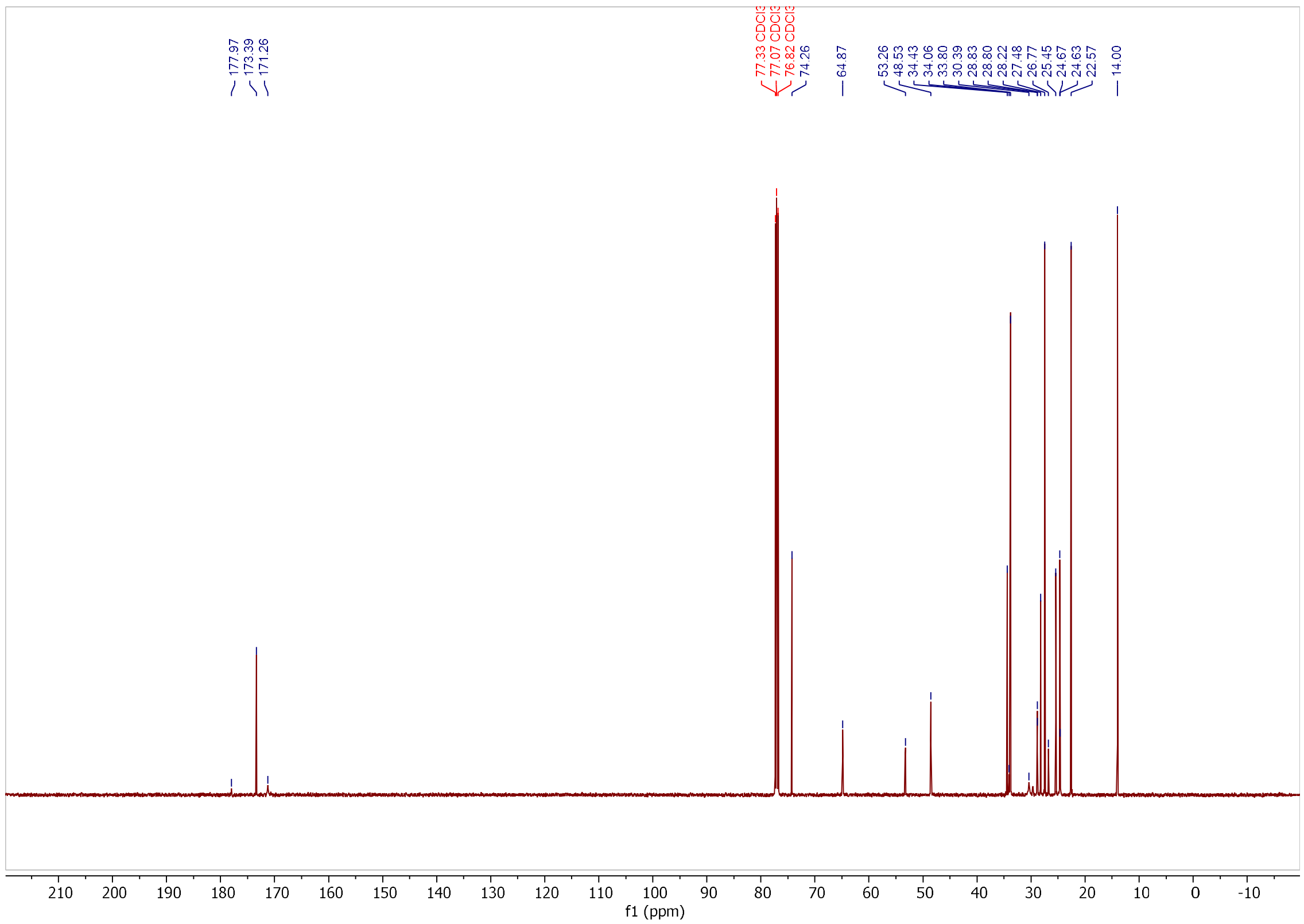

^{13}C-NMR spectrum of compound **A13** ($CDCl_3$, 126 MHz)

4. *In vitro* mRNA delivery experiments with LNPs

4.1 General procedure for preparing LNPs

LNPs were prepared by mixing lipids in ethanol with mRNA dissolved in an acidic aqueous. The mixing was either performed via pipetting or using a microfluidic device. LNPs were dialyzed against PBS, unless otherwise indicated. The details of the various different LNP preparation methods and stock solutions used to make LNPs are described below.

4.1.1 Lipid stock solution: Lipid stock solutions of DLin-MC3-DMA, SM-102, ALC-0315, 4A3-SC8, cKK-E12, C12-200, DOPE, DSPC, cholesterol, and DMG-PEG2K were prepared at a concentration of 10 mg/mL in ethanol. The S-lipids were dissolved in ethanol at a concentration of 15 mM. All stock solutions were sterile-filtered through a 0.22 µm membrane and confirmed to be endotoxin-free by the LAL assay (Section 7.13).

4.1.2 Lipid formulations: To prepare mixtures of lipids in ethanol, the appropriate volumes of stock solutions were added to a 200 µL PCR tube or 1.5 mL Eppendorf tube. Lipid compositions: either S-lipids (or cKK-E12, C12-200), DOPE, cholesterol, and DMG-PEG2K at a molar ratio of 35.9:24.2:38.7:1.2. DLin-MC3-DMA or SM-102, DSPC, cholesterol, and DMG-PEG2K at a molar ratio of 50:10:38.5:1.5, the latter formulation reflecting the formulation used in the FDA-approved drug Onpattro and Spikevax[3]. ALC-0315, DSPC, cholesterol, and DMG-PEG2K at a molar ratio of 46.3 : 9.4 : 42.7 : 1.6, reflecting the formulation used in the FDA-approved drug Comirnaty[3]. 4A3-SC8 formulation solution was prepared according to the protocol from Omo-Lamai et al and had lipid compositions composed of 4A3-SC8, cholesterol, DOPE, and DMG-PEG2K at a molar ratio of 50:38.5:10:1.5[4,5]. As an additional control, a Dlin formulation equivalent to the S-lipids formulation (named as Dlin eq) was prepared. The lipid composition contained Dlin, DOPE, cholesterol, and DMG-PEG2K at a molar ratio of 35.9:24.2:38.7:1.2.

4.1.3 Protocol for making LNPs via manual mixing: for the initial HEK cell screening experiments shown in the heat maps in Figures 2C-D, LNPs were prepared by mixing 1 µL of mRNA (1 mg/mL) with 5 µL of 10 mM citrate buffer (pH 4.0) by pipetting up and down. Subsequently, 2 µL of the mixed lipid formulation solution described above was added, and the mixture was briefly vortexed. The mixed solution was incubated at room temperature for 15 minutes. The final concentration of mRNA was 0.125 mg/mL. LNPs were stored on ice for 30 minutes to 2 hours before administration.

4.1.4 LNP microfluidic mixing: for all the following cell and animal studies, LNPs were prepared using the NanoAssemblr™ Ignite (DE, USA) for formulation volumes ⩾800 µL and a

PreciGenome microfluidic flow device (San Jose, CA, USA) for volumes <800 µL. mRNA was diluted in 10 mM citrate buffer (pH 4.0) to generate the aqueous phase. The aqueous phase was injected at a flow rate of 3 mL/min and the organic phase was injected at a flow rate of 1 mL/min. Unless otherwise specified, the resulting LNPs were dialyzed against 1000-fold excess of PBS 7.4 at 4 °C for 2-4 h, using PURD35050-1KT dialysis units for volumes <200 µL or PURN60100-1KT dialysis units for volumes 200–1000 µL (Sigma-Aldrich). After dialysis, the LNPs were stored at 4 °C until use. The LNP volume was recorded before and after dialysis to calculate the concentration. After dialysis, aliquots of the formulations were collected and adjusted to the desired final volume with PBS 7.4. The specific dilution factors are indicated in the corresponding experimental sections.

Table S1. LNP formulation composition and N/P ratio

Formulation	Lipid compositions	Weight ratio total lipids to mRNA	N/P ratio
SNP	S-lipid:DOPE:cholesterol:DMG-PEG2K = 35.9:24.2:38.7:1.2	21:1 ~ 24:1	4.1
Dlin	Dlin:DSPC:cholesterol:DMG-PEG2K = 50:10:38.5:1.5	20:1	5.9
SM-102	SM-102:DSPC:cholesterol:DMG-PEG2K = 50:10:38.5:1.5	20:1	5.6
ALC-0315	ALC-0315:DSPC:cholesterol:DMG-PEG2K = 46.3:9.4:42.7:1.6	20:1	5.0
4A3-SC8	4A3:DOPE:cholesterol:DMG-PEG2K = 50:10:38.5:1.5[5]	20:1	10.4
cKK-E12	cKK-E12:DOPE:cholesterol:DMG-PEG2K = 35.9:24.2:38.7:1.2	20:1	6.9
C12-200	C12-200:DOPE:cholesterol:DMG-PEG2K = 35.9:24.2:38.7:1.2	20:1	16.1
Dlin eq	Dlin:DOPE:cholesterol:DMG-PEG2K = 35.9:24.2:38.7:1.2	20:1	4.1

4.2 Delivery of firefly luciferase mRNA to HEK 293T cells with LNPs

HEK 293T cells were seeded in 96-well plates at a density of 10,000 cells per well in 100 µL of Dulbecco's Modified Eagle Medium (DMEM) with 10% FBS and incubated at 37 °C overnight. LNPs were formulated with firefly luciferase mRNA (GenScript, NJ, USA) as described in Section 4.1.3. LNP/mRNA complexes were diluted with PBS to a final mRNA

concentration of 5 μg/mL. Next, 10 μL of the diluted LNP mixture and 90 μL of fresh Opti-MEM were added to each well, replacing the old medium. The final dose of mRNA was 50 ng per well (n = 3 biologically independent replicates). After 24 hours, the cells were washed with PBS (pH 7.4) and analyzed using a luciferase assay kit (Goldbio, MO, USA) according to the manufacturer's instructions. Cells were washed with 200 μL PBS (pH 7.4) and lysed with 40 μL lysis buffer for 15 min. A 10 μL aliquot of the lysate was transferred to a black flat-bottom plate and mixed with 100 μL luciferase working solution (D-luciferin, 10 mg/mL, diluted 1:50 in luciferase assay buffer). Luminescence was measured immediately using a Tecan Infinite M200 plate reader (Tecan, Männedorf, Switzerland). Luciferase expression levels were normalized relative to total cell number, which was quantified by analyzing 10 μL of cell lysate using a BCA assay kit (Thermo Fisher, MA, USA). The relative cell number was calculated by comparing the BCA values in the treatment group with those in the control group.

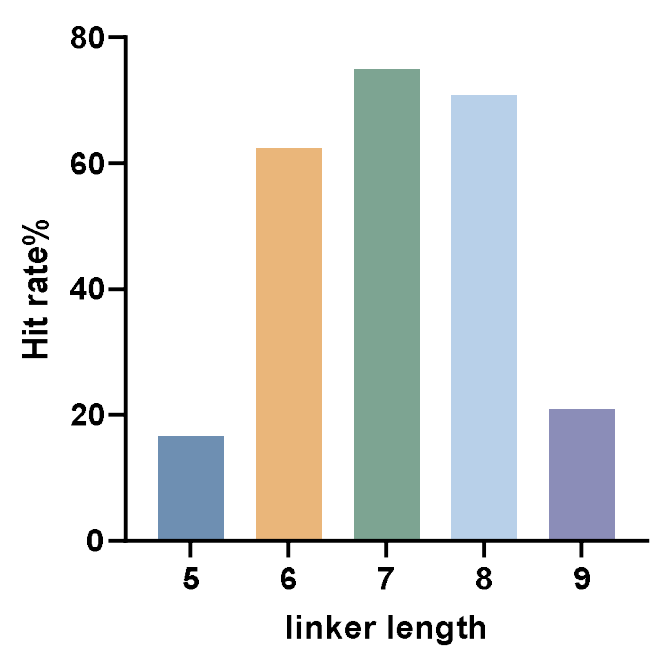

Figure S66. Effect of head–linker length S-lipid transfection efficiency (Figure 2D heat map).

The bar graph shows the percentage of epoxide-based S-lipid SNP formulations that transfected cells with a higher transfection efficiency than MC3-DLin LNPs. The hit rate is defined as the fraction of S-lipids whose transfection efficiency in HEK cells is better than that of MC3-DLin. Epoxide-based S-lipids are grouped by head-linker length (5-9 carbons). Lipids with 6, 7, and 8 carbon head-linkers exhibited the highest hit rates (greater than or equal to 60%), while S-lipids with 5 and 9 carbon head-linker lengths showed reduced efficacy.

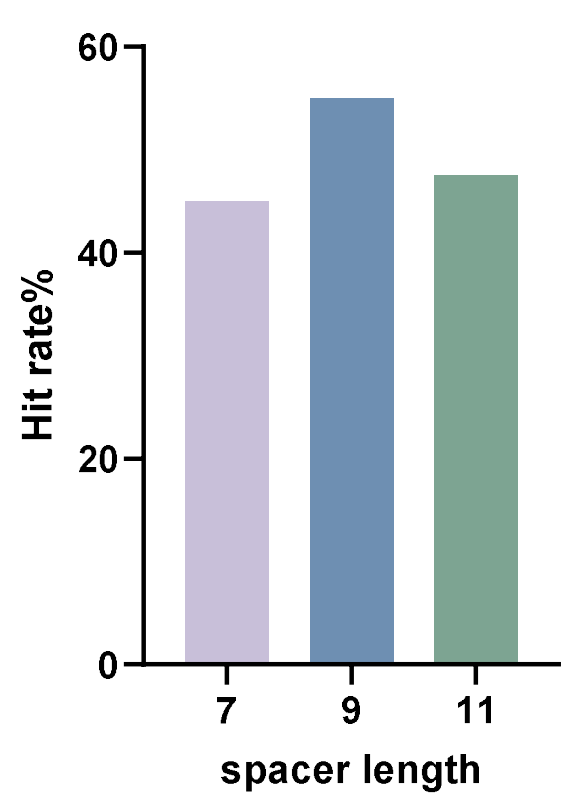

Figure S67. Effect of spacer length on S-lipid transfection efficiency (Figure 2D heat map).

The bar graph shows the percentage of epoxide-based S-lipid SNP formulations that transfected cells with a higher transfection efficiency than MC3-DLin LNPs. Hit rate is defined as the fraction of S-lipids whose transfection efficiency in HEK cells is better than that of MC3-DLin. Epoxide-based S-lipids are grouped by spacer length (7, 9, or 11 carbons). Hit rates were similar across spacer lengths, indicating that variations between 7 to 11 carbons in the spacer are well tolerated by the S-lipids.

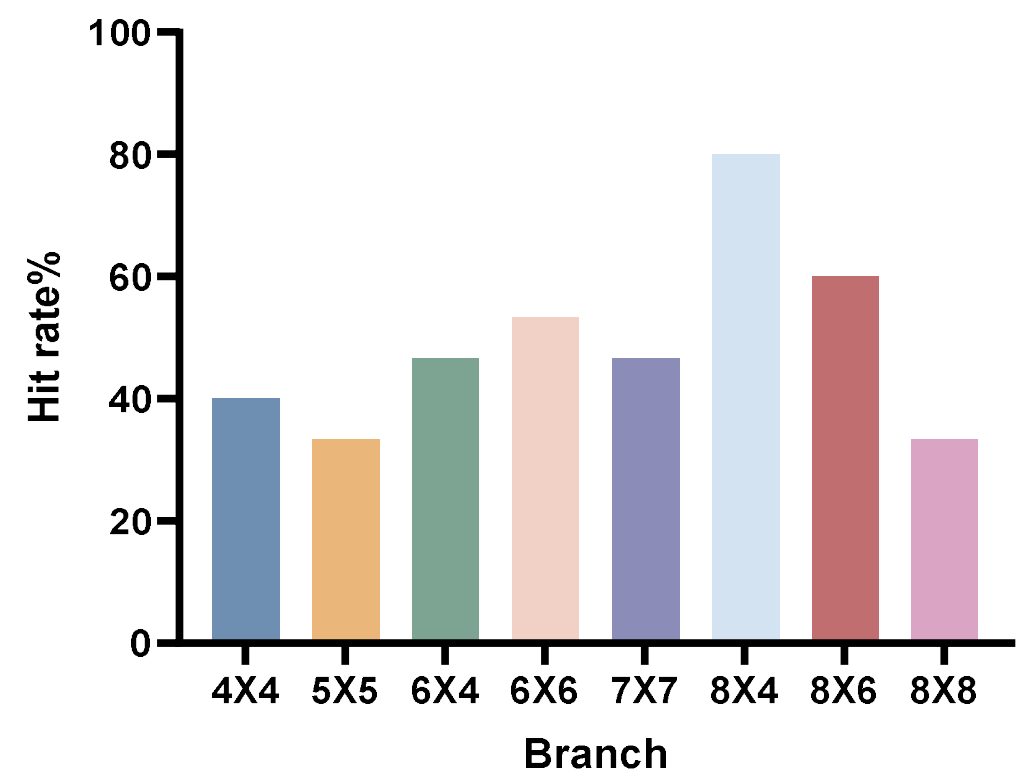

Figure S68. Effect of branch length on S-lipid transfection efficiency (Figure 2D heat map).

The bar graph shows the percentage of epoxide-based S-lipid SNP formulations that transfected cells with a higher transfection efficiency than MC3-DLin LNPs. Hit rate is defined as the fraction of S-lipids whose transfection efficiency in HEK cells is better than that of MC3-DLin. Epoxide-based S-lipids are grouped by branch length. S-lipids with asymmetric branches such as 8x4 and 8x6 showed the highest hit rates, suggesting that asymmetric branching enhances the S-lipid's ability to deliver mRNA.

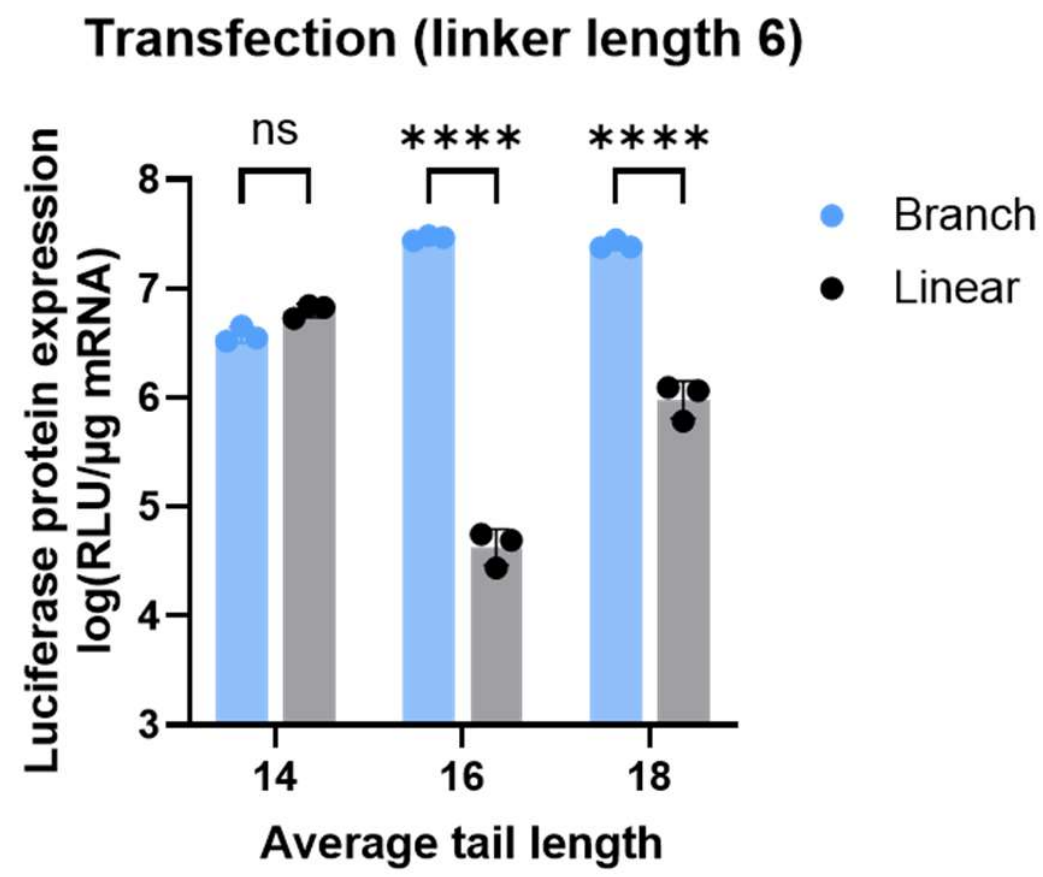

	Average tail length 14	Average tail length 16	Average tail length 18
Liner tails	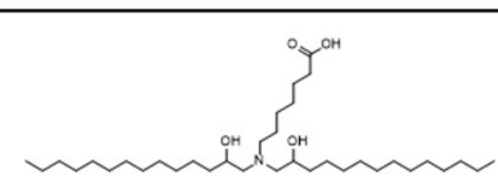	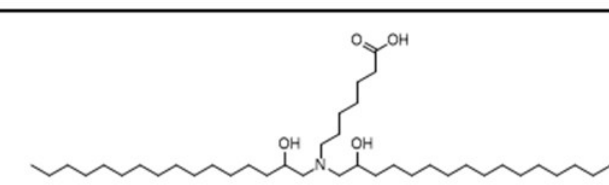	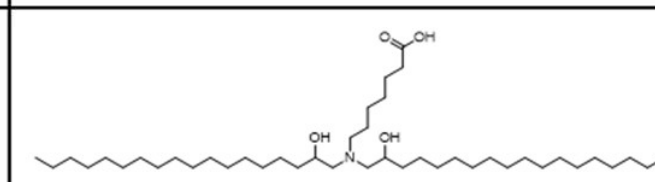
Branched tails	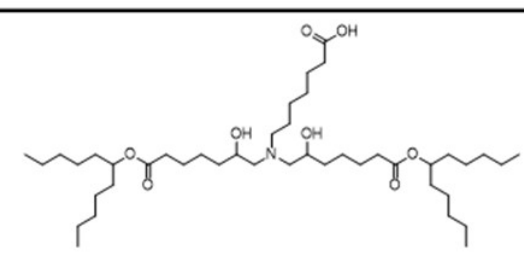	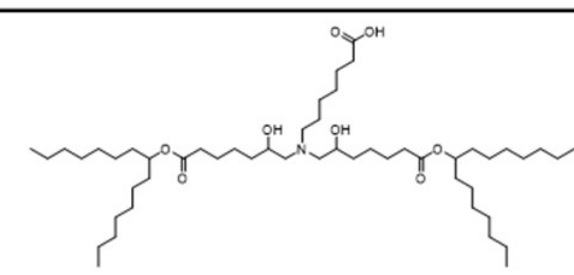	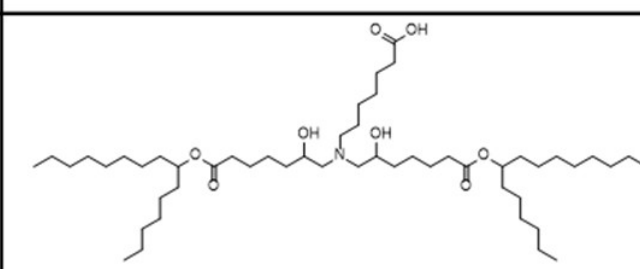

Figure S69. Epoxide based S-lipids with branched hydrophobic tails deliver mRNA more efficiently than S-lipids with linear tails.

SNPs formulated with a head-linker length of 6 with either branched or linear lipid tails were used to deliver luciferase mRNA (50 ng per well) to cells for 24 h (n = 3 biologically independent replicates). Luciferase expression (RLU/µg mRNA) was subsequently quantified. Average tail length is defined as the mean of both carbon chain lengths in the branch. For SNPs with the same average tail length, branched lipids yielded up to a 650-fold increase in luciferase expression compared to their unbranched counterparts, indicating that branched tails markedly enhanced mRNA delivery efficiency. Statistical differences were determined using two-way ANOVA followed by Šidák's test. *P < 0.05, **P < 0.01, ****P < 0.0001.

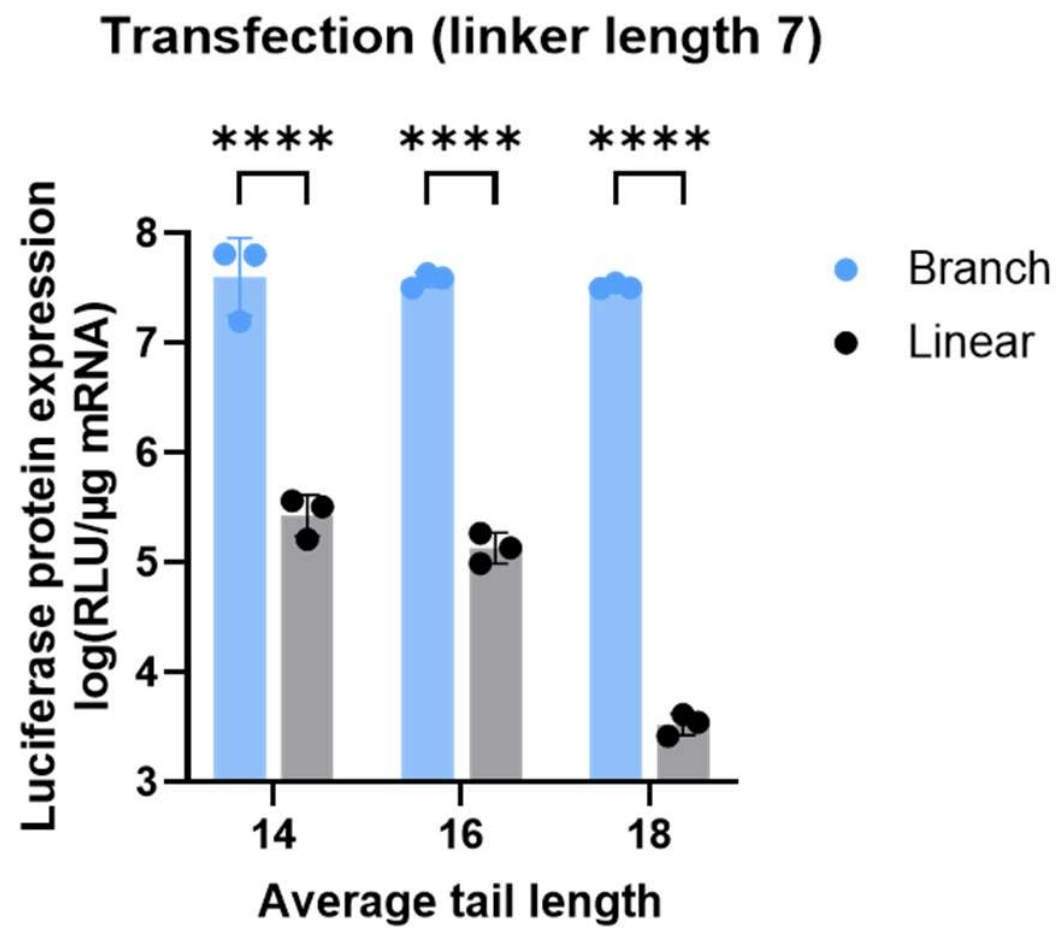

	Average tail length 14	Average tail length 16	Average tail length 18
Linear tails	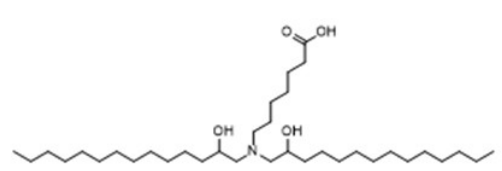	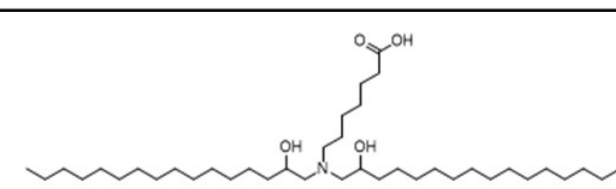	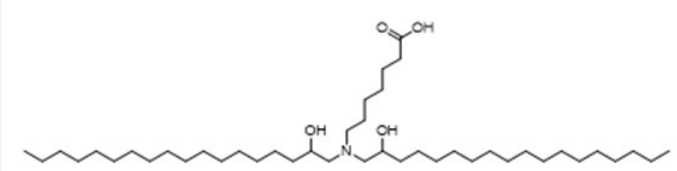
Branched tails	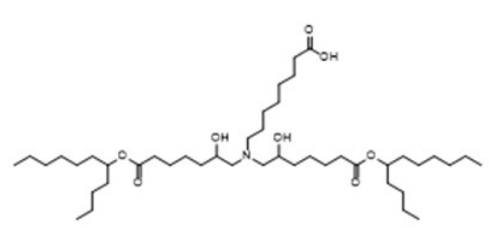	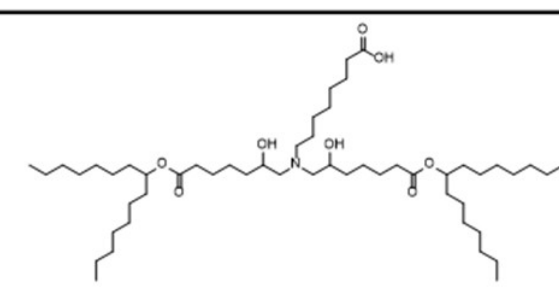	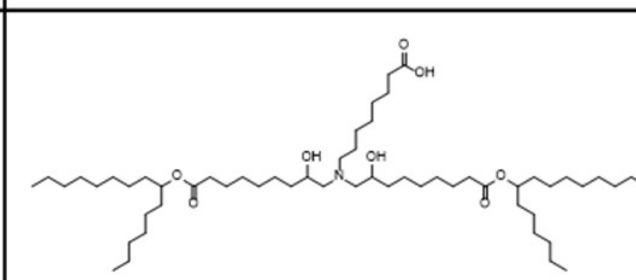

Figure S70. Epoxide based S-lipids with branched hydrophobic tails deliver mRNA more efficiently than S-lipids with linear tails.

SNPs formulated with a head-linker length of 7 with either branched or linear lipid tails, were used to deliver luciferase mRNA (50 ng per well) to cells for 24 h (n = 3 biologically independent replicates). Luciferase expression (RLU/µg mRNA) was subsequently quantified. Average tail length is defined as the mean of both carbon chain lengths in the branch. For SNPs with the same average tail length, branched lipids yielded up to a 9500-fold increase in luciferase expression compared to their linear counterparts, indicating that branched tails markedly enhanced mRNA delivery efficiency. Statistical differences were determined using two-way ANOVA followed by Šidák's test. *P < 0.05, **P < 0.01, ****P < 0.0001.

4.3 Delivery of EGFP mRNA to HEK 293T cells with LNPs

HEK 293T cells were seeded in 96-well plates at a density of 1 x 10^4 cells in 100 µL per well and incubated at 37 °C overnight in DMEM with 10% FBS. LNP/mRNA complexes were formulated as described in Section 4.1.4. Formulations containing 10 µg of GFP mRNA (GenScript, NJ, USA) were collected from the dialysis kit and diluted with PBS to a final volume of 200 µL. Aliquots of 2 µL of this dilution were mixed with Opti-MEM to a final volume of 100 µL. The resulting formulations were then added to each well without removing the existing culture medium (final mRNA concentration of 500 ng/mL). After 17 hours, the cells were washed with PBS at pH 7.4 and detached with Accutase® solution for 10 minutes at 37 °C. The transfection efficiency of the LNPs was analyzed using an Attune flow cytometer (Thermo

Fisher, MA, USA). Data analysis was performed using FlowJo v10.7.1 software (FlowJo, LLC, Ashland, OR).

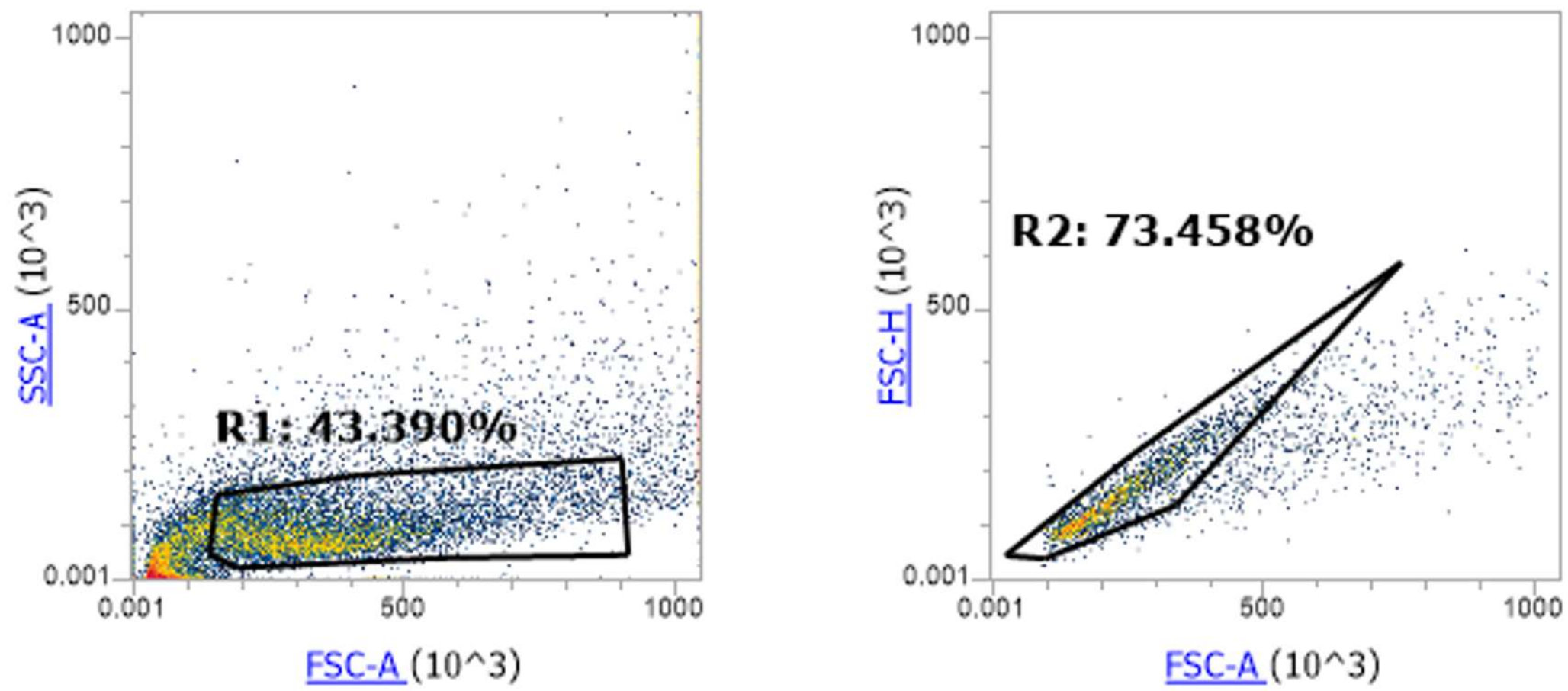

Figure S71. Flow cytometry gating strategy for analysis of EGFP mRNA transfection in HEK cells.

4.4 Delivery of EGFP mRNA to primary fibroblast cells with LNPs

Isolation of primary mouse fibroblasts: Primary dermal fibroblasts were isolated from Ai9 (*Gt(ROSA)*^{26Sor}) reporter mice following euthanasia. To ensure sterility, hair was removed using a topical depilatory agent, and the carcass was submerged sequentially in povidone-iodine and 70% ethanol for 10 minutes each. Ear pinnae tissue was harvested, washed serially through six wells of 70% ethanol and six wells of sterile PBS, and finely minced into fragments (<1 mm^3). The tissue was digested in 1.5 mL tubes containing 500 μL of 1 mg/mL collagenase in PBS at 37°C for 45 minutes, with vortexing every 15 minutes to facilitate dissociation. Following digestion, samples were centrifuged at 300x g for 5 minutes, and the pellet was resuspended in a minimal volume of D10 medium (DMEM supplemented with 10% FBS and 1% penicillin-streptomycin). The tissue suspension was plated using a low-volume explant technique to rely on surface tension for initial adhesion; cultures were maintained at 37°C with 5% CO_2, and additional medium was added after 48–72 hours upon confirmation of fibroblast outgrowth.

Cell transfection: Formulations containing 10 μg of GFP mRNA (GenScript, NJ, USA) were collected from the dialysis kit and diluted with PBS to a final volume of 200 μL. Aliquots of 2 μL of this dilution were mixed with Opti-MEM to a final volume of 100 μL. The resulting formulations were then added to each well without removing the existing culture medium (final mRNA concentration of 500 ng/mL). After 17 hours, the cells were washed with PBS at pH

7.4 and detached with Accutase® solution for 10 minutes at 37 °C. The transfection efficiency of the LNPs was analyzed using an Attune flow cytometer (Thermo Fisher, MA, USA). Data analysis was performed using FlowJo v10.7.1 software (FlowJo, LLC, Ashland, OR).

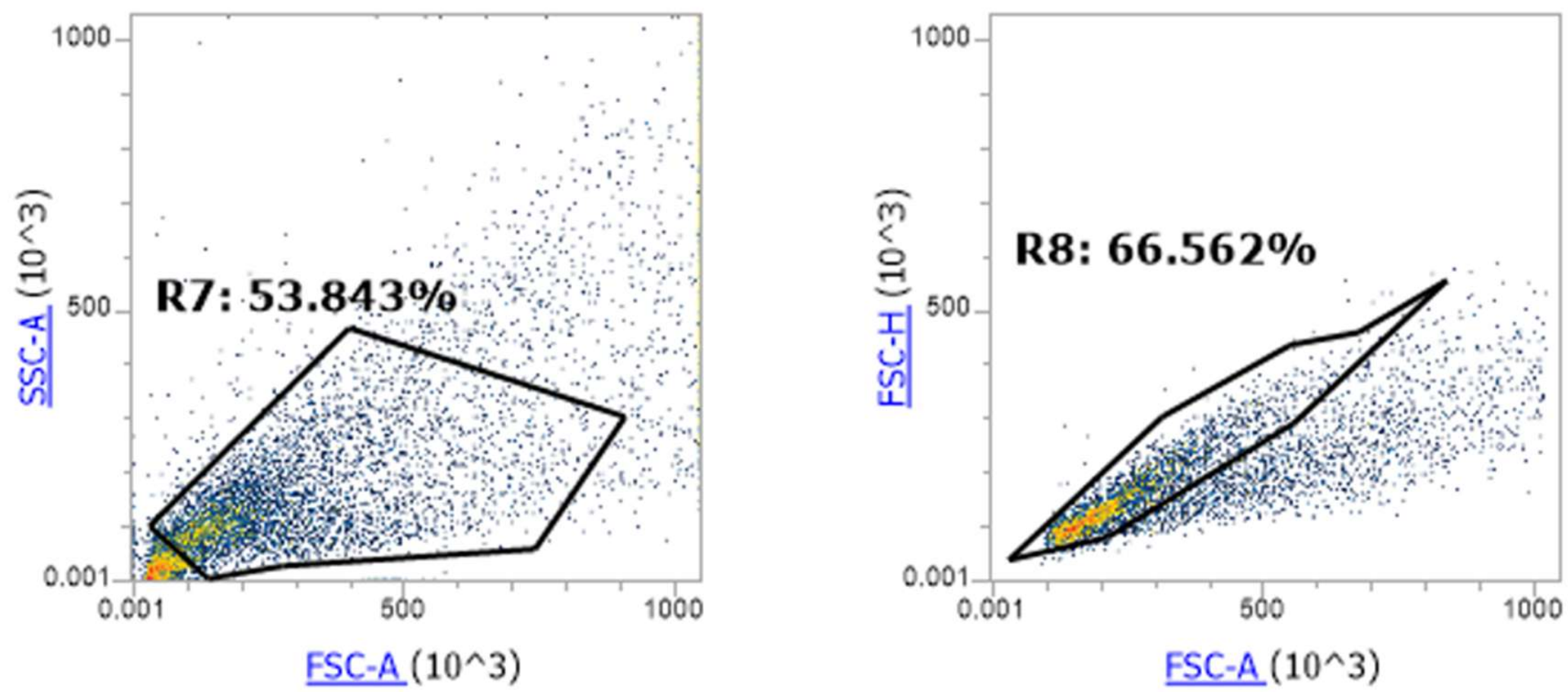

Figure S72. Flow cytometry gating strategy for analysis of EGFP mRNA transfection in primary fibroblast cells.

5. SNP Characterization

5.1 Evaluation of LNP mRNA encapsulation efficiency

The encapsulation efficiency of mRNA in LNPs was quantified using the RiboGreen assay. mRNA standards were prepared as follows. A 2 µg/mL solution of mRNA in 1X TE buffer (pH 7.4) was prepared by adding 1 µL of GFP mRNA, luciferase mRNA, or Cas9 mRNA (1.0 mg/mL, GenScript, NJ, USA) to 499 µL of 1X TE, which was then mixed thoroughly. A 100 ng/mL stock solution of RNA was prepared by diluting the 2 µg/mL RNA solution 20-fold with 1X TE. A 2000-fold diluted Quant-iT RiboGreen reagent was prepared by diluting the RiboGreen stock solution with 1X TE. LNPs were prepared as described in Section 4.1.4 without dialysis, and the SNP/mRNA complexes were diluted to 100 ng/mL mRNA with 1X TE buffer. Next, 175 µL of the 100 ng/mL LNP formulation was mixed with 350 µL of the 2000-fold diluted RiboGreen reagent and 175 µL of 1X TE in a 1.5 mL RNAse-free tube (final concentration 25 ng/mL). This mixture was incubated at room temperature for 2–5 minutes. For fluorescence measurements, 200 µL of each RNA standard (50 ng/mL, 25 ng/mL, 5 ng/mL, 1 ng/mL, and 0 ng/mL) or LNP formulation dilution was added to a black, flat-bottom 96-well plate in triplicate. The plate was shaken briefly and then incubated at room temperature for 2–5 minutes, protected from light. The fluorescence intensity was measured using a microplate

reader at excitation and emission wavelengths of 485 nm and 535 nm, respectively. Blank fluorescence values were subtracted from the samples, and the corrected fluorescence data were used to generate a standard curve of fluorescence versus RNA concentration. The concentration of unencapsulated mRNA was determined based on the standard curve, and the encapsulation efficiency of mRNA in LNPs was calculated using the formula: (1 - C/25) × 100%, where C is the concentration of unencapsulated mRNA in ng/mL.

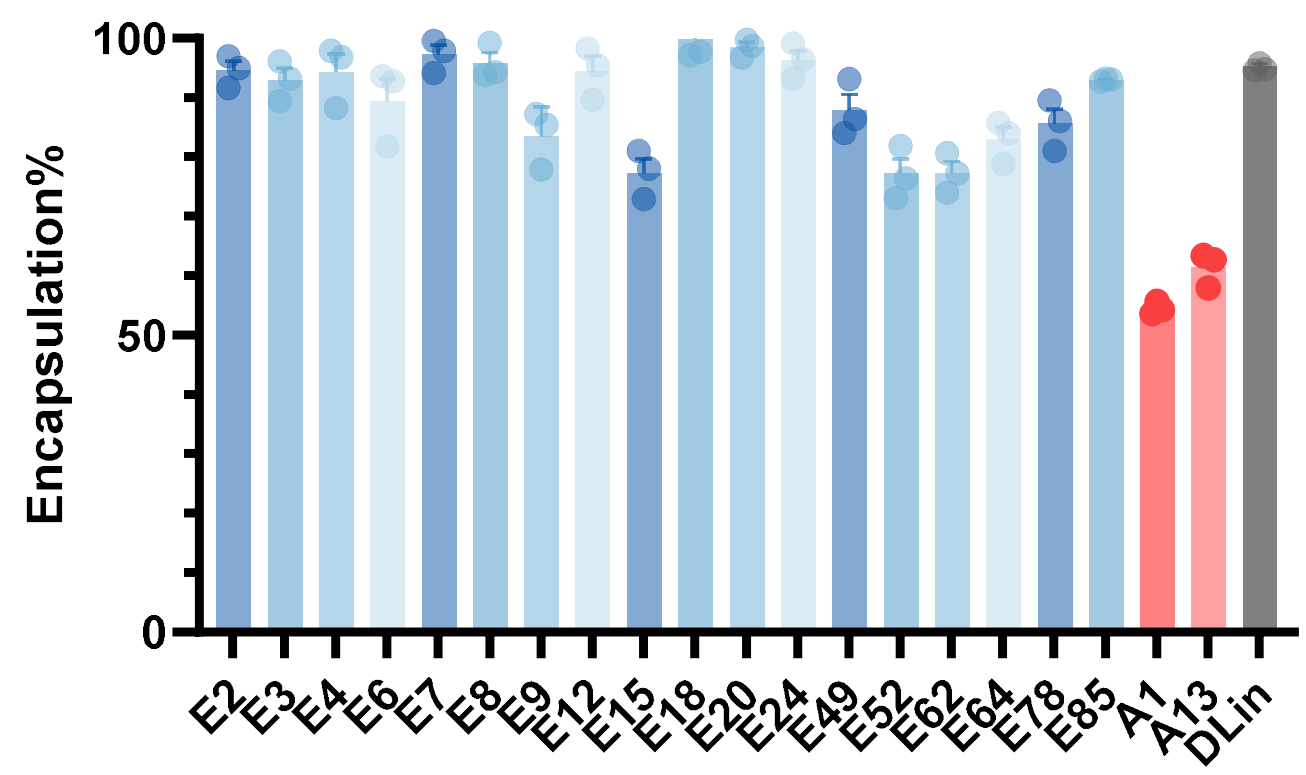

Figure S73. The GFP mRNA encapsulation efficiency of the SNPs depends on the chemical structure of the S-lipid.

The GFP mRNA encapsulation efficiency of 20 SNPs made with different S-lipids was determined (n = 3 biologically independent replicates). 10 SNPs encapsulated GFP mRNA as efficiently or better than LNPs made with traditional cationic ionizable lipids (average 95.1%).

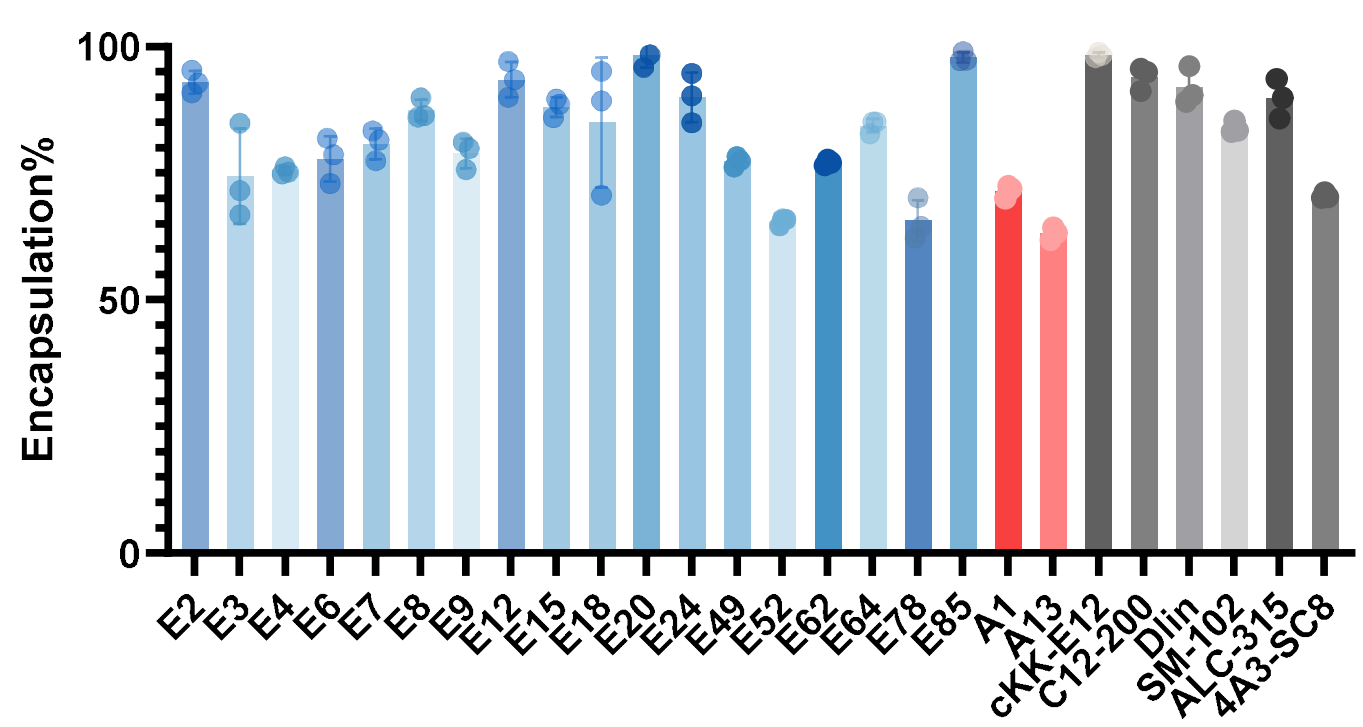

Figure S74. The luciferase mRNA encapsulation efficiency of the SNPs depends on the chemical structure of the S-lipid.

The luciferase mRNA encapsulation efficiency of 20 SNPs made with different S-lipids was determined (n = 3 biologically independent replicates). 6 SNPs encapsulated luciferase mRNA as efficiently or better than LNPs made with traditional cationic ionizable lipids (average 88.0%).

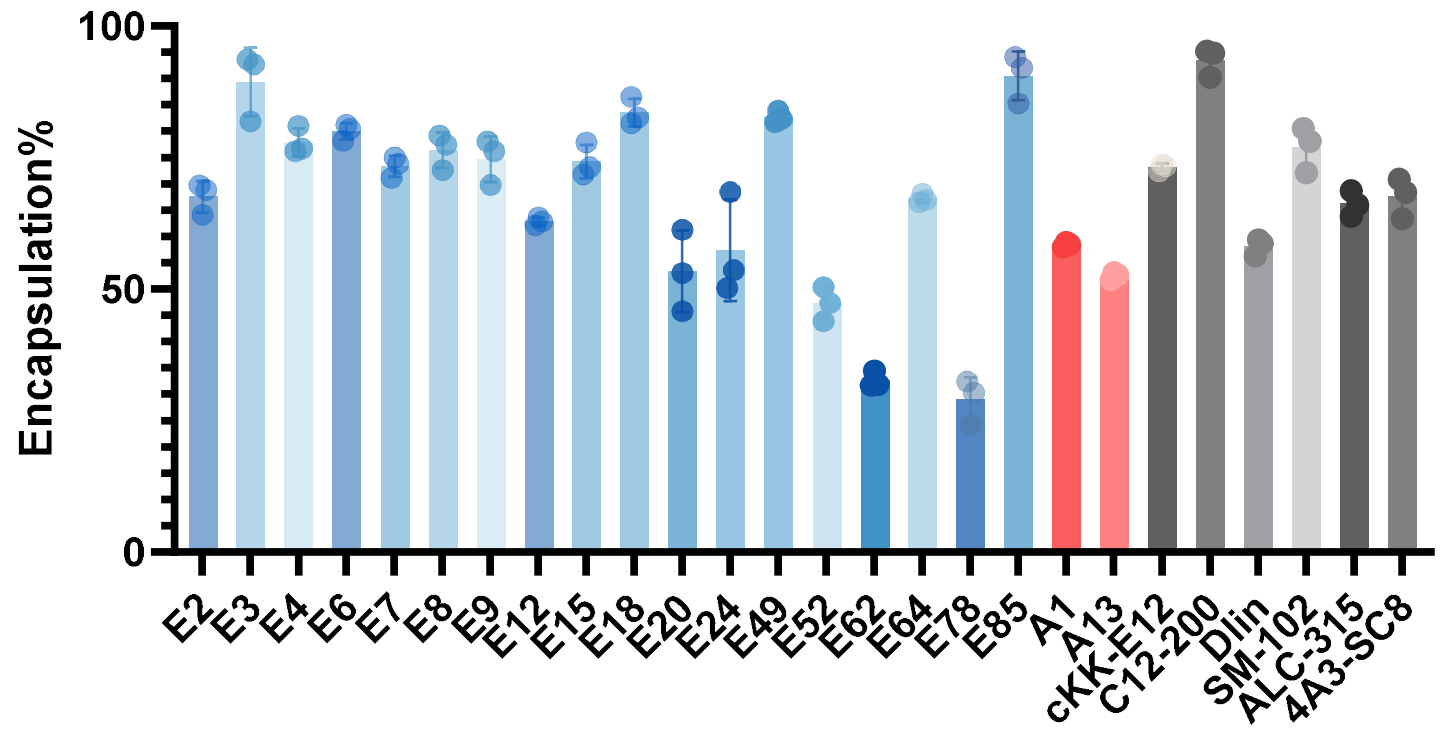

Figure S75. The Cas9 mRNA encapsulation efficiency of the SNPs depends on the chemical structure of the S-lipid.
The Cas9 mRNA encapsulation efficiency of 20 SNPs made with different S-lipids was determined (n = 3 biologically independent replicates). 6 SNPs encapsulated Cas9 mRNA as efficiently or better than LNPs made with traditional cationic ionizable lipids (average 72.6%).

We performed experiments to investigate if the S-lipid library synthesized above contained S-lipids that could encapsulate mRNA and deliver it into cells efficiently. The mRNA encapsulation efficiency of SNPs made with 20 different S-lipids and DMG-PEG, DOPE and cholesterol was investigated and compared against a panel of LNPs, using mRNAs of three different sizes, GFP mRNA (980 bases), luciferase mRNA (1914 bases) and Cas9 mRNA (4479 bases). SNPs can encapsulate mRNA of different sizes with an efficiency similar to traditional LNPs made with cationic ionizable lipids. For example, S-lipids made from epoxides (termed E-lipids) generated SNPs that encapsulated GFP, luciferase and Cas9 mRNA as efficiently as traditional cationic LNPs and had encapsulation rates similar to MC3-DLin LNPs (Figures S73-S75). The encapsulation rate of the SNPs did depend upon the chemical structure of the S-lipid, and S-lipids made from epoxides had encapsulation rates significantly higher than S-lipids made from acrylates, suggesting that the hydroxyl groups in the epoxide-based S-lipids play a key role in encapsulating mRNA[6].

5.2 Dynamic light scattering (DLS) analysis of SNPs and LNPs

DLS measurements of LNPs containing mRNA were made with a Malvern Zetasizer (Nano-ZS, Malvern Instruments, UK) instrument. LNP/mRNA complexes were formulated as described in Section 4.1.4. Formulations containing 10 μg of GFP mRNA (GenScript, NJ, USA) were collected from the dialysis kit and diluted with PBS to a final volume of 200 μL. Aliquots of 20 μL of this dilution were further diluted with 980 μL PBS to a final volume of 1000 μL (final

concentration 1000 ng/mL mRNA). The diluted sample was sterile-filtered using a 0.22 µm syringe filter and collected in a sterile conical tube. For the measurement, 100 µL of the filtered sample was transferred to a DLS cuvette (Sigma-Aldrich #759200) to determine the hydrodynamic size and polydispersity index (PDI). Results are shown in Figures S76 and S77.

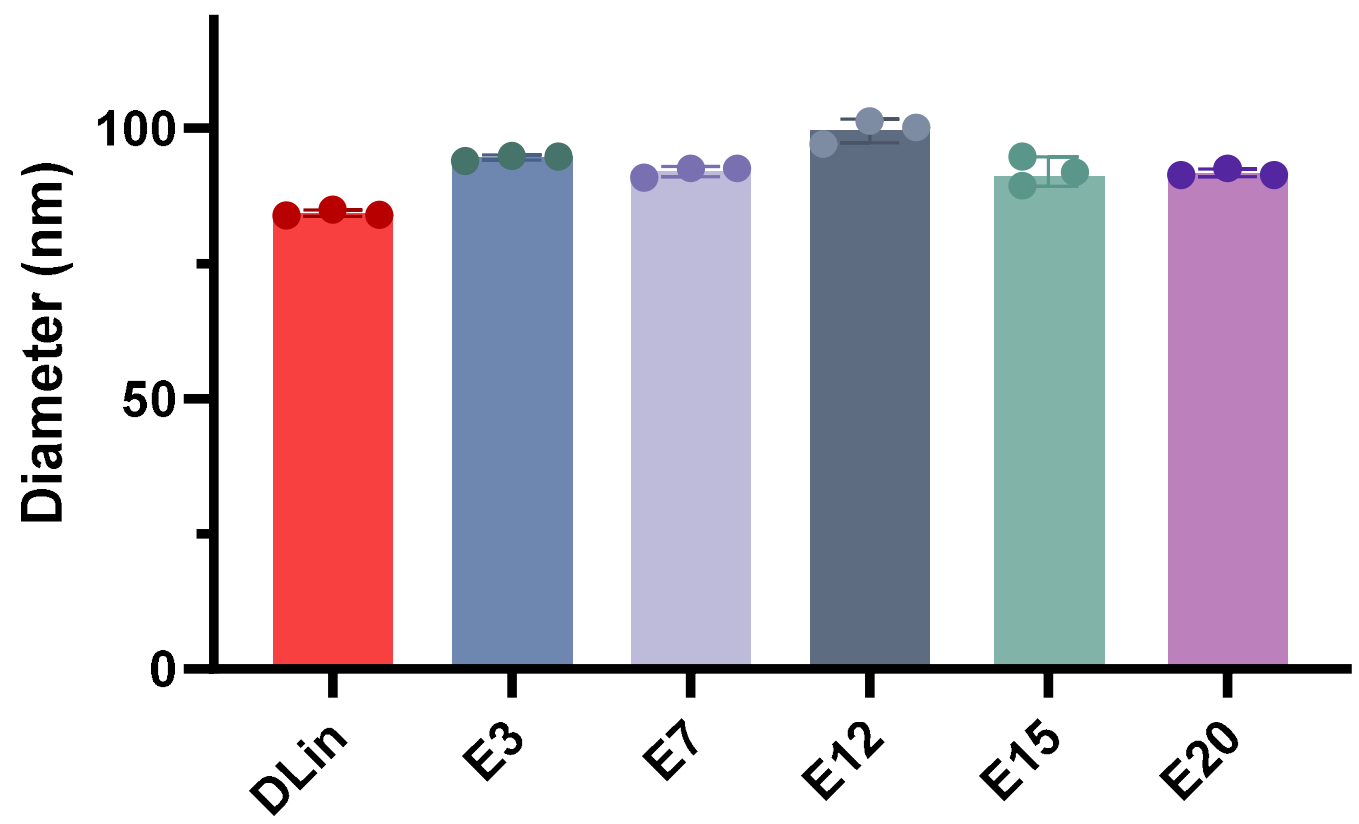

Figure S76. Hydrodynamic size of the SNPs.
Particle size was measured by dynamic light scattering (DLS) using a Malvern Zetasizer (n = 3 independent replicates). Sizes are reported as Z-average hydrodynamic diameters, corresponding to intensity-weighted values. SNPs formulated with top-performing S-lipids E3, E7, E12, E15, and E20 showed sizes ranging from 90 to 100 nm, with only slight variation across different lipid compositions.

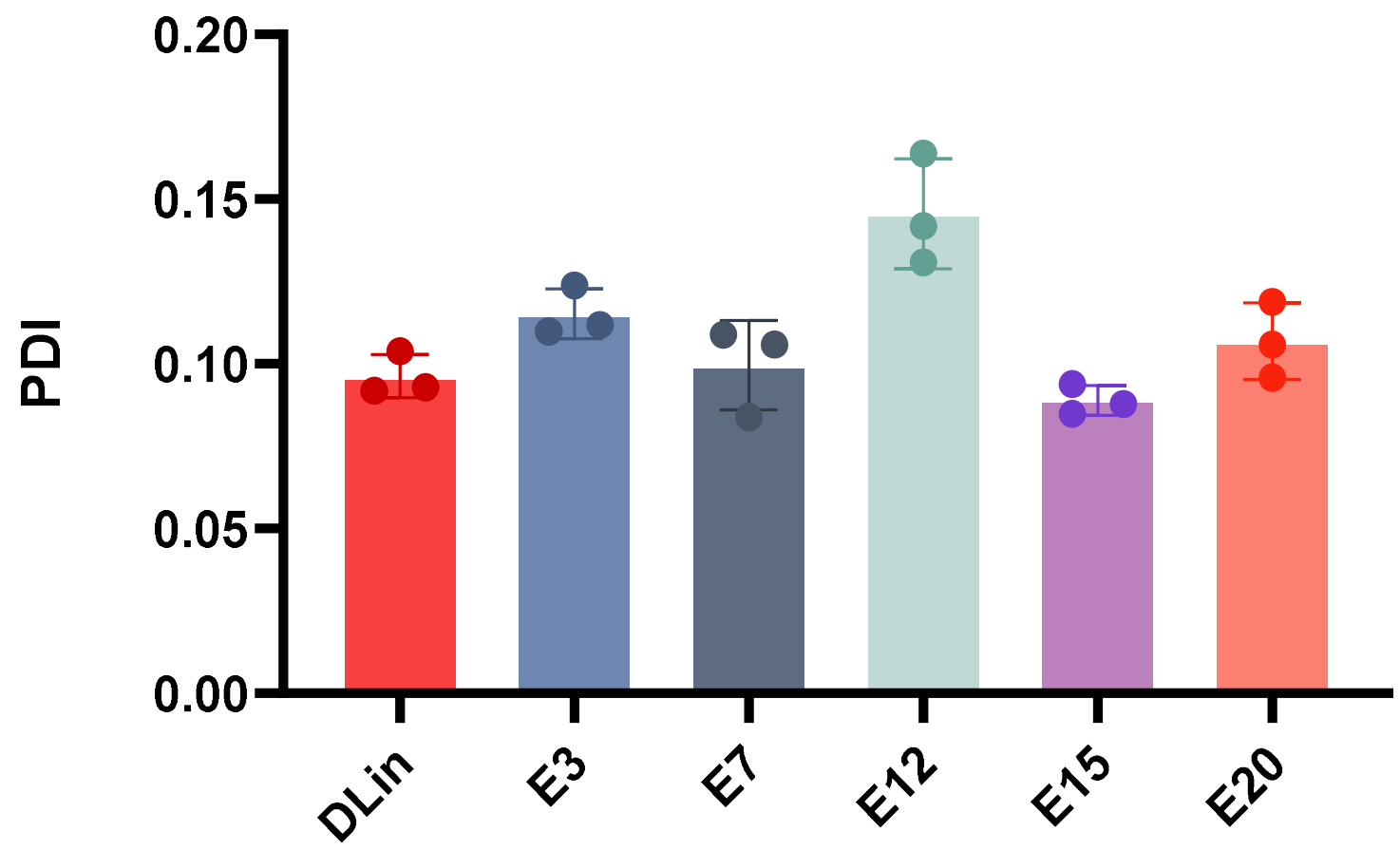

Figure S77. Hydrodynamic polydispersity index (PDI) of the SNPs.
PDI was measured by dynamic light scattering (DLS) using a Malvern Zetasizer (n = 3 independent replicates) instrument. SNP formulations exhibited low PDI values (<0.2).

5.3 Zeta potential of SNPs and LNPs

Zeta potential measurements of SNPs and LNPs were performed using a MONO ZetaView® PMX-120 instrument (Particle Metrix, Meerbusch, Germany). LNP/mRNA complexes were formulated as described in Section 4.1.4. Formulations containing 10 µg of GFP mRNA (GenScript, NJ, USA) were collected from the dialysis kit and diluted with PBS to a final volume of 200 µL. Aliquots of 20 µL of this dilution were further diluted with 980 µL to a final volume of 1000 µL (final concentration 1000 ng/mL mRNA). The sample was loaded into a folded capillary cell (DTS1070, Malvern Instruments) for measurements. Results are shown in Figure S78.

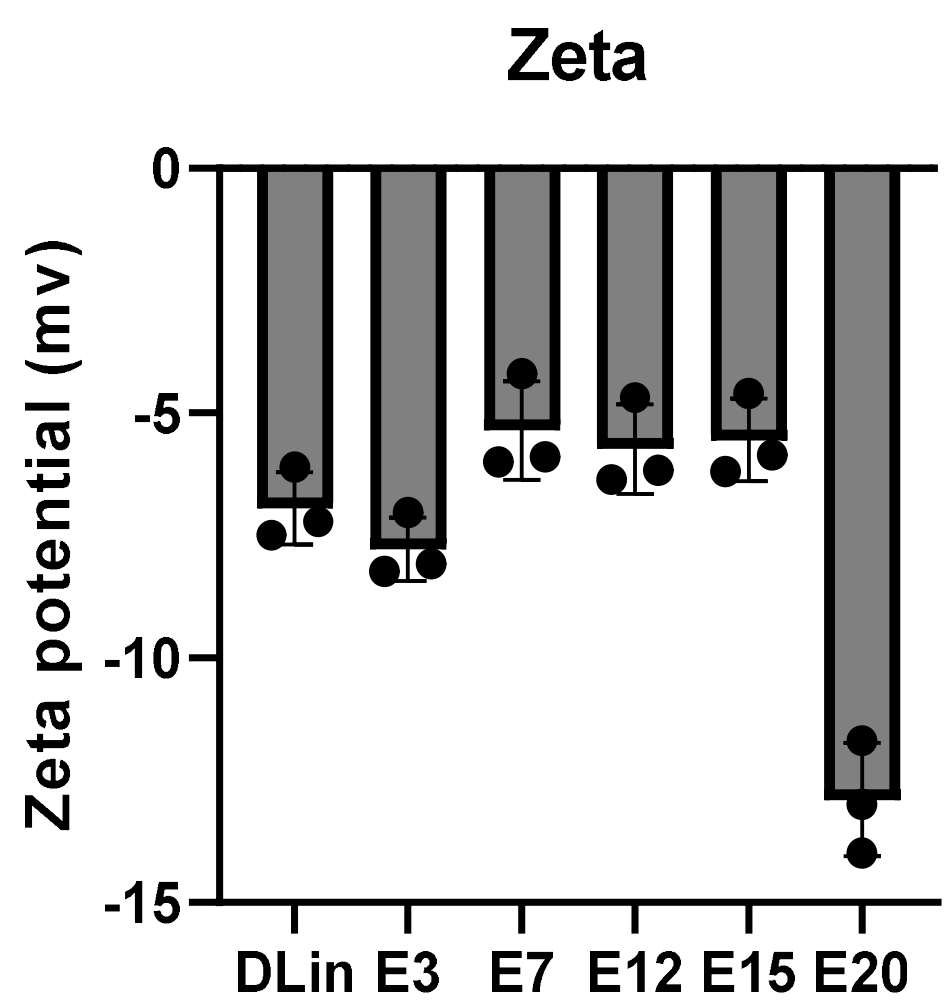

Figure S78. Zeta potential measurement of the SNPs.
The E20 SNP has a zeta potential at pH 7.4 that is significantly lower than LNPs made with the cationic ionizable lipid MC3-DLin (n = 3 independent replicates).

The zeta potential of E20 SNPs and DLin LNPs as a function of pH was determined. E20 SNPs or DLin LNPs were formulated as described in Section 4.1.4. Formulations containing 10 µg of GFP mRNA (GenScript, NJ, USA) were collected from the dialysis kit and diluted with PBS to a final volume of 200 µL. Aliquots of 20 µL of this dilution were further diluted with 980 µL of PBS buffers with pH values ranging from 3.0 to 9.0 (final concentration 1000 ng/mL mRNA). The sample was loaded into a folded capillary cell (DTS1070, Malvern Instruments) for measurements. Results are shown in Figures S79 and S80.

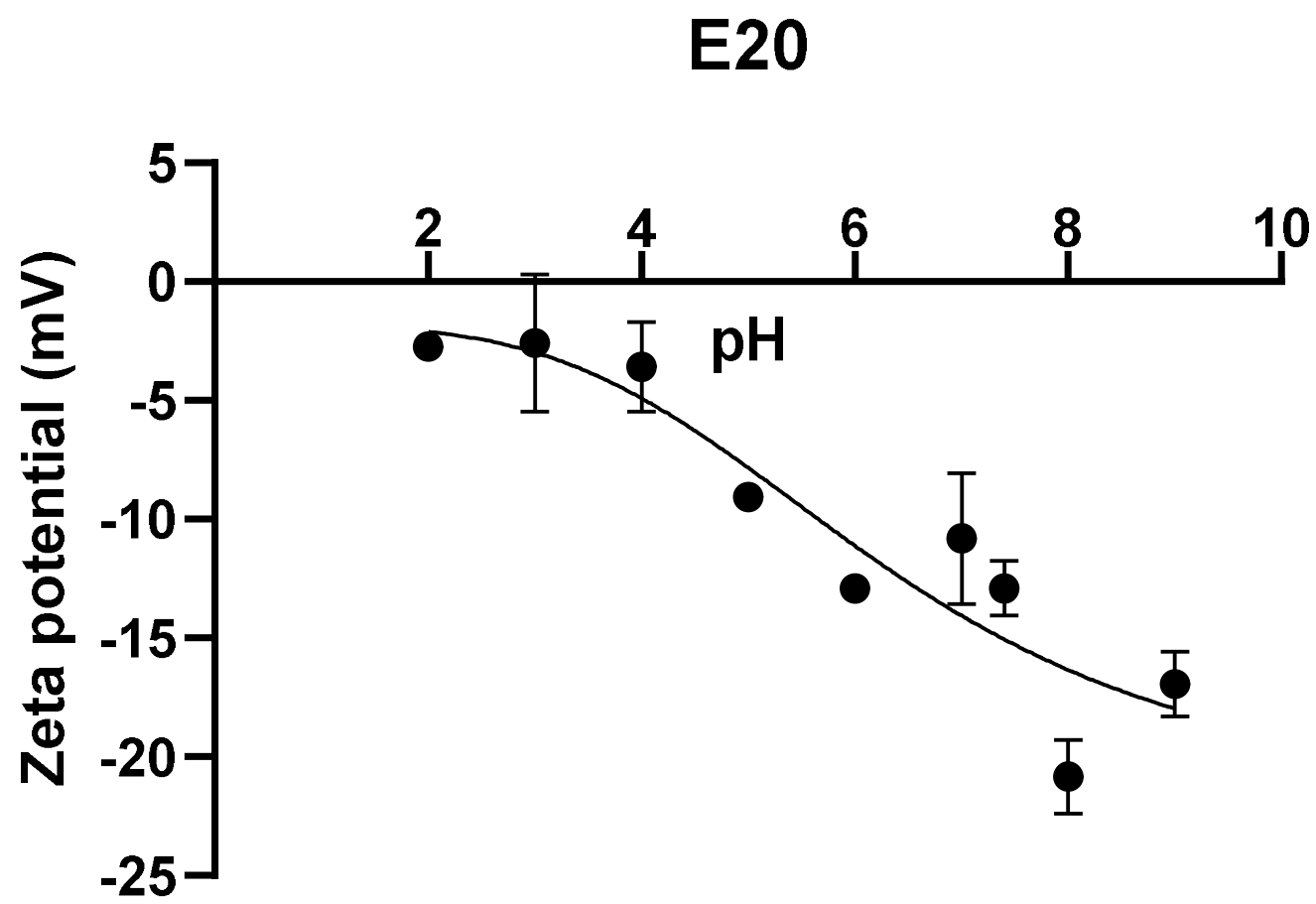

Figure S79. pH dependent zeta potential of E20 SNPs.

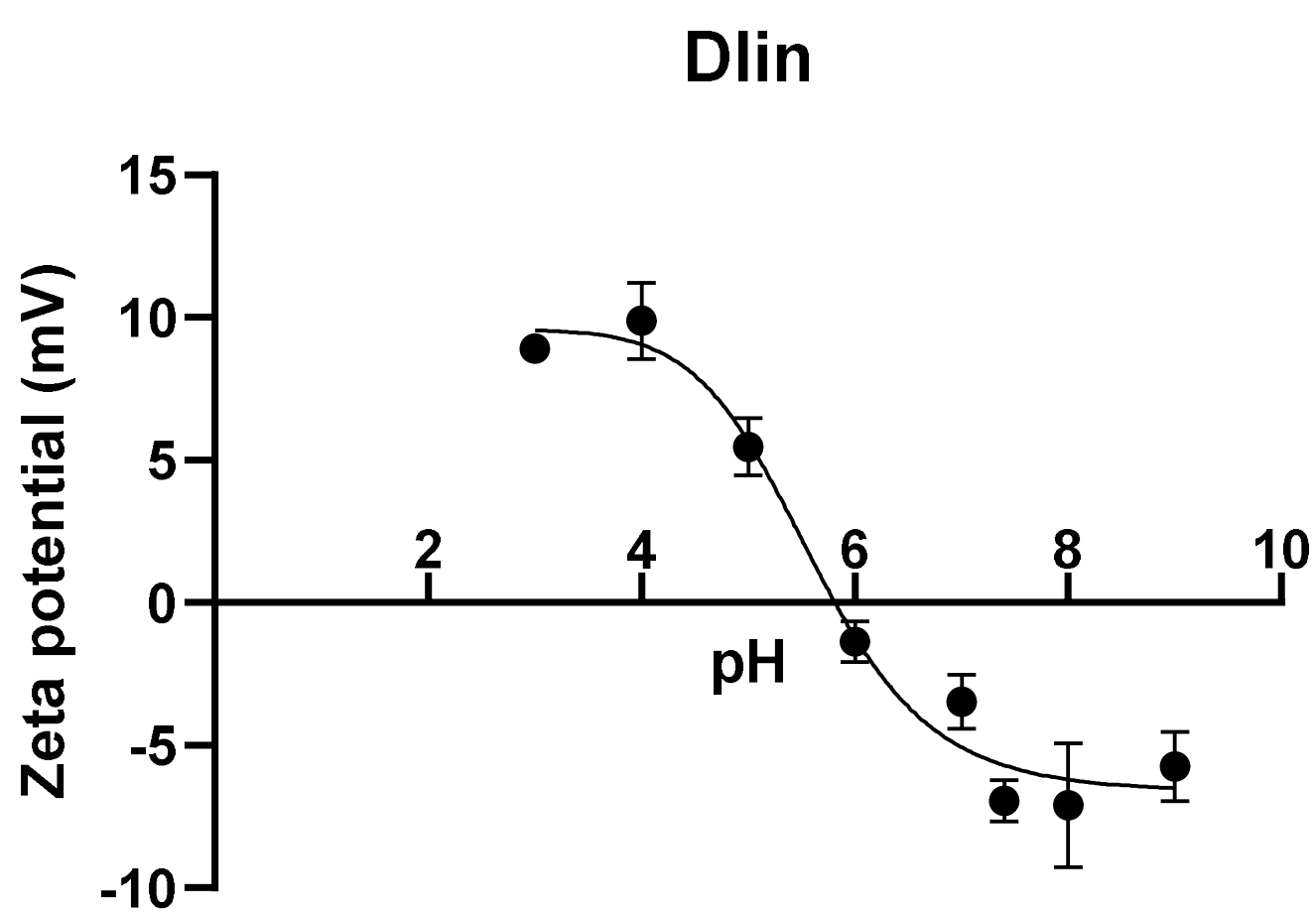

Figure S80. pH dependent zeta potential of DLin LNPs.

5.4 R18 assay for pKa measurements

The cationic fluorescent probe octadecyl rhodamine B chloride (R18) was used to evaluate the pKa at which SNPs become neutral. In this assay, the positively charged rhodamine fragment of R18 binds to negatively charged LNP surfaces, and the hydrophobic tails of R18 insert into the lipid bilayer, leading to close packing of the dye molecules and fluorescence self-quenching. As the pH decreases, the S-lipids become progressively protonated, shifting from negatively charged to zwitterionic and then to positively charged states. This charge transition alters the LNP surface charge and modulates R18 binding, enabling determination of the apparent pKa. SNP/mRNA complexes were prepared following the method in Section 4.1.4 without dialysis and diluted in PBS at pH 7.0 to achieve a final lipid concentration of 6

mM. They were then incubated for 15 minutes at room temperature and vortexed for 2 minutes. The solution was diluted to 600 μM lipid in PBS pH 7.0, and the R18 dye was added to generate a final concentration of 30 μM. This mixture was further diluted to 12.0 μM lipids in 1.5 mL of buffered solutions with pH values ranging from 2.0 to 12.0. After 30 minutes of incubation at room temperature, rhodamine fluorescence was measured at 20 °C with excitation and emission wavelengths of 568 and 594 nm, respectively. A sigmoidal best-fit analysis was used to determine the lipid pKa, defined as the pH at which 50% of the maximum fluorescence intensity was observed.

5.5 Cryo-TEM of SNPs

E20 SNP/mRNA complexes (GFP mRNA, GenScript, NJ, USA) were formulated following the method in Section 4.1.4. 5 μL aliquot of the dialyzed E20 SNP suspension (mRNA concentration was between 0.1-0.15 mg/mL) was applied to a glow-discharged R 2/2 Quantifoil Cu grid (Ted Pella, Inc.) and incubated for 20 seconds. Excess liquid was blotted for 4 seconds (blotting force = 5, 4 °C, 95–100% humidity), and the sample was plunge-frozen using an FEI Mark IV Vitrobot to form vitreous ice. Grids were stored in liquid nitrogen until they were imaged. Cryo-TEM imaging was performed on an FEI Talos Arctica operating at 200 kV under low-dose conditions. Images were acquired using a bottom-mount K3 camera (Gatan, Inc.) at 36,000× magnification (0.5705 Å/pixel) with a nominal under-focus of 1–3 μm to enhance contrast. Image processing was conducted using CryoSPARC v.4.5.3. Each sample was prepared in triplicate, with three grids per sample, and imaged at ten different fields to account for potential sample collapse.

Microscopy images were analyzed to determine the diameters of E20 SNPs. 300 E20 SNPs were analyzed. The edges of SNPs with clearly defined circular contours were manually outlined in red in the images. A custom Python script was then used to extract the red contours to obtain particle sizes. Images were converted from BGR to HSV color space, and the red-colored contours were extracted and identified. Each detected particle was fitted with an ellipse to obtain the major and minor axes. The particle diameter was approximated as the average of these two axes. Pixel dimensions were converted to nanometers using the existing scale bar of the FEI Talos Arctica microscope. The script also generated images showing the fitted ellipses for each particle, and all fittings were manually inspected to ensure that no particle merging or misfitting occurred. The extracted particle diameters were exported to a CSV file, from which the mean diameter and standard deviation were calculated.

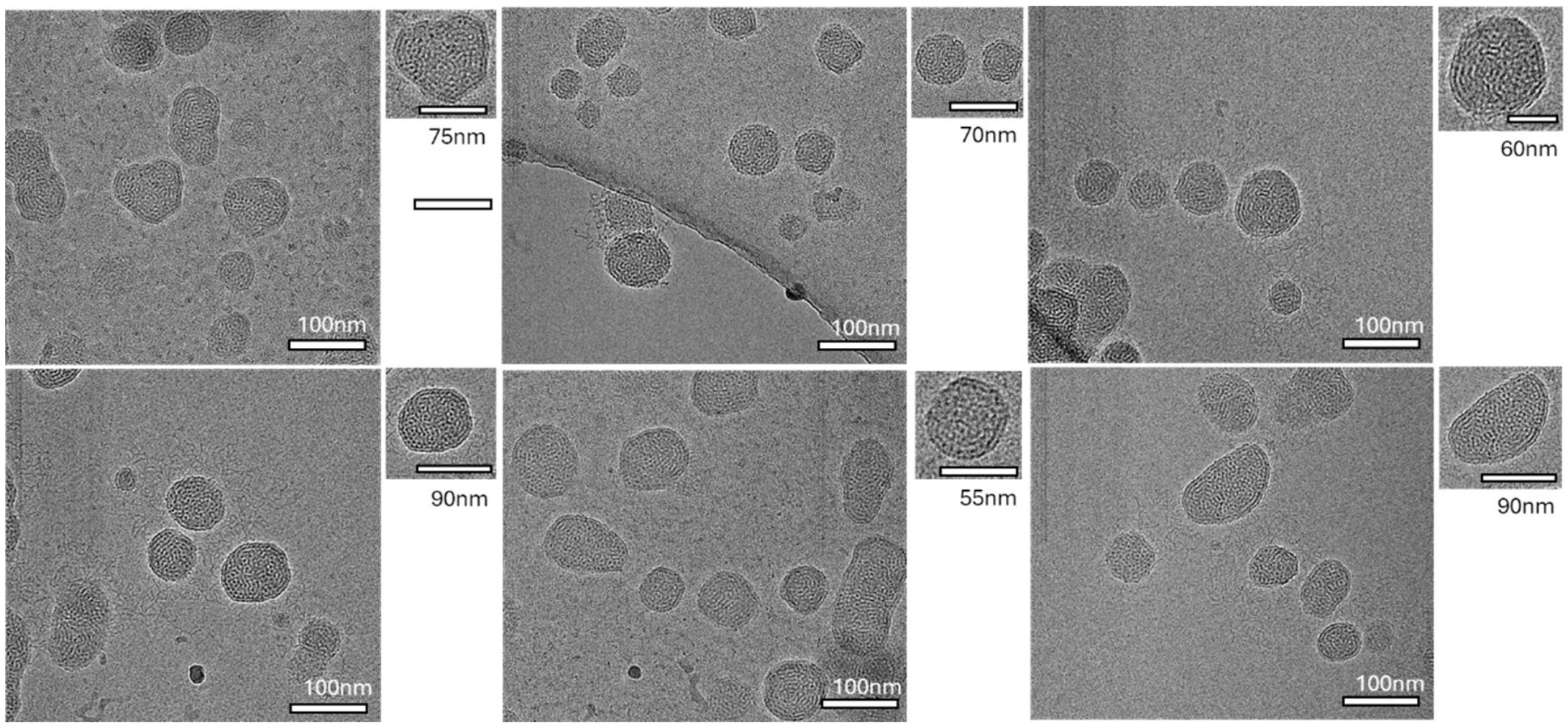

Figure S81. Representative cryo-TEM images of E20 SNPs.
Representative Cryo-TEM pictures of E20 SNPs are shown. Cryo-TEM demonstrated that E20-SNP contained a mixture of inverted hexagonal and bilayer phases in their core and had a shell that was predominantly composed of bilayer phases. The average particle diameter is 81 nm with a standard deviation of 18 nm. Images are representative of 85 fields of view collected from 3 independently prepared samples.

5.6 Small angle X-ray scattering (SAXS) of E20 SNPs

SAXS was performed to determine the mesophase structure of the E20 SNP. E20 SNPs were prepared as described in Section 4.1.4 and subsequently diluted in PBS 7.4 to a final concentration of 100 µg/mL mRNA. SAXS data was acquired on the SIBYLS beamline (12.3.1) at the Advanced Light Source, Lawrence Berkeley National Laboratory, in High-Throughput (HT-SAXS) mode using a Tecan liquid handler. After an initial assessment of the samples' resistance to radiation damage, data were acquired using 0.5 s exposures for 20 s at an X-ray wavelength of 1.234 Å, with a sample-to-detector distance of 2.094 m, yielding a q-range of 0.009–0.44 Å^{-1} ($q = \frac{4\pi}{\lambda} sin\theta$, where 2θ is the scattering angle and λ is the X-ray wavelength), using a Pilatus3 2M pixel detector. 2-D images were reduced and integrated using in-house scripts at SIBYLS. The sample dataset was then buffer subtracted and all 40 frames were averaged, given the absence of radiation damage, yielding the final 1-D SAXS profile. A background proportional to q^{-4} was subtracted to empirically account for the Form Factor, and the Structure Factor was analyzed by fitting Voigt curves corresponding to the Inverted Hexagonal (H_{II}) and Lamellar (L_α) phases, plus a Disordered peak to account for

residual contribution from the Form Factor. The centers of the higher order peaks for the two phases were constrained to be multiples of the first order peaks ($q_2 = \sqrt{3}q_1$ for the *HII* phase, and $q_2 = 2q_1$ for the L_α phase).

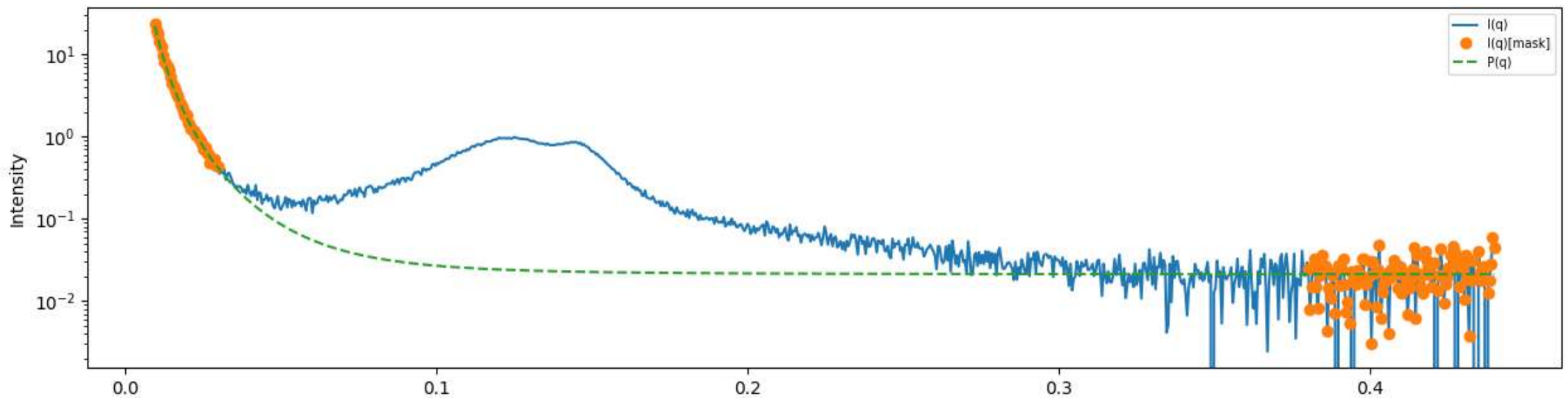

Figure S82. SAXS analysis of E20-SNPs.
SAXS analysis demonstrated that E20-SNP contained a mixture of inverted hexagonal and bilayer phases

5.7 Viscosity measurement of SNPs

To understand the relationship between spacer length and the stability of SNPs, we assessed LNP viscosity based on the fluorescent dye 9-(2,2-dicyanovinyl)juloidine (DCVJ) method[2]. DCVJ (72335-5MG, Sigma-Aldrich) is a molecular rotor whose fluorescence intensity depends on its rotational freedom, which is determined by its environment. In particular, DCVJ contains a julolidine moiety connected to a dicyano based quencher, and the quenching efficiency of the dicyano group is determined by the viscosity of the solvent. The viscosity of the SNP formulations was determined by quantifying the ratio of DCVJ fluorescence emission to absorbance in DCVJ solutions that contained SNPs. The fluorescence of the DCVJ/SNP solutions were compared to a calibration curve of DCVJ solutions of known viscosity.

Sample preparation: To prepare the 100 μM DCVJ stock, 1 μL of 50 mM DCVJ in DMSO was added to 499 μL of EtOH. Ethanol lipid solutions that contained S-lipids with different spacer lengths and the other lipids in the SNPs were prepared following the method described in Section 4.1.2. 20 μL of these formulations were added to 3 μL of the DCVJ stock solution, resulting in 0.1 mol% DCVJ in the formulation.

Samples measurement: DCVJ containing LNP solutions were added to a black 96-well plate with a transparent bottom. The plate was incubated for 5 minutes at 37 °C before measurement. Fluorescence was measured using a plate reader with an excitation of 440 nm, and emission spectra were collected at a 2 nm wavelength step from 460 to 650 nm, resulting in 96 total data points per well. To measure absorbance, 22.2 μL of SDS was added to the DCVJ-LNP solution and incubated at 60 °C for 10 minutes, followed by brief vortexing.

Absorbance was measured at wavelengths ranging from 350 to 550 nm with a 2 nm wavelength step.

Fluorescence intensity was corrected by normalizing fluorescence (F) to absorbance (A) via the ratio R = F/A. The area under the fluorescence and absorbance spectra with a wavelength step of 2 nm was integrated. Fluorescence values were integrated over the range of 470-650 nm, and absorbance values were integrated over the range of 390-510 nm. The resulting ratio was used to assess relative changes in viscosity.

Calibration curve: the membrane viscosity of each LNP was measured using a calibration curve prepared with a glycerol-methanol mixture. Glycerol-methanol mixtures were prepared at volume ratios of 0:100, 50:50, 60:40, 70:30, 80:20, 90:10, and 100:0, with a total volume of 200 µL for each mixture. To each mixture, 3 µL of 100 µM DCVJ was added, and the R value was measured at 30 °C using the methods described above. A viscosity versus R calibration curve was established

Viscosity calculation: viscosity was calculated from the experimental R values using the viscosity versus R calibration curve. Results are shown in Figures S83 and S84.

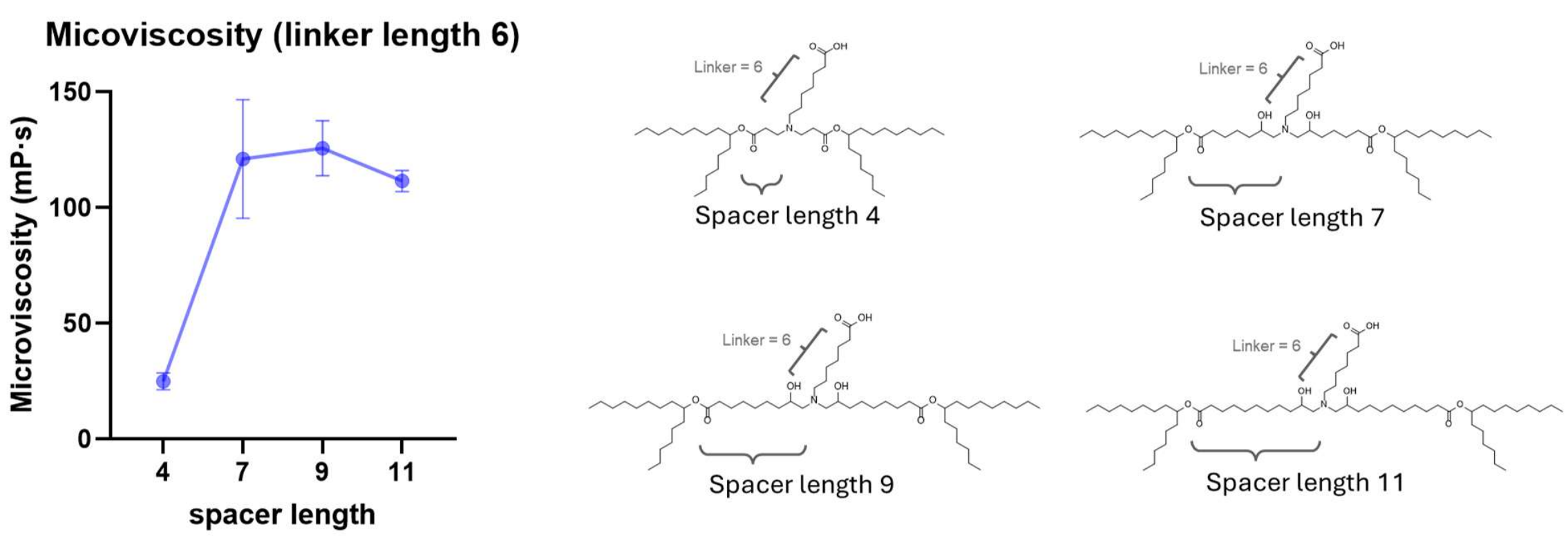

Figure S83. The S-lipid spacer length influences the microviscosity of the SNPs. The microviscosity of SNPs made with 4 different S-lipids that had a branched tail of 8X6 and a head linker length of 6 was investigated (n = 3 independent replicates). The S-lipids had spacer lengths of 4, 7, 9, and 11 carbons. The SNP microviscosity increased with spacer length up to 9 carbons and then began to decrease slightly at longer lengths.

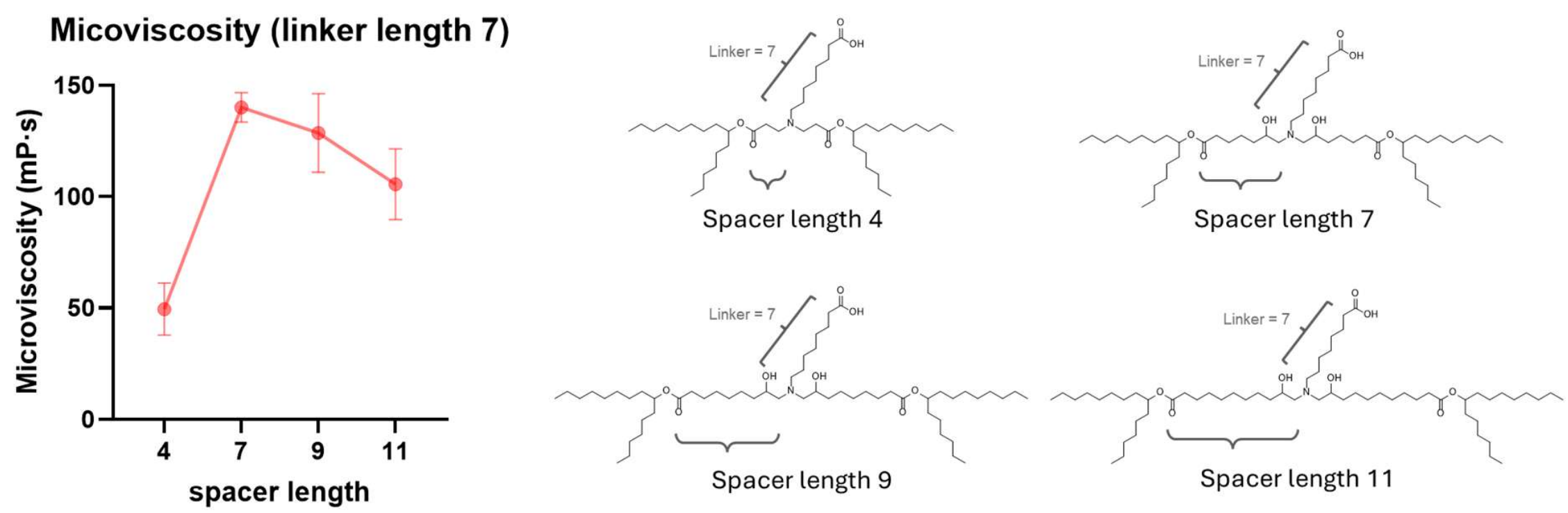

Figure S84. The S-lipid spacer length influences the microviscosity of the SNPs.
The microviscosity of SNPs made with 4 different S-lipids that had a branched tail of 8X6 and a head linker length of 7 was investigated (n = 3 independent replicates). The S-lipids had spacer lengths of 4, 7, 9, and 11 carbons. The SNP microviscosity increased with spacer length up to 7 carbons and then began to decrease slightly at longer lengths.

5.8 Shelf-life stability of SNPs at 4 °C

E20 SNPs or Dlin LNPs containing EGFP mRNA were prepared as described in Section 4.1.4. The dialyzed SNPs and LNPs were aliquoted into PCR tubes and stored at 4 °C for 7, 14, and 21 days and then analyzed for transfection activity. On the day of analysis, both LNPs and SNPs were further diluted with PBS and evaluated for transfection activity in HEK293T cells (final mRNA concentration of 500 ng/mL). The transfection efficiency of LNPs was quantified following the method detailed in Section 4.3.

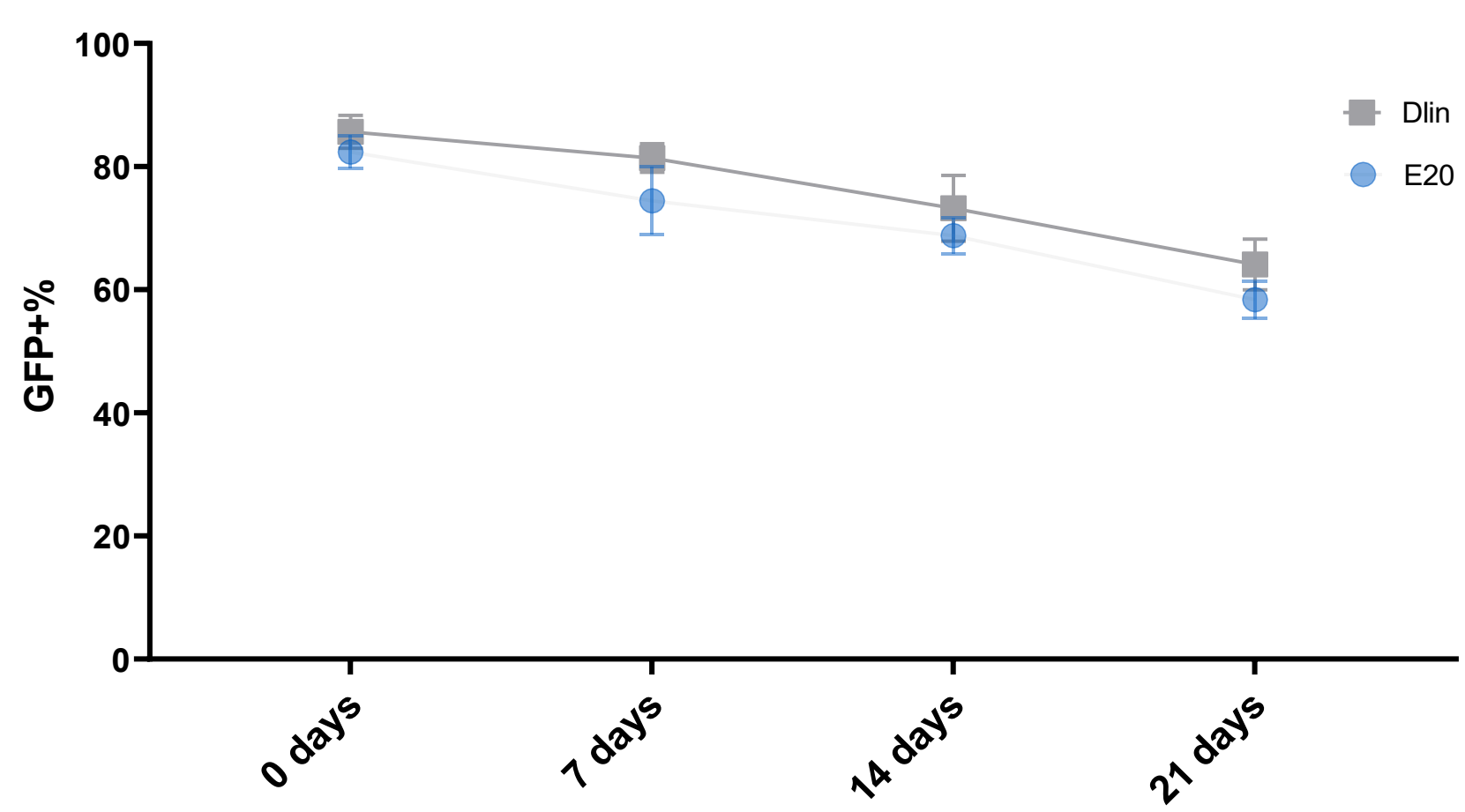

Figure S85. E20 SNPs and DLin LNPs have similar shelf-lives.
E20 SNPs transfected cells with similar efficacy to DLin LNPs after storage at 4 °C. Data are presented as mean ± SEM (n = 3 biological independent replicates).

6. *In vivo* mRNA and pDNA delivery experiments with LNPs

General mice protocol: All animal procedures were approved by the University of California Berkeley (UCB) and Davis Institutional Animal Care and Use Committees (IACUCs). All facilities used during the study period were accredited by the Association for the Assessment and Accreditation of Laboratory Animal Care International (AAALAC). C57BL/6J mice (000664) and BALB/c (000651) were purchased from The Jackson Laboratory. All mice were group-housed at the University of California, Davis and the University of California, Berkeley, with a 12-h light-dark cycle with ad libitum access to food and water at 20-22 °C with 40-60% humidity. BALB/c and C57BL/6J mice 10-16 weeks old were used for this study.

6.1 *In vivo* firefly luciferase mRNA delivery with SNP/mRNA complexes

Wild-type BALB/c mice weighing between 18–20 g, and aged 10–16 weeks old were used for firefly luciferase mRNA delivery experiments. SNP/mRNA complexes were made as described in Section 4.1.4 with luciferase mRNA (GenScript, NJ, USA). An aliquot of dialyzed SNP/mRNA complexes containing 25 µg mRNA was diluted to 500 µL using PBS (pH 7.4). A 100 µL volume of LNPs (0.25 mg/kg mRNA) was administered intravenously (IV) via retro-orbital injection. 4-6 hours later, a D-luciferin solution in PBS pH 7.4 (50 mg/kg) was injected into the intraperitoneal (IP) cavity of the mice. After 10 minutes, the luciferase luminescence intensity of the whole body and organs (lungs, liver, kidneys, heart, and spleen) was imaged using an IVIS spectrum imager (PerkinElmer, MA, USA). Results are shown in Figures S86, S87 and S88.

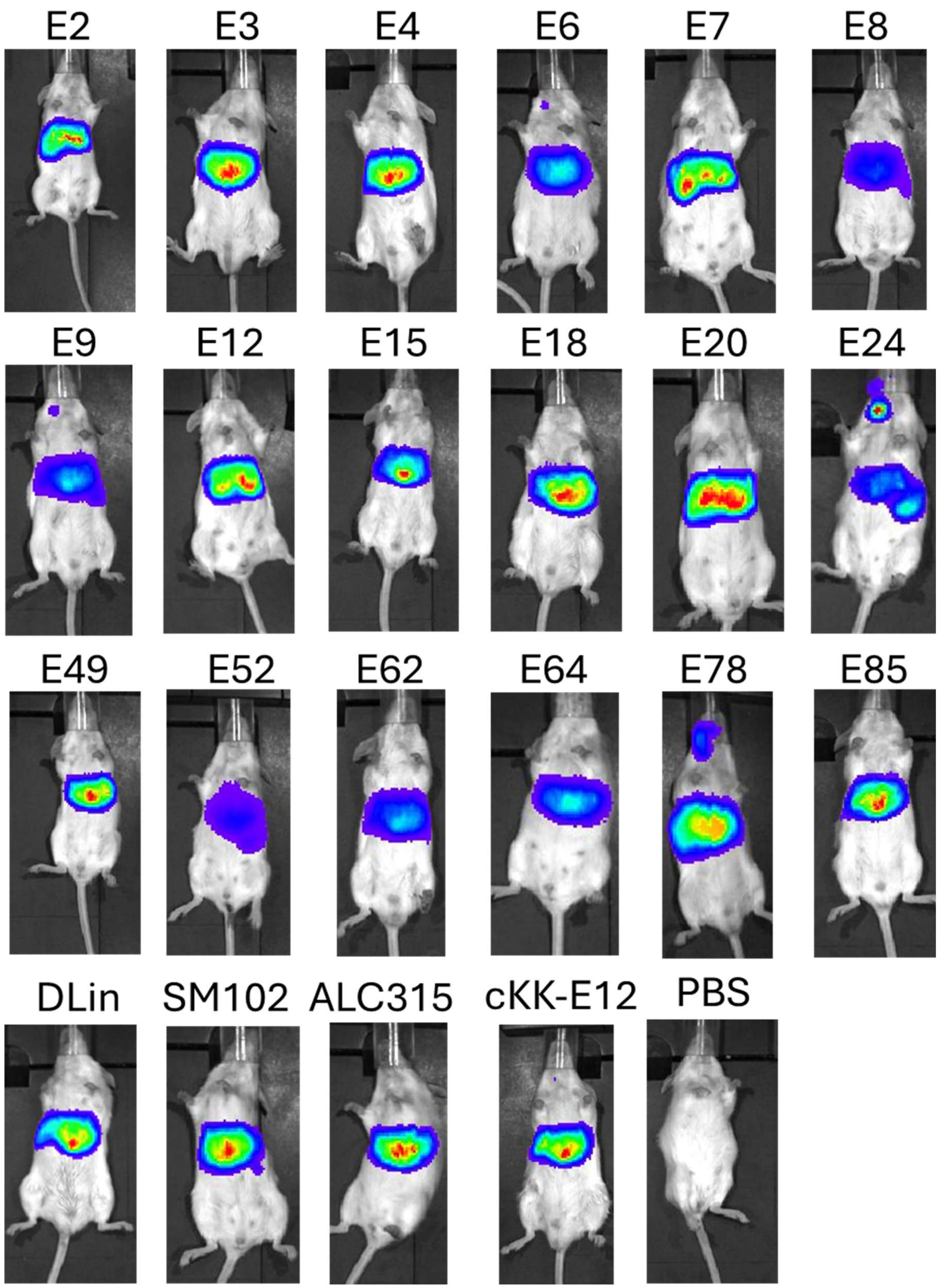

Figure S86. In vivo luminescence intensity of mice treated with SNPs. Luminescence signals reflect mRNA delivery efficiency, with red regions indicating higher luciferase expression. Mice treated with PBS served as negative controls and showed minimal signal. Several S-lipid formulations produced stronger luminescence than MC3-DLin.

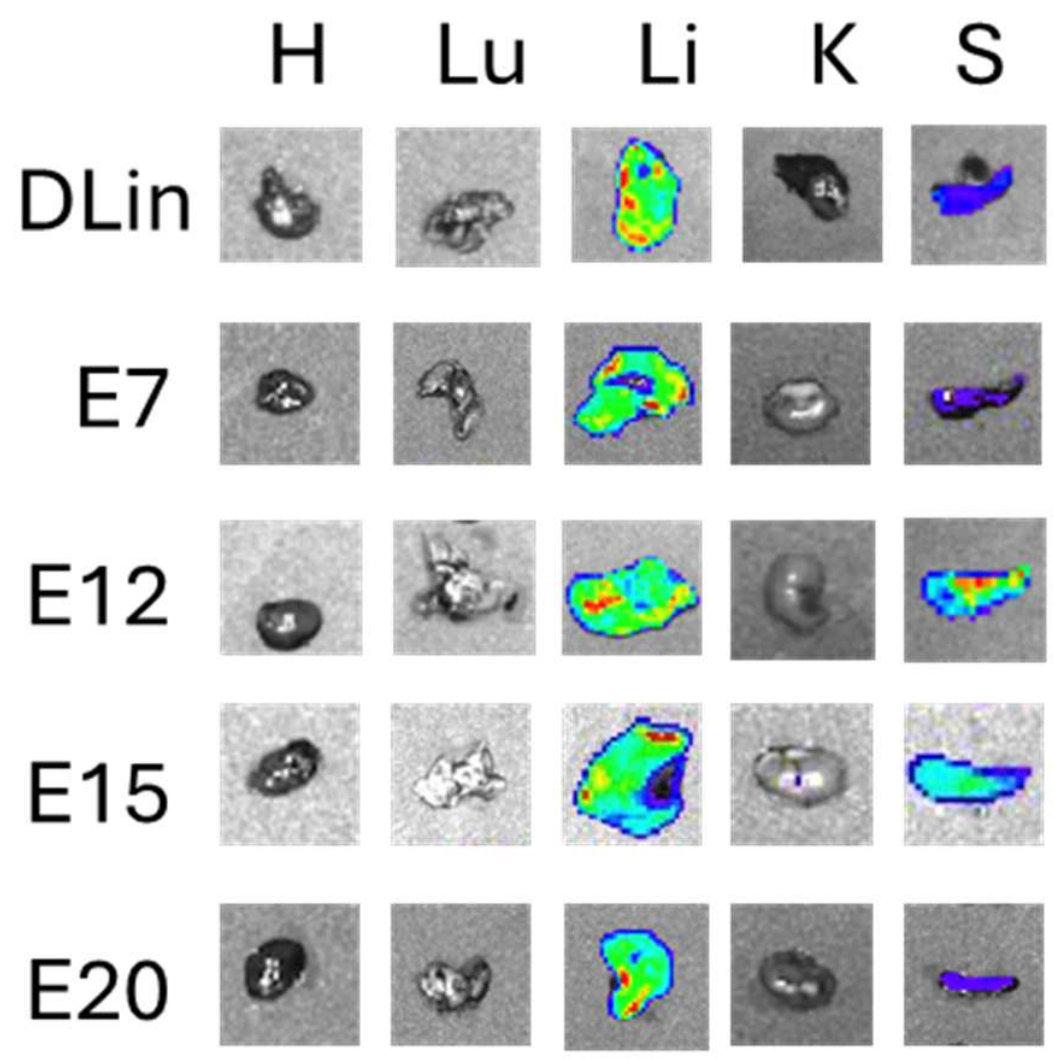

Figure S87. In vivo luciferase luminescence intensity of organs (lungs, liver, kidneys, heart, and spleen) treated with SNPs.

Ex vivo images of major organs (H: heart, Lu: lung, Li: liver, K: kidney, and S: spleen) show biodistribution of the luciferase signal post-injection. The SNP formulations shown here have strong hepatic tropism. PBS-injected mice were used as a negative control and showed no observable signal.

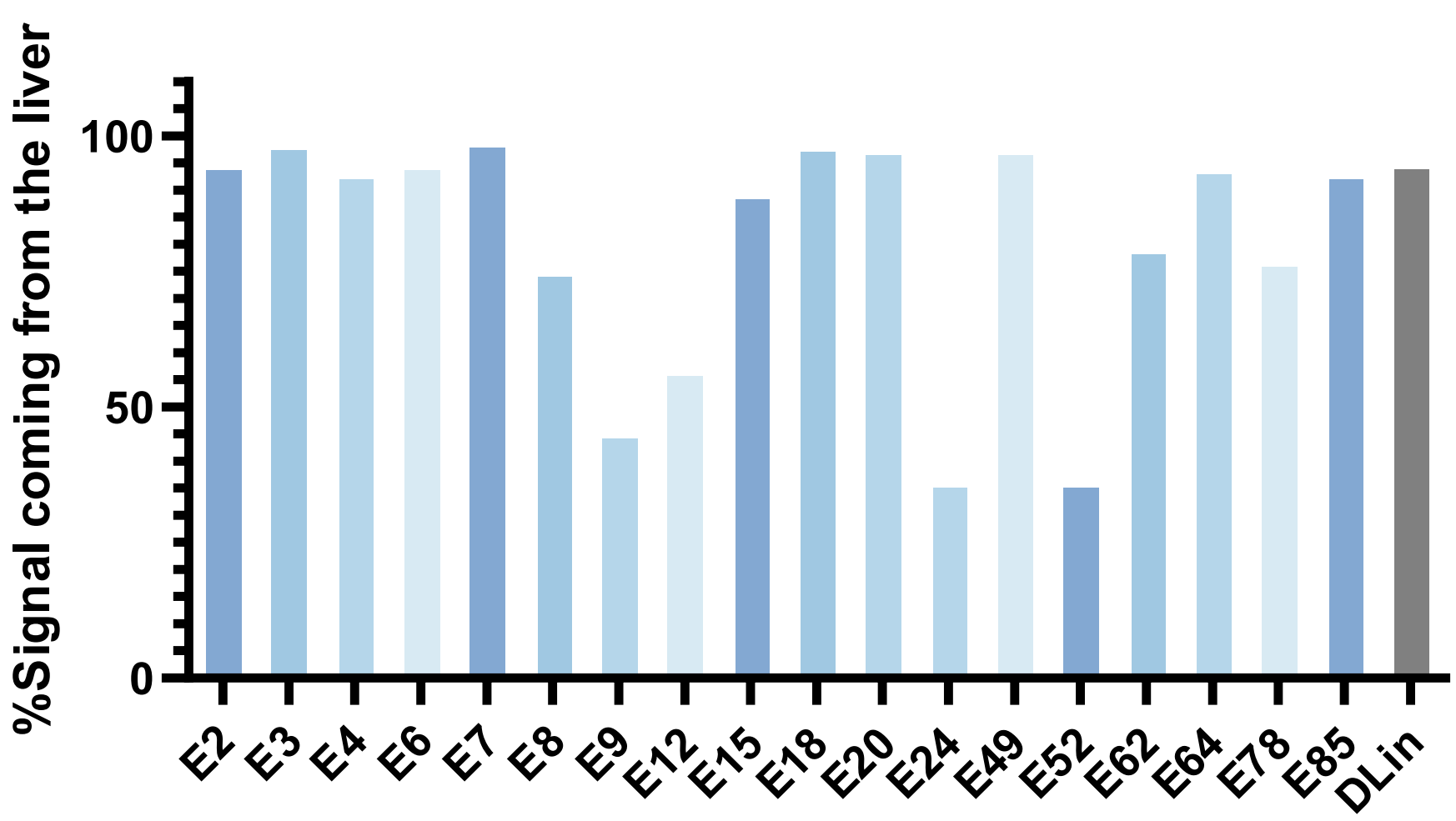

Figure S88. SNPs transfect the liver efficiently.

The organs from the mice treated with SNPs in Figure 3A of the main manuscript were imaged ex-vivo in an IVIS imaging machine and the percentage of the signal coming from the liver was quantified. Several SNPs delivered luciferase mRNA efficiently to the liver with selectivity (n = 1 biological independent replicates). Whole-body IVIS is shown in Figure S86, and organ-level IVIS is shown in Figure S87.

6.2 *In vivo* firefly luciferase pDNA delivery with SNP/pDNA complexes

Wild-type BALB/c mice weighing between 18–20 g, and aged 10–16 weeks old were used for firefly luciferase pDNA delivery experiments. E20 SNP and Dlin LNP formulations were prepared as described in Section 4.1.2. The ethanol was first evaporated under nitrogen air and then pipetted until the volume was reduced by half. SNP/pDNA complexes were subsequently prepared as described in Section 4.1.4 with luciferase nanoplasmid (pALD-Nanoplasmid-CMV-luc, Aldevron, North Dakota, USA). An aliquot of dialyzed DLin LNP/pDNA or E20 SNP/pDNA complexes containing 100 µg of pDNA was diluted with PBS (pH 7.4) to a final volume of 600 µL. A 120 µL dose (20 µg pDNA, 1.0 mg/kg) was administered intravenously (IV) via retro-orbital injection to 3 mice in each group. A group of 3 mice injected with PBS served as the blank control. At 1, 2, 3, 4, 5, 6, and 7 days post-injection, a D-luciferin solution in PBS pH 7.4 (50 mg/kg) was administered to mice by intraperitoneal (IP) injection. 10 minutes after the D-luciferin injection, the luminescence intensity of the whole body was measured using an IVIS spectrum imager (PerkinElmer, MA, USA).

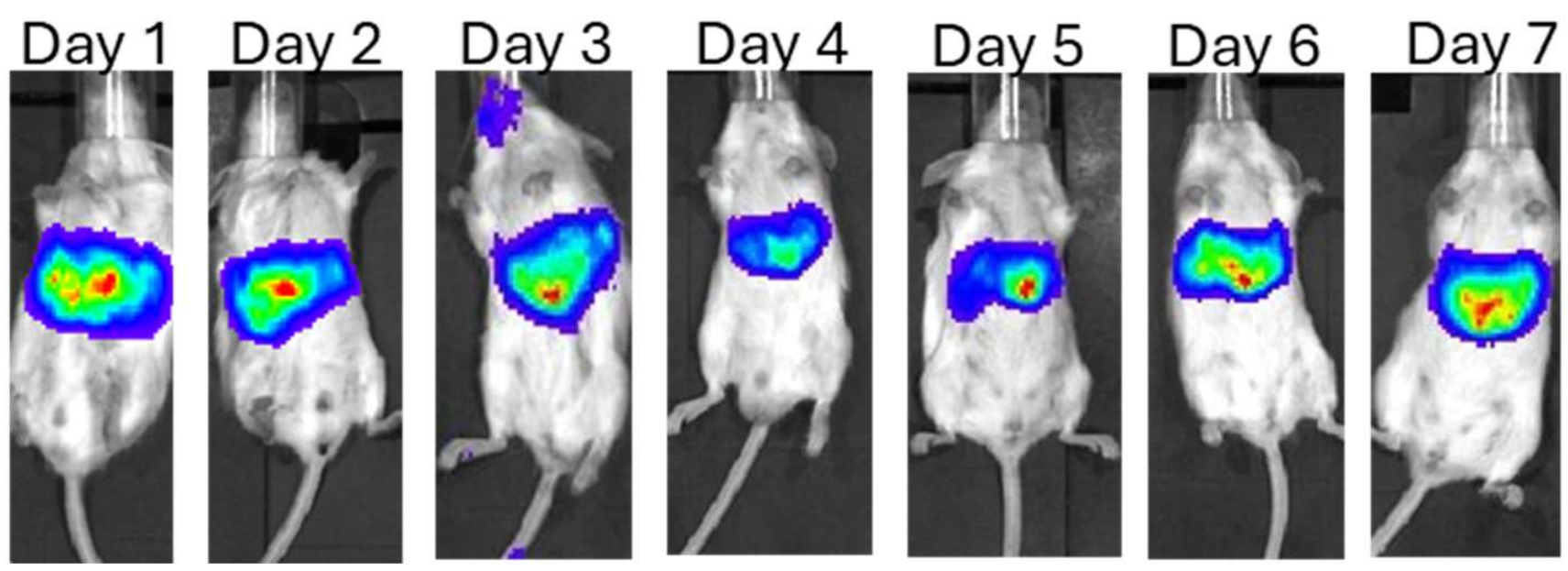

Figure S89. SNPs deliver pDNA efficiently.

Mice were administered intravenously 20 µg pDNA, 1.0 mg/kg at Day 0. At 1, 2, 3, 4, 5, 6, and 7 days post-injection. A D-luciferin solution in PBS pH 7.4 (50 mg/kg) was administered to mice by intraperitoneal (IP). 10 minutes after D-luciferin injection, the luminescence intensity of the whole body was measured using an IVIS spectrum. Representative figures of luminescence intensity were presented.

6.3 *In vivo* human EPO mRNA delivery with SNP/mRNA complexes

Wild-type BALB/c mice weighing between 18–20 g, and aged 10–16 weeks were used for hEPO mRNA delivery experiments. DLin LNP/mRNA and SNP/mRNA complexes were made as described in Section 4.1.4 with hEPO mRNA (RP-A00034-1, GenScript, NJ, USA). An aliquot of dialyzed LNP/ mRNA or SNP/mRNA complexes containing 10 µg mRNA was diluted to 500 µL with PBS (pH 7.4). A 100 µL volume of LNPs (0.1 mg/kg mRNA) was administered

intravenously (IV) via retro-orbital injection. The blood of the mice was collected 24 hours after injection and analyzed using an hEPO ELISA kit (DY286-05, R&D Systems, NE, USA), following the manufacturer's protocol.

6.4 Rescue of mice from acute lung injury with SNPs

The therapeutic potential of E20 SNP/IL-22 mRNA complexes (custom order from TriLink, San Diego, CA, USA) for treating acute lung injury was investigated. Wild-type BALB/c mice (male and female, Jackson Lab, 000651), 10–16 weeks old and weighing between 18 and 20g, were used in this experiment. The efficacy of E20 SNP/IL-22 mRNA was compared against LNPs formulated with the ionizable lipids 4A3-SC8 (benchmark traditional lipid), DLin-MC3-DMA (FDA-approved ionizable lipid), SM-102 (FDA-approved ionizable lipid), ALC-0315 (FDA-approved ionizable lipid) and IL-22 mRNA.

Mice were randomly assigned to four groups: LPS + IL-22 mRNA treatment group, untreated control group, LPS only group, and scrambled mRNA control group.

In the treatment group, mice were challenged with 3 mg/kg LPS intratracheally. They were then maintained for 3 hours to stimulate acute inflammatory injury. Subsequently, mice were administered 2.5 mg/kg doses of LNP/IL-22 mRNA complexes intratracheally (E20 SNPs and MC3-DLin, SM-102, ALC-0315, 4A3-SC8 LNPs prepared as described in Section 4.1.4) and were sacrificed 24 hours later.

In the untreated control group, mice received intratracheal PBS and were sacrificed 24 hours later.

In the LPS group, mice were challenged with 3 mg/kg LPS via intratracheal administration. After 3 hours, they were administered PBS intratracheally and were sacrificed 24 hours later.

In the scrambled mRNA control group, mice were challenged with 3 mg/kg LPS via intratracheal administration. After 3 hours, they were administered 2.5 mg/kg doses intratracheally of E20 SNP/luciferase mRNA complexes and were sacrificed 24 hours later.

Blood samples and bronchoalveolar lavage fluid (BALF) from the lower right lung were collected, and the total protein content was analyzed using the bicinchoninic acid assay (BCA). An equal volume (10 µL) of resuspended BALF cell suspension was mixed with 0.4% trypan blue solution (10 µL) and incubated at room temperature for 2 min. Viable cells were quantified using an automated cell counter. Total cell numbers were calculated and reported as cells per mL. Blood samples were analyzed for cytokines by Luminex (Invitrogen, CA, USA) according to the manufacturer’s protocol and IL-22 protein levels were measured by ELISA.

7. Immunostimulatory effects study of SNPs

7.1 Immunostimulation of human PBMCs with LNPs or SNPs

Peripheral blood mononuclear cells (PBMCs) were isolated from fresh leukopaks from 4 healthy donors (STEMCELL Technologies, MA, USA). The leukopak was diluted 1:1 with MACS/EasySep buffer (2% FBS, 2 mM EDTA in PBS) and centrifuged at 100 × g for 15 minutes. The supernatant was discarded, and the cell pellet was resuspended in 50 mL of MACS/EasySep buffer. The suspension was then centrifuged at 400 × g for 5 minutes, and the supernatant was removed again. This washing step was repeated twice. PBMCs were counted using a 3% acetic acid solution containing methylene blue. Cells were cultured in 48-well plates at a density of 5 –10 x 10^5 per well in 200 µL RPMI medium supplemented with 10% FBS and incubated overnight at 37 °C. LNP/mRNA complexes containing 10 µg of firefly luciferase mRNA (GenScript, NJ, USA) were formulated as described in Section 4.1.4 and diluted with PBS to a total volume of 100 µL. Aliquots of 2, 4, or 8 µL of this dilution were further diluted with Opti-MEM to a final volume of 200 µL. These dilutions were added to each well without removing the existing culture medium, resulting in final mRNA concentrations of 0.5 µg/mL, 1 µg/mL, or 2 µg/mL, respectively. Cells were incubated at 37 °C in a humidified atmosphere with 5% CO_2 for 24 hours or 48 hours. After incubation, the cells were pelleted by centrifugation at 1000 × g for 10 min at 4 °C. The supernatants were collected and stored at −80°C until cytokine analysis. Cytokine levels were measured using ELISA kits (R&D Systems, NE, USA) according to the manufacturer's instructions. The PBMC pellets were collected, and their Luciferase expression was tested using the method described in Section 4.2.

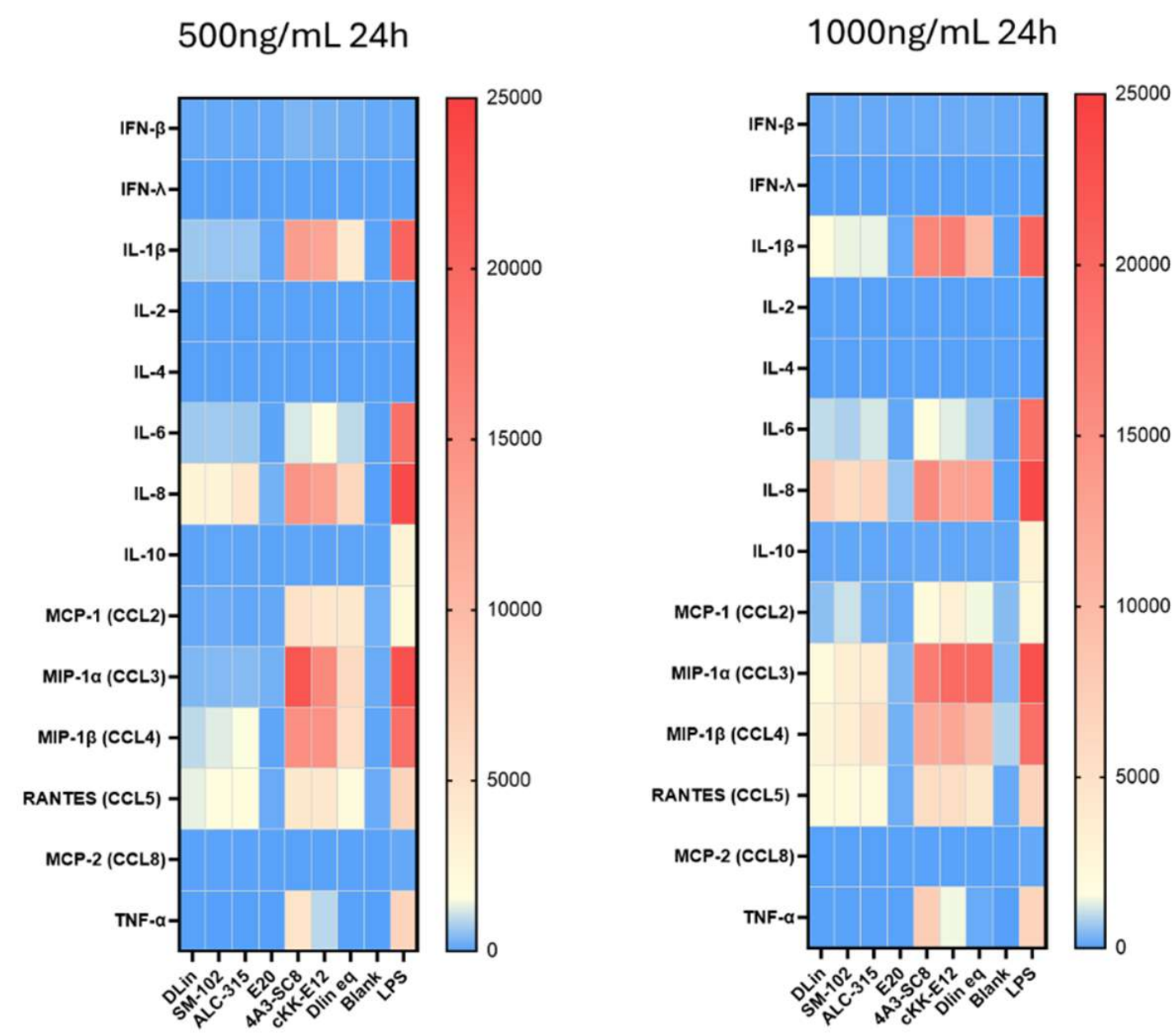

Figure S90. Heat map of cytokine levels in PBMCs (4 healthy donors) treated with E20-SNPs or traditional LNPs (500 ng/mL and 1000 ng/mL mRNA) for 24 hours.

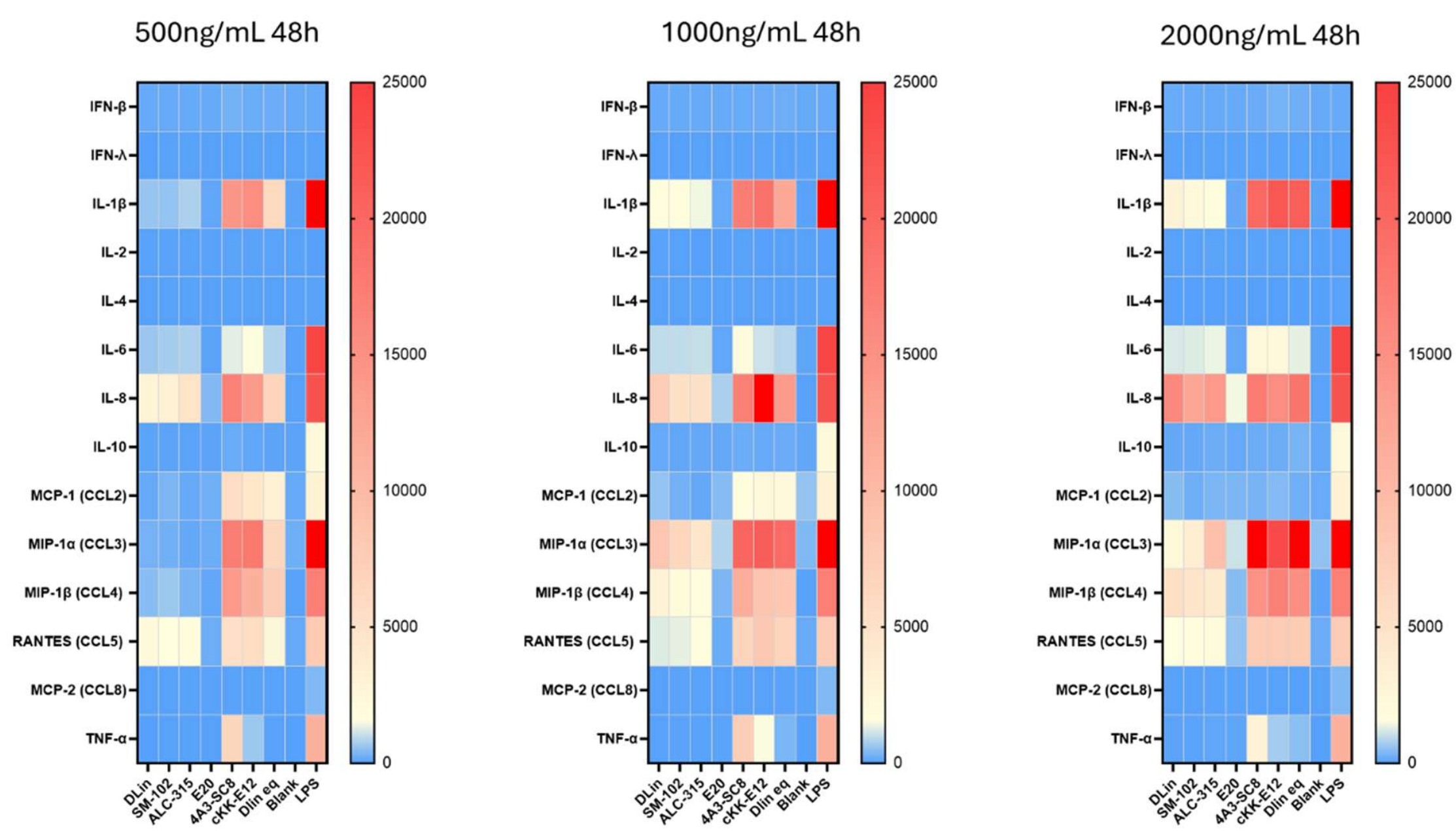

Figure S91. Heat map of cytokine levels in PBMCs (4 healthy donors) treated with E20-SNPs or traditional LNPs (500, 1000, and 2000 ng/mL mRNA) for 48 hours.

The neutrophil content in the PBMC preparation was quantified using flow cytometry. Cells were stained with fluorophore-conjugated anti-human CD66b antibodies (BioLegend Cat.

No. 305103, supplied at 200 µg/mL) and APC anti-human CD15 antibodies (BioLegend Cat. No. 323007, supplied at 100 µg/mL) according to the manufacturer's instructions. After staining, cells were washed and flow cytometry analysis. Flow cytometry analysis (Figure S92) showed that neutrophils, defined as CD15⁺CD66b⁺ double-positive cells, represent a low-frequency population (0.046% of total cells).

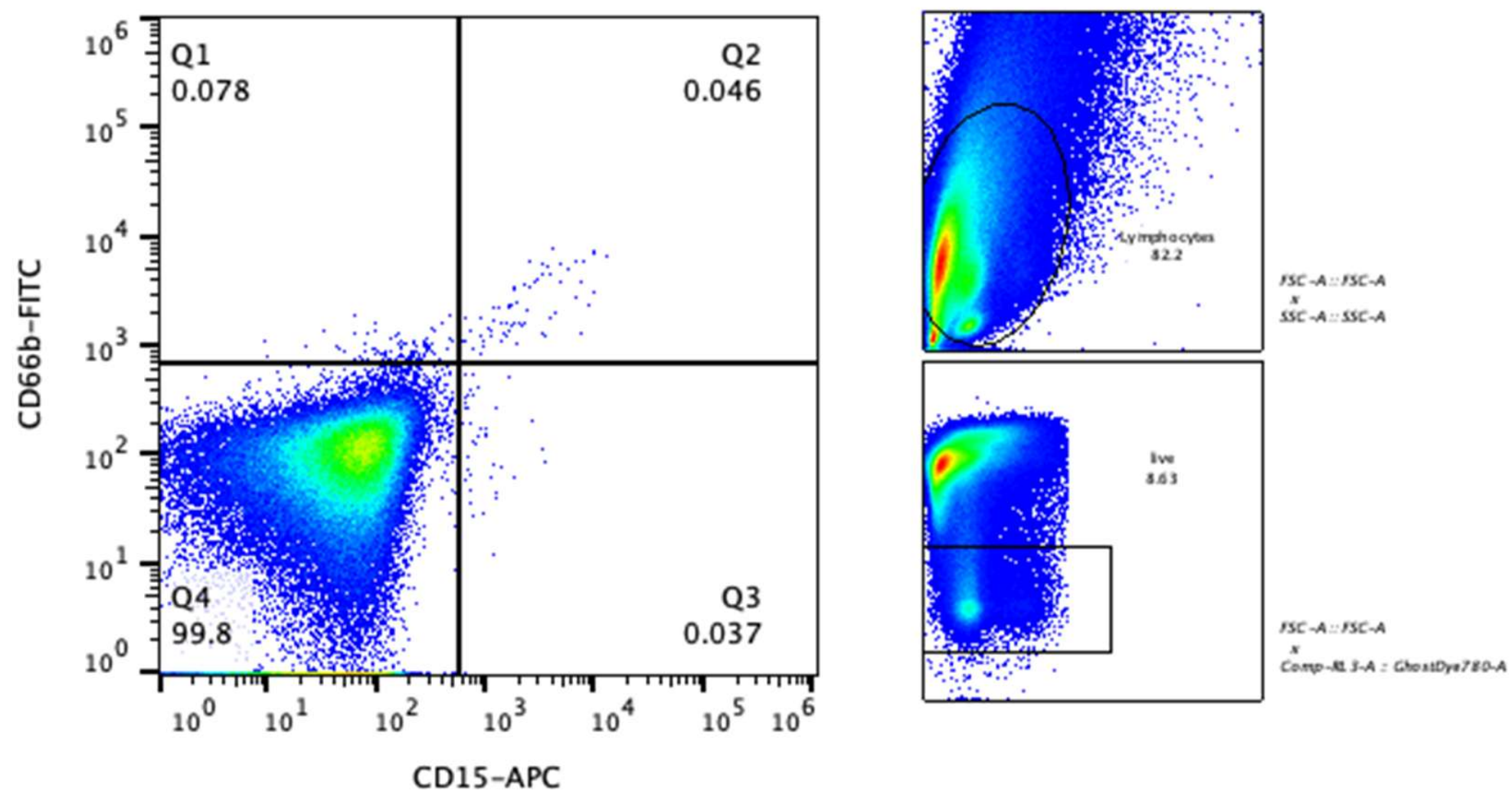

Figure S92. Analysis of neutrophil content in human PBMC preparations
Flow cytometry showed that neutrophils, defined as CD15 and CD66b double-positive cells, represent a low-frequency population in the human PBMC preparations used in this manuscript (0.046% of total cells).

7.2 Internalization of LNPs and SNPs by human PBMCs

Human PBMCs were isolated as described in Section 7.1. Cells were cultured in 96-well plates at a density of 2.5 – 5 x 10^5 cells per well in 100 µL RPMI medium with 10% FBS and incubated overnight at 37 °C. LNP/mRNA complexes containing Cy5-labeled mRNA (Catalot R1010, Apexbio Technology) were prepared as described in Section 4.1.4 and added to cells to generate a final concentration of 2000 ng/mL mRNA. Following 2, 4, and 6 hours of incubation, cells were centrifuged at 1000g for 10 minutes and washed with 200 µL of PBS 7.4 three times. Cy5 fluorescence was quantified by flow cytometry to assess cellular uptake.

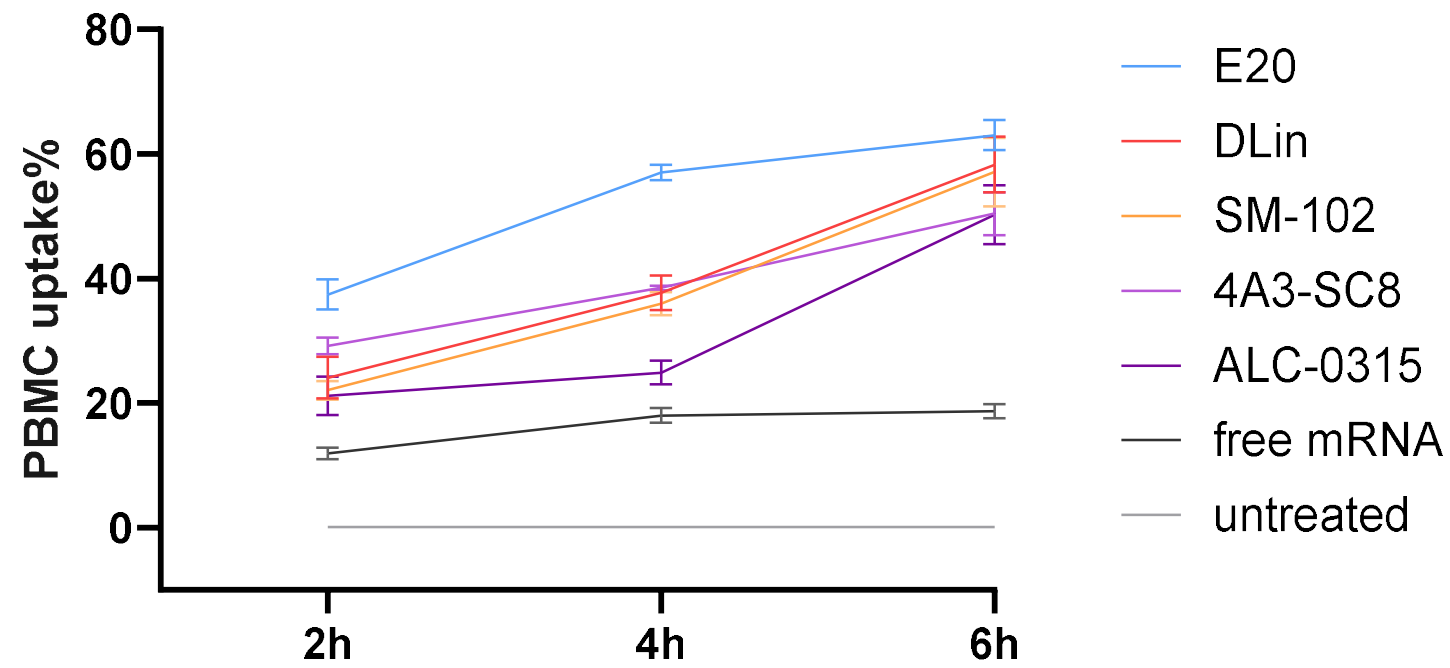

Figure S93. Internalization of E20 SNPs and LNPs by human PBMCs.
E20 SNPs are efficiently internalized by human PBMCs.

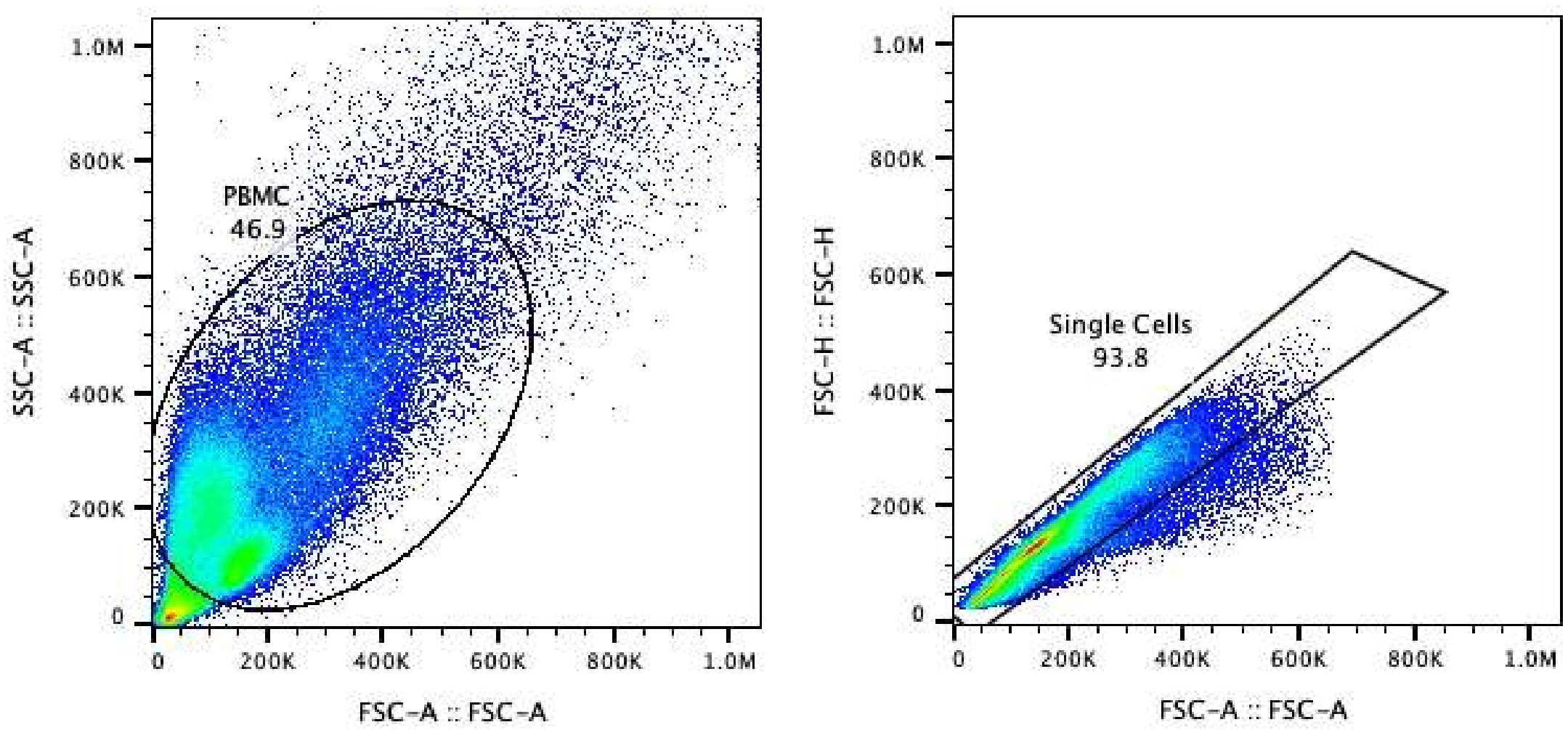

Figure S94. Flow cytometry gating strategy for assessing PBMC uptake of LNPs and SNPs.

7.3 Immunostimulatory effects of SNP/mRNA complexes in LPS-treated mice

Wild-type BALB/c mice and C57BL/6 mice weighing between 18–20 g and aged 10–16 weeks were used to investigate LNP mediated inflammation exacerbation. Lipopolysaccharide (LPS) from E. coli (Sigma, MO, USA) was injected intravenously (retro-orbital IV) into mice at 1 mg/kg. LNP/mRNA complexes were made as described in Section 4.1.4 with luciferase mRNA and dialyzed in PBS at 4 °C for 2 hours. 4 hours after the LPS challenge, LNP/mRNA complexes were retro-orbital intravenous (IV) injection at a dose of 1 mg/kg mRNA. The blood of the mice was collected 4 or 24 hours (BALB/c) and 4 hours (C57BL/6) after LNP/mRNA injection and analyzed using Luminex (Invitrogen, CA, USA), following the manufacturer's protocols.

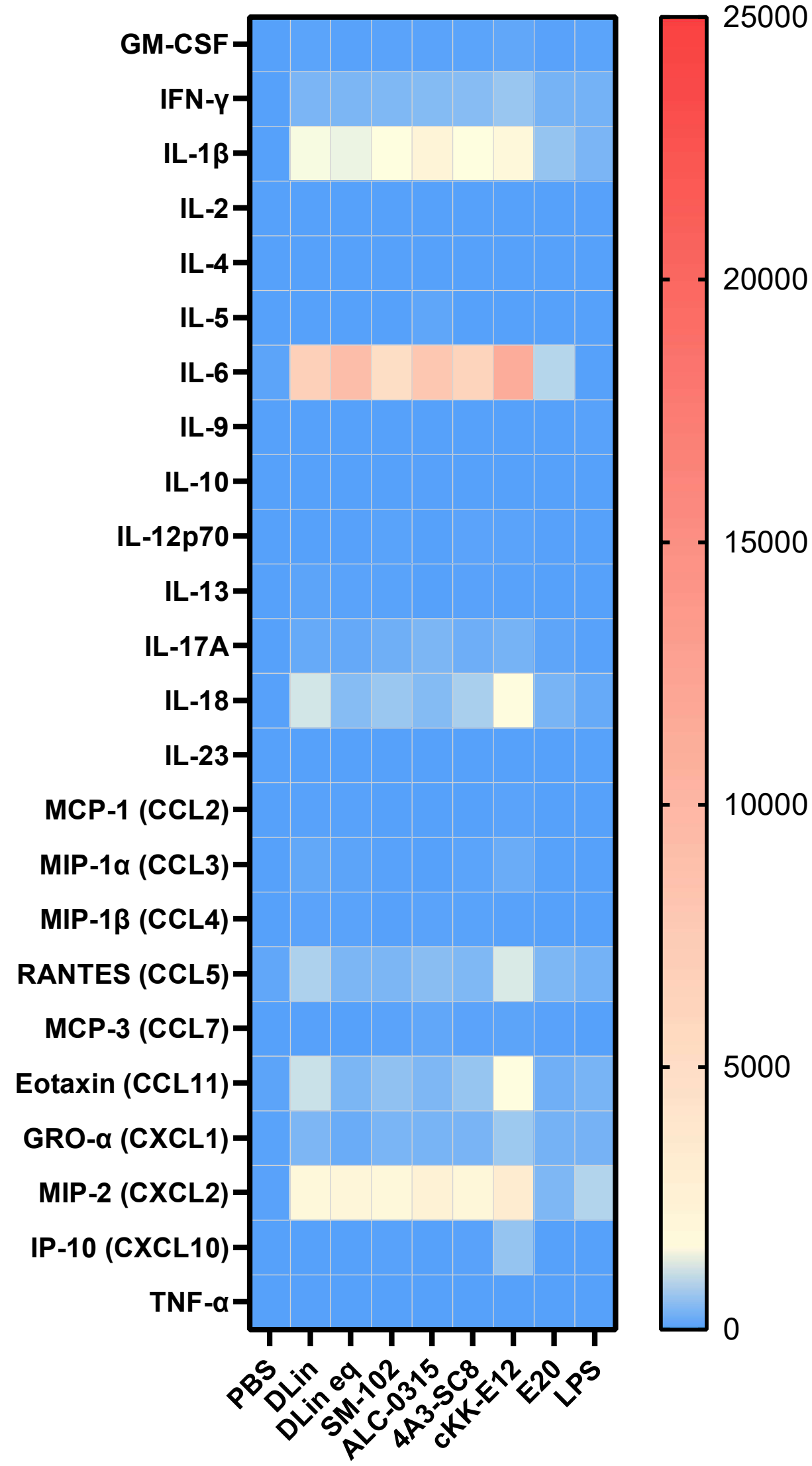

Figure S95. Heat map of cytokine levels in LPS-treated BALB/c mice dosed with E20-SNPs or traditional LNPs (1mg/kg mRNA) for 24 hours.
Blood samples in the LPS-treated mice experiment were collected 24 h after injection of LNPs or SNPs and were analyzed for cytokine levels by Luminex (n = 3 biologically independent replicates, reduced in groups where animals died)

In a separate experiment, mice were treated as described above and administered D-luciferin (50 mg/kg in PBS, pH 7.4) via intraperitoneal (IP) injection 4 hours after the injection of LNPs or SNPs. After 10 minutes, the luminescence intensity was imaged using an IVIS spectrum imager (PerkinElmer, MA, USA). Results are shown in Figure S96.

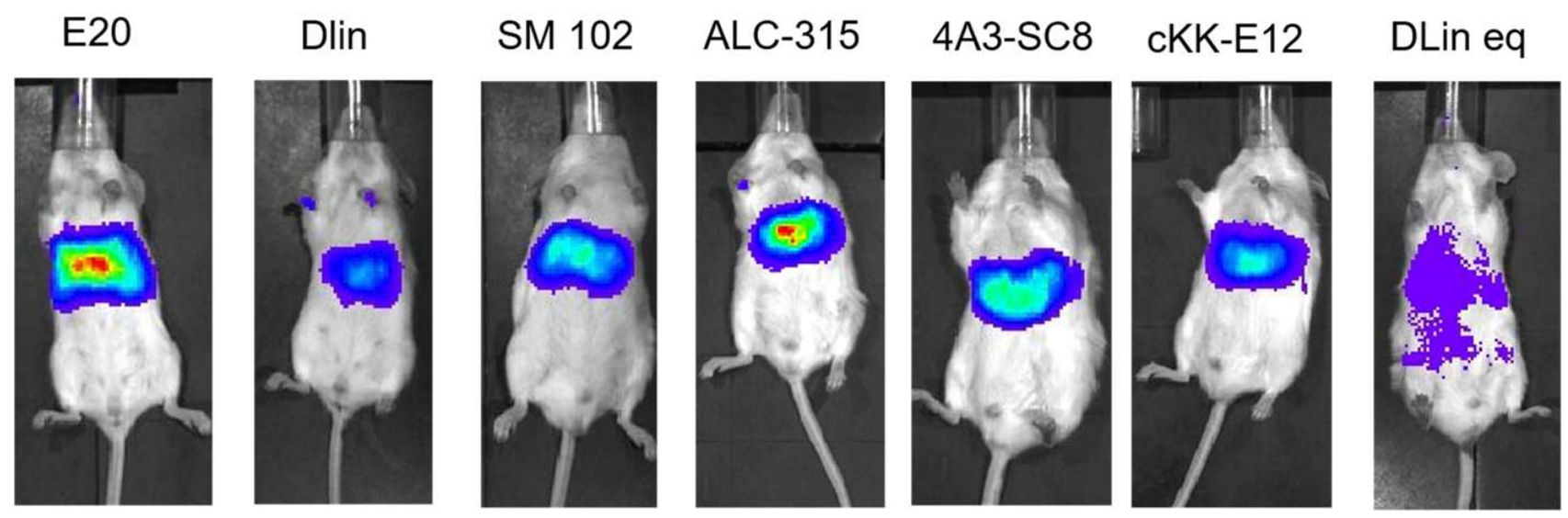

Figure S96. In vivo luminescence image in LPS-pre-treated mice dosed with LNPs.

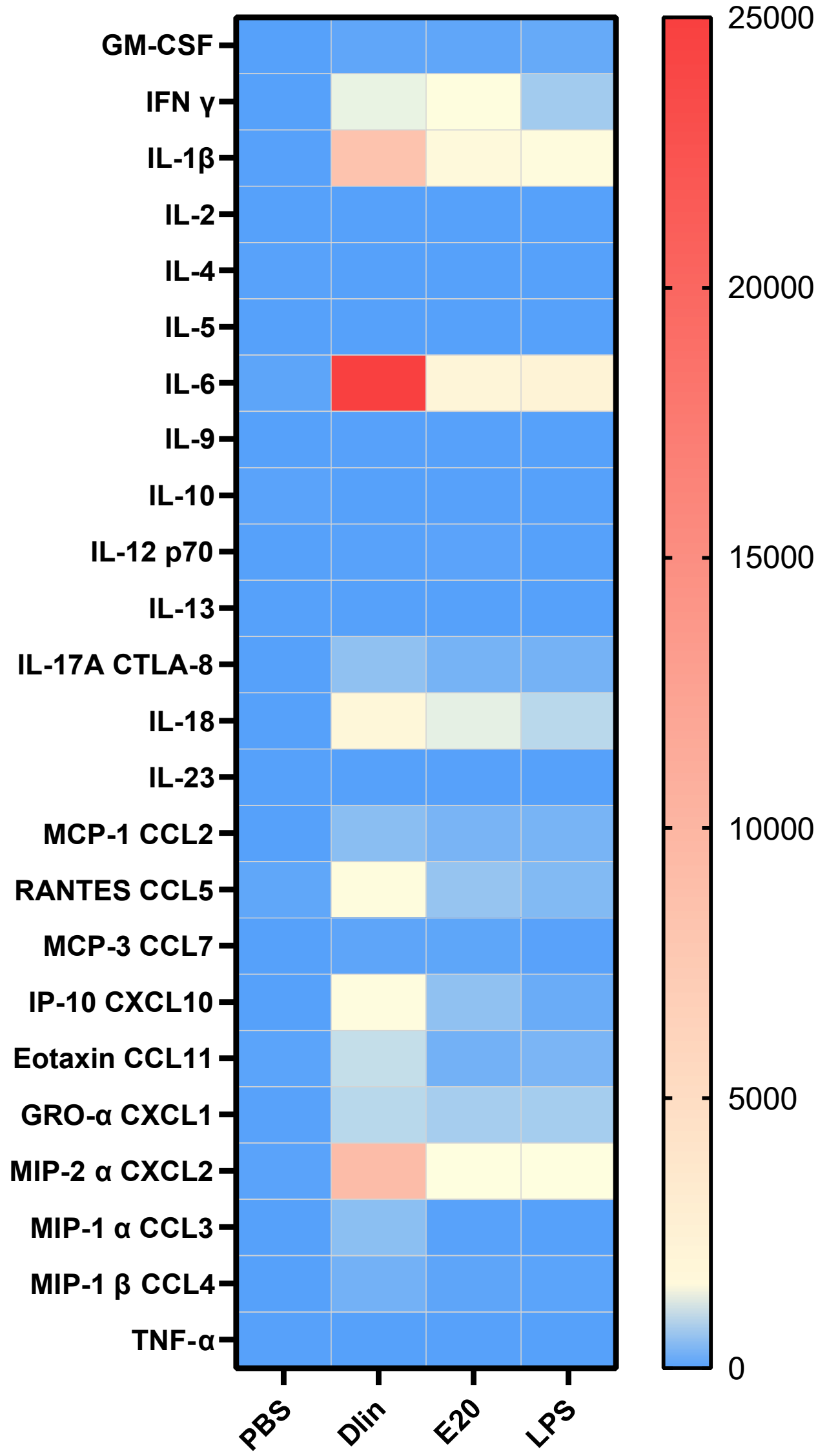

Figure S97. E20 SNPs do not exacerbate inflammation in C57BL/6 mice.
Blood samples in the LPS-treated mice experiment were collected 4 h after injection of LNPs or SNPs and were analyzed for cytokine levels by Luminex (n = 3 biologically independent replicates)

7.4 Immunostimulatory effects of SNP/pDNA complexes in mice

Wild-type BALB/c mice weighing between 18–20 g, and aged 10–16 weeks were used for firefly luciferase pDNA delivery experiments. LNP and SNP formulations were prepared as described in Section 4.1 except luciferase nanoplasmid (pALD-Nanoplasmid-CMV-luc, Aldevron, North Dakota, USA) was used instead of mRNA. An aliquot of dialyzed DLin LNP/pDNA or E20 SNP/pDNA complexes containing 100 µg of pDNA was diluted with PBS (pH 7.4) to a final volume of 600 µL. A 120 µL dose (20 µg pDNA, 1.0 mg/kg) of pDNA/LNP complexes was administered intravenously via retro-orbital injection to 3 mice for each group. A group of 3 mice injected with PBS served as the blank control. Mice were monitored and a survival curve was recorded. Mice were sacrificed after 7 days of observation, and their blood and organs were collected. All three mice in the DLin treated group died and we could not collect their blood, but their organs were collected. The cytokine levels in the serum of SNP treated mice were analyzed by Luminex (Invitrogen, CA, USA) according to the manufacturer's protocol. Liver damage was assessed by measuring the Alanine aminotransferase (ALT) activity in the serum, using a fluorometric kit (AB105134-1001). The heart, lung, liver, spleen, and kidney histology were analyzed to further evaluate organ damage.

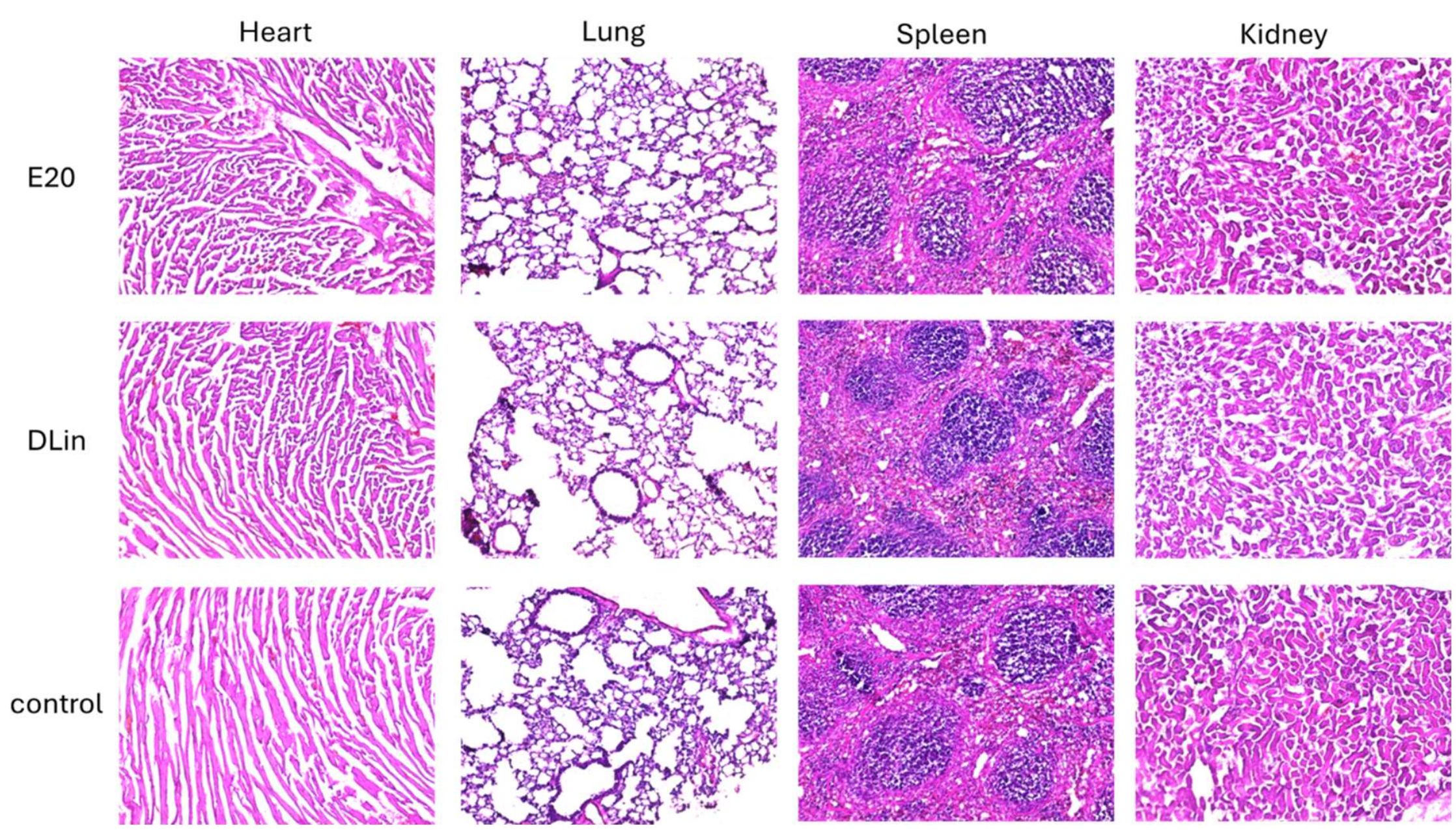

Figure S98. Histology of heart, lung, spleen, and kidney in mice dosed with E20 SNPs, DLin LNPs, or PBS.

No obvious organ damage was observed in the heart, lung, spleen, or kidney. Liver histology is shown in Extended Figure 2A in extended data. Images are representative of 3 independent experiments with similar results.

7.5 Endosomal membrane disruption study by Gal-8 reporter cells

Gal8-YFP-MDA-MB231 cells were graciously provided by Dr. Craig L. Duvall. Cells were cultured in a 96-well plate at a density of 20,000 cells/mL in 100 µL Dulbecco's Modified Eagle Medium (DMEM) with 10% FBS per well and incubated at 37 °C overnight. LNP/mRNA complexes containing 10 µg of firefly luciferase mRNA (GenScript, NJ, USA) were formulated as described in Section 4.1.4 and diluted with PBS to a total volume of 400 µL. Aliquots of 0.8, 4, or 8 µL of this dilution were mixed with Opti-MEM to a final volume of 100 µL. The resulting formulations were then added to each well without removing the existing culture medium, generating solutions with mRNA concentrations of 0.1 µg/mL, 0.5 µg/mL, and 1 µg/mL. We did not titrate to a higher concentration because the YFP punctures merged together and difficult to count even at 1 µg/mL of mRNA. Cells were incubated with LNPs or SNPs for 4 hours. After incubation, the supernatants were aspirated, and the cells were washed three times with PBS at pH 7.4. Cells were fixed in 10% formalin for 24 hours, and the nuclei were stained with DAPI.

Fluorescence microscopy was then performed, and the number of puncta per cell was quantified using a custom written Python script in combination with the PyImageJ package. A hybrid puncta-detection workflow, similar to that described in PunctaFinder[7], was used to optimize parameter selection. First, each image was converted to 8-bit, and the lowest 75% of pixel intensities were suppressed. A sliding-window approach with patch diameters of 3, 5, and 7 pixels was used to identify puncta candidates. For each candidate patch, intensity statistics were also collected from a surrounding ring of 2 pixels. Global image mean intensity and background noise standard deviation were calculated, with noise estimated from a 64 × 64 pixel region in each image corner. Candidate puncta were retained only if the patch mean intensity exceeded the ring mean by at least 33% or 5 intensity units, whichever criterion was more stringent. In addition, each patch was required to contain a central peak that was at least 0.78 background noise standard deviations above the patch mean, to show a correlation of at least 0.25 with an ideal Gaussian spot, and to satisfy a maximum local coefficient of variation threshold to exclude irregular intensity distributions. A mean-intensity prefilter was also applied so that candidate patches were required to exceed the estimated local background noise by a minimum multiplicative factor and offset, which reduced retention of dim fluctuations consistent with background. To exclude elongated cellular structures such as filopodia, Otsu thresholding was then applied to the retained patches to segment candidate puncta. Segmented objects smaller than 6 px^2 or larger than 130 px^2 were discarded. Finally,

overlapping detections were filtered so that candidates with substantial shared areas were not counted multiple times. To count cells by DAPI staining, 8-bit images were binarized in ImagePro, and the standard "Count/Size" function was used to count bright objects with automatic intensity selection. Eight views per condition (100, 500, 1000 ng/mL) were imaged. To get the average number of puncta per cell, we divided the number of Gal-8 puncta observed per view by the number of DAPI-positive nuclei per view.

The luciferase mRNA delivery efficiency of the SNPs to Gal8 reporter cells was also investigated. Cells were incubated with SNPs and LNPs (0.1 µg/mL, 0.5 µg/mL, and 1 µg/mL mRNA) for 17 h at 37 °C and the luciferase expression level was quantified as described in Section 4.2.

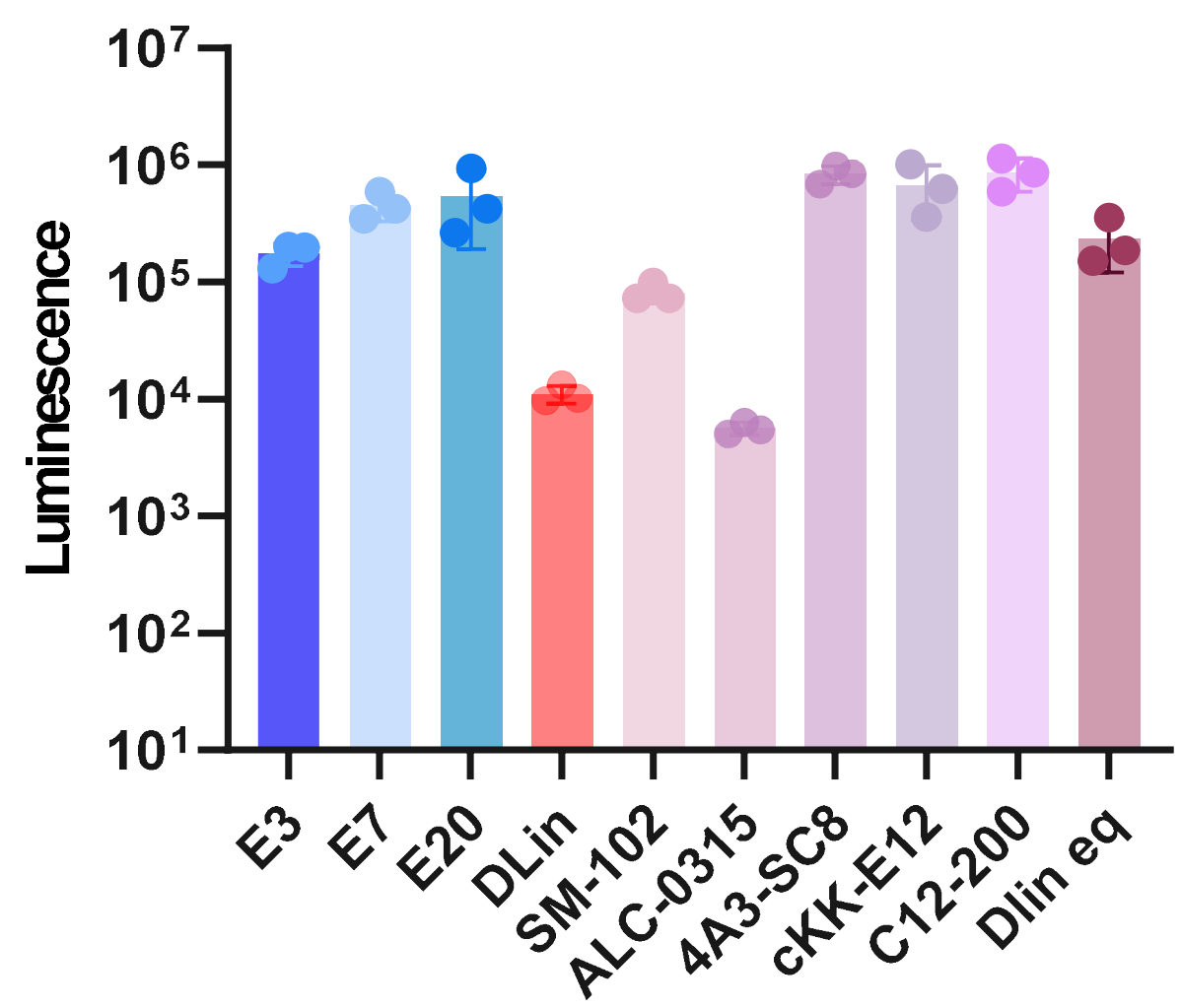

Figure S99. SNPs deliver luciferase mRNA (100 ng/mL) to Gal-8 reporter cells efficiently.

SNPs were formulated with luciferase mRNA (100 ng/mL) and delivered to Gal-8 reporter cells, and their efficacy was compared against a panel of traditional LNPs. SNPs deliver mRNA to Gal-8 reporter cells with similar efficiency as traditional LNPs (n = 3 biological independent replicates).

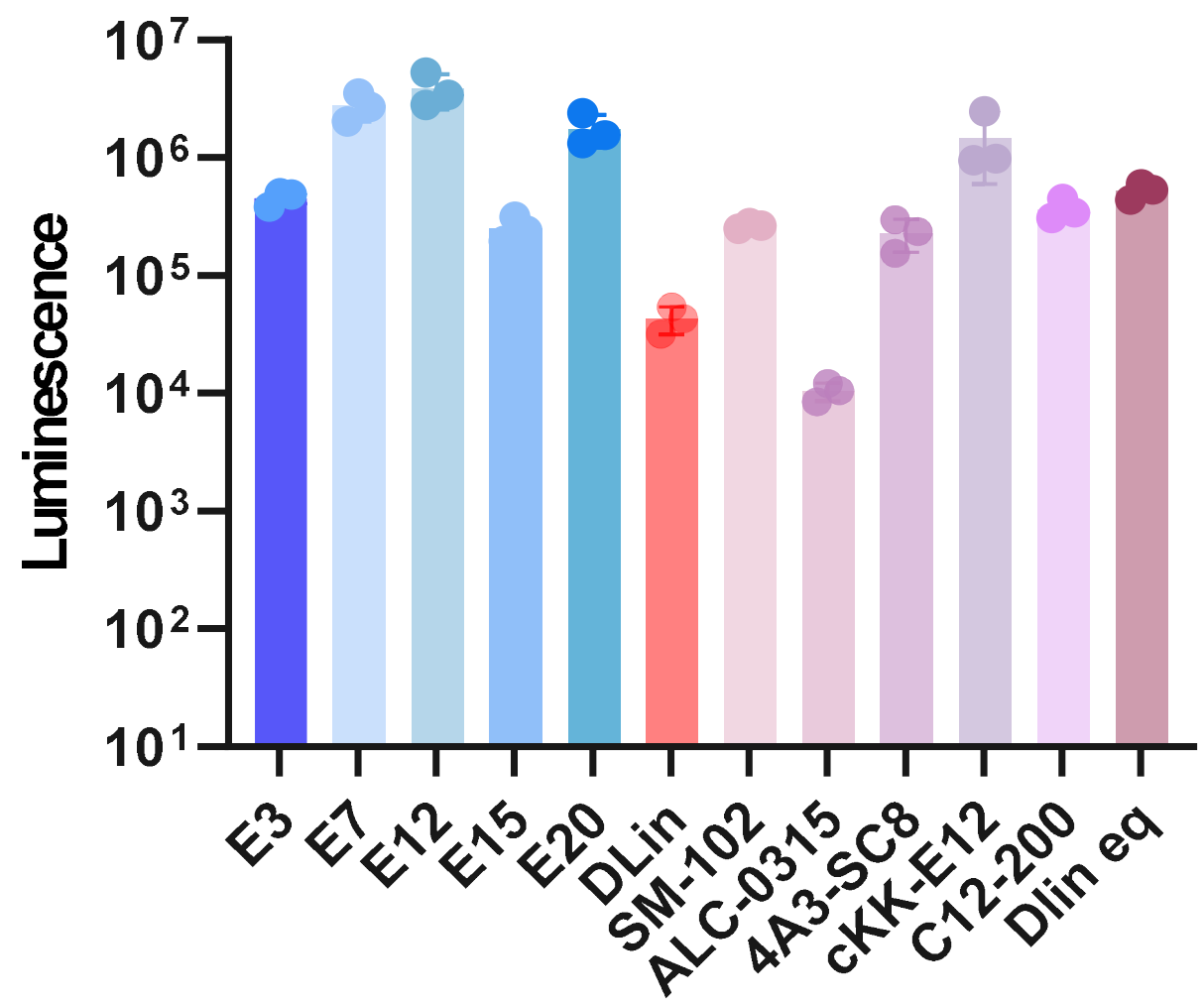

Figure S100. SNPs deliver luciferase mRNA (500 ng/mL) to Gal-8 reporter cells efficiently.

SNPs were formulated with luciferase mRNA (500 ng/mL) and delivered to Gal-8 reporter cells, and their efficacy was compared against a panel of traditional LNPs. SNPs deliver mRNA to Gal-8 reporter cells with similar efficiency as traditional LNPs (n = 3 biological independent replicates).

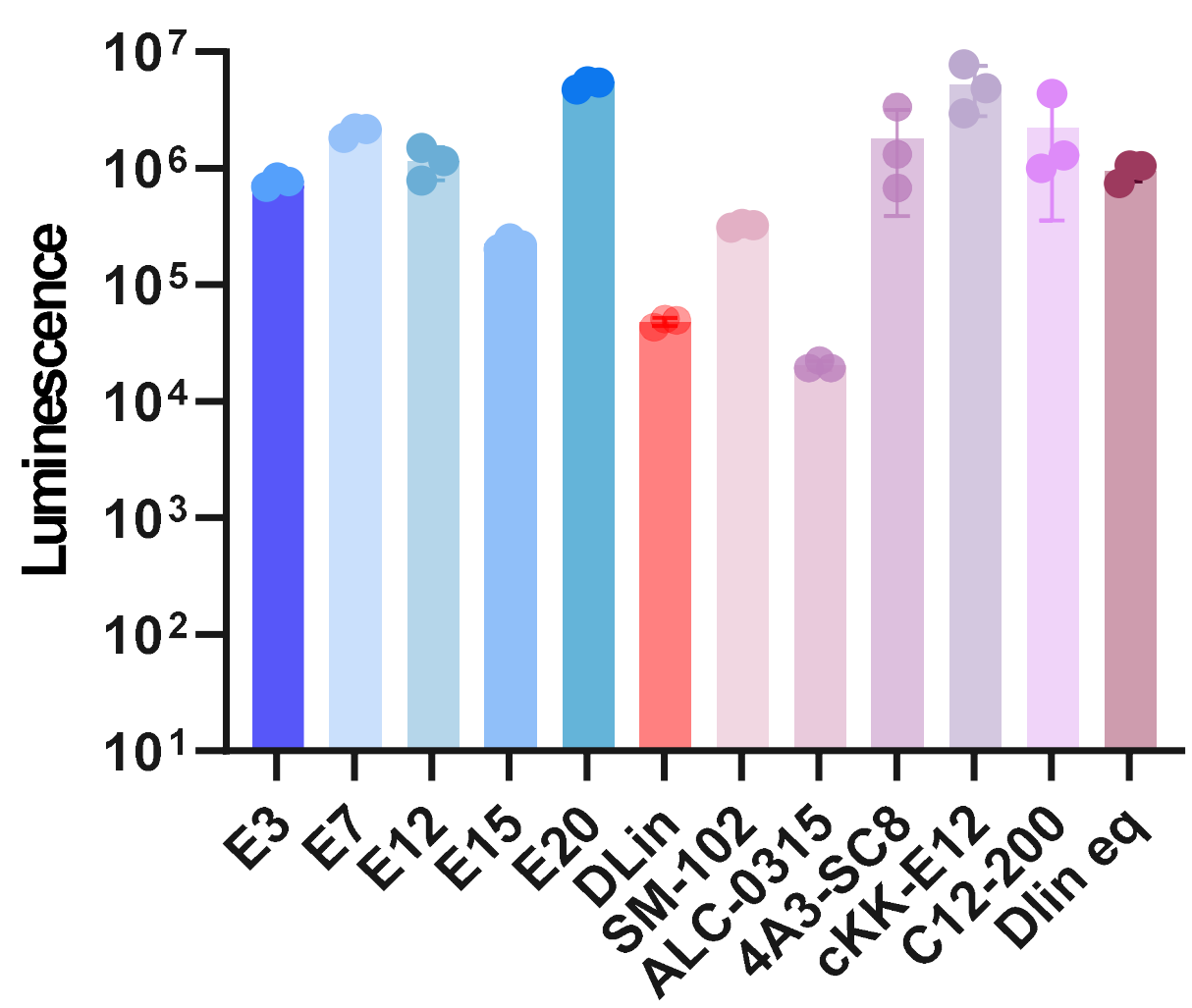

Figure S101. SNPs deliver luciferase mRNA (1000 ng/mL) to Gal-8 reporter cells efficiently.

SNPs were formulated with luciferase mRNA (1000 ng/mL) and delivered to Gal-8 reporter cells and their efficacy was compared against a panel of traditional LNPs. SNPs deliver mRNA to Gal-8 reporter cells with similar efficiency as traditional LNPs (n = 3 biological independent replicates).

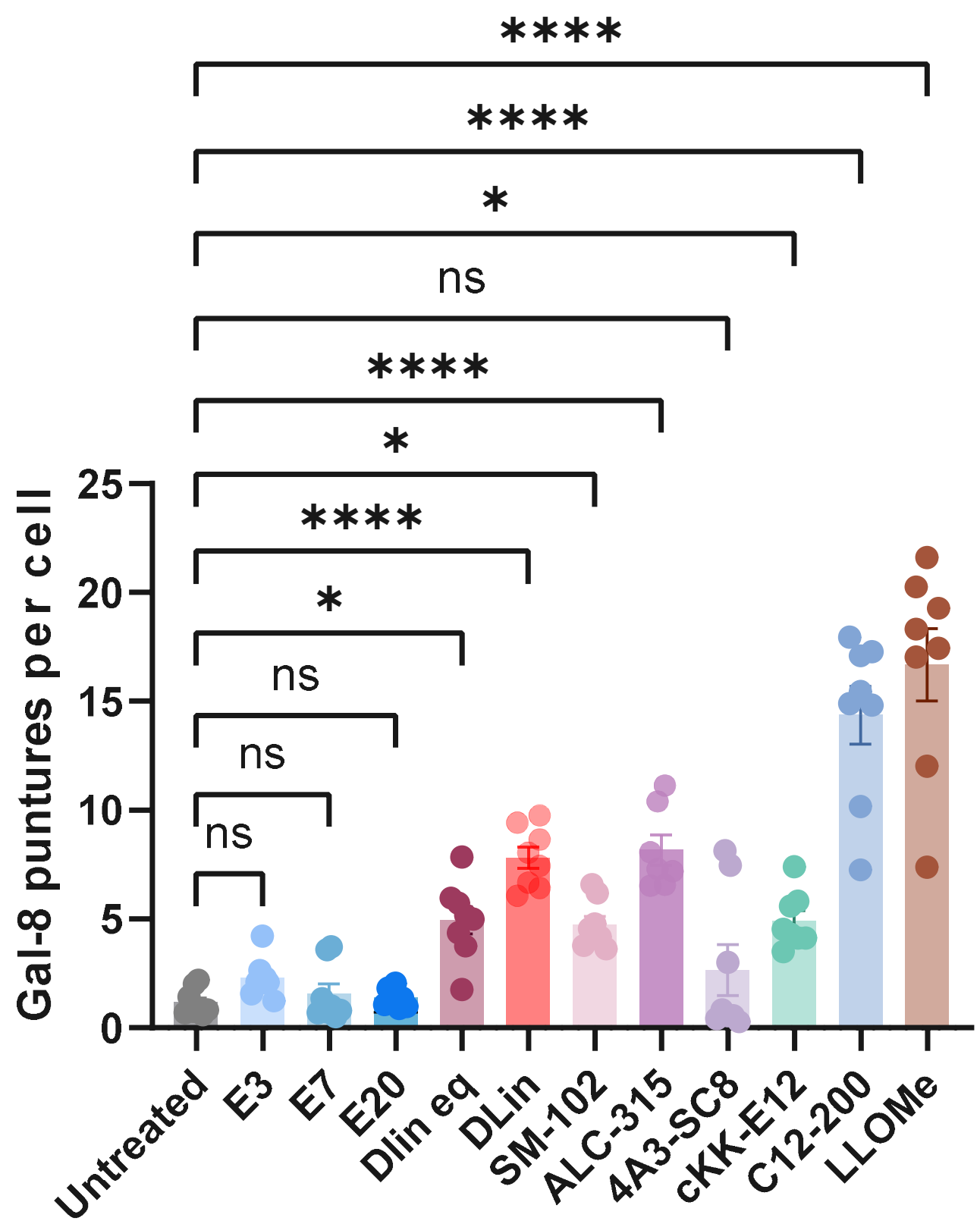

Figure S102. SNPs do not recruit galectin-8 to endosomes (100 ng/mL mRNA). E20-SNPs caused minimal galectin-8 recruitment to endosomes at an mRNA concentration of 100 ng/mL, whereas traditional LNPs caused substantial levels of galectin-8 recruitment to endosomes (n = 8 views from 3 biologically independent replicates).

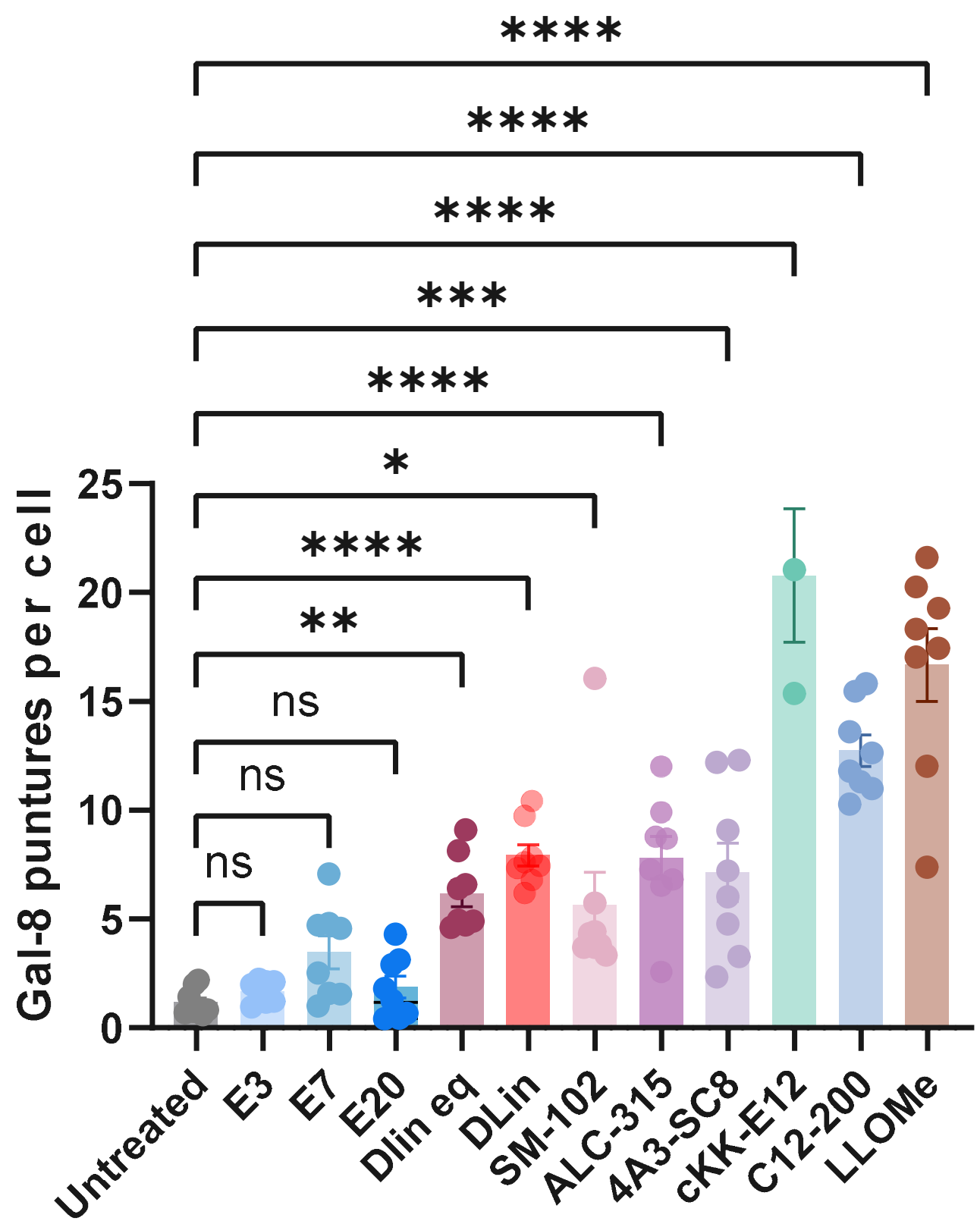

Figure S103. SNPs do not recruit galectin-8 to endosomes (500 ng/mL mRNA). E20-SNPs caused minimal galectin-8 recruitment to endosomes at an mRNA concentration of 500 ng/mL, whereas traditional LNPs caused substantial levels of galectin-8 recruitment to endosomes (n = 8 views from 3 biologically independent replicates).

7.6 Bafilomycin inhibition of SNP and LNP transfection

Gal8-YFP–MDA-MB-231 cells were cultured in 96-well plates at a density of 20,000 cells/mL and incubated at 37 °C overnight in 100 µL Dulbecco's Modified Eagle Medium (DMEM) with 10% FBS per well. LNP/mRNA complexes were formulated as described in Section 4.1.4. Formulations containing 10 µg of luciferase mRNA (GenScript, NJ, USA) were collected from the dialysis kit and diluted with PBS to a final volume of 400 µL. Aliquots of 8 µL of this dilution were mixed with Opti-MEM to a generate a final volume of 100 µL. These dilutions were added to each well without removing the existing culture medium, resulting in final mRNA concentration of 1 µg/mL. Cells were pretreated for 30 min with either bafilomycin A1 (100 nM final concentration) or PBS, and the media was then aspirated. LNPs or SNPs were added to the cells to generate a 1 µg/mL mRNA concentration and were incubated at 37 °C for 17 h. After incubation, luciferase expression was quantified as described in Section 4.2.

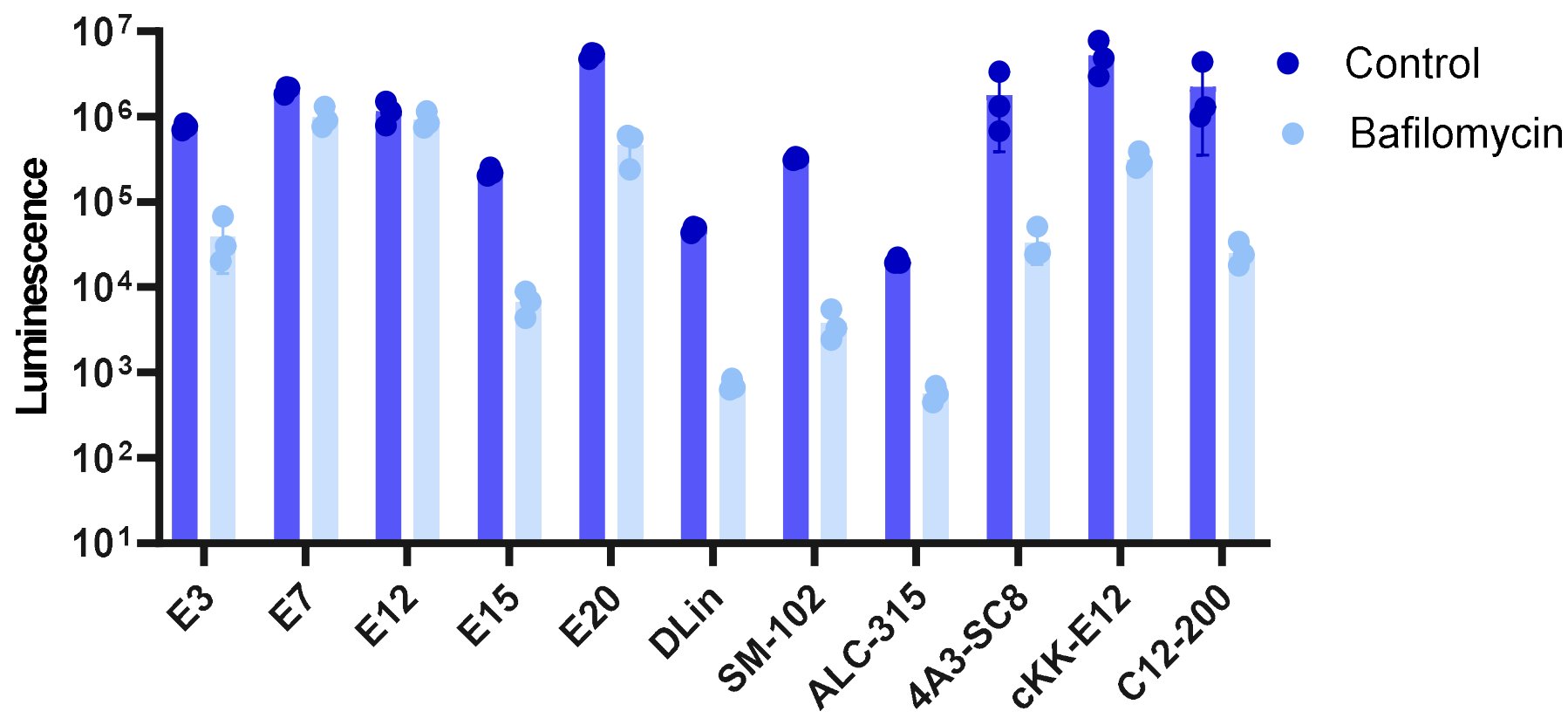

Figure S104. Bafilomycin inhibition of SNP/LNP transfection.
Bafilomycin inhibits SNPs' transfections of Gal8-YFP–MDA-MB-231 cells (n = 3 biologically independent replicates).

7.7 Hemolysis of red blood cell membranes with LNPs and SNPs

A 5% (v/v) RBC Solution was prepared by diluting 0.5 mL of defibrinated rabbit RBCs (Hemostat Laboratories, Dixon, CA, USA) in 9.5 mL of PBS at the desired pH (7.4 or 5.5). The suspension was centrifuged at 4°C (400g, 5 min) and the supernatant was removed. This process was repeated three additional times, with the pellet diluted and resuspended in PBS to a total volume of 10 mL each time. After the last centrifugation, the supernatant was discarded, and 100 µL of the collected RBC pellet was diluted in 9.9 mL of PBS at the desired pH, generating a 1% (v/v) solution.

SNPs were prepared as described in Section 4.1 and were dissolved in PBS at the desired pH at a 500 µM concentration of total lipids. 100 µL of each LNP solution was mixed with 50 µL of the 1% RBC solution (at the same pH, described above) and added to 200 µL PCR tubes, which were then capped and incubated at 37°C for 30 minutes. Following incubation, the plates were centrifuged at 2,000 g for 10 minutes and a 100 µL of the supernatant was carefully collected and transferred to black optical bottom 96-well plates (Thermo Scientific, Waltham, MA, USA). The absorbance of the solutions was at 546 nm was measured with a plate reader to measure the hemoglobin concentration.

A positive control (complete hemolysis) was prepared by adding 50 µL of the 1% RBC solution to 50 µL of Bioscience RBC Lysis Buffer (Invitrogen, Carlsbad, CA, USA). The solution was incubated at 37°C for 30 minutes, and subsequently 50 µL of PBS at the same pH was added to achieve the same concentration of RBC as the other conditions. A negative control

(no hemolysis) was prepared by adding 50 µL of RBC solution to 100 µL of PBS at the same pH. The solution was then incubated at 37°C for 30 minutes. Positive and negative control solutions were centrifuged at 2,000 g for 10 min, and 100 µL of the supernatant was taken and the absorbance of the solutions was at 546 nm was measured. The LNP solution had a non-negligible impact on absorbance measurements, necessitating the use of a blank control that consisted of LNPs in PBS at the appropriate pH. The controls were analyzed via spectrophotometry in triplicate, and the absorbance was measured as described above.

The absorbance measurements for the positive, negative, and blank controls were averaged. The percent hemolysis was calculated as

$$Hemolysis\% = \frac{Absorbance - Average\ Negative\ Control - Average\ Blank\ Control}{Average\ Positive\ Control - Average\ Blank\ Control}$$

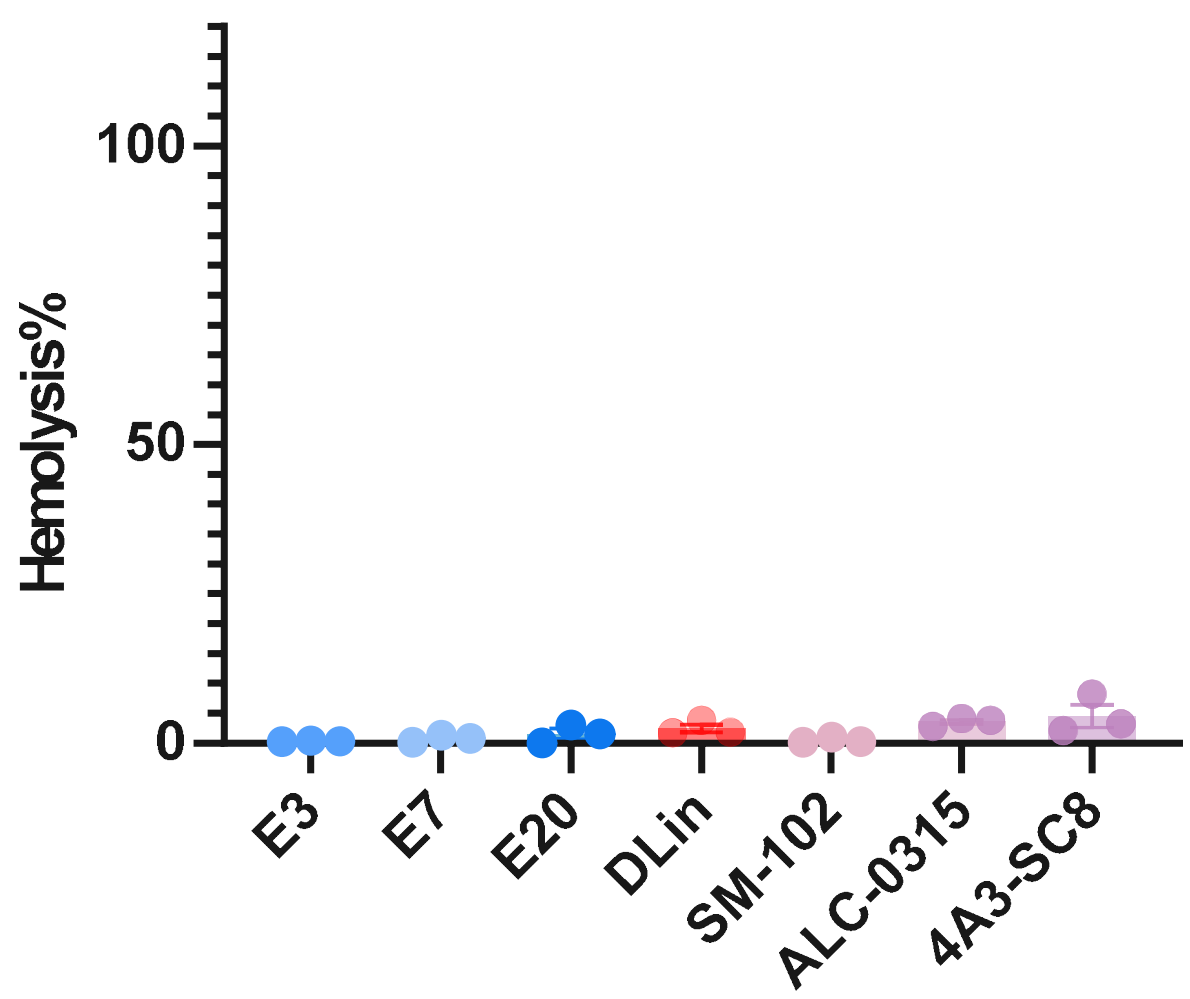

Figure S105. Hemolysis of red blood cell membranes with LNPs and SNPs at pH 7.4
A hemolysis assay at pH 7.4 was performed with SNPs or traditional LNPs at a concentration of 500 µM of lipids (n = 3 biologically independent replicates). At physiological pH, neither SNPs nor traditional LNPs are membrane disruptive.

7.8 Cellular trafficking of SNPs and LNPs

HeLa cells were seeded in confocal dishes with complete medium and incubated overnight. Cy5-labeled SNPs or LNPs (2000 ng/mL mRNA) were added to the dishes with complete medium and incubated for 2 hours, 4 hours, and 12 hours. The cells were then washed with PBS and fixed with 4% paraformaldehyde (PFA). The fixed cells were blocked with 5% BSA for 30 min. Primary antibody dilutions of anti-EEA1 antibody (Abcam, EPR4245, supplied at

1.36 mg/mL), anti-LAMP1 antibody (Abcam, EPR21026, supplied at 0.639 mg/mL), and anti-Rab11 antibody (Abcam, EPR7587(B), supplied at 0.414 mg/mL) were added to the dishes and incubated for 2 h. The primary antibodies were removed and the cells were washed with PBS 3 times, and secondary antibodies were added and incubated for 1 h. Secondary antibodies were removed and washed 3 times with PBS, after which the cells were stained with DAPI and analyzed by confocal microscopy (ImageXpress, San Jose, CA, USA).

7.9 Activation of TLR4 in TLR4 Reporter HEK293 cells with LNPs or SNPs

Human TLR4 Reporter HEK293 Cells were maintained in growth medium supplemented with 10 µg/mL Blasticidin, 200 µg/mL Hygromycin B Gold, 1 µg/mL Puromycin, and 100 µg/mL Zeocin, and this medium was used for routine subculturing.

On Day 0, cells were gently rinsed with pre-warmed PBS and incubated in fresh pre-warmed PBS containing accutase at 37 °C for 1–5 minutes to facilitate detachment by gentle pipetting. The cell suspension was then adjusted to a concentration of 280,000 cells/mL in freshly prepared, pre-warmed test medium. 100 µL of this suspension was dispensed into each well of a 96-well plate and incubated at 37°C in a CO_2 incubator for 20 hours.

On Day 1, LNP/mRNA complexes containing 10 µg GFP mRNA were formulated as described in Section 4.1.4 and diluted with PBS to a total volume of 200 µL. Aliquots of 2, 4, or 8 µL of this dilution were mixed with the cell culture medium to a final volume of 100 µL. The resulting formulations were then added to each well without removing the existing culture medium, resulting in final mRNA concentrations of 0.5 µg/mL, 1 µg/mL, or 2 µg/mL, respectively. The vehicle control was prepared by mixing 10 µL ethanol with 30 µL mRNA in citrate buffer, followed by dialysis against PBS (pH 7.4). Untreated wells served as the baseline control.

On Day 2, TLR4 activation was quantified. A QUANTI-Luc™ 4 Reagent working solution was prepared by diluting 0.75 mL of the 20X stock with 14.25 mL of sterile water, followed by brief vortexing to obtain a 15 mL working solution. For luminescence detection, 20 µL of cell culture supernatant was transferred into each well of a white 96-well plate, and 50 µL of QUANTI-Luc™ 4 working solution was added into each well. Luminescence was recorded immediately using a plate reader, and untreated cells were used as the baseline. The TLR4 activation levels were calculated by normalizing the luminescence values to the untreated group.

7.10 Activation of complement with LNPs or SNPs

Human serum was purchased from STEMCELL Technologies (Cat. No. 200-0159). The serum was prepared by the manufacturer from whole blood collected from healthy donors using serum separation tubes without anticoagulant, followed by centrifugation to remove clots and cellular components. Serum samples (90 μL) were incubated with 10 μL of either control or SNPs/LNPs formulated as described in Section 4.1.4, (final mRNA concentration: 2,000 ng/mL). Zymosan (5 mg/mL) was used as a positive control. PBS was used as a negative control. Samples were incubated for 1 h at 37 °C and subsequently centrifuged at 500 x g for 5 min to pellet the zymosan. Supernatants were transferred to fresh polypropylene tubes, immediately sealed, and stored at −80 °C until analysis.

On the day of analysis, serum samples were rapidly thawed at 37 °C and assayed for C3a using a Human Complement C3a ELISA (BMS2089, Invitrogen, USA), according to the manufacturer's instructions.

Complement level compared to the PBS group was calculated by the following formula:

$$Complement\ fold\ change = \frac{Experimental\ group\ C3a - PBS\ group\ C3a}{PBS\ group\ C3a}$$

7.11 SNP/LNP release of platelet activating factor (PAF)

Peripheral blood mononuclear cells (PBMCs) were isolated from fresh leukopaks obtained from healthy donors (Stemcell Technologies, MA, USA). The leukopak was diluted 1:1 with MACS/EasySep buffer (2% FBS, 2 mM EDTA in PBS) and centrifuged at 100 × g for 15 minutes with a slow brake. The supernatant was discarded, and the cell pellet was resuspended in 50 mL of MACS/EasySep buffer. The suspension was then centrifuged at 400 × g for 5 minutes, and the supernatant was removed. This washing step was repeated twice. PBMCs were counted using a 3% acetic acid solution containing methylene blue. A total of 5–10 × 10^5 cells were plated per well in 48-well plates. LNP/mRNA complexes containing 10 μg of firefly luciferase mRNA (GenScript, NJ, USA) were formulated as described in Section 4.1.4 and diluted with PBS to a total volume of 200 μL. Aliquots of 8 μL of this dilution were mixed with Opti-MEM to a final volume of 100 μL. The resulting formulations were then added to each well without removing the existing culture medium, resulting in final mRNA concentration of 2 μg/mL. PBS was used as a negative control and LPS (final concentration 100 ng/mL) was used as a positive control. Cells were incubated at 37 °C in a humidified atmosphere with 5% CO_2 for 24 hours. After incubation, the cells were pelleted by centrifugation at 1000x g for

10min at 4 °C. The supernatants were collected and stored at −80°C until analysis. PAF concentrations in the culture supernatants were quantified using a an ELISA kit (MyBioSource, Catalot# MBS2700185) according to the manufacturer's instructions. PAF release was subtracted with the background generated from the PBS group and the PAF release was quantified as described below:

$$PAF\ release = PAF_{treatment} - PAF_{PBS}$$

7.12 LDH assessment

Cell membrane integrity was assessed using a fluorescence-based lactate dehydrogenase (LDH) release assay (CyQUANT™ LDH Cytotoxicity Assay – Fluorescence Kit, Thermo Fisher Scientific, Cat. No. C20302) according to the manufacturer's instructions. HEK 293T cells were cultured in 96-well plates at a density of 10,000 cells per well in 100µL DMEM media and incubated overnight at 37 °C with 5% CO_2. Cells were treated with SNPs or LNPs (2000 ng/mL mRNA concentration). Spontaneous LDH release, maximum LDH release, and background controls were prepared in parallel. For spontaneous release controls, cells were treated with sterile water; for the maximum release group, nothing was added to the cells until the lysis step. After 24-hour incubation, the maximum LDH group was lysed by addition of 10 µL of 10x Lysis Buffer per well and incubated for 45 min at 37 °C. 50 µL of culture supernatant from each well was added to a 96-well plate. For the positive control group, 50 µL of 1X LDH Positive Control reagent was added to the 96-well plate. Reaction Mixture solution (50 µL) was added to each well and mixed gently and incubated for 10 min at room temperature, protected from light. The reaction was terminated by adding 50 µL of Stop Solution. Fluorescence was measured immediately after incubation using a microplate reader with excitation at 560 nm and emission at 590 nm. Background fluorescence from medium-only wells was subtracted from all the readings.

Percent cytotoxicity was calculated using the formula:

$$\%Cytotoxicity = \frac{LNP\ treated\ LDH - Spontaneous\ LDH}{Maximun\ LDH - Spontaneous\ LDH}$$

$$\%Viability = 100\% - \%Cytotoxicity$$

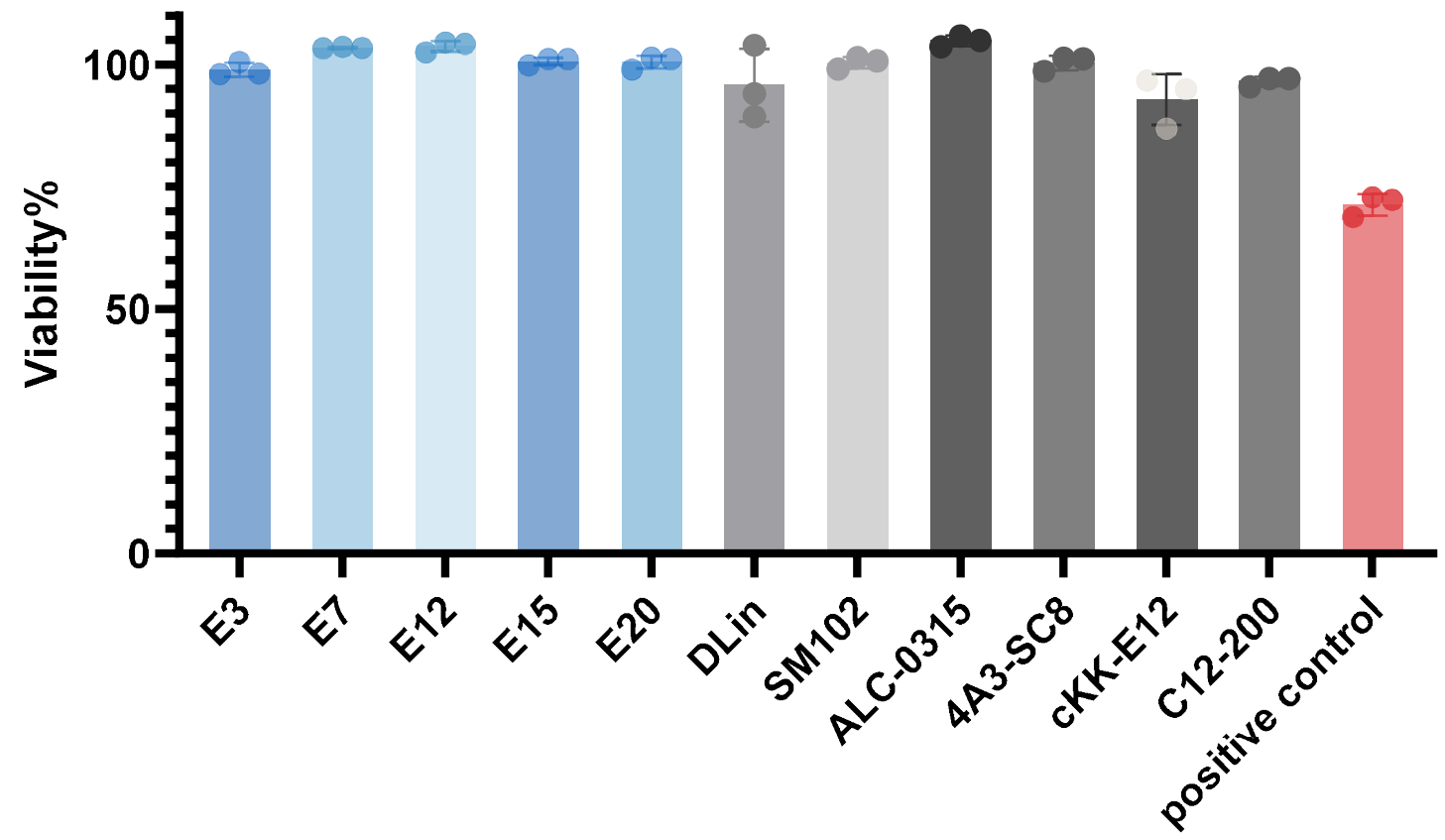

Figure S106. LNPs and SNPs do not trigger the release of LDH from cells.
LNPs and SNPs do not trigger the release of LDH from cells (n = 3 biologically independent replicates).

7.13 Endotoxin assessment

Endotoxin levels were measured using a gel-clot Limulus amebocyte lysate assay (ToxinSensor™ Gel Clot Endotoxin Assay Kit, GenScript, Cat. No. L00351) following the manufacturer's instructions. Samples or standards from the stock solutions were dissolved in endotoxin-free water. Equal volumes of sample or endotoxin standard and reconstituted LAL reagent were mixed, incubated at 37°C for 1 hour, and assessed for gel formation by inverting tubes 180°. Endotoxin concentrations were determined semi-quantitatively by comparison with the standards and reported as EU/mL. The lowest detection limit of the assay was 0.25 EU/mL.

Table S2 Endotoxin levels of LNP components measured by the gel-clot LAL assay

Lipid / Component	Endotoxin level (EU/mL)
**DOPE**	< 0.25
DSPC	< 0.25
DMG-PEG	< 0.25
Cholesterol	< 0.25
MC3-DLin	< 0.25
SM-102	< 0.25
ALC-0315	< 0.25
4A3-SC8	< 0.25
cKK-E12	< 0.25

C12-200	< 0.25
E20	< 0.25
GFP mRNA	< 0.25
Luciferase mRNA	< 0.25
Cas9 mRNA	< 0.25
EPO mRNA	< 0.25
IL22 mRNA	< 0.25
Cy5-labled mRNA	< 0.25
Luciferase Nano plasmid	< 0.25

References

1 Han, X. *et al.* In situ combinatorial synthesis of degradable branched lipidoids for systemic delivery of mRNA therapeutics and gene editors. *Nat Commun* **15**, 1762, doi:10.1038/s41467-024-45537-z (2024).

2 Hashiba, K. *et al.* Branching Ionizable Lipids Can Enhance the Stability, Fusogenicity, and Functional Delivery of mRNA (vol 03, 2200071, 2023). *Small Sci* **3**, doi:ARTN 230028510.1002/smsc.202300285 (2023).

3 Schoenmaker, L. *et al.* mRNA-lipid nanoparticle COVID-19 vaccines: Structure and stability. *Int J Pharm* **601**, 120586, doi:10.1016/j.ijpharm.2021.120586 (2021).

4 Omo-Lamai, S. *et al.* Limiting endosomal damage sensing reduces inflammation triggered by lipid nanoparticle endosomal escape. *Nat Nanotechnol* **20**, 1285-1297, doi:10.1038/s41565-025-01974-5 (2025).

5 Omo-Lamai, S. *et al.* Lipid Nanoparticle-Associated Inflammation is Triggered by Sensing of Endosomal Damage: Engineering Endosomal Escape Without Side Effects. *bioRxiv*, doi:10.1101/2024.04.16.589801 (2024).

6 Chen, Q. B. *et al.* Minimizing the ratio of ionizable lipid in lipid nanoparticles for in vivo base editing. *Natl Sci Rev* **11**, doi:ARTN nwae13510.1093/nsr/nwae135 (2024).

7 Terpstra, H. M. *et al.* PunctaFinder: An algorithm for automated spot detection in fluorescence microscopy images. *Mol Biol Cell* **35**, mr9, doi:10.1091/mbc.E24-06-0254 (2024).
