# Supplementary material for: Charge-switching ionizable lipids lower the toxicity of lipid nanoparticles
Source: Nat Nanotechnol. 2026 Sep 8;21(10):1479–88. doi: 10.1038/s41565-026-02262-6 (PMC13661284; doi:10.1038/s41565-026-02262-6)
Supplement: Supplementary file 2 [file 41565_2026_2262_MOESM2_ESM.pdf]

nature portfolio

Corresponding author(s): Niren Murthy, Hesong Han and Aijun Wang

Last updated by author(s): Jun 16, 2026

Reporting Summary

Nature Portfolio wishes to improve the reproducibility of the work that we publish. This form provides structure for consistency and transparency in reporting. For further information on Nature Portfolio policies, see our Editorial Policies and the Editorial Policy Checklist.

Statistics

For all statistical analyses, confirm that the following items are present in the figure legend, table legend, main text, or Methods section.

n/a	Confirmed	
☐	☒	The exact sample size (*n*) for each experimental group/condition, given as a discrete number and unit of measurement
☐	☒	A statement on whether measurements were taken from distinct samples or whether the same sample was measured repeatedly
☐	☒	The statistical test(s) used AND whether they are one- or two-sided<br>*Only common tests should be described solely by name; describe more complex techniques in the Methods section.*
☒	☐	A description of all covariates tested
☒	☐	A description of any assumptions or corrections, such as tests of normality and adjustment for multiple comparisons
☐	☒	A full description of the statistical parameters including central tendency (e.g. means) or other basic estimates (e.g. regression coefficient) AND variation (e.g. standard deviation) or associated estimates of uncertainty (e.g. confidence intervals)
☐	☒	For null hypothesis testing, the test statistic (e.g. *F*, *t*, *r*) with confidence intervals, effect sizes, degrees of freedom and *P* value noted<br>*Give P values as exact values whenever suitable.*
☒	☐	For Bayesian analysis, information on the choice of priors and Markov chain Monte Carlo settings
☒	☐	For hierarchical and complex designs, identification of the appropriate level for tests and full reporting of outcomes
☒	☐	Estimates of effect sizes (e.g. Cohen's *d*, Pearson's *r*), indicating how they were calculated

Our web collection on statistics for biologists contains articles on many of the points above.

Software and code

Policy information about availability of computer code

Data collection

Malvern Zetasizer (Nano-ZS)
TECAN i-control (v. 1.6.19.2)
SIBYLS beamline (12.3.1)
Living Image software version 4.2 (Caliper Life Sciences)
EVOS M5000
LabSolutions (v. 5.98)
Advion Mass Express (v. 6.9.43.1)
MetaXpress (v.6.7.2.290)

Data analysis	Attune Cytometric Software (v. 5.1.1)<br>Chemdraw (v. 23.1.2)<br>MestReNova (v. 14.2.3-29241)<br>Advion Data Express (v. 6.9.43.1)<br>FlowJo v10 10.7.1<br>ImageJ (v. 1.54g)<br>Python (v. 3.14.0)<br>Image-Pro plus (v. 6.0.0.260)<br>Graphpad Prism (v. 10.4.1)

For manuscripts utilizing custom algorithms or software that are central to the research but not yet described in published literature, software must be made available to editors and reviewers. We strongly encourage code deposition in a community repository (e.g. GitHub). See the Nature Portfolio guidelines for submitting code & software for further information.

Data

Policy information about availability of data

All manuscripts must include a data availability statement. This statement should provide the following information, where applicable:

- Accession codes, unique identifiers, or web links for publicly available datasets
- A description of any restrictions on data availability
- For clinical datasets or third party data, please ensure that the statement adheres to our policy

The authors declare that the data supporting the findings of this study are available within the paper and its Supplementary Information files. Should any raw data files be needed in another format they are available from the corresponding author upon request.

Research involving human participants, their data, or biological material

Policy information about studies with human participants or human data. See also policy information about sex, gender (identity/presentation), and sexual orientation and race, ethnicity and racism.

Reporting on sex and gender	N/A
Reporting on race, ethnicity, or other socially relevant groupings	N/A
Population characteristics	N/A
Recruitment	N/A
Ethics oversight	N/A

Note that full information on the approval of the study protocol must also be provided in the manuscript.

Field-specific reporting

Please select the one below that is the best fit for your research. If you are not sure, read the appropriate sections before making your selection.

☒ Life sciences ☐ Behavioural & social sciences ☐ Ecological, evolutionary & environmental sciences

For a reference copy of the document with all sections, see nature.com/documents/nr-reporting-summary-flat.pdf

Life sciences study design

All studies must disclose on these points even when the disclosure is negative.

Sample size	No predetermine of sample size. Sample sizes were selected based on established practices in the field and our previous experience with similar experiments. For in vitro experiments, n = 3 biologically independent replicates were performed unless otherwise stated. For human PBMC experiments, n = 4 healthy donors were used. For in vivo experiments, n = 3 mice were used for most experiments unless otherwise stated. Fig. 3A, the in vivo luciferase mRNA initial screening study, was performed with n = 2 biological replicates. Fig. 3D, the LPS pre-treatment mRNA delivery efficiency study was performed with n = 3 biological replicates for all groups except this cKK-E12 group, which had n = 2 because one mouse died during the experiment. Sample sizes were selected to ensure data reliability and appropriate statistical analysis. Details of sample sizes are provided in the figure legends where relevant.
Data exclusions	No data were excluded.
Replication	Cell experiments were performed with n = 3 biological replicates unless otherwise stated, and the sample sizes are indicated in the figure legends. The in vitro S-lipid screening experiments in figures 2C-D were performed with n = 3 biologically independent replicates. Experiments done with HEK293 cells, fibroblasts, Gal-8 reporter cells, and red blood cells (hemolysis study) were performed with an n = 3 sample size. Experiment focus on the measurement of PAF release, TLR4 activation, and complement activation were done with an n = 3 sample size.

Human PBMC experiments were performed with n = 4 healthy donors, including the cytokine heat map in Fig. 4D. For animal experiments, mice were randomly allocated into treatment groups. The in vivo luciferase mRNA screening experiment in Fig. 3A was performed with n = 2 biological replicates, while the hEPO delivery, LPS-pretreated mouse, pDNA delivery/toxicity, long-term pDNA expression, tissue injury, cytokine, ALT, and acute lung injury experiments were performed with n = 3 biological replicates unless otherwise stated. In Fig. 3D, the LPS pretreatment experiment was performed with n = 3 biological replicates for all groups except cKK-E12 group, which had n = 2 biological replicates because one mouse died during the experiment.

Randomization	For animal experiments, mice were randomly allocated into each treatment group. For cell and PBMC experiments, samples were allocated into experimental groups randomly to reduce bias.
Blinding	The experiments were not done in a strictly blinded manner. Some of those performing the experiments were blinded (e. g., during mice injection and cell isolation). We did not blind the authors that synthesized the LNPs because the materials used in the formulations needed to be clear. When possible, the data collection and analysis were done by separate individuals.

Reporting for specific materials, systems and methods

We require information from authors about some types of materials, experimental systems and methods used in many studies. Here, indicate whether each material, system or method listed is relevant to your study. If you are not sure if a list item applies to your research, read the appropriate section before selecting a response.

Materials & experimental systems

n/a	Involved in the study	
☐	☒	Antibodies
☐	☒	Eukaryotic cell lines
☒	☐	Palaeontology and archaeology
☐	☒	Animals and other organisms
☒	☐	Clinical data
☒	☐	Dual use research of concern
☒	☐	Plants

Methods

n/a	Involved in the study	
☒	☐	ChIP-seq
☐	☒	Flow cytometry
☒	☐	MRI-based neuroimaging

Antibodies

Antibodies used	The antibodies used were FITC anti-human CD66b antibody (BioLegend, Cat. No. 305103, clone G10F5), APC anti-human CD15 antibody (BioLegend, Cat. No. 323007, clone W6D3), anti-EEA1 antibody (Abcam, Cat. No. ab109110, clone EPR4245), anti-LAMP1 antibody (Abcam, Cat. No. ab208943, clone EPR21026), and anti-Rab11A antibody (Abcam, Cat. No. ab128913, clone EPR7587(B)).
Validation	The antibodies used in this manuscript were purchased from commercial suppliers and selected based on supplier-provided validation data.<br>(1) FITC anti-human CD66b antibody (BioLegend, Cat. No. 305103, clone G10F5; supplied at 200 µg/mL) was used to identify CD66b-positive neutrophils. The antibody was purified by affinity chromatography, conjugated with FITC under optimal conditions, and quality-control tested by the supplier for flow cytometry using immunofluorescent staining followed by flow-cytometric analysis. (https://www.biolegend.com/nl-be/products/fitc-anti-human-cd66b-antibody-666?GroupID=BLG10273)<br>(2) APC anti-human CD15 antibody (BioLegend, Cat. No. 323007, clone W6D3; supplied at 100 µg/mL) was used to identify CD15-positive neutrophils. The antibody is a mouse IgG1, κ monoclonal antibody conjugated to APC. BioLegend lists this product as flow-cytometry quality tested, with each lot quality-control tested by immunofluorescent staining followed by flow-cytometric analysis. (https://www.biolegend.com/nl-be/products/apc-anti-human-cd15-ssea-1-antibody-3702)<br>(3) Anti-EEA1 antibody (Abcam, Cat. No. ab109110, clone EPR4245; supplied at 1.36 mg/mL) was used as an early endosome marker. The antibody is a rabbit monoclonal IgG that reacts with human, mouse, rat, and African green monkey samples. Abcam lists this antibody as suitable for immunocytochemistry/immunofluorescence (ICC/IF) and western blot (WB), and verified its specificity using knockout cells.(https://www.abcam.com/en-us/products/primary-antibodies/eea1-antibody-epr4245-early-endosome-marker-ab109110)<br>(4) Anti-LAMP1 antibody (Abcam, Cat. No. ab208943, clone EPR21026; supplied at 0.639 mg/mL) was used as a lysosomal marker. The antibody is a rabbit monoclonal IgG that reacts with mouse samples. Abcam lists this antibody as suitable for immunocytochemistry/immunofluorescence (ICC/IF), intracellular flow cytometry, immunohistochemistry, immunoprecipitation, and western blot. (https://www.abcam.com/en-us/products/primary-antibodies/lamp1-antibody-epr21026-lysosome-marker-ab208943)<br>(5) Anti-Rab11A antibody (Abcam, Cat. No. ab128913, clone EPR7587(B); supplied at 0.414 mg/mL) was used to detect Rab11A-associated endosomal compartment. The antibody is a rabbit monoclonal IgG that reacts with human, mouse, and rat samples. Abcam lists this antibody as suitable for western blot and immunoprecipitation; immunofluorescence use was therefore laboratory-optimized rather than supplier-validated.(https://www.abcam.com/en-us/products/primary-antibodies/rab11a-antibody-epr7587b-ab128913)

Eukaryotic cell lines

Policy information about cell lines and Sex and Gender in Research

Cell line source(s)	HEK 293T cells were purchased from the cell facility at the University of California, Berkeley; HeLa cells were purchased from the ATCC (#CCL-2); Primary mouse fibroblasts were isolated from Ai9 mice as described in Supplementary Materials section

4.4; Gal8-YFP-MDA-MB231 cells were obtained from Dr. Craig L. Duvall's lab. Human TLR4 Dual Reporter HEK 293 cells were purchased from InvivoGen (#hkd-htlr4).

Authentication	The cell lines were not further authenticated after receiving.
Mycoplasma contamination	The cell lines were not tested for mycoplasma contamination.
Commonly misidentified lines (See ICLAC register)	HEK 293T cells were used in the research, they are ideal for mRNA transfection studies due to their high transfection efficiency and rapid growth, facilitating effective gene expression studies and protein production.HeLa cells were also used, they are widely used for determining IC50 values in drug testing due to their robust growth and extensive historical data, and provide reliable and consistent results in cytotoxicity assays.

Animals and other research organisms

Policy information about studies involving animals; ARRIVE guidelines recommended for reporting animal research, and Sex and Gender in Research

Laboratory animals	Ai9 transgenic mice (007909), C57BL/6J mice (000664) and BALB/c (000651) were purchased from The Jackson Laboratory. All mice were group-housed at the University of California, Davis and the University of California, Berkeley, with a 12-h light-dark cycle with ad libitum access to food and water at 20-22 °C with 40-60% humidity. Ai9 mice 16-20 weeks old were used for this study. BALB/c and C57BL/6J mice 10-16 weeks old were used for this study.
Wild animals	No wild animals were used in this study.
Reporting on sex	Both male and female animals were used in this study. Sex was determined based on vendor-provided information and was considered during study design. Animals were randomly assigned to experimental groups, with both sexes distributed across groups when possible. Data were not analyzed separately by sex because the study was designed to evaluate treatment efficacy and toxicity rather than sex-specific effects, and the group sizes were not powered for sex-based comparisons. Therefore, the conclusions apply to both male and female animals under the conditions tested.
Field-collected samples	The study did not involve samples collected from the field.
Ethics oversight	Housing, maintenance, and animal experiments were conducted in accordance with protocols approved by the institutional animal care and use committees (IACUC) at the University of California, Davis and the University of California, Berkeley. All animal facilities used during the study period were accredited by the Association for Assessment and Accreditation of Laboratory Animal Care International.<br>(1) For animal experiments conducted at the University of California, Davis, all procedures were approved by the University of California, Davis Institutional Animal Care and Use Committee (IACUC).<br>(2) For animal experiments conducted at the University of California, Berkeley, all procedures were approved by the University of California, Berkeley Institutional Animal Care and Use Committee (IACUC).

Note that full information on the approval of the study protocol must also be provided in the manuscript.

Plants

Seed stocks	N/A
Novel plant genotypes	N/A
Authentication	N/A

Flow Cytometry

Plots

Confirm that:

- ☒ The axis labels state the marker and fluorochrome used (e.g. CD4-FITC).
- ☒ The axis scales are clearly visible. Include numbers along axes only for bottom left plot of group (a 'group' is an analysis of identical markers).
- ☒ All plots are contour plots with outliers or pseudocolor plots.
- ☒ A numerical value for number of cells or percentage (with statistics) is provided.

Methodology

Sample preparation	For HEK293T cells, the cells were trypsinized, quenched in medium, pelleted, and resuspended in PBS before flow cytometry analysis.<br>For primary mouse fibroblasts, the cells were washed with PBS (pH 7.4), detached with Accutase® solution for 10 min at 37 ° C, pelleted, and resuspended in PBS before flow cytometry analysis.<br>For PBMCs, cells were stained with fluorophore-conjugated anti-human CD66b antibodies (BioLegend Cat.No. 305103) and APC anti-human CD15 antibodies (BioLegend Cat. No. 323007) according to the manufacturer's instructions. Live/Dead staining was performed by GhostDye 780. After staining, cells were washed and analyzed by flow cytometry.<br>For PBMC mRNA uptake experiments, cells were centrifuged at 1000g for 10 min and washed three times with 200 µL PBS (pH 7.4). Cy5 fluorescence was then quantified by flow cytometry to assess cellular uptake.
Instrument	Attune NxT acoustic focusing cytometer
Software	FlowJo v10 10.7.1
Cell population abundance	Not applicable. Human PBMCs were used without sorting into specific cell populations. Therefore, post-sort purity was not determined.
Gating strategy	1. For GFP expression analysis, debris was excluded based on FSC-A/SSC-A. Single cells were gated using FSC-A/FSC-H, and GFP-positive cells were quantified based on GFP-A fluorescence.<br>2. For the neutrophil analysis, PBMCs were first gated by FSC/SSC to exclude debris. Live cells were then identified as Live/Dead dye-negative cells. Neutrophils were defined as $CD15^{+}CD66b^{+}$ cells.<br>3. For PBMC uptake studies, total PBMCs were gated by FSC/SSC to exclude debris, followed by singlet gating using FSC-A/FSC-H. mRNA uptake was quantified based on the $Cy5^{+}$ cell population.

☒ Tick this box to confirm that a figure exemplifying the gating strategy is provided in the Supplementary Information.
